# Palladium-Catalyzed Electrooxidative Double C–H Arylation

**DOI:** 10.1021/jacs.3c08479

**Published:** 2023-12-27

**Authors:** Zhipeng Lin, João C. A. Oliveira, Alexej Scheremetjew, Lutz Ackermann

**Affiliations:** † Institut für Organische und Biomolekulare Chemie, Georg-August-Universität Göttingen, Tammannstraße 2, 37077 Göttingen, Germany; ‡ Wöhler Research Institute for Sustainable Chemistry (WISCh), Georg-August-Universität Göttingen, Tammannstraße 2, 37077 Göttingen, Germany

## Abstract

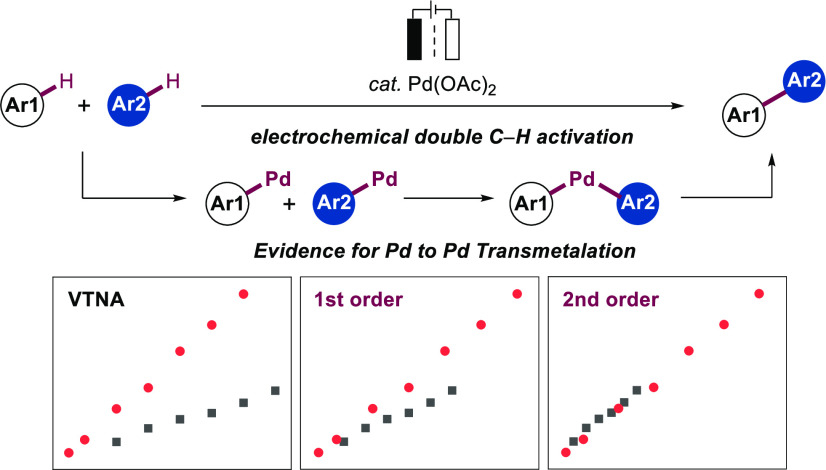

The electrochemical transition metal-catalyzed cross-dehydrogenative reaction has emerged as a promising platform to achieve a sustainable and atom-economic organic synthesis that avoids hazardous oxidants and minimizes undesired byproducts and circuitous functional group operations. However, a poor mechanistic understanding still prevents the widespread adoption of this strategy. In this regard, we herein present an electrochemical palladium-catalyzed oxidative coupling strategy to access biaryls in the absence of a stoichiometric chemical oxidant. The robust palladaelectrocatalysis considerably suppresses the occurrence of homocoupling and oxygenation, being compatible even with electron-deficient arenes. Late-stage functionalization and Boscalid precursor synthesis further highlighted the practical importance of our electrolysis. Remarkably, mechanistic studies including the evaluation of the reaction order of each component by variable time normalization analysis (VTNA) and initial rate analysis, H/D exchange experiment, kinetic isotope effect, and stoichiometric organometallic experiments provided strong support for the involvement of transmetalation between two organopalladium complexes in the turnover limiting step. Therefore, matching the concentrations or lifetimes of two distinct organopalladium intermediates is revealed to be a pivot to the success of electrooxidative catalysis. Moreover, the presence of cationic copper(II) seems to contribute to the stabilization of the palladium(0) catalyst instead of playing a role in the oxidation of the catalyst.

## Introduction

**Figure 1:**
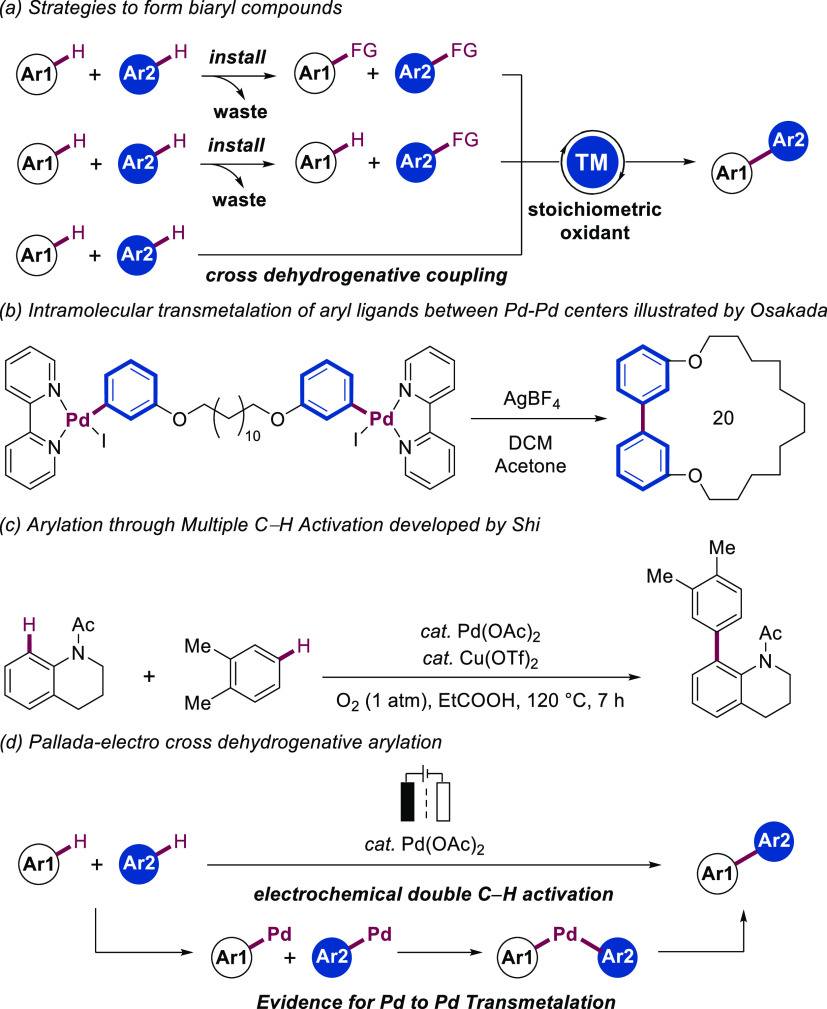
Novel electrochemical cross-dehydrogenative coupling.

Biaryl scaffolds represent an important class of structural motifs embedded in non-natural pharmaceuticals, agrochemicals, ligands, and π-conjugated materials.^1^ Conventional halogen- and organometal-based cross-coupling reactions that access biaryls usually generate superstoichiometric chemical waste through multiple functional group manipulations.^2^ In sharp contrast, palladium-catalyzed cross-dehydrogenative coupling^3^ of simple arenes has emerged as a direct and rapid avenue in line with an atom-economic and green synthesis (Figure 1a). Significant progress has been made in palladium-catalyzed double C–H activation for biaryl formation since the seminal works by *inter alia* Lu, Fagnou, Deboef, and Sanford.^4^ Nevertheless, challenges such as reduced catalytic efficacy, limited substrate scope, and byproduct formation have raised intriguing questions about the mechanism of such transformation.^5^

Mechanistically, a commonly accepted rational catalytic cycle involves one palladium(II) center, which undergoes two sequential C–H cleavages via concerted metalation deprotonation (CMD),^6^ base-assisted internal electrophilic substitution (BIES),^7^ σ-bond metathesis,^8^ or electrophilic metalation. However, high-energy barriers have been associated with such elementary steps, casting doubt on the reaction mechanism.^9^ Alternatively, organometallic reactions built on a Pd(II)/Pd(IV) redox system^10^ have been explored as a feasible platform for addressing such unfavored energetic limitations. In this context, Sanford, Michael, and Yu, among others, put forward C–H activation at palladium(IV) species to provide distinguished selectivity and functional group tolerance under mild conditions.^11^ As an alternative, catalysis involving binuclear palladium(III) was reported by Ritter,^12^ providing new insights into palladium catalysis. Remarkably, mechanistic studies by Echavarren pointed to transmetalation-type reactions between palladium(II) complexes being more facile than a Pd(II)/Pd(IV) redox cycle within the Catellani regime.^13^ Although the Pd-to-Pd transmetalation was recognized by Davidson and Triggs as early as 1968,^14^ few reports have provided experimental evidence for such a pathway.^15^ In 2003, Osakada elegantly illustrated an aryl transmetalation process via an intramolecular ligand exchange (Figure 1b).^16^ Additionally, Hartwig and Stahl conducted detailed mechanistic studies for the Pd–Pd cooperative modus operandi for the direct arylation of aryl halide and the homocoupling of xylene, respectively.^9b,17^

Recently, the merger of transition metal-catalyzed C–H activation^18^ and electro-organic synthesis^19^ has surfaced as a uniquely effective approach for sustainable molecular synthesis.^20^ Harnessing the advantages of replacing toxic and undesirable stoichiometric chemical oxidants with electricity, our group has significantly contributed to the progress on electrochemical C–H activation catalyzed by 3d-, 4d-, and 5d-metals.^21^ Referring to palladaelectrocatalysis,^22^ we have extended the scope of oxidative coupling to asymmetric catalysis^23^ and undirected C–H olefination.^24^ However, to the best of our knowledge, biaryl formation via electrochemical palladium-catalyzed double C–H activation has proven elusive.

Herein, inspired by the elegant multiple C–H activation developed by Shi (Figure 1c),^5o^ we report on a novel electrochemical palladium-catalyzed cross-dehydrogenative transformation for the synthesis of biaryl devoid of stoichiometric chemical oxidant and prefunctionalized fragments (Figure 1d). The electrooxidative conditions exhibit broad applicability, including electron-deficient arenes. Late-stage functionalization as well as Boscalid precursor synthesis has been proved feasible under our electrolysis conditions. Notably, a rare bimetallic mechanism featuring a Pd-to-Pd aryl transfer process as the turnover limiting step was disclosed. Mechanistic studies comprising reaction order studies by VTNA and initial rate analysis, isotope experiments, and stoichiometric organometallic reactions provided strong support for a bimetallic Pd-to-Pd transmetalation mechanism. Moreover, Cu(OTf)_2_ seems to be crucial for the stabilization of palladium(0) intermediates rather than participating in the oxidation of catalysts.^5o,25^

## Results and Discussion

**Scheme 1:**
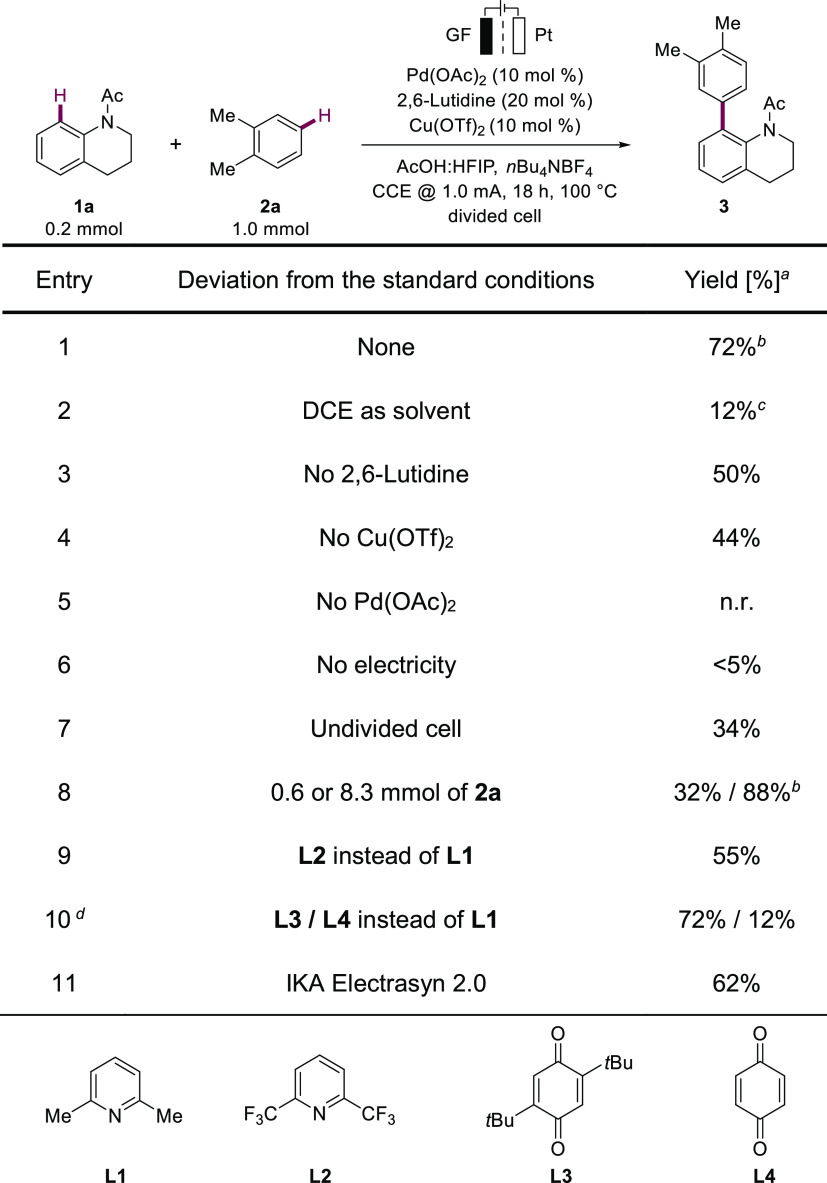
Control Experiments for Palladaelectro Cross-Dehydrogenative Arylation General reaction conditions: divided cell, anodic chamber: **1a** (0.20 mmol), **2a** (1.0 mmol), Pd(OAc)_2_ (10 mol %), 2.6-lutidine (20 mol %), Cu(OTf)_2_ (10 mol %), *n*Bu_4_NBF_4_ (40 mg), HFIP/AcOH (1.0 mL: 2.0 mL), cathodic chamber: **2a** (1.0 mmol), *n*Bu_4_NBF_4_ (40 mg), HFIP/AcOH (1.0 mL: 2.0 mL), 100 °C, electrolysis (CCE) at 1.0 mA, 18 h, graphite felt (GF) anode (10 mm × 15 mm × 2 mm), Pt plate cathode (10 mm × 15 mm × 0.25 mm), NMR yields using CH_2_Br_2_ as an internal standard. Isolated yield. 80 °C. Without Cu(OTf)_2_.

We initiated our studies for the envisioned electrochemical dual C–H activation using *N*-acetyltetrahydroquinoline (**1a**) and *o*-xylene (**2a**) as substrates in a divided cell setup (Scheme 1, Entry 1). Using dichloroethane (DCE) as the solvent resulted in a drastic reduction in the yield of product **3** (Entry 2). Similarly, changing the solvent ratio led to a drop in performance, highlighting the H-bonding donor ability of HFIP on stabilizing intermediates (Supplementary Table 7).^26^ The metallaelectrocatalysis occurred in the absence of Cu(OTf)_2_ or 2,6-lutidine, whereas when present in catalytic amounts, an improvement in the turnover number and robustness was observed (entries 3 and 4). Control experiments revealed the indispensable role of both the palladium catalyst and the electricity in the electrooxidative double C–H arylation (entries 5 and 6). A divided cell electrolyzer was beneficial to provide good reactivity and chemoselectivity. (Entry 7 and Supplementary Table 4).^27^ Further optimization demonstrated that adjusting the stoichiometry of reactant **2a** had a substantial influence on the isolated yield (Entry 8). In addition, 2,6-bis(trifluoromethyl)pyridine (**L2**) was found to be an inferior substitute for lutidine (Entry 9). Interestingly, similar reaction efficiency was obtained when replacing Cu(OTf)_2_ and lutidine with 2,6-di-*tert*-butyl benzoquinone (**L3**; Entry 10 and Supplementary Figure 9).^28^

**Scheme 2:**
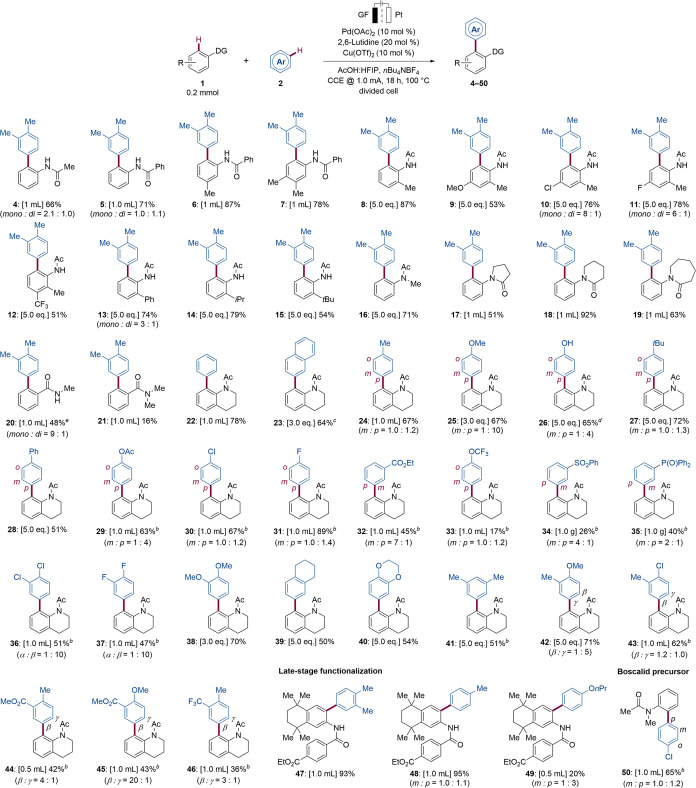
Versatility for Electrochemical Palladium-Catalyzed Double C–H Activation Standard reaction conditions. The amount of arenes used is indicated. See SI for more experimental details. 20 mol % Pd(OAc)_2_, 0.5 mL of TFA as a cosolvent, 90 °C. As an isomeric mixture. Triisopropyl(phenoxy)silane as a substrate. 20 mol % Pd(OAc)_2_ was used.

With the optimized reaction conditions in hand, we explored the versatility of electro-oxidation (Scheme 2). We were pleased to find that a wide variety of functional groups involving labile halides and potential Shono-type oxidation alkylated amide motifs were compatible with the robust palladaelectrocatalysis. Acetanilide (**1b**) and benzanilide (**1c**) provided both mono- and bis-arylated products **4** and **5**, respectively. Anilide derivatives bearing a methyl group on the *m-*position significantly inhibited the formation of difunctionalized products, thus delivering monoarylated products **6** and **7** with excellent site selectivity. When a wide range of *o*-functionalities were introduced into the acetanilides, a significant improvement in reactivity was observed when compared to those bearing no *o*-substituents (**8**–**15**). The trifluoromethyl group was also identified as a compatible moiety, thus affording product **12** in moderate yield. Notably, *N*-methylacetanilide **1n** furnished solely monoarylated products **16**, possibly due to steric effects. Furthermore, substrates containing different ring-size directing groups like pyrrolidinone (**1o**), piperidinone (**1p**), and azepinone (**1q**) were also converted, affording uniquely monoarylated products **17**–**19**. Next, we explored substrates equipped with alternative directing groups. Gratifyingly, *N*-methylbenzamide and *N,N*-dimethylbenzamide were compatible under the reaction conditions (**20**–**21**). Unfortunately, carboxylic acid did not mirror the reactivity and was unable to afford the desired product.

Thereafter, we examined the scope of directing-group-free arenes **2** in the electrocatalysis (Scheme 2). A set of electronically diverse arenes **2** were compatible with the robust electrochemical conditions, providing products **22**–**46** in moderate-to-excellent yields. Hence, benzene (**2b**) and naphthalene (**2c**) were tested, giving good yields for the respective arylated products (**22** and **23**). Likewise, toluene (**2d**) and anisole (**2e**) were suitable substrates, providing *p*-arylated products **24** and **25** as major regioisomers. Interestingly, in situ deprotected product **26** was observed when triisopropyl(phenoxy)silane (**2f**) was subjected to the reaction conditions. Phenylacetate (**2i**) was also found to be a suitable substrate, furnishing the desired product (**29**) in good yield. Notably, electronically deficient arenes were compatible under the electrolysis conditions in conjugation with trifluoroacetyl (TFA) as a cosolvent, providing the desired biaryls (**30**–**37**) in low-to-excellent yields. Here, TFA was thought to accelerate the C–H activation of electron-poor arenes,^5k^ while for electron-rich arenes, it led to the formation of a homocoupling product as the major product. Unfortunately, bromobenzene was not tolerated under electrocatalysis conditions. Veratrol (**2r**) and 2,3-dihydrobenzo[*b*][1,4]dioxine (**2t**) were identified as amenable substrates; however, we observed that arenes with higher electron densities such as 1,3,5-trimethoxybenzene usually delivered the self-polymerized product. Furthermore, 1,3-disubstituted and asymmetrical 1,2-disubstituted arenes were selectively functionalized, affording products **41**–**46**. We have applied our electrochemical methodology to late-stage diversification of Tamibarotene ester, affording a series of arylated products (**47**–**49**) in excellent yields. Furthermore, the precursor (**50**) for Boscalid was successfully furnished by our electrocatalysis. However, the presence of the methyl group on the *N*-center was revealed to be necessary for the reactivity to unwind.

Hitherto, our preliminary studies of the electrochemical palladium-catalyzed cross-dehydrogenative arylation left several key questions unanswered. First, the failure of bromobenzene as a starting material and the observation of palladium black jeopardized the proposal of Pd(IV) in our mechanism. Second, low yields for the desired products were usually associated with the homocoupling of simple arenes, which surpassed the cross arylation. Third, the catalytic efficiency was sensitive to the concentration of the palladium catalyst. These questions motivated us to explore the reaction mechanism in detail.

**Figure 2:**
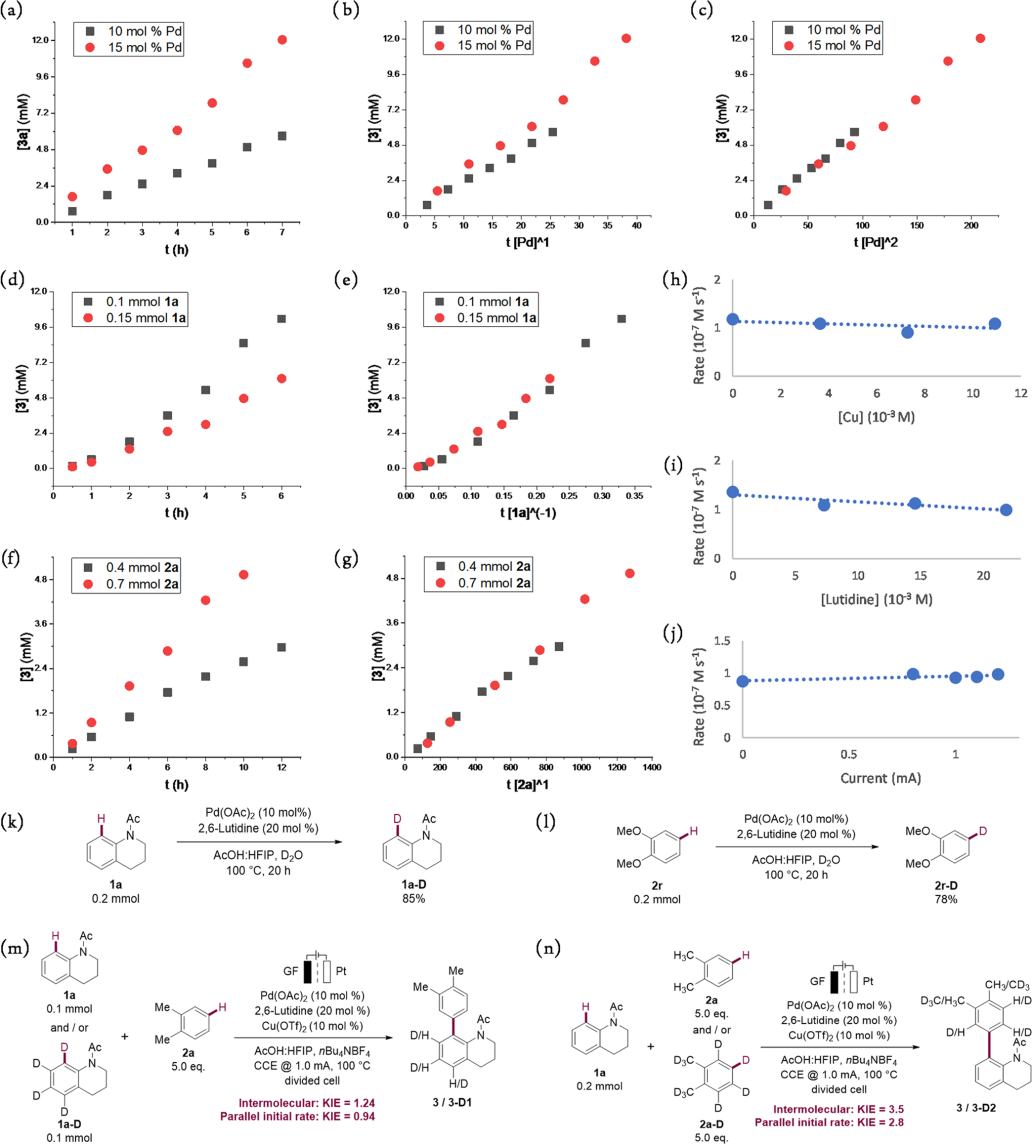
Mechanistic studies; see the Supporting Information for more reaction details. (a–g) VTNA analysis using *o*-xylene as a substrate. (h–j) Initial rate studies using 1,2-dichlorobenzene as a substrate. (k, l) H/D exchange experiment. Dideuterated dimethoxybenzene could be a possible product. (m, n) Kinetic isotope effect.

We began our mechanistic interrogation by determining the turnover limiting step through kinetic studies (Figure 2). The kinetic order of the reaction components was determined by using variable time normalization analysis (VTNA) derived from the reaction progress kinetic analysis developed by Blackmond.^29^Figure 2a shows the kinetic profile of the reaction with two different Pd(OAc)_2_ concentrations, where two distinct slopes were observed. When the two profiles were replotted as product concentration versus normalized time scale by a first-order factor of catalyst concentration (t [Pd]^1^), two reaction progress curves failed to overlap (Figure 2b) until a second-order correlation was used (Figure 2c), indicating that the kinetic order for palladium is the second order rather than the first order. The observation of [Pd]^2^ suggests that two intramolecular or intermolecular palladium nuclei are involved in the turnover limiting step. Next, inferior reaction progress was observed when increasing substrate **1a** loading (Figure 2d,e), corresponding to an inverse first order. Therefore, we hypothesized that losing 1 equiv of **1a** from a palladium off-cycle species is necessary to activate the catalyst.^30^ Likewise, an experimental order of one for substrate **2a** was obtained by using an analogous procedure (Figure 2f,g). To further corroborate the kinetic data obtained from VTNA, we conducted initial rate analyses using dichlorobenzene (**2p**) as the substrate (see the SI, Section 7.2). As a result, the kinetic orders obtained from VTNA were supported by the supplementary initial rate analyses, suggesting that the reaction pathway of electron-poor arenes is relevant to the mechanism of the reaction of electron-rich arenes. In addition, initial rate analyses pointed at zero kinetic orders for copper, lutidine, and current (Figure 2h–j). Notably, identical initial rates were obtained even in the absence of these components, supporting the idea that copper, lutidine, and electricity are solely involved in the regeneration of the active catalyst. Based on the above mechanistic findings, we assumed that 2 equiv of palladium, 1 equiv of **1a**, and 1 equiv of **2a** were involved in the rate-determining step. With this in mind, two possible reaction pathway candidates could be accounted for: a Pd(II)-to-Pd(II) transmetalation mechanism or a previously reported bimetallic Pd(IV) manifold.^30^ However, the transmetalation pathway seems to be a more plausible pathway over the dimeric palladium catalyst for three reasons: (1) electricity and Cu(OTf)_2_ were not involved in the oxidation of Pd(II) to Pd(IV), (2) the dimeric catalyst was generally considered as the precatalyst,^31^ and (3) the observed inverse first order for **1a** contradicted the described transformation between the resting-state catalyst and the dimeric palladium complex in the literature.^30^

To further assess the transmetalation mechanism based on two organopalladium complexes, it is of interest to know if the two C–H activation steps proceed before the transmetalation step or not; thus, we conducted isotope experiments to investigate the nature of the C–H activation step. H/D exchange experiments (Figure 2k,l) illustrated that for both isotopes, the yields exceeded the catalytic amount of Pd(OAc)_2_, which can be indicative of a reversible metalation for each of the substrates. Additionally, kinetic isotope effect (KIE) studies revealed a secondary KIE or no KIE for substrate **1a** and a primary KIE for substrate **2a** (Figure 2m,n), indicative of a facile C–H cleavage of **1a**, whereas the step for **2a** is slow. The observation of deuterated products and KIE are consistent with C–H cleavages occurring during the catalytic cycle but before the turnover limiting step.^32^ Moreover, the large KIE for **2a** implied that the C–H activation of **2a** could replace the transmetalation as the rate-determining step when lowering the temperature or reducing the stoichiometry in **2a**.

**Figure 3:**
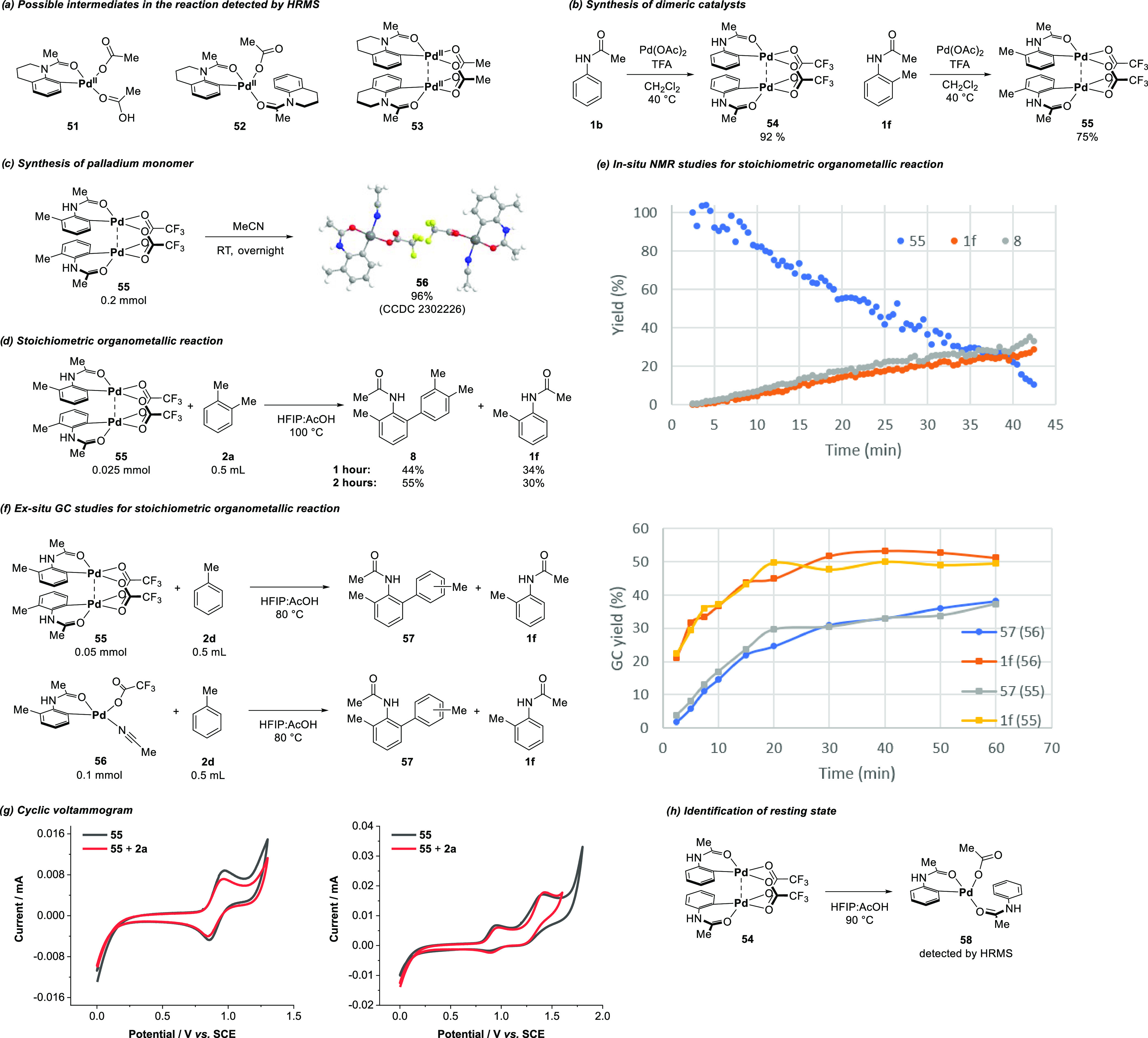
Organopalladium studies. See the SI for more details.

To further validate our finding on cooperative aryl transfer between the two palladium centers, it is necessary to identify the relations between the resting-state catalyst and the active catalyst in the rate-determining step, in particular for the organopalladium complexes with anilide. Therefore, we first conducted an HRMS analysis to detect the possible intermediates under our catalytic conditions. Three intermediates (**51**–**53**) could be postulated from the interpretation of the HRMS spectrum (Figure 3a and Supplementary Figures 64 and 65). Next, we synthesized known dimeric complexes **54** and **55** (Figure 3b) with diacetate bridges using noncoordinating dichloromethane as a solvent.^5j,5k,33^ The easy access to complexes **54** and **55** under mild conditions agrees with the observed KIE value for **1a**. Treating dimeric palladium complex **55** with MeCN at room temperature led to the formation of monomeric palladium complex **56** in near-quantitive yield (Figure 3c).^34^ The stoichiometric organometallic reaction between **55** and **2a** afforded 44% of product **8** in 1 h and 55% in 2 h (Figure 3d), implying that organopalladium **55** could presumably be a precursor for the active catalyst. The assumption was substantiated by in situ NMR studies on the reaction of **55** and xylene,^35^ where an induction period of precatalyst **55** was observed (Figure 3e and Supplementary Figure 83).^36^ Reaction profiles of complexes **55** and **56** obtained from ex situ GC measurements showed a comparable reaction rate for both intermediates (Figure 3f). When considering the fact that complex **56** was stabilized by the strongly coordinated acetonitrile, monomeric palladium was the more kinetically favored active catalyst. Additionally, DFT calculations were carried out at the B3LYP-D4/6-311+G(2d,p)-SDD+ SMD(AcOH)/B3LYP-D3(BJ)/6-31G(d,p)-LANL2DZ level of theory (see the SI, Section 7.8), revealing the C–H activation of xylene on Pd(OAc)_2_ to be energetically favorable with an energy barrier of 16.4 kcal mol^–1^. However, on a dimeric catalyst, the same elementary step proved to be more energetically disfavored with a barrier of 25.2 kcal mol^–1^. Moreover, cyclic voltammetry (CV) measurements revealed good stability for complex **55** at room temperature in HFIP/AcOH (Figure 3g). No catalytic current was observed at room temperature when adding an excess amount of **2a**, repudiating the proposal of second C–H activation at the Pd(III) or Pd(IV) center.^22d,37^ Heating complex **54** to 90 °C in the solvent mixture used for catalysis induced the occurrence of complex **58** (Figure 3h and Supplementary Figure 66). With these observations as well as our kinetic studies, we proposed that intermediate **52** could be identified as the resting state and monomer **51** was the on-cycle active catalyst.

**Figure 4:**
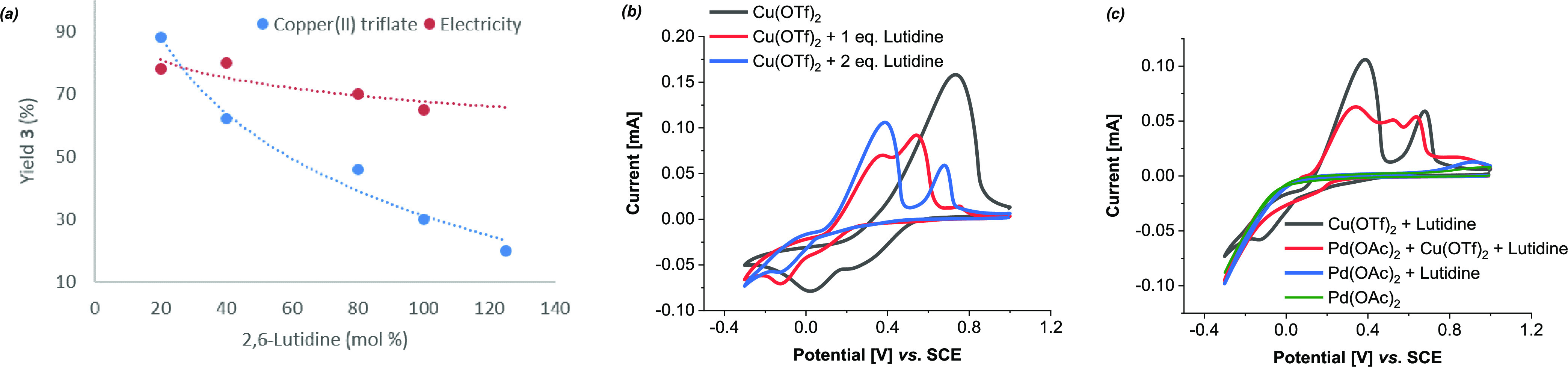
Role of Cu(OTf)_2_ and lutidine. See the SI for more details. (a) Reaction efficacy dependency on lutidine using Cu(OTf)_2_ or electricity as the oxidant. (b, c) Cyclic voltammograms. Cu(OTf)_2_ (5 mM), Pd(OAc)_2_ (5 mM), lutidine (5 or 10 mM), and 0.1 M *n*Bu_4_NBF_4_ in HFIP/AcOH (1:2), 100 mV s^–1^, room temperature.

Additionally, we turned our attention to the exact roles of Cu(OTf)_2_ and 2,6-lutidine in the regeneration of the active catalyst. When increasing the amount of 2,6-lutidine in the presence of 1 equiv of Cu(OTf)_2_ as a chemical oxidant, deterioration in the yield of product **3** from 88 to 20% was observed (Figure 4a), while the reaction using electricity as the oxidation agent retained its catalytic efficiency. This entailed a diminishing oxidative ability of Cu^2+^ in the presence of lutidine, which was supported by CV studies (Figure 4b). Moreover, the new oxidative event observed in Figure 4c when palladium and copper were mixed endorsed a heterometallic interaction. Hence, we proposed that copper salt serves as a palladium(0) stabilizer rather than a redox catalyst.^38^ Further experimentation and electroanalytical studies have been conducted (see the Supporting Information).

**Figure 5:**
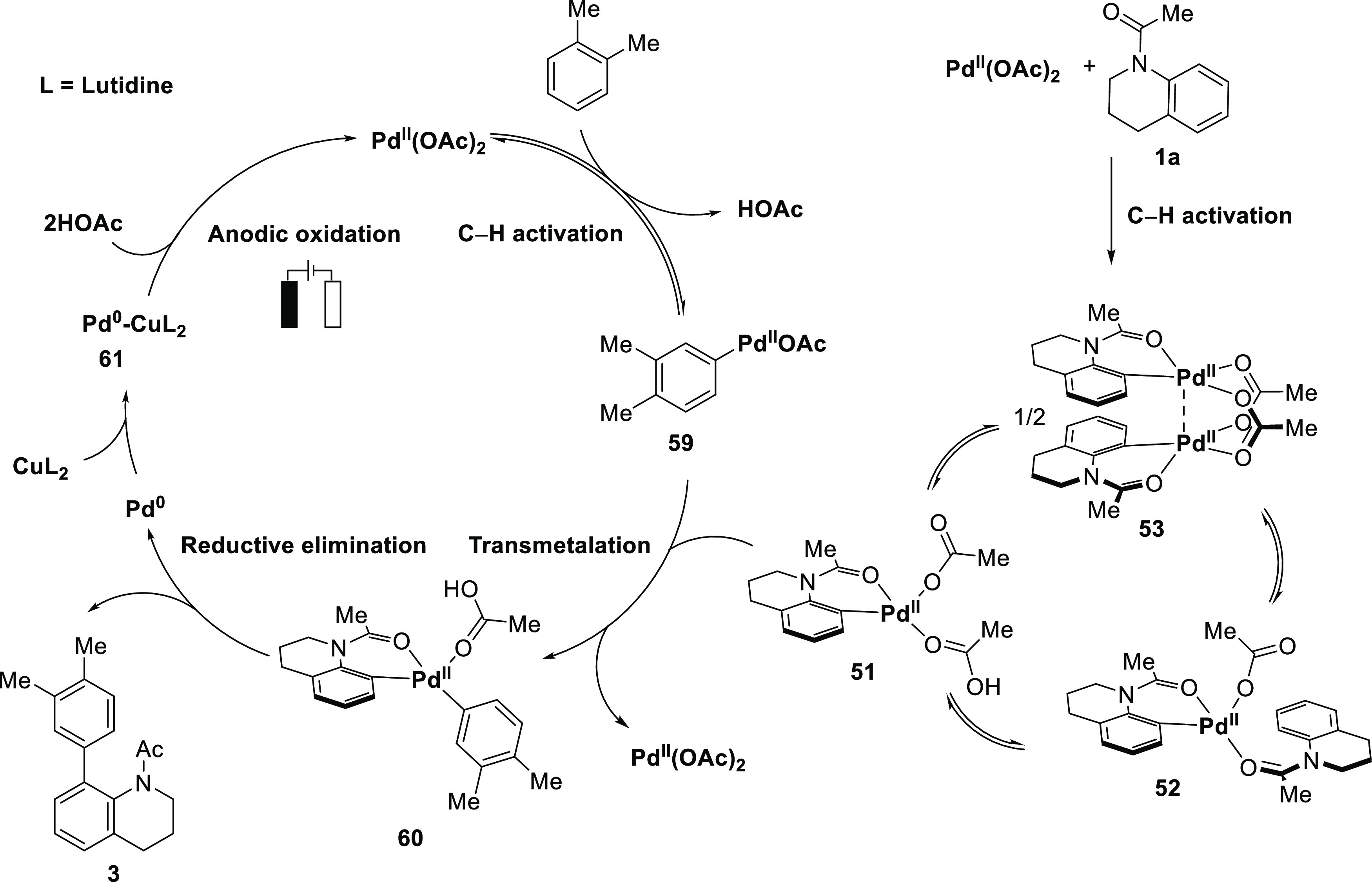
Proposed catalytic cycle.

Based on our mechanistic studies, a plausible catalytic cycle is presented in Figure 5. C–H activations of **1a** and **2a** occur concurrently, giving rise to complexes **51** and **59**, respectively. Here, dimeric catalyst **53** is considered a precatalyst for monomeric palladacycle **51**. Off-cycle species **52** could exist in a different level of concentration depending on the ratio of substrate **1** and Pd(OAc)_2_. Then, intermolecular transmetalation of **51** to **59** affords **60**, followed by reductive elimination, giving desired product **3**. The transmetalation between two organopalladium complexes was determined to be the turnover limiting step of the overall reaction. The generated Pd(0) during product formation is then stabilized by Cu complexes, which through anodic oxidation form active Pd(II), thus closing the catalytic cycle.

In summary, we have reported on versatile electrochemical palladium-catalyzed oxidative double C–H arylation without chemical oxidants. The robust electrolysis condition exhibited extraordinary reactivity; thus, a variety of arenes including electron-deficient arenes were compatible. Late-stage functionalization highlighted the synthetic value of our methodology. In addition, detailed mechanistic studies were conducted, thus supporting a bimetallic mechanism involving a transmetalation process as the rate-determining step.

## Supporting Information Available

Experimental procedures and compound characterization data including ^1^H and ^13^C NMR spectra and kinetic analyses (PDF)

## Abbreviations

CMD: concerted metalation deprotonation
DCE: dichloroethane
HFIP: hexafluorisopropanol
VTNA: variable time normalization analysis
KIE: kinetic isotope effect
CV: cyclic voltammogram
HRMS: high-resolution mass spectrometry
NMR: nuclear magnetic resonance
CDA: cross-dehydrogenative arylation
CDC: cross-dehydrogenative coupling
DFT: density functional theory
