# Supplementary material for: Palladium-Catalyzed Electrooxidative Double C–H Arylation
Source: J Am Chem Soc. 2023 Dec 27;146(1):228–39. doi: 10.1021/jacs.3c08479 (PMC10785825; doi:10.1021/jacs.3c08479)
Supplement: Supplementary file 1 — ja3c08479_si_001.pdf [file ja3c08479_si_001.pdf]

Supporting Information

Palladium-Catalyzed Electrooxidative Double C–H Arylation

Zhipeng Lin,[†,‡] João C. A. Oliveira,[†,‡] Alexej Scheremetjew,[†,‡] and Lutz Ackermann[†,‡,*]

[†] Institut für Organische und Biomolekulare Chemie, Georg-August-Universität Göttingen, Tammannstraße 2, 37077, Göttingen, Germany

[‡] Wöhler Research Institute for Sustainable Chemistry (WISCh), Georg-August-Universität Göttingen, Tammannstraße 2, 37077, Göttingen, Germany

Contents

1 General Remarks 5
2 General Procedure A: Electrochemical Cross Dehydrogenative Coupling 6
3 General Procedure B: Electrochemical Cross Dehydrogenative Coupling 7
4 Starting Material Syntheses 8
4.1 Acetylation of Aniline Derivatives 8
4.2 Buchwald-Hartwig Amination 8
4.3 Syntheses of *N*-methylacetanilide 9
4.4 Syntheses of *N*-arylbenzamide 9
4.5 Esterification of Tamibarotene 9
5 Reaction Set-up 10
6 Supplementary Reaction Information 12
6.1 Effect of Cu Salt and Lutidine 12
6.2 Role of Electricity 14
6.3 Role of Divided Cell 15
6.4 Quality of Divided Cell 16
6.5 Role of Temperature 17
6.6 Role of Xylene Stoichiometry 18
6.7 Role of HFIP 19
6.8 Role of Ligand 21
6.9 Reaction Cell Voltage 22
6.10 Reaction Using IKA Potentiostat 23
7 Supplementary Mechanistic Studies 24
7.1 VTNA 24
7.1.1 VTNA Studies on [Pd] 24
7.1.2 VTNA Studies on [**1a**] 27
7.1.3 VTNA Studies on [**2a**] 28

7.2 Initial Rate Analysis 29
7.2.1 Kinetic Dependence of Initial Rate on [Pd] 29
7.2.2 Kinetic Dependence of Initial Rate on Electricity 33
7.2.3 Kinetic Dependence of Initial Rate on [Cu] 36
7.2.4 Kinetic Dependence of Initial Rate on [Lutidine] 39
7.2.5 Kinetic Dependence of Initial Rate on [**1a**] 42
7.2.6 Kinetic Dependence of Initial Rate on [**2p**] 46
7.2.7 Kinetic Dependence of Initial Rate on Temperature 50
7.3 H/D Exchange 53
7.4 Kinetic Isotope Effect 57
7.4.1 KIE for **2a** 57
7.4.2 KIE for **1a** 59
7.5 Organopalladium Complex 61
7.5.1 Intermediates under Catalytic Conditions 61
7.5.2 Synthesis of Palladium Complex 63
7.5.3 Intermediates in Stoichiometric Organopalladium Reaction 63
7.5.4 Stoichiometric Organopalladium Reaction with Substrate **2a** 65
7.6 Cyclic Voltammograms 66
7.7 Organometallic Reaction Profile 70
7.7.1 Pre-experiments 70
7.7.2 In-situ NMR Analysis 73
7.7.3 Ex-situ GC Measurements 79
7.8 Computational Studies 80
7.8.1 Cartesian coordinates of the optimized structures 85
8 Crystallographic Data 100
9 Unsuccessful Simple Arenes 110
10 Boscalid 111

10.1 Synthetic Route 111

10.2 Prices of the chemicals 111

10.3 *N*-demethylation 111

11 Additional Studies for the Role of Copper 113

11.1 CV studies 113

11.2 Reaction Profiles 118

12 Characterization 120

13 NMR Spectrum 154

14 Reference 221

1 General Remarks

Catalytic reactions were carried out in a divided electrochemical cell using pre-dried glassware, arenes **1** or **2**, and ligands (**L1-L4**) were used as obtained by commercial sources, if not noted otherwise. Other chemicals were obtained from commercial sources and were used without further purification. Platinum electrodes (10 mm × 15 mm × 0.25 mm, 99.9%; obtained from ChemPur® Karlsruhe, Germany) and Graphite felt (GF) electrodes (10 mm × 15 mm × 6 mm, SIGRACELL®GFA 6 EA, obtained from SGL Carbon, Wiesbaden, Germany) were connected using stainless steel adapters. Electrocatalysis was conducted using a Metrohm MULTI AUTOLAB M204 potentiostat or ROHDE & SCHWARZ HMP4040 Potentiostat in two-electrode constant current mode. Yields refer to isolated compounds, estimated to be >95% pure as determined by ^{1}H-NMR. Chromatography: Merck silica gel 60 (40–63 µm). NMR: Spectra were recorded on a Varian Unity 300, Mercury 300, Inova 500 or Bruker Avance III 300, Bruker Avance III HD 400 and Bruker Avance III HD 500 in the solvent indicated; chemical shifts (δ) are given in ppm relative to the residual solvent peak. All IR spectra were recorded on a Bruker FT-IR Alpha device. MS: EI-MS- and ESI-MS-spectra were recorded with Finnigan MAT 95, 70 eV and Finnigan LCQ; High resolution mass spectrometry (HRMS) with APEX IV 7T FTICR. M. p.: Stuart melting point apparatus SMP3, Barloworld Scientific, values are uncorrected. Cyclic voltammograms were recorded on Metro*hm* Autolab PGSTAT204 potentiostat.

2 General Procedure A: Electrochemical Cross Dehydrogenative Coupling

The electrocatalysis was carried out in a pre-dried divided cell, with a GF anode (10 mm × 15 mm × 6 mm) and a platinum cathode (10 mm × 15 mm × 0.25 mm). arene **1** (0.20 mmol, 1.0 equiv.), arene **2** (1.0 mmol, 5.0 equiv.), $Pd(OAc)_2$ (4.5 mg, 10 mol %), $Cu(OTf)_2$ (7.2 mg, 10 mol %), 2,6-lutidine (4.6 µL, 20 mol %) and $n$$Bu_4NBF_4$ (40.0 mg, 0.61 equiv.) were placed in the anodic chamber and dissolved in AcOH (2.6 mL) and HFIP (1.3 mL). arene **2** (1.0 mmol, 5.0 equiv.) and $n$$Bu_4NBF_4$ (40.0 mg, 0.61 equiv.) were placed in the cathodic chamber and dissolved in AcOH (2.6 mL) and HFIP (1.3 mL). Electrocatalysis was performed at 100 °C with a constant current of 1.0 mA and a stirring rate of 500 rpm for 20 h. At ambient temperature, the reaction mixture was diluted with EtOAc (5.0 mL). The GF anode was washed with EtOAc (3 × 10 mL) in an ultrasonic bath and the washings were added to the reaction mixture. The resulting mixture was loaded in a separating funnel with 50 mL sat. Na_2CO_3 solution and extracted with EtOAc (3 × 20 mL), then the combined organic phase was washed with brine (2 × 30 mL). The combined organic layer was dried over Na_2SO_4, filtered, and the solvent was removed *in vacuo*. CH_2Br_2 (14.0 µL, 0.20 mmol, 1.0 equiv.) was added as the internal standard to determine the NMR yield. The crude mixture was purified by flash column chromatography on silica gel to yield the products **3**–**50**.

3 General Procedure B: Electrochemical Cross Dehydrogenative Coupling

The electrocatalysis was carried out in a pre-dried divided cell, with a GF anode (10 mm × 15 mm × 6 mm) and a platinum cathode (10 mm × 15 mm × 0.25 mm). arene **1** (0.20 mmol, 1.0 equiv.), arene **2** (1.0 mL), $Pd(OAc)_2$ (4.5 mg, 10 mol %), $Cu(OTf)_2$ (7.2 mg, 10 mol %), 2,6-lutidine (4.6 µL, 20 mol %) and $n Bu_4NBF_4$ (200 mg, 3.0 equiv.) were placed in the anodic chamber and dissolved in AcOH (2.0 mL), HFIP (1.0 mL) and TFA (0.50 mL). arene **2** (1.0 mL) and $n Bu_4NBF_4$ (200 mg, 3.0 equiv.) were placed in the cathodic chamber and dissolved in AcOH (2.0 mL), HFIP (1.0 mL) and TFA (0.50 mL). Electrocatalysis was performed at 90 °C with a constant current of 1.0 mA and a stirring rate of 500 rpm for 20 h. At ambient temperature, the reaction mixture was diluted with EtOAc (5.0 mL). The GF anode was washed with EtOAc (3 × 10 mL) in an ultrasonic bath and the washings were added to the reaction mixture. The resulting mixture was loaded in a separating funnel with 50 mL sat. Na_2CO_3 solution and extracted with EtOAc (3 × 20 mL), then the combined organic phase was washed with brine (2 × 30 mL). The combined organic layer was dried over Na_2SO_4, filtered, and the solvent was removed *in vacuo*. CH_2Br_2 (14.0 µL, 0.20 mmol, 1.0 equiv.) was added as the internal standard to determine the NMR yield. The crude mixture was purified by flash column chromatography on silica gel to yield the products **3**–**50**.

4 Starting Material Syntheses

4.1 Acetylation of Aniline Derivatives

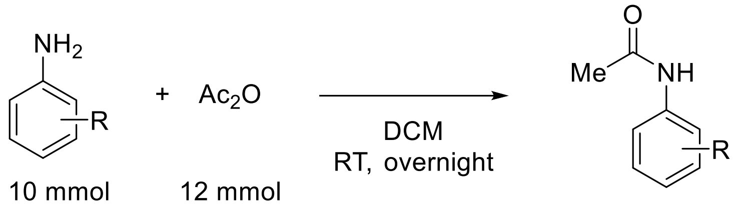

Supplementary Figure 1 Acetylation of Aniline

Aniline (10.0 mmol), DCM (20 mL) were loaded to a dry flask. Then acetic acid anhydride (12.0 mmol, 1.2 equiv.) was added dropwise at 0 °C. The reaction mixture was allowed to warm to room temperature and stirred overnight. At ambient temperature, the reaction mixture was quenched with sat. $NaHCO_3$ solution (30 mL) and extracted with DCM (3 × 20 mL). The organic layer was dried over anhydrous Na_2SO_4, filtered and removed the solvent in *vacuo*. Purification by column chromatography on silica gel provided the product.

4.2 Buchwald-Hartwig Amination

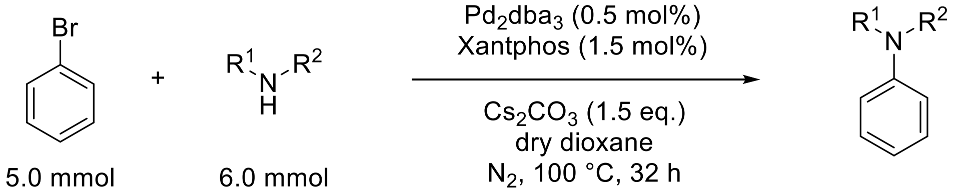

Supplementary Figure 2 Buchwald-Hartwig Coupling

The Buchwald-Hartwig aminations were conducted following the procedure described in the literature.[1] Amide (6 mmol, 1.2 equiv.), Pd_2dba_3 (22.9 mg, 0.5 mol %), Xantphos (21.7 mg, 1.5 mol %), and Cs_2CO_3 (2.5 g, 1.5 equiv.) were charged in a flame-dried resealable Schlenk tube. The Schlenk tube was capped with a rubber septum. Then the Schlenk tube was evacuated and refilled with N_2 twice. Bromobenzene (0.53 mL, 5 mmol) and anhydrous 1,4-dioxane (5 ml) were added by syringe through the septum. The Schlenk tube was sealed with an additional layer of Parafilm. Then the mixture was stirred at 100 °C for 32 hours until the starting aryl halide had been completely consumed as judged by GC-MS analysis. The reaction mixture was allowed to cool to room temperature and diluted with dichloromethane (10 mL), filtered and concentrated in *vacuo*. Purification of the crude material by flash chromatography on silica gel furnished the desired product.

4.3 Syntheses of *N*-methylacetanilide

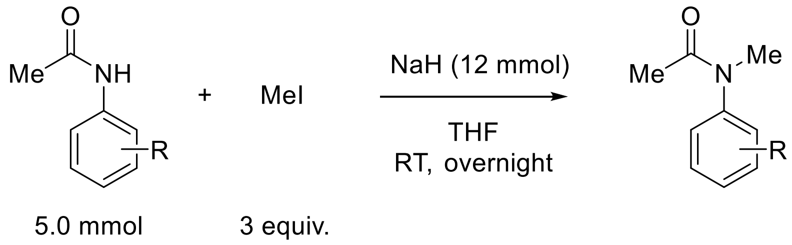

Supplementary Figure 3 Methylation of *N*-acetanilide

The syntheses of N-methylacetanilide were conducted following the procedure described in the literature.[2] To a solution of acetanilides (5.0 mmol) in dry THF (20 mL) were added NaH (60% dispersion in mineral oil, 300 mg, 1.5 equiv.) portion-wise. Then MeI (1.0 mL, 3.0 equiv.) was added to the reaction mixture. The reaction mixture was stirred at room temperature overnight. After the reaction was completed, the reaction mixture was quenched with water (30 mL) in an ice bath and then the product was extracted with CH_2Cl_2 (3 × 20 mL), dried over Na_2SO_4, and concentrated in *vacuo*. The residue was purified by column chromatography on silica gel to give the *N*-methylacetanilide.

4.4 Syntheses of *N*-arylbenzamide

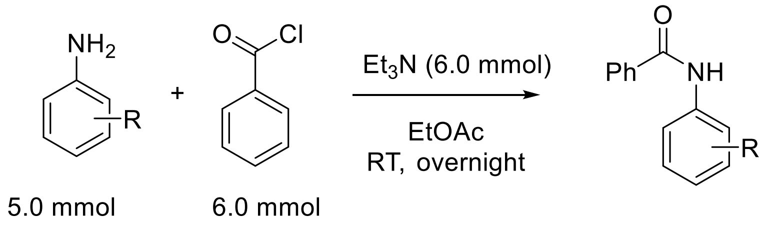

Supplementary Figure 4 Syntheses of *N*-arylbenzamide

The syntheses of *N*-arylbenzamide were implemented following the procedure described in the literature.[3] The aniline (5.0 mmol) was dissolved in ethyl acetate (30 mL) in the presence of Et_3N (900 µL, 1.2 equiv.). A solution of the benzoyl chloride (698 µL, 1.2 equiv.) in ethyl acetate (10 mL) was added dropwise. The reaction mixture was stirred at room temperature overnight. After the reaction, the solvent was removed in *vacuo*. The residue was directly purified by column chromatography on silica gel to give the corresponding *N*-arylbenzamide.

4.5 Esterification of Tamibarotene

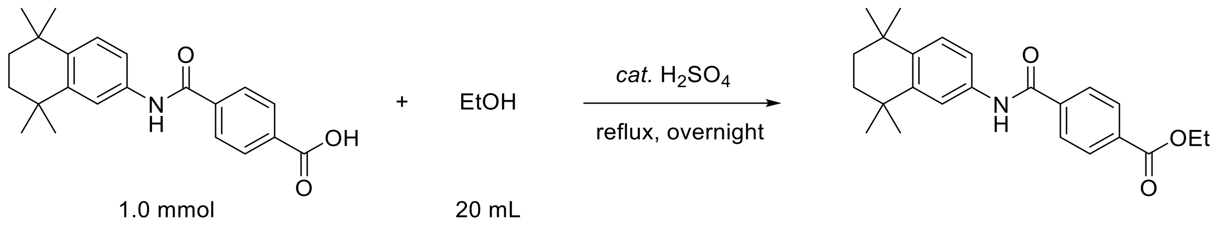

Supplementary Figure 5 Esterification of Tamibarotene

To a solution of Tamibarotene (1.0 mmol) in EtOH (20 mL), a drop of concentrated H_2SO_4 was added and the reaction mixture was refluxed overnight. Then the reaction mixture was concentrated in *vacuo*. The residue was purified by column chromatography on silica gel to give Tamibarotene ethyl ester.

5 Reaction Set-up

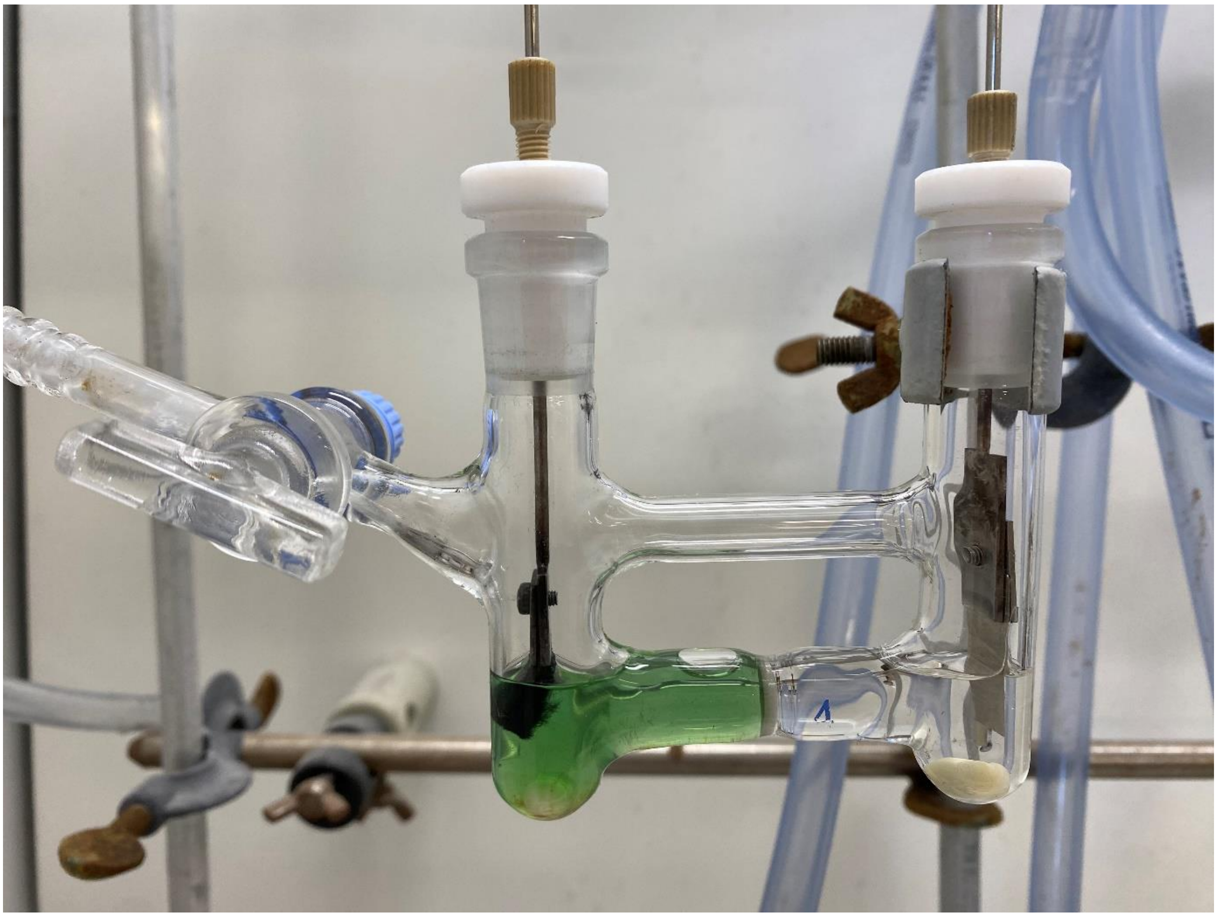

Supplementary Figure 6 Reaction Set-up

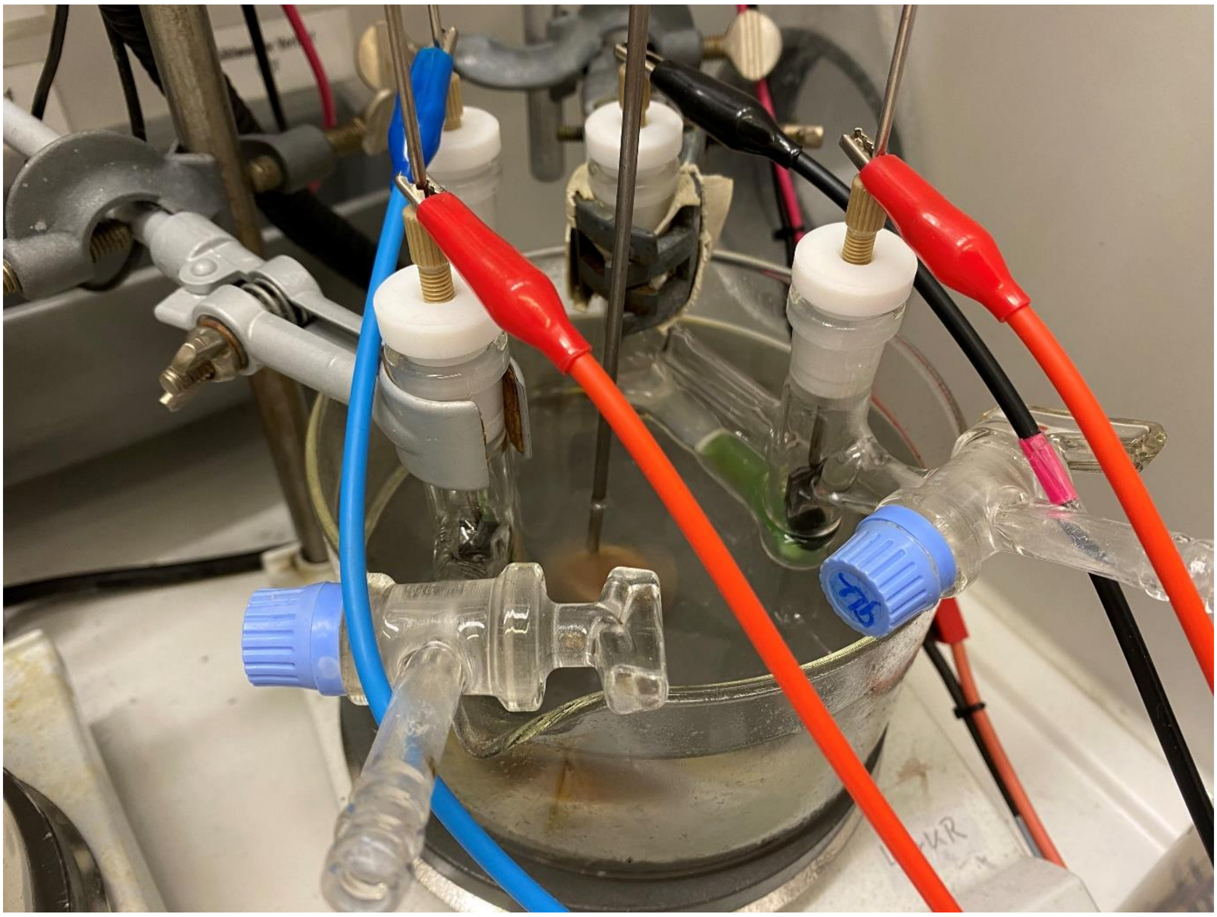

Supplementary Figure 7 Proceeding Reaction Set-up

6 Supplementary Reaction Information

6.1 Effect of Cu Salt and Lutidine

Supplementary Table 1 Effect of $Cu(OTf)_2$ Concentration in Presence of 1.0 mL Xylene

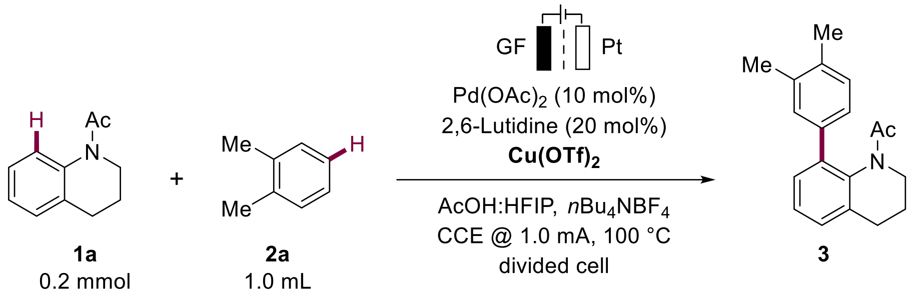

Entry	Amount of $Cu(OTf)_2$	Yield **3** (%)
1	20 mol %	89%
2	10 mol %	88%
3	5.0 mol %	93%
4	0 mol %	40%

Reaction conditions: divided cell, anodic chamber: **1a** (0.20 mmol), **2a** (1.0 mL), $Pd(OAc)_2$ (10 mol %), 2,6-lutidine (20 mol %), $Cu(OTf)_2$, $n Bu_4NBF_4$ (40 mg), HFIP:AcOH (1.0 mL : 2.0 mL), cathodic chamber: **2a** (1.0 mL), $n Bu_4NBF_4$ (40 mg), HFIP:AcOH (1.0 mL : 2.0 mL), 100 °C, electrolysis (CCE) at 1.0 mA, 20 h, graphite felt (GF) anode (10 mm × 15 mm × 2 mm), Pt-plate cathode (10 mm × 15 mm × 0.25 mm), isolated yield.

Although the presence of $Cu(OTf)_2$ is not necessary to achieve catalytic turnover, its use could enable superior turnover numbers and reproducibility due to its ability to avoid generation of Pd black.

Supplementary Table 2 Effect of $Cu(OTf)_2$ Concentration in Presence of 1.0 mmol Xylene

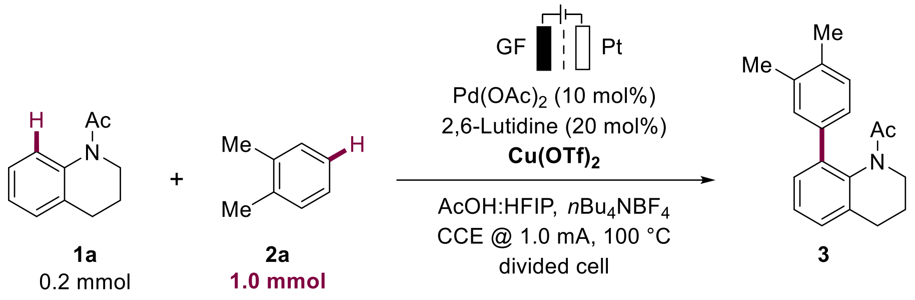

Entry	Amount of $Cu(OTf)_2$	Yield **3** (%)
1	20 mol %	78%
2	10 mol %	72%
3	5.0 mol %	46%[a]
4	0 mol %	44%[a]

Reaction conditions: divided cell, anodic chamber: **1a** (0.20 mmol), **2a** (1.0 mmol), $Pd(OAc)_2$ (10 mol %), 2,6-lutidine (20 mol %), $Cu(OTf)_2$, $n$$Bu_4NBF_4$ (40 mg), HFIP:AcOH (1.3 mL : 2.6 mL), cathodic chamber: **2a** (1.0 mmol), $n$$Bu_4NBF_4$ (40 mg), HFIP:AcOH (1.3 mL : 2.6 mL), 100 °C, electrolysis (CCE) at 1.0 mA, 20 h, graphite felt (GF) anode (10 mm × 15 mm × 2 mm), Pt-plate cathode (10 mm × 15 mm × 0.25 mm), isolated yield. a. Yields are determined by crude H-NMR analyses with CH_2Br_2 as internal standard.

6.2 Role of Electricity

Supplementary Table 3 Reactions without Electricity

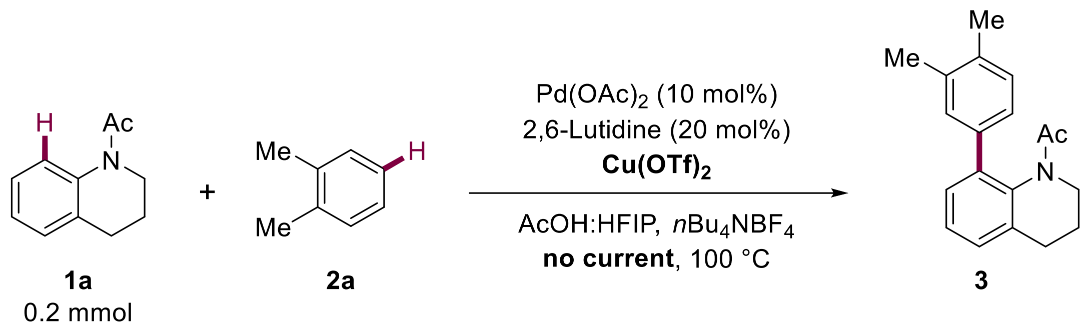

Entry	**2a** (mmol)	$Cu(OTf)_2$ (mol %)	SM (%)	Yield **3** (%)
1[a]	1.0	5.0	--	< 5%
2	1.0	10	--	< 5%
3	1.0	20	83%	8%
4	8.3 (1 mL)	20	--	22%

Reaction conditions: **1a** (0.20 mmol), **2a** (1.0–8.3 mmol), $Pd(OAc)_2$ (10 mol %), 2,6-lutidine (20 mol %), $Cu(OTf)_2$, *n*Bu_4NBF_4 (40 mg), HFIP:AcOH (1.3 mL : 2.6 mL), 100 °C, 20 h, graphite felt (GF) anode (10 mm × 15 mm × 6 mm), Pt-plate cathode (10 mm × 15 mm × 0.25 mm), yields were determined by crude NMR measurement with CH_2Br_2 as internal standard. a. 80 °C.

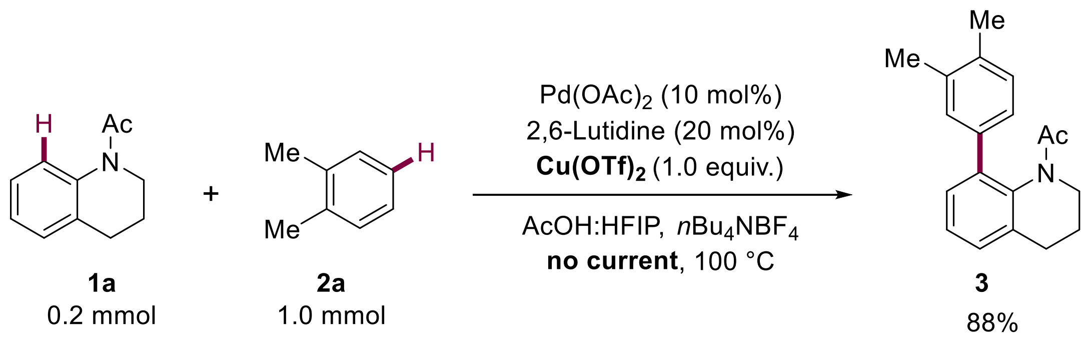

Supplementary Figure 8 Reaction Using Stoichiometric $Cu(OTf)_2$

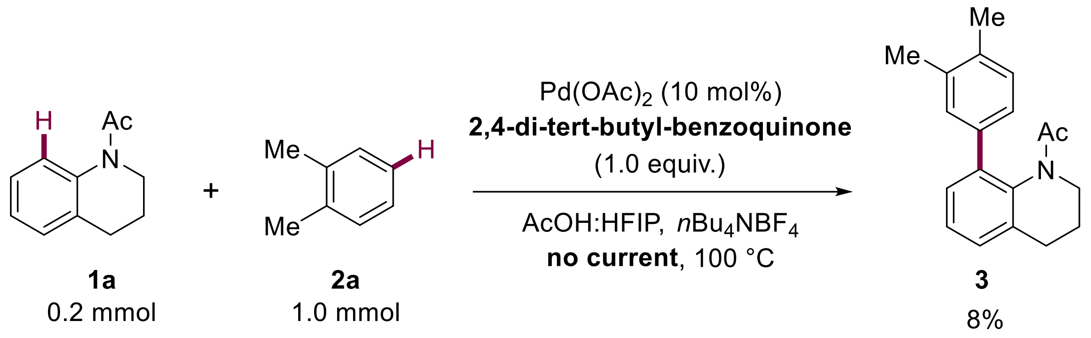

Supplementary Figure 9 Reaction Using Stoichiometric Benzoquinone

6.3 Role of Divided Cell

Supplementary Table 4 Chemoselectivity-Dependence on Reactor

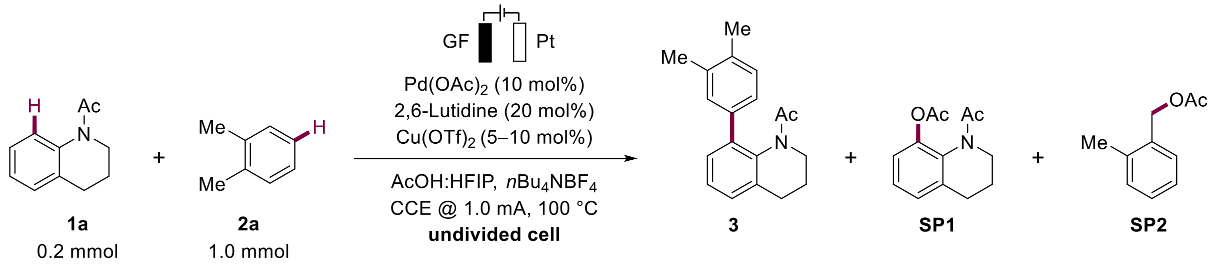

Entry	Amount of $Cu(OTf)_2$	SM (%)	TON of **3**	TON of **SP1**	TON of **SP2**
1	10 mol %	29%	3.4	1.4	1.7
2[a]	5 mol %	45%	2.2	3.0	4.1

Reaction conditions: undivided cell, **1a** (0.20 mmol), **2a** (1.0 mL), $Pd(OAc)_2$ (10 mol %), 2,6-lutidine (20 mol %), $Cu(OTf)_2$, $n Bu_4NBF_4$ (40 mg), HFIP:AcOH (1.3 mL : 2.6 mL), 100 °C, electrolysis (CCE) at 1.0 mA, 18 h, graphite felt (GF) anode (10 mm × 15 mm × 2 mm), Pt-plate cathode (10 mm × 15 mm × 0.25 mm). Starting material and product **3** were quantified via crude NMR measurement with CH_2Br_2 as internal standard. Turnover numbers (TON) of **SP1** and **SP2** were determined by GC using dodecane as internal standard. a. 80 °C.

Low yield and chemoselectivity were observed when using an undivided cell to carry out the electrolysis.

6.4 Quality of Divided Cell

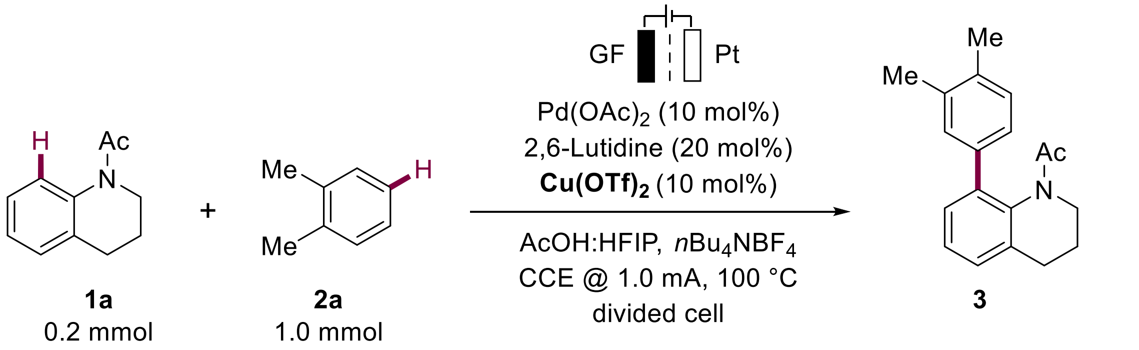

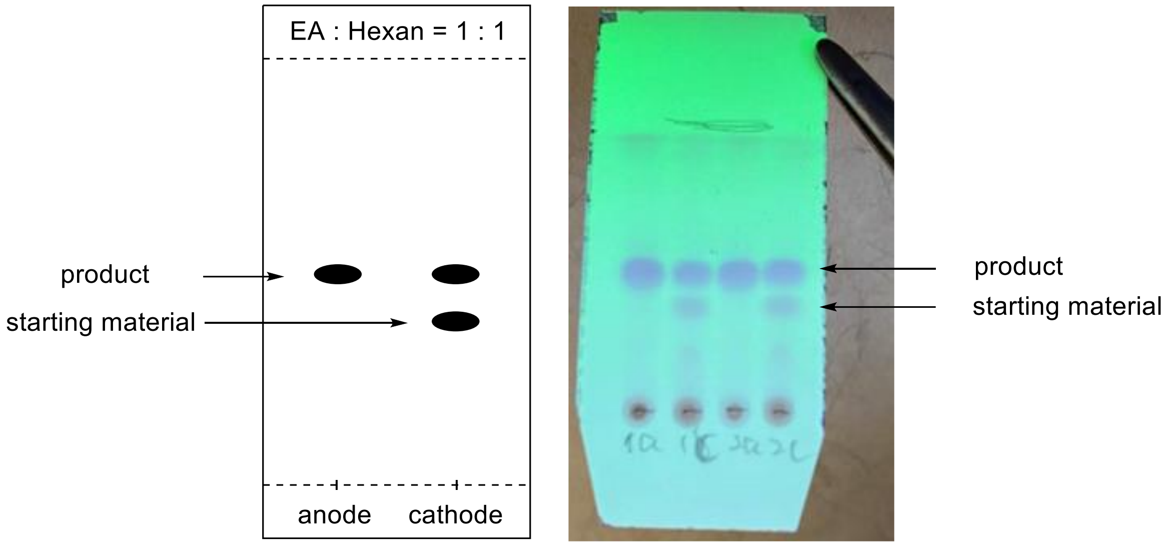

Supplementary Figure 10 Starting Material and Product Distribution on Both Chamber

Reaction conditions: divided cell, anodic chamber: **1a** (0.20 mmol), **2a** (1.0 mmol), $Pd(OAc)_2$ (10 mol %), 2,6-lutidine (20 mol %), $Cu(OTf)_2$ (10 mol %), $n$$Bu_4NBF_4$ (40 mg), HFIP:AcOH (1.3 mL : 2.6 mL), cathodic chamber: **2a** (1.0 mmol), $n$$Bu_4NBF_4$ (40 mg), HFIP:AcOH (1.3 mL : 2.6 mL), 100 °C, electrolysis (CCE) at 1.0 mA, 20 h, graphite felt (GF) anode (10 mm × 15 mm × 2 mm), Pt-plate cathode (10 mm × 15 mm × 0.25 mm), isolated yield.

Approximately, an average of 10% of starting material could be isolated from the cathodic chamber, depending on the quality of the membranes.

6.5 Role of Temperature

Supplementary Table 5 Effect of Temperature

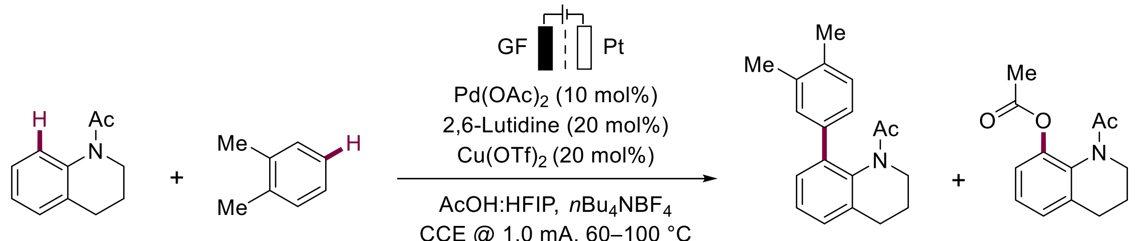

1a 0.2 mmol **2a** 1.0 mL **3** **SP1**

Entry	Temperature	SM (%)	Yield **3** (%)	Yield **SP1** (%)
1	100 °C		89%	n.d.
2	80 °C		81%	n.d.
3	60 °C	40%	20%	21%

Reaction conditions: divided cell, anodic chamber: **1a** (0.20 mmol), **2a** (1.0 mL), $Pd(OAc)_2$ (10 mol %), 2,6-lutidine (20 mol %), $Cu(OTf)_2$ (20 mol %), $n$$Bu_4NBF_4$ (40 mg), HFIP:AcOH (1.0 mL : 2.0 mL), cathodic chamber: **2a** (1.0 mL), $n$$Bu_4NBF_4$ (40 mg), HFIP:AcOH (1.0 mL : 2.0 mL), 60–100 °C, electrolysis (CCE) at 1.0 mA, 20 h, graphite felt (GF) anode (10 mm × 15 mm × 2 mm), Pt-plate cathode (10 mm × 15 mm × 0.25 mm), isolated yield.

6.6 Role of Xylene Stoichiometry

Supplementary Table 6 Yields Depending on the Amount of Xylene

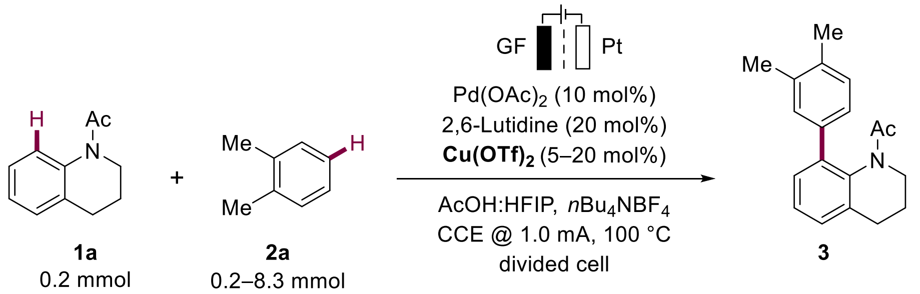

Entry	Amount of $Cu(OTf)_2$	**2a** (mmol)	Yield **3** (%)
1	20 mol %	8.3 (excess)	89%
2	20 mol %	1.0 (5.0 eq.)	78%
3	20 mol %	0.2 (2.0 eq.)	31%[a]
4	10 mol %	8.3 (excess)	88%
5	10 mol %	1.0 (5.0 eq.)	72%
6	10 mol %	0.6 (3.0 eq.)	32%[a]
7	5.0 mol %	1.0 (5.0 eq.)	46%[a]
8	5.0 mol %	0.6 (3.0 eq.)	28%[a]
9	5.0 mol %	0.2 (2.0 eq.)	12%[a]

Reaction conditions: divided cell, anodic chamber: **1a** (0.20 mmol), **2a** (0.2–8.3 mmol), $Pd(OAc)_2$ (10 mol %), 2,6-lutidine (20 mol %), $Cu(OTf)_2$ (5–20 mol %), $n$$Bu_4NBF_4$ (40 mg), HFIP:AcOH (1.0 mL : 2.0 mL), cathodic chamber: **2a** (0.2–8.3 mmol), $n$$Bu_4NBF_4$ (40 mg), HFIP:AcOH (1.0 mL : 2.0 mL), 100 °C, electrolysis (CCE) at 1.0 mA, 20 h, graphite felt (GF) anode (10 mm × 15 mm × 2 mm), Pt-plate cathode (10 mm × 15 mm × 0.25 mm), isolated yield. a. Yields are determined by crude H-NMR analyses with CH_2Br_2 as internal standard.

6.7 Role of HFIP

Supplementary Table 7 Effect of Solvent with Low Concentration of *n*Bu_4NBF_4 Using Electricity

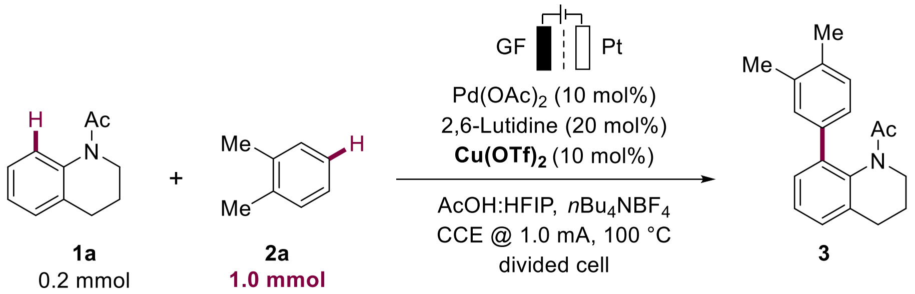

Entry	AcOH (mL)	HFIP (mL)	Yield **3** (%)
1	2.0	1.0	72%*isolated*
2	2.5	0.5	56%
3	3.0	0.3	52%
4	3.0	0	14%

Reaction conditions: divided cell, anodic chamber: **1a** (0.20 mmol), **2a** (1.0 mmol), $Pd(OAc)_2$ (10 mol %), 2,6-lutidine (20 mol %), $Cu(OTf)_2$ (10 mol %), *n*Bu_4NBF_4 (40 mg), HFIP:AcOH, cathodic chamber: **2a** (1.0 mmol), *n*Bu_4NBF_4 (40 mg), HFIP:AcOH, 100 °C, electrolysis (CCE) at 1.0 mA, 20 h, graphite felt (GF) anode (10 mm × 15 mm × 2 mm), Pt-plate cathode (10 mm × 15 mm × 0.25 mm). Yields are determined by crude H-NMR analyses with CH_2Br_2 as internal standard.

Supplementary Table 8 Effect of Solvent with High Concentration of *n*Bu_4NBF_4 Using Electricity

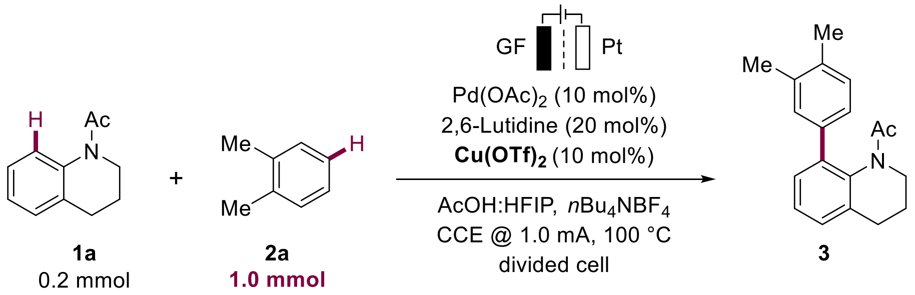

Entry	AcOH (mL)	HFIP (mL)	Yield **3** (%)
1	2.0	1.0	78%
2	2.5	0.5	45%
3	3.0	0.3	52%
4	3.0	0	36%

Reaction conditions: divided cell, anodic chamber: **1a** (0.20 mmol), **2a** (1.0 mmol), $Pd(OAc)_2$ (10 mol %), 2,6-lutidine (20 mol %), $Cu(OTf)_2$ (10 mol %), *n*Bu_4NBF_4 (200 mg), HFIP:AcOH,

cathodic chamber: **2a** (1.0 mmol), *n*Bu_4NBF_4 (200 mg), HFIP:AcOH, 100 °C, electrolysis (CCE) at 1.0 mA, 20 h, graphite felt (GF) anode (10 mm × 15 mm × 2 mm), Pt-plate cathode (10 mm × 15 mm × 0.25 mm). Yields are determined by crude H-NMR analyses with CH_2Br_2 as internal standard.

Supplementary Table 9 Effect of Solvent with Low Concentration of *n*Bu_4NBF_4 Using Stoichiometric $Cu(OTf)_2$

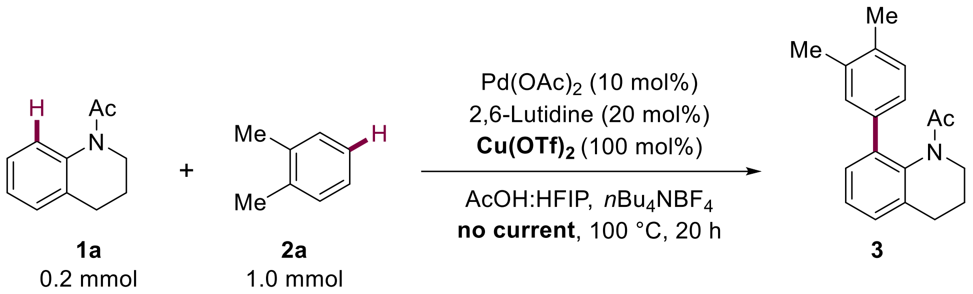

Entry	AcOH (mL)	HFIP (mL)	Yield **3** (%)
1	2.0	1.0	88%
2	2.5	0.5	60%
3	3.0	0.3	48%
4	3.0	0	40%

Reaction conditions: **1a** (0.20 mmol), **2a** (1.0 mmol), $Pd(OAc)_2$ (10 mol %), 2,6-lutidine (20 mol %), $Cu(OTf)_2$ (10 mol %), *n*Bu_4NBF_4 (40 mg), HFIP:AcOH, 100 °C, 20 h, yields were determined by crude NMR measurement with CH_2Br_2 as internal standard.

6.8 Role of Ligand

Supplementary Table 10 Effect of Ligand on Regioselectivity

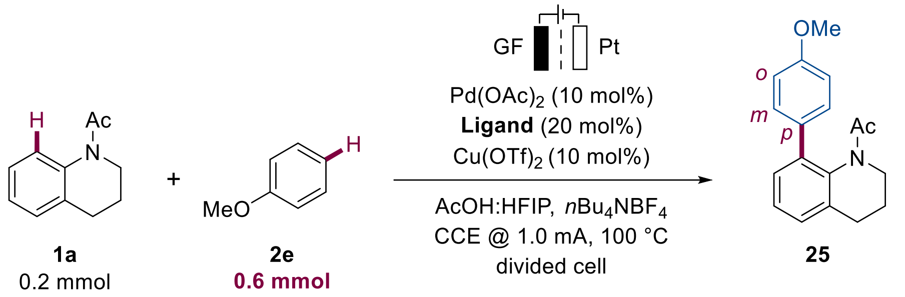

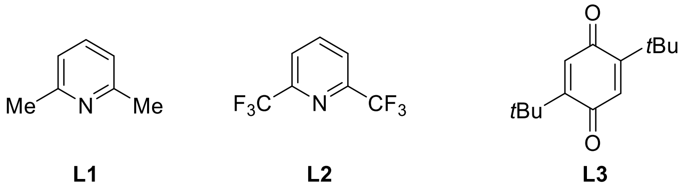

Entry	Ligand	Yield **25** (%)	*o* : *m* : *p*	*p* : *others*
1	**L1**	61%	1.0 : 3.8 : 24.0	5.0 : 1
2	**L2**	55%	1.0 : 2.7 : 18.4	5.0 : 1
3	**L3**	62%[a]	1.0 : 2.2 : 12.7	4.0 : 1

Reaction conditions: divided cell, anodic chamber: **1a** (0.20 mmol), **2e** (0.60 mmol), $Pd(OAc)_2$ (10 mol %), Ligand (20 mol %), $Cu(OTf)_2$ (10 mol %), $n$$Bu_4NBF_4$ (40 mg), HFIP:AcOH (1.3 mL : 2.6 mL), cathodic chamber: **2e** (0.60 mmol), $n$$Bu_4NBF_4$ (40 mg), HFIP:AcOH (1.3 mL : 2.6 mL), 100 °C, electrolysis (CCE) at 1.0 mA, 20 h, graphite felt (GF) anode (10 mm × 15 mm × 2 mm), Pt-plate cathode (10 mm × 15 mm × 0.25 mm), isolated yield. a. no $Cu(OTf)_2$ was used.

6.9 Reaction Cell Voltage

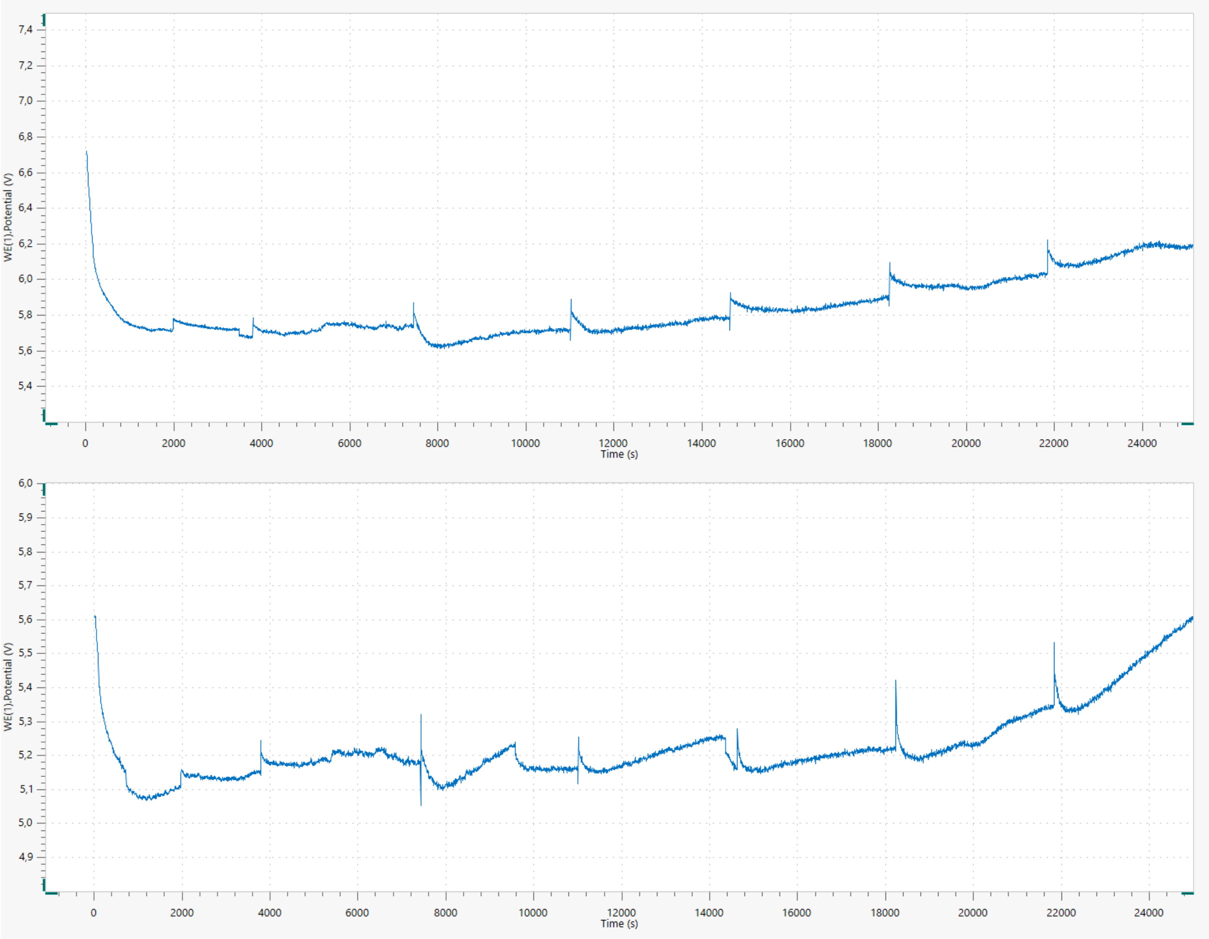

Supplementary Figure 11 Reaction Cell Voltage Using 200 mg *n*Bu_4NBF_4

6.10 Reaction Using IKA Potentiostat

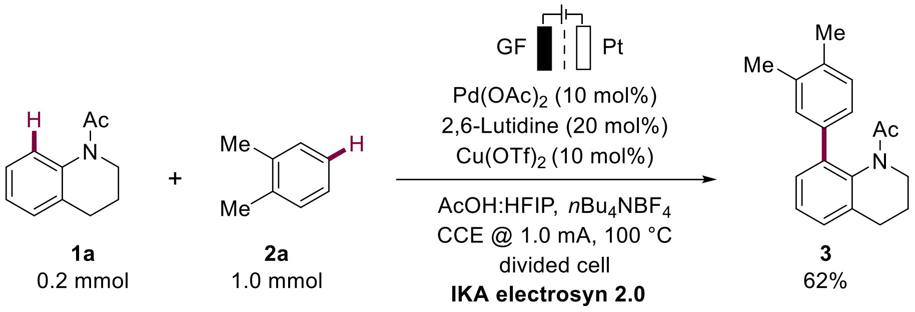

Reaction conditions: divided cell, anodic chamber: **1a** (0.20 mmol), **2a** (1.0 mmol), $Pd(OAc)_2$ (10 mol %), 2,6-lutidine (20 mol %), $Cu(OTf)_2$ (10 mol %), $n Bu_4NBF_4$ (200 mg), HFIP:AcOH (1.3 mL : 2.6 mL), cathodic chamber: **2a** (1.0 mmol), $n Bu_4NBF_4$ (200 mg), HFIP:AcOH (1.3 mL : 2.6 mL), 100 °C, electrolysis (CCE) at 1.0 mA for 20 h, graphite felt (GF) anode (10 mm × 15 mm × 2 mm), Pt-plate cathode (10 mm × 15 mm × 0.25 mm), isolated yield.

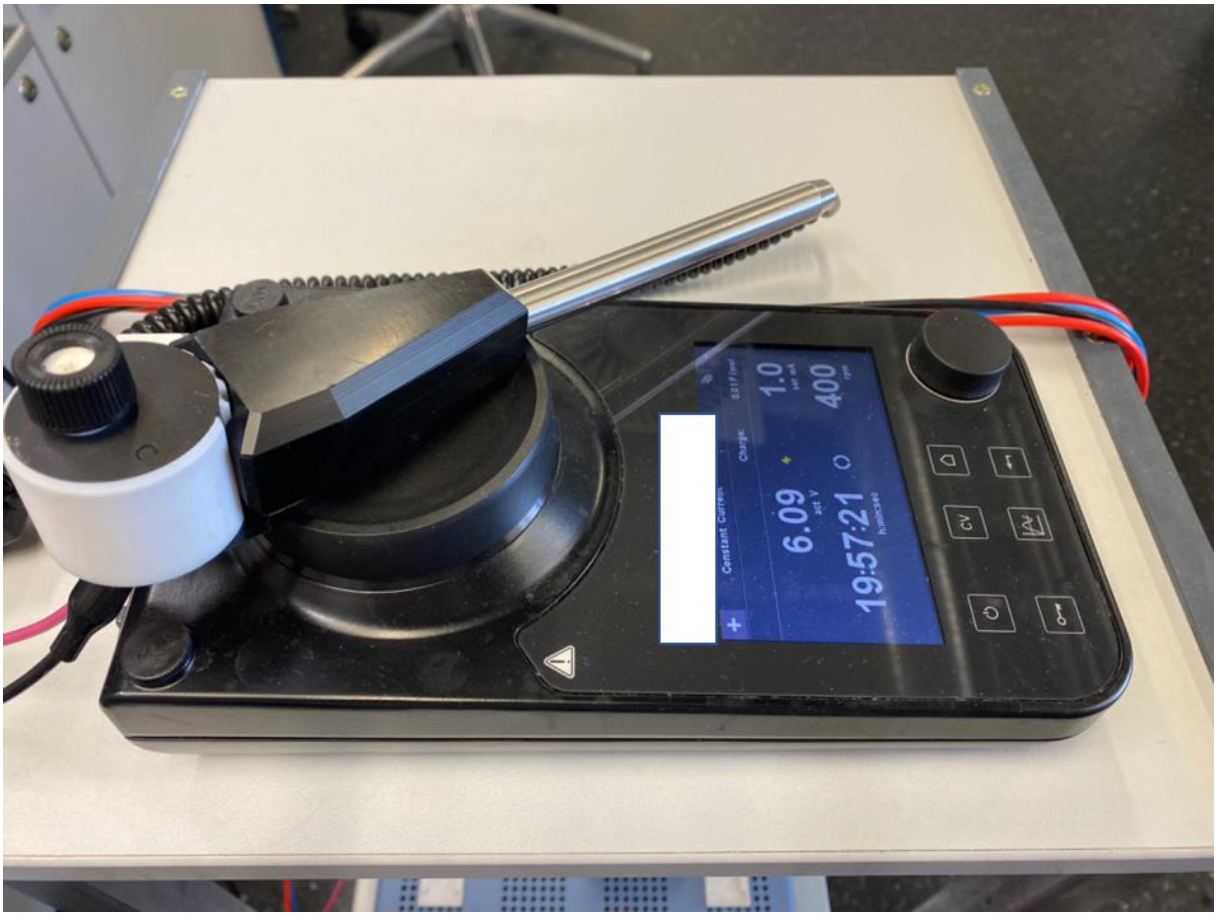

Supplementary Figure 12 Reaction Run with IKA Electrasyn 2.0. The potentiostat was connected to the divided cell reactor through two cables.

7 Supplementary Mechanistic Studies

7.1 VTNA

7.1.1 VTNA Studies on [Pd]

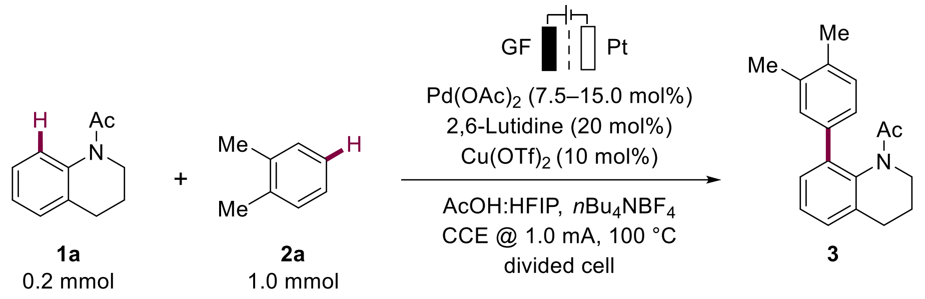

Reaction conditions: divided cell, anodic chamber: **1a** (0.20 mmol), **2a** (1.0 mmol), $Pd(OAc)_2$ (7.5–15.0 mol %), 2,6-lutidine (20 mol %), $Cu(OTf)_2$ (10 mol %), $n Bu_4NBF_4$ (200 mg), HFIP:AcOH (5.4 mL, 1 : 2), cathodic chamber: **2a** (1.0 mmol), $n Bu_4NBF_4$ (200 mg), HFIP:AcOH (5.4 mL, 1 : 2), 100 °C, electrolysis (CCE) at 1.0 mA, graphite felt (GF) anode (10 mm × 15 mm × 2 mm), Pt-plate cathode (10 mm × 15 mm × 0.25 mm), GC yield. At specific intervals shown on the horizontal axis, a small aliquot of the reaction mixture (0.05 mL) was taken, filtered through a small silica gel column, added with 0.05 mL internal standard stock solution (0.2 mmol dodecane in 5.5 mL EtOAc), and analyzed by GC. To obtain a reliable result, data was generated by using the same reactor, potentiostat, heating plate, and GC.

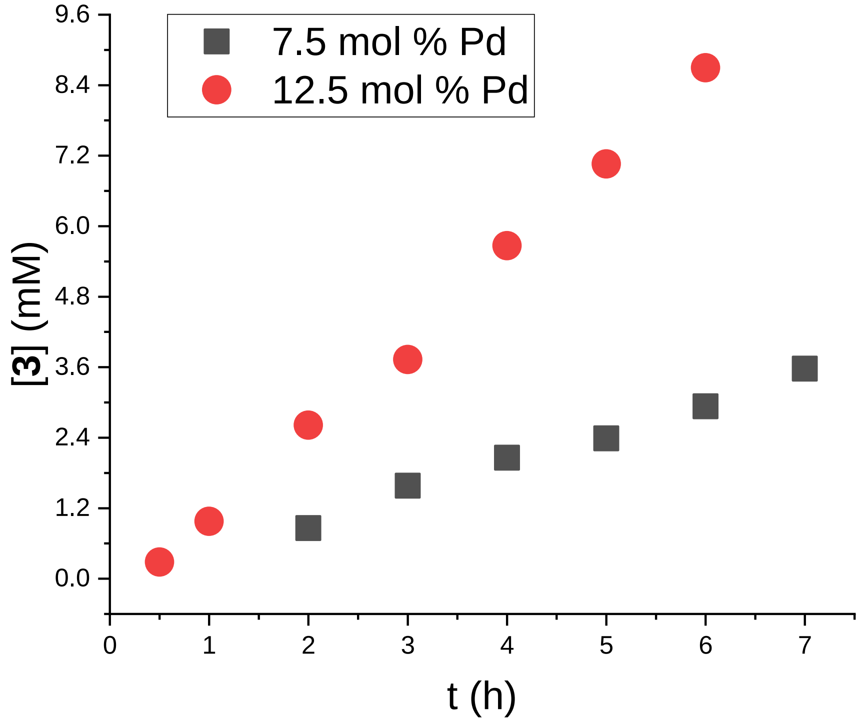

Supplementary Figure 13 Reaction Profile

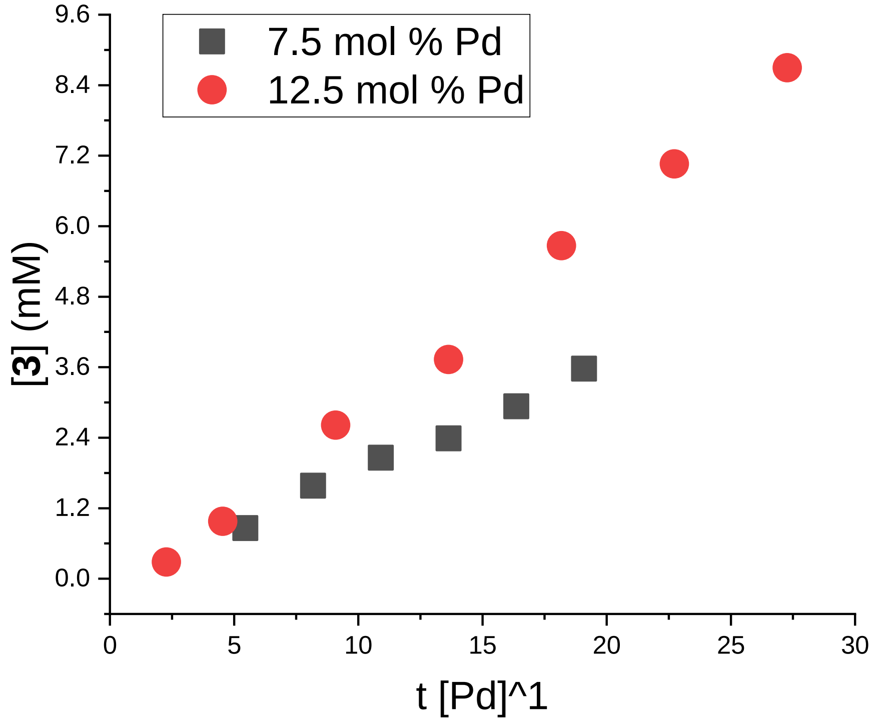

Supplementary Figure 14 First Order Correlation

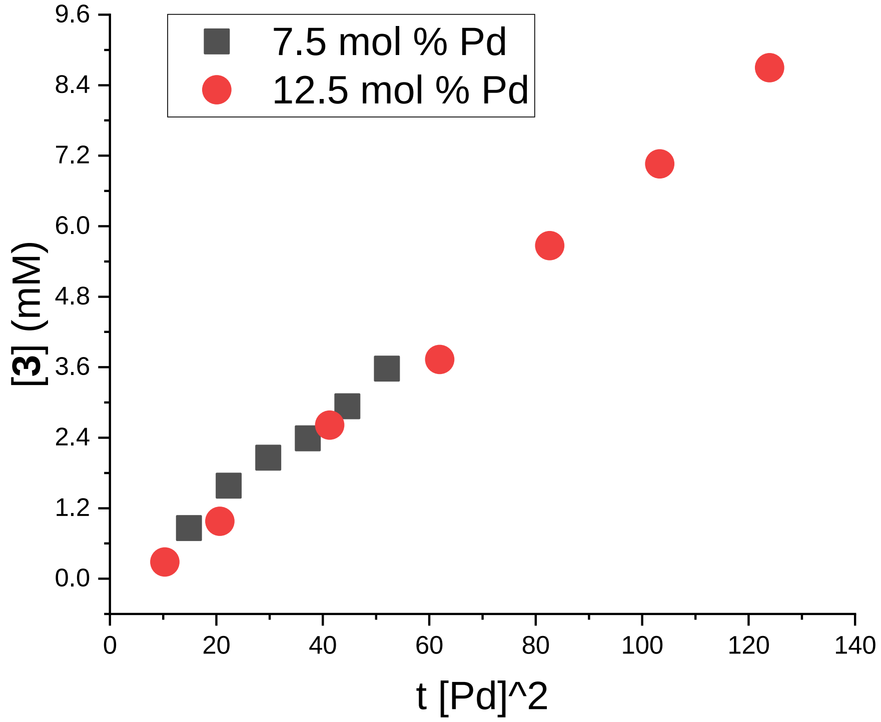

Supplementary Figure 15 Second Order Correlation

7.1.2 VTNA Studies on [**1a**]

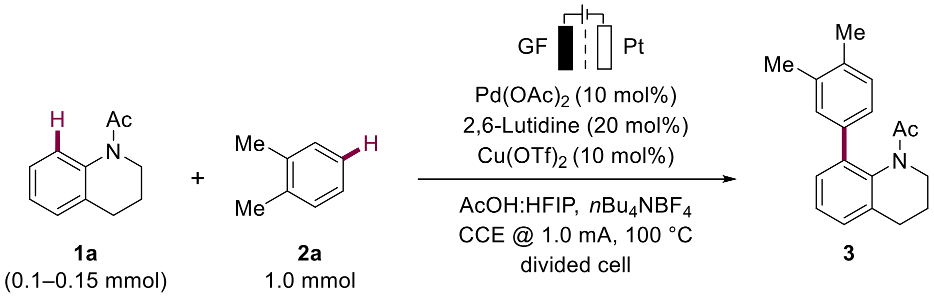

Reaction conditions: divided cell, anodic chamber: **1a** (0.1–0.15 mmol), **2a** (1.0 mmol), $Pd(OAc)_2$ (10 mol %), 2,6-lutidine (20 mol %), $Cu(OTf)_2$ (10 mol %), $n$$Bu_4NBF_4$ (200 mg), HFIP:AcOH (5.4 mL, 1 : 2), cathodic chamber: **2a** (1.0 mmol), $n$$Bu_4NBF_4$ (200 mg), HFIP:AcOH (5.4 mL, 1 : 2), 100 °C, electrolysis (CCE) at 1.0 mA, graphite felt (GF) anode (10 mm × 15 mm × 2 mm), Pt-plate cathode (10 mm × 15 mm × 0.25 mm), GC yield. At specific intervals shown on the horizontal axis, a small aliquot of the reaction mixture (0.05 mL) was taken, filtered through a small silica gel column, added with 0.05 mL internal standard stock solution (0.2 mmol dodecane in 5.5 mL EtOAc), and analyzed by GC. To obtain a reliable result, data was generated by using the same reactor, potentiostat, heating plate, and GC.

7.1.3 VTNA Studies on [2a]

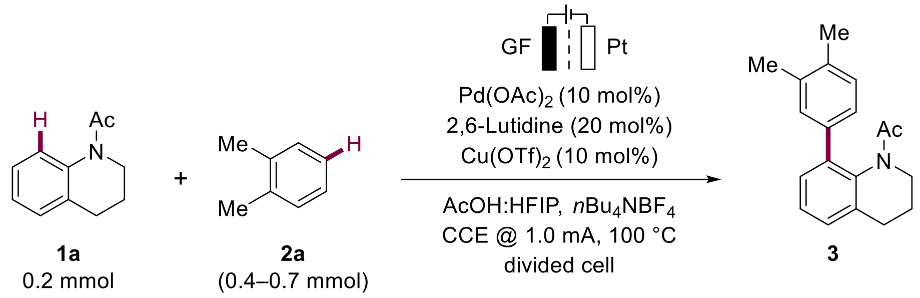

Reaction conditions: divided cell, anodic chamber: **1a** (0.20 mmol), **2a** (0.4–0.7 mmol), $Pd(OAc)_2$, 2,6-lutidine (20 mol %), $Cu(OTf)_2$ (10 mol %), *n*Bu_4NBF_4 (200 mg), HFIP:AcOH (5.4 mL, 1 : 2), cathodic chamber: **2a** (0.4–0.7 mmol), *n*Bu_4NBF_4 (200 mg), HFIP:AcOH (5.4 mL, 1 : 2), 100 °C, electrolysis (CCE) at 1.0 mA, graphite felt (GF) anode (10 mm × 15 mm × 2 mm), Pt-plate cathode (10 mm × 15 mm × 0.25 mm), GC yield. At specific intervals shown on the horizontal axis, a small aliquot of the reaction mixture (0.05 mL) was taken, filtered through a small silica gel column, added with 0.05 mL internal standard stock solution (0.2 mmol dodecane in 5.5 mL EtOAc), and analyzed by GC. To obtain a reliable result, data was generated by using the same reactor, potentiostat, heating plate, and GC.

7.2 Initial Rate Analysis

7.2.1 Kinetic Dependence of Initial Rate on [Pd]

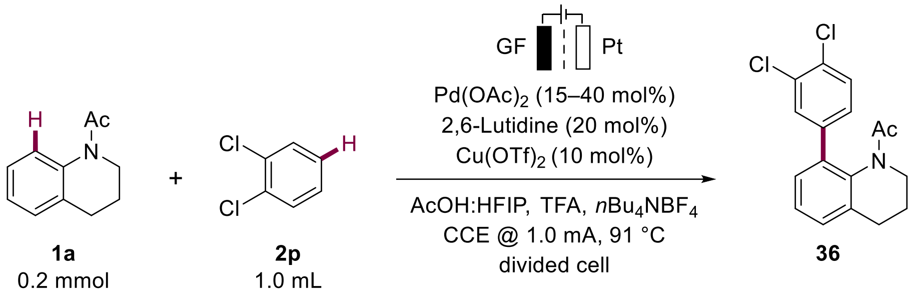

Reaction conditions: divided cell, anodic chamber: **1a** (0.20 mmol), **2p** (1.0 mL), $Pd(OAc)_2$ (15–40 mol %), 2,6-lutidine (20 mol %), $Cu(OTf)_2$ (10 mol %), *n*Bu_4NBF_4 (200 mg), HFIP:AcOH (4.0 mL, 1 : 2), TFA (0.5 mL), cathodic chamber: **2p** (1.0 mL), *n*Bu_4NBF_4 (200 mg), HFIP:AcOH (4.0 mL, 1 : 2), TFA (0.5 mL), 91 °C, electrolysis (CCE) at 1.0 mA, graphite felt (GF) anode (10 mm × 15 mm × 2 mm), Pt-plate cathode (10 mm × 15 mm × 0.25 mm), GC yield. At specific intervals shown on the horizontal axis, a small aliquot of the reaction mixture (0.1 mL) was taken, filtered through a small silica gel column, added with 0.1 mL internal standard stock solution (0.2 mmol dodecane in 5.5 mL EtOAc), and analyzed by GC. Only the β-isomer was quantified for the kinetics studies. To obtain a reliable result, data was generated by using the same reactor, potentiostat, heating plate, and GC.

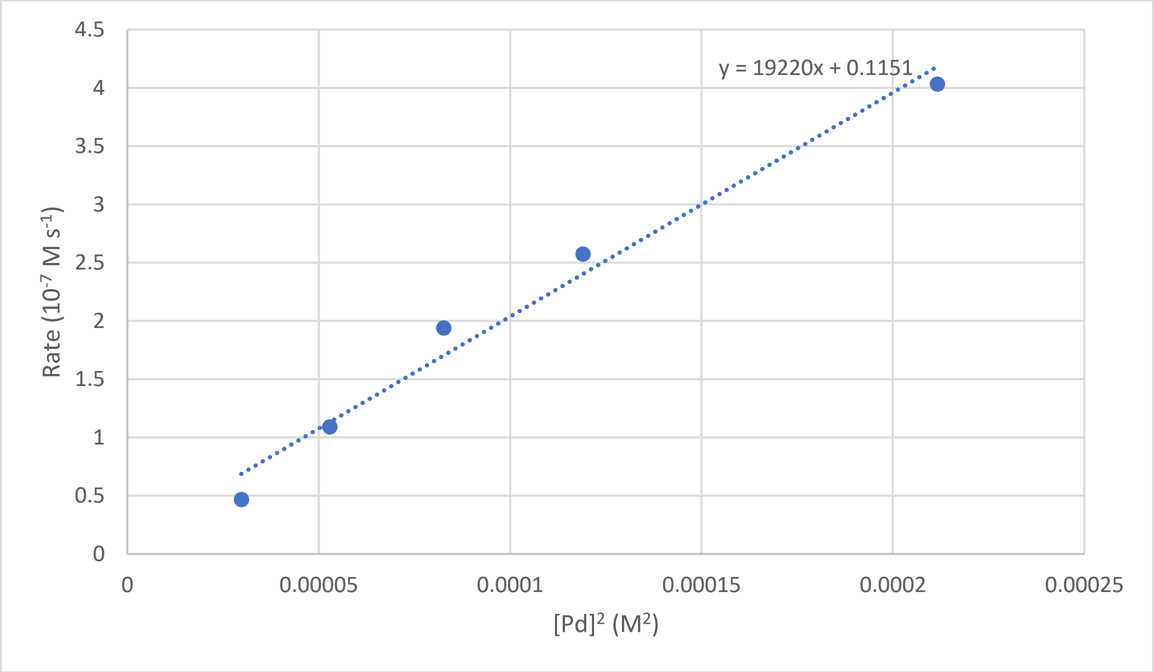

Supplementary Figure 16 Second Order Correlation on [Pd]

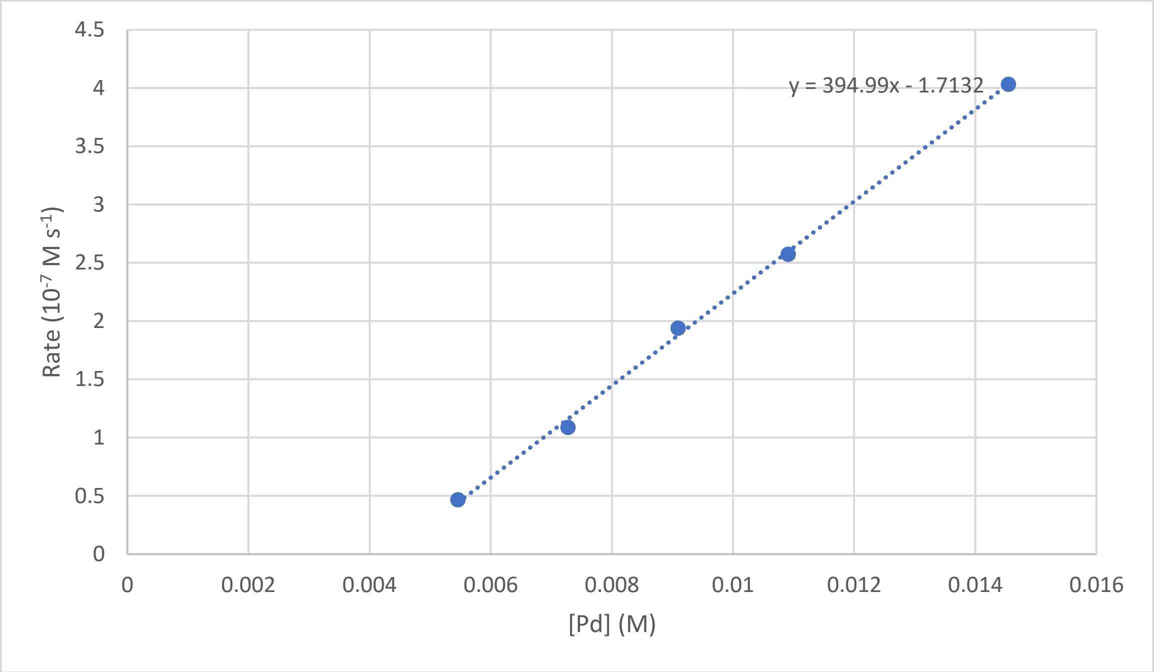

Supplementary Figure 17 First Order Correlation on [Pd]

Although both first-order and second-order correlations gave linear plot, we could easily exclude first-order on [Pd] due to the negatively large intercept on the plot.

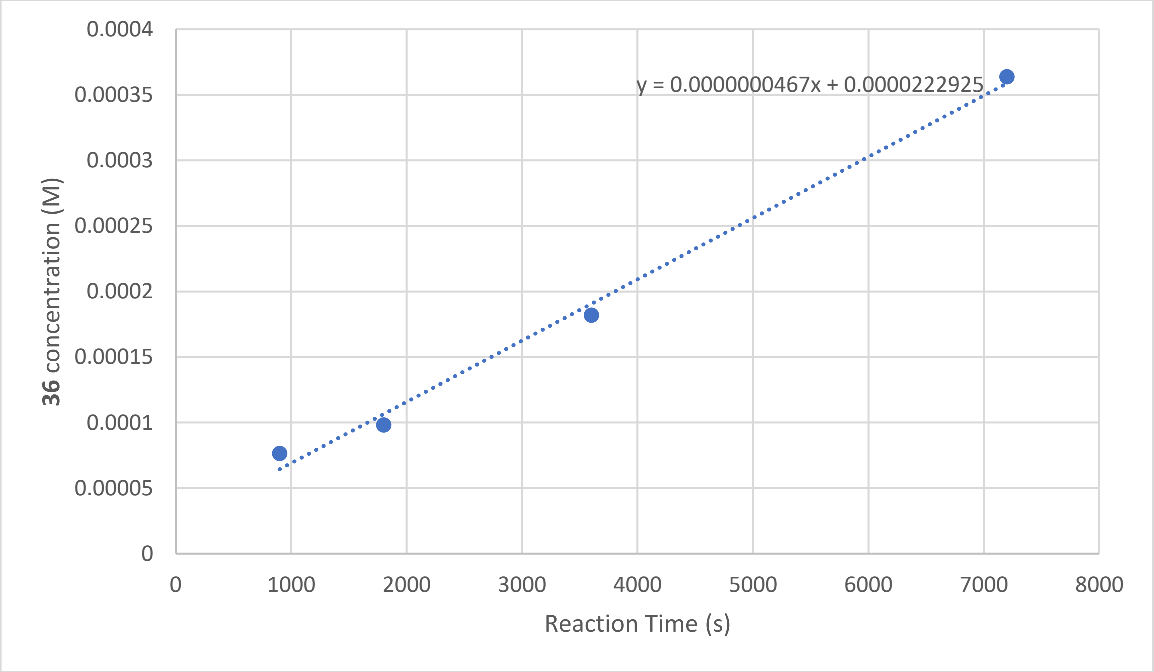

Supplementary Figure 18 Reaction Rate Using 15 mol % Pd

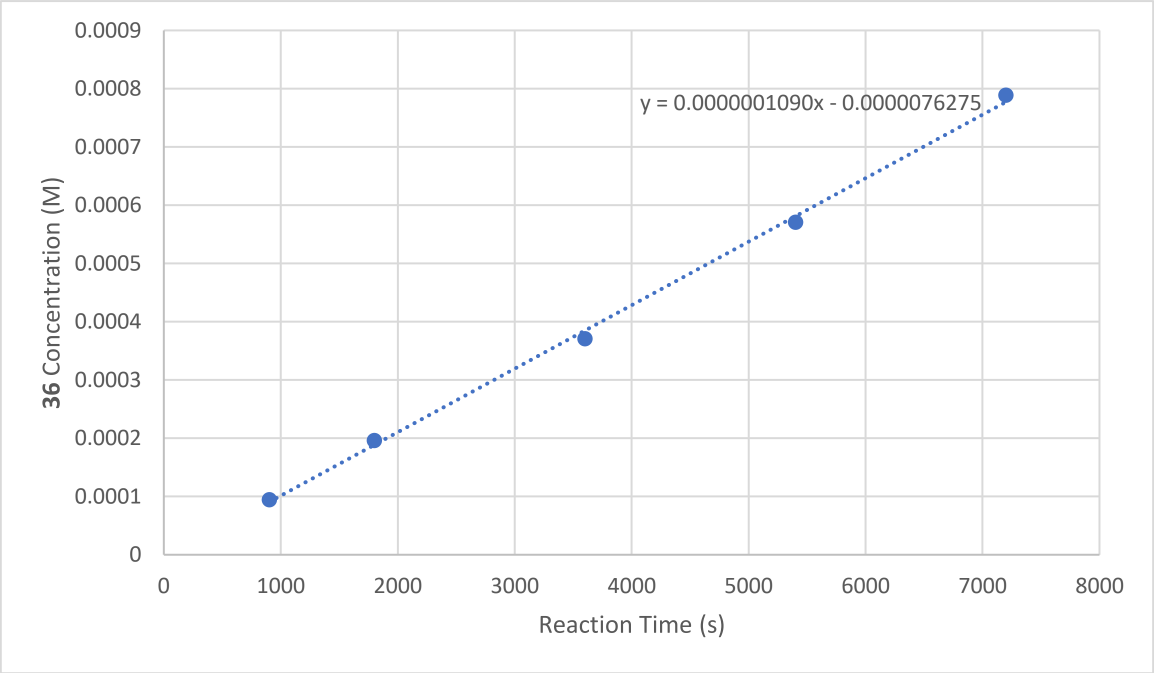

Supplementary Figure 19 Reaction Rate Using 20 mol % Pd

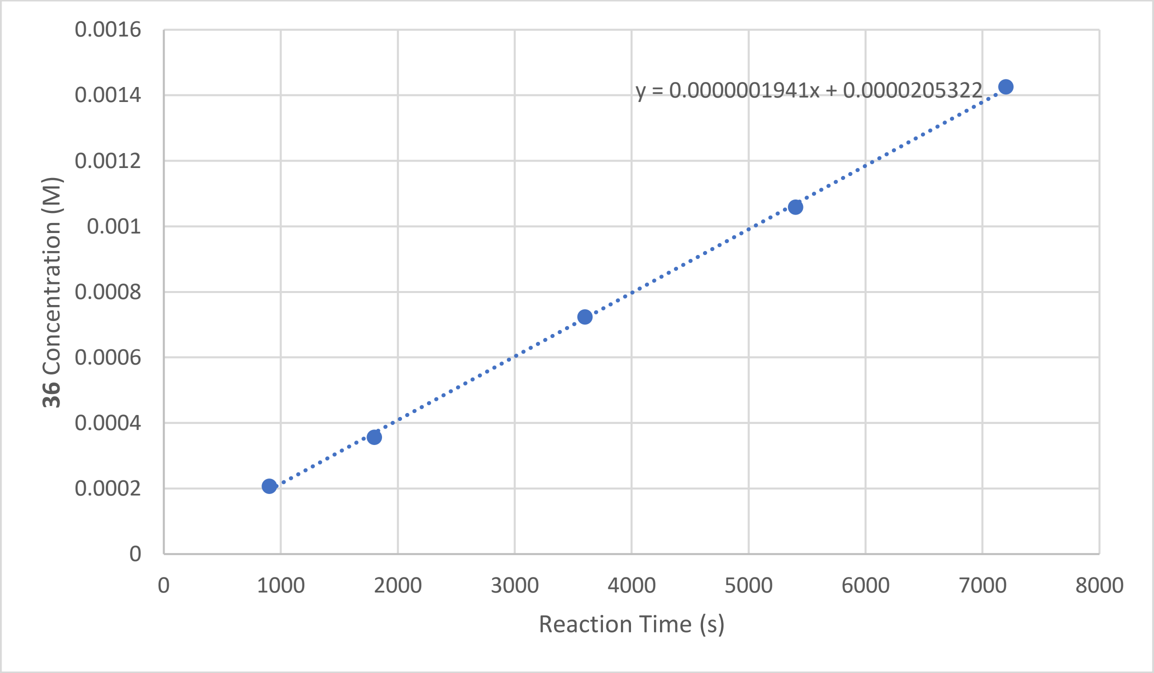

Supplementary Figure 20 Reaction Rate Using 25 mol % Pd

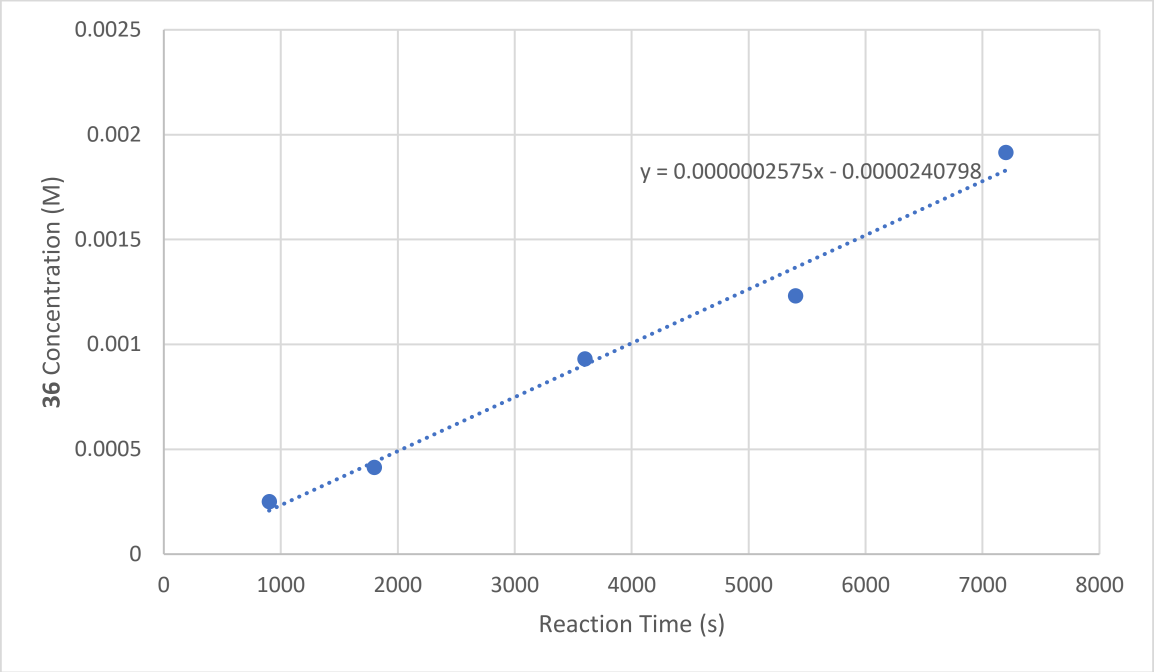

Supplementary Figure 21 Reaction Rate Using 30 mol % Pd

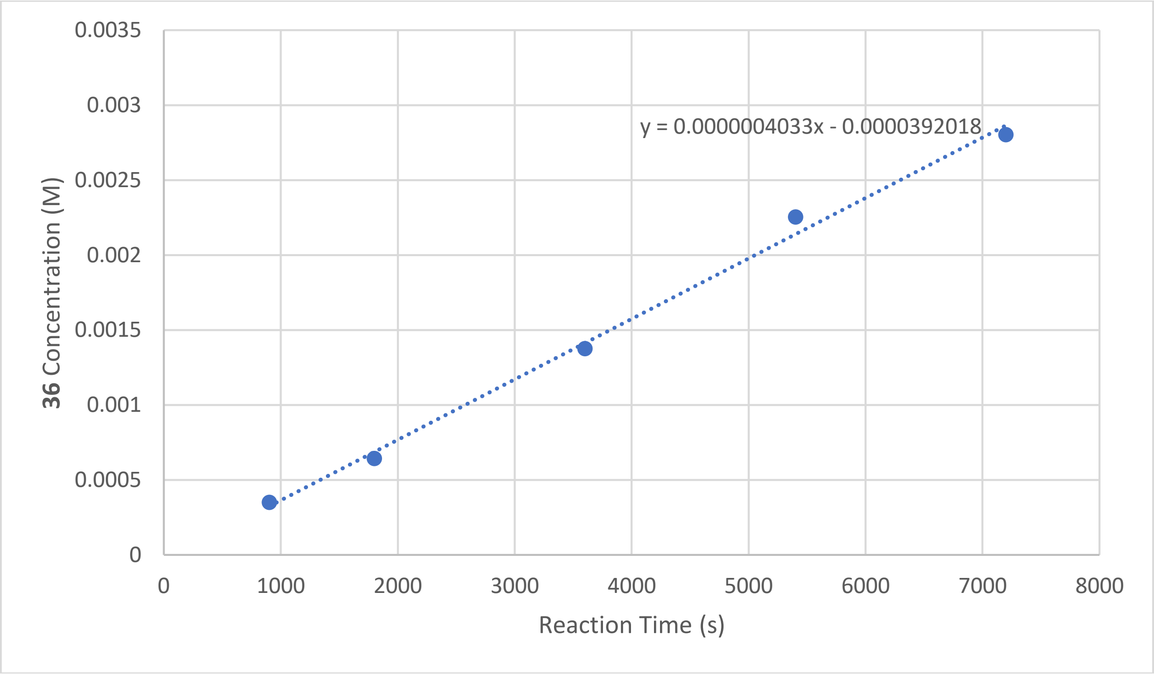

Supplementary Figure 22 Reaction Rate Using 40 mol % Pd

7.2.2 Kinetic Dependence of Initial Rate on Electricity

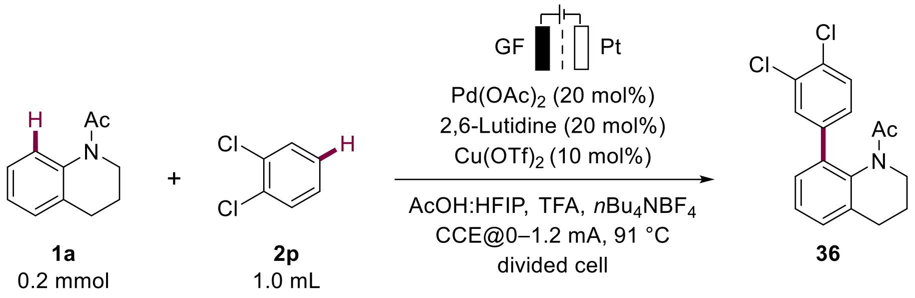

Reaction conditions: divided cell, anodic chamber: **1a** (0.20 mmol), **2p** (1.0 mL), $Pd(OAc)_2$ (20 mol %), 2,6-lutidine (20 mol %), $Cu(OTf)_2$ (10 mol %), $n Bu_4NBF_4$ (200 mg), HFIP:AcOH (4.0 mL, 1 : 2), TFA (0.5 mL), cathodic chamber: **2p** (1.0 mL), $n Bu_4NBF_4$ (200 mg), HFIP:AcOH (4.0 mL, 1 : 2), TFA (0.5 mL), 91 °C, electrolysis (CCE) at 0–1.2 mA, graphite felt (GF) anode (10 mm × 15 mm × 2 mm), Pt-plate cathode (10 mm × 15 mm × 0.25 mm), GC yield. At specific intervals shown on the horizontal axis, a small aliquot of the reaction mixture (0.1 mL) was taken, filtered through a small silica gel column, added with 0.1 mL internal standard stock solution (0.2 mmol dodecane in 5.5 mL EtOAc), and analyzed by GC. Only the β-isomer was quantified for the kinetics studies. To obtain a reliable result, data was generated by using the same reactor, potentiostat, heating plate, and GC.

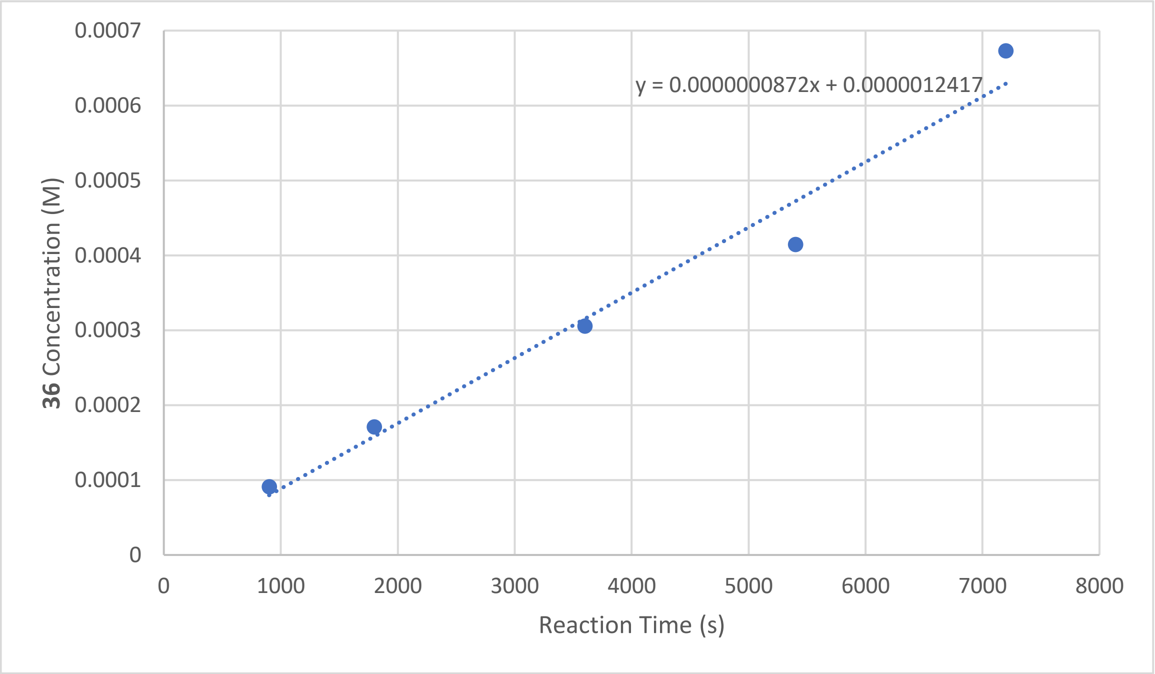

Supplementary Figure 23 Reaction Rate Using 0 mA

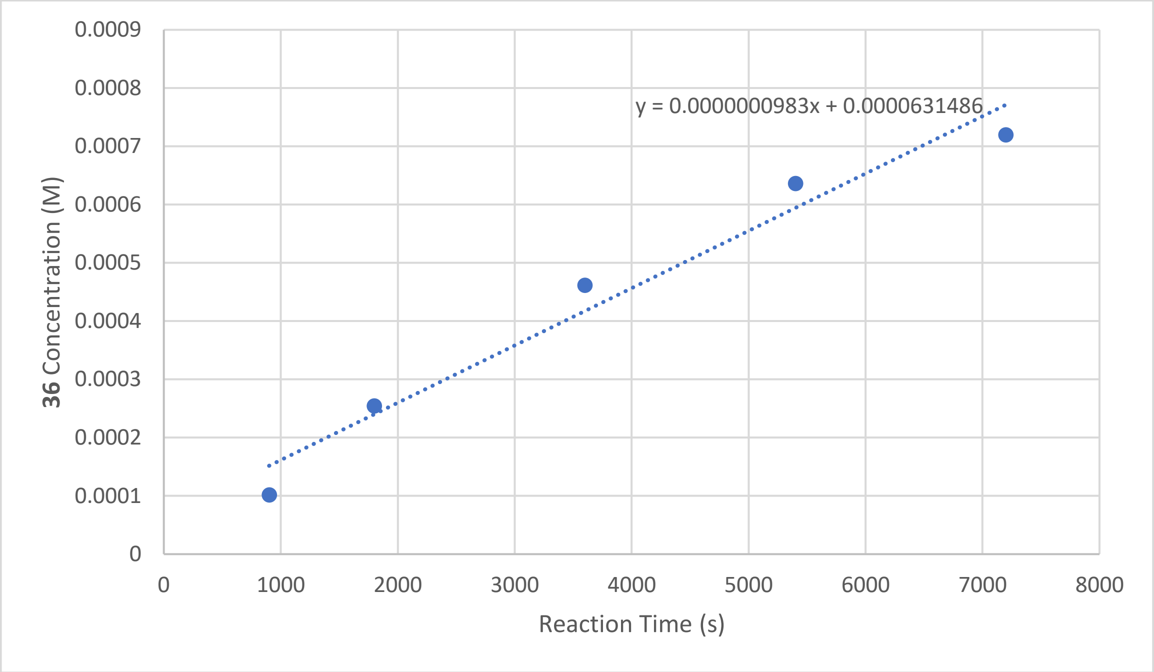

Supplementary Figure 24 Reaction Rate Using 0.8 mA

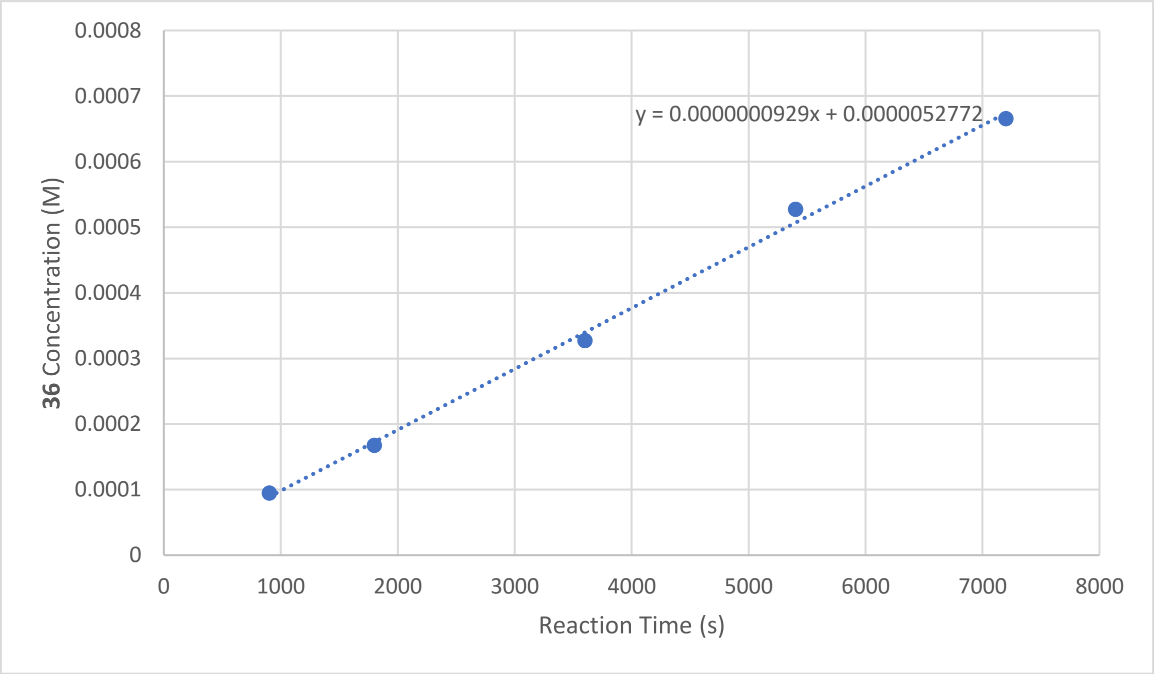

Supplementary Figure 25 Reaction Rate Using 1.0 mA

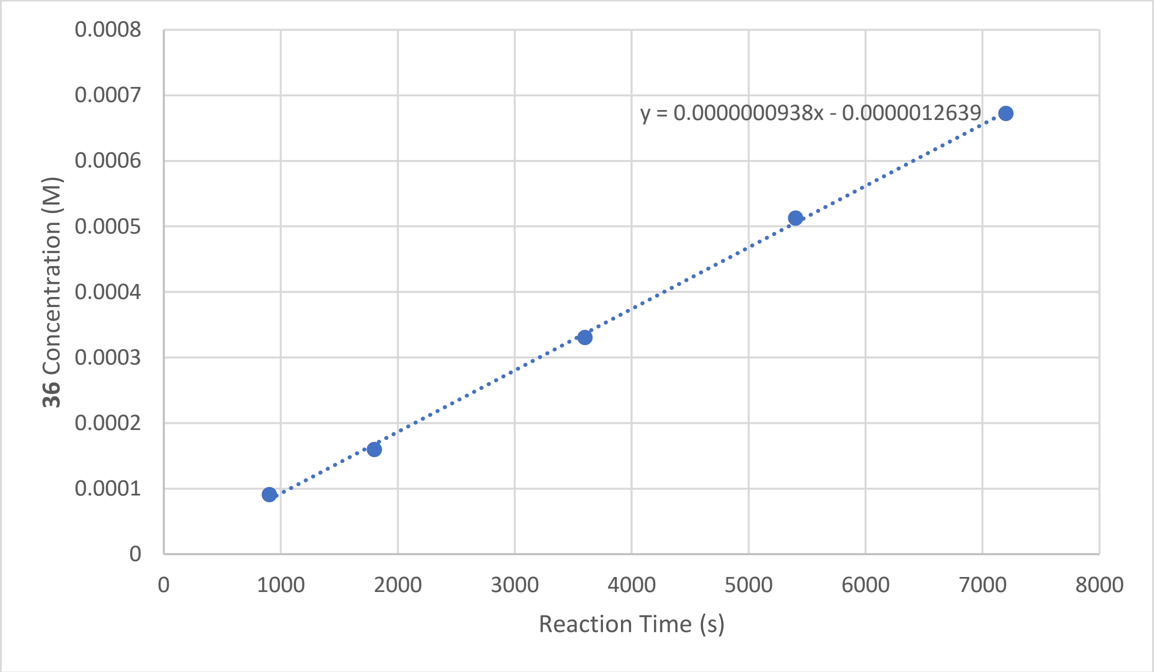

Supplementary Figure 26 Reaction Rate Using 1.1 mA

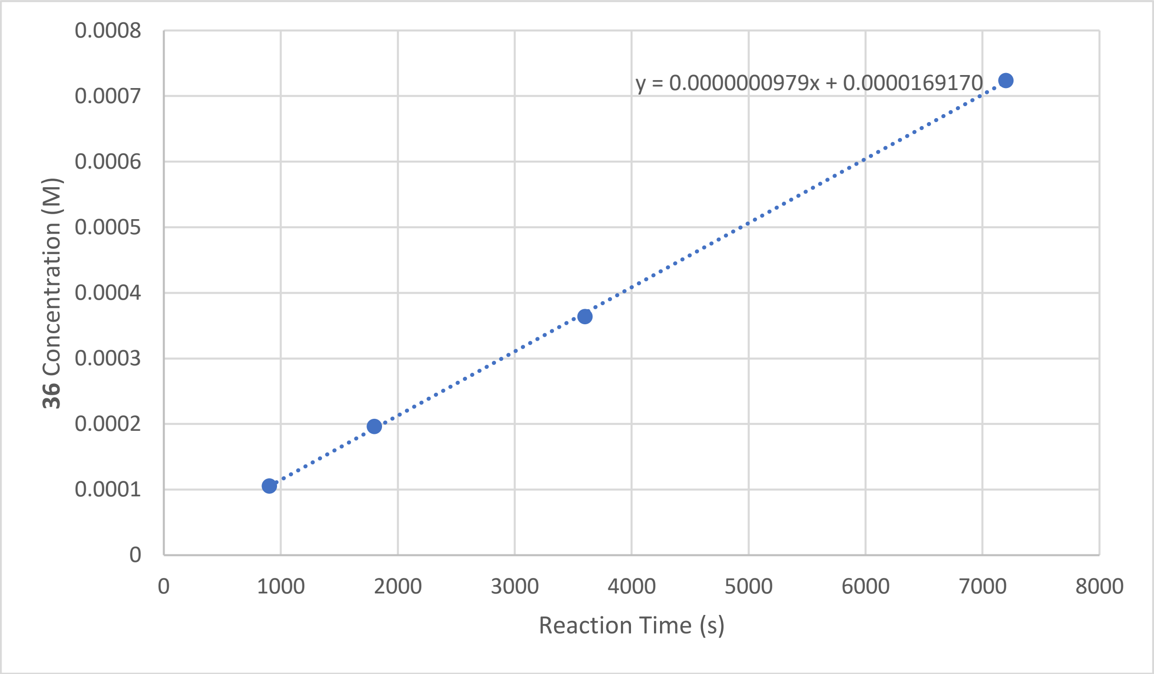

Supplementary Figure 27 Reaction Rate Using 1.2 mA

7.2.3 Kinetic Dependence of Initial Rate on [Cu]

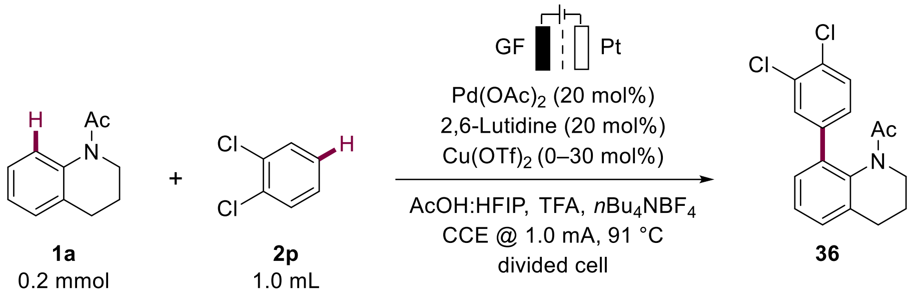

Reaction conditions: divided cell, anodic chamber: **1a** (0.20 mmol), **2p** (1.0 mL), $Pd(OAc)_2$ (20 mol %), 2,6-lutidine (20 mol %), $Cu(OTf)_2$ (0–30 mol %), $n$$Bu_4NBF_4$ (200 mg), HFIP:AcOH (4.0 mL, 1 : 2), TFA (0.5 mL), cathodic chamber: **2p** (1.0 mL), $n$$Bu_4NBF_4$ (200 mg), HFIP:AcOH (4.0 mL, 1 : 2), TFA (0.5 mL), 91 °C, electrolysis (CCE) at 1.0 mA, graphite felt (GF) anode (10 mm × 15 mm × 2 mm), Pt-plate cathode (10 mm × 15 mm × 0.25 mm), GC yield. At specific intervals shown on the horizontal axis, a small aliquot of the reaction mixture (0.1 mL) was taken, filtered through a small silica gel column, added with 0.1 mL internal standard stock solution (0.2 mmol dodecane in 5.5 mL EtOAc), and analyzed by GC. Only the β-isomer was quantified for the kinetics studies. To obtain a reliable result, data was generated by using the same reactor, potentiostat, heating plate, and GC.

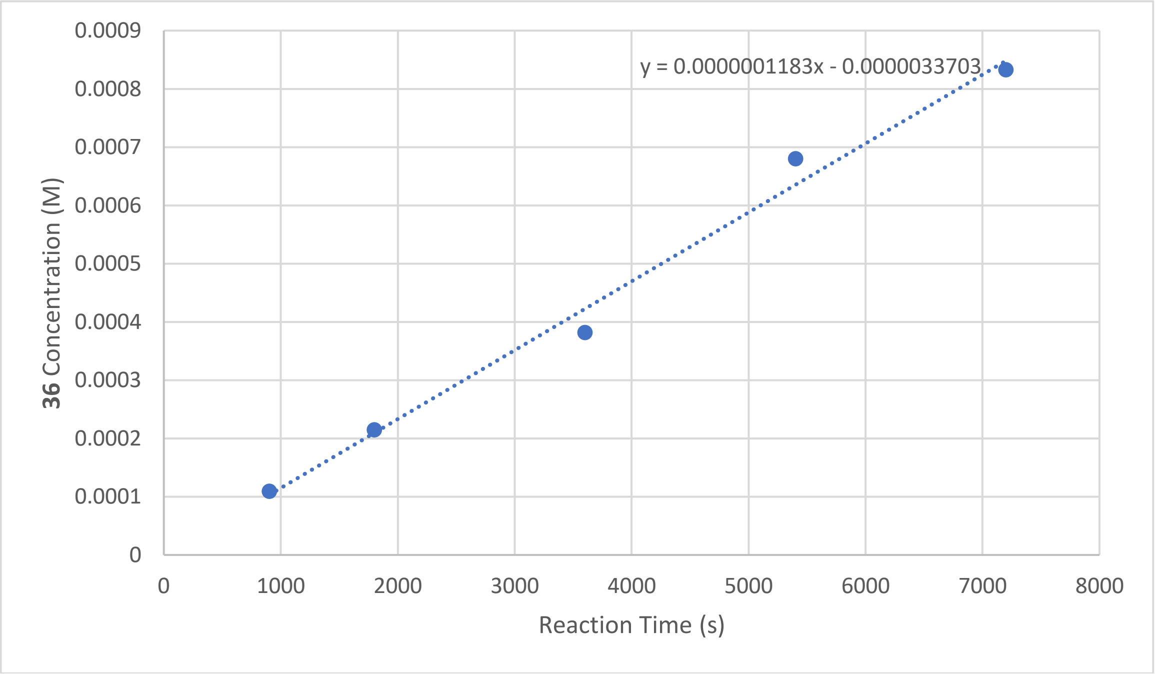

Supplementary Figure 28 Reaction Rate Using 0 mol % Cu

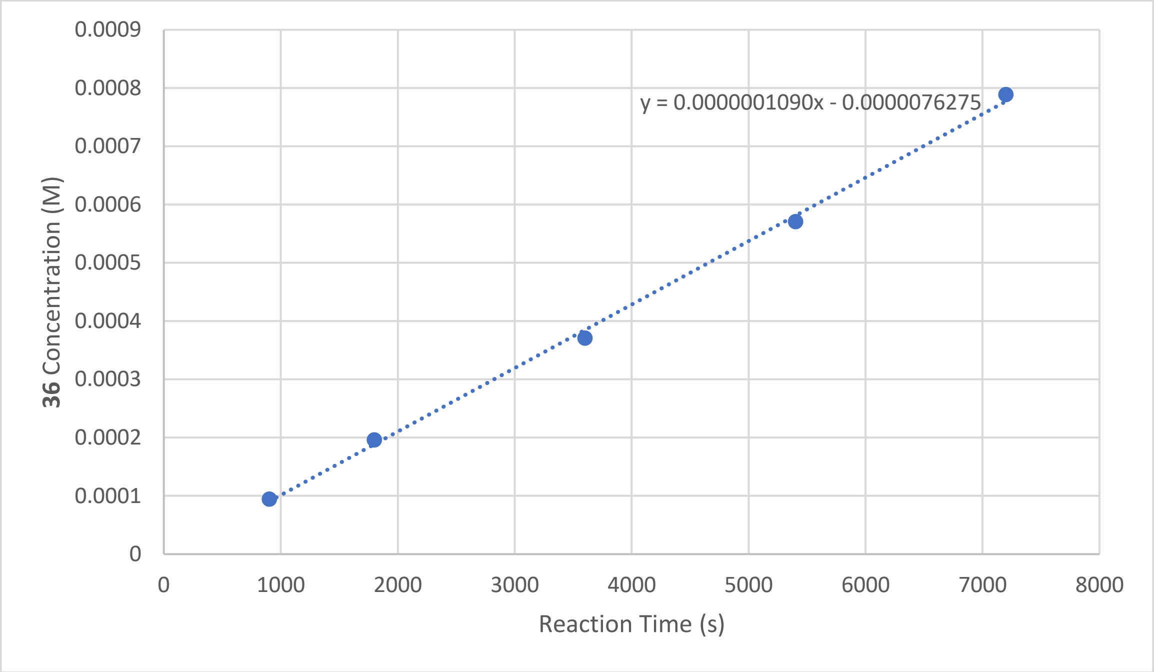

Supplementary Figure 29 Reaction Rate Using 10 mol % Cu

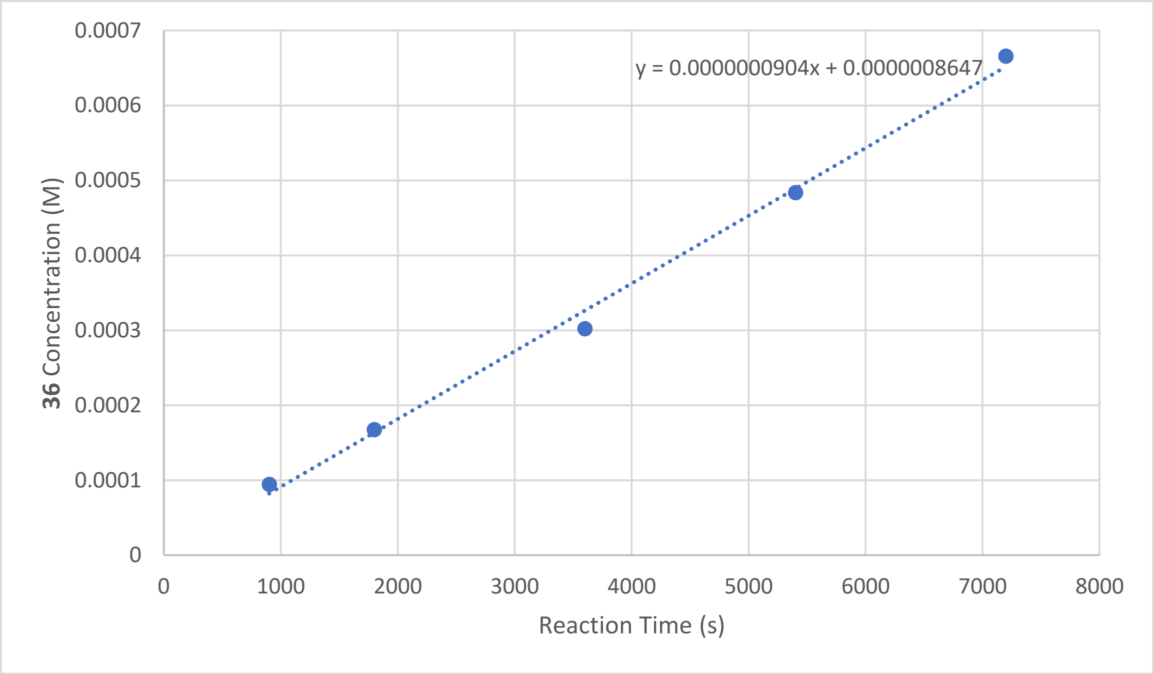

Supplementary Figure 30 Reaction Rate Using 20 mol % Cu

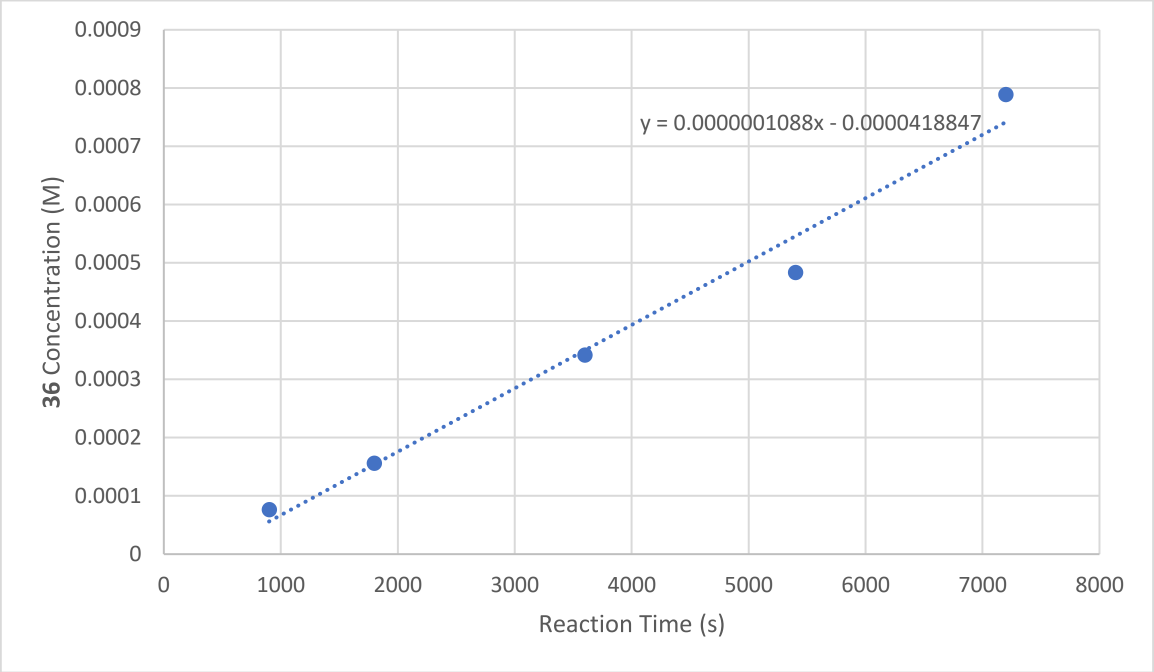

Supplementary Figure 31 Reaction Rate Using 30 mol % Cu

7.2.4 Kinetic Dependence of Initial Rate on [Lutidine]

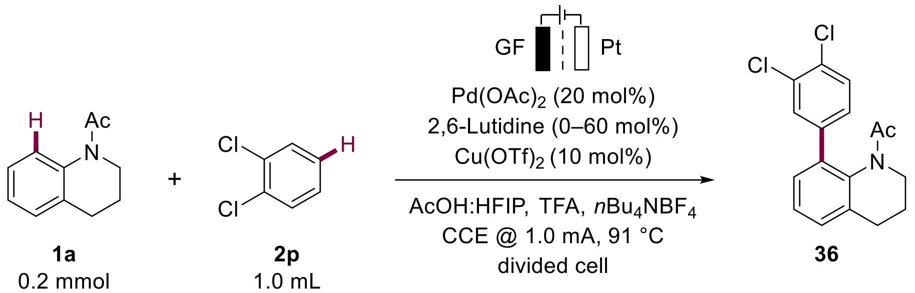

1a 0.2 mmol + **2p** 1.0 mL → **36**

Reaction conditions: divided cell, anodic chamber: **1a** (0.20 mmol), **2p** (1.0 mL), $Pd(OAc)_2$ (20 mol %), 2,6-lutidine (0–60 mol %), $Cu(OTf)_2$ (10 mol %), *n*Bu_4NBF_4 (200 mg), HFIP:AcOH (4.0 mL, 1 : 2), TFA (0.5 mL), cathodic chamber: **2p** (1.0 mL), *n*Bu_4NBF_4 (200 mg), HFIP:AcOH (4.0 mL, 1 : 2), TFA (0.5 mL), 91 °C, electrolysis (CCE) at 1.0 mA, graphite felt (GF) anode (10 mm × 15 mm × 2 mm), Pt-plate cathode (10 mm × 15 mm × 0.25 mm), GC yield. At specific intervals shown on the horizontal axis, a small aliquot of the reaction mixture (0.1 mL) was taken, filtered through a small silica gel column, added with 0.1 mL internal standard stock solution (0.2 mmol dodecane in 5.5 mL EtOAc), and analyzed by GC. Only the *β*-isomer was quantified for the kinetics studies. To obtain a reliable result, data was generated by using the same reactor, potentiostat, heating plate, and GC.

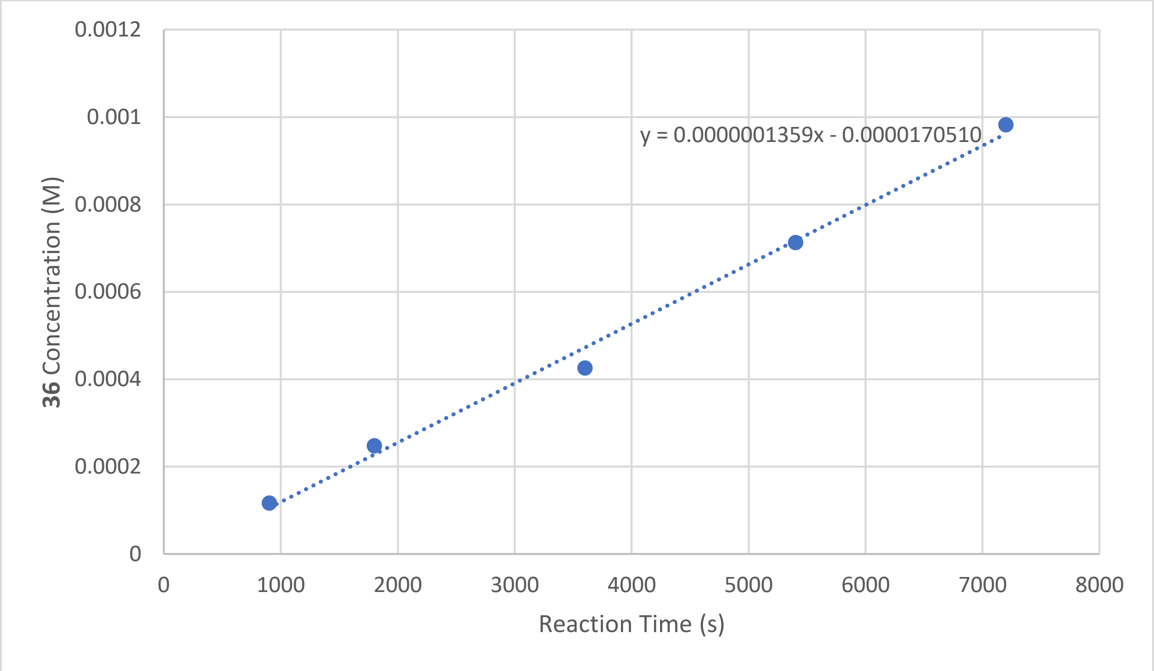

Supplementary Figure 32 Reaction Rate Using 0 mol % Lutidine

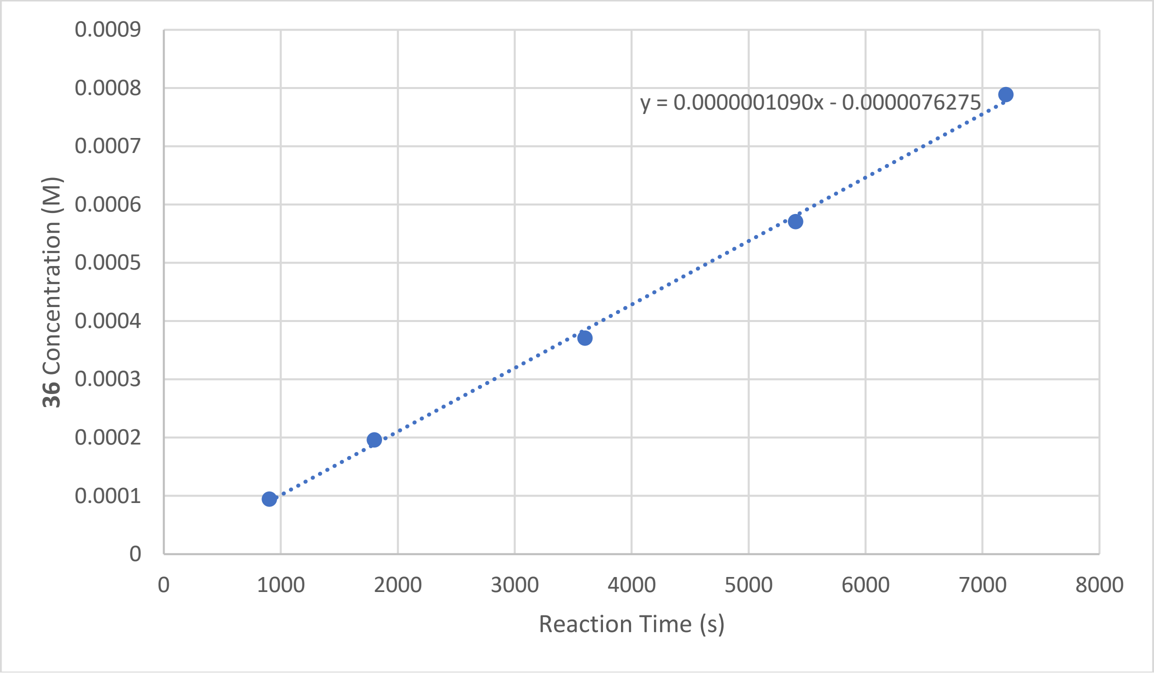

Supplementary Figure 33 Reaction Rate Using 20 mol % Lutidine

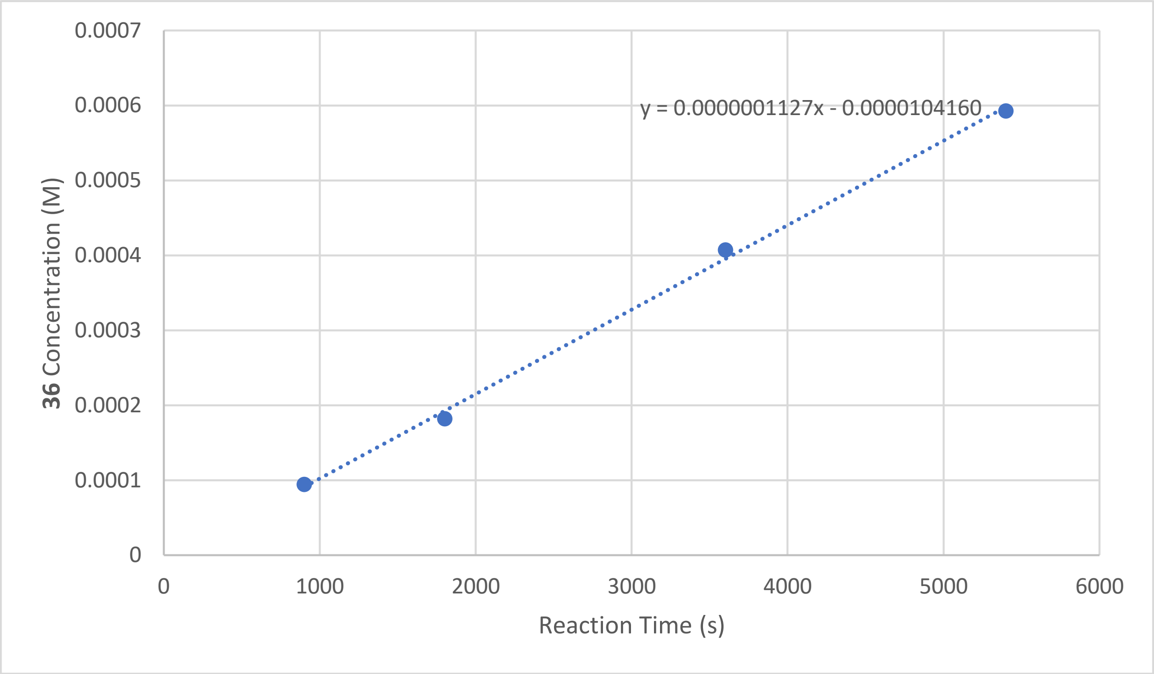

Supplementary Figure 34 Reaction Rate Using 40 mol % Lutidine

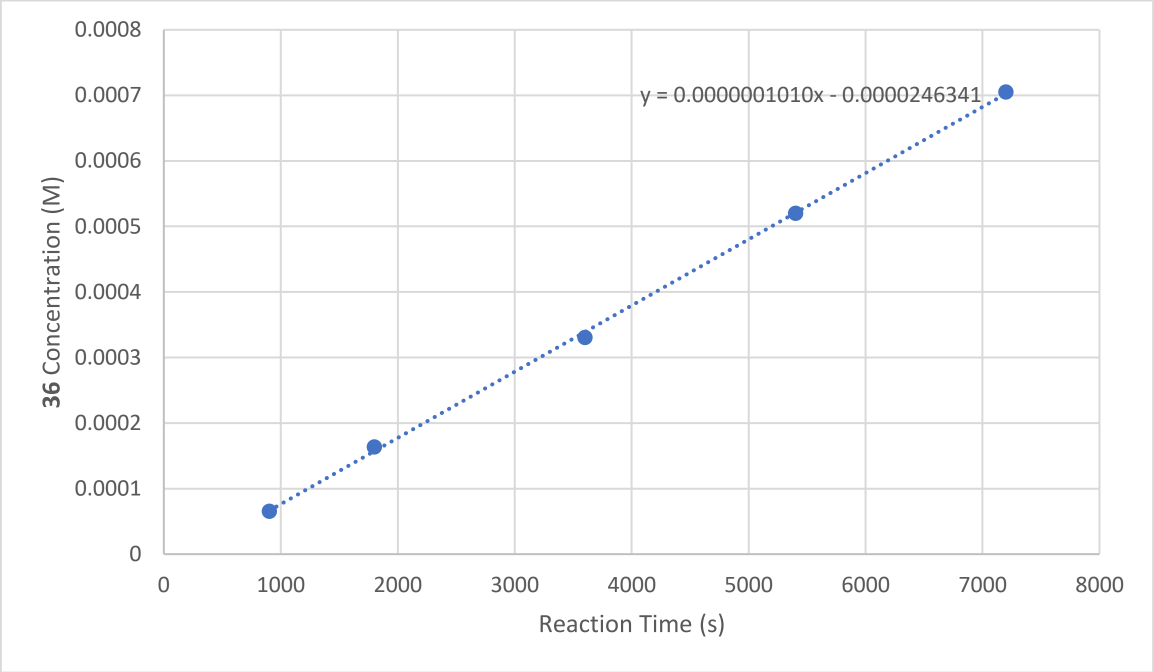

Supplementary Figure 35 Reaction Rate Using 60 mol % Lutidine

7.2.5 Kinetic Dependence of Initial Rate on [1a]

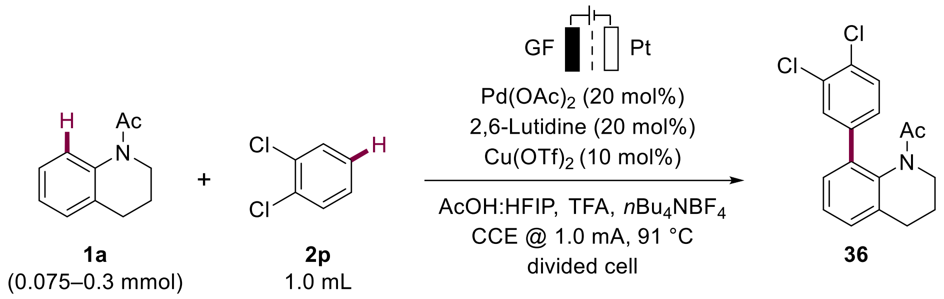

Reaction conditions: divided cell, anodic chamber: **1a** (0.075–0.3 mmol), **2p** (1.0 mL), $Pd(OAc)_2$ (20 mol %), 2,6-lutidine (20 mol %), $Cu(OTf)_2$ (10 mol %), $n\mathrm{Bu_4NBF_4}$ (200 mg), HFIP:AcOH (4.0 mL, 1 : 2), TFA (0.5 mL), cathodic chamber: **2p** (1.0 mL), $n\mathrm{Bu_4NBF_4}$ (200 mg), HFIP:AcOH (4.0 mL, 1 : 2), TFA (0.5 mL), 91 °C, electrolysis (CCE) at 1.0 mA, graphite felt (GF) anode (10 mm × 15 mm × 2 mm), Pt-plate cathode (10 mm × 15 mm × 0.25 mm), GC yield. At specific intervals shown on the horizontal axis, a small aliquot of the reaction mixture (0.1 mL) was taken, filtered through a small silica gel column, added with 0.1 mL internal standard stock solution (0.2 mmol dodecane in 5.5 mL EtOAc), and analyzed by GC. Only the β-isomer was quantified for the kinetics studies. To obtain a reliable result, data was generated by using the same reactor, potentiostat, heating plate, and GC.

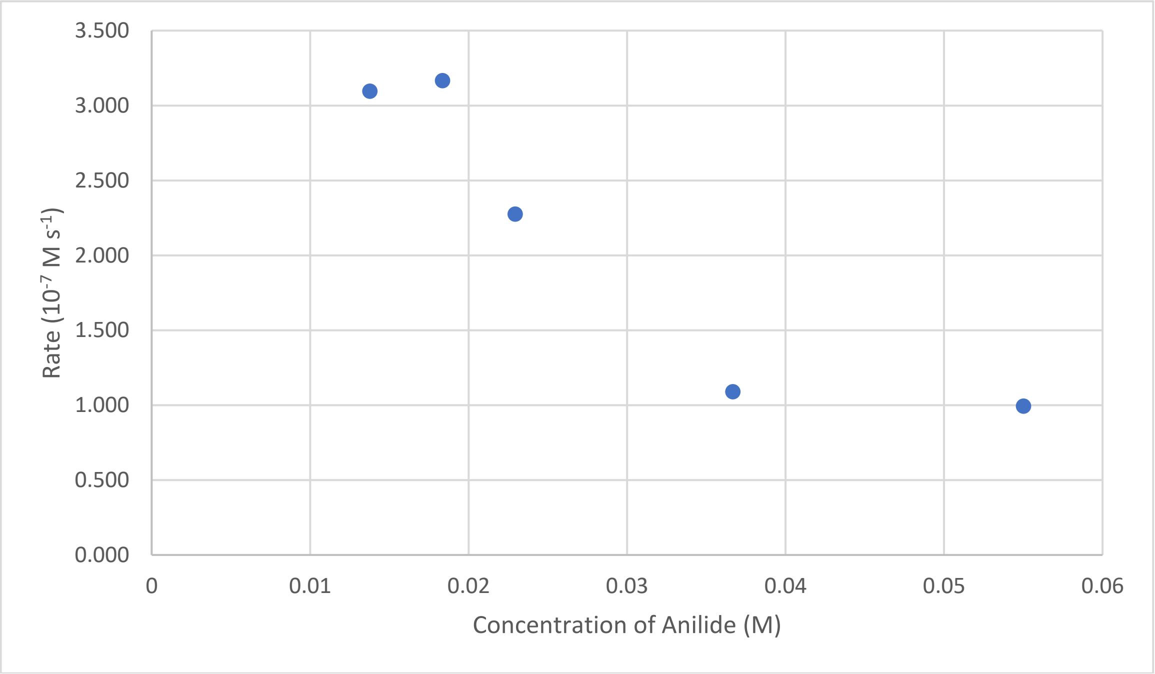

Supplementary Figure 36 Reverse Order on [1a]

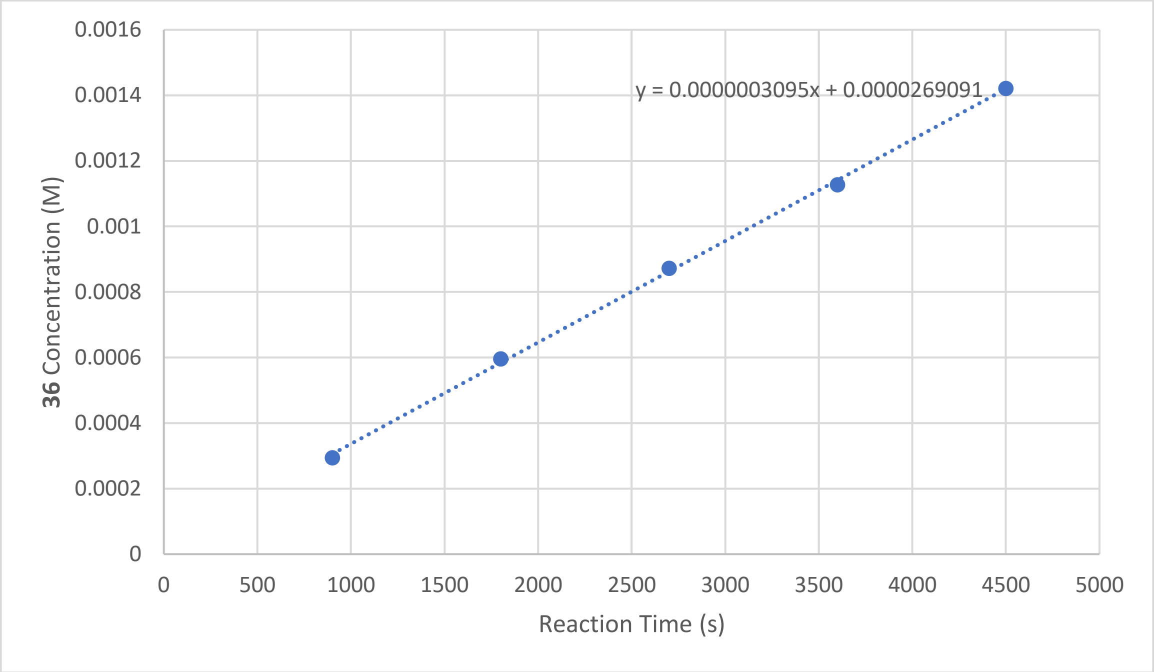

Supplementary Figure 37 Reaction Rate Using 0.075 mmol 1a

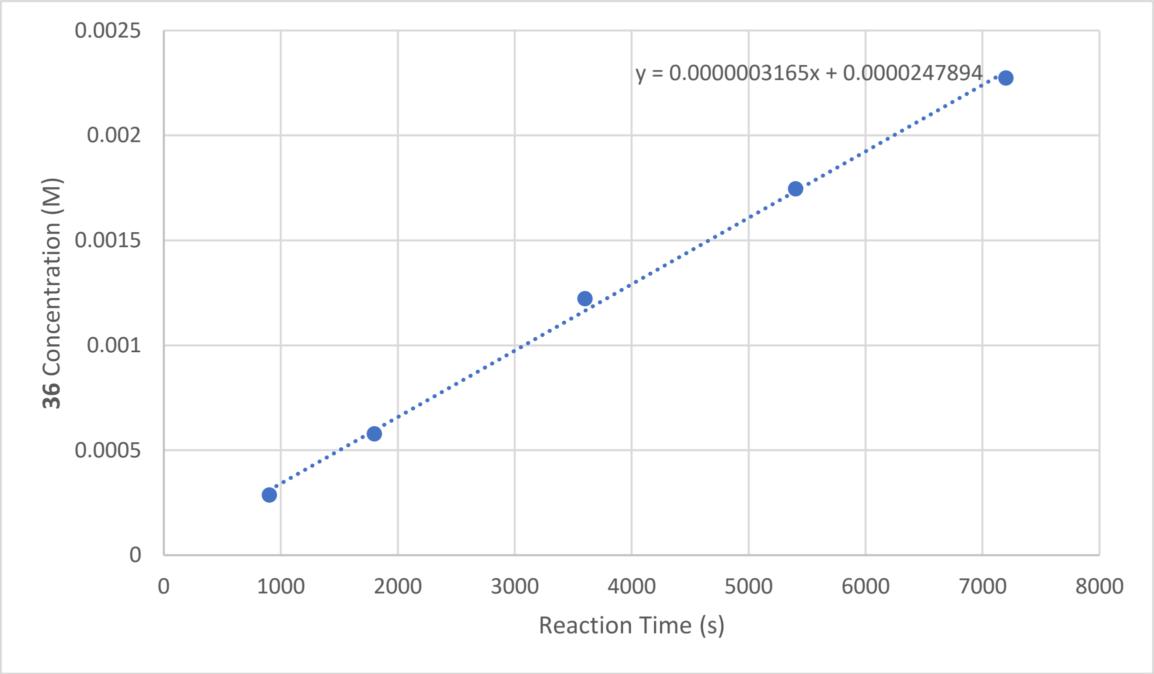

Supplementary Figure 38 Reaction Rate Using 0.1 mmol 1a

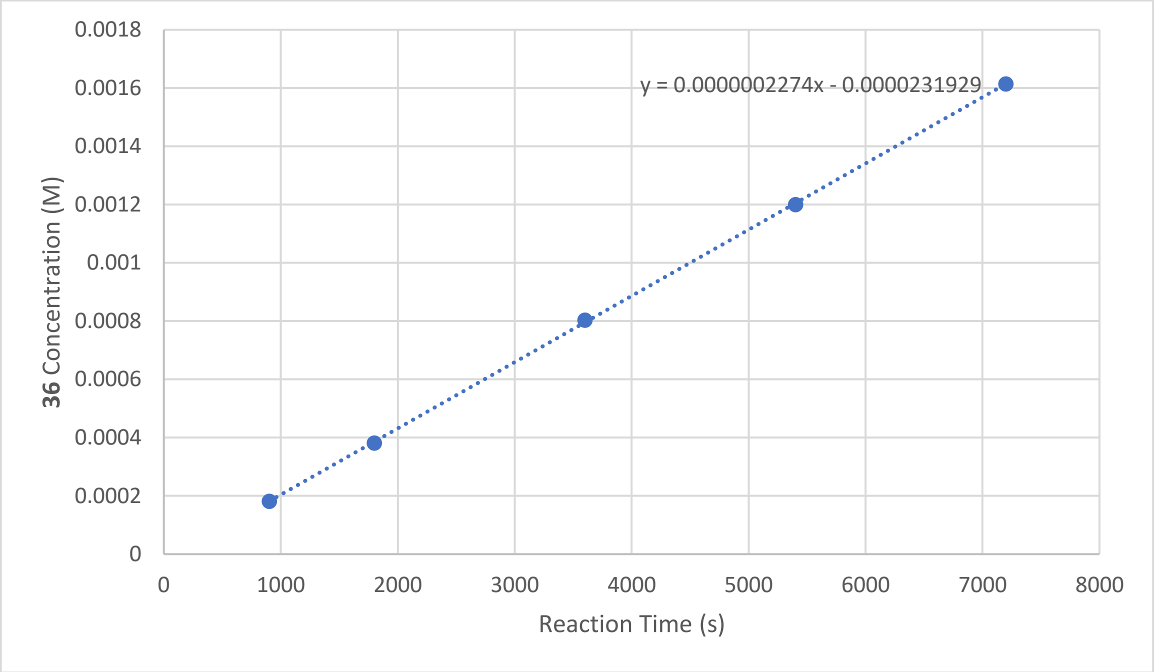

Supplementary Figure 39 Reaction Rate Using 0.125 mmol 1a

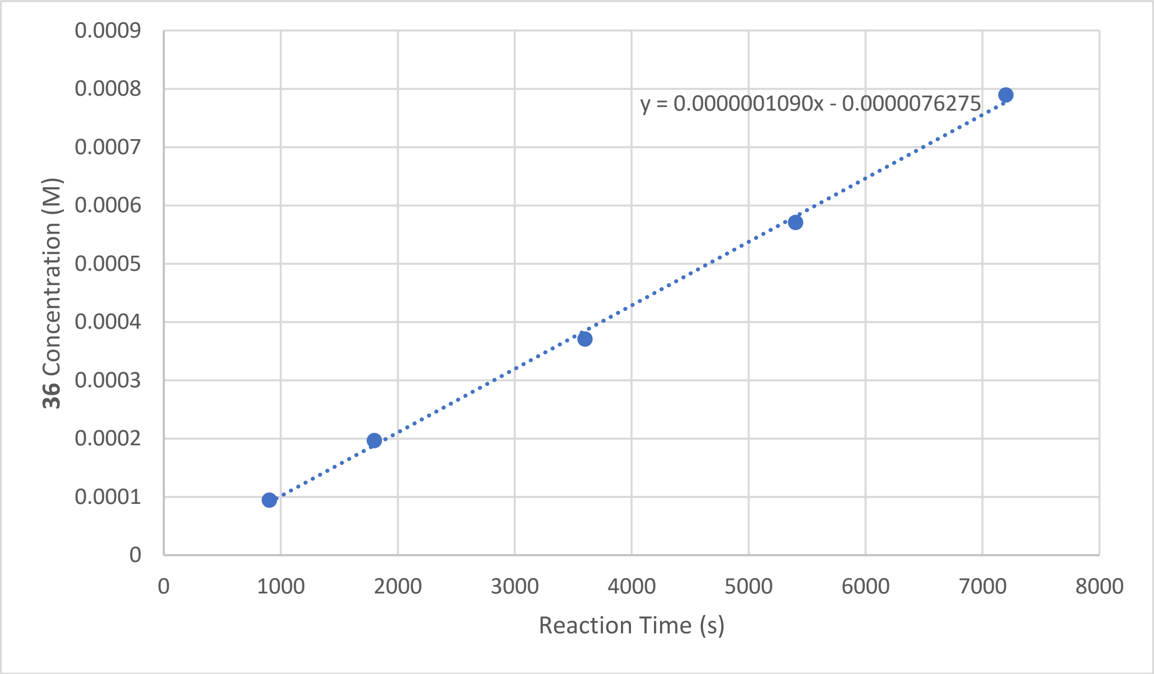

Supplementary Figure 40 Reaction Rate Using 0.2 mmol 1a

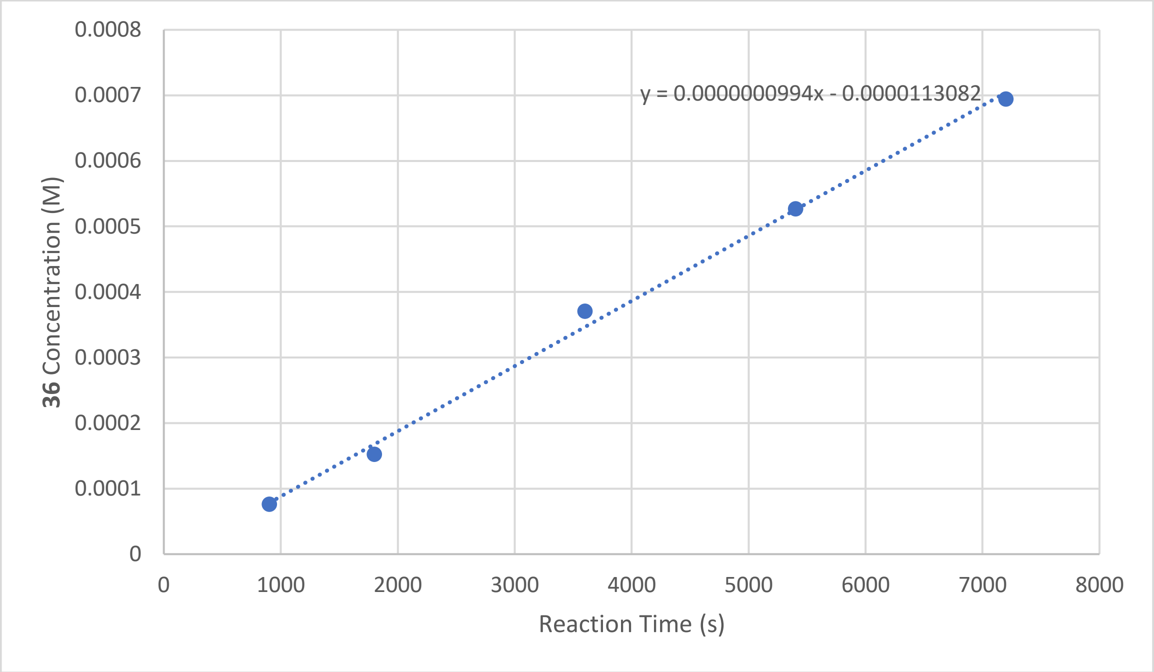

Supplementary Figure 41 Reaction Rate Using 0.3 mmol 1a

7.2.6 Kinetic Dependence of Initial Rate on [2p]

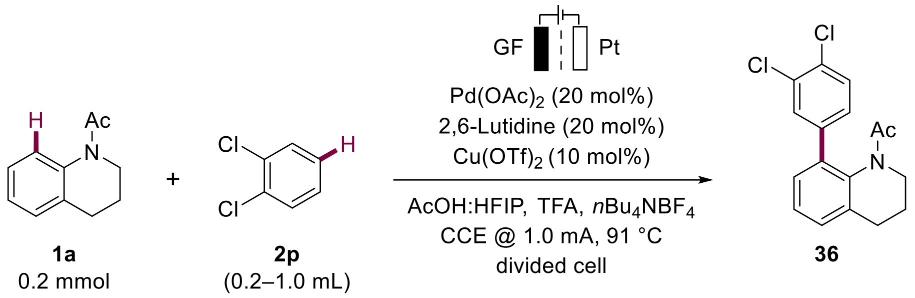

1a 0.2 mmol + **2p** (0.2–1.0 mL) → **36**

Reaction conditions: divided cell, anodic chamber: **1a** (0.2 mmol), **2p** (0.2–1.0 mL), $Pd(OAc)_2$ (20 mol %), 2,6-lutidine (20 mol %), $Cu(OTf)_2$ (10 mol %), $n Bu_4NBF_4$ (200 mg), HFIP:AcOH (4.0 mL, 1 : 2), TFA (0.5 mL), cathodic chamber: **2p** (0.2–1.0 mL), $n Bu_4NBF_4$ (200 mg), HFIP:AcOH (4.0 mL, 1 : 2), TFA (0.5 mL), 91 °C, electrolysis (CCE) at 1.0 mA, graphite felt (GF) anode (10 mm × 15 mm × 2 mm), Pt-plate cathode (10 mm × 15 mm × 0.25 mm), GC yield. At specific intervals shown on the horizontal axis, a small aliquot of the reaction mixture (0.1 mL) was taken, filtered through a small silica gel column, added with 0.1 mL internal standard stock solution (0.2 mmol dodecane in 4.7 mL, 4.8 mL, 4.9 mL, 5.0 mL, or 5.5 mL EtOAc), and analyzed by GC. Only the *β*-isomer was quantified for the kinetics studies. To obtain a reliable result, data was generated by using the same reactor, potentiostat, heating plate, and GC.

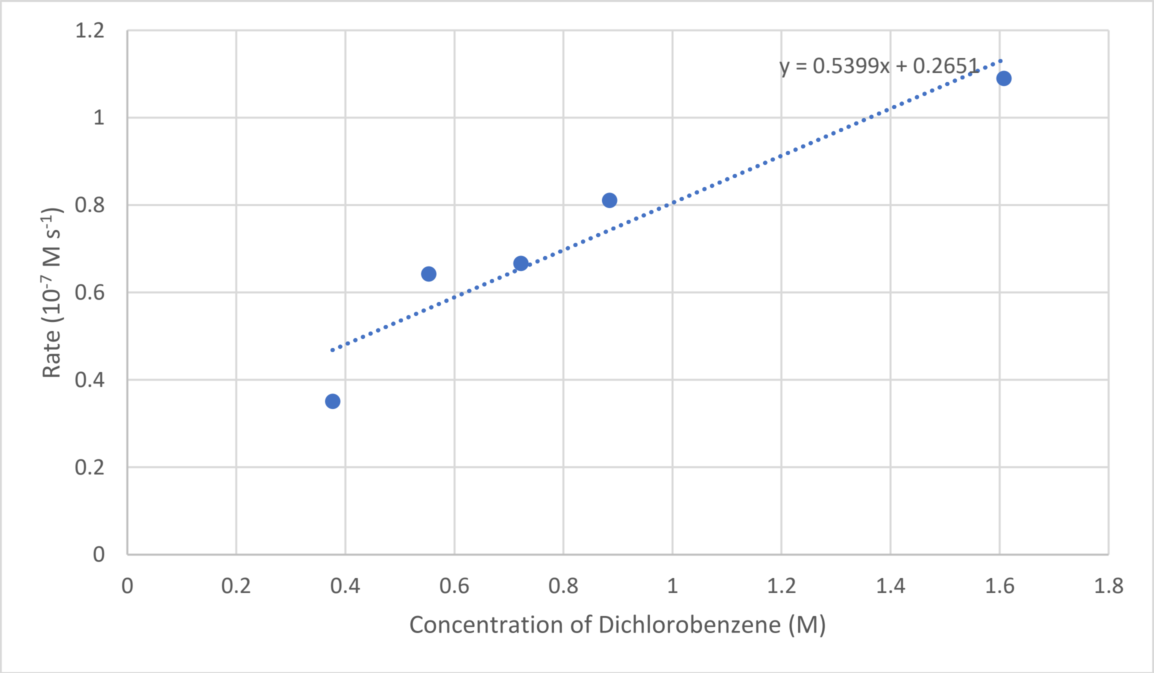

Supplementary Figure 42 First Order on [2p] with the Fifth Point

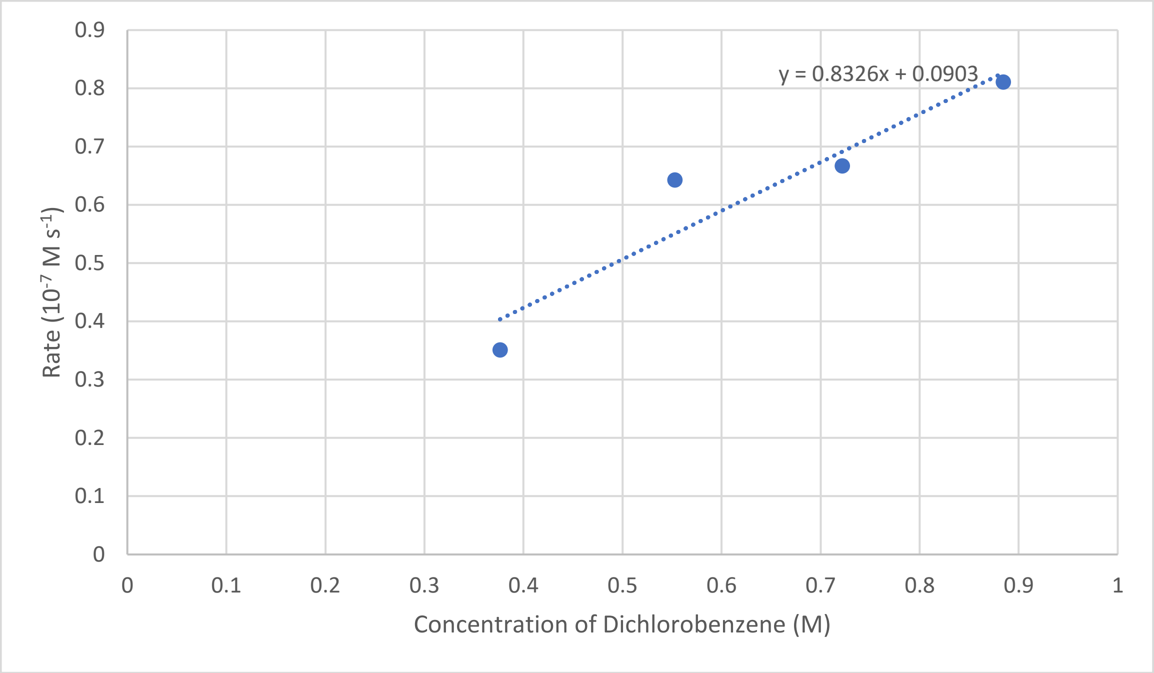

Supplementary Figure 43 First Order on [2p] without the Fifth Point

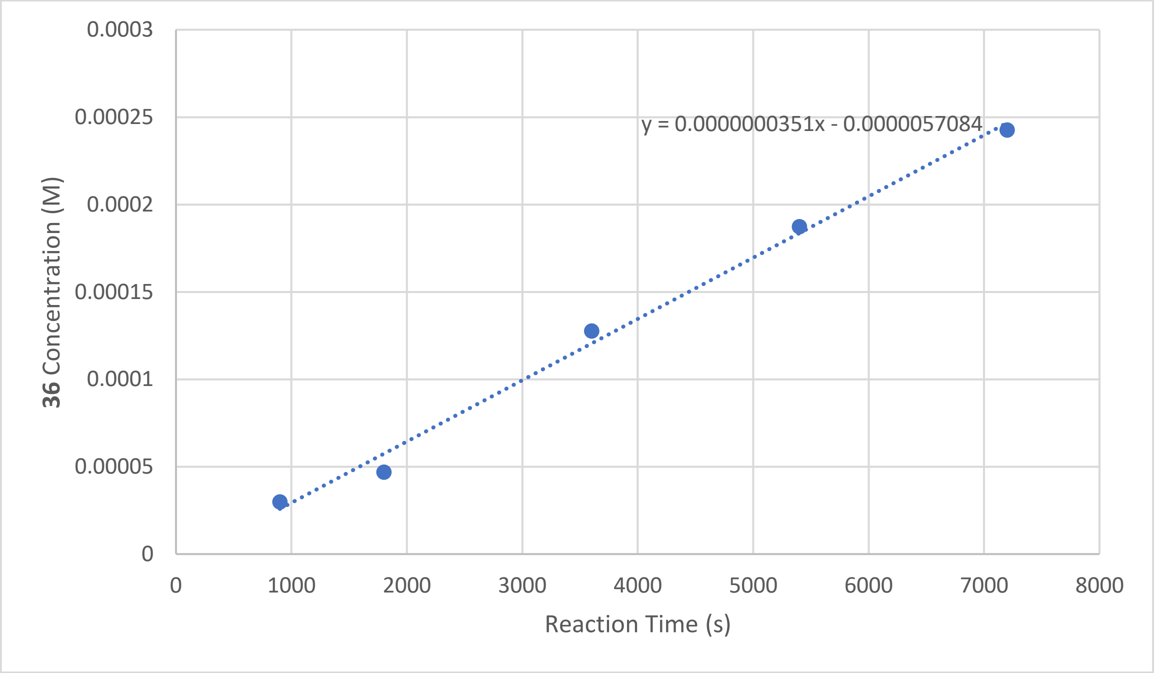

Supplementary Figure 44 Reaction Rate Using 0.2 mL 2p

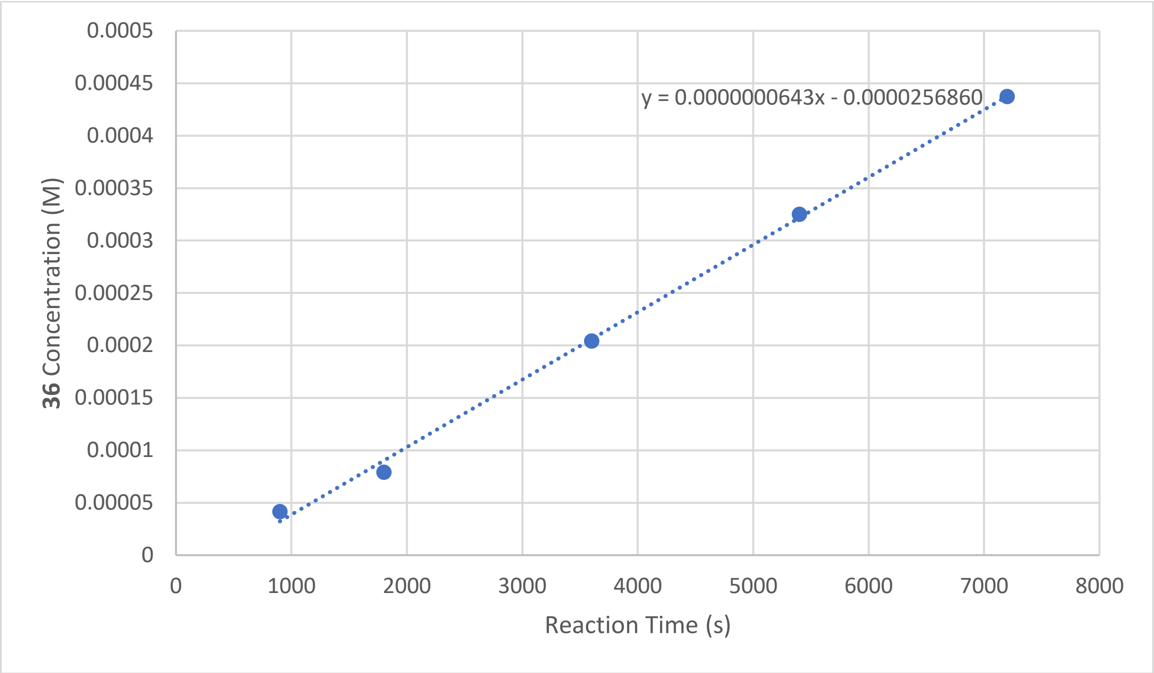

Supplementary Figure 45 Reaction Rate Using 0.3 mL 2p

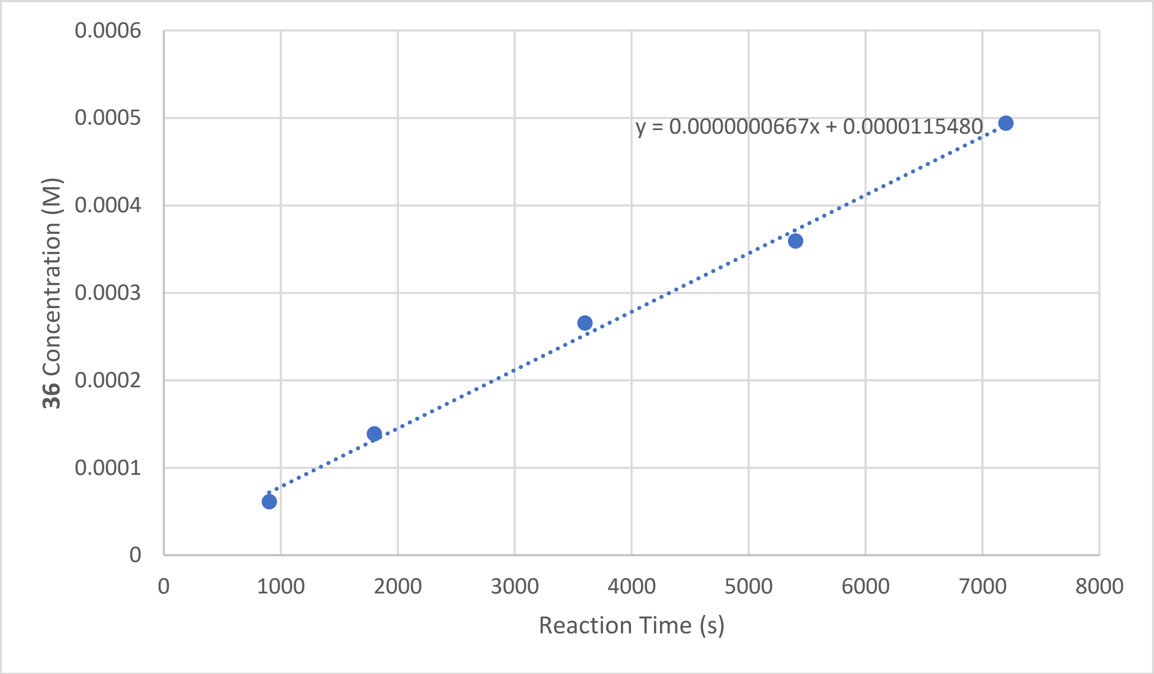

Supplementary Figure 46 Reaction Rate Using 0.4 mL 2p

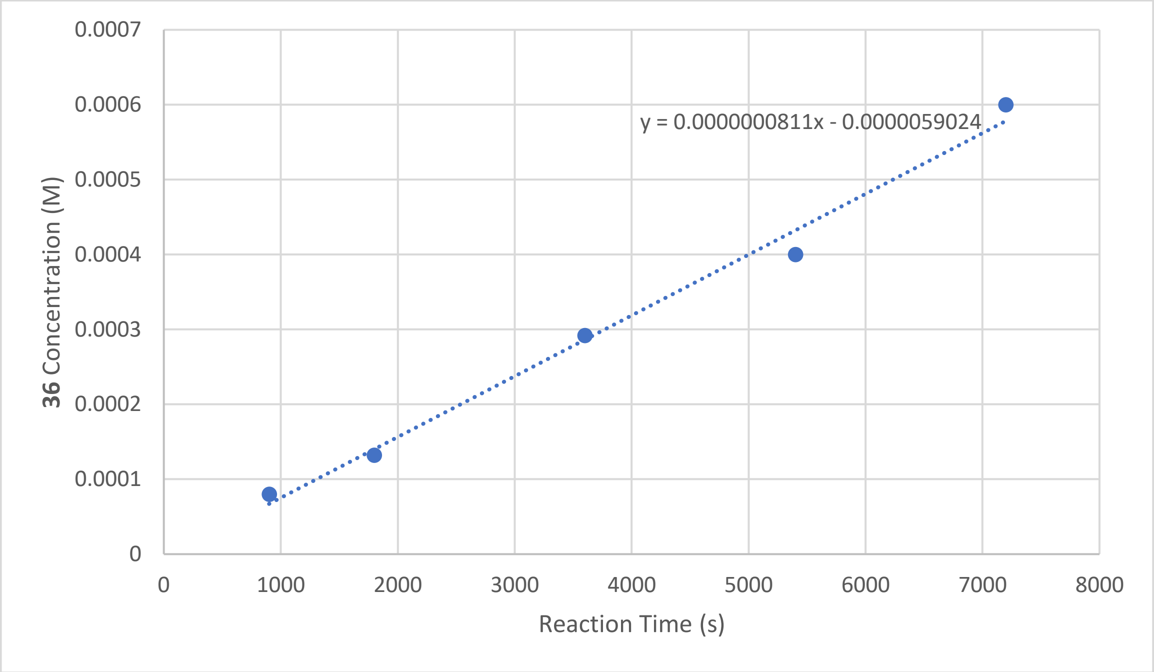

Supplementary Figure 47 Reaction Rate Using 0.5 mL 2p

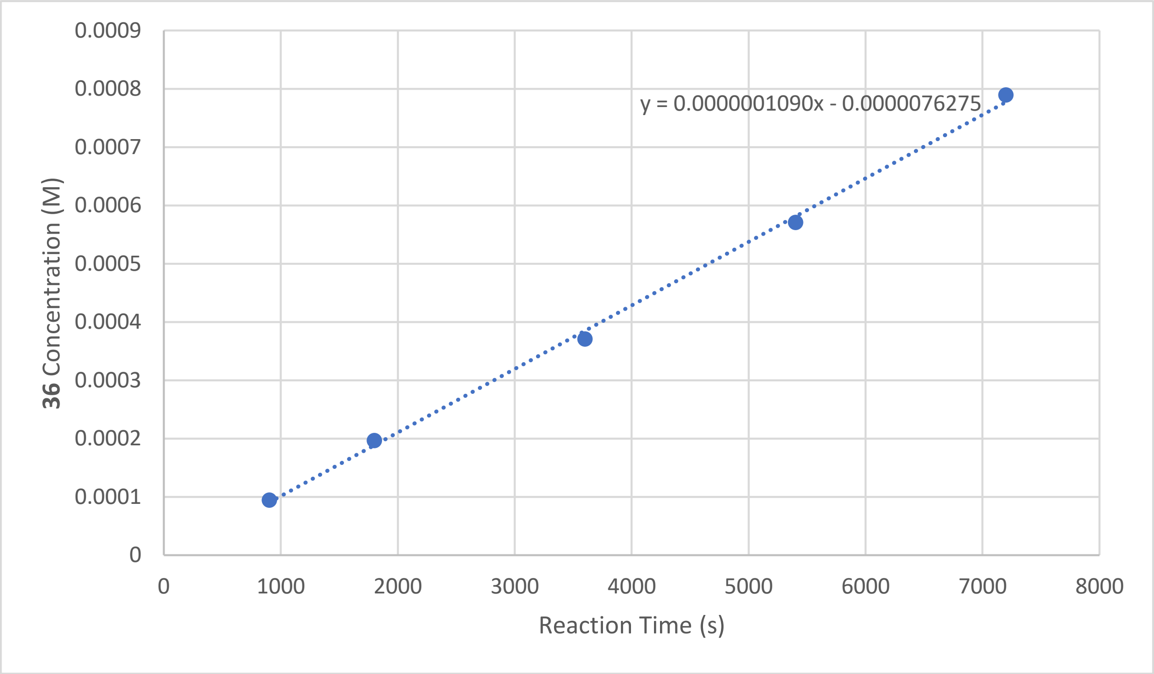

Supplementary Figure 48 Reaction Rate Using 1.0 mL 2p

7.2.7 Kinetic Dependence of Initial Rate on Temperature

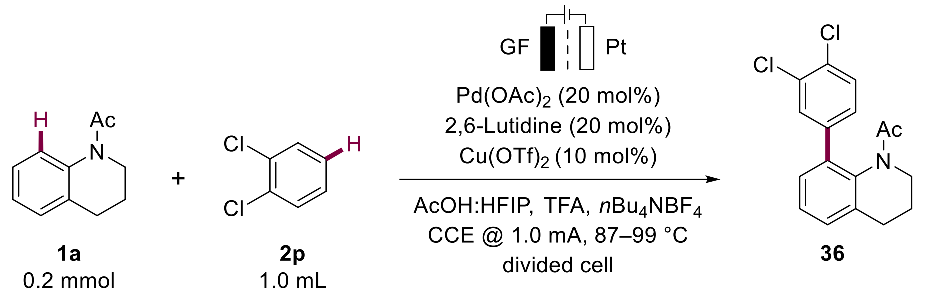

Reaction conditions: divided cell, anodic chamber: **1a** (0.20 mmol), **2p** (1.0 mL), $Pd(OAc)_2$ (20 mol %), 2,6-lutidine (20 mol %), $Cu(OTf)_2$ (10 mol %), $n$$Bu_4NBF_4$ (200 mg), HFIP:AcOH (4.0 mL, 1 : 2), TFA (0.5 mL), cathodic chamber: **2p** (1.0 mL), $n$$Bu_4NBF_4$ (200 mg), HFIP:AcOH (4.0 mL, 1 : 2), TFA (0.5 mL), temperature 87–99 °C, electrolysis (CCE) at 1.0 mA, graphite felt (GF) anode (10 mm × 15 mm × 2 mm), Pt-plate cathode (10 mm × 15 mm × 0.25 mm), GC yield. At specific intervals shown on the horizontal axis, a small aliquot of the reaction mixture (0.1 mL) was taken, filtered through a small silica gel column, added with 0.1 mL internal standard stock solution (0.2 mmol dodecane in 5.5 mL EtOAc), and analyzed by GC. Only the β-isomer was quantified for the kinetics studies. To obtain a reliable result, data was generated by using the same reactor, potentiostat, heating plate, and GC.

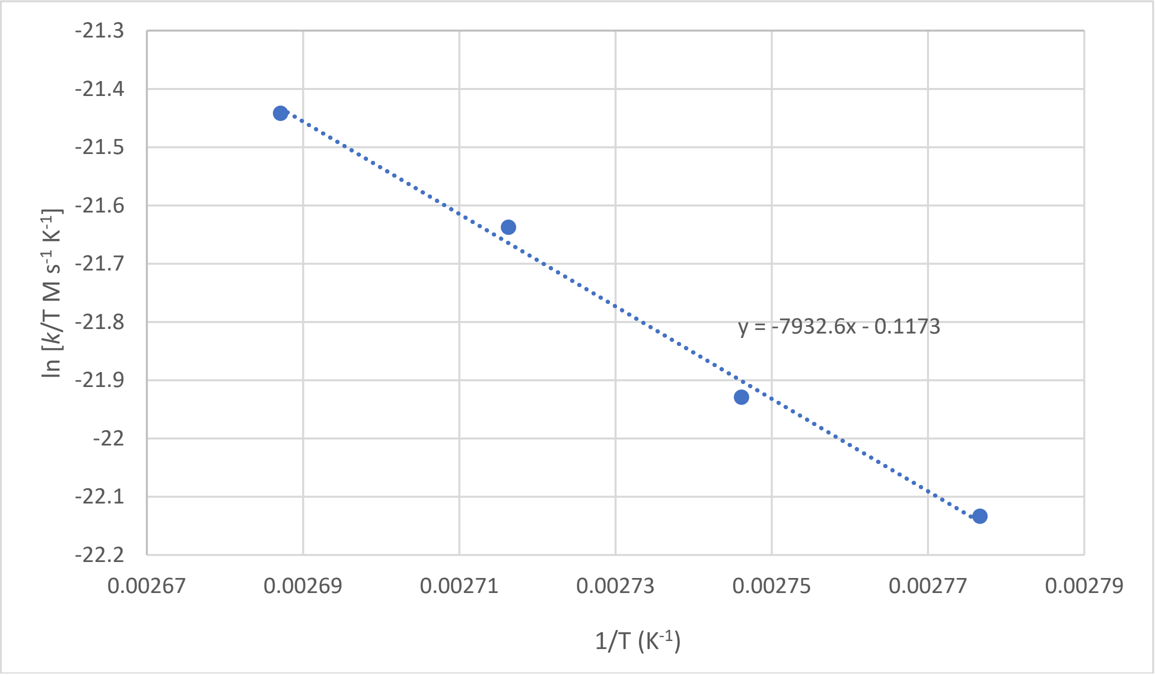

Supplementary Figure 49 Eyring Plot

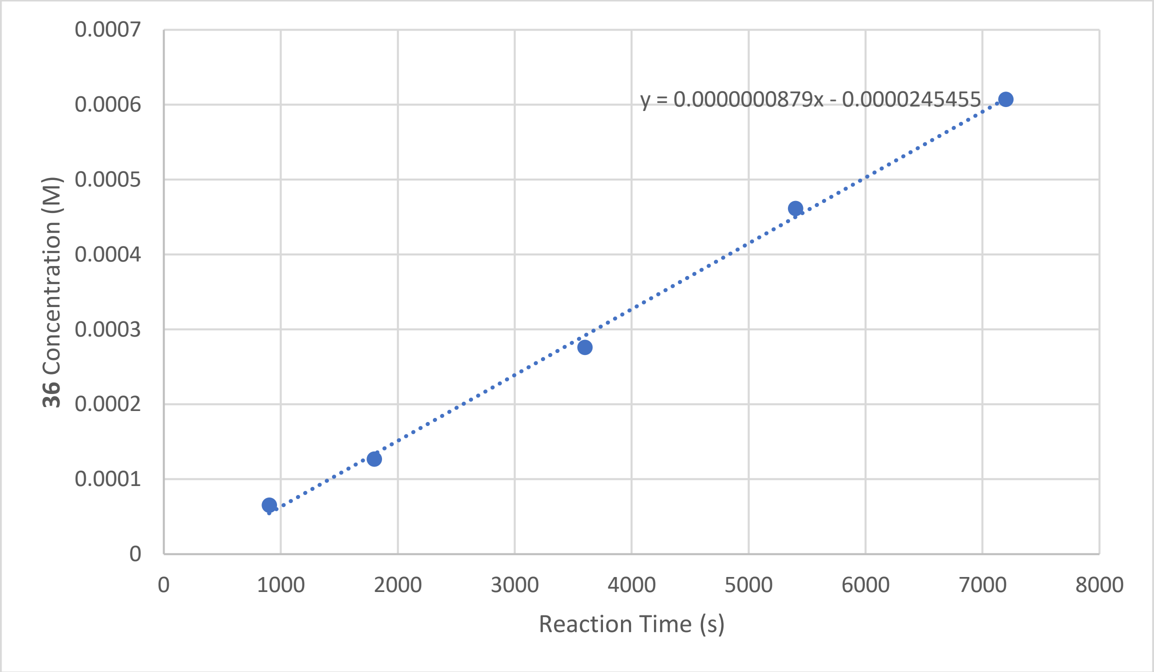

Supplementary Figure 50 Reaction Rate at 87 °C

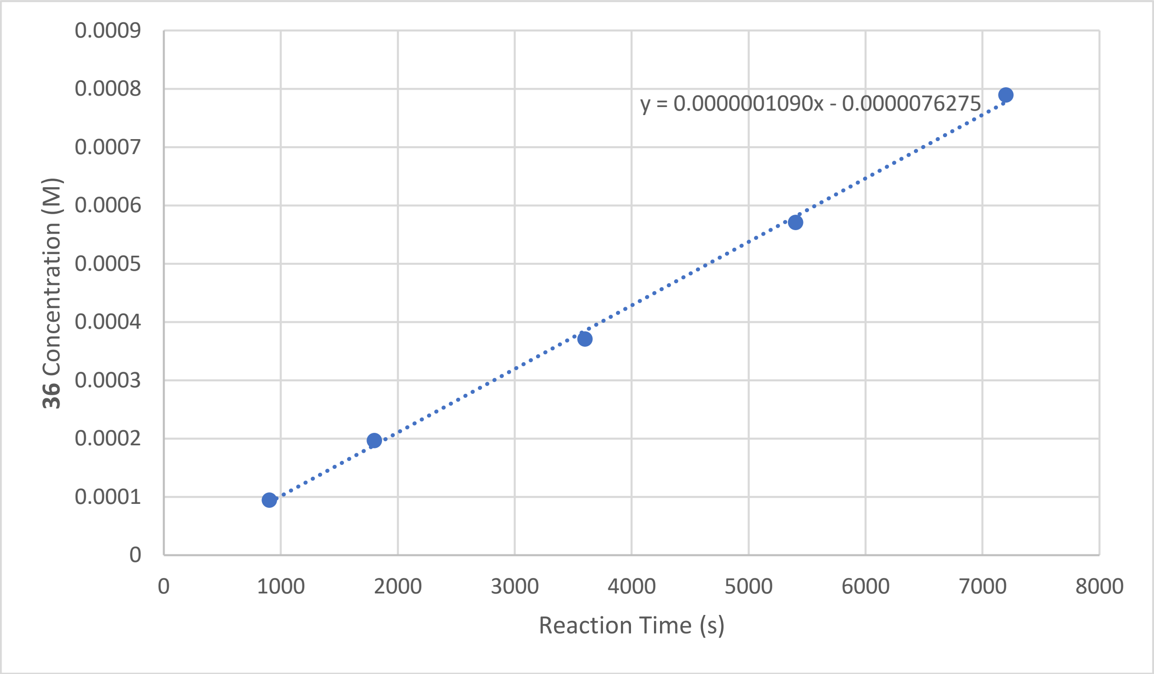

Supplementary Figure 51 Reaction Rate at 91 °C

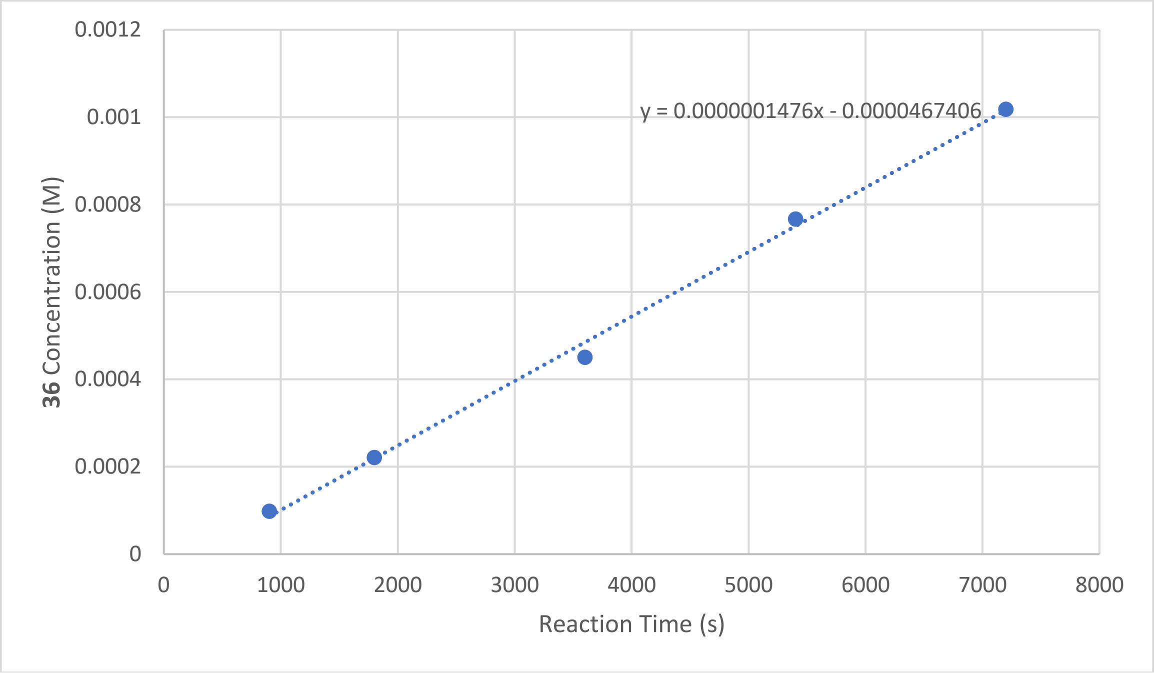

Supplementary Figure 52 Reaction Rate at 95 °C

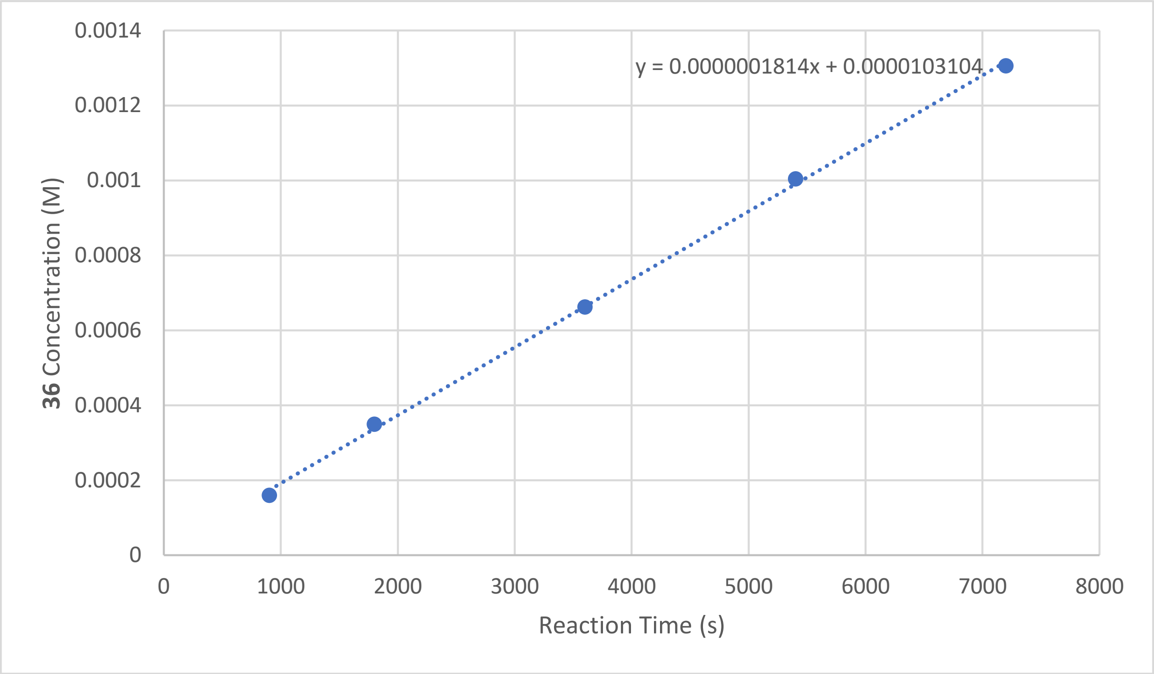

Supplementary Figure 53 Reaction Rate at 99 °C

Higher temperature did bring about faster initial rate, however, due to the boiling point of TFA, losing TFA at higher temperature conditions during the course of the reaction could lead to a lower overall yield.

7.3 H/D Exchange

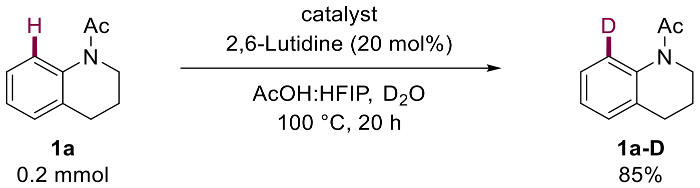

Reaction conditions: **1a** (0.20 mmol), $Pd(OAc)_2$ (10 mol %) or $Cu(OTf)_2$ (10 mol %), 2,6-lutidine (20 mol %, HFIP:AcOH (0.3 mL : 0.6 mL) were added to a 10 mL glass vial, 100 °C, 20 h. After the reaction, the reaction solution was filtered and transferred to NMR tube for crude H-NMR analysis.

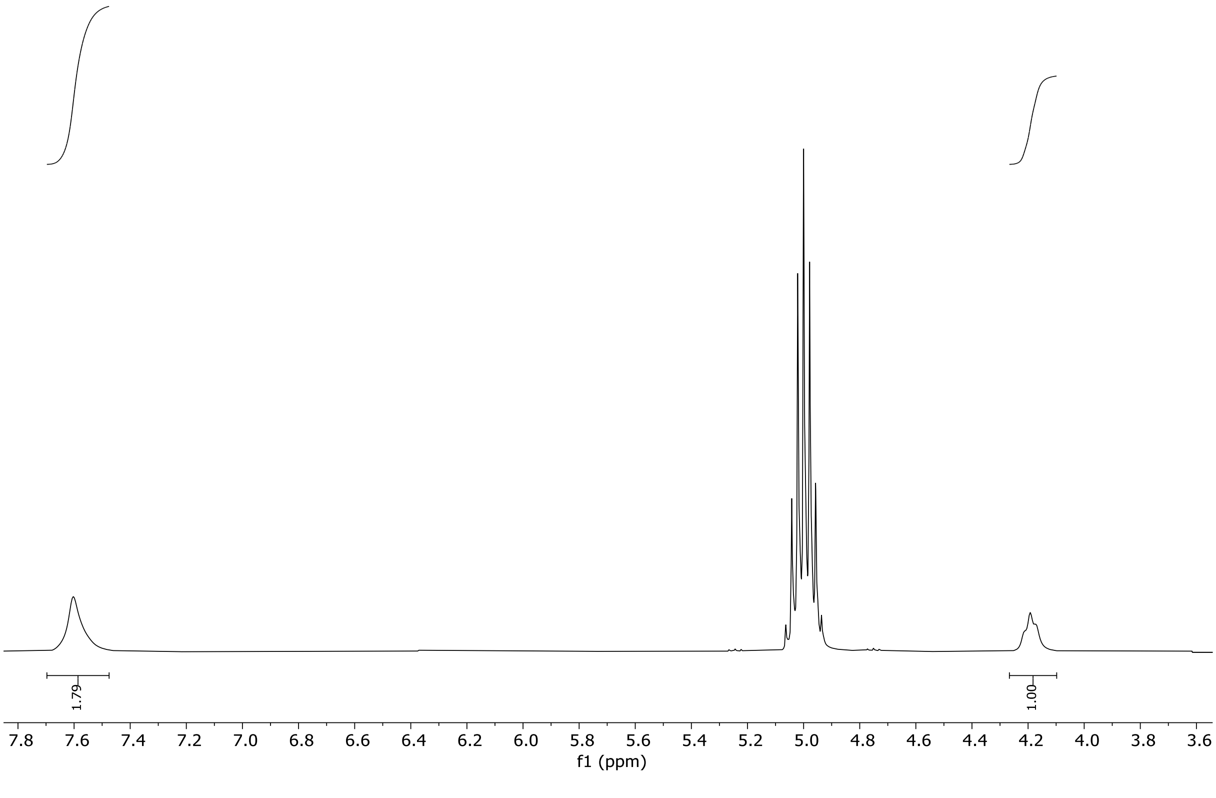

Supplementary Figure 54 H/D Exchange of 1a without Catalyst

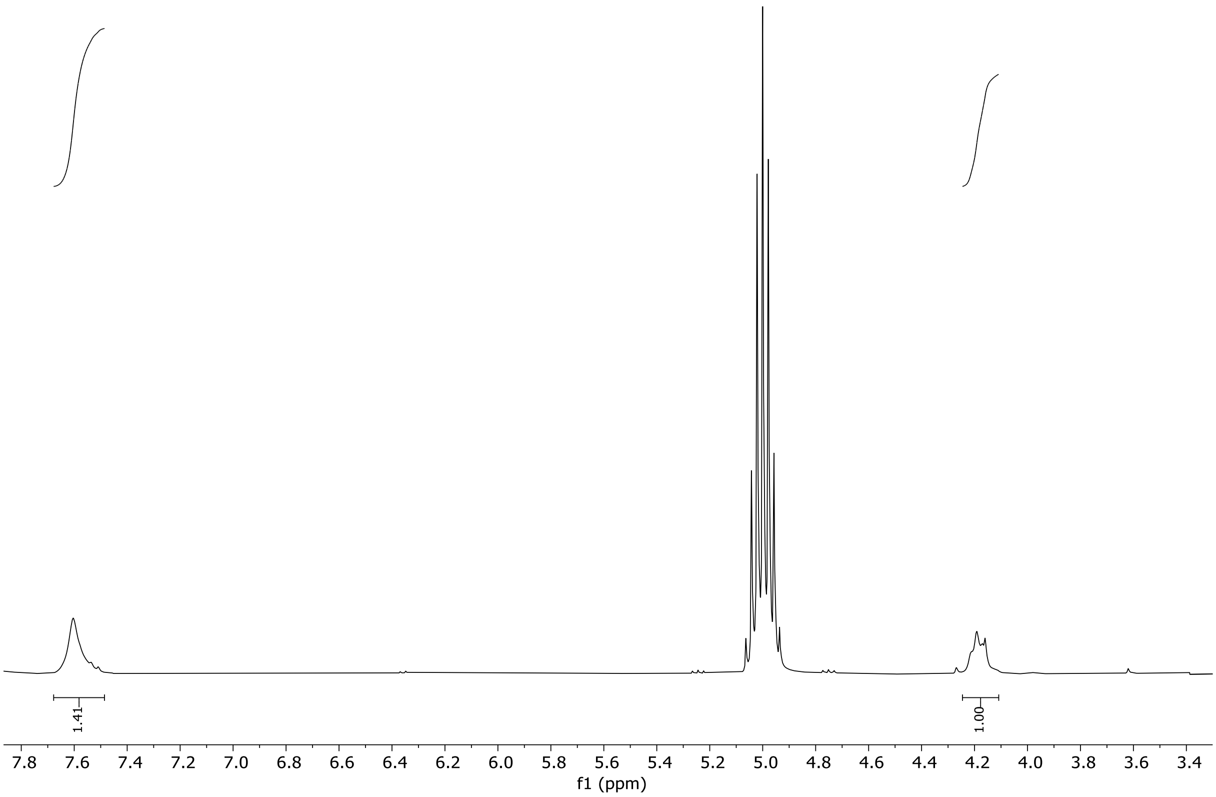

Supplementary Figure 55 H/D Exchange of 1a with 10 mol % $Pd(OAc)_2$

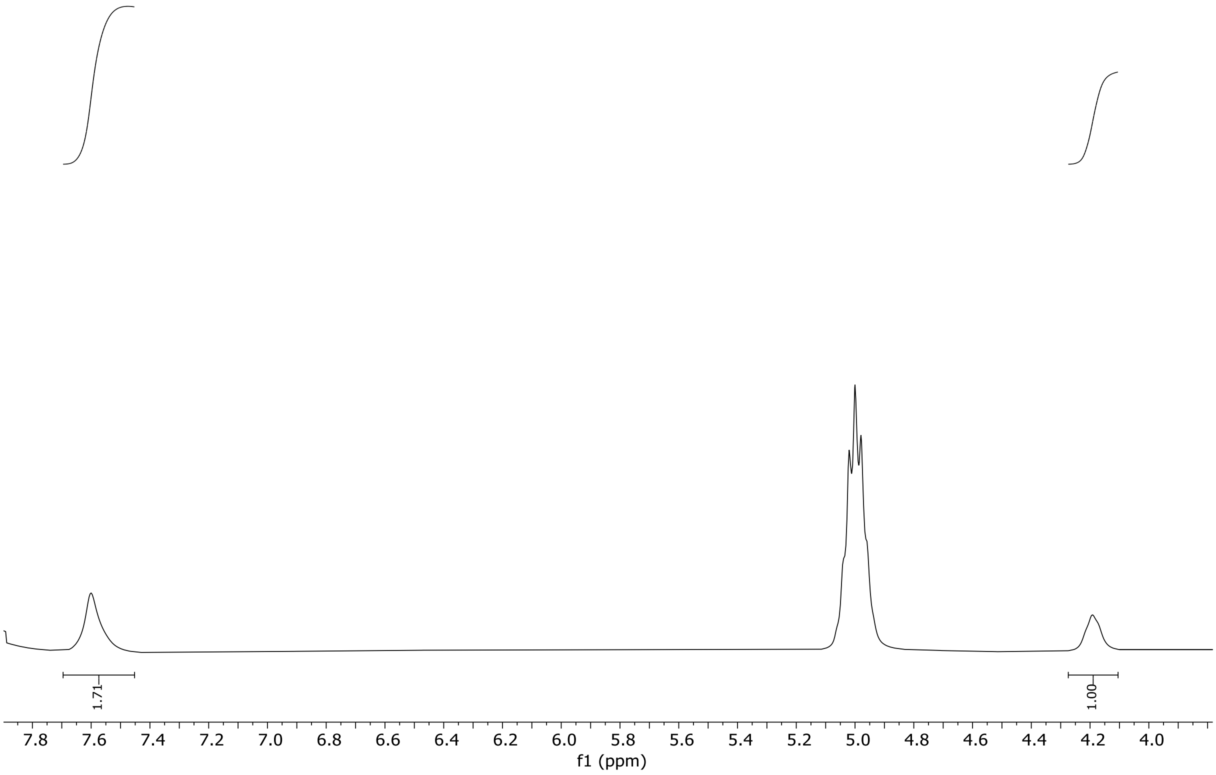

Supplementary Figure 56 H/D Exchange of 1a with 10 mol % $Cu(OTf)_2$

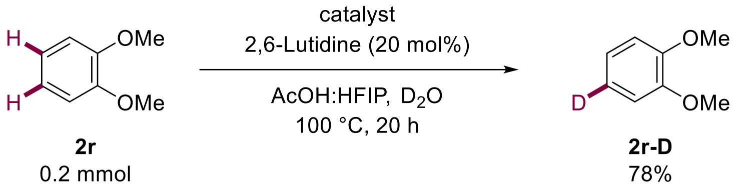

Reaction conditions: **2r** (0.20 mmol), $Pd(OAc)_2$ (10 mol %) or $Cu(OTf)_2$ (10 mol %), 2,6-lutidine (20 mol %, HFIP:AcOH (0.3 mL : 0.6 mL) were added to a 10 mL glass vial, 100 °C, 20 h. After the reaction, the reaction solution was filtered and transferred to NMR tube for crude H-NMR analysis.

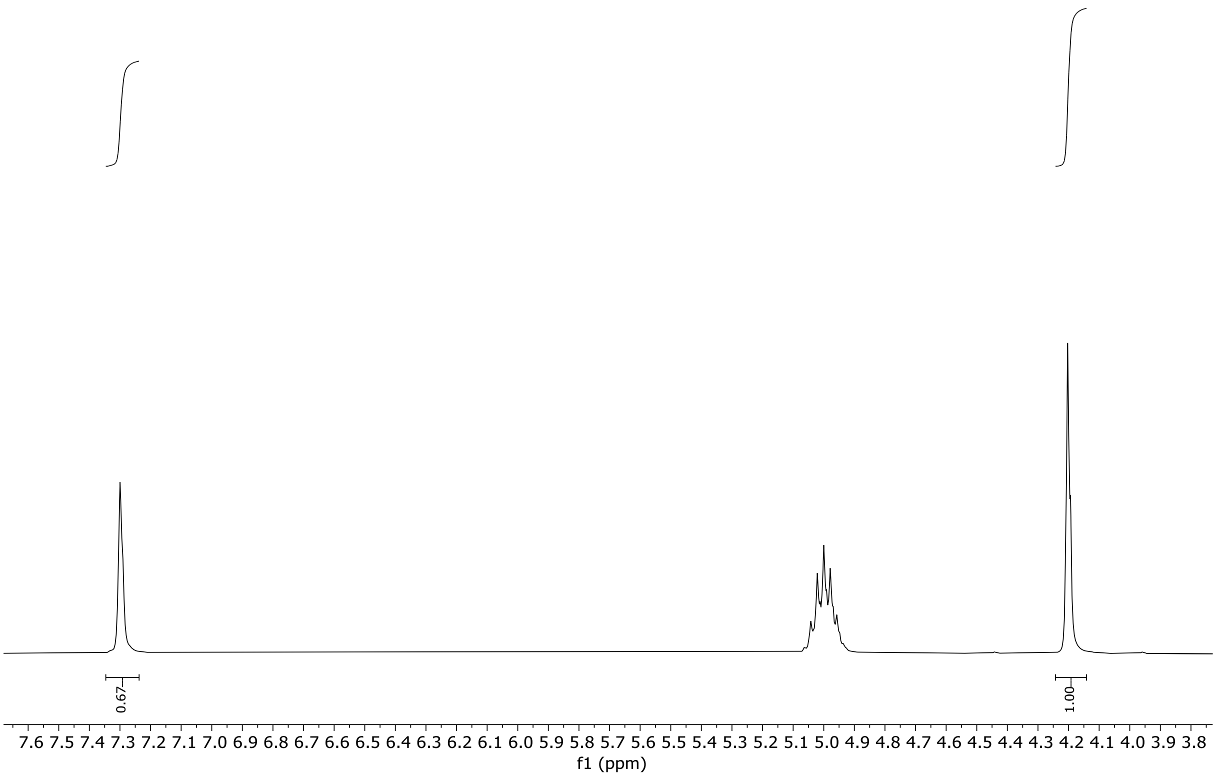

Supplementary Figure 57 H/D Exchange of 2r without Catalyst

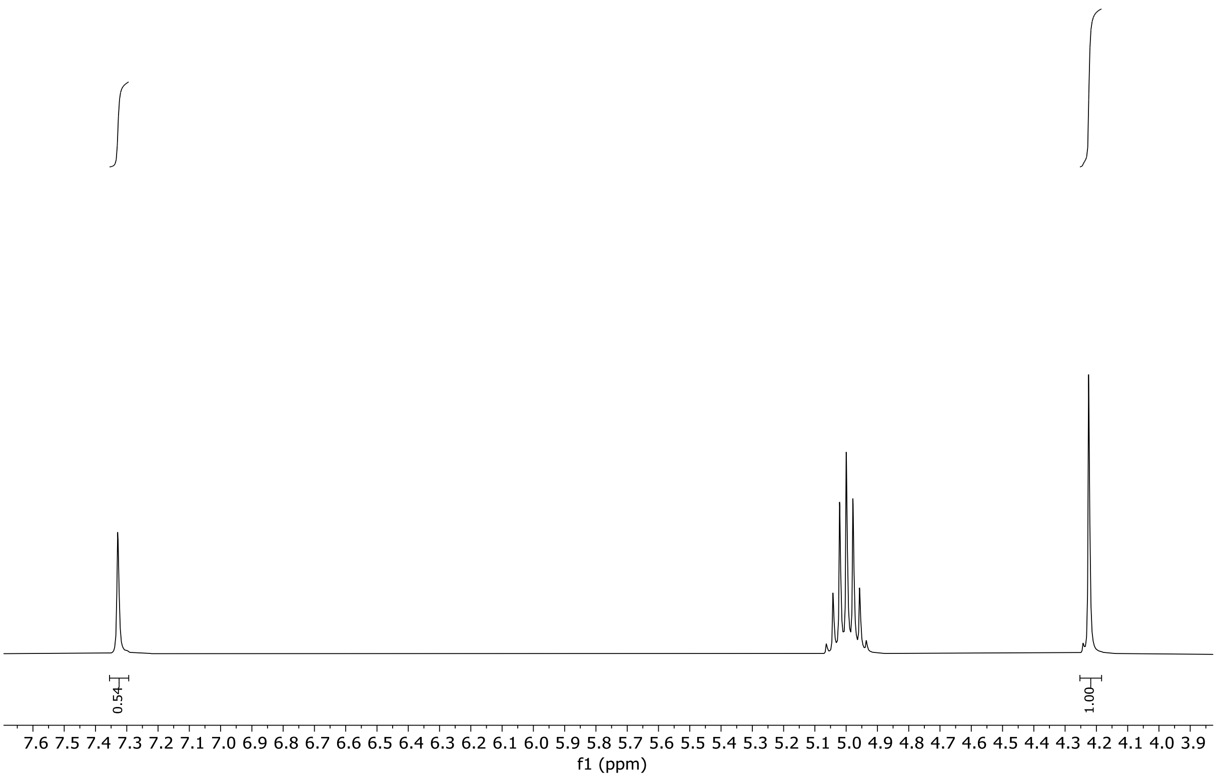

Supplementary Figure 58 H/D Exchange of 2r with 10 mol % Pd(OAc)$_2$

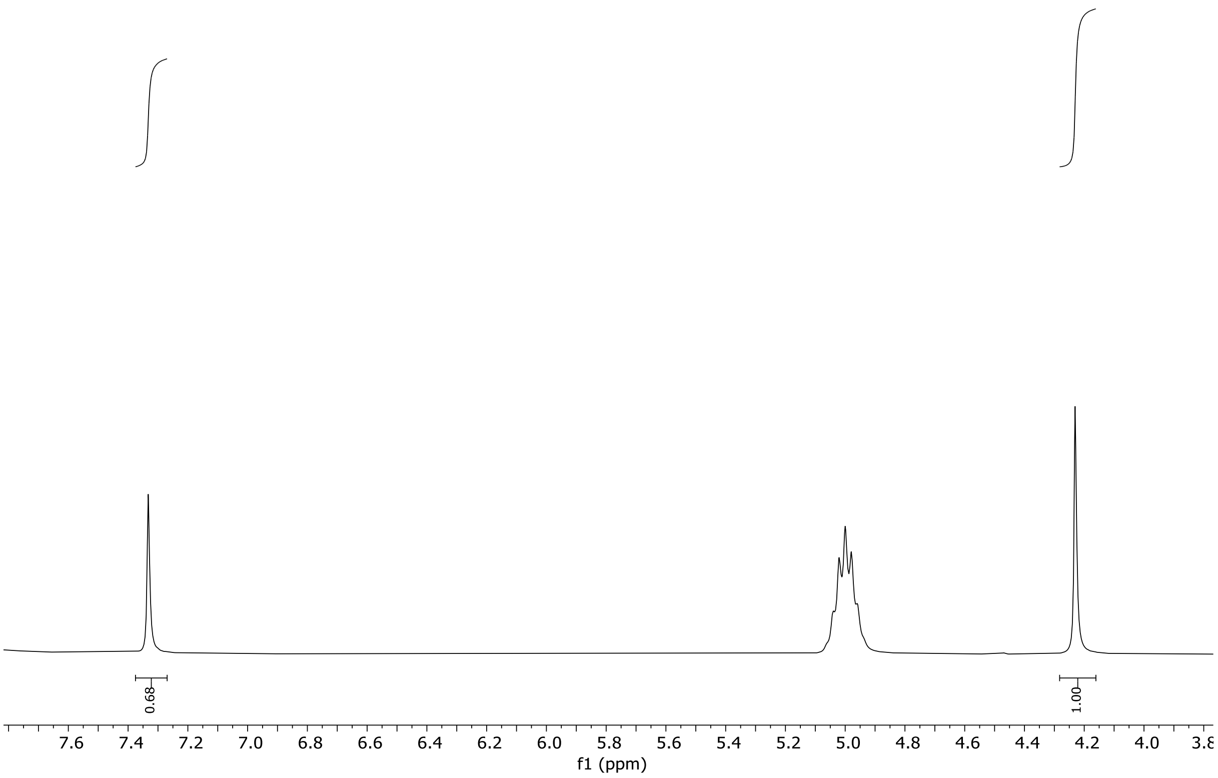

Supplementary Figure 59 H/D Exchange of 2r with 10 mol % Cu(OTf)$_2$

7.4 Kinetic Isotope Effect

7.4.1 KIE for **2a**

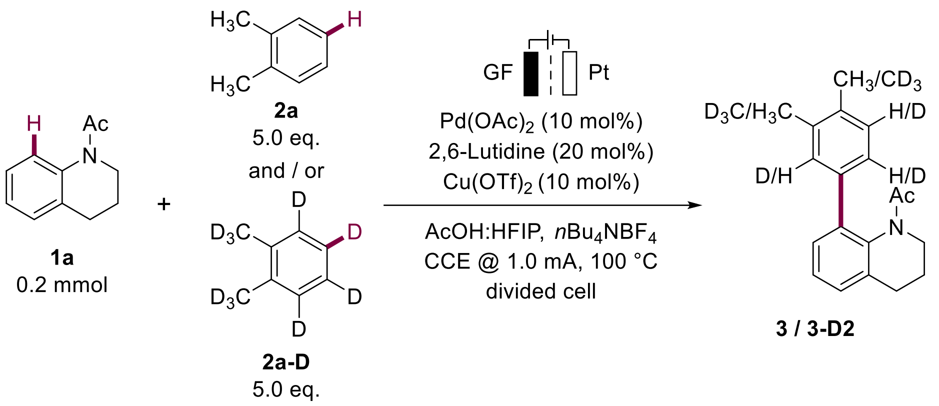

Reaction conditions: divided cell, anodic chamber: **1a** (0.20 mmol), **2a** (5.0 eq.) and/or **2a-D** (5.0 eq.), $Pd(OAc)_2$ (10 mol %), 2,6-lutidine (20 mol %), $Cu(OTf)_2$ (10 mol %), $n$$Bu_4NBF_4$ (200 mg), HFIP:AcOH (1.3 mL : 2.6 mL), cathodic chamber: **2a** (5.0 eq.) and/or **2a-D** (5.0 eq.), $n$$Bu_4NBF_4$ (200 mg), HFIP:AcOH (1.3 mL : 2.6 mL), 100 °C, electrolysis (CCE) at 1.0 mA, 20 h, graphite felt (GF) anode (10 mm × 15 mm × 2 mm), Pt-plate cathode (10 mm × 15 mm × 0.25 mm). For intermolecular competition experiment, KIE results were obtained from the H-NMR analysis for the isolated product. For initial rate analysis, the KIE results were based on the GC yield. At specific intervals shown on the horizontal axis, a small aliquot of the reaction mixture (0.1 mL) was taken, filtered through a small silica gel column, added with 0.1 mL internal standard stock solution (0.2 mmol dodecane in 5.5 mL EtOAc), and analyzed by GC. To ensure reliable results, the same reactor was utilized.

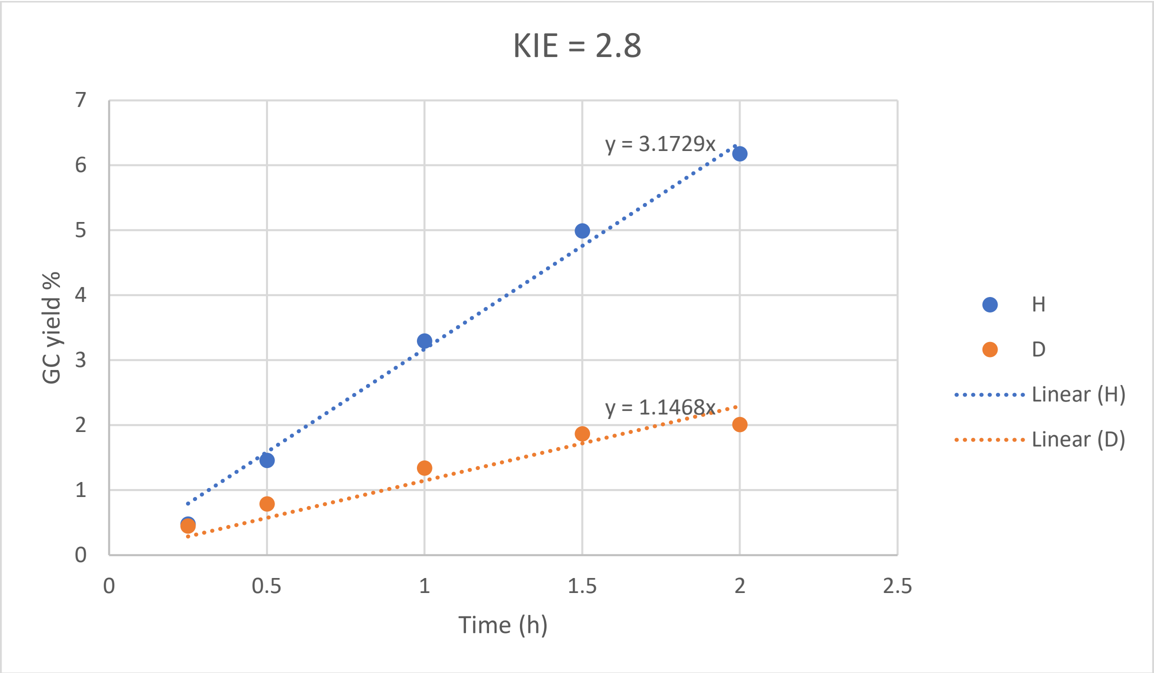

Supplementary Figure 60 Initial Rate KIE for 2a_KIE=2.8

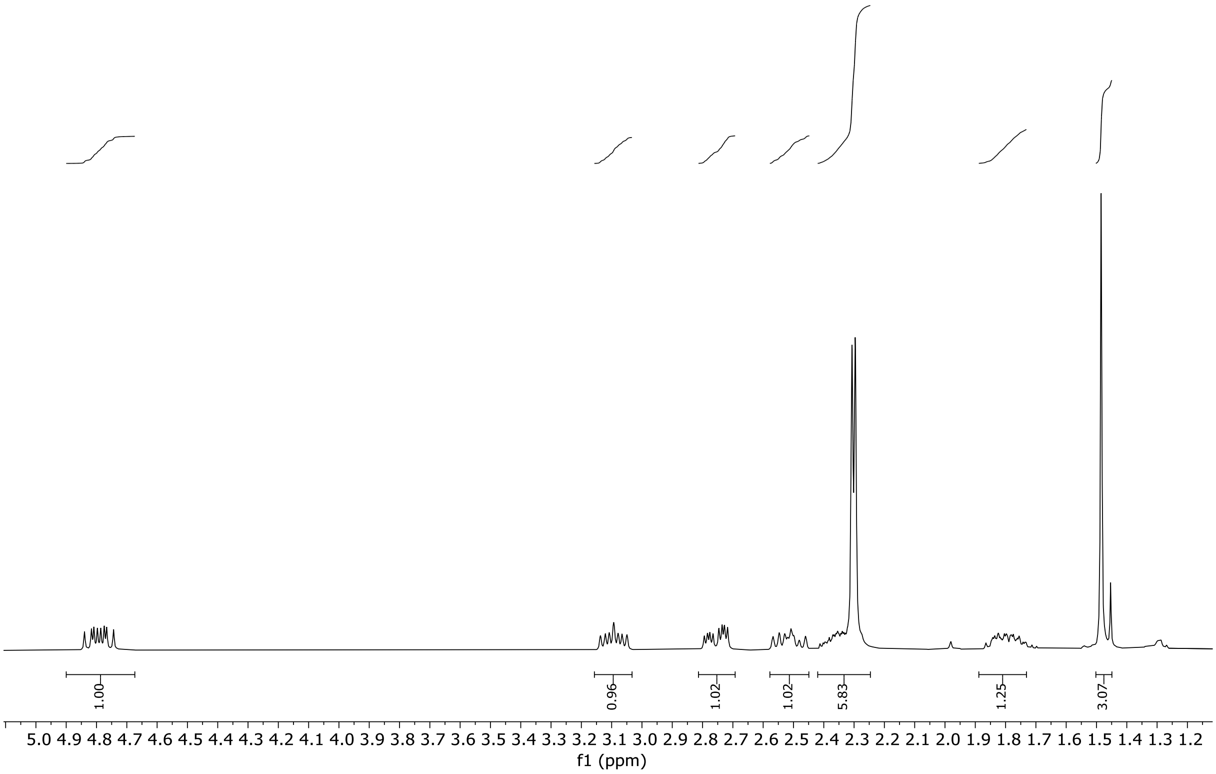

Supplementary Figure 61 Intermolecular KIE for 2a_KIE=3.5

7.4.2 KIE for **1a**

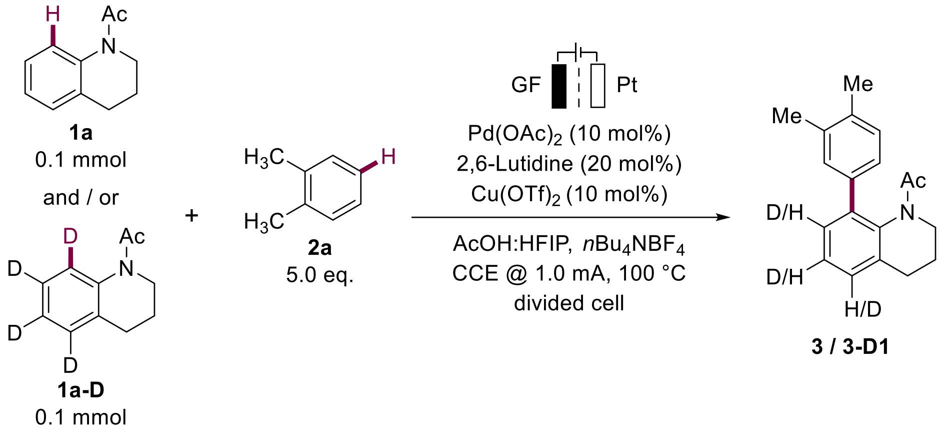

Reaction conditions: divided cell, anodic chamber: **1a** (0.20 mmol) or **1a-D** (0.20 mmol), or **1a** (0.10 mmol) and **1a-D** (0.10 mmol) for intermolecular experiment, **2a** (5.0 eq.), $Pd(OAc)_2$ (10 mol %), 2,6-lutidine (20 mol %), $Cu(OTf)_2$ (10 mol %), $n$$Bu_4NBF_4$ (200 mg), HFIP:AcOH (1.3 mL : 2.6 mL), cathodic chamber: **2a** (5.0 eq.), $n$$Bu_4NBF_4$ (200 mg), HFIP:AcOH (1.3 mL : 2.6 mL), 100 °C, electrolysis (CCE) at 1.0 mA, 4 h, graphite felt (GF) anode (10 mm × 15 mm × 2 mm), Pt-plate cathode (10 mm × 15 mm × 0.25 mm). For intermolecular competition experiment, KIE results were obtained from the H-NMR analysis for the isolated product. For initial rate analysis, the KIE results were based on the GC yield. At specific intervals shown on the horizontal axis, a small aliquot of the reaction mixture (0.1 mL) was taken, filtered through a small silica gel column, added with 0.1 mL internal standard stock solution (0.2 mmol dodecane in 5.5 mL EtOAc), and analyzed by GC. To ensure reliable results, the same reactor was utilized.

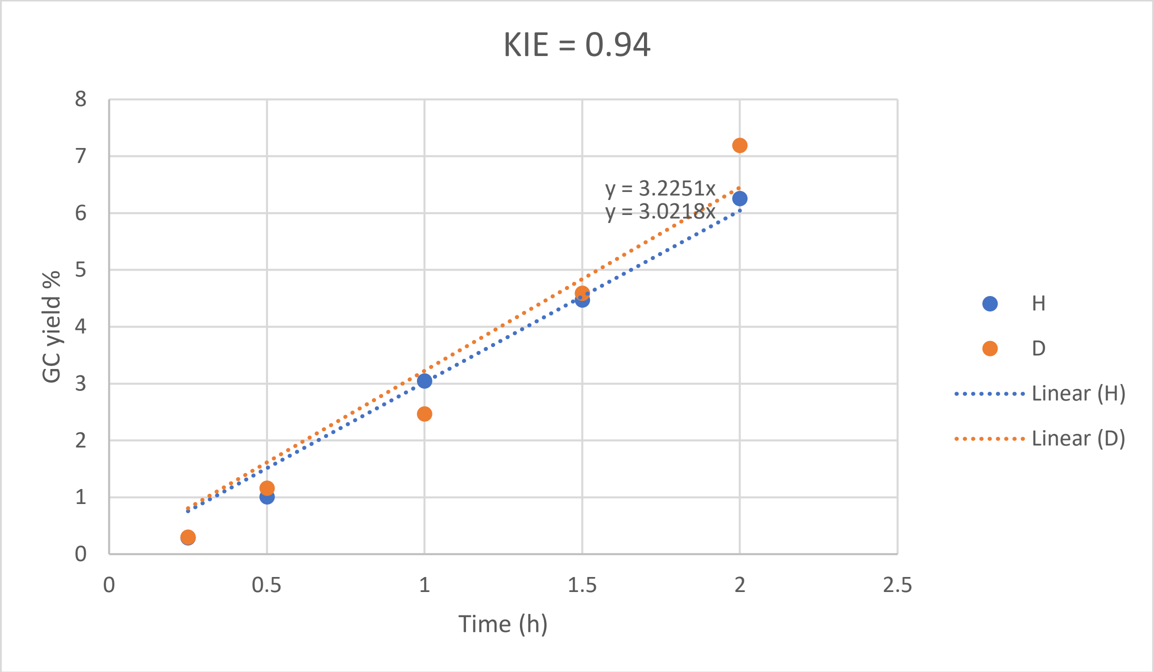

Supplementary Figure 62 Initial Rate KIE for 1a_KIE=0.94

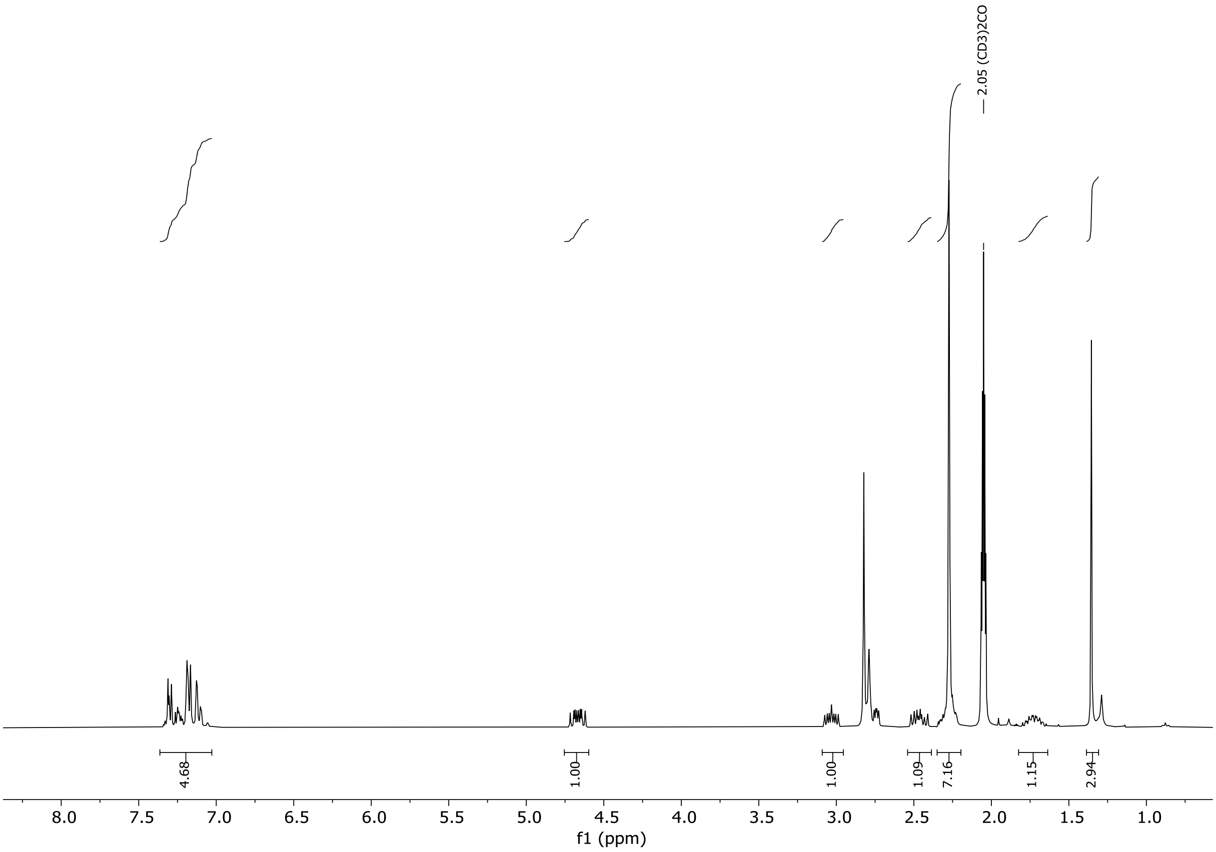

Supplementary Figure 63 Intermolecular KIE for 1a_KIE=1.24

7.5 Organopalladium Complex

7.5.1 Intermediates under Catalytic Conditions

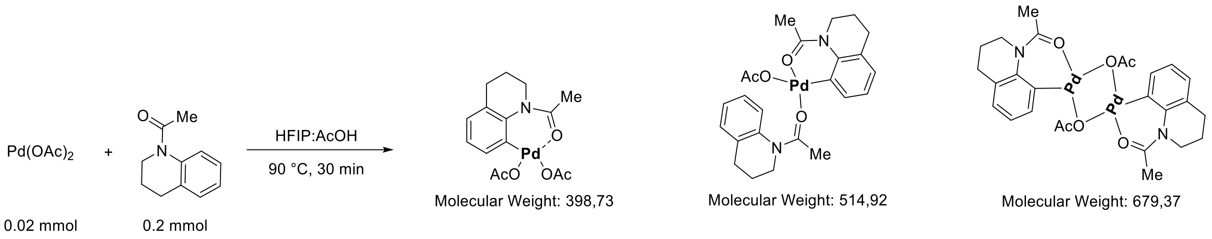

Reaction conditions: **1a** (0.2 mmol), $Pd(OAc)_2$ (10 mol %), HFIP:AcOH (1.3 mL : 2.6 mL) were added to a 10 mL glass vial at 90 °C for 30 min. After the reaction, the reaction solution was filtered and measured by HRMS.

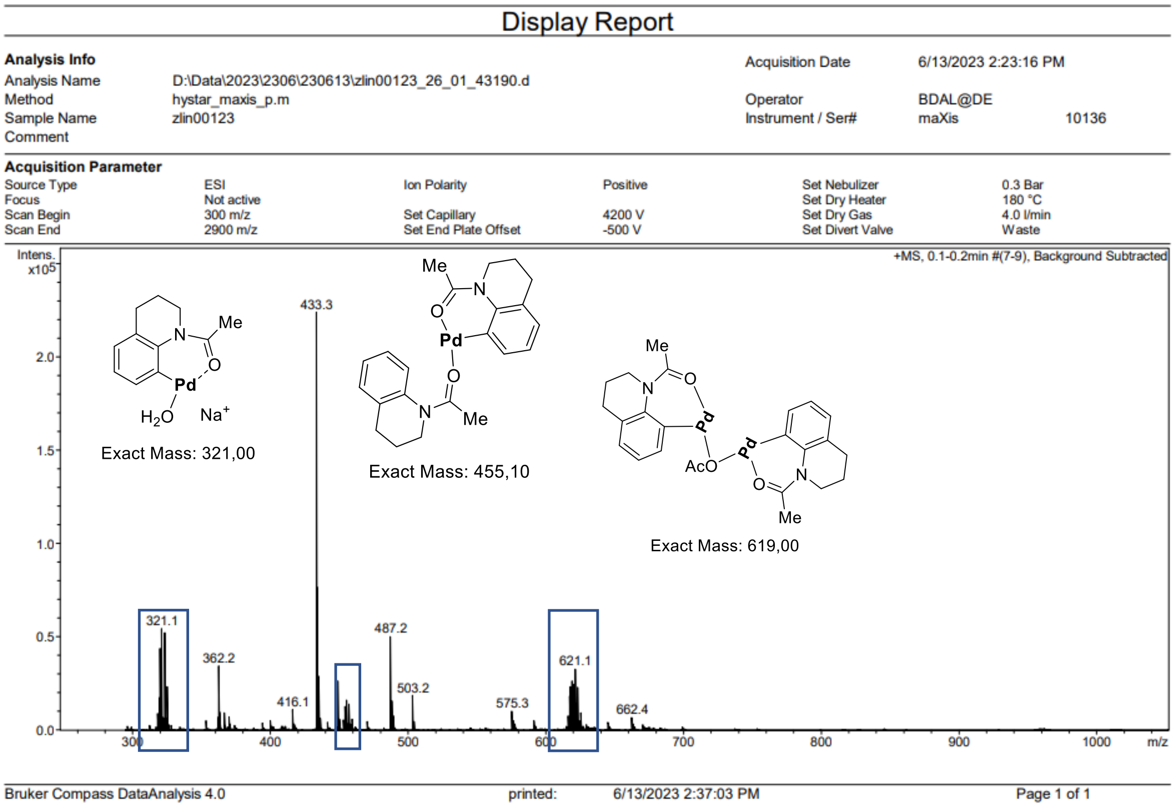

Supplementary Figure 64 Possible Intermediates under Catalytic Reaction Using 0.2 mmol 1a

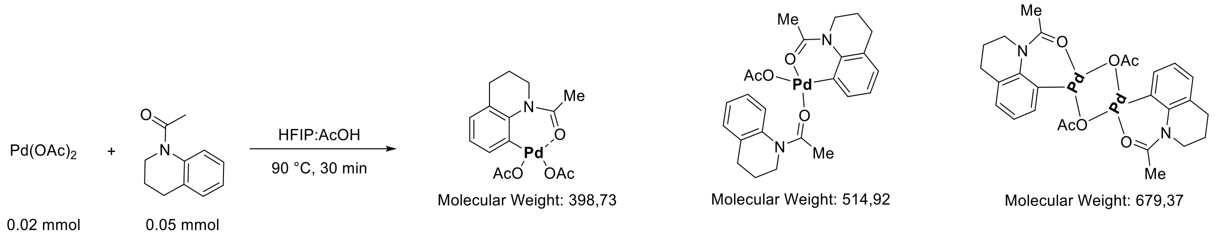

Reaction conditions: **1a** (0.05 mmol), Pd(OAc)$_2$ (10 mol %), HFIP:AcOH (1.3 mL : 2.6 mL) were added to a 10 mL glass vial at 90 °C for 30 min. After the reaction, the reaction solution was filtered and measured by HRMS.

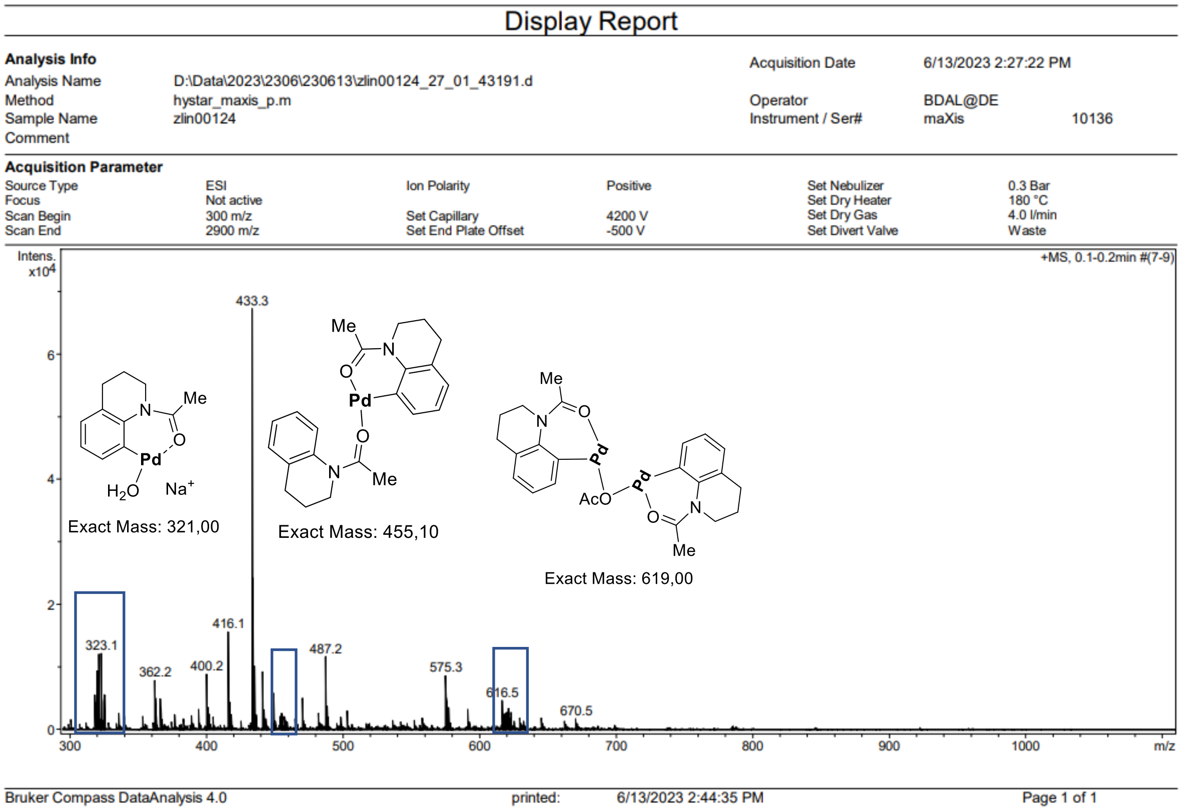

Supplementary Figure 65 Possible Intermediates under Catalytic Reaction Using 0.05 mmol 1a

7.5.2 Synthesis of Palladium Complex

The syntheses of complex **54** and **55** were carried out according to the procedure described in the literature (figure 3b in the manuscript).[4] To a 10 mL glass vial, substrate **1b** or **1f** (0.2 mmol, 1.0 equiv.), $Pd(OAc)_2$ (0.2 mmol, 1.0 equiv.), TFA (0.2 mmol, 1.0 equiv.) and 2 mL DCM were loaded and sealed with a cap. The reaction mixture was stirred at 40 °C for 12 h. After the reaction was completed, the precipitate was collected by filtration, washed with DCM (1 mL) and hexane (5 mL), and dried in *vacuo* to afford product **54** or **55**.

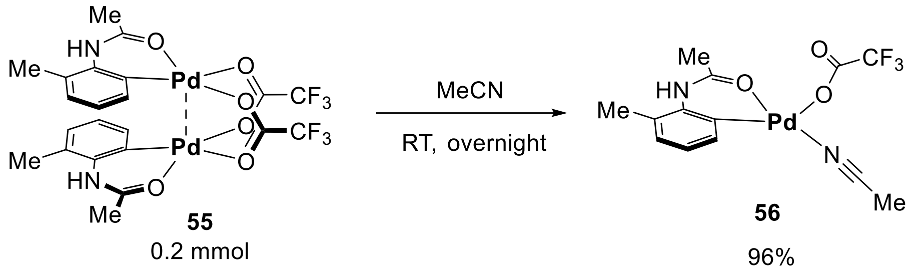

The synthesis of complex **56** was carried out according to the procedure described in the literature.[5] To a 10 mL glass vial, **55** (0.2 mmol) and 2 mL MeCN were loaded and sealed with a cap. The reaction mixture was stirred at room temperature overnight. After the reaction was completed, the solvent was removed in *vacuo* to afford product **56** in 96% yield.

7.5.3 Intermediates in Stoichiometric Organopalladium Reaction

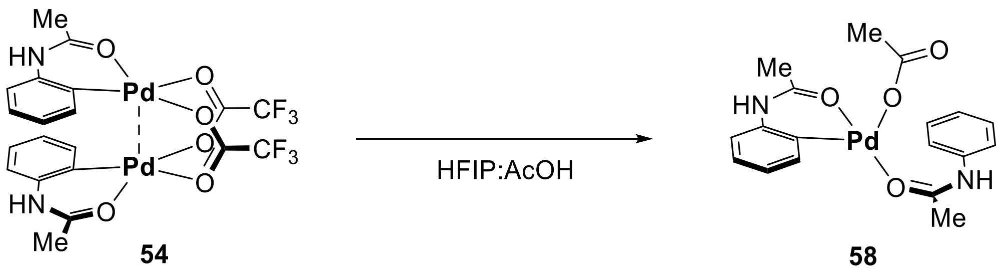

Reaction conditions: **54** (5.0 mg), without or with **1b** (0.2 mmol), HFIP:AcOH (0.3 mL : 0.6 mL) were added to a 10 mL glass vial at RT or 90 °C for 15 min. After the reaction, the reaction solution was analyzed by HRMS.

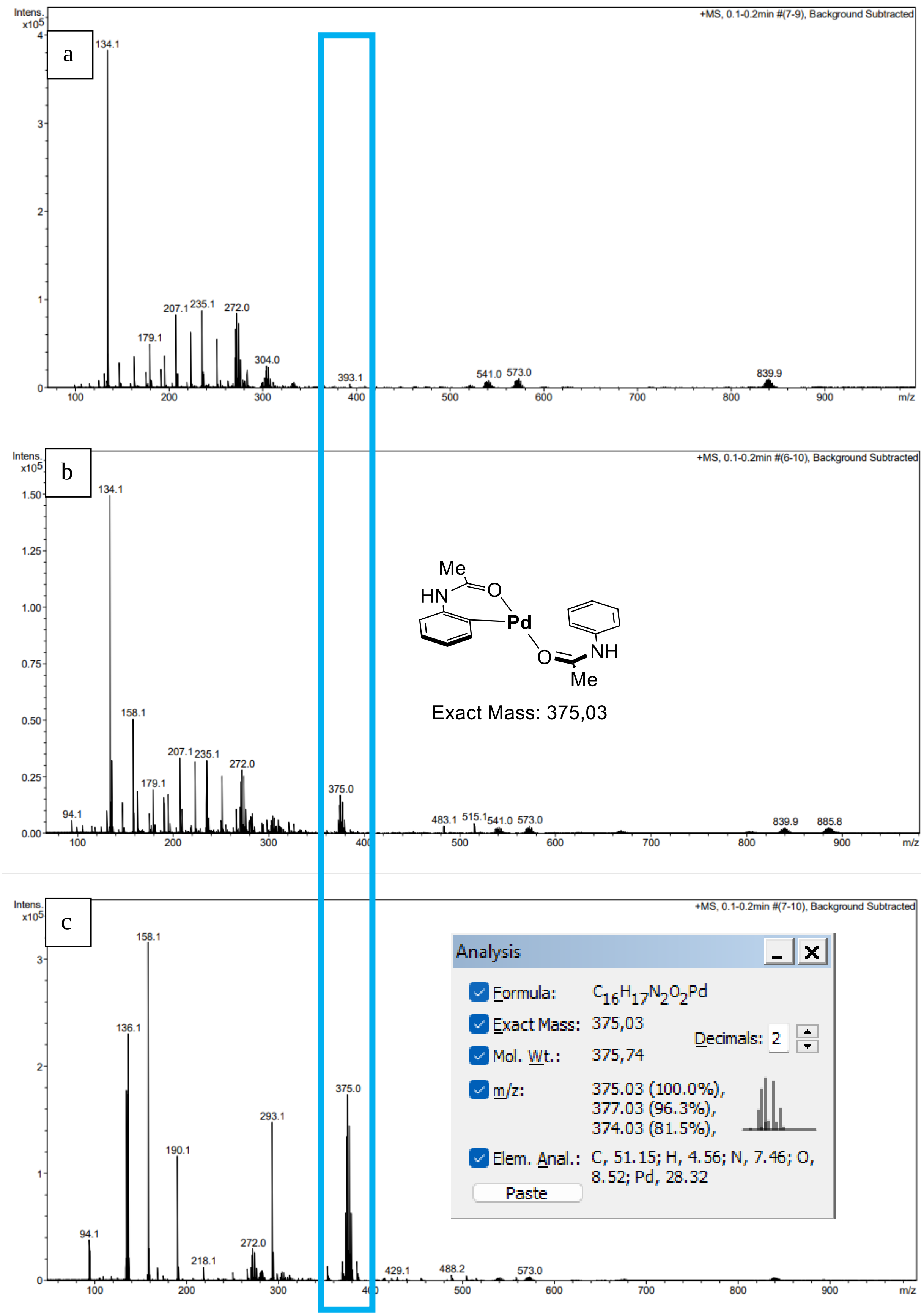

Supplementary Figure 66 Evolution of Complex 54. a) **54** at rt; b) **54** at 90 °C; c) **54** at 90 °C with 0.2 mmol **1b**.

7.5.4 Stoichiometric Organopalladium Reaction with Substrate **2a**

Reaction conditions: **55** (0.025 mmol), **2a** (0.5 mL), HFIP:AcOH (1.3 mL : 2.6 mL) were added to a 10 mL glass vial and heated to 100 °C. After the reaction, the solvent was removed in *vacuo* and the residue was directly purified by column chromatography on silica gel.

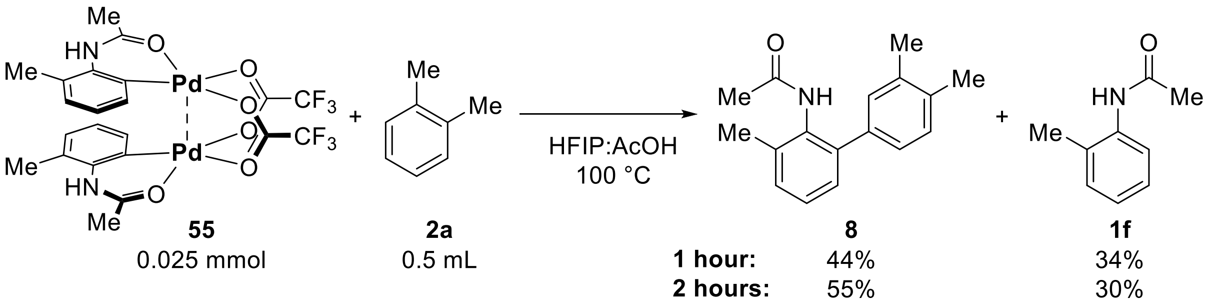

Supplementary Figure 67 Reaction of Complex 55

7.6 Cyclic Voltammograms

CV measurements were conducted with a Metrohm Autolab PGSTAT204 potentiostat and Nova 2.1 software. A glassy carbon (disk, diameter: 3 mm), a coiled platinum wire counter electrode and a saturated calomel (SCE) reference electrode were employed. The voltammograms were recorded at room temperature in HFIP:AcOH (1.3 mL:2.6 mL) with 0.1 M $n\text{Bu}_4\text{NBF}_4$ as supporting electrolyte under N_2 atomsphere. The scan rate is 100 mV/s. Deviations from the general experimental conditions are indicated in the respective figures and descriptions. $n\text{Bu}_4\text{NBF}_4$ was recrystallized from EtOAc and *n*-hexane before use.

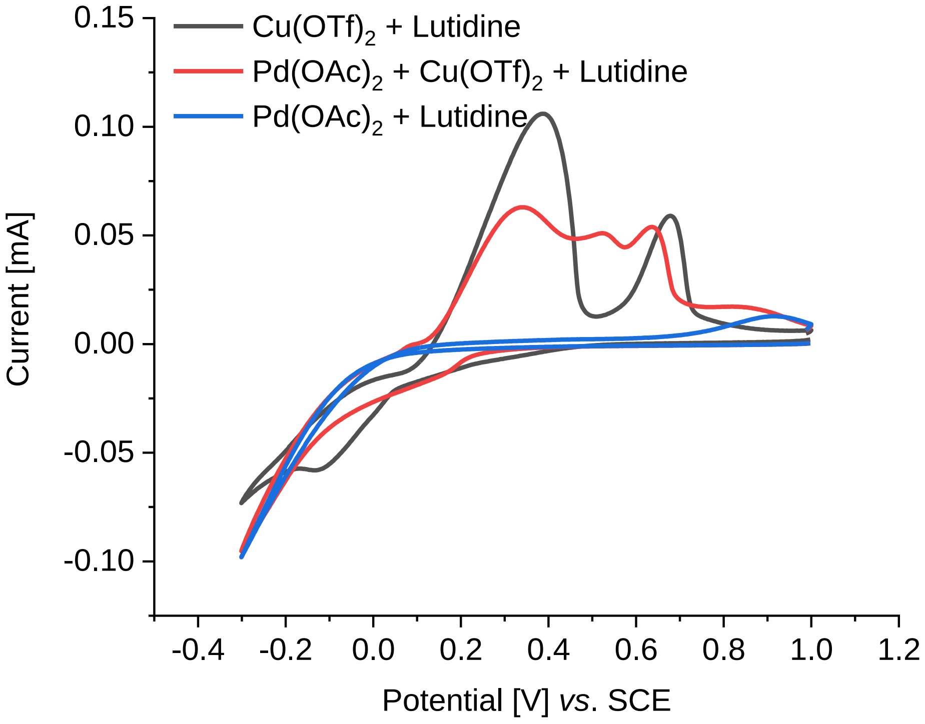

Supplementary Figure 68 CV Studies for the Role of $Pd(OAc)_2$, $Cu(OTf)_2$ and Lutidine. $Pd(OAc)_2$ (10 mM), $Cu(OTf)_2$ (10 mM) and Lutidine (20 mM).

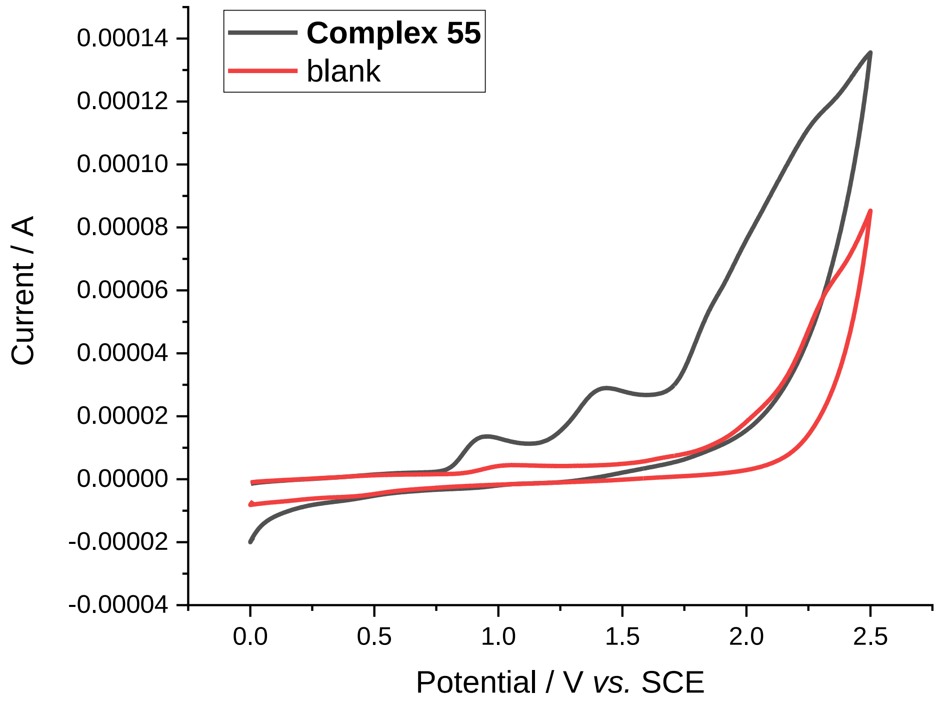

Supplementary Figure 69 CV Studies of Complex 55. 55 (2 mM).

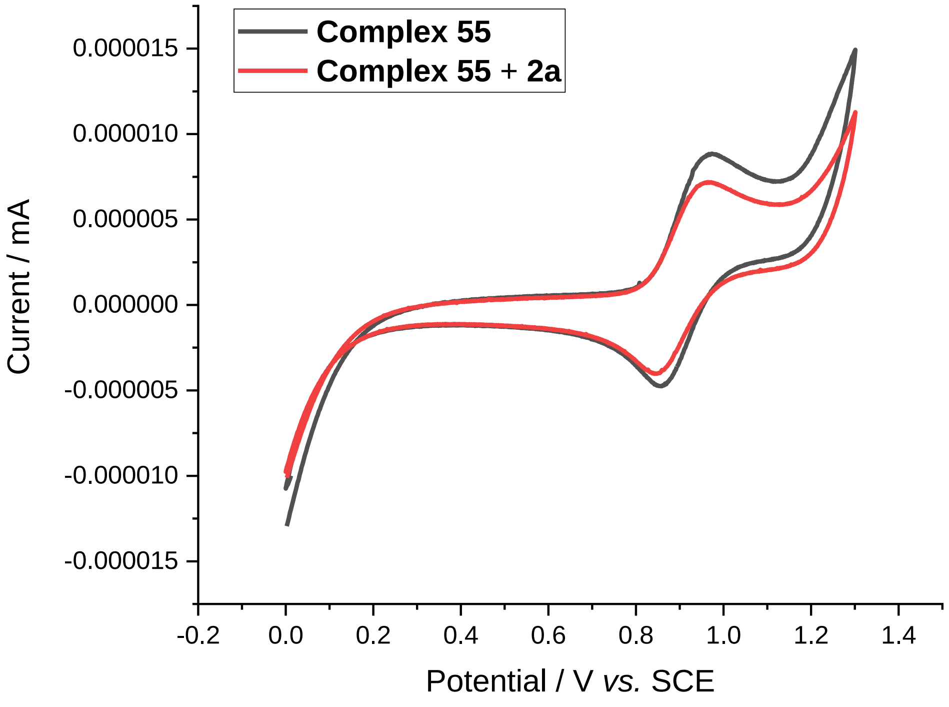

Supplementary Figure 70 CV Studies of Complex 55 and 2a. 55 (2 mM), **2a** (830 mM).

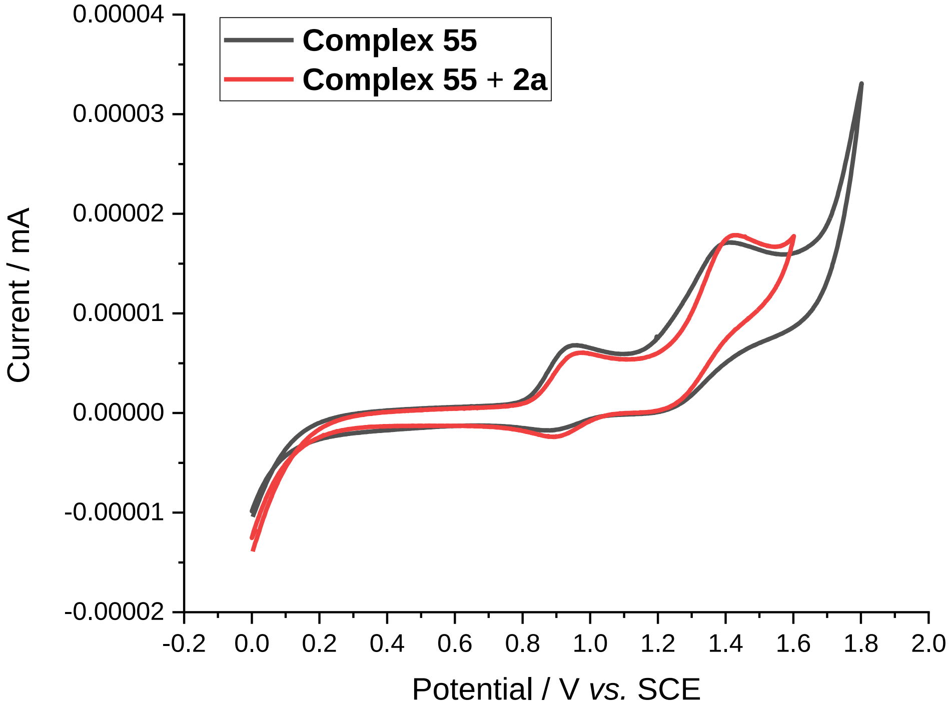

Supplementary Figure 71 CV Studies of Complex 55 and 2a with Larger Potential Window. **55** (2 mM), **2a** (830 mM).

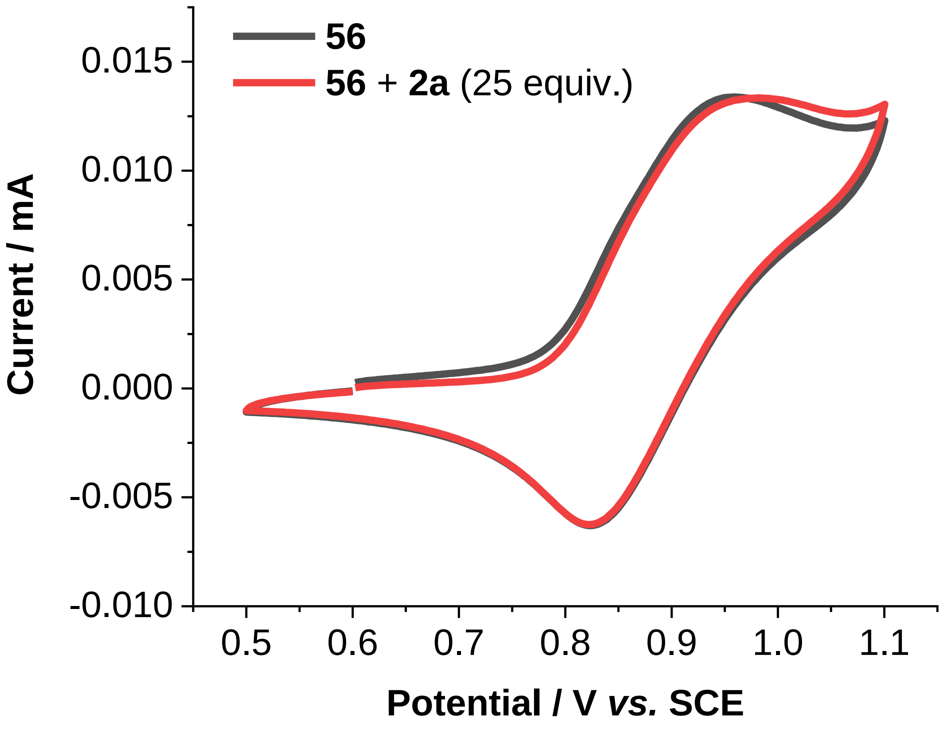

Supplementary Figure 72 CV Studies of Complex 56 and 2a. 56 (4 mM), **2a** (100 mM).

7.7 Organometallic Reaction Profile

7.7.1 Pre-experiments

Reaction conditions: 10 mL glass vial, **55** (0.05 mmol), **2a** (0.1–0.5 mL), HFIP:AcOH (5.0 mL, 1 : 2), 80-100 °C. At specific intervals shown on the horizontal axis, a small aliquot of the reaction mixture (0.05 mL) was taken, filtered through a small silica gel column, added with 0.05 mL internal standard stock solution (0.1 mmol dodecane in 5.5 mL EtOAc), and analyzed by GC. The GC yields were calibrated with a calibrating curve.

Reaction conditions: 10 mL glass vial, $Pd(OAc)_2$ (0.10 mmol), **1f** (0.10 mmol), **2a** (0.5 mL), HFIP:AcOH (5.0 mL, 1 : 2), 100 °C. At specific intervals shown on the horizontal axis, a small aliquot of the reaction mixture (0.05 mL) was taken, filtered through a small silica gel column, added with 0.05 mL internal standard stock solution (0.1 mmol dodecane in 5.5 mL EtOAc), and analyzed by GC. The GC yields were calibrated with a calibrating curve.

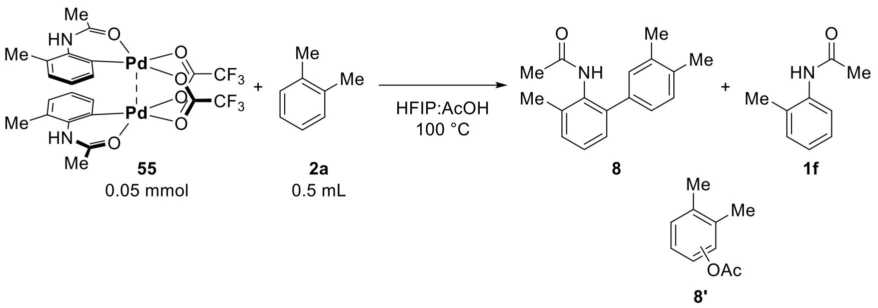

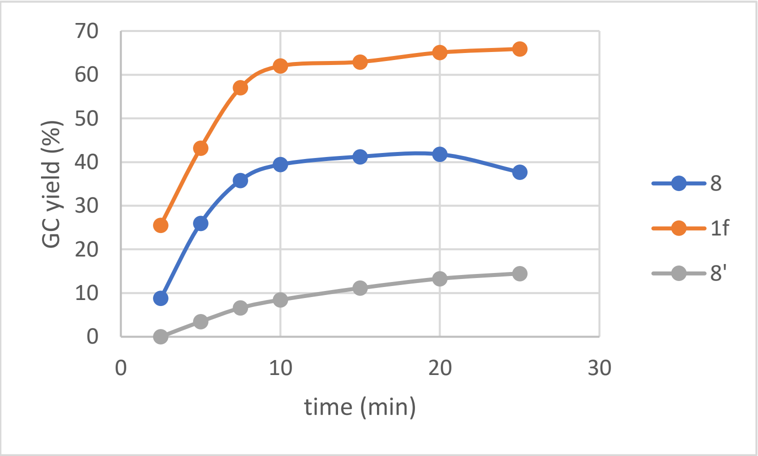

Supplementary Figure 73 Organometallic reaction of 0.05 mmol 55 at 100 °C. The profile was obtained from ex-situ GC analysis. Yields were calculated on amount of Pd.

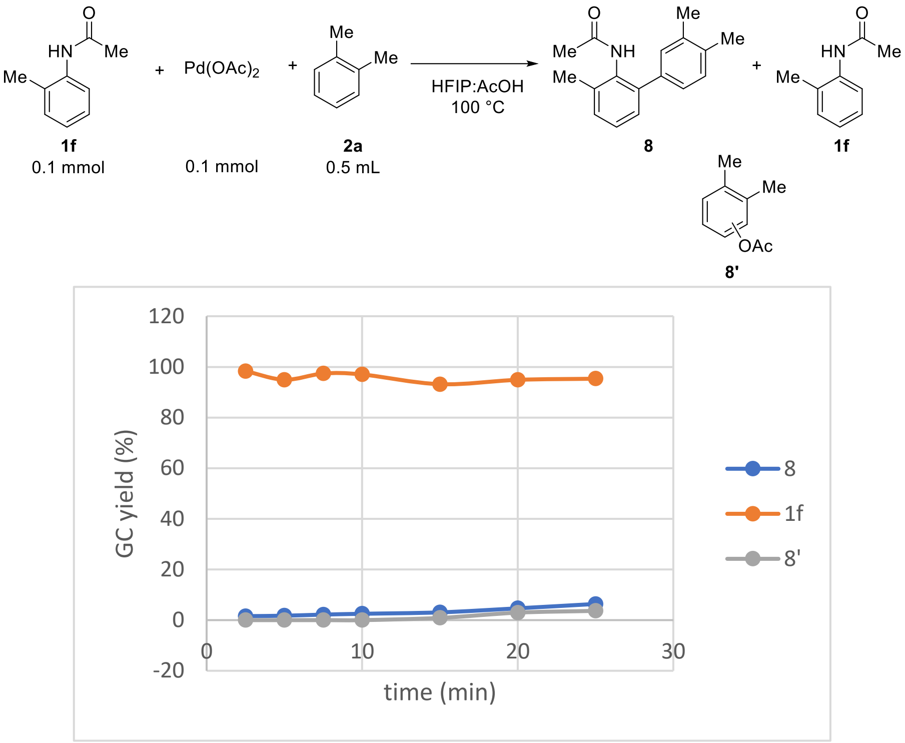

Supplementary Figure 74 Stoichiometric reaction using analogous concentration of Pd and 1f to the organometallic reaction in Supplementary Figure 72. The profile was obtained from ex-situ GC analysis. Yields were calculated on amount of Pd.

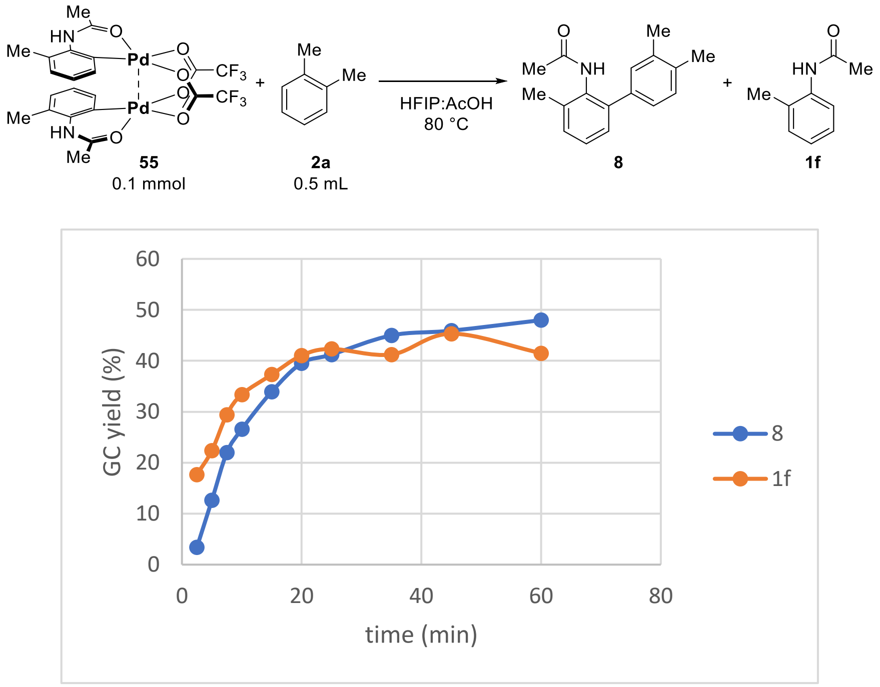

Supplementary Figure 75 Organometallic reaction of 0.05 mmol 55 at 80 °C. The profile was obtained from ex-situ GC analysis. Yields were calculated on amount of Pd.

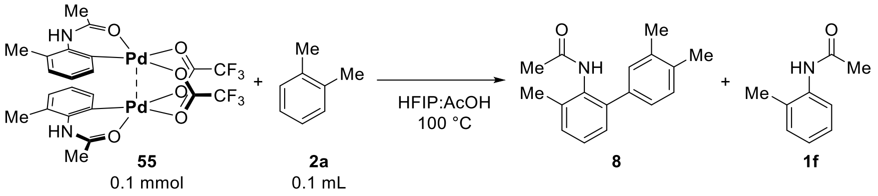

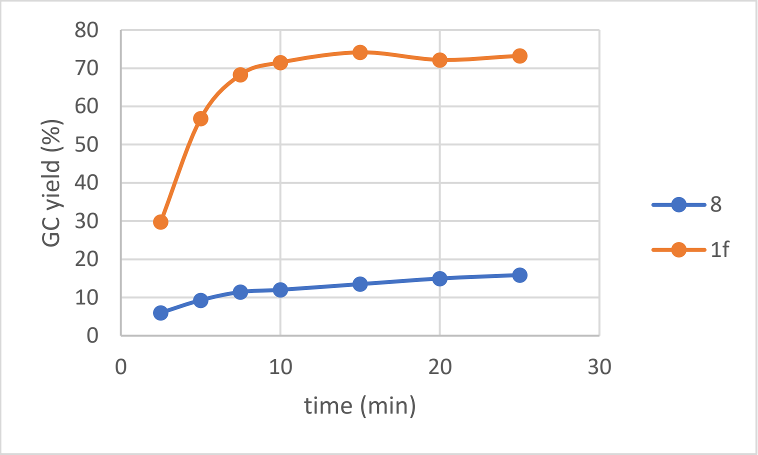

Supplementary Figure 76 Organometallic reaction of 0.05 mmol 55 with 0.1 ml 2a at 100 °C. The profile was obtained from ex-situ GC analysis. Yields were calculated on amount of Pd.

We did several pre-experiments using ex-situ GC analysis for monitoring the process of organometallic reaction of **55**. The rate of the organometallic reaction using stoichiometric amount of **55** is faster than that with the same concentration of $Pd(OAc)_2$ and **1a** (Supplementary Figure 73 & 74), indicating that **55** might be the on-cycle species or the pre-catalyst. Changing the temperature or the stoichiometric amount of **2a** influenced the rate of formation of **8**. Strikingly, we found the yield of **1a** was larger than that of **8** in first 2.5 minutes and the rate of the formation of **8** in the next few minutes seems faster (Supplementary Figure 73, 75 & 76). However, it is difficult to conclude the involvement of an induction period in the first 2–3 minutes for lacking of temperature information.

7.7.2 In-situ NMR Analysis

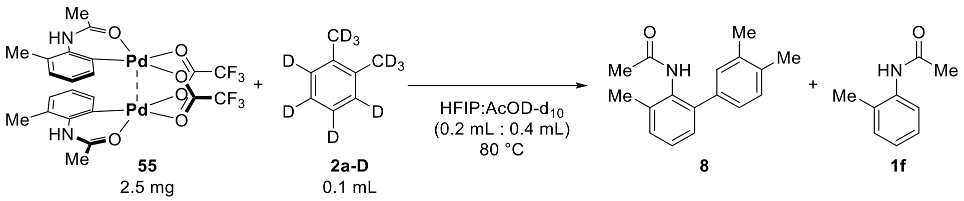

Supplementary Figure 77 Reaction Scheme for In-situ NMR Studies

Based on the pre-experiments, we further designed the VT-NMR studies in HFIP : AcOD-d4 : *o*-xylene-d10 (0.4 mL : 0.2 mL : 0.1 mL) at 80 °C. The amount of dimeric palladium species **55** used in the reaction is 2.5 mg. The use of *o*-xylene-d10 instead of *o*-xylene could enlarge the induction period for the convenience of detection. To reduce the dead time of the monitoring, we prepared a reference NMR sample with the same NMR tube, the same filling volume and the same solvent for the shimming. After the shimming, the reference sample was quickly replaced with the experimental sample. Despite these measures, it takes 3 minutes to get a good shimming. The inferior shimming in the first 3 minutes could be explained by the too rapid temperature variation, which was recorded in Supplementary Figure 78. Peaks with good resolutions were observed on the 3 minutes' spectrum, indicating that the reaction temperature has reach 79 °C homogeneously before 3 minutes. At 5 minutes, the reaction temperature was heated to 80 °C in a steady manner.

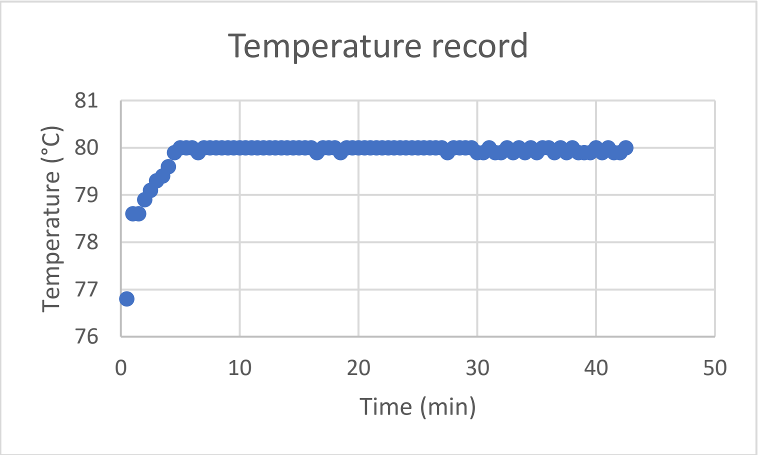

Supplementary Figure 78 Temperature record of the in-situ NMR measurement

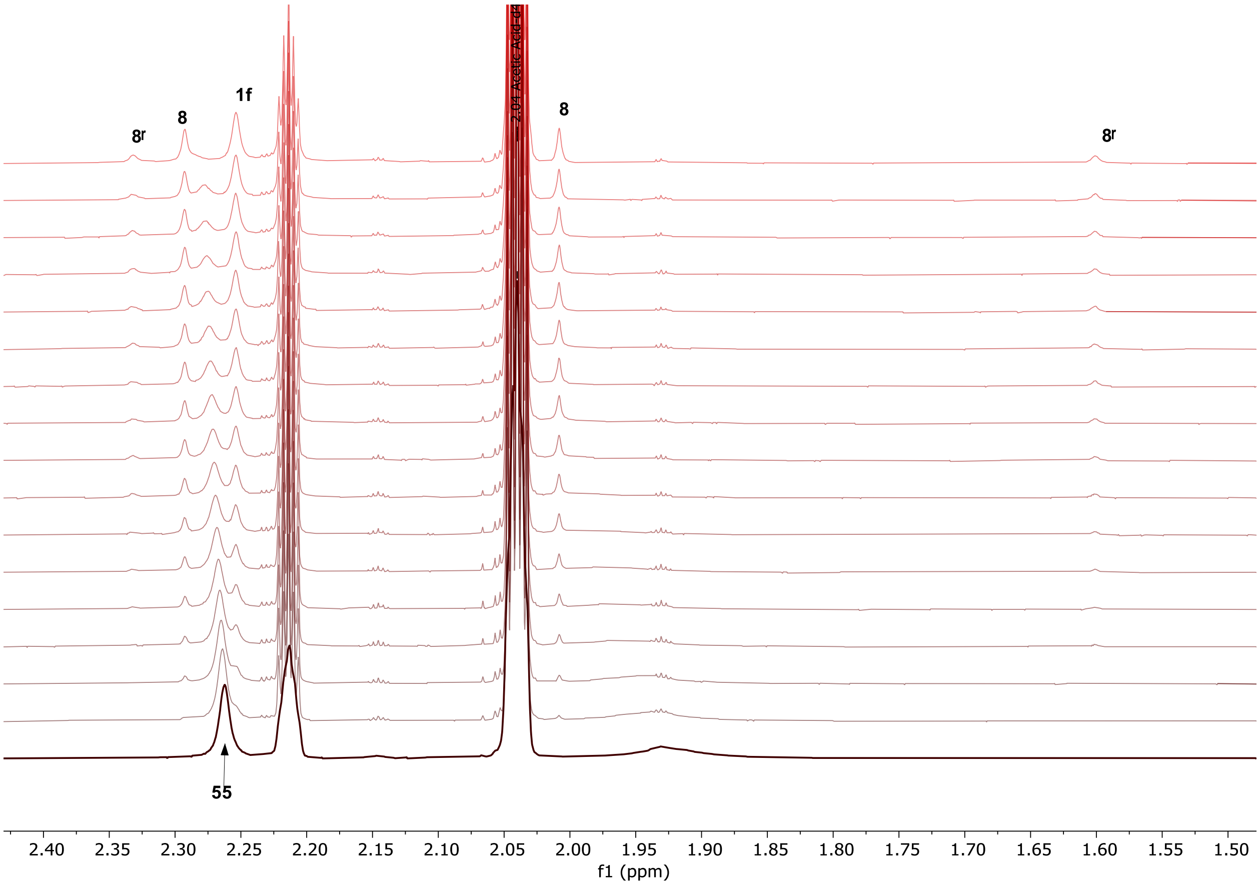

Supplementary Figure 79 ^{1}H-NMR spectrum 1 obtained from In-situ NMR detection at 80 °C. 600 MHz. The shown spectrum started from 2.5 min with an interval of 2.5 min (from bottom to top).

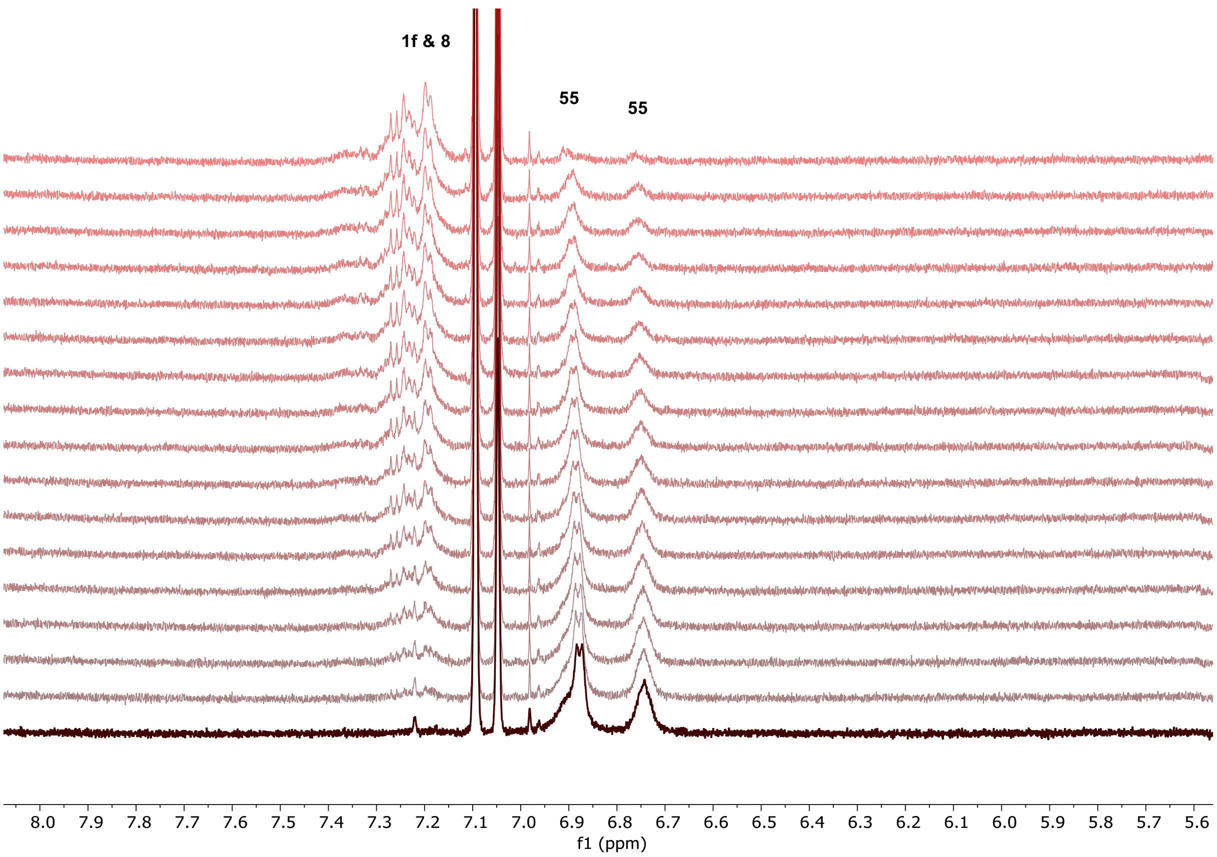

Supplementary Figure 80 **^{1}H-NMR spectrum 1 obtained from In-situ NMR detection at 80 °C. 600 MHz.** The shown spectrum started from 2.5 min with an interval of 2.5 min (from bottom to top).

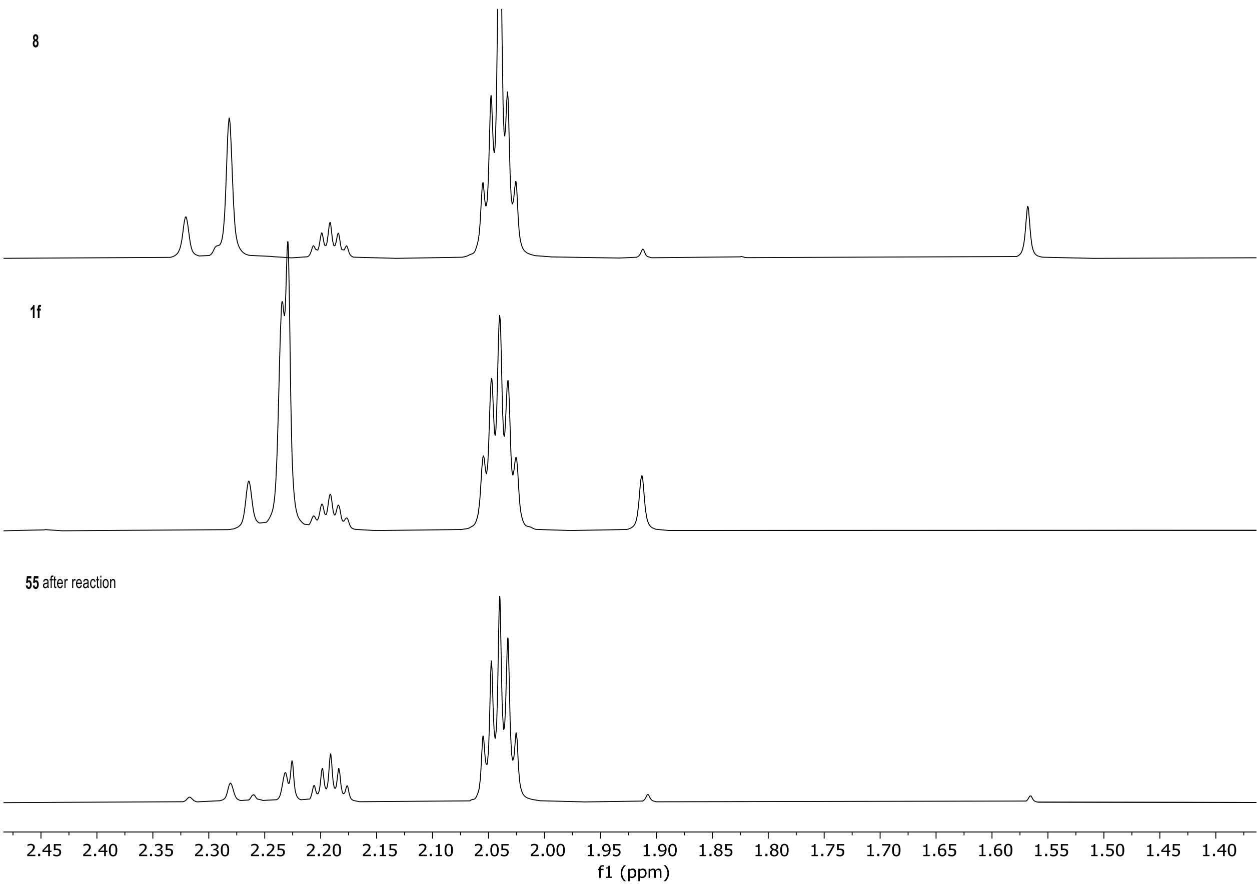

Supplementary Figure 81 NMR spectrum used as reference. Room temperature, 300 MHz.

Supplementary Figure 79–81 exhibited the chemical shifts of the key compounds, which could be used as references.

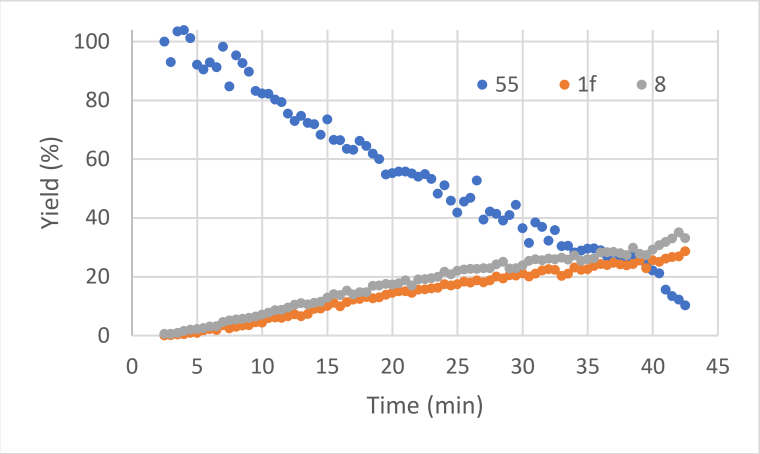

Supplementary Figure 82 Organometallic reaction profile from In-situ NMR analysis at 80 °C.

In the reaction profile monitored by NMR at 80 °C (Supplementary Figure 82), a relative method was used to quantified the compounds. The concentration of complex **55** decreased monotonically, indicating that it was converted to the desired product or the reaction intermediates. The yield of **1f** should be underestimated due to excessive background subtraction, leading to an overall reaction mass balance of 70%. Notably, an induction period was observed on the profile of **8**, suggesting that complex **55** is not the on-cycle species.

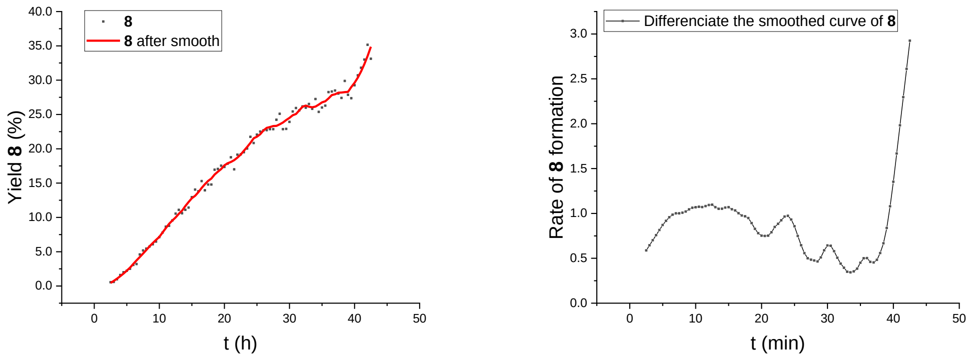

Supplementary Figure 83 Data processing for curve 8. a) Smooth the curve of **8**. b) The rate of **8** formation derived from the differentiation of curve **8**.

We smoothed and differentiated the curve of **8**, thus obtaining a rate profile of **8** formation (Supplementary Figure 83). If the intermediates **55** is the on-cycle species, the reaction rate should decrease monotonically as **55** did. However, the variation of the reaction rate exhibited a much more complicated pattern, which undergoes sequential rising, steading, descending and rising, indicating a more sophisticated mechanism. The rising at the end of the reaction monitoring might attributed to the unknown interference outside the reaction system. Therefore, our proposal of transmetalation is more plausible according to these results.

7.7.3 Ex-situ GC Measurements

Reaction conditions: 10 mL glass vial, **55** (0.05 mmol) or **56** (0.10 mmol), **2d** (0.5 mL), HFIP:AcOH (5.0 mL, 1 : 2), 80 °C. At specific intervals shown on the horizontal axis, a small aliquot of the reaction mixture (0.05 mL) was taken, filtered through a small silica gel column, added with 0.05 mL internal standard stock solution (0.1 mmol dodecane in 5.5 mL EtOAc), and analyzed by GC. The GC yields were calibrated with a calibrating curve. To obtain a reliable result, data was generated by using the same reactor, heating plate, and GC. The reactions of **55** and **56** and the respective samplings were performed at the same time.

7.8 Computational Studies

All calculation were performed using Gaussian 16, Revision A.03 package.[6] All structures were optimized at the B3LYP[7] level of theory in combination with D3 dispersion corrections with Becke-Johnson damping scheme (D3(BJ)).[8] All atoms were described with the 6-31G(d,p) basis set, whereas palladium was described with a LANL2DZ basis set with a Los Alamos effective core potential.[9] Analytical frequencies were carried out at the same level of theory in order to identify each stationary point as either an intermediate or a transition. These also provided thermal and non-thermal corrections to the Gibbs free energy at 373.15 K. The electronic energy was then refined through B3LYP[7] single-point calculations on the optimized geometries in combination with a standalone version of Grimme’s most recent dispersion correction D4,[10] with a 6-311+G(2d,p) basis set for all atom, expect for palladium, which was described with a SSD basis set and corresponding pseudo-potential.[11] Solvent effects for acetic acid of HFIP were included implicitly through the use of the SMD model.[12] The parameters for acetic acid were used as implemented in the Gaussian 16, whereas for HFIP the parameters were taken from Liu, Engle and coworkers.[13] Energies reported are based on gas-phase Gibbs free energies with 6-31G(d,p)-LANL2DZ basis set for which the electronic energies were refined to B3LYP-D4 with 6-311+G(2d,p)-SDD basis set and solvent effects.

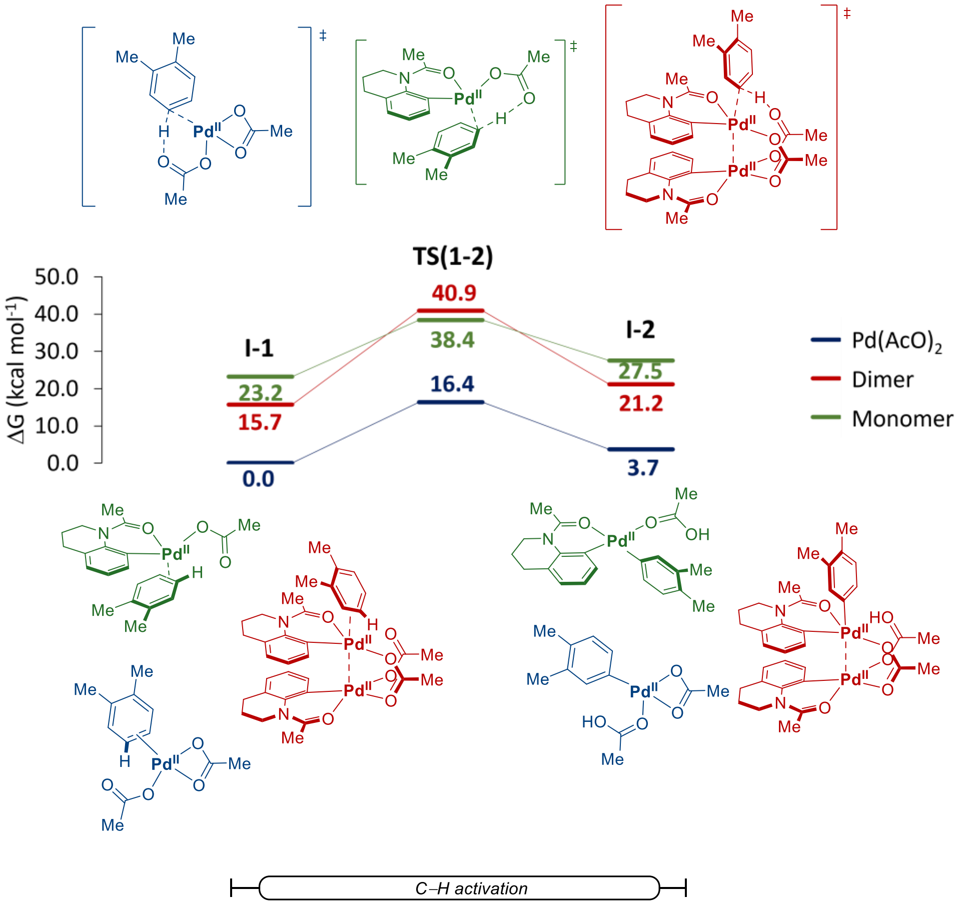

Supplementary Figure 84 **Computed relative Gibbs free energies ($\Delta G_{373.15}$) in kcal mol^{-1} for three possible pathways for the C–H activation elementary step at the B3LYP-D4/6-311+G(2d,p)-SDD+SMD(AcOH)/B3LYP-D3(BJ)/6-31G(d,p)-LANL2DZ level of theory.**

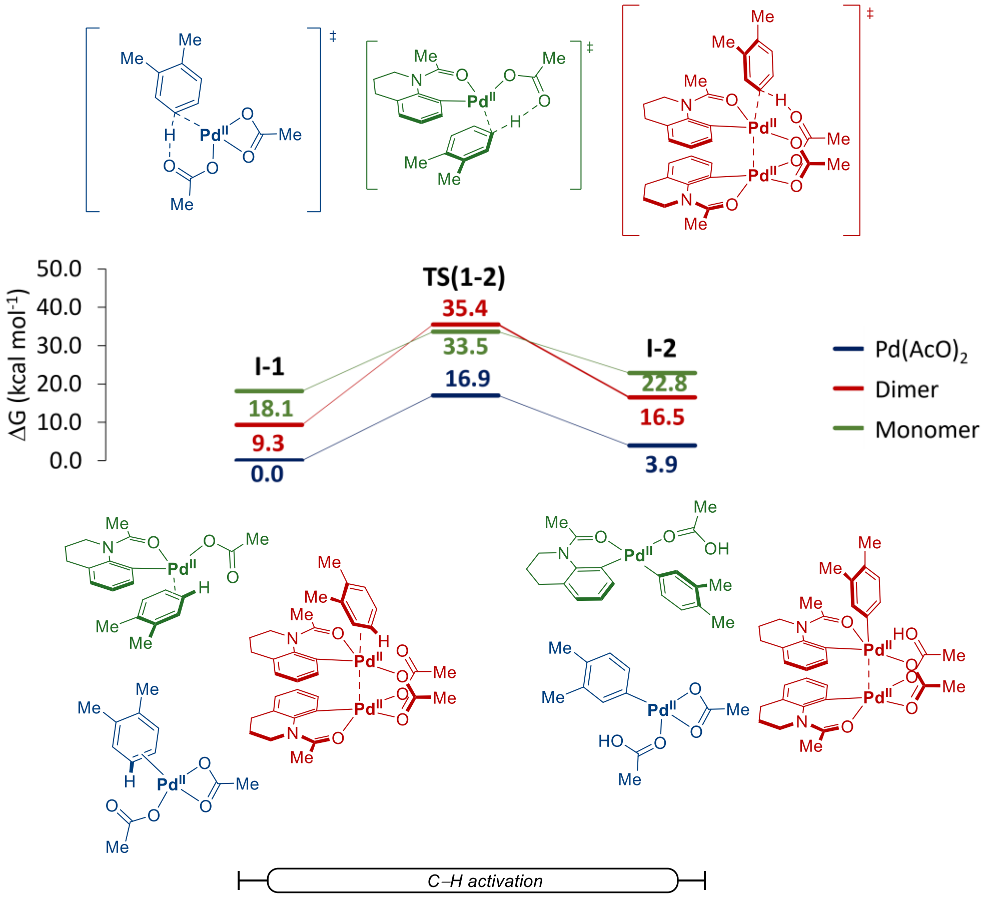

Supplementary Figure 85 Computed relative Gibbs free energies ($\Delta G_{373.15}$) in kcal mol^{-1} for three possible pathways for the C–H activation elementary step at the B3LYP-D4/6-311+G(2d,p)-SDD+SMD(HFIP)/B3LYP-D3(BJ)/6-31G(d,p)-LANL2DZ level of theory.

Supplementary Table 11 Calculated electronic energies at the B3LYP-D4/6-311+G(2d,p)-SDD +SMD(AcOH)/B3LYP-D3(BJ)/6-31G(d,p)-LANL2DZ level of theory and Gibbs free Energies with dispersion corrections for all structures in the present work (all in Hartree).[a]

Structure	Electronic Energy	Total Gibbs Free Energy
**I-1[ac]**	-896.126976	-895.938161
**TS(1-2)[ac]**	-896.095500	-895.912088
**I-2[ac]**	-896.121661	-895.932271
**I-1[m]**	-1224.171942	-1223.832046
**TS(1-2)[m]**	-1224.142170	-1223.807919
**I-2[m]**	-1224.161996	-1223.825277
**I-1[d]**	-2137.377550	-2136.806734
**TS(1-2)[d]**	-2137.336093	-2136.766506
**I-2[d]**	-2137.371416	-2136.797935
**$Pd(AcO)_2$**	-585.102829	-585.052172
**Pd-1a-dimer (53)**	-1826.376072	-1825.945686
**Pd-1a-monomer**	-913.161195	-912.962627

[a] Superscripts ac, m and d correspond to the pathways C–H activation where palladium acetate, one palladium center or a palladium dimer is involved, respectively.

Supplementary Table 12 Calculated electronic energies at the B3LYP-D4/6-311+G(2d,p)-SDD +SMD(HFIP)/B3LYP-D3(BJ)/6-31G(d,p)-LANL2DZ level of theory and Gibbs free Energies with dispersion corrections for all structures in the present work (all in Hartree).[a]

Structure	Electronic Energy	Total Gibbs Free Energy
**I-1[ac]**	-896.130453	-895.941638
**TS(1-2)[ac]**	-896.098055	-895.914643
**I-2[ac]**	-896.124775	-895.935385
**I-1[m]**	-1224.179231	-1223.839335
**TS(1-2)[m]**	-1224.148955	-1223.814704
**I-2[m]**	-1224.168574	-1223.831855
**I-1[d]**	-2137.391124	-2136.820308
**TS(1-2)[d]**	-2137.348272	-2136.778685
**I-2[d]**	-2137.382413	-2136.808932
**$Pd(AcO)_2$**	-585.102021	-585.051364
**Pd-1a-dimer (53)**	-1826.376072	-1825.945686
**Pd-1a-monomer**	-913.165556	-912.966988

[a] Superscripts ac, m and d correspond to the pathways for C–H activation where palladium acetate, one palladium center or a palladium dimer is involved, respectively.

7.8.1 Cartesian coordinates of the optimized structures

I-1[ac]

Lowest frequency = 28.0416 cm^{-1}

Charge = 0, Multiplicity = 1

```
       33

Pd     0.629904    -0.326560     0.235218
 C    -0.123291     1.754453    -0.579777
 C    -1.268330     1.472153    -1.354549
 C    -0.229678     1.738349     0.834203
 C    -2.482128     1.159602    -0.763828
 H    -1.184027     1.484142    -2.436568
 C    -1.481115     1.452384     1.429881
 H     0.602557     2.072144     1.443445
 C    -2.591062     1.148334     0.660067
 H    -1.562604     1.457421     2.512453
 C    -3.672529     0.808202    -1.614103
 H    -3.436557     0.895504    -2.676765
 H    -3.993054    -0.222148    -1.422233
 H    -4.530277     1.455661    -1.399836
 C    -3.896576     0.786857     1.315889
 H    -4.710773     1.443505     0.989196
 H    -4.189495    -0.237512     1.058691
 H    -3.822457     0.852977     2.403598
 O     2.419958     0.421717     0.710743
 C     3.160563     0.819587    -0.301863
 O     2.776143     0.916263    -1.463626
 O     0.929719    -2.464320     0.174968
 C    -0.312808    -2.605354    -0.057144
 O    -1.038850    -1.548491    -0.111303
 C    -0.912078    -3.958894    -0.291695
 H    -0.705136    -4.265237    -1.322449
 H    -0.451732    -4.690212     0.375831
 H    -1.992405    -3.926333    -0.142448
 C     4.584885     1.143014     0.121023
 H     5.123514     0.207198     0.301062
 H     5.084535     1.698148    -0.673622
 H     4.594879     1.713125     1.052996
 H     0.800292     2.049748    -1.063765
```

TS(1-2)[ac]

Lowest frequency = -933.5161 cm^{-1}

Charge = 0, Multiplicity = 1

```
   33

Pd     1.122552    -0.076118     0.070707
 C    -0.859509     0.651576    -0.075705
 C    -1.666964    -0.042268    -1.005248
 C    -1.423785     0.973749     1.177769
 C    -2.982145    -0.398591    -0.725886
 H    -1.245347    -0.306933    -1.971064
 C    -2.733826     0.617616     1.472918
 H    -0.832131     1.521584     1.905628
 C    -3.522581    -0.068720     0.540140
 H    -3.161796     0.871490     2.438993
 C    -3.811925    -1.132260    -1.748773
 H    -3.239753    -1.306134    -2.662889
 H    -4.144676    -2.106158    -1.371161
 H    -4.714234    -0.570192    -2.016970
 C    -4.937714    -0.448878     0.883737
 H    -5.652237     0.003522     0.185800
 H    -5.083439    -1.533633     0.821003
 H    -5.201628    -0.128879     1.894140
 O     1.977640     1.773418     0.333325
 C     1.495463     2.798041    -0.250181
 O     0.388660     2.828608    -0.861680
 O     2.778946    -1.575072     0.198427
 C     1.898766    -2.465759     0.004464
 O     0.673888    -2.105592    -0.161723
 C     2.245496    -3.926830    -0.003941
 H     3.297122    -4.060500    -0.261914
 H     2.075600    -4.338195     0.996743
 H     1.603056    -4.463158    -0.705153
 C     2.336826     4.053860    -0.216966
 H     2.934186     4.091633    -1.133899
 H     1.694191     4.934896    -0.192100
 H     3.013676     4.036886     0.637335
 H    -0.212015     1.668407    -0.572206
```

I-2[ac]

Lowest frequency = 26.0884 cm^{-1}

Charge = 0, Multiplicity = 1

```
   33
```

Pd	1.446333	0.081494	0.205365
C	1.355866	2.058497	0.161466
C	0.605583	2.783473	1.096919
C	2.106264	2.748744	-0.795028
C	0.586341	4.183007	1.090048
H	0.024160	2.257157	1.849515
C	2.079580	4.145098	-0.812241
H	2.722059	2.203010	-1.502439
C	1.332449	4.877190	0.115232
H	2.660916	4.678927	-1.560263
C	-0.226006	4.933048	2.116073
H	-0.740018	4.244684	2.791743
H	0.404713	5.591232	2.725336
H	-0.982861	5.571486	1.644445
C	1.329961	6.384597	0.074843
H	0.318967	6.784302	-0.070417
H	1.705436	6.813935	1.011487
H	1.957047	6.757618	-0.738663
O	-0.611969	-0.109578	0.671222
C	-1.518830	0.379083	-0.015233
O	-1.325069	1.271112	-0.966249
O	2.417788	-2.005766	0.024454
C	3.456612	-1.365541	-0.279561
O	3.427974	-0.073093	-0.334513
C	4.766476	-2.052078	-0.563564
H	4.602557	-3.115711	-0.739627
H	5.254602	-1.590019	-1.424901
H	5.429177	-1.929033	0.299310
C	-2.953402	-0.005056	0.183521
H	-3.520066	0.871384	0.511887
H	-3.378911	-0.332731	-0.768844
H	-3.024951	-0.798652	0.925074
H	-0.377547	1.551056	-0.946455

I-1^{m}

Lowest frequency = 18.9211 cm^{-1}

Charge = 0, Multiplicity = 1

51

Pd	-0.886433	-0.722044	-0.737256
C	0.784574	0.324467	-0.928346
C	0.788896	1.608515	-0.358103
C	1.934433	-0.131022	-1.571459
C	1.968215	2.375782	-0.303055
N	-0.414962	2.182222	0.185437
C	3.103669	0.635192	-1.562587

H	1.928398	-1.099450	-2.058029
C	2.041426	3.710369	0.418053
C	3.121780	1.862493	-0.906470
C	-1.616407	1.976944	-0.405376
C	-0.217157	3.115931	1.297019
H	3.997870	0.270941	-2.060864
C	0.672840	4.264399	0.826792
H	2.578612	4.436270	-0.201689
H	2.652494	3.573413	1.321047
H	4.034200	2.452431	-0.876744
O	-1.773198	1.134355	-1.325899
C	-2.832104	2.748174	0.036560
H	0.263016	2.564117	2.113910
H	-1.180987	3.461258	1.661754
H	0.781529	5.012712	1.618324
H	0.184000	4.753849	-0.023519
H	-2.642204	3.801022	0.251517
H	-3.570685	2.662444	-0.759793
H	-3.233696	2.241039	0.920480
C	-0.164757	-1.887453	1.377798
C	1.017720	-1.337041	1.899393
C	-0.083480	-2.799709	0.299832
C	2.263131	-1.663150	1.377645
H	0.952510	-0.625133	2.717114
C	1.185295	-3.149640	-0.205989
H	-0.978652	-3.309217	-0.040652
C	2.350576	-2.596167	0.309235
H	1.249928	-3.865707	-1.020585
C	3.690826	-2.953432	-0.277858
H	4.192332	-2.063893	-0.675916
H	4.360455	-3.387977	0.473188
H	3.584591	-3.674802	-1.091696
C	3.503226	-0.988413	1.898584
H	4.278865	-1.709363	2.178619
H	3.928511	-0.335074	1.127084
H	3.278215	-0.372974	2.772907
O	-2.795274	-1.662938	-0.473245
C	-3.467679	-1.065559	0.454257
O	-3.014473	-0.196371	1.224676
H	-1.123489	-1.642960	1.822050
C	-4.928626	-1.489855	0.552247
H	-5.020561	-2.575262	0.462240
H	-5.364172	-1.147692	1.492558
H	-5.485248	-1.046831	-0.280861

TS(1-2)^{m}

Lowest frequency = -1038.4158 cm^{-1}

Charge = 0, Multiplicity = 1

51

Pd	-0.107885	1.060928	-0.415825
C	0.658201	-0.776728	-0.485125
C	1.996384	-0.992277	-0.102210
C	-0.058544	-1.862543	-1.002017
C	2.587376	-2.272859	-0.184520
N	2.840912	0.085384	0.365719
C	0.512976	-3.130004	-1.099631
H	-1.083233	-1.720874	-1.320600
C	4.007484	-2.570550	0.267715
C	1.822233	-3.331595	-0.679272
C	2.788799	1.328023	-0.166753
C	3.916969	-0.312494	1.281066
H	-0.067543	-3.958153	-1.496928
C	4.825974	-1.318710	0.583892
H	4.512688	-3.179334	-0.489689
H	3.954985	-3.194184	1.170950
H	2.274321	-4.318286	-0.742199
O	1.844810	1.732115	-0.881912
C	3.917019	2.305094	0.089777
H	3.449587	-0.767138	2.162402
H	4.457896	0.568246	1.617940
H	5.685497	-1.563940	1.216041
H	5.212424	-0.861760	-0.334868
H	4.901600	1.872580	-0.106031
H	3.754096	3.158846	-0.566336
H	3.895897	2.659239	1.125712
C	-2.028513	0.282542	0.195474
C	-2.034275	-0.757526	1.150481
C	-2.905124	0.151151	-0.902371
C	-2.835104	-1.890388	1.029664
H	-1.375670	-0.676634	2.011284
C	-3.706624	-0.976664	-1.047293
H	-2.956295	0.946984	-1.640643
C	-3.677684	-2.008454	-0.098677
H	-4.369051	-1.069489	-1.904496
C	-4.541707	-3.228587	-0.281045
H	-3.938278	-4.143651	-0.312744
H	-5.246690	-3.348356	0.550450
H	-5.118887	-3.171535	-1.206985
C	-2.773192	-2.987120	2.062297
H	-3.758361	-3.202080	2.492486
H	-2.404327	-3.924126	1.627082
H	-2.101303	-2.713722	2.879486
O	-0.753838	3.149437	-0.438381
C	-1.634767	3.509966	0.388157
O	-2.295711	2.718718	1.139569
H	-2.032937	1.497161	0.714521
C	-1.962648	4.986475	0.497207
H	-2.967239	5.158671	0.098099
H	-1.974784	5.284703	1.548609
H	-1.241550	5.583806	-0.060721

I-2^m^

Lowest frequency = 16.6584 cm^{-1}

Charge = 0, Multiplicity = 1

```
   51

Pd    -0.063413     0.779940    -0.539895
 C     1.026284    -0.878986    -0.557269
 C     2.359677    -0.849012    -0.093013
 C     0.565475    -2.072032    -1.132759
 C     3.196471    -1.983228    -0.188299
 N     2.943818     0.332158     0.509296
 C     1.380806    -3.197801    -1.237700
 H    -0.453359    -2.120190    -1.495251
 C     4.624942    -2.025305     0.331731
 C     2.684574    -3.150179    -0.761592
 C     2.709062     1.591292     0.064418
 C     3.991022     0.060647     1.501396
 H     0.995734    -4.109001    -1.687234
 C     5.143193    -0.674302     0.826167
 H     5.284463    -2.429539    -0.444029
 H     4.662632    -2.742896     1.162735
 H     3.329061    -4.023004    -0.833141
 O     1.781254     1.887229    -0.715235
 C     3.609578     2.722348     0.525037
 H     3.548493    -0.563845     2.286297
 H     4.310340     0.989394     1.967128
 H     5.979441    -0.806871     1.520471
 H     5.503316    -0.062453    -0.009425
 H     4.671617     2.495106     0.400068
 H     3.351274     3.595201    -0.072861
 H     3.432694     2.961568     1.578925
 C    -1.751254    -0.267618    -0.269114
 C    -1.955627    -1.150911     0.806386
 C    -2.811833    -0.109977    -1.178587
 C    -3.154219    -1.852178     0.990584
 H    -1.149287    -1.313765     1.516029
 C    -4.006882    -0.811602    -1.012993
 H    -2.707931     0.568163    -2.021656
 C    -4.200450    -1.684655     0.062179
 H    -4.811048    -0.678408    -1.733915
 C    -5.502827    -2.428261     0.221671
 H    -5.353106    -3.514961     0.213136
 H    -5.994037    -2.188225     1.172821
 H    -6.198370    -2.181241    -0.584765
 C    -3.313146    -2.782871     2.167367
 H    -4.149416    -2.484348     2.811646
 H    -3.518251    -3.810913     1.844452
```

H	-2.407413	-2.799125	2.778990
O	-1.146618	2.762700	-0.544903
C	-2.024071	3.080265	0.256304
O	-2.598736	2.240571	1.103957
H	-2.285193	1.324517	0.899599
C	-2.556849	4.482646	0.358630
H	-3.601709	4.495761	0.033464
H	-2.534659	4.813873	1.400196
H	-1.965760	5.149219	-0.267397

I-1[d]

Lowest frequency = 11.0197 cm^{-1}

Charge = 0, Multiplicity = 1

84

Pd	-0.429894	0.578360	-0.858620
Pd	-2.088481	-1.260752	0.581858
O	-3.357314	0.110680	1.585328
C	-4.138789	0.991493	1.058828
O	-4.164866	1.371323	-0.121939
O	-3.186305	-1.190303	-1.137461
C	-3.038458	-0.342538	-2.076933
O	-2.016336	0.360623	-2.306222
C	-4.229059	-0.181409	-2.990546
H	-3.928797	0.221994	-3.958637
H	-4.756682	-1.129613	-3.108786
H	-4.896407	0.526625	-2.489036
C	-5.117417	1.604463	2.065831
H	-4.584809	1.929262	2.964955
H	-5.651151	2.444950	1.618695
H	-5.838830	0.842480	2.378985
O	-1.206320	-1.520347	2.392067
C	-0.004199	-1.792949	2.609328
C	2.295163	-2.271272	2.101241
C	0.463359	-2.842725	0.423381
C	3.236854	-2.657595	0.977754
H	2.432404	-2.977538	2.928181
H	2.537542	-1.270078	2.460533
C	1.374073	-3.717340	-0.214951
C	2.747759	-3.959002	0.357579
H	4.245374	-2.745598	1.396242
H	3.257778	-1.870632	0.219431
H	3.421506	-4.308285	-0.430966
H	2.728885	-4.744236	1.127415
N	0.865490	-2.280008	1.695136
C	-0.786392	-2.567587	-0.167904
C	1.018939	-4.321208	-1.419562

C	-1.117911	-3.230724	-1.358163
C	-0.232225	-4.099223	-1.985960
H	-2.083545	-3.029748	-1.804489
H	-0.509766	-4.587032	-2.916111
H	1.735276	-4.982389	-1.900765
C	0.465412	-1.565576	4.028166
H	1.187745	-0.747138	4.074134
H	-0.410486	-1.295499	4.615724
H	0.932801	-2.459151	4.451218
C	1.023483	0.865321	0.419378
C	2.390568	0.792670	0.092261
C	0.641018	1.190590	1.722976
C	3.366171	1.090511	1.073967
N	2.864531	0.403896	-1.214198
C	1.596200	1.502334	2.689664
H	-0.414449	1.215163	1.969869
C	4.861112	1.057696	0.805157
C	2.946387	1.455732	2.357457
C	2.191836	-0.458526	-2.032049
C	4.211670	0.870924	-1.569708
H	1.285167	1.785436	3.691136
C	5.222715	0.409438	-0.529043
H	5.367646	0.549427	1.632582
H	5.236343	2.090295	0.809822
H	3.701399	1.695807	3.101643
O	0.992204	-0.740927	-1.899982
C	2.929781	-1.149407	-3.162382
H	4.185996	1.966176	-1.607569
H	4.463031	0.523043	-2.567483
H	6.236897	0.685936	-0.833709
H	5.187138	-0.683373	-0.458051
H	3.906124	-1.533628	-2.857088
H	2.296056	-1.977059	-3.478810
H	3.071068	-0.474760	-4.013247
C	-1.625859	2.901760	-0.623627
C	-0.518166	3.079421	-1.479633
C	-1.571535	3.428681	0.675544
C	0.643918	3.747752	-1.040211
H	-0.592855	2.759708	-2.515615
C	-0.432742	4.094718	1.102171
H	-2.424027	3.298935	1.333003
C	0.688991	4.248681	0.267037
H	-0.386159	4.492602	2.112602
C	1.939687	4.899919	0.798913
H	2.748092	4.163483	0.890641
H	2.300826	5.699035	0.142050
H	1.770173	5.327400	1.790147
C	1.824766	3.890280	-1.964065
H	2.012378	4.937727	-2.230034
H	2.739709	3.518077	-1.490166
H	1.669083	3.329637	-2.889407
H	-2.534531	2.415170	-0.960269

TS(1-2)[d]

Lowest frequency = -1173.2108 cm^{-1}

Charge = 0, Multiplicity = 1

84

Pd	-0.253286	0.324561	-0.976829
Pd	-1.429701	-1.981830	0.820028
O	-3.571624	-1.320234	0.936949
C	-4.223604	-1.019073	-0.098781
O	-3.795657	-0.331255	-1.085944
O	-1.951164	-2.872792	-0.978609
C	-1.854154	-2.237691	-2.084646
O	-1.318413	-1.123334	-2.282597
C	-2.515075	-2.915489	-3.270434
H	-1.988406	-2.666809	-4.193287
H	-2.563958	-3.996222	-3.129309
H	-3.535137	-2.523472	-3.342052
C	-5.645788	-1.538122	-0.210187
H	-6.120337	-1.541913	0.773254
H	-6.227825	-0.948477	-0.919850
H	-5.602132	-2.574738	-0.562179
O	-0.939637	-1.066730	2.601611
C	0.159671	-0.488656	2.787688
C	2.495751	0.041511	2.475247
C	1.487753	-2.046601	1.423644
C	3.757411	-0.390286	1.751120
H	2.697736	0.030795	3.552011
H	2.226689	1.056848	2.184980
C	2.787834	-2.589922	1.316493
C	3.957084	-1.887424	1.956180
H	4.588956	0.204123	2.144130
H	3.672842	-0.173747	0.683707
H	4.892522	-2.236113	1.508307
H	4.017666	-2.105750	3.032234
N	1.329546	-0.846717	2.212549
C	0.413000	-2.664751	0.757588
C	2.997098	-3.732851	0.546398
C	0.654821	-3.829102	0.017677
C	1.934795	-4.363449	-0.095731
H	-0.176346	-4.291449	-0.501131
H	2.103420	-5.258889	-0.687546
H	4.006341	-4.127665	0.463676
C	0.133522	0.687510	3.734352
H	0.354029	1.611526	3.192374
H	-0.871784	0.748494	4.147182
H	0.859788	0.587991	4.545440
C	1.016436	1.706462	-0.258749

C	2.361013	1.744433	-0.711211
C	0.657221	2.640045	0.724663
C	3.302116	2.640763	-0.147607
N	2.878451	0.878683	-1.749415
C	1.566064	3.545661	1.269472
H	-0.360635	2.665898	1.076028
C	4.764876	2.715396	-0.562996
C	2.886124	3.533620	0.842174
C	2.480608	-0.405304	-1.918897
C	4.014099	1.410465	-2.512675
H	1.236791	4.252749	2.026238
C	5.185856	1.646738	-1.570644
H	5.394892	2.670054	0.332207
H	4.939789	3.705621	-1.004036
H	3.613527	4.220863	1.266777
O	1.424388	-0.856453	-1.442928
C	3.339402	-1.380873	-2.693643
H	3.692583	2.354370	-2.967886
H	4.258157	0.727377	-3.322547
H	6.076923	1.960206	-2.123627
H	5.426313	0.702672	-1.067704
H	4.404223	-1.278792	-2.472862
H	3.001263	-2.377556	-2.410695
H	3.191375	-1.256299	-3.771571
C	-2.046099	1.467877	-0.410924
C	-2.096802	2.635859	-1.206354
C	-2.386509	1.613871	0.958140
C	-2.414178	3.894578	-0.691888
H	-1.873152	2.553613	-2.267245
C	-2.758019	2.847434	1.474486
H	-2.405694	0.734324	1.591401
C	-2.753621	4.001459	0.673431
H	-3.047875	2.935755	2.519211
C	-3.119657	5.335869	1.267497
H	-2.304779	6.060852	1.152918
H	-3.994793	5.769723	0.768875
H	-3.348020	5.247175	2.332373
C	-2.397847	5.113635	-1.579574
H	-3.371032	5.618521	-1.597424
H	-1.663735	5.852572	-1.235598
H	-2.141736	4.842458	-2.606730
H	-2.727371	0.418730	-0.834836

I-2[d]

Lowest frequency = 20.5461 cm^{-1}

Charge = 0, Multiplicity = 1

84

Pd	0.413463	-0.566642	-0.884289
Pd	0.652722	2.213725	0.620007
O	2.890395	2.122092	0.946125
C	3.878997	1.734665	0.318562
O	3.867502	1.242058	-0.898104
O	0.819279	3.009898	-1.305671
C	1.108984	2.213150	-2.247454
O	1.537402	1.025248	-2.097783
C	0.922684	2.723607	-3.657688
H	0.006761	2.269076	-4.051173
H	0.820156	3.808850	-3.676979
H	1.752645	2.398767	-4.288251
C	5.257235	1.774947	0.921051
H	5.250531	2.366491	1.835524
H	5.551239	0.745444	1.146540
H	5.974079	2.178709	0.202202
O	0.399456	1.378859	2.471931
C	-0.520092	0.559713	2.727611
C	-2.654818	-0.553504	2.506899
C	-2.185039	1.619277	1.271476
C	-3.988346	-0.489881	1.786300
H	-2.843170	-0.509727	3.585041
H	-2.149601	-1.491724	2.280701
C	-3.578435	1.832501	1.158004
C	-4.539358	0.927789	1.883919
H	-4.651032	-1.232996	2.241362
H	-3.860147	-0.760203	0.736048
H	-5.535401	1.007170	1.438306
H	-4.637997	1.214654	2.940831
N	-1.741017	0.568597	2.151947
C	-1.296373	2.420863	0.534883
C	-4.058032	2.831298	0.313063
C	-1.808774	3.437432	-0.279181
C	-3.180001	3.644836	-0.397611
H	-1.117447	4.042805	-0.851847
H	-3.559584	4.429248	-1.046370
H	-5.132400	2.969059	0.226352
C	-0.200869	-0.491453	3.761194
H	-0.178666	-1.479034	3.290846
H	0.784985	-0.265695	4.163644
H	-0.933453	-0.516991	4.572103
C	-0.682515	-2.040082	-0.093406
C	-2.002943	-2.282735	-0.553715
C	-0.231979	-2.836044	0.975377
C	-2.835273	-3.245359	0.070456
N	-2.602717	-1.582806	-1.674022
C	-1.032072	-3.801200	1.585284
H	0.782032	-2.703528	1.327588
C	-4.267675	-3.545233	-0.350415
C	-2.332356	-3.993472	1.137632
C	-2.365793	-0.285876	-2.009797

C	-3.624621	-2.346662	-2.403029
H	-0.638617	-4.397465	2.404419
C	-4.790767	-2.652663	-1.475210
H	-4.921946	-3.482708	0.526481
H	-4.308367	-4.591295	-0.681100
H	-2.976517	-4.732863	1.607247
O	-1.376951	0.358324	-1.626955
C	-3.356684	0.454905	-2.888150
H	-3.156288	-3.277155	-2.744841
H	-3.930611	-1.796407	-3.288983
H	-5.605614	-3.143375	-2.017216
H	-5.182365	-1.707020	-1.082164
H	-4.397020	0.230483	-2.643035
H	-3.170275	1.516540	-2.726920
H	-3.191628	0.228874	-3.947137
C	2.127441	-1.343553	-0.234165
C	2.967583	-2.041574	-1.110476
C	2.599826	-1.094542	1.061410
C	4.247772	-2.471001	-0.738092
H	2.629502	-2.249815	-2.122774
C	3.872524	-1.522332	1.447506
H	1.995017	-0.536128	1.767417
C	4.713269	-2.208185	0.565711
H	4.223305	-1.313958	2.457150
C	6.087383	-2.652036	1.003045
H	6.208051	-3.740270	0.931888
H	6.875839	-2.210713	0.380279
H	6.281727	-2.364206	2.040257
C	5.117924	-3.205764	-1.727808
H	6.056513	-2.669775	-1.916106
H	5.393925	-4.203948	-1.365484
H	4.605162	-3.327062	-2.685560
H	2.938817	1.108711	-1.250248

Pd(AcO)$_2$

Lowest frequency = 33.4479 cm^{-1}

Charge = 0, Multiplicity = 1

15

Pd	-2.004466	-1.014647	0.548100
O	0.012337	-1.522456	0.506316
C	0.328309	-0.283120	0.534217
O	-0.638309	0.553748	0.577486
O	-4.021262	-0.506852	0.590387
C	-4.337374	-1.745872	0.552011
O	-3.370618	-2.583057	0.519217
C	-5.762847	-2.191353	0.515958

H	-6.087543	-2.257409	-0.527941
H	-5.856774	-3.179033	0.970465
H	-6.394213	-1.464064	1.029121
C	1.752680	0.164865	0.487281
H	2.049590	0.294076	-0.558983
H	1.859434	1.123391	0.997911
H	2.396869	-0.591563	0.938661

Pd-1a-dimer (50)

Lowest frequency = 21.4715 cm^{-1}

Charge = 0, Multiplicity = 1

66

Pd	0.725944	-0.997385	-1.083971
Pd	2.029175	0.628887	0.931537
O	3.270432	-1.188514	0.938179
C	2.933951	-2.271913	0.404981
O	1.896269	-2.512180	-0.312199
O	3.146774	1.345989	-0.647556
C	3.186921	0.735118	-1.766381
O	2.469344	-0.230238	-2.129028
C	4.248101	1.227501	-2.733624
H	3.960098	1.002145	-3.761313
H	4.417327	2.297862	-2.603836
H	5.186799	0.707911	-2.513611
C	3.852087	-3.467644	0.594108
H	4.452933	-3.346212	1.496271
H	3.274465	-4.393092	0.632864
H	4.523993	-3.527984	-0.268801
O	1.068323	0.019030	2.618394
C	-0.173665	0.052204	2.773337
C	-2.467107	0.567780	2.259929
C	-0.583294	1.979569	1.286008
C	-3.344501	1.369045	1.319517
H	-2.752702	0.806463	3.291296
H	-2.625487	-0.498738	2.096579
C	-1.532785	2.989711	1.002684
C	-2.970036	2.840562	1.433427
H	-4.389960	1.179818	1.585453
H	-3.194011	1.028984	0.291983
H	-3.611082	3.469888	0.808342
H	-3.113612	3.175060	2.471115
N	-1.019717	0.852842	2.083865
C	0.733625	2.102071	0.800387
C	-1.149833	4.106952	0.261278
C	1.089621	3.255995	0.091997
C	0.161457	4.256329	-0.180759

H	2.102705	3.335461	-0.283453
H	0.453834	5.136218	-0.747035
H	-1.896588	4.865878	0.042701
C	-0.727771	-0.876570	3.827427
H	-1.295440	-1.685716	3.360455
H	0.121675	-1.304373	4.357483
H	-1.382589	-0.359766	4.533835
C	-0.936352	-1.753535	-0.325294
C	-2.221014	-1.393386	-0.785270
C	-0.845288	-2.681855	0.719636
C	-3.384996	-1.940942	-0.194173
N	-2.430190	-0.442049	-1.852539
C	-1.982806	-3.240267	1.300919
H	0.140060	-2.978989	1.056170
C	-4.800232	-1.607267	-0.638615
C	-3.242091	-2.862389	0.846592
C	-1.557304	0.539974	-2.181390
C	-3.692965	-0.583415	-2.595202
H	-1.885910	-3.970628	2.099816
C	-4.877684	-0.464055	-1.649017
H	-5.413734	-1.385196	0.241442
H	-5.236668	-2.508232	-1.090143
H	-4.137851	-3.285027	1.294583
O	-0.356762	0.551713	-1.843572
C	-2.022031	1.738341	-2.979777
H	-3.683570	-1.573062	-3.068127
H	-3.728160	0.154895	-3.391832
H	-5.816941	-0.503512	-2.209542
H	-4.836310	0.509854	-1.149891
H	-3.027563	2.064064	-2.706103
H	-1.312918	2.539139	-2.769936
H	-1.998014	1.528379	-4.054224

Pd-1a-monomer

Lowest frequency = 21.3953 cm^{-1}

Charge = 0, Multiplicity = 1

33

Pd	3.034697	-1.155381	1.617010
C	2.192118	-2.283082	2.977479
C	2.892090	-3.116301	3.868887
C	0.794649	-2.278381	2.992774
C	2.181659	-3.971898	4.743473
N	4.335014	-3.133043	3.941502
C	0.088582	-3.101819	3.866725
H	0.269703	-1.614365	2.314736
C	2.854971	-4.920140	5.719140

C	0.785214	-3.945616	4.723944
C	5.152367	-2.752264	2.937679
C	4.893609	-3.626935	5.211865
H	-0.997378	-3.089510	3.872255
C	4.360986	-5.023631	5.500545
H	2.383852	-5.906374	5.650922
H	2.667679	-4.562923	6.741050
H	0.246014	-4.606410	5.397798
O	4.767328	-2.188205	1.885795
C	6.642377	-3.008248	3.026504
H	4.585494	-2.932317	6.002903
H	5.977749	-3.610039	5.164969
H	4.858337	-5.443953	6.380191
H	4.592086	-5.672363	4.647687
H	6.873535	-4.047867	3.272403
H	7.063170	-2.764521	2.052171
H	7.111272	-2.362450	3.775683
O	3.260415	0.460357	-0.024169
C	2.054242	0.720198	0.218084
O	1.418159	0.053348	1.123667
C	1.307784	1.806558	-0.512876
H	0.385964	1.399974	-0.938021
H	1.023874	2.592074	0.194229
H	1.931526	2.229006	-1.301017

8 Crystallographic Data

Supplementary Table 13 Crystal data and structure refinement for 36

CCDC number	2281047
Empirical formula	$C_{17}H_{15}Cl_2NO$
Formula weight	320.20
Temperature [K]	200.00
Crystal system	monoclinic
Space group (number)	$P2_1/n$ (14)
a [Å]	8.6706(8)
b [Å]	15.0012(14)
c [Å]	12.2862(9)
α [°]	90
β [°]	110.160(2)
γ [°]	90
Volume [Å^3]	1500.2(2)
Z	4
ρ_{calc} [gcm^{-3}]	1.418
μ [mm^{-1}]	0.430
F(000)	664
Crystal size [mm^3]	0.552×0.285×0.176
Crystal colour	colourless
Crystal shape	block
Radiation	MoK_α (λ=0.71073 Å)
2θ range [°]	4.45 to 61.06 (0.70 Å)
Index ranges	−12 ≤ h ≤ 10<br>0 ≤ k ≤ 21<br>0 ≤ l ≤ 17
Reflections collected	4515
Independent reflections	4515<br>R_{int} = 0.0449<br>R_{sigma} = 0.0260
Completeness to θ = 25.242°	100.0 %
Data / Restraints / Parameters	4515/2/202
Goodness-of-fit on F^2	1.050
Final *R* indexes [$I \geq 2\sigma(I)$]	R_1 = 0.0391<br>wR_2 = 0.0991
Final *R* indexes [all data]	R_1 = 0.0451<br>wR_2 = 0.1038
Largest peak/hole [eÅ^{-3}]	0.38/-0.30

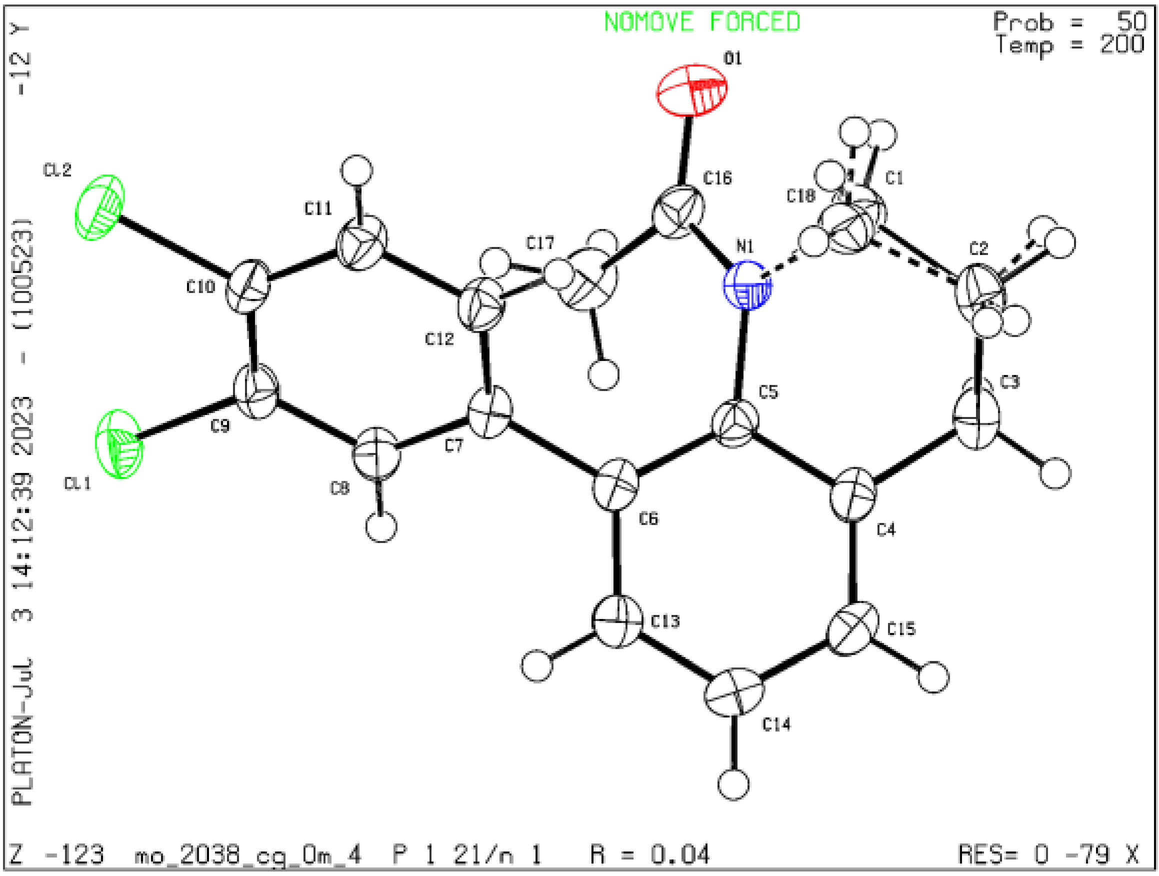

Supplementary Figure 86 Crystal Structure of 36

Supplementary Table 14 Crystal data and structure refinement for 25

CCDC number	2281048
Empirical formula	$C_{18}H_{19}NO_2$
Formula weight	281.34
Temperature [K]	200.00
Crystal system	monoclinic
Space group (number)	$P2_1/n$ (14)
a [Å]	8.4396(11)
b [Å]	9.1461(14)
c [Å]	19.060(3)
α [°]	90
β [°]	101.813(5)
γ [°]	90
Volume [Å^3]	1440.1(4)
Z	4
ρ_{calc} [gcm^{-3}]	1.298
μ [mm^{-1}]	0.084
F(000)	600
Crystal size [mm^3]	0.671×0.321×0.064
Crystal colour	colourless
Crystal shape	plate
Radiation	MoK_α (λ=0.71073 Å)
2θ range [°]	4.37 to 61.16 (0.70 Å)
Index ranges	−11 ≤ h ≤ 11<br>−13 ≤ k ≤ 12<br>−27 ≤ l ≤ 24
Reflections collected	38513
Independent reflections	4354<br>R_{int} = 0.0434<br>R_{sigma} = 0.0203
Completeness to θ = 25.242°	100.0 %
Data / Restraints / Parameters	4354/69/259
Goodness-of-fit on F^2	1.057
Final *R* indexes [I≥2σ(I)]	R_1 = 0.0433<br>wR_2 = 0.1182
Final *R* indexes [all data]	R_1 = 0.0530<br>wR_2 = 0.1266
Largest peak/hole [eÅ^{-3}]	0.31/-0.18

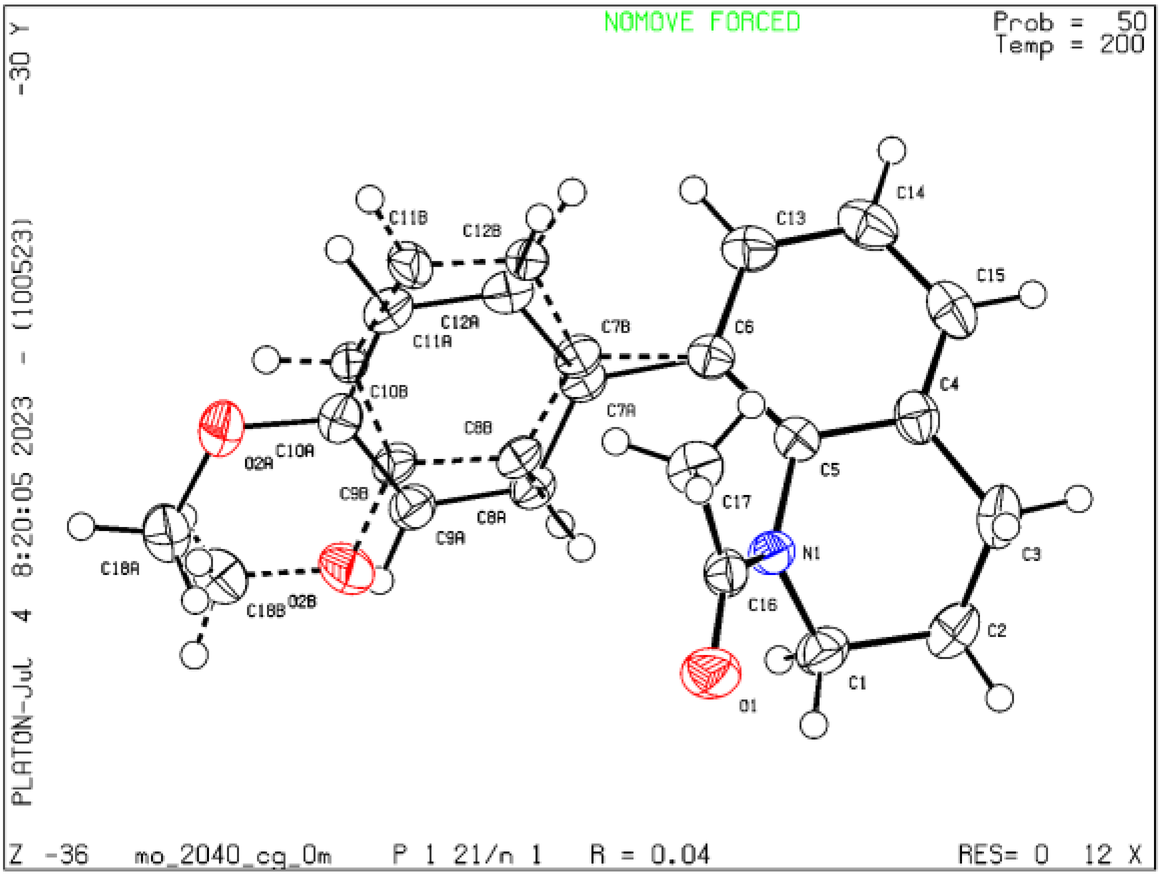

Supplementary Figure 87 Crystal Structure of 25. A disorder where the methoxy group sits in *meta* position (ca. 7%) instead of *para* was found during the crystallographic analysis.

Supplementary Table 15 Crystal data and structure refinement for 22

CCDC number	2281049
Empirical formula	$C_{17}H_{17}NO$
Formula weight	251.31
Temperature [K]	100.00
Crystal system	monoclinic
Space group (number)	$P2_1/c$ (14)
a [Å]	8.3205(6)
b [Å]	9.6385(6)
c [Å]	16.4100(11)
α [°]	90
β [°]	97.311(2)
γ [°]	90
Volume [Å^3]	1305.34(15)
Z	4
ρ_{calc} [gcm^{-3}]	1.279
μ [mm^{-1}]	0.079
F(000)	536
Crystal size [mm^3]	0.517×0.424×0.317
Crystal colour	colourless
Crystal shape	block
Radiation	MoK_α (λ=0.71073 Å)
2θ range [°]	4.91 to 65.27 (0.66 Å)
Index ranges	−12 ≤ h ≤ 12<br>−14 ≤ k ≤ 14<br>−24 ≤ l ≤ 24
Reflections collected	52821
Independent reflections	4750<br>R_{int} = 0.0319<br>R_{sigma} = 0.0144
Completeness to θ = 25.242°	100.0 %
Data / Restraints / Parameters	4750/0/173
Goodness-of-fit on F^2	1.048
Final *R* indexes [I≥2σ(I)]	R_1 = 0.0377<br>wR_2 = 0.1107
Final *R* indexes [all data]	R_1 = 0.0389<br>wR_2 = 0.1121
Largest peak/hole [eÅ^{-3}]	0.45/-0.23

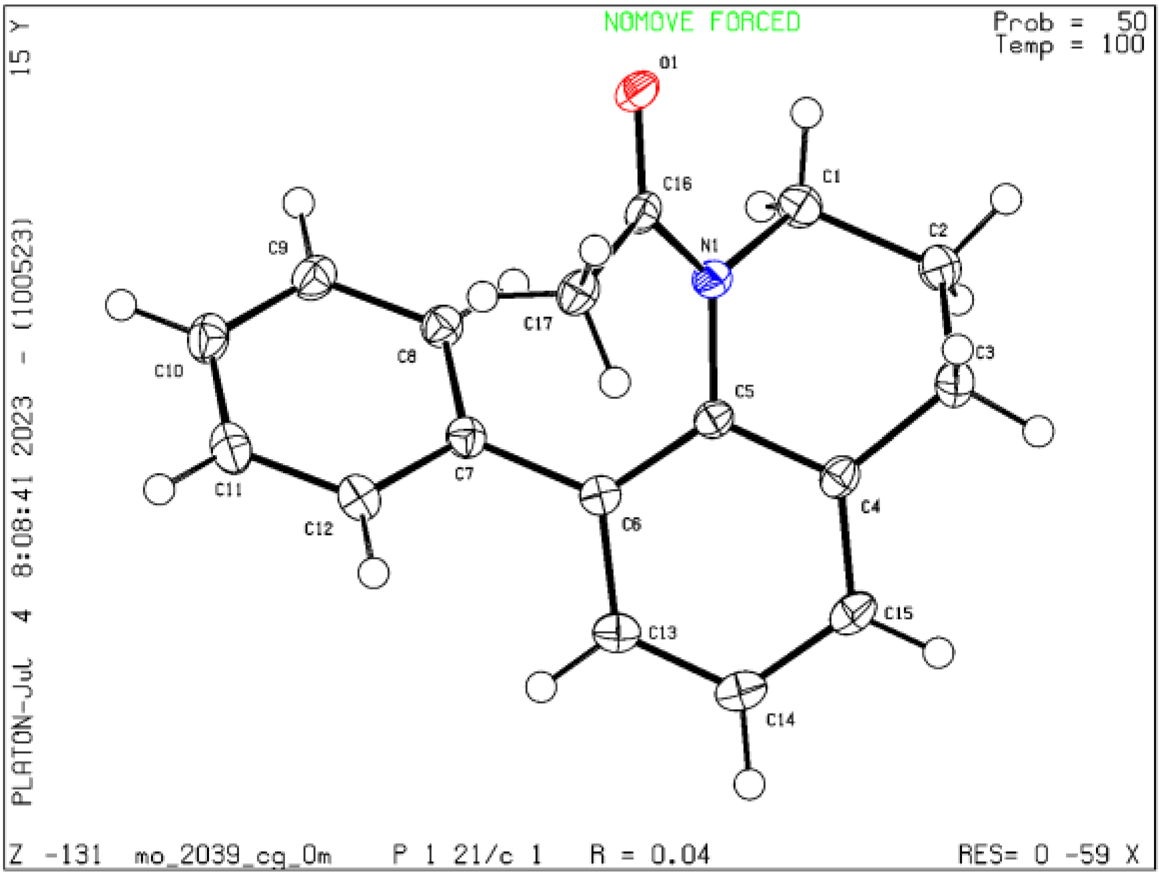

Supplementary Figure 88 **Crystal Structure of 22**

Supplementary Table 16 Crystal data and structure refinement for 26

CCDC number	2285982
Empirical formula	$C_{17}H_{17}NO_2$
Formula weight	267.31
Temperature [K]	100.00
Crystal system	monoclinic
Space group (number)	$P2_1/c$ (14)
a [Å]	8.6183(4)
b [Å]	9.5414(9)
c [Å]	16.0190(13)
α [°]	90
β [°]	92.995(3)
γ [°]	90
Volume [Å^3]	1315.45(17)
Z	4
ρ_{calc} [gcm^{-3}]	1.350
μ [mm^{-1}]	0.088
F(000)	568
Crystal size [mm^3]	0.428×0.206×0.182
Crystal colour	colourless
Crystal shape	block
Radiation	MoK_α (λ=0.71073 Å)
2θ range [°]	4.73 to 59.20 (0.72 Å)
Index ranges	−11 ≤ h ≤ 10<br>−13 ≤ k ≤ 12<br>−22 ≤ l ≤ 22
Reflections collected	34011
Independent reflections	3651<br>R_{int} = 0.0279<br>R_{sigma} = 0.0140
Completeness to θ = 25.242°	100.0 %
Data / Restraints / Parameters	3651/0/186
Goodness-of-fit on F^2	1.044
Final *R* indexes [I≥2σ(I)]	R_1 = 0.0371<br>wR_2 = 0.0964
Final *R* indexes [all data]	R_1 = 0.0400<br>wR_2 = 0.0989
Largest peak/hole [eÅ^{-3}]	0.44/-0.21

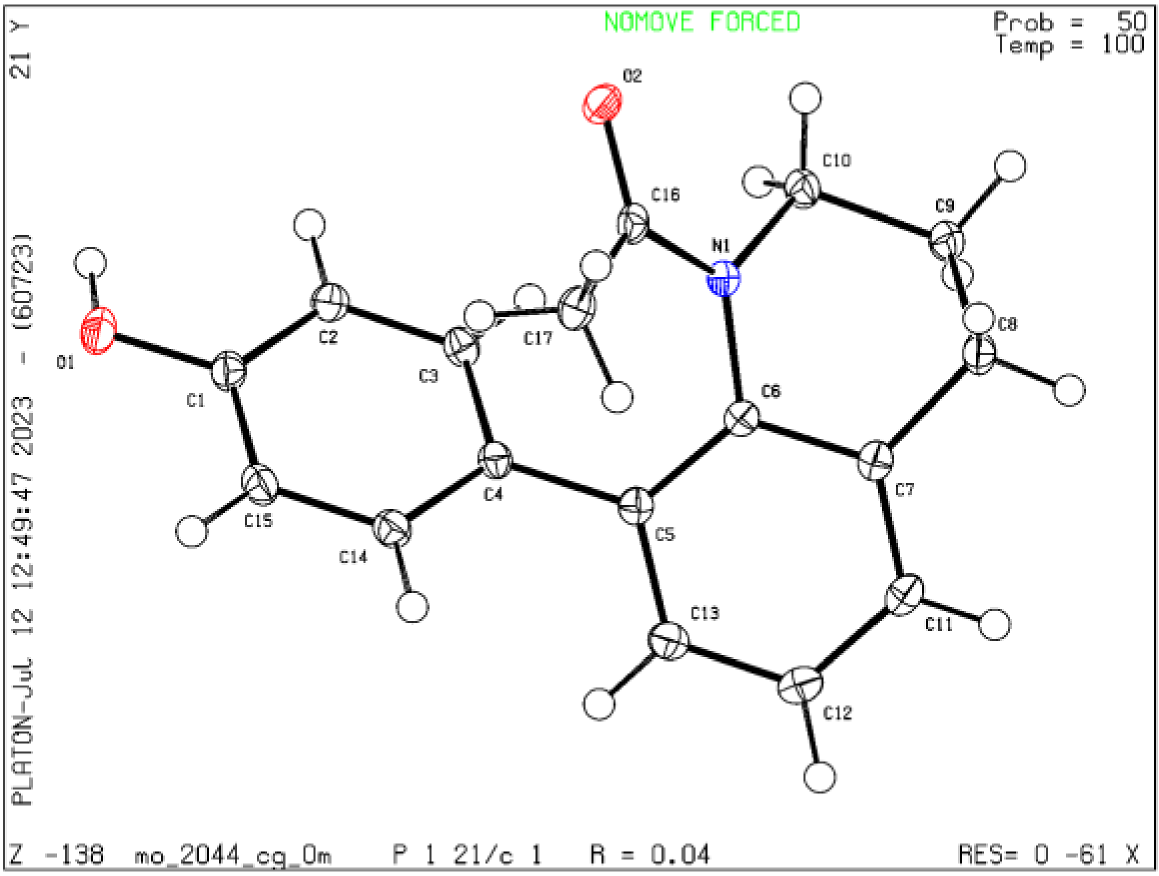

Supplementary Figure 89 **Crystal Structure of 26**

Supplementary Table 17 Crystal data and structure refinement for 56

CCDC number	2302226
Empirical formula	$C_{13}H_{13}F_3N_2O_3Pd$
Formula weight	408.65
Temperature [K]	100.00
Crystal system	triclinic
Space group (number)	$P\overline{1}$ (2)
a [Å]	9.5709(5)
b [Å]	11.3898(5)
c [Å]	14.4554(9)
α [°]	75.640(2)
β [°]	76.595(2)
γ [°]	79.334(3)
Volume [Å^3]	1471.30(14)
Z	4
ρ_{calc} [gcm^{-3}]	1.845
μ [mm^{-1}]	1.307
F(000)	808
Crystal size [mm^3]	0.341×0.156×0.153
Crystal colour	yellow
Crystal shape	block
Radiation	MoK_{α} (λ=0.71073 Å)
2θ range [°]	4.23 to 61.09 (0.70 Å)
Index ranges	−12 ≤ h ≤ 13<br>−15 ≤ k ≤ 16<br>−20 ≤ l ≤ 20
Reflections collected	14923
Independent reflections	14923<br>R_{int} = 0.0214<br>R_{sigma} = 0.0157
Completeness to θ = 25.242°	99.8 %
Data / Restraints / Parameters	14923/0/412
Goodness-of-fit on F^2	1.066
Final *R* indexes [I≥2σ(I)]	R_1 = 0.0203<br>wR_2 = 0.0559
Final *R* indexes [all data]	R_1 = 0.0217<br>wR_2 = 0.0570
Largest peak/hole [eÅ^{-3}]	0.75/-0.71

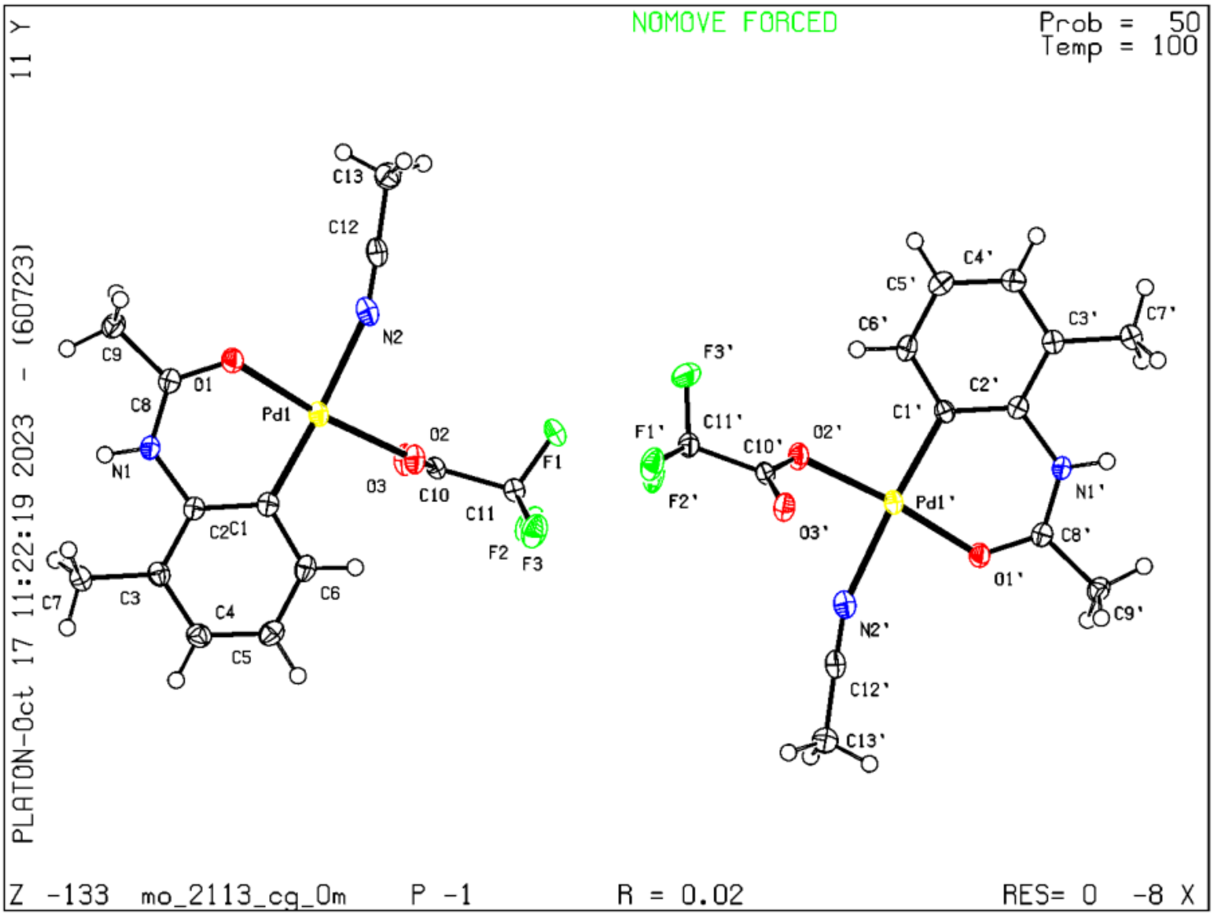

Supplementary Figure 90 Crystal Structure of 56

9 Unsuccessful Simple Arenes

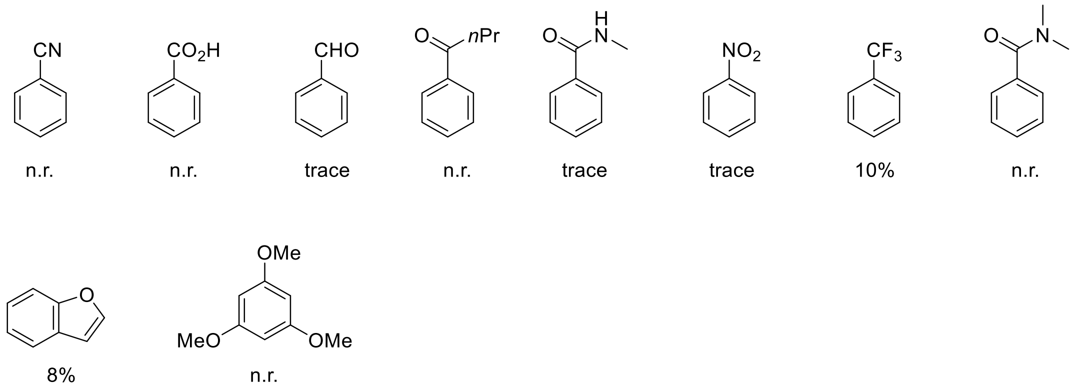

Supplementary Figure 91 Failed arene scope

10 Boscalid

10.1 Synthetic Route

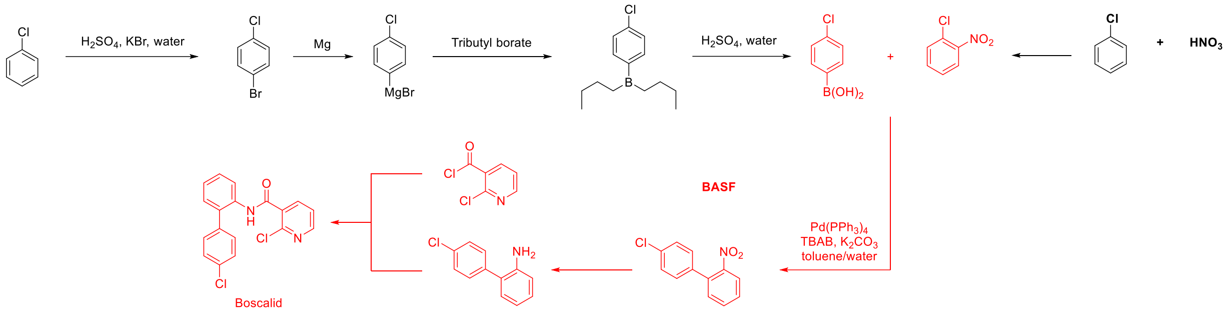

Supplementary Figure 92 Synthetic route of Boscalid in industry. The route was obtained from the literature.[14]

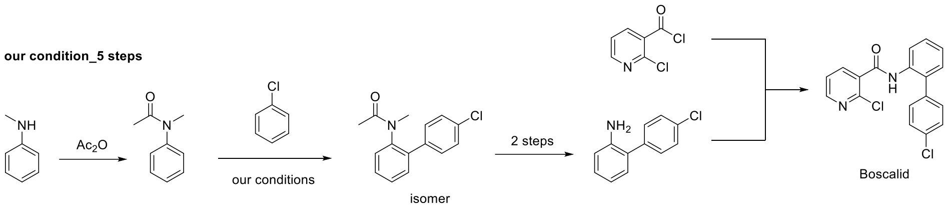

Supplementary Figure 93 Our synthetic route

10.2 Prices of the chemicals

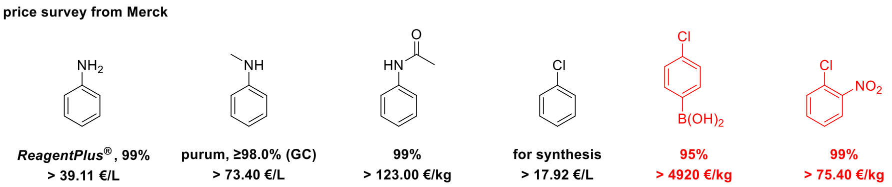

Supplementary Figure 94 The prices of the chemical offered by the Merck online website

10.3 *N*-demethylation

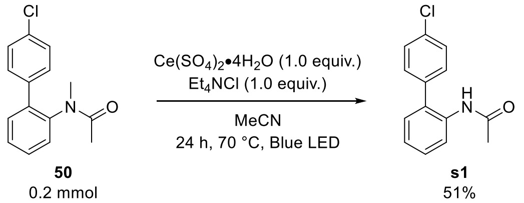

The *N*-demethylation of amide was conducted following the procedure reported previously.[15] To a 10 mL vial contained a magnetic stir bar, amide **50** (0.2 mmol), Et_4NCl (1.0 equiv.), $Ce(SO_4)_2{\cdot}4H_2O$ (1.0 equiv.), and CH_3CN (1.0 mL) were added under atmosphere of air. The

mixture was stirred at 1400 RPM for 24 h under irradiation by blue LEDs at 70 °C. The reaction mixture was concentrated under reduced pressure and purified by column chromatography on silica gel (*n*-hexane/EtOAc = 2:1) to afford demethylated amide **s1** in 51% yield. ^{1}H NMR (400 MHz, $CDCl_3$) δ 8.23 (d, *J* = 8.2 Hz, 1H), 7.53 – 7.47 (m, 2H), 7.42 (ddd, *J* = 8.5, 6.7, 2.3 Hz, 1H), 7.38 – 7.33 (m, 2H), 7.28 – 7.20 (m, 2H), 7.05 (s, 1H), 2.08 (s, 3H). ^{13}C NMR (101 MHz, $CDCl_3$) δ 168.3 (C_q), 136.7 (C_q), 134.6 (C_q), 134.2 (C_q), 130.6 (CH), 130.0 (CH), 129.3 (CH), 128.8 (CH), 124.7 (CH), 122.3 (CH), 24.6 (CH_3). IR (ATR): 3416, 3272, 3028, 1663, 1518, 1476, 1444, 1370, 1288, 1090 cm^{-1}. MS (ESI) *m*/*z* (relative intensity): 268 (100) $[M + Na]^+$, 246 (10) $[M + H]^+$. HR-MS (ESI): *m*/*z* calcd. for $[C_{14}H_{12}ClNO + Na]^+$ 268.0500 found 268.0503.

11 Additional Studies for the Role of Copper

11.1 CV studies

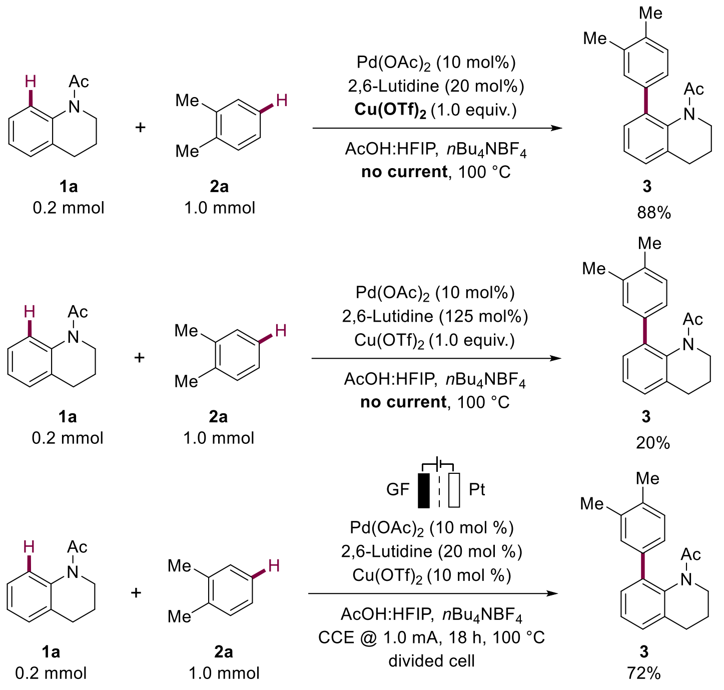

Supplementary Figure 95 Reaction using stoichiometric $Cu(OTf)_2$ or electricity

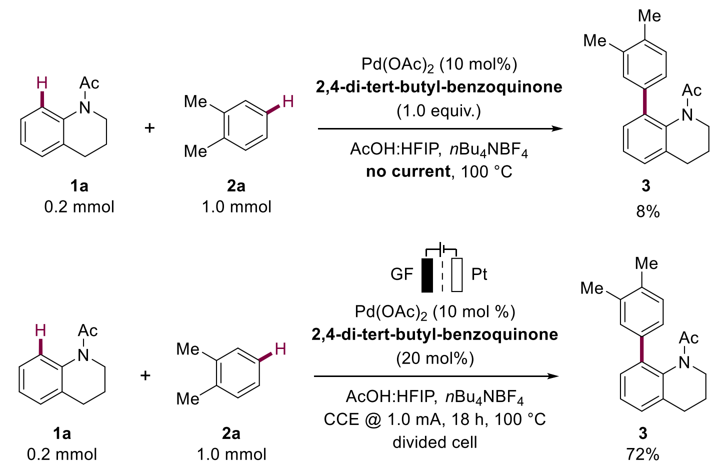

Supplementary Figure 96 Reaction using stoichiometric BQ or electricity

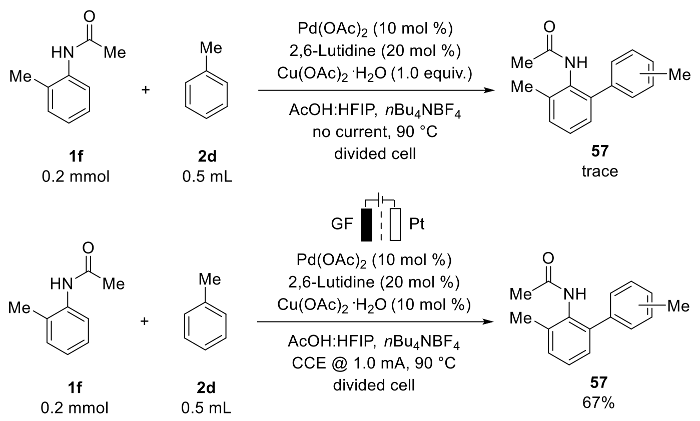

Supplementary Figure 97 Reaction using stoichiometric $Cu(OAc)_2 \cdot H_2O$ or electricity.

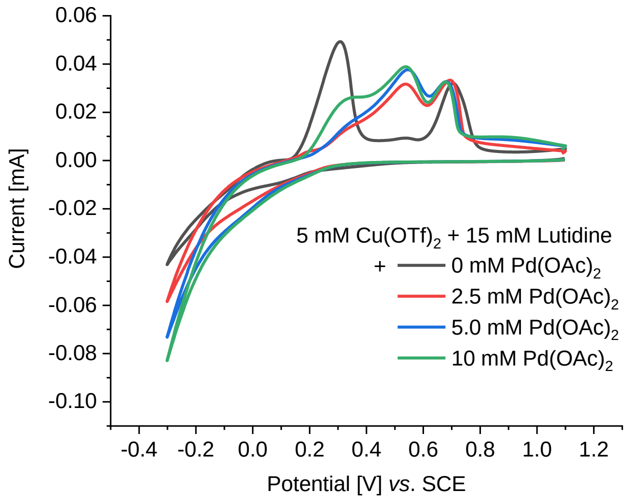

Supplementary Figure 98 Cyclic voltammetry titration experiments. $Pd(OAc)_2$ (0–15 mM), $Cu(OTf)_2$ (5 mM) and Lutidine (15 mM). The voltammograms were recorded at room temperature in HFIP:AcOH (1.3 mL:2.6 mL) with 0.1 M nBu_4NBF_4 as supporting electrolyte under N_2 atmosphere. The scan rate is 100 mV/s.

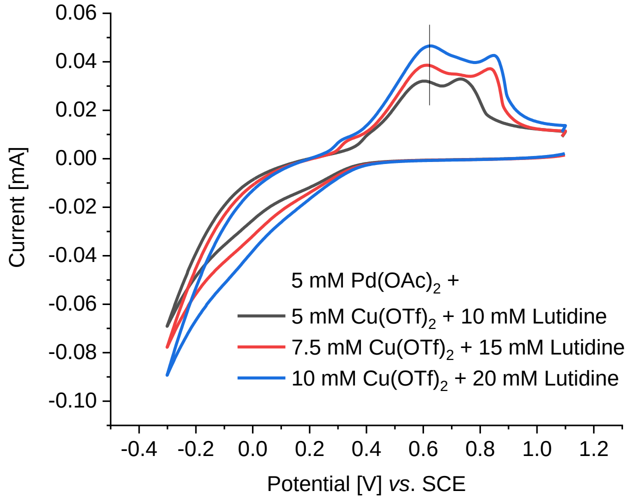

Supplementary Figure 99 Cyclic voltammetry titration experiments. $Pd(OAc)_2$ (5 mM), $Cu(OTf)_2$ (5–10 mM) and Lutidine (10–20 mM). The voltammograms were recorded at room temperature in HFIP:AcOH (1.3 mL:2.6 mL) with 0.1 M nBu_4NBF_4 as supporting electrolyte under N_2 atmosphere. The scan rate is 100 mV/s.

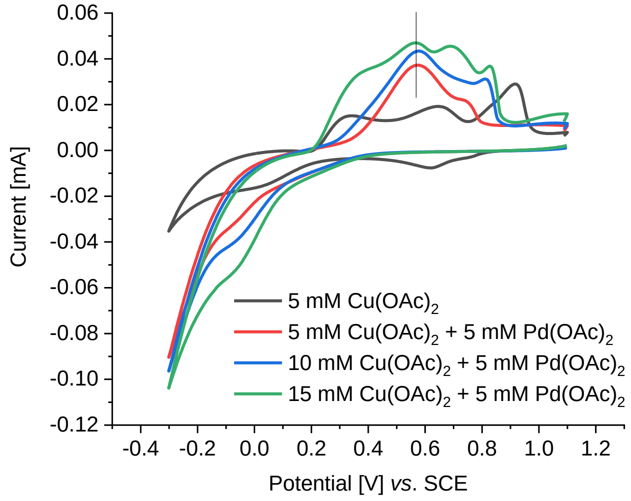

Supplementary Figure 100 Cyclic voltammetry titration experiments. $Pd(OAc)_2$ (5 mM), $Cu(OAc)_2 \cdot H_2O$ (5–15 mM). The voltammograms were recorded at room temperature in HFIP:AcOH (1.3 mL:2.6 mL) with 0.1 M nBu_4NBF_4 as supporting electrolyte under N_2 atmosphere. The scan rate is 100 mV/s.

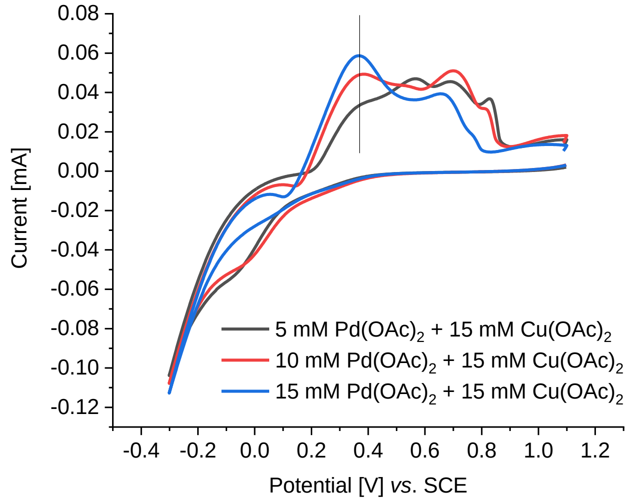

Supplementary Figure 101 Cyclic voltammetry titration experiments. $Pd(OAc)_2$ (5–15 mM), $Cu(OAc)_2 \cdot H_2O$ (15 mM). The voltammograms were recorded at room temperature in HFIP:AcOH (1.3 mL:2.6 mL) with 0.1 M nBu_4NBF_4 as supporting electrolyte under N_2 atmosphere. The scan rate is 100 mV/s.

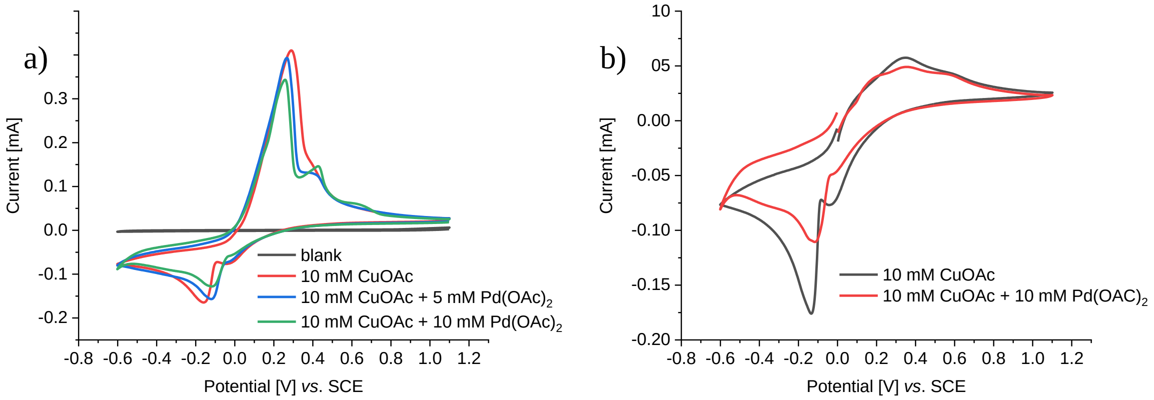

Supplementary Figure 102 Cyclic voltammetry titration experiments. a) Starting voltage at 1.1 V; b) starting potential at 0 V. $Pd(OAc)_2$ (5–10 mM), CuOAc (10 mM). The voltammograms were recorded at room temperature in MeOH (4 mL) with 0.1 M nBu_4NBF_4 as supporting electrolyte under N_2 atmosphere. The scan rate is 100 mV/s.

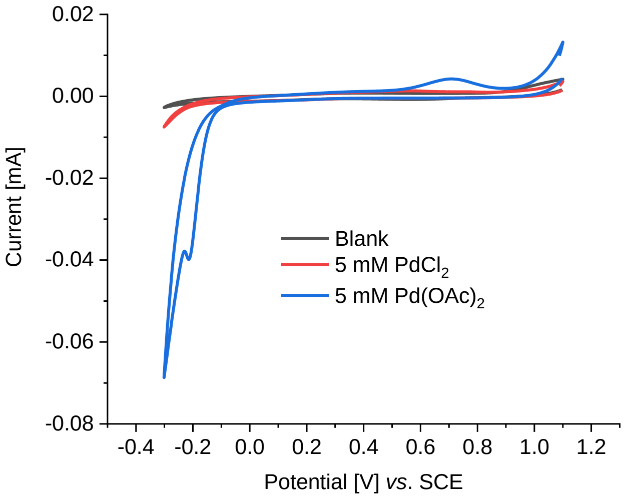

Supplementary Figure 103 Cyclic voltammetry. The voltammograms were recorded at room temperature in MeOH (4 mL) with 0.1 M nBu_4NBF_4 as supporting electrolyte under N_2 atmosphere. The scan rate is 100 mV/s.

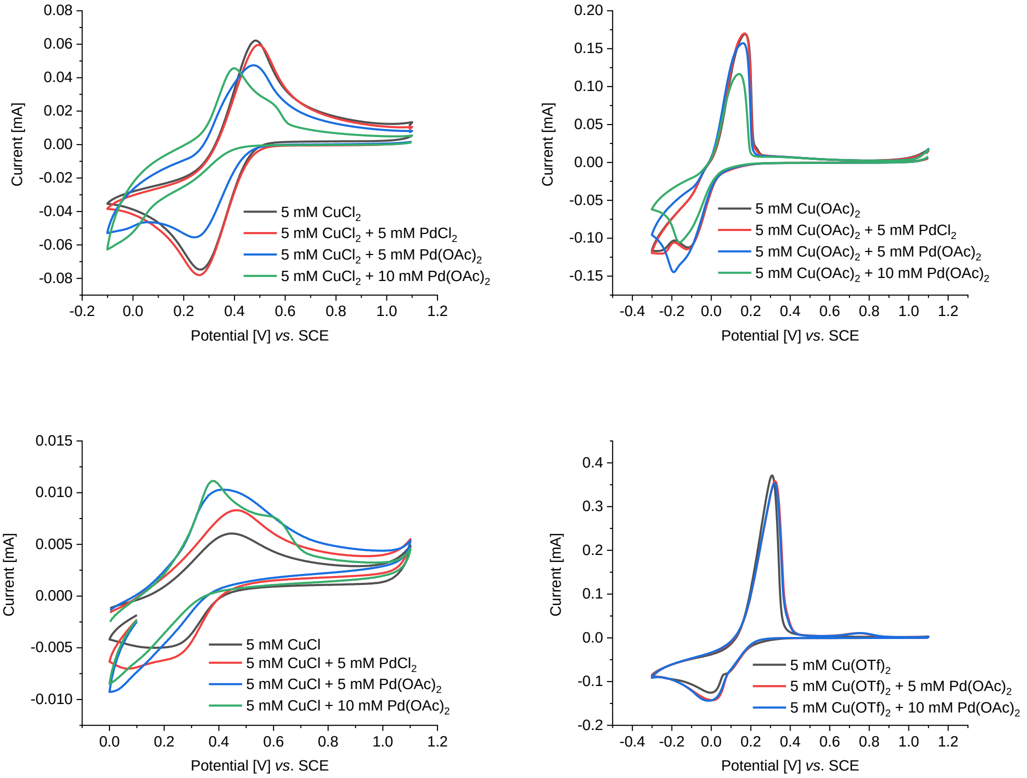

Supplementary Figure 104 Cyclic voltammetry titration experiments. The voltammograms were recorded at room temperature in MeOH (4 mL) with 0.1 M nBu_4NBF_4 as supporting electrolyte under N_2 atmosphere. The scan rate is 100 mV/s.

11.2 Reaction Profiles

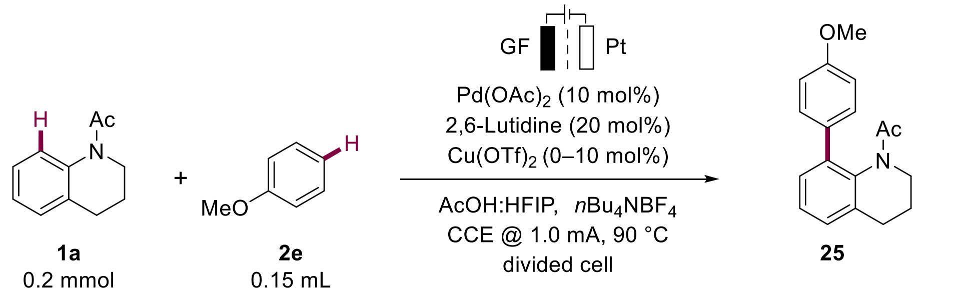

Reaction conditions: divided cell, anodic chamber: **1a** (0.20 mmol), **2e** (0.15 mL), $Pd(OAc)_2$ (10 mol %), 2,6-lutidine (20 mol %), $Cu(OTf)_2$ (0–10 mol %), $n$$Bu_4NBF_4$ (200 mg), HFIP:AcOH (5.4 mL, 1 : 2), cathodic chamber: **2e** (0.15 mL), $n$$Bu_4NBF_4$ (200 mg), HFIP:AcOH (5.4 mL, 1 : 2), 90 °C, electrolysis (CCE) at 1.0 mA, graphite felt (GF) anode (10 mm × 15 mm × 2 mm), Pt-plate cathode (10 mm × 15 mm × 0.25 mm), GC yield. At specific intervals shown on the horizontal axis, a small aliquot of the reaction mixture (0.05 mL) was

taken, filtered through a small silica gel column, added with 0.05 mL internal standard stock solution (0.2 mmol dodecane in 5.5 mL EtOAc), and analyzed by GC. Only the *para*-isomer was quantified for the kinetics studies. To obtain a reliable result, data was generated by using the same reactor, potentiostat, heating plate, and GC.

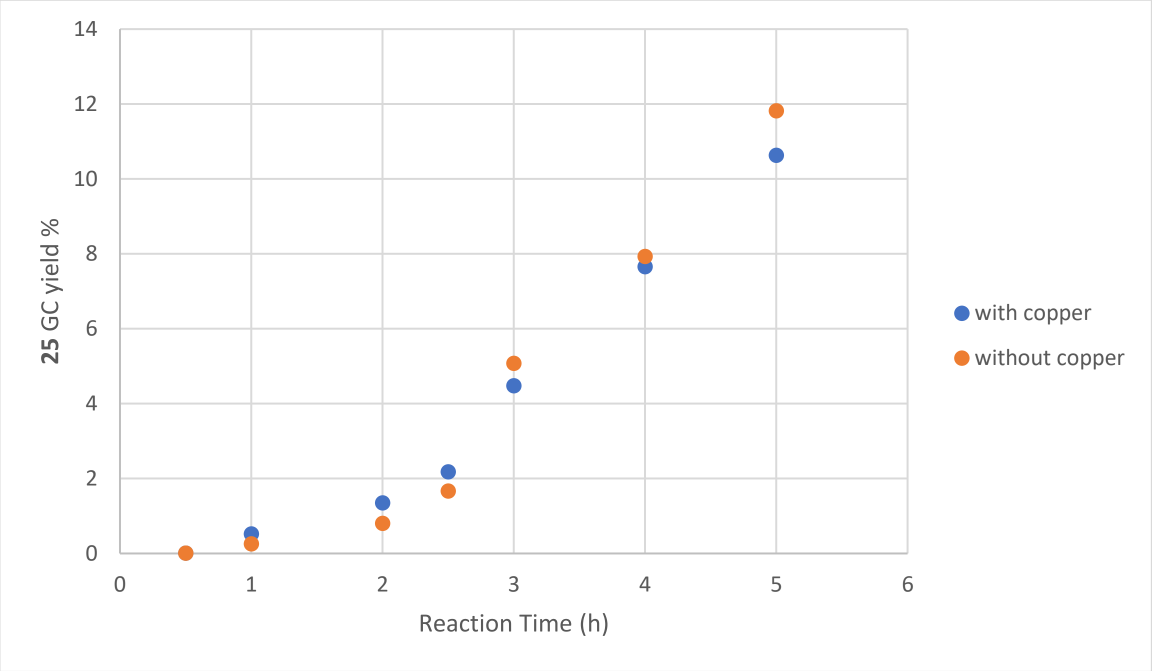

Supplementary Figure 105 Reaction profiling for the role of copper 1

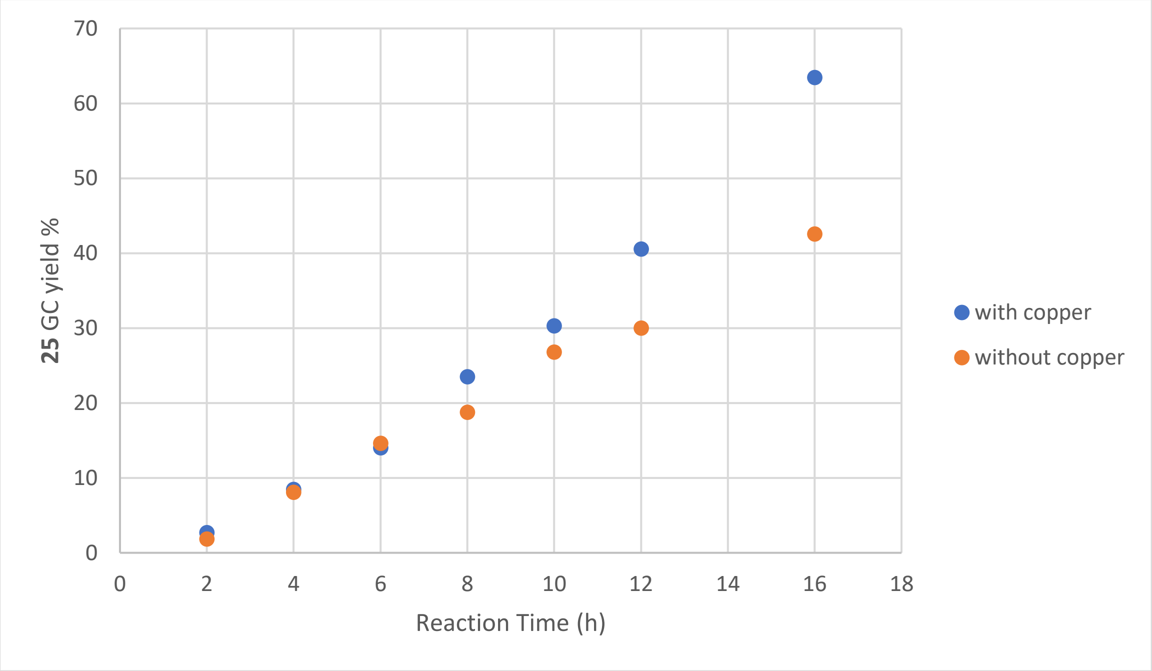

Supplementary Figure 106 Reaction profiling for the role of copper 2

12 Characterization

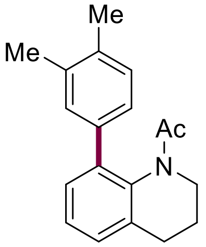

1-(8-(3,4-dimethylphenyl)-3,4-dihydroquinolin-1(*2H*)-yl)ethan-1-one (3)

The general procedure **A** was followed using 1-(3,4-dihydroquinolin-1(*2H*)-yl)ethan-1-one (**1a**) (31.7 µL, 0.20 mmol) and *o*-xylene (**2a**) (120.6 µL, 1.0 mmol, 5.0 equiv.) and *n*Bu_4NBF_4 (40 mg, 0.12 mmol, 0.61 equiv.) at 100 °C for 20 h. Isolation by column chromatography (*n*-hexane/EtOAc = 2:1) yielded **3** (40.1 mg, 72%). The product is known and the characterization is in consistence with that reported in the literature.[16] ^{1}H NMR (400 MHz, $CDCl_3$) δ 7.31 (dd, *J* = 7.8, 1.8, 1H), 7.25 (t, *J* = 7.5 Hz, 1H), 7.18 – 7.14 (m, 2H), 7.11 (d, *J* = 1.9 Hz, 1H), 7.07 (dd, *J* = 7.8, 2.0 Hz, 1H), 4.83 – 4.71 (m, 1H), 3.12 – 3.02 (m, 1H), 2.77 – 2.68 (m, 1H), 2.55 – 2.44 (m, 1H), 2.39 – 2.22 (m, 7H), 1.83 – 1.69 (m, 1H), 1.46 (s, 3H). ^{13}C NMR (101 MHz, $CDCl_3$) δ 170.3 (C_q), 138.0 (C_q), 137.8 (C_q), 137.5 (C_q), 137.0 (C_q), 136.5 (C_q), 135.9 (C_q), 130.2 (CH), 129.4 (CH), 128.6 (CH), 126.7 (CH), 126.3 (CH), 125.5 (CH), 41.6 (CH_2), 26.8 (CH_2), 24.3 (CH_2), 21.9 (CH_3), 19.9 (CH_3), 19.4 (CH_3). IR (ATR): 2939, 2877, 1655, 1448, 1374, 1338, 1260, 1208, 1025, 791 cm^{-1}. MS (ESI) *m*/*z* (relative intensity): 302 (100) $[M + Na]^+$, 280 (30) $[M + H]^+$. HR-MS (ESI): *m*/*z* calcd. for $[C_{19}H_{20}NO + Na]^+$ 302.1515 found 302.1520.

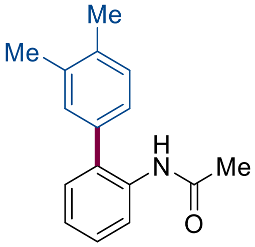

***N*-(3',4'-dimethyl-[1,1'-biphenyl]-2-yl)acetamide (4)**

The general procedure **A** was followed using *N*-phenylacetamide (**1b**) (27.0 mg, 0.20 mmol) and *o*-xylene (**2a**) (1.0 mL, 8.3 mmol) and *n*Bu_4NBF_4 (40 mg, 0.12 mmol, 0.61 equiv.) at 100 °C for 18 h. Isolation by column chromatography (*n*-hexane/EtOAc = 5:1) yielded **4** (mono-arylated product 21.3 mg, 45% and di-arylated product 14.5 mg, 21%). The product is known and the characterization is in consistence with that reported in the literature.[17] Mono-arylated product, ^{1}H NMR (400 MHz, $CDCl_3$) δ 8.28 (d, *J* = 8.2 Hz, 1H), 7.34 (ddd, *J* = 8.6, 7.2, 1.8 Hz, 1H), 7.25 – 7.19 (m, 3H), 7.18 – 7.07 (m, 3H), 2.33 (s, 3H), 2.33 (s, 3H), 2.02 (s, 3H). ^{13}C NMR

(101 MHz, $CDCl_3$) δ 168.2 (C_q), 137.5 (C_q), 136.5 (C_q), 135.6 (C_q), 134.8 (C_q), 132.1 (C_q), 130.5 (CH), 130.3 (CH), 130.1 (CH), 128.2 (CH), 126.5 (CH), 124.2 (CH), 121.3 (CH), 24.7 (CH_3), 19.9 (CH_3), 19.5 (CH_3). IR (ATR): 3415, 3279, 2923, 1663, 1583, 1518, 1444, 1367, 1296, 760 cm^{-1}. MS (ESI) *m/z* (relative intensity): 262 (100) $[M + Na]^+$, 240 (10) $[M + H]^+$. HR-MS (ESI): *m/z* calcd. for $[C_{16}H_{17}NO + Na]^+$ 262.1202 found 262.1204.

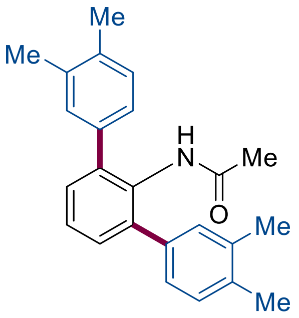

N-(3,3'',4,4''-tetramethyl-[1,1':3',1''-terphenyl]-2'-yl)acetamide (di-4)

^{1}H NMR (300 MHz, $CDCl_3$) δ 7.45 – 7.28 (m, 3H), 7.19 – 7.06 (m, 6H), 6.56 (s, 1H), 2.30 (s, 12H), 1.74 (s, 3H). ^{13}C NMR (75 MHz, $CDCl_3$) δ 169.3 (C_q), 140.6 (C_q), 137.4 (C_q), 136.4 (C_q), 135.6 (C_q), 131.2 (C_q), 130.0 (CH), 129.8 (CH), 129.5 (CH), 127.6 (CH), 126.1 (CH), 23.0 (CH_3), 19.8 (CH_3), 19.5 (CH_3). IR (ATR): 3242, 3018, 2970, 2920, 1660, 1507, 1445, 1368, 1294, 797 cm^{-1}. MS (ESI) *m/z* (relative intensity): 366 (100) $[M + Na]^+$, 344 (50) $[M + H]^+$. HR-MS (ESI): *m/z* calcd. for $[C_{24}H_{25}NO + Na]^+$ 366.1828 found 366.1819.

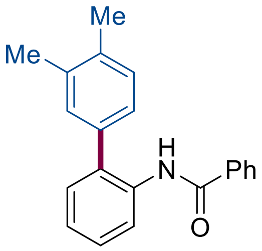

***N* -(3',4'-dimethyl-[1,1'-biphenyl]-2-yl)benzamide (5)**

The general procedure **A** was followed using *N*-phenylbenzamide (**1c**) (39.5 mg, 0.20 mmol) and *o*-xylene (**2a**) (1.0 mL, 8.3 mmol) and *n*Bu_4NBF_4 (200 mg, 0.61 mmol, 3.0 equiv.) at 100 °C for 20 h. Isolation by column chromatography (*n*-hexane/EtOAc = 10:1) yielded **5** (mono-arylated product 20.5 mg, 34% and di-arylated product 30.0 mg, 37%). Mono-arylated product, ^{1}H NMR (400 MHz, $CDCl_3$) δ 8.56 (d, *J* = 8.2 Hz, 1H), 8.13 (s, 1H), 7.73 – 7.65 (m, 2H), 7.51 – 7.46 (m, 1H), 7.44 – 7.37 (m, 3H), 7.31 – 7.25 (m, 2H), 7.24 – 7.16 (m, 3H), 2.35 (s, 3H), 2.33 (s, 3H). ^{13}C NMR (101 MHz, $CDCl_3$) δ 165.0 (C_q), 137.7 (C_q), 136.7 (C_q), 135.5 (C_q), 135.0 (C_q), 132.3 (C_q), 131.7 (CH), 130.6 (CH), 130.4 (CH), 130.1 (CH), 128.8 (CH), 128.3 (CH), 126.9 (CH), 126.6 (CH), 124.3 (CH), 120.9 (CH), 19.8 (CH_3), 19.6 (CH_3). IR (ATR):

3421, 3059, 3030, 2920, 1677, 1582, 1519, 1444, 1302, 758 cm^{-1}. MS (ESI) *m*/*z* (relative intensity): 324 (100) $[M + Na]^+$, 302 (30) $[M + H]^+$. HR-MS (ESI): *m*/*z* calcd. for $[C_{21}H_{19}NO + Na]^+$ 324.1359 found 324.1367.

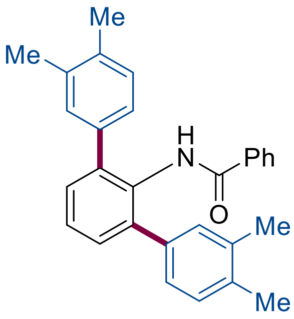

N-(3,3'',4,4''-tetramethyl-[1,1':3',1''-terphenyl]-2'-yl)benzamide (di-5)

^{1}H NMR (400 MHz, $CDCl_3$) δ 7.47 – 7.36 (m, 6H), 7.31 (t, *J* = 7.5 Hz, 2H), 7.25 (s, 2H), 7.21 (d, *J* = 7.9 Hz, 2H), 7.12 (m, 3H), 2.24 (s, 6H), 2.22 (s, 6H). ^{13}C NMR (101 MHz, $CDCl_3$) δ 167.3 (C_q), 140.6 (C_q), 137.3 (C_q), 136.4 (C_q), 135.6 (C_q), 135.4 (C_q), 131.3 (C_q), 131.2 (CH), 130.1 (CH), 129.8 (CH), 129.6 (CH), 128.4 (CH), 127.6 (CH), 126.9 (CH), 126.1 (CH), 19.8 (CH_3), 19.5 (CH_3). IR (ATR): 3278, 3260, 3238, 1640, 1520, 1488, 1451, 1304, 1240, 795 cm^{-1}. MS (ESI) *m*/*z* (relative intensity): 428 (90) $[M + Na]^+$, 406 (100) $[M + H]^+$. HR-MS (ESI): *m*/*z* calcd. for $[C_{29}H_{27}NO + H]^+$ 406.2165 found 406.2155.

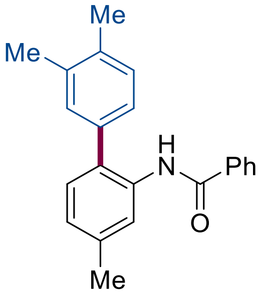

N-(3',4,4'-trimethyl-[1,1'-biphenyl]-2-yl)benzamide (6)

The general procedure **A** was followed using *N*-(*m*-tolyl)benzamide (**1d**) (42.3 mg, 0.20 mmol) and *o*-xylene (**2a**) (1.0 mL, 8.3 mmol) and *n*Bu_4NBF_4 (200 mg, 0.61 mmol, 3.0 equiv.) at 100 °C for 20 h. Isolation by column chromatography (*n*-hexane/EtOAc = 10:1) yielded **6** (55.0 mg, 87%). ^{1}H NMR (300 MHz, $CDCl_3$) δ 8.41 (s, 1H), 8.13 (s, 1H), 7.69 – 7.62 (m, 2H), 7.48 (d, *J* = 7.1 Hz, 1H), 7.45 – 7.37 (m, 2H), 7.29 – 7.15 (m, 4H), 7.03 (d, *J* = 7.9 Hz, 1H), 2.45 (s, 3H), 2.35 (s, 3H), 2.33 (s, 3H). ^{13}C NMR (75 MHz, $CDCl_3$) δ 165.0 (C_q), 138.3 (C_q), 137.6 (C_q), 136.4 (C_q), 135.6 (C_q), 135.0 (C_q), 134.8 (C_q), 131.6 (CH), 130.7 (CH), 130.4 (CH), 129.8 (CH), 129.6 (C_q), 128.7 (CH), 126.9 (CH), 126.7 (CH), 125.1 (CH), 121.5 (CH), 21.6 (CH_3), 19.8 (CH_3), 19.6 (CH_3). IR (ATR): 3420, 3058, 3020, 2918, 1677, 1577, 1530, 1464, 1294, 706 cm^-

[1]. MS (ESI) *m*/*z* (relative intensity): 338 (100) [M + Na]$^+$, 316 (100) [M + H]$^+$. HR-MS (ESI): *m*/*z* calcd. for [$C_{22}H_{21}NO$ + H]$^+$ 316.1696 found 316.1690.

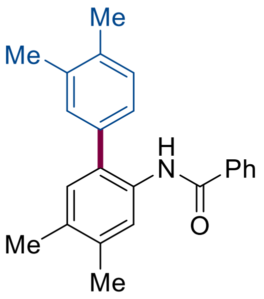

***N*-(3',4,4',5-tetramethyl-[1,1'-biphenyl]-2-yl)benzamide (7)**

The general procedure **A** was followed using *N*-(3,4-dimethylphenyl)benzamide (**1e**) (45.0, 0.20 mmol) and *o*-xylene (**2a**) (1.0 mL, 8.3 mmol) and *n*Bu_4NBF_4 (200 mg, 0.61 mmol, 3.0 equiv.) at 100 °C for 20 h. Isolation by column chromatography (*n*-hexane/EtOAc = 10:1) yielded **7** (51.1 mg, 78%). ^{1}H NMR (300 MHz, $CDCl_3$) δ 8.32 (s, 1H), 8.04 (s, 1H), 7.69 – 7.62 (m, 2H), 7.47 (d, *J* = 7.2 Hz, 1H), 7.44 – 7.37 (m, 2H), 7.27 – 7.19 (m, 2H), 7.17 (d, *J* = 7.6 Hz, 1H), 7.08 (s, 1H), 2.36 (s, 3H), 2.34 (s, 3H), 2.32 (s, 3H), 2.29 (s, 3H). ^{13}C NMR (75 MHz, $CDCl_3$) δ 164.9 (C_q), 137.5 (C_q), 136.7 (C_q), 136.3 (C_q), 135.6 (C_q), 135.1 (C_q), 132.7 (C_q), 132.5 (C_q), 131.5 (CH), 131.1 (CH), 130.6 (CH), 130.3 (CH), 130.1 (C_q), 128.7 (CH), 126.9 (CH), 126.6 (CH), 122.4 (CH), 19.9 (CH_3), 19.8 (CH_3), 19.6 (CH_3), 19.3 (CH_3). IR (ATR): 3421, 3308, 2970, 2918, 1673, 1578, 1520, 1493, 1452, 1292 cm^{-1}. MS (ESI) *m*/*z* (relative intensity): 352 (80) [M + Na]$^+$, 330 (100) [M + H]$^+$. HR-MS (ESI): *m*/*z* calcd. for [$C_{23}H_{23}NO$ + H]$^+$ 330.1852 found 330.1850.

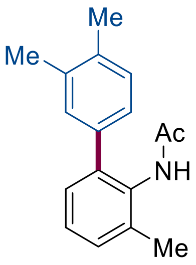

***N*-(3,3',4'-trimethyl-[1,1'-biphenyl]-2-yl)acetamide (8)**

The general procedure **A** was followed using *N*-(*o*-tolyl)acetamide (**1f**) (29.8 mg, 0.20 mmol) and *o*-xylene (**2a**) (120.6 µL, 1.0 mmol, 5.0 equiv.) and *n*Bu_4NBF_4 (40 mg, 0.12 mmol, 0.61 equiv.) at 100 °C for 20 h. Isolation by column chromatography (*n*-hexane/EtOAc = 2:1) yielded **8** (44.3 mg, 87%). The product is known and the characterization is in consistence with that reported in the literature.[17] Rotamers in a ratio of 5:1 could be found on ^{1}H NMR and ^{13}C NMR.[18] For the major rotamer, ^{1}H NMR (400 MHz, $CDCl_3$) δ 7.25 – 7.23 (m, 2H), 7.18 – 7.14

(m, 2H), 7.10 (s, 1H), 7.06 (d, J = 8.1 Hz, 1H), 6.64 (s, 1H), 2.32 – 2.28 (m, 9H), 2.02 (s, 3H). ^{13}C NMR (75 MHz, $CDCl_3$) δ 169.3 (C_q), 139.3 (C_q), 137.1 (C_q), 136.7 (C_q), 136.6 (C_q), 135.8 (C_q), 132.6 (C_q), 130.1 (CH), 129.9 (CH), 129.6 (CH), 127.9 (CH), 127.3 (CH), 126.3 (CH), 23.1 (CH_3), 19.8 (CH_3), 19.5 (CH_3), 18.7 (CH_3). IR (ATR): 3278, 3019, 2922, 1655, 1522, 1508, 1465, 1368, 1289, 1247 cm^{-1}. MS (ESI) *m*/*z* (relative intensity): 276 (100) [M + Na]$^+$, 254 (100) [M + H]$^+$. HR-MS (ESI): *m*/*z* calcd. for [$C_{17}H_{19}NO$ + Na]$^+$ 276.1359 found 276.1359.

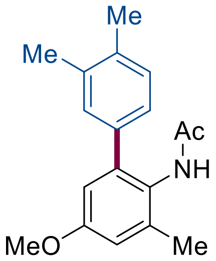

***N*-(5-methoxy-3,3',4'-trimethyl-[1,1'-biphenyl]-2-yl)acetamide (9)**

The general procedure **A** was followed using *N*-(4-methoxy-2-methylphenyl)acetamide (**1g**) (35.8 mg, 0.20 mmol) and *o*-xylene (**2a**) (120.6 µL, 1.0 mmol, 5.0 equiv.) and *n*Bu_4NBF_4 (40 mg, 0.12 mmol, 0.61 equiv.) at 100 °C for 20 h. Isolation by column chromatography (*n*-hexane/EtOAc = 2:1) yielded **9** (30.1 mg, 53%). Rotamers in a ratio of 5:1 could be found on ^{1}H NMR and ^{13}C NMR. For the major rotamer, ^{1}H NMR (400 MHz, $CDCl_3$) δ 7.16 (d, J = 7.8 Hz, 1H), 7.10 (s, 1H), 7.06 (d, J = 7.5 Hz, 1H), 6.79 (s, 1H), 6.70 (s, 1H), 6.48 (s, 1H), 3.80 (s, 3H), 2.32 – 2.25 (m, 9H), 2.01 (s, 3H). ^{13}C NMR (75 MHz, $CDCl_3$) δ 169.8 (C_q), 158.3 (C_q), 140.8 (C_q), 138.2 (C_q), 137.2 (C_q), 136.6 (C_q), 135.9 (C_q), 130.0 (CH), 129.6 (CH), 126.1 (CH), 125.6 (C_q), 115.2 (CH), 113.1 (CH), 55.4 (CH_3), 23.0 (CH_3), 19.8 (CH_3), 19.5 (CH_3), 18.9 (CH_3). IR (ATR): 2994, 1770, 1759, 1654, 1600, 1457, 1374, 1245, 1206, 1055 cm^{-1}. MS (ESI) *m*/*z* (relative intensity): 306 (90) [M + Na]$^+$, 284 (100) [M + H]$^+$. HR-MS (ESI): *m*/*z* calcd. for [$C_{18}H_{21}NO_2$ + H]$^+$ 284.1645 found 284.1645.

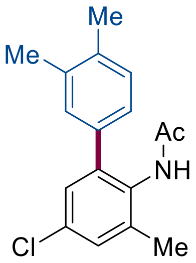

***N*-(5-chloro-3,3',4'-trimethyl-[1,1'-biphenyl]-2-yl)acetamide (10)**

The general procedure **A** was followed using *N*-(4-chloro-2-methylphenyl)acetamide (**1h**) (36.7 mg, 0.20 mmol) and *o*-xylene (**2a**) (120.6 µL, 1.0 mmol, 5.0 equiv.) and *n*Bu_4NBF_4 (200 mg,

0.61 mmol, 3.0 equiv.) at 100 °C for 20 h. Isolation by column chromatography (*n*-hexane/EtOAc = 2:1) yielded **10** (mono-arylated product 39.0 mg, 68% and di-arylated product 6.2 mg, 8%). Rotamers in a ratio of 10:1 could be found on ^{1}H NMR and ^{13}C NMR of mono-arylated product. For the major rotamer, ^{1}H NMR (300 MHz, $CDCl_3$) δ 7.22 (d, *J* = 2.4 Hz, 1H), 7.18 – 7.13 (m, 2H), 7.06 (d, *J* = 1.9 Hz, 1H), 7.02 (dd, *J* = 7.7, 2.0 Hz, 1H), 6.61 (s, 1H), 2.31 – 2.25 (m, 9H), 2.00 (s, 3H). ^{13}C NMR (75 MHz, $CDCl_3$) δ 169.4 (C_q), 140.8 (C_q), 138.6 (C_q), 136.9 (C_q), 136.4 (C_q), 135.8 (C_q), 132.6 (C_q), 131.3 (C_q), 129.9 (CH), 129.7 (CH), 129.6 (CH), 127.7 (CH), 126.1 (CH), 23.0 (CH_3), 19.8 (CH_3), 19.5 (CH_3), 18.7 (CH_3). IR (ATR): 3241, 3017, 2971, 2921, 1655, 1578, 1522, 1464, 1369, 1289 cm^{-1}. MS (ESI) *m*/*z* (relative intensity): 310 (50) $[M + Na]^+$, 288 (100) $[M + H]^+$. HR-MS (ESI): *m*/*z* calcd. for $[C_{17}H_{18}ClNO + H]^+$ 288.1150 found 288.1150.

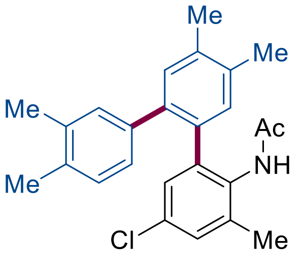

***N*-(5-chloro-3,3'',4',4'',5'-pentamethyl-[1,1':2',1''-terphenyl]-2-yl)acetamide (di-10)**

^{1}H NMR (300 MHz, $CDCl_3$) δ 7.23 (d, *J* = 2.5 Hz, 1H), 7.19 (s, 1H), 7.15 (d, *J* = 2.4 Hz, 1H), 7.06 (s, 1H), 7.01 – 6.96 (m, 2H), 6.80 (dd, *J* = 7.8, 2.1 Hz, 1H), 6.02 (s, 1H), 2.33 (s, 3H), 2.30 (s, 3H), 2.24 (s, 3H), 2.19 (s, 3H), 2.05 (s, 3H), 1.76 (s, 3H). ^{13}C NMR (75 MHz, $CDCl_3$) δ 168.1 (C_q), 141.1 (C_q), 138.4 (C_q), 138.2 (C_q), 137.5 (C_q), 136.9 (C_q), 136.9 (C_q), 136.0 (C_q), 135.7 (C_q), 134.1 (C_q), 132.5 (CH), 132.5 (C_q), 131.3 (C_q), 130.8 (CH), 129.8 (CH), 129.8 (CH), 129.4 (CH), 128.7 (CH), 126.2 (CH), 23.0 (CH_3), 19.7 (CH_3), 19.5 (CH_3), 19.4 (CH_3), 19.4 (CH_3), 18.5 (CH_3). IR (ATR): 3277, 2972, 2926, 2859, 1660, 1578, 1519, 1492, 1451, 1368 cm^{-1}. MS (ESI) *m*/*z* (relative intensity): 414 (100) $[M + Na]^+$, 392 (15) $[M + H]^+$. HR-MS (ESI): *m*/*z* calcd. for $[C_{25}H_{26}ClNO + Na]^+$ 414.1595 found 414.1594.

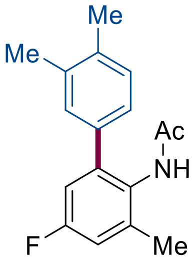

***N*-(5-fluoro-3,3',4'-trimethyl-[1,1'-biphenyl]-2-yl)acetamide (11)**

The general procedure **A** was followed using *N*-(4-fluoro-2-methylphenyl)acetamide (**1i**) (33.4 mg, 0.20 mmol) and *o*-xylene (**2a**) (120.6 µL, 1.0 mmol, 5.0 equiv.) and nBu_4NBF_4 (200 mg, 0.61 mmol, 3.0 equiv.) at 100 °C for 20 h. Isolation by column chromatography (*n*-hexane/EtOAc = 2:1) yielded **11** (mono-arylated product 36.1 mg, 66% and di-arylated product 9.0 mg, 12%). Rotamers in a ratio of 7:1 could be found on ^{1}H NMR and ^{13}C NMR of mono arylated product. For the major rotamer, ^{1}H NMR (500 MHz, $CDCl_3$) δ 7.16 (d, *J* = 7.6 Hz, 1H), 7.07 (d, *J* = 2.0 Hz, 1H), 7.03 (dd, *J* = 7.7, 2.1 Hz, 1H), 6.93 (dd, *J* = 9.0, 2.9 Hz, 1H), 6.87 (dd, *J* = 8.9, 3.0 Hz, 1H), 6.61 (s, 1H), 2.30 (s, 3H), 2.29 (s, 3H), 2.27 (s, 3H), 2.00 (s, 3H). ^{13}C NMR (126 MHz, $CDCl_3$) δ 169.6 (C_q), 161.1 (d, *J* = 246.3 Hz, C_q), 141.3 (d, *J* = 8.8 Hz, C_q), 139.2 (d, *J* = 8.7 Hz, C_q), 136.7 (C_q), 136.3 (C_q), 136.1 (d, *J* = 1.9 Hz, C_q), 129.8 (CH), 129.7 (CH), 128.6 (d, *J* = 3.0 Hz, C_q), 126.0 (CH), 116.2 (d, *J* = 22.0 Hz, CH), 114.4 (d, *J* = 22.4 Hz, CH), 22.9 (CH_3), 19.7 (CH_3), 19.5 (CH_3), 18.8 (CH_3). ^{19}F NMR (471 MHz, $CDCl_3$) δ -115.5 (t, *J* = 8.9 Hz). IR (ATR): 3256, 3031, 2924, 1655, 1597, 1524, 1470, 1370, 1236, 1152 cm^{-1}. MS (ESI) *m*/*z* (relative intensity): 294 (60) $[M + Na]^+$, 272 (100) $[M + H]^+$. HR-MS (ESI): *m*/*z* calcd. for $[C_{17}H_{18}FNO + H]^+$ 272.1445 found 272.1440.

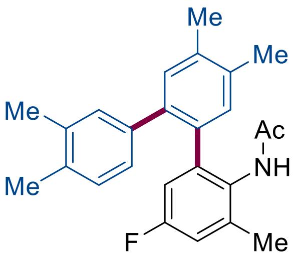

***N*-(5-fluoro-3,3'',4',4'',5'-pentamethyl-[1,1':2',1''-terphenyl]-2-yl)acetamide (di-11)**

Di-arylated product, ^{1}H NMR (300 MHz, $CDCl_3$) δ 7.19 (s, 1H), 7.07 (s, 1H), 7.01 – 6.95 (m, 2H), 6.93 (dd, *J* = 9.0, 3.0 Hz, 1H), 6.87 (dd, *J* = 9.1, 2.9 Hz, 1H), 6.82 (dd, *J* = 7.8, 2.0 Hz, 1H), 5.99 (s, 1H), 2.33 (s, 3H), 2.30 (s, 3H), 2.23 (s, 3H), 2.18 (s, 3H), 2.07 (s, 3H), 1.77 (s, 3H). ^{13}C NMR (75 MHz, $CDCl_3$) δ 168.3 (C_q), 161.1 (d, *J* = 245.6 Hz, C_q), 141.6 (d, *J* = 8.7 Hz, C_q), 138.8 (d, *J* = 9.0 Hz, C_q), 138.5 (C_q), 137.5 (C_q), 136.9 (C_q), 136.8 (C_q), 135.9 (C_q), 135.6 (C_q), 134.4 (d, *J* = 1.6 Hz, C_q), 132.4 (CH), 130.8 (CH), 129.8 (CH), 129.8 (CH), 128.7 (d, *J* = 2.7 Hz, C_q), 126.2 (CH), 116.0 (d, *J* = 21.9 Hz, CH), 115.4 (d, *J* = 22.3 Hz, CH), 23.0 (CH_3), 19.7 (CH_3), 19.5 (CH_3), 19.4 (CH_3), 19.4 (CH_3), 18.7 (d, *J* = 1.6 Hz, CH_3). ^{19}F NMR (282 MHz, $CDCl_3$) δ -115.72 (t, *J* = 9.0 Hz). IR (ATR): 3279, 3016, 2922, 2859, 1759, 1656, 1597, 1522, 1468, 1246 cm^{-1}. MS (ESI) *m*/*z* (relative intensity): 398 (100) $[M + Na]^+$, 376 (20) $[M + H]^+$. HR-MS (ESI): *m*/*z* calcd. for $[C_{25}H_{26}FNO + Na]^+$ 398.1891 found 391.1887.

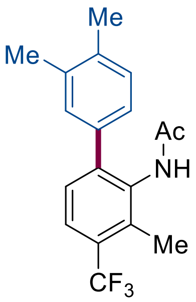

N-(3,3',4'-trimethyl-4-(trifluoromethyl)-[1,1'-biphenyl]-2-yl)acetamide (12)

The general procedure **A** was followed using *N*-(2-methyl-3-(trifluoromethyl)phenyl)acetamide (**1j**) (33.0 mg, 0.20 mmol) and *o*-xylene (**2a**) (120.6 µL, 1.0 mmol, 5.0 equiv.) and *n*Bu_4NBF_4 (200 mg, 0.61 mmol, 3.0 equiv.) at 100 °C for 18 h. Isolation by column chromatography (*n*-hexane/EtOAc = 2:1) yielded **12** (32.5 mg, 51%). Rotamers in a ratio of 10:1 could be found on ^{1}H NMR and ^{13}C NMR. For the major rotamer, ^{1}H NMR (300 MHz, $CDCl_3$) δ 7.62 (d, *J* = 8.1 Hz, 1H), 7.24 (d, *J* = 7.5 Hz, 1H), 7.19 (d, *J* = 7.7 Hz, 1H), 7.07 (d, *J* = 1.8 Hz, 1H), 7.03 (dd, *J* = 7.7, 1.9 Hz, 1H), 6.64 (s, 1H), 2.39 (s, 3H), 2.31 (s, 3H), 2.30 (s, 3H), 2.02 (s, 3H). ^{13}C NMR (101 MHz, $CDCl_3$) δ 169.5 (C_q), 143.1 (C_q), 137.0 (C_q), 136.7 (C_q), 136.1 (C_q), 136.0 (q, *J* = 1.5 Hz, C_q), 134.4 (C_q), 129.6 (CH), 129.1 (q, *J* = 29.9 Hz, C_q), 126.0 (CH), 125.7 (CH), 124.9 (q, *J* = 5.8 Hz, CH), 124.3 (q, *J* = 273.6 Hz, C_q), 23.1 (CH_3), 19.8 (CH_3), 19.6 (CH_3), 14.6 (q, *J* = 2.5 Hz, CH_3). ^{19}F NMR (282 MHz, $CDCl_3$) δ -60.8. IR (ATR): 3238, 3018, 2972, 2925, 1657, 1524, 1424, 1319, 1174, 1118 cm^{-1}. MS (ESI) *m/z* (relative intensity): 344 (70) $[M + Na]^+$, 322 (100) $[M + H]^+$. HR-MS (ESI): *m/z* calcd. for $[C_{18}H_{18}F_3NO + H]^+$ 322.1413 found 322.1412.

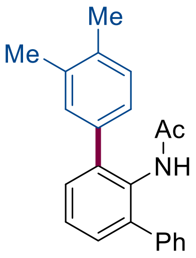

N-(3,4-dimethyl-[1,1':3',1''-terphenyl]-2'-yl)acetamide (13)

The general procedure **A** was followed using *N*-([1,1'-biphenyl]-2-yl)acetamide (**1k**) (42.3 mg, 0.20 mmol) and *o*-xylene (**2a**) (120.6 µL, 1.0 mmol, 5.0 equiv.) and *n*Bu_4NBF_4 (200 mg, 0.61 mmol, 3.0 equiv.) at 100 °C for 20 h. Isolation by column chromatography (*n*-hexane/EtOAc = 10:1) yielded **13** (mono-arylated product 36.0 mg, 57% and di-arylated product 14.0 mg, 17%). Rotamers in a ratio of 3:1 could be found on ^{1}H NMR and ^{13}C NMR of mono-arylated product. For the major rotamer, ^{1}H NMR (300 MHz, $CDCl_3$) δ 7.49 – 7.29 (m, 8H), 7.22 – 7.11 (m, 3H), 6.54 (s, 1H), 2.30 (s, 6H), 1.72 (s, 3H). ^{13}C NMR (75 MHz, $CDCl_3$) δ 169.3 (C_q), 140.9 (C_q),

140.7 (C_q), 140.0 (C_q), 137.2 (C_q), 136.5 (C_q), 135.7 (C_q), 131.2 (C_q), 130.0 (CH), 130.0 (CH), 129.7 (CH), 129.5 (CH), 128.8 (CH), 128.2 (CH), 127.7 (CH), 127.2 (CH), 126.1 (CH), 23.0 (CH_3), 19.8 (CH_3), 19.5 (CH_3). Di-arylated product is a mixture of isomer, pure NMR spectrum were not able to obtained. IR (ATR): 3237, 3019, 2971, 2920, 1769, 1659, 1495, 1452, 1374, 1285 cm^{-1}. MS (ESI) *m/z* (relative intensity): 338 (100) $[M + Na]^+$, 316 (10) $[M + H]^+$. HR-MS (ESI): *m/z* calcd. for $[C_{22}H_{21}NO + Na]^+$ 338.1515 found 338.1513.

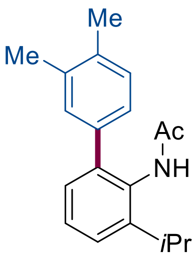

N-(3-isopropyl-3',4'-dimethyl-[1,1'-biphenyl]-2-yl)acetamide (14)

The general procedure **A** was followed using *N*-(2-isopropylphenyl)acetamide (**1l**) (35.4 mg, 0.20 mmol) and *o*-xylene (**2a**) (120.6 µL, 1.0 mmol, 5.0 equiv.) and nBu_4NBF_4 (200 mg, 0.61 mmol, 3.0 equiv.) at 100 °C for 20 h. Isolation by column chromatography (*n*-hexane/EtOAc = 10:1) yielded **14** (mono-arylated product 42.0 mg, 75% and di-arylated product 4%). Rotamers in a ratio of 3.6:1 could be found on ^{1}H NMR and ^{13}C NMR. For the major rotamer, ^{1}H NMR (300 MHz, $CDCl_3$) δ 7.37 – 7.32 (m, 2H), 7.20 – 7.13 (m, 2H), 7.13 – 7.06 (m, 2H), 6.53 (s, 1H), 3.13 (p, *J* = 6.9 Hz, 1H), 2.30 (s, 3H), 2.29 (s, 3H), 1.99 (s, 3H), 1.26 (s, 3H), 1.24 (s, 3H). ^{13}C NMR (75 MHz, $CDCl_3$) δ 170.1 (C_q), 147.1 (C_q), 140.0 (C_q), 137.5 (C_q), 136.5 (C_q), 135.7 (C_q), 131.2 (C_q), 130.2 (CH), 129.5 (CH), 128.0 (CH), 127.9 (CH), 126.2 (CH), 125.4 (CH), 28.7 (CH_3), 23.7 (CH_3), 23.1 (CH), 19.8 (CH_3), 19.5 (CH_3). NMR spectrum for di-arylated product were not able to obtained. IR (ATR): 3235, 2961, 2926, 1770, 1759, 1653, 1523, 1456, 1366, 1246 cm^{-1}. MS (ESI) *m/z* (relative intensity): 304 (100) $[M + Na]^+$, 282 (40) $[M + H]^+$. HR-MS (ESI): *m/z* calcd. for $[C_{19}H_{23}NO + Na]^+$ 304.1672 found 304.1682.

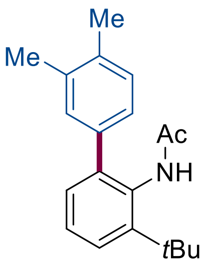

N-(3-(*tert*-butyl)-3',4'-dimethyl-[1,1'-biphenyl]-2-yl)acetamide (15)

The general procedure **A** was followed using *N*-(2-(*tert*-butyl)phenyl)acetamide (**1m**) (38.2 mg, 0.20 mmol) and *o*-xylene (**2a**) (120.6 µL, 1.0 mmol, 5.0 equiv.) and *n*Bu_4NBF_4 (40 mg, 0.12 mmol, 0.61 equiv.) at 100 °C for 18 h. Isolation by column chromatography (*n*-hexane/EtOAc = 10:1) yielded **15** (32.1 mg, 54%). Rotamers in a ratio of 1.4:1 could be found on ^{1}H NMR and ^{13}C NMR. ^{1}H NMR (300 MHz, $CDCl_3$) δ 7.50 – 7.41 (m, 1H), 7.39 – 7.03 (m, 5H), 6.96 (s, 1H), 6.71 (s, 1H), 2.33 – 2.23 (m, 6H), 1.79 (s, 3H), 1.48 – 1.40 (m, 9H+3H). ^{13}C NMR (75 MHz, $CDCl_3$) δ 172.6 (C_q), 169.5 (C_q), 147.9 (C_q), 147.8 (C_q), 143.5 (C_q), 143.2 (C_q), 138.3 (C_q), 137.1 (C_q), 136.6 (C_q), 136.0 (C_q), 135.7 (C_q), 135.1 (C_q), 133.5 (C_q), 132.7 (C_q), 130.8 (CH), 130.2 (CH), 129.8 (CH), 129.5 (CH), 129.2 (CH), 129.0 (CH), 128.4 (CH), 127.9 (CH), 127.1 (CH), 126.3 (CH), 126.3 (CH), 126.2 (CH), 35.7 (C_q), 35.5 (C_q), 31.2 (CH_3), 30.9 (CH_3), 23.5 (CH_3), 20.7 (CH_3), 19.9 (CH_3), 19.8 (CH_3), 19.5 (CH_3), 19.5 (CH_3). IR (ATR): 3280, 2992, 2962, 1770, 1758, 1660, 1518, 1366, 1245, 1059 cm^{-1}. MS (ESI) *m*/*z* (relative intensity): 318 (70) $[M + Na]^+$, 296 (100) $[M + H]^+$. HR-MS (ESI): *m*/*z* calcd. for $[C_{20}H_{25}NO + H]^+$ 296.2009 found 296.2002.

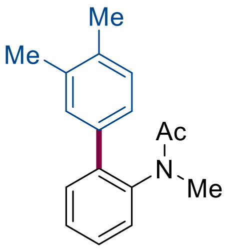

***N*-(3',4'-dimethyl-[1,1'-biphenyl]-2-yl)-*N*-methylacetamide (16)**

The general procedure **A** was followed using *N*-methyl-*N*-phenylacetamide (**1n**) (29.8 mg, 0.20 mmol) and *o*-xylene (**2a**) (120.6 µL, 1.0 mmol, 5.0 equiv.) and *n*Bu_4NBF_4 (40 mg, 0.12 mmol, 0.61 equiv.) at 100 °C for 20 h. Isolation by column chromatography (*n*-hexane/EtOAc = 2:1) yielded **16** (36.1 mg, 71%). The product is known and the characterization is in consistence with that reported in the literature.[16] ^{1}H NMR (400 MHz, $CDCl_3$) δ 7.42 – 7.34 (m, 3H), 7.22 (d, J = 7.1 Hz, 1H), 7.16 (d, J = 7.7 Hz, 1H), 7.05 (s, 1H), 7.01 (d, J = 7.8 Hz, 1H), 3.03 (s, 3H), 2.29 (s, 6H), 1.79 (s, 3H). ^{13}C NMR (101 MHz, $CDCl_3$) δ 170.7 (C_q), 142.0 (C_q), 139.9 (C_q), 136.9 (C_q), 136.2 (C_q), 131.5 (CH), 130.0 (CH), 129.6 (CH), 128.5 (CH), 128.4 (CH), 128.3 (CH), 125.7 (CH), 37.1 (CH_3), 22.4 (CH_3), 20.0 (CH_3), 19.5 (CH_3). IR (ATR): 3020, 2968, 2921, 1659, 1484, 1444, 1375, 1349, 1299, 761 cm^{-1}. MS (ESI) *m*/*z* (relative intensity): 276 (90) $[M + Na]^+$, 254 (100) $[M + H]^+$. HR-MS (ESI): *m*/*z* calcd. for $[C_{17}H_{19}NO + H]^+$ 254.1539 found 254.1538.

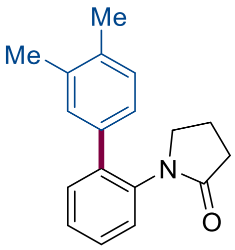

1-(3',4'-Dimethyl-[1,1'-biphenyl]-2-yl)pyrrolidin-2-one (17)

The general procedure **A** was followed using 1-phenylpyrrolidin-2-one (**1o**) (32.2 mg, 0.20 mmol) and *o*-xylene (**2a**) (1.0 mL, 8.3 mmol) and nBu$_4$NBF$_4$ (40 mg, 0.12 mmol, 0.61 equiv.) at 100 °C for 20 h. Isolation by column chromatography (*n*-hexane/EtOAc = 1:1) yielded **17** (27.0 mg, 51%). The product is known and the characterization is in consistence with that reported in the literature.[4] ^{1}H NMR (300 MHz, CDCl$_3$) δ 7.42 – 7.27 (m, 4H), 7.18 – 7.13 (m, 2H), 7.11 (dd, J = 7.8, 1.7 Hz, 1H), 3.23 (t, J = 6.9 Hz, 2H), 2.43 (t, J = 8.0 Hz, 2H), 2.30 (s, 3H), 2.29 (s, 3H), 1.88 (tt, J = 7.8, 6.8 Hz, 2H). ^{13}C NMR (101 MHz, CDCl$_3$) δ 175.6 (C$_q$), 139.5 (C$_q$), 136.6 (C$_q$), 136.6 (C$_q$), 136.1 (C$_q$), 135.9 (C$_q$), 130.9 (CH), 129.7 (CH), 129.4 (CH), 128.3 (CH), 128.2 (CH), 127.9 (CH), 125.6 (CH), 50.0 (CH$_2$), 31.2 (CH$_2$), 19.7 (CH$_3$), 19.5 (CH$_3$), 19.0 (CH$_2$). IR (ATR): 2972, 2920, 2877, 1694, 1485, 1446, 1406, 1301, 1231, 759 cm^{-1}. MS (ESI) *m*/*z* (relative intensity): 288 (90) [M + Na]$^+$, 266 (100) [M + H]$^+$. HR-MS (ESI): *m*/*z* calcd. for [C$_{18}$H$_{19}$NO + H]$^+$ 266.1539 found 266.1543.

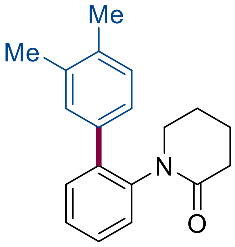

(1-(3',4'-Dimethyl-[1,1'-biphenyl]-2-yl)piperidin-2-one (18)

The general procedure **A** was followed using 1-phenylpiperidin-2-one (**1p**) (35.0 mg, 0.20 mmol) and *o*-xylene (**2a**) (1.0 mL, 8.3 mmol) and nBu$_4$NBF$_4$ (200 mg, 0.61 mmol, 3.0 equiv.) at 100 °C for 20 h. Isolation by column chromatography (*n*-hexane/EtOAc = 1:1) yielded **18** (50.8 mg, 92%). ^{1}H NMR (300 MHz, CDCl$_3$) δ 7.33 – 7.22 (m, 3H), 7.17 – 7.12 (m, 1H), 7.08 – 6.99 (m, 3H), 3.27 – 3.10 (m, 1H), 2.97 – 2.81 (m, 1H), 2.48 – 2.25 (m, 2H), 2.19 (s, 6H), 1.79 – 1.48 (m, 3H), 1.38 – 1.22 (m, 1H). ^{13}C NMR (75 MHz, CDCl$_3$) δ 170.9 (C$_q$), 140.8 (C$_q$), 139.5 (C$_q$), 136.5 (C$_q$), 136.4 (C$_q$), 135.8 (C$_q$), 131.0 (CH), 129.7 (CH), 129.5 (CH), 128.7 (CH), 128.3 (CH), 127.9 (CH), 125.9 (CH), 51.0 (CH$_2$), 32.7 (CH$_2$), 23.2 (CH$_2$), 21.2 (CH$_2$), 19.8 (CH$_3$), 19.5 (CH$_3$). IR (ATR): 3058, 2943, 2865, 1649, 1484, 1444, 1413, 1346,

1166, 759 cm^{-1}. MS (ESI) *m/z* (relative intensity): 302 (60) [M + Na]$^+$, 280 (100) [M + H]$^+$. HR-MS (ESI): *m/z* calcd. for [$C_{19}H_{21}NO$ + H]$^+$ 280.1696 found 280.1700.

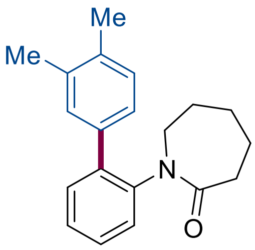

1-(3',4'-Dimethyl-[1,1'-biphenyl]-2-yl)azepan-2-one (19)

The general procedure **A** was followed using 1-phenylazepan-2-one (**1q**) (37.9 mg, 0.20 mmol) and *o*-xylene (**2a**) (1.0 mL, 8.3 mmol) and *n*Bu_4NBF_4 (200 mg, 0.61 mmol, 3.0 equiv.) at 100 °C for 20 h. Isolation by column chromatography (*n*-hexane/EtOAc = 1:1) yielded **19** (37.0 mg, 63%). ^{1}H NMR (300 MHz, $CDCl_3$) δ 7.42 – 7.30 (m, 3H), 7.23 – 7.18 (m, 1H), 7.17 – 7.08 (m, 3H), 3.26 – 3.08 (m, 2H), 2.68 – 2.46 (m, 2H), 2.30 (s, 3H), 2.29 (s, 3H), 1.86 – 1.65 (m, 3H), 1.57 – 1.42 (m, 2H), 1.36 – 1.21 (m, 1H). ^{13}C NMR (75 MHz, $CDCl_3$) δ 176.9 (C_q), 142.5 (C_q), 139.2 (C_q), 137.0 (C_q), 136.5 (C_q), 135.8 (C_q), 131.1 (CH), 129.9 (CH), 129.6 (CH), 128.8 (CH), 128.1 (CH), 127.6 (CH), 126.1 (CH), 52.9 (CH_2), 37.5 (CH_2), 29.9 (CH_2), 27.8 (CH_2), 23.2 (CH_2), 19.8 (CH_3), 19.6 (CH_3). IR (ATR): 3019, 2925, 2856, 1653, 1481, 1467, 1443, 1411, 1200, 758 cm^{-1}. MS (ESI) *m/z* (relative intensity): 316 (40) [M + Na]$^+$, 294 (100) [M + H]$^+$. HR-MS (ESI): *m/z* calcd. for [$C_{20}H_{23}NO$ + H]$^+$ 294.1852 found 294.1846.

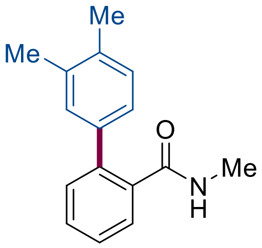

***N*,3',4'-trimethyl-[1,1'-biphenyl]-2-carboxamide (20)**

The general procedure **A** was followed using *N*-methylbenzamide (**1r**) (27.0 mg, 0.20 mmol) and *o*-xylene (**2a**) (1.0 mL, 8.3 mmol), $Pd(OAc)_2$ (9.2 mg, 20 mol %) and *n*Bu_4NBF_4 (200 mg, 0.61 mmol, 3.0 equiv.) at 100 °C for 20 h. Isolation by column chromatography (*n*-hexane/EtOAc = 5:1) yielded **20** (mono-arylated product 20.5 mg, 43% and di-arylated product 3.5 mg, 5%). The product is known and the characterization is in consistence with that reported in the literature.[19] ^{1}H NMR (500 MHz, $CDCl_3$) δ 7.70 (ddd, *J* = 7.6, 1.5, 0.5 Hz, 1H), 7.45 (td, *J* = 7.5, 1.5 Hz, 1H), 7.38 (td, *J* = 7.5, 1.4 Hz, 1H), 7.35 (ddd, *J* = 7.6, 1.4, 0.5 Hz, 1H), 7.20 – 7.16 (m, 2H), 7.13 (dd, *J* = 7.6, 1.9 Hz, 1H), 5.24 (s, 1H), 2.69 (d, *J* = 4.9 Hz, 3H), 2.30 (s, 6H).

^{13}C NMR (126 MHz, $CDCl_3$) δ 170.4 (C_q), 139.4 (C_q), 137.6 (C_q), 136.9 (C_q), 136.2 (C_q), 135.3 (C_q), 130.1 (CH), 130.1 (CH), 129.8 (CH), 129.8 (CH), 128.9 (CH), 127.3 (CH), 126.0 (CH), 26.7 (CH_3), 19.8 (CH_3), 19.5 (CH_3). NMR spectrum for di-arylated product were not able to obtained. IR (ATR): 3258, 2914, 1770, 1759, 1638, 1519, 1487, 1245, 1052, 793 cm^{-1}. MS (ESI) *m*/*z* (relative intensity): 262 (100) $[M + Na]^+$, 240 (80) $[M + H]^+$. HR-MS (ESI): *m*/*z* calcd. for $[C_{16}H_{17}NO + Na]^+$ 262.1202 found 262.1210.

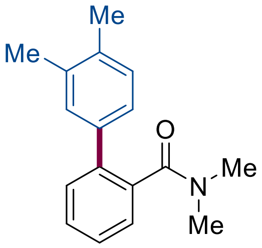

N,N,3',4'-tetramethyl-[1,1'-biphenyl]-2-carboxamide (21)

The general procedure **A** was followed using *N*,*N*-dimethylbenzamide (**1s**) (29.8 mg, 0.20 mmol) and *o*-xylene (**2a**) (1.0 mL, 8.3 mmol) and *n*Bu_4NBF_4 (200 mg, 0.61 mmol, 3.0 equiv.) at 100 °C for 20 h. Isolation by column chromatography (*n*-hexane/EtOAc = 5:1) yielded **21** (8.2 mg, 16%). ^1H NMR (400 MHz, $CDCl_3$) δ 7.47 – 7.32 (m, 4H), 7.23 (s, 1H), 7.19 (d, *J* = 8.0 Hz, 1H), 7.14 (d, *J* = 7.8 Hz, 1H), 2.87 (d, *J* = 1.5 Hz, 3H), 2.43 (d, *J* = 1.5 Hz, 3H), 2.29 (s, 6H). ^{13}C NMR (101 MHz, $CDCl_3$) δ 171.7 (C_q), 138.9 (C_q), 137.6 (C_q), 136.6 (C_q), 136.0 (C_q), 135.6 (C_q), 129.7 (CH), 129.6 (CH), 129.4 (CH), 129.3 (CH), 127.4 (CH), 127.4 (CH), 125.9 (CH), 38.1 (CH_3), 34.7 (CH_3), 19.8 (CH_3), 19.5 (CH_3). IR (ATR): 3019, 2936, 2879, 1769, 1758, 1632, 1507, 1393, 1246, 1051 cm^{-1}. MS (ESI) *m*/*z* (relative intensity): 276 (100) $[M + Na]^+$, 254 (60) $[M + H]^+$. HR-MS (ESI): *m*/*z* calcd. for $[C_{17}H_{19}NO + Na]^+$ 276.1359 found 276.1363.

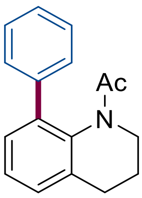

1-(8-Phenyl-3,4-dihydroquinolin-1(2*H*)-yl)ethan-1-one (22)

The general procedure **A** was followed using 1-(3,4-dihydroquinolin-1(2*H*)-yl)ethan-1-one (**1a**) (31.7 µL, 0.20 mmol) and benzene (**2b**) (1.0 mL, 11.2 mmol) and *n*Bu_4NBF_4 (200 mg, 0.61 mmol, 3.0 equiv.) at 90 °C for 20 h. Isolation by column chromatography (*n*-hexane/EtOAc = 2:1) yielded **22** (39.0 mg, 78%). The product is known and the characterization is in consistence with that reported in the literature.[16] ^1H NMR (300 MHz, $CDCl_3$) δ 7.46 – 7.26 (m, 7H), 7.22 –

7.18 (m, 1H), 4.86 – 4.72 (m, 1H), 3.14 – 3.00 (m, 1H), 2.79 – 2.70 (m, 1H), 2.57 – 2.44 (m, 1H), 2.41 – 2.26 (m, 1H), 1.87 – 1.68 (m, 1H), 1.44 (s, 3H). ^{13}C NMR (75 MHz, $CDCl_3$) δ 170.2 (C_q), 139.1 (C_q), 138.2 (C_q), 137.7 (C_q), 137.7 (C_q), 129.0 (CH), 128.8 (CH), 128.3 (CH), 127.5 (CH), 126.9 (CH), 126.9 (CH), 41.7 (CH_2), 26.9 (CH_2), 24.4 (CH_2), 21.9 (CH_3). IR (ATR): 2948, 2877, 1770, 1759, 1656, 1463, 1430, 1375, 1246, 759 cm^{-1}. MS (ESI) *m/z* (relative intensity): 274 (100) $[M + Na]^+$, 252 (10) $[M + H]^+$. HR-MS (ESI): *m/z* calcd. for $[C_{17}H_{17}NO + Na]^+$ 274.1202 found 274.1204.

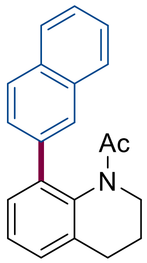

1-(8-(Naphthalen-2-yl)-3,4-dihydroquinolin-1(*2H*)-yl)ethan-1-one (23)

The general procedure **A** was followed using 1-(3,4-dihydroquinolin-1(*2H*)-yl)ethan-1-one (**1a**) (31.7 µL, 0.20 mmol) and naphthalene (**2c**) (77.0 mg, 0.60 mmol, 3.0 equiv.) and nBu$_4$NBF$_4$ (40 mg, 0.12 mmol, 0.61 equiv.) at 100 °C for 20 h. Isolation by column chromatography (*n*-hexane/EtOAc = 2:1) yielded **23** (38.5 mg, 64%). Isomers could not be separated and identified. ^{1}H NMR (400 MHz, $CDCl_3$) δ 8.07 – 7.79 (m, 3H), 7.57 – 7.39 (m, 4H), 7.39 – 7.28 (m, 2H), 7.25 – 7.19 (m, 1H), 4.92 – 4.34 (m, 1H), 3.31 – 2.96 (m, 1H), 2.86 – 2.71 (m, 1H), 2.61 – 2.47 (m, 1H), 2.44 – 2.28 (m, 1H), 1.90 – 1.77 (m, 1H), 1.60 – 1.28 (m, 3H). ^{13}C NMR (101 MHz, $CDCl_3$) δ 170.7 (C_q), 170.7 (C_q), 170.3 (C_q), 139.3 (C_q), 139.2 (C_q), 138.6 (C_q), 138.3 (C_q), 138.0 (C_q), 137.8 (C_q), 137.7 (C_q), 137.7 (C_q), 136.9 (C_q), 136.8 (C_q), 135.9 (C_q), 135.6 (C_q), 134.6 (C_q), 133.8 (C_q), 133.5 (C_q), 132.6 (C_q), 131.1 (C_q), 131.0 (CH), 130.5 (CH), 129.1 (CH), 128.9 (CH), 128.7 (CH), 128.6 (CH), 128.6 (CH), 128.4 (CH), 128.3 (CH), 128.2 (CH), 127.7 (CH), 127.5 (CH), 127.4 (CH), 127.3 (CH), 127.1 (CH), 127.0 (CH), 126.5 (CH), 126.4 (CH), 126.3 (CH), 126.2 (CH), 126.1 (CH), 126.0 (CH), 125.8 (CH), 125.6 (CH), 125.4 (CH), 125.2 (CH), 125.1 (CH), 41.9 (CH_2), 41.8 (CH_2), 41.8 (CH_2), 27.2 (CH_2), 27.1 (CH_2), 26.9 (CH_2), 24.7 (CH_2), 24.5 (CH_2), 24.4 (CH_2), 22.4 (CH_3), 22.1 (CH_3), 22.0 (CH_3). IR (ATR): 2996, 2948, 1769, 1758, 1656, 1456, 1374, 1245, 1060, 795 cm^{-1}. MS (ESI) *m/z* (relative intensity): 324 (100) $[M + Na]^+$, 302 (70) $[M + H]^+$. HR-MS (ESI): *m/z* calcd. for $[C_{21}H_{19}NO + Na]^+$ 324.1359 found 324.1361.

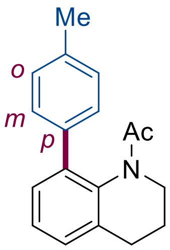

1-(8-(*p*-Tolyl)-3,4-dihydroquinolin-1(2*H*)-yl)ethan-1-one (24)

The general procedure **A** was followed using 1-(3,4-dihydroquinolin-1(2*H*)-yl)ethan-1-one (**1a**) (31.7 µL, 0.20 mmol) and toluene (**2d**) (1.0 mL, 9.4 mmol) and *n*Bu_4NBF_4 (40 mg, 0.12 mmol, 0.61 equiv.) at 100 °C for 18 h. Isolation by column chromatography (*n*-hexane/EtOAc = 2:1) yielded **24** (35.6 mg, 67%). The product is known and the characterization is in consistence with that reported in the literature.[16] The regioselectivity was determined by the analysis of ^{13}C NMR, the ratio of the isomers was determined by the integration of ^{1}H NMR, *m* : *p* = 1.0 : 1.2. ^{1}H NMR (400 MHz, $CDCl_3$) δ 7.44 – 7.15 (m, 7H), 4.94 – 4.76 (m, 1H), 3.21 – 3.06 (m, 1H), 2.88 – 2.73 (m, 1H), 2.64 – 2.49 (m, 1H), 2.47 – 2.32 (m, 4H), 1.94 – 1.74 (m, 1H), 1.51 (s, $3H^{m}$), 1.51 (s, $3H^{p}$). ^{13}C NMR (101 MHz, $CDCl_3$) δ 170.3 (C_q), 170.3 (C_q), 139.0 (C_q), 138.5 (C_q), 138.1 (C_q), 138.1 (C_q), 137.8 (C_q), 137.7 (C_q), 137.6 (C_q), 137.6 (C_q), 137.3 (C_q), 136.1 (C_q), 129.7 (CH), 128.9 (CH), 128.9 (CH), 128.8 (CH), 128.6 (CH), 128.3 (CH), 128.1 (CH), 126.9 (CH), 126.8 (CH), 126.7 (CH), 126.5 (CH), 125.3 (CH), 41.7 (CH_2), 41.7 (CH_2), 26.9 (CH_2), 26.9 (CH_2), 24.4 (CH_2), 24.3 (CH_2), 21.9 (CH_3), 21.6 (CH_3), 21.1 (CH_3). IR (ATR): 2947, 2873, 1770, 1758, 1658, 1456, 1374, 1246, 1060, 779 cm^{-1}. MS (ESI) *m*/*z* (relative intensity): 288 (60) $[M + Na]^+$, 266 (100) $[M + H]^+$. HR-MS (ESI): *m*/*z* calcd. for $[C_{18}H_{19}NO + H]^+$ 266.1539 found 266.1536.

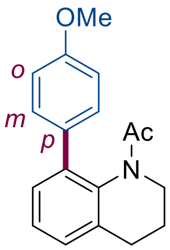

1-(8-(4-Methoxyphenyl)-3,4-dihydroquinolin-1(2*H*)-yl)ethan-1-one (25)

The general procedure **A** was followed using 1-(3,4-dihydroquinolin-1(2*H*)-yl)ethan-1-one (**1a**) (31.7 µL, 0.20 mmol) and anisole (**2e**) (65.2 µL, 0.60 mmol, 3.0 equiv.) and *n*Bu_4NBF_4 (40 mg, 0.12 mmol, 0.61 equiv.) at 100 °C for 18 h. Isolation by column chromatography (*n*-hexane/EtOAc = 2:1) and gel permeation chromatography yielded **25** (37.7 mg, 67%). The product is known and the characterization is in consistence with that reported in the literature.[20]

The regioselectivity was determined by the analysis of ^{13}C NMR, the ratio of the isomers was determined by the integration of ^{1}H NMR, *m* : *p* = 1 : 10. ^{1}H NMR (400 MHz, $CDCl_3$) δ 7.31 – 7.23 (m, 4H), 7.17 – 7.13 (m, 1H), 6.97 – 6.92 (m, 2H), 4.86 – 4.71 (m, 1H), 3.82 (s, 3H), 3.11 – 2.99 (m, 1H), 2.81 – 2.67 (m, 1H), 2.59 – 2.41 (m, 1H), 2.39 – 2.24 (m, 1H), 1.85 – 1.68 (m, 1H), 1.46 (s, 3H). ^{13}C NMR (101 MHz, $CDCl_3$) δ 170.3 (C_q), 159.0 (C_q), 138.1 (C_q), 137.6 (C_q), 137.3 (C_q), 131.3 (C_q), 129.4 (CH), 128.5 (CH), 126.8 (CH), 126.3 (CH), 114.5 (CH), 55.2 (CH_3), 41.6 (CH_2), 26.8 (CH_2), 24.3 (CH_2), 21.8 (CH_3). IR (ATR): 2996, 2947, 1770, 1759, 1655, 1513, 1456, 1375, 1247, 1031 cm^{-1}. MS (ESI) *m*/*z* (relative intensity): 304 (100) $[M + Na]^+$, 282 (90) $[M + H]^+$. HR-MS (ESI): *m*/*z* calcd. for $[C_{18}H_{19}NO_2 + Na]^+$ 304.1308 found 304.1305.

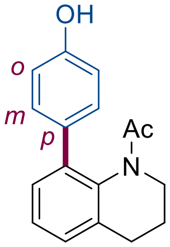

1-(8-(4-Hydroxyphenyl)-3,4-dihydroquinolin-1(*2H*)-yl)ethan-1-one (26)

The general procedure **A** was followed using 1-(3,4-dihydroquinolin-1(*2H*)-yl)ethan-1-one (**1a**) (31.7 µL, 0.20 mmol) and triisopropyl(phenoxy)silane (**2f**) (282.0 µL, 1.0 mmol, 5.0 equiv.) and nBu_4NBF_4 (200 mg, 0.61 mmol, 3.0 equiv.) at 100 °C for 20 h. Isolation by column chromatography (DCM/EtOAc = 2:1) yielded **26** (35.0 mg, 65%). The regioselectivity was determined by the analysis of ^{13}C NMR, the ratio of the isomers was determined by the integration of ^{1}H NMR, *m* : *p* = 1 : 4. ^{1}H NMR (400 MHz, $CDCl_3$) δ 8.59 (s, 1H), 7.33 (d, *J* = 7.4 Hz, 1H), 7.31 – 7.25 (m, 1H), 7.20 (d, *J* = 8.2 Hz, 2H), 7.16 (d, *J* = 7.5 Hz, 1H), 6.84 (d, *J* = 8.3 Hz, 2H), 4.83 – 4.70 (m, 1H), 3.25 – 3.14 (m, 1H), 2.80 – 2.71 (m, 1H), 2.59 – 2.44 (m, 1H), 2.40 – 2.28 (m, 1H), 1.84 – 1.71 (m, 1H), 1.53 (s, 3H). ^{13}C NMR (101 MHz, $CDCl_3$) δ 171.9 (C_q), 156.9 (C_q), 138.1 (C_q), 137.7 (C_q), 136.7 (C_q), 129.8 (C_q), 129.3 (CH), 128.6 (CH), 127.2 (CH), 126.1 (CH), 116.2 (CH), 42.3 (CH_2), 26.9 (CH_2), 24.4 (CH_2), 22.0 (CH_3). IR (ATR): 2990, 2956, 1770, 1758, 1622, 1458, 1385, 1245, 1106, 1059 cm^{-1}. MS (ESI) *m*/*z* (relative intensity): 290 (100) $[M + Na]^+$, 268 (50) $[M + H]^+$. HR-MS (ESI): *m*/*z* calcd. for $[C_{17}H_{17}NO_2 + Na]^+$ 290.1150 found 290.1150.

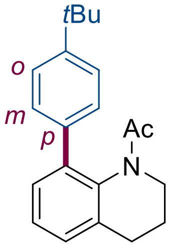

1-(8-(4-(*tert*-Butyl)phenyl)-3,4-dihydroquinolin-1(2*H*)-yl)ethan-1-one (27)

The general procedure **A** was followed using 1-(3,4-dihydroquinolin-1(2*H*)-yl)ethan-1-one (**1a**) (31.7 µL, 0.20 mmol) and *tert*-butylbenzene (**2g**) (153.0 µL, 1.0 mmol, 5.0 equiv.) and *n*Bu_4NBF_4 (200 mg, 0.61 mmol, 3.0 equiv.) at 100 °C for 20 h. Isolation by column chromatography (*n*-hexane/EtOAc = 2:1) yielded **27** (44.5 mg, 72%). The regioselectivity was determined by the analysis of ^{13}C NMR, the ratio of the isomers was determined by the integration of ^{1}H NMR, *m* : *p* = 1.0 : 1.3. ^{1}H NMR (300 MHz, $CDCl_3$) δ 7.43 (d, *J* = 8.4 Hz, 1H), 7.37 – 7.33 (m, 2H), 7.32 – 7.14 (m, 4H), 4.87 – 4.70 (m, 1H), 3.15 – 3.00 (m, 1H), 2.80 – 2.68 (m, 1H), 2.59 – 2.43 (m, 1H), 2.41 – 2.26 (m, 1H), 1.87 – 1.69 (m, 1H), 1.44 (s, $3H^p$), 1.42 (s, $3H^m$), 1.33 (s, $9H^p$), 1.33 (s, $9H^m$). ^{13}C NMR (75 MHz, $CDCl_3$) δ 170.5 (C_q), 170.2 (C_q), 151.8 (C_q), 150.6 (C_q), 138.7 (C_q), 138.3 (C_q), 138.1 (C_q), 137.7 (C_q), 137.7 (C_q), 137.6 (C_q), 136.0 (C_q), 128.8 (CH), 128.7 (CH), 127.9 (CH), 126.9 (CH), 126.9 (CH), 126.7 (CH), 126.6 (CH), 126.0 (CH), 125.6 (CH), 125.4 (CH), 124.4 (CH), 41.7 (CH_2), 41.6 (CH_2), 34.7 (C_q), 34.6 (C_q), 31.3 (CH_3), 31.3 (CH_3), 27.0 (CH_2), 26.9 (CH_2), 24.4 (CH_2), 24.3 (CH_2), 21.8 (CH_3), 21.8 (CH_3). IR (ATR): 2958, 2872, 1770, 1759, 1660, 1456, 1437, 1373, 1246, 1060 cm^{-1}. MS (ESI) *m*/*z* (relative intensity): 330 (70) $[M + Na]^+$, 308 (100) $[M + H]^+$. HR-MS (ESI): *m*/*z* calcd. for $[C_{21}H_{25}NO + H]^+$ 308.2009 found 308.2009.

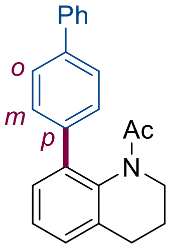

1-(8-([1,1'-Biphenyl]-4-yl)-3,4-dihydroquinolin-1(2*H*)-yl)ethan-1-one (28)

The general procedure **A** was followed using 1-(3,4-dihydroquinolin-1(2*H*)-yl)ethan-1-one (**1a**) (31.7 µL, 0.20 mmol) and 1,1'-biphenyl (**2h**) (154.0 mg, 1.0 mmol, 5.0 equiv.) and *n*Bu_4NBF_4 (200 mg, 0.61 mmol, 3.0 equiv.) at 100 °C for 20 h. Isolation by column chromatography (*n*-hexane/EtOAc = 2:1) yielded **28** (33.5 mg, 51%). The product is known and the characterization is in consistence with that reported in the literature.[16] The regioselectivity was

determined by the analysis of ^{13}C NMR. ^{1}H NMR (300 MHz, $CDCl_3$) δ 7.69 – 7.59 (m, 4H), 7.51 – 7.34 (m, 6H), 7.30 (t, *J* = 7.5 Hz, 1H), 7.21 (d, *J* = 7.2 Hz, 1H), 4.95 – 4.71 (m, 1H), 3.19 – 3.04 (m, 1H), 2.84 – 2.70 (m, 1H), 2.59 – 2.45 (m, 1H), 2.43 – 2.26 (m, 1H), 1.89 – 1.70 (m, 1H), 1.50 (s, 3H). ^{13}C NMR (75 MHz, $CDCl_3$) δ 170.3 (C_q), 140.4 (C_q), 140.3 (C_q), 138.2 (C_q), 138.0 (C_q), 137.8 (C_q), 137.3 (C_q), 128.8 (CH), 128.7 (CH), 128.7 (CH), 127.7 (CH), 127.5 (CH), 127.0 (CH), 127.0 (CH), 126.9 (CH), 41.7 (CH_2), 26.9 (CH_2), 24.4 (CH_2), 22.0 (CH_3). IR (ATR): 3059, 2938, 1770, 1758, 1655, 1457, 1374, 1245, 1061, 764 cm^{-1}. MS (ESI) *m/z* (relative intensity): 350 (90) $[M + Na]^+$, 328 (100) $[M + H]^+$. HR-MS (ESI): *m/z* calcd. for $[C_{23}H_{22}NO + H]^+$ 328.1696 found 328.1696.

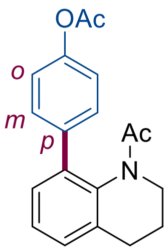

4-(1-acetyl-1,2,3,4-tetrahydroquinolin-8-yl)phenyl acetate (29)

The general procedure **B** was followed using 1-(3,4-dihydroquinolin-1(2*H*)-yl)ethan-1-one (**1a**) (31.7 µL, 0.20 mmol) and phenyl acetate (**2i**) (1.0 mL, 7.9 mmol) and *n*Bu_4NBF_4 (200 mg, 0.61 mmol, 3.0 equiv.) at 90 °C for 20 h. Isolation by column chromatography (*n*-hexane/EtOAc = 1:1) yielded **29** (39.0 mg, 63%). The regioselectivity was determined by the analysis of ^{1}H NMR and ^{13}C NMR, the ratio of the isomers was determined by the integration of ^{1}H NMR spectrum, *m* : *p* = 1 : 4. A phenol derivative derived from *p*-arylated product was observed. For *p*-**29**, ^{1}H NMR (400 MHz, $CDCl_3$) δ 7.52 – 7.01 (m, 7H), 4.87 – 4.71 (m, 1H), 3.20 – 3.00 (m, 1H), 2.81 – 2.67 (m, 1H), 2.57 – 2.44 (m, 1H), 2.39 – 2.26 (m, 4H), 1.86 – 1.70 (m, 1H), 1.46 (s, 3H). ^{13}C NMR (101 MHz, $CDCl_3$) δ 170.3 (C_q), 169.4 (C_q), 150.1 (C_q), 138.2 (C_q), 137.7 (C_q), 136.8 (C_q), 136.7 (C_q), 129.4 (CH), 128.8 (CH), 127.1 (CH), 127.1 (CH), 122.3 (CH), 41.8 (CH_2), 26.9 (CH_2), 24.3 (CH_2), 21.9 (CH_3), 21.2 (CH_3). IR (ATR): 2948, 2877, 1757, 1650, 1458, 1369, 1191, 1015, 910, 728 cm^{-1}. MS (ESI) *m/z* (relative intensity): 332 (100) $[M + Na]^+$, 310 (10) $[M + H]^+$. HR-MS (ESI): *m/z* calcd. for $[C_{19}H_{19}NO_3 + Na]^+$ 332.1257 found 332.1258.

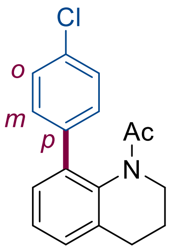

1-(8-(4-Chlorophenyl)-3,4-dihydroquinolin-1(2*H*)-yl)ethan-1-one (30)

The general procedure **B** was followed using 1-(3,4-dihydroquinolin-1(2*H*)-yl)ethan-1-one (**1a**) (31.7 µL, 0.20 mmol) and chlorobenzene (**2j**) (1.0 mL, 9.9 mmol) and *n*Bu_4NBF_4 (40 mg, 0.12 mmol, 0.61 equiv.) at 90 °C for 20 h. Isolation by column chromatography (*n*-hexane/EtOAc = 2:1) yielded **30** (38.4 mg, 67%). The regioselectivity was determined by the analysis of ^{13}C NMR, the ratio of the isomers was determined by the integration of ^1H NMR, *m : p* = 1.0 : 1.2. ^1H NMR (400 MHz, $CDCl_3$) δ 7.44 – 7.18 (m, 7H), 4.86 – 4.69 (m, 1H), 3.09 – 3.00 (m, 1H), 2.80 – 2.71 (m, 1H), 2.56 – 2.44 (m, 1H), 2.40 – 2.29 (m, 1H), 1.84 – 1.69 (m, 1H), 1.48 (s, $3H^m$), 1.46 (s, $3H^p$). ^{13}C NMR (101 MHz, $CDCl_3$) δ 170.1 (C_q), 170.1 (C_q), 141.0 (C_q), 138.4 (C_q), 138.3 (C_q), 137.8 (C_q), 137.7 (C_q), 137.6 (C_q), 136.5 (C_q), 136.4 (C_q), 134.9 (C_q), 133.7 (C_q), 130.3 (CH), 129.6 (CH), 129.3 (CH), 128.7 (CH), 128.6 (CH), 128.5 (CH), 127.7 (CH), 127.5 (CH), 127.3 (CH), 127.1 (CH), 126.5 (CH), 41.8 (CH_2), 26.9 (CH_2), 24.3 (CH_2), 22.0 (CH_3), 21.9 (CH_3). IR (ATR): 2948, 2876, 1769, 1759, 1657, 1457, 1374, 1338, 1247, 782 cm^{-1}. MS (ESI) *m*/*z* (relative intensity): 308 (10) $[M + Na]^+$, 286 (100) $[M + H]^+$. HR-MS (ESI): *m*/*z* calcd. for $[C_{17}H_{16}NO + H]^+$ 286.0993 found 286.0992.

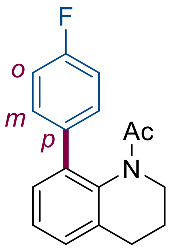

1-(8-(4-Fluorophenyl)-3,4-dihydroquinolin-1(2*H*)-yl)ethan-1-one (31)

The general procedure **B** was followed using 1-(3,4-dihydroquinolin-1(2*H*)-yl)ethan-1-one (**1a**) (31.7 µL, 0.20 mmol) and fluorobenzene (**2k**) (1.0 mL, 10.7 mmol) and *n*Bu_4NBF_4 (200 mg, 0.61 mmol, 3.0 equiv.) at 90 °C for 20 h. Isolation by column chromatography (*n*-hexane/EtOAc = 2:1) yielded **31** (47.7 mg, 89%). The product is known and the characterization is in consistence with that reported in the literature.[16] The regioselectivity was determined by the analysis of ^{13}C NMR, the ratio of the isomers was determined by the integration of ^1H NMR, *m : p* = 1.0 : 1.4. ^1H NMR (400 MHz, $CDCl_3$) δ 7.42 – 7.25 (m, 4H),

7.24 – 7.18 (m, 1H), 7.15 – 7.00 (m, 2H), 4.86 – 4.71 (m, 1H), 3.12 – 2.96 (m, 1H), 2.81 – 2.70 (m, 1H), 2.58 – 2.44 (m, 1H), 2.40 – 2.26 (m, 1H), 1.86 – 1.69 (m, 1H), 1.48 (s, $3H^m$), 1.46 (s, $3H^p$). ^{13}C NMR (101 MHz, $CDCl_3$) δ 170.2 (C_q), 170.1 (C_q), 163.1 (d, *J* = 246.7 Hz, C_q), 162.3 (d, *J* = 247.6 Hz, C_q), 141.3 (d, *J* = 7.7 Hz, C_q), 138.3 (C_q), 138.3 (C_q), 137.8 (C_q), 137.7 (C_q), 136.7 (C_q), 136.5 (d, *J* = 2.2 Hz, C_q), 135.1 (d, *J* = 3.5 Hz, C_q), 130.6 (d, *J* = 8.4 Hz, CH), 130.0 (d, *J* = 8.1 Hz, CH), 128.7 (CH), 128.7 (CH), 127.5 (CH), 127.1 (CH), 127.1 (CH), 127.0 (CH), 124.0 (d, *J* = 3.0 Hz, CH), 116.1 (d, *J* = 21.5 Hz, CH), 115.4 (d, *J* = 22.1 Hz, CH), 114.5 (d, *J* = 21.0 Hz, CH), 41.8 (CH_2), 26.9 (CH_2), 24.4 (CH_2), 24.3 (CH_2), 21.9 (CH_3). ^{19}F NMR (377 MHz, $CDCl_3$) δ -111.98 (td, *J* = 9.3, 6.0 Hz, $1F^m$), -114.43 (ddd, *J* = 14.0, 8.6, 5.3 Hz, $1F^p$). IR (ATR): 2948, 2878, 1770, 1656, 1509, 1468, 1374, 1237, 840, 792 cm^{-1}. MS (ESI) *m*/*z* (relative intensity): 292 (100) $[M + Na]^+$, 270 (90) $[M + H]^+$. HR-MS (ESI): *m*/*z* calcd. for $[C_{17}H_{16}NO + Na]^+$ 292.1108 found 292.1110.

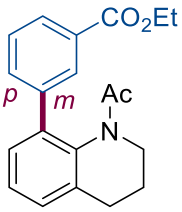

Ethyl 3-(1-acetyl-1,2,3,4-tetrahydroquinolin-8-yl)benzoate (32)

The general procedure **B** was followed using 1-(3,4-dihydroquinolin-1(2*H*)-yl)ethan-1-one (**1a**) (31.7 µL, 0.20 mmol) and ethyl benzoate (**2l**) (1.0 mL, 7.0 mmol) and nBu_4NBF_4 (200 mg, 0.61 mmol, 3.0 equiv.) at 90 °C for 20 h. Isolation by column chromatography (*n*-hexane/EtOAc = 1:1) yielded **32** (28.9 mg, 45%). The regioselectivity was determined by the analysis of ^{1}H NMR and ^{13}C NMR, the ratio of the isomers was determined by the integration of GC spectrum, *m* : *p* = 7 : 1. For *m*-**32**, ^{1}H NMR (400 MHz, $CDCl_3$) δ 8.08 (s, 1H), 8.00 (dt, *J* = 7.4, 1.6 Hz, 1H), 7.51 (dt, *J* = 7.8, 1.7 Hz, 1H), 7.47 (t, *J* = 7.6 Hz, 1H), 7.34 (dd, *J* = 7.7, 1.9 Hz, 1H), 7.30 (t, *J* = 7.5 Hz, 1H), 7.24 – 7.20 (m, 1H), 4.86 – 4.71 (m, 1H), 4.38 (q, *J* = 7.1 Hz, 2H), 3.13 – 3.04 (m, 1H), 2.80 – 2.71 (m, 1H), 2.56 – 2.45 (m, 1H), 2.39 – 2.27 (m, 1H), 1.86 – 1.73 (m, 1H), 1.47 – 1.36 (m, 6H). ^{13}C NMR (101 MHz, $CDCl_3$) δ 170.0 (C_q), 166.3 (C_q), 139.4 (C_q), 138.4 (C_q), 137.8 (C_q), 136.8 (C_q), 132.6 (CH), 131.4 (C_q), 129.6 (CH), 129.0 (CH), 128.8 (CH), 128.6 (CH), 127.4 (CH), 127.1 (CH), 61.2 (CH_2), 41.8 (CH_2), 26.9 (CH_2), 24.4 (CH_2), 22.0 (CH_3), 14.3 (CH_3). IR (ATR): 2979, 2945, 1717, 1660, 1457, 1371, 1248, 1114, 1023, 755 cm^{-1}. MS (ESI) *m*/*z* (relative intensity): 346 (100) $[M + Na]^+$, 324 (10) $[M + H]^+$. HR-MS (ESI): *m*/*z* calcd. for $[C_{20}H_{21}NO_3 + Na]^+$ 346.1414 found 346.1423.

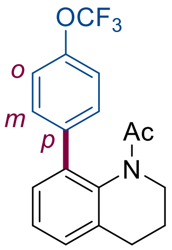

1-(8-(4-(Trifluoromethoxy)phenyl)-3,4-dihydroquinolin-1(*2H*)-yl)ethan-1-one (33)

The general procedure **B** was followed using 1-(3,4-dihydroquinolin-1(*2H*)-yl)ethan-1-one (**1a**) (31.7 µL, 0.20 mmol) and (trifluoromethoxy)benzene (**2m**) (1.0 mL, 7.6 mmol) and *n*Bu_4NBF_4 (200 mg, 0.61 mmol, 3.0 equiv.) at 90 °C for 20 h. Isolation by column chromatography (*n*-hexane/EtOAc = 2:1) yielded **33** (11.2 mg, 17%). The regioselectivity was determined by the analysis of ^{13}C NMR, the ratio of the isomers was determined by the integration of GC spectrum, *m : p* = 1.0 : 1.2. ^{1}H NMR (400 MHz, $CDCl_3$) δ 7.44 (t, *J* = 8.0 Hz, 1H^m), 7.40 – 7.36 (m, 2H^p), 7.31 – 7.17 (m, 6H^m+5H^p), 4.85 – 4.73 (m, 1H^{m+p}), 3.10 – 2.99 (m, 1H^{m+p}), 2.81 – 2.71 (m, 1H^{m+p}), 2.56 – 2.45 (m, 1H^{m+p}), 2.39 – 2.28 (m, 1H^{m+p}), 1.85 – 1.72 (m, 1H^{m+p}), 1.46 (s, 3H^{m+p}). ^{13}C NMR (101 MHz, $CDCl_3$) δ 170.2 (C_q), 170.1 (C_q), 149.7 (q, *J* = 1.6 Hz, C_q), 148.7 (q, *J* = 1.9 Hz, C_q), 141.2 (C_q), 138.4 (C_q), 138.3 (C_q), 137.8 (C_q), 137.7 (C_q), 136.3 (C_q), 136.2 (C_q), 130.4 (CH), 129.8 (CH), 128.7 (CH), 128.6 (CH), 127.7 (CH), 127.4 (CH), 127.2 (CH), 126.7 (CH), 121.4 (CH), 121.0 (CH), 119.9 (CH), 120.5 (q, *J* = 257.4 Hz, C_q), 120.5 (q, *J* = 257.6 Hz, C_q), 41.8 (CH_2), 26.9 (CH_2), 26.8 (CH_2), 24.3 (CH_2), 24.3 (CH_2), 21.9 (CH_3), 21.8 (CH_3). ^{19}F NMR (377 MHz, $CDCl_3$) δ -57.7, -57.7. IR (ATR): 3069, 2928, 2878, 2852, 1660, 1459, 1375, 1256, 1220, 1163 cm^{-1}. MS (ESI) *m*/*z* (relative intensity): 358 (100) $[M + Na]^+$, 336 (40) $[M + H]^+$. HR-MS (ESI): *m*/*z* calcd. for $[C_{18}H_{16}F_3NO_2 + Na]^+$ 358.1025 found 358.1037.

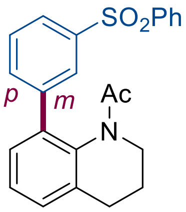

1-(8-(3-(phenylsulfonyl)phenyl)-3,4-dihydroquinolin-1(*2H*)-yl)ethan-1-one (34)

The general procedure **B** was followed using 1-(3,4-dihydroquinolin-1(*2H*)-yl)ethan-1-one (**1a**) (31.7 µL, 0.20 mmol) and sulfonyldibenzene (**2n**) (1.0 g, 4.6 mmol) and *n*Bu_4NBF_4 (200 mg, 0.61 mmol, 3.0 equiv.) at 90 °C for 20 h. Isolation by column chromatography (*n*-hexane/EtOAc = 1:1) yielded **34** (20.2 mg, 26%). The regioselectivity was determined by the analysis of ^{1}H NMR and ^{13}C NMR, the ratio of the isomers was determined by the integration of ^{1}H NMR spectrum, *m : p* = 4 : 1. An unseperatable impurity by column chromatography was

observed. For *m*-**34**, ^{1}H NMR (400 MHz, $CDCl_3$) δ 8.03 – 7.90 (m, 3H), 7.86 (dt, *J* = 7.4, 1.8 Hz, 1H), 7.62 – 7.47 (m, 5H), 7.34 – 7.23 (m, 3H), 4.81 – 4.68 (m, 1H), 3.09 – 2.99 (m, 1H), 2.81 – 2.72 (m, 1H), 2.56 – 2.44 (m, 1H), 2.39 – 2.25 (m, 1H), 1.85 – 1.72 (m, 1H), 1.29 (s, 3H). ^{13}C NMR (101 MHz, $CDCl_3$) δ 169.6 (C_q), 142.5 (C_q), 141.3 (C_q), 140.5 (C_q), 138.4 (C_q), 137.8 (C_q), 135.7 (C_q), 133.3 (CH), 132.9 (CH), 130.0 (CH), 129.4 (CH), 128.6 (CH), 128.0 (CH), 127.7 (CH), 127.6 (CH), 127.2 (CH), 126.5 (CH), 41.8 (CH_2), 26.7 (CH_2), 24.2 (CH_2), 21.7 (CH_3). IR (ATR): 3069, 2947, 2875, 1655, 1447, 1375, 1306, 1154, 1098, 732 cm^{-1}. MS (ESI) *m*/*z* (relative intensity): 414 (100) $[M + Na]^+$, 392 (10) $[M + H]^+$. HR-MS (ESI): *m*/*z* calcd. for $[C_{23}H_{21}NO_3S + Na]^+$ 414.1134 found 414.1135.

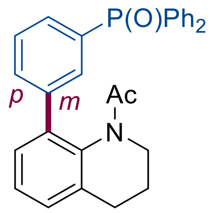

1-(8-(3-(oxo(phenyl)-λ⁴-phosphaneyl)phenyl)-3,4-dihydroquinolin-1(*2H*)-yl)ethan-1-one (35)

The general procedure **B** was followed using 1-(3,4-dihydroquinolin-1(*2H*)-yl)ethan-1-one (**1a**) (31.7 µL, 0.20 mmol) and triphenylphosphine oxide (**2o**) (1.0 g, 3.6 mmol) and *n*Bu_4NBF_4 (200 mg, 0.61 mmol, 3.0 equiv.) at 90 °C for 20 h. Isolation by column chromatography (*n*-DCM/acetone = 2:1) yielded **35** (36.0 mg, 40%). The regioselectivity was determined by the analysis of ^{1}H NMR and ^{13}C NMR, the ratio of the isomers was determined by the integration of ^{1}H NMR spectrum, *m* : *p* = 2 : 1. ^{1}H NMR (400 MHz, $CDCl_3$) δ 7.81 – 7.60 (m, 6H), 7.61 – 7.43 (m, 8H), 7.37 – 7.13 (m, 3H), 4.85 – 4.55 (m, 1H), 3.12 – 2.64 (m, 2H), 2.56 – 2.39 (m, 1H), 2.37 – 2.20 (m, 1H), 1.83 – 1.64 (m, 1H), 1.46 (s, 3H), 1.44 (s, 3H). ^{13}C NMR (101 MHz, $CDCl_3$) δ 170.0 (C_q), 169.9 (C_q), 142.9 (d, *J* = 2.9 Hz, C_q)^{p}, 139.5 (d, *J* = 12.2 Hz, C_q)^{m}, 138.4 (C_q), 138.2 (C_q), 137.8 (C_q), 137.8 (C_q), 136.7 (C_q), 136.5 (C_q), 133.8 (d, *J* = 102.9 Hz, C_q)^{m}, 132.8 (d, *J* = 10.2 Hz, CH), 132.3 (d, *J* = 104.3 Hz, C_q), 132.2 (CH), 132.2 (CH), 132.2 (d, *J* = 104.6 Hz, C_q), 132.2 (CH), 132.1 (CH), 132.1, 132.1 (d, *J* = 104.6 Hz, C_q), 132.1 (CH), 132.0 (CH), 132.0 (CH), 132.0 (CH), 132.0 (CH), 131.9 (CH), 131.2 (d, *J* = 9.8 Hz, CH), 129.4 (d, *J* = 12.4 Hz, CH), 128.7 (d, *J* = 12.4 Hz, CH, CH), 128.7 (d, *J* = 12.2 Hz, CH), 128.6 (d, *J* = 12.0 Hz, CH), 128.5 (d, *J* = 12.4 Hz, CH), 128.9 (CH), 128.8 (CH), 127.8 (CH), 127.5 (CH), 127.2 (CH), 127.1 (CH), 41.8 (CH_2), 41.7 (CH_2), 26.8 (CH_2), 26.7 (CH_2), 24.3 (CH_2), 24.2 (CH_2), 22.0 (CH_2), 21.9 (CH_2). The coupling constands for the peaks between 131.7–132.5 can't be identified. ^{31}P NMR (162 MHz, $CDCl_3$) δ 29.1, 29.0. IR (ATR): 3441, 3058, 2941, 1654, 1454,

1437, 1375, 1190, 1120, 723 cm^{-1}. MS (ESI) *m/z* (relative intensity): 474 (100) $[M + Na]^+$, 452 (10) $[M + H]^+$. HR-MS (ESI): *m/z* calcd. for $[C_{29}H_{26}NO_2P + Na]^+$ 474.1593 found 474.1589.

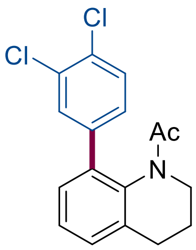

1-(8-(3,4-Dichlorophenyl)-3,4-dihydroquinolin-1(*2H*)-yl)ethan-1-one (36)

The general procedure **B** was followed using 1-(3,4-dihydroquinolin-1(*2H*)-yl)ethan-1-one (**1a**) (31.7 µL, 0.20 mmol) and 1,2-dichlorobenzene (**2p**) (1.0 mL, 8.8 mmol) and *n*Bu_4NBF_4 (40 mg, 0.12 mmol, 0.61 equiv.) at 90 °C for 20 h. Isolation by column chromatography (*n*-hexane/EtOAc = 2:1) yielded **36** (32.7 mg, 51%). The regioselectivity was determined by the analysis of ^{1}H NMR, the ratio of the isomers was determined by the integration of ^{1}H NMR, $\alpha : \beta = 1 : 10$. ^{1}H NMR (300 MHz, $CDCl_3$) δ 7.48 (d, *J* = 5.3 Hz, 1H), 7.47 (d, *J* = 0.9 Hz, 1H), 7.33 – 7.21 (m, 3H), 7.17 (dd, *J* = 8.4, 2.1 Hz, 1H), 4.84 – 4.71 (m, 1H), 3.11 – 2.97 (m, 1H), 2.82 – 2.70 (m, 1H), 2.58 – 2.42 (m, 1H), 2.41 – 2.25 (m, 1H), 1.86 – 1.67 (m, 1H), 1.50 (s, 3H). ^{13}C NMR (75 MHz, $CDCl_3$) δ 170.0 (C_q), 139.1 (C_q), 138.4 (C_q), 137.7 (C_q), 135.3 (C_q), 133.2 (C_q), 131.9 (C_q), 131.0 (CH), 130.2 (CH), 128.5 (CH), 127.8 (CH), 127.6 (CH), 127.2 (CH), 41.8 (CH_2), 26.8 (CH_2), 24.3 (CH_2), 22.0 (CH_3). IR (ATR): 2947, 2930, 1770, 1759, 1661, 1458, 1373, 1246, 1061, 1028 cm^{-1}. MS (ESI) *m/z* (relative intensity): 342 (100) $[M + Na]^+$, 320 (40) $[M + H]^+$. HR-MS (ESI): *m/z* calcd. for $[C_{17}H_{15}Cl_2NO + Na]^+$ 342.0423 found 342.0413.

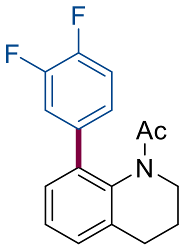

1-(8-(3,4-Difluorophenyl)-3,4-dihydroquinolin-1(*2H*)-yl)ethan-1-one (37)

The general procedure **B** was followed using 1-(3,4-dihydroquinolin-1(*2H*)-yl)ethan-1-one (**1a**) (31.7 µL, 0.20 mmol) and 1,2-difluorobenzene (**2q**) (1.0 mL, 10.2 mmol) and *n*Bu_4NBF_4 (200 mg, 0.61 mmol, 3.0 equiv.) at 90 °C for 20 h. Isolation by column chromatography (*n*-hexane/EtOAc = 2:1) yielded **37** (27.0 mg, 47%). The regioselectivity was determined by the analysis of ^{13}C NMR, the ratio of the isomers was determined by the integration of ^{1}H NMR,

α : *β* = 1 : 10. ^{1}H NMR (300 MHz, $CDCl_3$) δ 7.33 – 7.13 (m, 5H), 7.11 – 7.03 (m, 1H), 4.87 – 4.70 (m, 1H), 3.10 – 2.95 (m, 1H), 2.84 – 2.69 (m, 1H), 2.60 – 2.41 (m, 1H), 2.40 – 2.25 (m, 1H), 1.85 – 1.68 (m, 1H), 1.49 (s, 3H). ^{13}C NMR (101 MHz, $CDCl_3$) δ 170.1 (C_q), 150.6 (dd, *J* = 249.1, 12.8 Hz, C_q), 149.9 (dd, *J* = 250.0, 12.7 Hz, C_q), 138.4 (C_q), 137.7 (C_q), 136.1 (dd, *J* = 5.9, 4.1 Hz, C_q), 135.6 (C_q), 128.6 (CH), 127.6 (CH), 127.2 (CH), 124.5 (dd, *J* = 6.2, 3.6 Hz, CH), 118.0 (d, *J* = 17.2 Hz, CH), 117.4 (d, *J* = 17.8 Hz, CH), 41.8 (CH_2), 26.8 (CH_2), 24.3 (CH_2), 21.9 (CH_3). ^{19}F NMR (282 MHz, $CDCl_3$) δ -136.4 (ddd, *J* = 20.2, 11.3, 8.1 Hz), -138.9 (dddd, *J* = 21.6, 10.0, 7.5, 4.3 Hz). IR (ATR): 2948, 2880, 1770, 1657, 1518, 1459, 1374, 1267, 1240, 771 cm^{-1}. MS (ESI) *m*/*z* (relative intensity): 310 (100) $[M + Na]^+$, 288 (70) $[M + H]^+$. HR-MS (ESI): *m*/*z* calcd. for $[C_{17}H_{15}F_2NO + Na]^+$ 310.1014 found 310.1008.

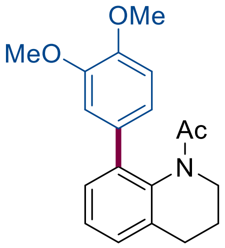

1-(8-(3,4-Dimethoxyphenyl)-3,4-dihydroquinolin-1(2*H*)-yl)ethan-1-one (38)

The general procedure **A** was followed using 1-(3,4-dihydroquinolin-1(2*H*)-yl)ethan-1-one (**1a**) (31.7 µL, 0.20 mmol) and veratrole (**2r**) (76.5 µL, 0.60 mmol, 3.0 equiv.) and *n*Bu_4NBF_4 (40 mg, 0.12 mmol, 0.61 equiv.) at 100 °C for 18 h. Isolation by column chromatography (*n*-hexane/EtOAc = 1:1) and gel permeation chromatography yielded **38** (43.4 mg, 70%). ^{1}H NMR (600 MHz, $CDCl_3$) δ 7.32 (ddd, *J* = 7.8, 1.6, 0.8 Hz, 1H), 7.25 (t, *J* = 7.6 Hz, 1H), 7.16 (dt, *J* = 7.3, 1.2 Hz, 1H), 6.93 (dd, *J* = 8.3, 1.9 Hz, 1H), 6.91 (d, *J* = 8.3 Hz, 1H), 6.86 (d, *J* = 1.9 Hz, 1H), 4.81 – 4.74 (m, 1H), 3.89 (s, 3H), 3.87 (s, 3H), 3.08 – 3.00 (m, 1H), 2.76 – 2.69 (m, 1H), 2.53 – 2.45 (m, 1H), 2.37 – 2.28 (m, 1H), 1.81 – 1.70 (m, 1H), 1.47 (s, 3H). ^{13}C NMR (126 MHz, $CDCl_3$) δ 170.4 (C_q), 149.1 (C_q), 148.5 (C_q), 138.3 (C_q), 137.5 (C_q), 137.3 (C_q), 131.6 (C_q), 128.4 (CH), 126.8 (CH), 126.4 (CH), 120.6 (CH), 111.6 (CH), 111.4 (CH), 55.9 (CH_3), 55.8 (CH_3), 41.5 (CH_2), 26.9 (CH_2), 24.3 (CH_2), 21.8 (CH_3). IR (ATR): 2995, 2947, 2837, 1770, 1653, 1518, 1458, 1376, 1249, 1025 cm^{-1}. MS (ESI) *m*/*z* (relative intensity): 334 (100) $[M + Na]^+$, 312 (80) $[M + H]^+$. HR-MS (ESI): *m*/*z* calcd. for $[C_{19}H_{21}NO_3 + Na]^+$ 334.1414 found 334.1417.

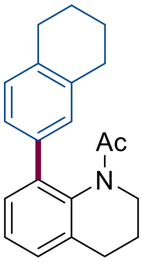

1-(8-(5,6,7,8-Tetrahydronaphthalen-2-yl)-3,4-dihydroquinolin-1(2*H*)-yl)ethan-1-one (39)

The general procedure **A** was followed using 1-(3,4-dihydroquinolin-1(2*H*)-yl)ethan-1-one (**1a**) (31.7 µL, 0.20 mmol) and 1,2,3,4-tetrahydronaphthalene (**2s**) (136.0 µL, 1.0 mmol, 5.0 equiv.) and *n*Bu_4NBF_4 (200 mg, 0.61 mmol, 3.0 equiv.) at 100 °C for 20 h. Isolation by column chromatography (*n*-hexane/EtOAc = 2:1) yielded **39** (30.4 mg, 50%). ^{1}H NMR (300 MHz, $CDCl_3$) δ 7.30 (dd, *J* = 7.8, 1.8 Hz, 1H), 7.25 (t, *J* = 7.4 Hz, 1H), 7.16 (dd, *J* = 7.3, 2.1 Hz, 1H), 7.11 – 7.01 (m, 3H), 4.84 – 4.68 (m, 1H), 3.14 – 3.00 (m, 1H), 2.85 – 2.68 (m, 5H), 2.56 – 2.42 (m, 1H), 2.41 – 2.24 (m, 1H), 1.89 – 1.70 (m, 5H), 1.48 (s, 3H). ^{13}C NMR (75 MHz, $CDCl_3$) δ 170.4 (C_q), 138.1 (C_q), 138.0 (C_q), 137.7 (C_q), 137.7 (C_q), 136.6 (C_q), 136.2 (C_q), 129.8 (CH), 128.9 (CH), 128.7 (CH), 126.8 (CH), 126.4 (CH), 125.3 (CH), 41.7 (CH_2), 29.5 (CH_2), 29.1 (CH_2), 26.9 (CH_2), 24.4 (CH_2), 23.2 (CH_2), 22.1 (CH_3). IR (ATR): 2929, 2876, 1656, 1456, 1404, 1373, 1339, 1260, 1246, 791 cm^{-1}. MS (ESI) *m*/*z* (relative intensity): 328 (100) $[M + Na]^+$, 306 (10) $[M + H]^+$. HR-MS (ESI): *m*/*z* calcd. for $[C_{21}H_{23}NO + Na]^+$ 328.1672 found 328.1673.

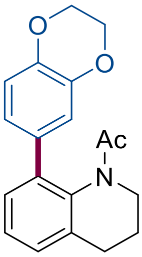

1-(8-(2,3-Dihydrobenzo[*b*][1,4]dioxin-6-yl)-3,4-dihydroquinolin-1(2*H*)-yl)ethan-1-one (40)

The general procedure **A** was followed using 1-(3,4-dihydroquinolin-1(2*H*)-yl)ethan-1-one (**1a**) (31.7 µL, 0.20 mmol) and 2,3-dihydrobenzo[*b*][1,4]dioxine (**2t**) (136.2 mg, 1.0 mmol, 5.0 equiv.) and *n*Bu_4NBF_4 (200 mg, 0.61 mmol, 3.0 equiv.) at 100 °C for 20 h. Isolation by column chromatography (*n*-hexane/EtOAc = 1:1) yielded **40** (33.5 mg, 54%). ^{1}H NMR (300 MHz, $CDCl_3$) δ 7.30 – 7.20 (m, 2H), 7.15 (dd, *J* = 6.3, 2.7 Hz, 1H), 6.92 – 6.76 (m, 3H), 4.81 – 4.67 (m, 1H), 4.27 (s, 4H), 3.09 – 2.97 (m, 1H), 2.79 – 2.65 (m, 1H), 2.55 – 2.40 (m, 1H), 2.37 – 2.24 (m, 1H), 1.82 – 1.66 (m, 1H), 1.50 (s, 3H). ^{13}C NMR (75 MHz, $CDCl_3$) δ 170.8 (C_q), 143.8 (C_q), 143.2 (C_q), 138.2 (C_q), 137.4 (C_q), 137.3 (C_q), 132.3 (C_q), 128.7 (CH), 127.0 (CH), 126.5 (CH), 121.4 (CH), 117.9 (CH), 117.1 (CH), 64.4 (CH_2), 64.4 (CH_2), 41.8 (CH_2), 26.8 (CH_2),

24.3 (CH_2), 21.9 (CH_3). IR (ATR): 2983, 2946, 2880, 1770, 1653, 1508, 1471, 1377, 1246, 1064 cm^{-1}. MS (ESI) *m*/*z* (relative intensity): 332 (100) $[M + Na]^+$, 310 (55) $[M + H]^+$. HR-MS (ESI): *m*/*z* calcd. for $[C_{19}H_{19}NO_3 + Na]^+$ 332.1257 found 332.1257.

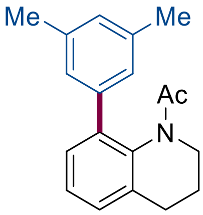

1-(8-(3,5-Dimethylphenyl)-3,4-dihydroquinolin-1(2*H*)-yl)ethan-1-one (41)

The general procedure **B** was followed using 1-(3,4-dihydroquinolin-1(2*H*)-yl)ethan-1-one (**1a**) (31.7 µL, 0.20 mmol) and *m*-xylene (**2u**) (123.4 µL, 1.0 mmol, 5.0 equiv.) and *n*Bu_4NBF_4 (200 mg, 0.61 mmol, 3.0 equiv.) at 90 °C for 20 h. Isolation by column chromatography (*n*-hexane/EtOAc = 2:1) yielded **41** (28.6 mg, 51%). The product is known and the characterization is in consistence with that reported in the literature.[16] ^{1}H NMR (300 MHz, $CDCl_3$) δ 7.31 (dd, *J* = 7.8, 1.9 Hz, 1H), 7.25 (t, *J* = 7.4 Hz, 1H), 7.17 (dd, *J* = 7.1, 2.3 Hz, 1H), 6.95 (s, 1H), 6.94 (s, 2H), 4.88 – 4.69 (m, 1H), 3.15 – 2.97 (m, 1H), 2.82 – 2.65 (m, 1H), 2.57 – 2.41 (m, 1H), 2.40 – 2.24 (m, 7H), 1.86 – 1.66 (m, 1H), 1.47 (s, 3H). ^{13}C NMR (75 MHz, $CDCl_3$) δ 170.3 (C_q), 139.1 (C_q), 138.4 (C_q), 138.2 (C_q), 138.0 (C_q), 137.7 (C_q), 129.2 (CH), 128.9 (CH), 126.8 (CH), 126.6 (CH), 126.1 (CH), 41.7 (CH_2), 27.0 (CH_2), 24.4 (CH_2), 22.0 (CH_3), 21.5 (CH_3). IR (ATR): 2948, 1759, 1658, 1602, 1459, 1417, 1374, 1339, 1246, 1061 cm^{-1}. MS (ESI) *m*/*z* (relative intensity): 302 (100) $[M + Na]^+$, 280 (60) $[M + H]^+$. HR-MS (ESI): *m*/*z* calcd. for $[C_{19}H_{21}NO + Na]^+$ 302.1515 found 302.1519.

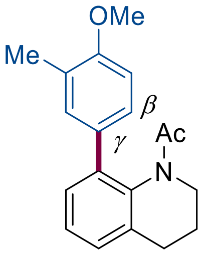

1-(8-(4-Methoxy-3-methylphenyl)-3,4-dihydroquinolin-1(2*H*)-yl)ethan-1-one (42)

The general procedure **A** was followed using 1-(3,4-dihydroquinolin-1(2*H*)-yl)ethan-1-one (**1a**) (31.7 µL, 0.20 mmol) and 1-methoxy-2-methylbenzene (**2v**) (124.7 µL, 1.0 mmol, 5.0 equiv.) and *n*Bu_4NBF_4 (200 mg, 0.61 mmol, 3.0 equiv.) at 100 °C for 20 h. Isolation by column chromatography (*n*-hexane/EtOAc = 2:1) yielded **42** (41.7 mg, 71%) as a mixture of isomer. The regioselectivity was determined by the analysis of NOESY NMR, the ratio of the isomers

was determined by the integration of ^{1}H NMR, $\beta : \gamma = 1 : 5$. For γ-arylated product, ^{1}H NMR (500 MHz, $CDCl_3$) δ 7.29 (ddd, J = 7.8, 1.7, 0.8 Hz, 1H), 7.24 (t, J = 7.5 Hz, 1H), 7.16 – 7.11 (m, 3H), 6.84 (d, J = 9.1 Hz, 1H), 4.83 – 4.69 (m, 1H), 3.84 (s, 3H), 3.13 – 3.01 (m, 1H), 2.77 – 2.66 (m, 1H), 2.54 – 2.40 (m, 1H), 2.37 – 2.28 (m, 1H), 2.23 (s, 3H), 1.82 – 1.67 (m, 1H), 1.47 (s, 3H). ^{13}C NMR (126 MHz, $CDCl_3$) δ 170.4 (C_q), 157.2 (C_q), 138.1 (C_q), 137.6 (C_q), 137.5 (C_q), 130.9 (C_q), 130.5 (CH), 128.6 (CH), 127.2 (C_q), 126.8 (CH), 126.6 (CH), 126.1 (CH), 110.2 (CH), 55.3 (CH_3), 41.6 (CH_2), 26.9 (CH_2), 24.4 (CH_2), 21.9 (CH_3), 16.4 (CH_3). IR (ATR): 2995, 2948, 1770, 1655, 1507, 1459, 1375, 1246, 1138, 792 cm^{-1}. MS (ESI) m/z (relative intensity): 318 (100) $[M + Na]^+$, 296 (20) $[M + H]^+$. HR-MS (ESI): m/z calcd. for $[C_{19}H_{21}NO_2 + Na]^+$ 318.1465 found 318.1468.

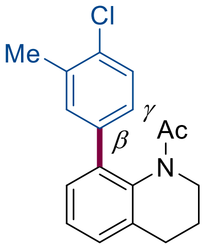

1-(8-(4-chloro-3-methylphenyl)-3,4-dihydroquinolin-1(*2H*)-yl)ethan-1-one (43)

The general procedure **B** was followed using 1-(3,4-dihydroquinolin-1(*2H*)-yl)ethan-1-one (**1a**) (31.7 µL, 0.20 mmol) and 1-chloro-2-methylbenzene (**2w**) (1.0 mL, 8.6 mmol) and nBu_4NBF_4 (200 mg, 0.61 mmol, 3.0 equiv.) at 90 °C for 20 h. Isolation by column chromatography (*n*-hexane/EtOAc = 2:1) yielded **43** (37.1 mg, 62%). The regioselectivity was determined by the analysis of HMBC NMR, the ratio of the isomers was determined by the integration of ^{1}H NMR, $\beta : \gamma = 1.2 : 1.0$. ^{1}H NMR (400 MHz, $CDCl_3$) δ 7.37 (d, J = 6.4 Hz, $1H^{\beta}$), 7.35 (s, $1H^{\gamma}$), 7.30 – 7.17 (m, $4H^{\beta+\gamma}$), 7.10 (dt, J = 8.2, 2.4 Hz, $1H^{\beta+\gamma}$), 4.82 – 4.70 (m, $1H^{\beta+\gamma}$), 3.12 – 2.97 (m, $1H^{\beta+\gamma}$), 2.80 – 2.68 (m, $1H^{\beta+\gamma}$), 2.55 – 2.43 (m, $1H^{\beta+\gamma}$), 2.42 – 2.26 (m, $4H^{\beta+\gamma}$), 1.85 – 1.69 (m, $1H^{\beta+\gamma}$), 1.48 (s, $3H^{\gamma}$), 1.47 (s, $3H^{\beta}$). ^{13}C NMR (101 MHz, $CDCl_3$) δ 170.2 (C_q), 170.2 (C_q), 138.3 (C_q), 138.3 (C_q), 137.7 (C_q), 137.7 (C_q), 137.6 (C_q), 136.7 (C_q), 136.4 (C_q), 135.4 (C_q), 134.9 (C_q), 133.9 (C_q), 131.5 (CH), 130.8 (CH), 129.7 (CH), 128.8 (CH), 128.6 (CH), 128.6 (CH), 127.2 (CH), 127.1 (CH), 127.0 (CH), 127.0 (CH), 127.0 (CH), 126.4 (CH), 41.8 (CH_2), 26.9 (CH_2), 24.4 (CH_2), 24.4 (CH_2), 22.0 (CH_3), 22.0 (CH_3), 20.3 (CH_3), 19.8 (CH_3). IR (ATR): 2947, 2876, 1653, 1457, 1371, 1334, 1261, 1193, 1047, 730 cm^{-1}. MS (ESI) m/z (relative intensity): 322 (100) $[M + Na]^+$, 300 (10) $[M + H]^+$. HR-MS (ESI): m/z calcd. for $[C_{18}H_{18}ClNO + Na]^+$ 322.0969 found 322.0979.

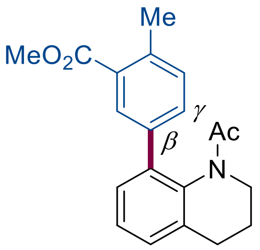

Methyl 5-(1-acetyl-1,2,3,4-tetrahydroquinolin-8-yl)-2-methylbenzoate (44)

The general procedure **B** was followed using 1-(3,4-dihydroquinolin-1(2*H*)-yl)ethan-1-one (**1a**) (31.7 µL, 0.20 mmol) and methyl 2-methylbenzoate (**2x**) (0.5 mL, 3.6 mmol) and *n*Bu_4NBF_4 (200 mg, 0.61 mmol, 3.0 equiv.) at 90 °C for 20 h. Isolation by column chromatography (*n*-hexane/EtOAc = 1:1) yielded **44** (27.1 mg, 42%). The regioselectivity was determined by the analysis of HMBC NMR, the ratio of the isomers was determined by the integration of ^{1}H NMR, $\beta : \gamma = 4 : 1$. For *β*-arylated product, ^{1}H NMR (400 MHz, $CDCl_3$) δ 7.97 (d, *J* = 2.1 Hz, 1H), 7.35 (dd, *J* = 7.9, 2.1 Hz, 1H), 7.34 – 7.31 (m, 1H), 7.30 – 7.26 (m, 2H), 7.24 – 7.18 (m, 1H), 4.82 – 4.72 (m, 1H), 3.89 (s, 3H), 3.12 – 3.03 (m, 1H), 2.79 – 2.70 (m, 1H), 2.61 (s, 3H), 2.54 – 2.45 (m, 1H), 2.38 – 2.28 (m, 1H), 1.84 – 1.70 (m, 1H), 1.46 (s, 3H). ^{13}C NMR (101 MHz, $CDCl_3$) δ 170.2 (C_q), 167.7 (C_q), 139.7 (C_q), 138.3 (C_q), 137.7 (C_q), 136.7 (C_q), 136.7 (C_q), 132.5 (CH), 131.6 (CH), 130.7 (CH), 130.3 (C_q), 128.6 (CH), 127.1 (CH), 127.0 (CH), 52.0 (CH_3), 41.8 (CH_2), 26.9 (CH_2), 24.4 (CH_2), 22.0 (CH_3), 21.6 (CH_3). IR (ATR): 2950, 2852, 1722, 1658, 1456, 1436, 1374, 1291, 1080, 780 cm^{-1}. MS (ESI) *m*/*z* (relative intensity): 346 (100) $[M + Na]^+$, 324 (20) $[M + H]^+$. HR-MS (ESI): *m*/*z* calcd. for $[C_{20}H_{21}NO_3 + Na]^+$ 346.1414 found 346.1415.

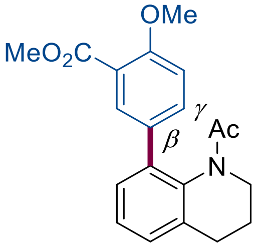

Methyl 5-(1-acetyl-1,2,3,4-tetrahydroquinolin-8-yl)-2-methoxybenzoate (45)

The general procedure **B** was followed using 1-(3,4-dihydroquinolin-1(2*H*)-yl)ethan-1-one (**1a**) (31.7 µL, 0.20 mmol) and methyl 2-methoxybenzoate (**2y**) (1.0 mL, 7.0 mmol) and *n*Bu_4NBF_4 (200 mg, 0.61 mmol, 3.0 equiv.) at 90 °C for 20 h. Isolation by column chromatography (*n*-hexane/EtOAc = 1:1) yielded **45** (29.4 mg, 43%). The regioselectivity was determined by the analysis of NOESY NMR. ^{1}H NMR (400 MHz, $CDCl_3$) δ 7.88 (d, *J* = 2.4 Hz, 1H), 7.42 (dd, *J* = 8.7, 2.5 Hz, 1H), 7.31 (ddd, *J* = 7.8, 1.8, 0.7 Hz, 1H), 7.26 (t, *J* = 7.5 Hz, 1H), 7.18 (ddd, *J* =

7.2, 1.8, 0.9 Hz, 1H), 7.01 (d, *J* = 8.7 Hz, 1H), 4.83 – 4.70 (m, 1H), 3.92 (s, 3H), 3.89 (s, 3H), 3.10 – 3.00 (m, 1H), 2.77 – 2.68 (m, 1H), 2.54 – 2.43 (m, 1H), 2.38 – 2.25 (m, 1H), 1.84 – 1.69 (m, 1H), 1.47 (s, 3H). ^{13}C NMR (101 MHz, $CDCl_3$) δ 170.2 (C_q), 166.4 (C_q), 158.7 (C_q), 138.3 (C_q), 137.6 (C_q), 136.3 (C_q), 133.2 (CH), 131.9 (CH), 131.0 (C_q), 128.5 (CH), 127.1 (CH), 126.9 (CH), 120.5 (C_q), 112.6 (CH), 56.1 (CH_3), 52.2 (CH_3), 41.7 (CH_2), 26.8 (CH_2), 24.3 (CH_2), 22.0 (CH_3). IR (ATR): 2951, 2848, 1731, 1656, 1610, 1504, 1436, 1375, 1275, 1235 cm^{-1}. MS (ESI) *m*/*z* (relative intensity): 362 (100) [M + Na]$^+$, 340 (20) [M + H]$^+$. HR-MS (ESI): *m*/*z* calcd. for [$C_{20}H_{21}NO_4$ + Na]$^+$ 362.1363 found 362.1364.

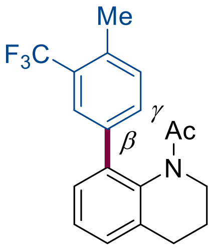

1-(8-(4-Methyl-3-(trifluoromethyl)phenyl)-3,4-dihydroquinolin-1(2*H*)-yl)ethan-1-one (46)

The general procedure **B** was followed using 1-(3,4-dihydroquinolin-1(2*H*)-yl)ethan-1-one (**1a**) (31.7 µL, 0.20 mmol) and trifluorotoluene (**2z**) (1.0 mL, 8.1 mmol) and *n*Bu_4NBF_4 (200 mg, 0.61 mmol, 3.0 equiv.) at 90 °C for 20 h. Isolation by column chromatography (*n*-hexane/EtOAc = 1:1) yielded **46** (24.0 mg, 36%). The regioselectivity was determined by the analysis of HSQC NMR, the ratio of the isomers was determined by the integration of ^{1}H NMR, *β* : *γ* = 3 : 1. For *β*-arylated product, ^{1}H NMR (400 MHz, $CDCl_3$) δ 7.62 (d, *J* = 1.9 Hz, 1H), 7.37 (dd, *J* = 7.9, 1.9 Hz, 1H), 7.34 – 7.28 (m, 3H), 7.28 – 7.20 (m, 1H), 4.82 – 4.72 (m, 1H), 3.11 – 3.02 (m, 1H), 2.80 – 2.72 (m, 1H), 2.56 – 2.45 (m, 4H), 2.40 – 2.29 (m, 1H), 1.84 – 1.74 (m, 1H), 1.45 (s, 3H). ^{13}C NMR (101 MHz, $CDCl_3$) δ 170.2 (C_q), 138.4 (C_q), 137.7 (C_q), 136.9 (C_q), 136.4 (C_q), 136.0 (q, *J* = 1.8 Hz, C_q), 132.7 (CH), 131.3 (CH), 129.7 (q, *J* = 29.9 Hz, C_q), 128.6 (CH), 127.4 (CH), 127.6 (CH), 125.8 (q, *J* = 5.6 Hz, CH), 124.4 (q, *J* = 273.7 Hz, C_q), 41.8 (CH_2), 26.9 (CH_2), 24.4 (CH_2), 22.0 (CH_3), 19.1 (q, *J* = 2.1 Hz, CH_3). ^{19}F NMR (377 MHz, $CDCl_3$) δ -61.5, -61.7. IR (ATR): 2946, 2877, 1660, 1458, 1374, 1315, 1269, 1120, 1055, 1041 cm^{-1}. MS (ESI) *m*/*z* (relative intensity): 356 (100) [M + Na]$^+$, 334 (20) [M + H]$^+$. HR-MS (ESI): *m*/*z* calcd. for [$C_{19}H_{18}F_3NO$ + Na]$^+$ 356.1233 found 356.1232.

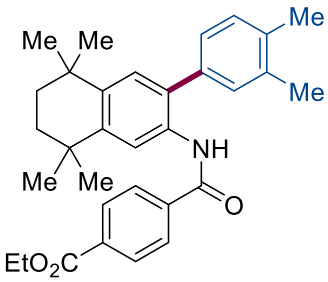

Ethyl 4-((3-(3,4-dimethylphenyl)-5,5,8,8-tetramethyl-5,6,7,8-tetrahydronaphthalen-2-yl)carbamoyl)benzoate (47)

The general procedure **A** was followed using Tamibarotene ester (**1t**) (76.0 mg, 0.20 mmol) and *o*-xylene (**2a**) (1.0 mL, 8.3 mmol) and *n*Bu_4NBF_4 (200 mg, 0.61 mmol, 3.0 equiv.) at 100 °C for 20 h. Isolation by column chromatography (*n*-hexane/EtOAc = 10:1) yielded **47** (90.0 mg, 93%). ^{1}H NMR (300 MHz, $CDCl_3$) δ 8.47 (s, 1H), 8.14 – 8.02 (m, 3H), 7.75 – 7.65 (m, 2H), 7.26 (d, *J* = 7.6 Hz, 1H), 7.22 (m, 2H), 7.18 (dd, *J* = 7.6, 1.9 Hz, 1H), 4.40 (q, *J* = 7.1 Hz, 2H), 2.35 (s, 3H), 2.33 (s, 3H), 1.74 (s, 4H), 1.44 – 1.37 (m, 9H), 1.30 (s, 6H). ^{13}C NMR (75 MHz, $CDCl_3$) δ 165.7 (C_q), 164.0 (C_q), 145.3 (C_q), 141.5 (C_q), 138.9 (C_q), 137.6 (C_q), 136.5 (C_q), 135.7 (C_q), 133.1 (C_q), 132.2 (C_q), 130.6 (CH), 130.4 (CH), 130.0 (C_q), 129.9 (CH), 128.1 (CH), 126.8 (CH), 126.6 (CH), 118.9 (CH), 61.4 (CH_2), 35.1 (C_q), 34.6 (CH_2), 34.1 (CH_2), 31.9 (CH_3), 31.8 (CH_3), 19.9 (CH_3), 19.6 (CH_3), 14.3 (CH_3). IR (ATR): 3414, 2958, 2924, 1720, 1679, 1516, 1271, 1107, 1019, 724 cm^{-1}. MS (ESI) *m*/*z* (relative intensity): 506 (100) $[M + Na]^+$, 484 (50) $[M + H]^+$. HR-MS (ESI): *m*/*z* calcd. for $[C_{32}H_{37}NO_3 + Na]^+$ 506.2666 found 506.2660.

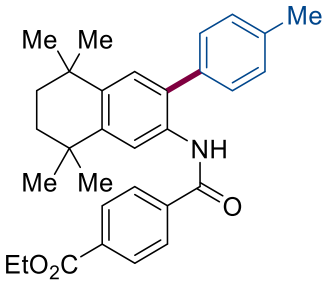

Ethyl 4-((5,5,8,8-tetramethyl-3-(*p*-tolyl)-5,6,7,8-tetrahydronaphthalen-2-yl)carbamoyl)benzoate (48)

The general procedure **A** was followed using Tamibarotene ester (**1t**) (76.0 mg, 0.20 mmol) and toluene (**2d**) (1.0 mL, 9.4 mmol) and *n*Bu_4NBF_4 (200 mg, 0.61 mmol, 3.0 equiv.) at 100 °C for 20 h. Isolation by column chromatography (*n*-hexane/EtOAc = 10:1) yielded **48** (89.0 mg, 95%). The regioselectivity was determined by the analysis of ^{13}C NMR, the ratio of the isomers was determined by the integration of ^{1}H NMR, *m* : *p* = 1.0 : 1.1. ^{1}H NMR (300 MHz, $CDCl_3$) δ 8.52 – 8.45 (m, 1H), 8.11 – 8.00 (m, 3H), 7.74 – 7.65 (m, 2H), 7.44 – 7.20 (m, 5H), 4.39 (q, *J*

= 7.1 Hz, 2H), 2.44 – 2.41 (m, 3H), 1.75 (s, 4H), 1.46 – 1.35 (m, 9H), 1.31 (s, 6H^{m}), 1.30 (s, 6H^{p}). ^{13}C NMR (75 MHz, $CDCl_3$) δ 165.7 (C_q), 165.7 (C_q), 164.2 (C_q), 164.1 (C_q), 145.5 (C_q), 145.4 (C_q), 141.6 (C_q), 141.6 (C_q), 139.0 (C_q), 138.8 (C_q), 138.2 (C_q), 137.9 (C_q), 135.3 (C_q), 133.1 (C_q), 132.1 (C_q), 132.1 (C_q), 130.2 (C_q), 130.2 (CH), 130.1 (C_q), 130.0 (CH), 129.9 (CH), 129.2 (CH), 129.1 (CH), 128.7 (CH), 128.2 (CH), 128.0 (CH), 126.8 (CH), 126.8 (CH), 126.3 (CH), 119.2 (CH), 119.1 (CH), 61.4 (CH_2), 35.1 (C_q), 34.6 (CH_2), 34.1 (CH_2), 31.9 (CH_3), 31.8 (CH_3), 21.5 (CH_3), 21.3 (CH_3), 14.3 (CH_3). IR (ATR): 3423, 2959, 2925, 1720, 1678, 1569, 1517, 1473, 1273, 1108 cm^{-1}. MS (ESI) *m*/*z* (relative intensity): 492 (100) $[M + Na]^+$, 470 (80) $[M + H]^+$. HR-MS (ESI): *m*/*z* calcd. for $[C_{31}H_{35}NO_3 + Na]^+$ 492.2509 found 492.2503.

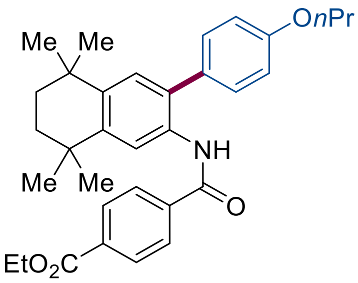

Ethyl 4-((5,5,8,8-tetramethyl-3-(4-propoxyphenyl)-5,6,7,8-tetrahydronaphthalen-2-yl)carbamoyl)benzoate (49)

The general procedure **A** was followed Tamibarotene ester (**1t**) (76.0 mg, 0.20 mmol) and propoxybenzene (**2a'**) (0.5 mL, 3.5 mmol) and *n*Bu_4NBF_4 (200 mg, 0.61 mmol, 3.0 equiv.) at 100 °C for 20 h. Isolation by column chromatography (*n*-hexane/EtOAc = 5:1) yielded **49** (21.0 mg, 20%). The regioselectivity was determined by the analysis of ^{13}C NMR, the ratio of the isomers was determined by the integration of ^{1}H NMR, *m* : *p* = 1 : 3. ^{1}H NMR (400 MHz, $CDCl_3$) δ 8.47 (s, 1H), 8.08 – 8.04 (m, 2H), 7.98 (s, 1H), 7.72 – 7.66 (m, 2H), 7.39 – 7.32 (m, 2H), 7.20 (s, 1H), 7.04 – 6.99 (m, 2H), 4.39 (q, *J* = 7.2 Hz, 2H), 3.98 (t, *J* = 6.6 Hz, 2H), 1.91 – 1.81 (m, 2H), 1.76 – 1.71 (m, 4H), 1.43 – 1.37 (m, 9H), 1.30 (s, 6H), 1.08 (t, *J* = 7.4 Hz, 3H). ^{13}C NMR (101 MHz, $CDCl_3$) δ 165.7 (C_q), 164.1 (C_q), 159.0 (C_q), 145.2 (C_q), 141.5 (C_q), 138.9 (C_q), 135.2 (C_q), 132.3 (C_q), 130.5 (CH), 130.1 (C_q), 130.0 (CH), 129.9 (C_q), 128.2 (CH), 126.8 (CH), 119.1 (CH), 115.2 (CH), 69.7 (CH_2), 61.4 (CH_2), 35.2 (C_q), 34.6 (CH_2), 34.1 (CH_2), 31.9 (CH_3), 31.8 (CH_3), 22.6 (CH_2), 14.3 (CH_3), 10.6 (CH_3). IR (ATR): 3421, 2962, 2932, 1720, 1678, 1514, 1472, 1273, 1246, 1108 cm^{-1}. MS (ESI) *m*/*z* (relative intensity): 536 (100) $[M + Na]^+$, 514 (30) $[M + H]^+$. HR-MS (ESI): *m*/*z* calcd. for $[C_{33}H_{39}NO_4 + Na]^+$ 536.2771 found 536.2768.

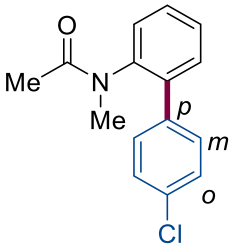

N-(4'-chloro-[1,1'-biphenyl]-2-yl)-N-methylacetamide (50)

The general procedure **B** was followed using *N*-methyl-*N*-phenylacetamide (**1n**) (29.8 mg, 0.20 mmol) and chlorobenzene (**2j**) (1.0 mL, 9.9 mmol) and $n\mathrm{Bu_4NBF_4}$ (200 mg, 0.61 mmol, 3.0 equiv.) at 90 °C for 20 h. Isolation by column chromatography (*n*-hexane/EtOAc = 2:1) yielded **50** (34.0 mg, 65%). The regioselectivity was determined by the analysis of ^{13}C NMR, the ratio of the isomers was determined by the integration of ^{1}H NMR, *m* : *p* = 1.0 : 1.2. ^{1}H NMR (300 MHz, $CDCl_3$) δ 7.46 – 7.28 (m, 4H), 7.36 – 7.32 (m, 1H), 7.25 – 7.13 (m, 3H), 3.02 (s, $3H^m$), 3.01 (s, $3H^p$), 1.80 (s, $3H^m$), 1.79 (s, $3H^p$). ^{13}C NMR (75 MHz, $CDCl_3$) δ 170.5 (C_q), 170.5 (C_q), 142.0 (C_q), 141.9 (C_q), 140.5 (C_q), 138.4 (C_q), 138.4 (C_q), 137.1 (C_q), 134.6 (C_q), 134.0 (C_q), 131.3 (CH), 131.3 (CH), 130.0 (CH), 129.7 (CH), 129.4 (CH), 129.2 (CH), 129.0 (CH), 128.7 (CH), 128.6 (CH), 128.6 (CH), 128.5 (CH), 127.9 (CH), 126.4 (CH), 37.1 (CH_3), 37.0 (CH_3), 22.4 (CH_3). IR (ATR): 3060, 2984, 2935, 1660, 1477, 1375, 1350, 1244, 1090, 757 cm^{-1}. MS (ESI) *m*/*z* (relative intensity): 282 (100) $[M + Na]^+$, 260 (40) $[M + H]^+$. HR-MS (ESI): *m*/*z* calcd. for $[C_{15}H_{14}NOCl + Na]^+$ 282.0656 found 282.0661.

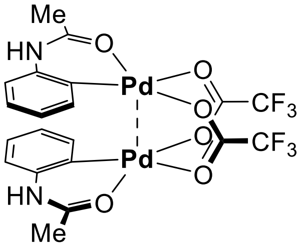

Dimeric organopalladium complex (54)

The product is known and the characterization is in consistence with that reported in the literature.[21] ^{1}H NMR (400 MHz, Acetone-d_6) δ 10.80 (s, 2H), 7.11 – 6.96 (m, 4H), 6.96 – 6.75 (m, 4H), 1.45 (s, 6H). ^{13}C NMR (101 MHz, Acetone-d_6) δ 167.1 (C_q), 165.0 (q, J = 37.1 Hz, C_q), 132.6 (CH), 131.1 (C_q), 125.4 (CH), 122.9 (CH), 116.0 (CH), 115.8 (C_q), 115.4 (q, J = 287.6 Hz, C_q), 19.3 (CH_3). ^{19}F NMR (377 MHz, Acetone-d_6) δ -75.2. IR (ATR): 3319, 1669, 1616, 1541, 1463, 1199, 1153, 854, 753, 732 cm^{-1}.

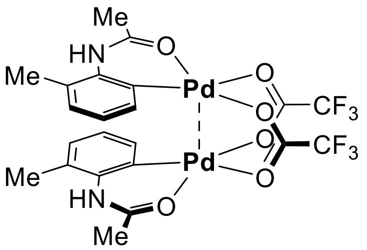

Dimeric organopalladium complex (55)

The product is known and the characterization is in consistence with that reported in the literature.[22] ^{1}H NMR (600 MHz, Acetone-d$_6$) δ 9.30 (s, 2H), 6.95 (d, *J* = 7.2 Hz, 2H), 6.90 (d, *J* = 8.0 Hz, 2H), 6.77 (t, *J* = 7.6 Hz, 2H), 2.39 (s, 6H), 1.59 (s, 6H). ^{13}C NMR (126 MHz, Acetone-d$_6$) δ 169.2 (C$_q$), 165.8 (q, *J* = 33.6 Hz, C$_q$), 131.5 (CH), 129.9 (C$_q$), 128.6 (CH), 124.3 (C$_q$), 123.6 (CH), 117.1 (C$_q$), 116.3 (q, *J* = 287.6 Hz, C$_q$), 20.5 (CH$_3$), 18.9 (CH$_3$). ^{19}F NMR (471 MHz, Acetone-d$_6$) δ -75.3. IR (ATR): 3440, 1659, 1618, 1537, 1445, 1192, 1149, 851, 766, 731 cm^{-1}. HR-MS (ESI): *m*/*z* calcd. for $[C_{22}H_{20}F_6N_2O_6Pd_2 + Na]^+$ 755.9204 found 755.9193.

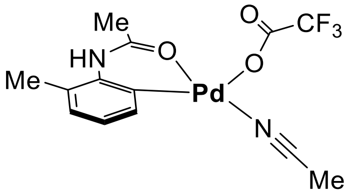

Monomeric organopalladium complex (56)

^{1}H NMR (400 MHz, Acetone-d$_6$) δ 9.35 (s, 1H), 6.95 (d, *J* = 7.1 Hz, 1H), 6.90 (d, *J* = 8.0 Hz, 1H), 6.77 (t, *J* = 7.6 Hz, 1H), 2.38 (s, 3H), 2.05 (s, 3H), 1.59 (s, 3H). ^{13}C NMR (101 MHz, Acetone-d$_6$) δ 169.3 (C$_q$), 165.8 (q, *J* = 38.1 Hz, C$_q$), 131.5 (CH), 130.0 (C$_q$), 128.6 (CH), 124.4 (C$_q$), 123.7 (CH), 117.7 (C$_q$), 117.2 (C$_q$), 116.3 (q, *J* = 287.7 Hz, C$_q$), 20.6 (CH$_3$), 19.0 (CH$_3$), 1.1 (CH$_3$). ^{19}F NMR (377 MHz, Acetone-d$_6$) δ -70.0. IR (ATR): 3436, 2924, 1668, 1618, 1539, 1444, 1375, 1197, 1150, 852 cm^{-1}. HR-MS (ESI): *m*/*z* calcd. for $[C_{13}H_{13}F_3N_2O_3Pd – CO_2CF_3]^+$ 295.0062 found 295.0071.

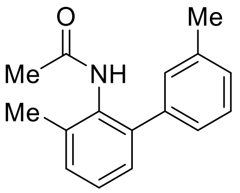

***N*-(3,3'-dimethyl-[1,1'-biphenyl]-2-yl)acetamide (57)**

The position-selectivity was determined by the ^{13}C NMR, *m* : *p* = 2 : 1. For *m*-**57**, ^{1}H NMR (400 MHz, $CDCl_3$) δ 7.53 – 7.00 (m, 7H), 6.62 (s, 1H), 2.39 (s, 3H), 2.31 (s, 3H), 2.01 (s, 3H). ^{13}C NMR (101 MHz, $CDCl_3$) δ 169.3 (C_q), 139.5 (C_q), 139.5 (C_q), 138.1 (C_q), 136.8 (C_q), 132.6 (C_q), 130.1 (CH), 129.7 (CH), 128.3 (CH), 128.2 (CH), 127.9 (CH), 127.4 (CH), 126.0 (CH), 23.1 (CH_3), 21.5 (CH_3), 18.7 (CH_3). IR (ATR): 3244, 3022, 2975, 2920, 1651, 1519, 1461, 1369, 1289, 776 cm^{-1}. MS (ESI) *m*/*z* (relative intensity): 262 (100) $[M + Na]^+$, 240 (10) $[M + H]^+$. HR-MS (ESI): *m*/*z* calcd. for $[C_{16}H_{17}NO + Na]^+$ 262.1202 found 262.1208.

13 NMR Spectrum

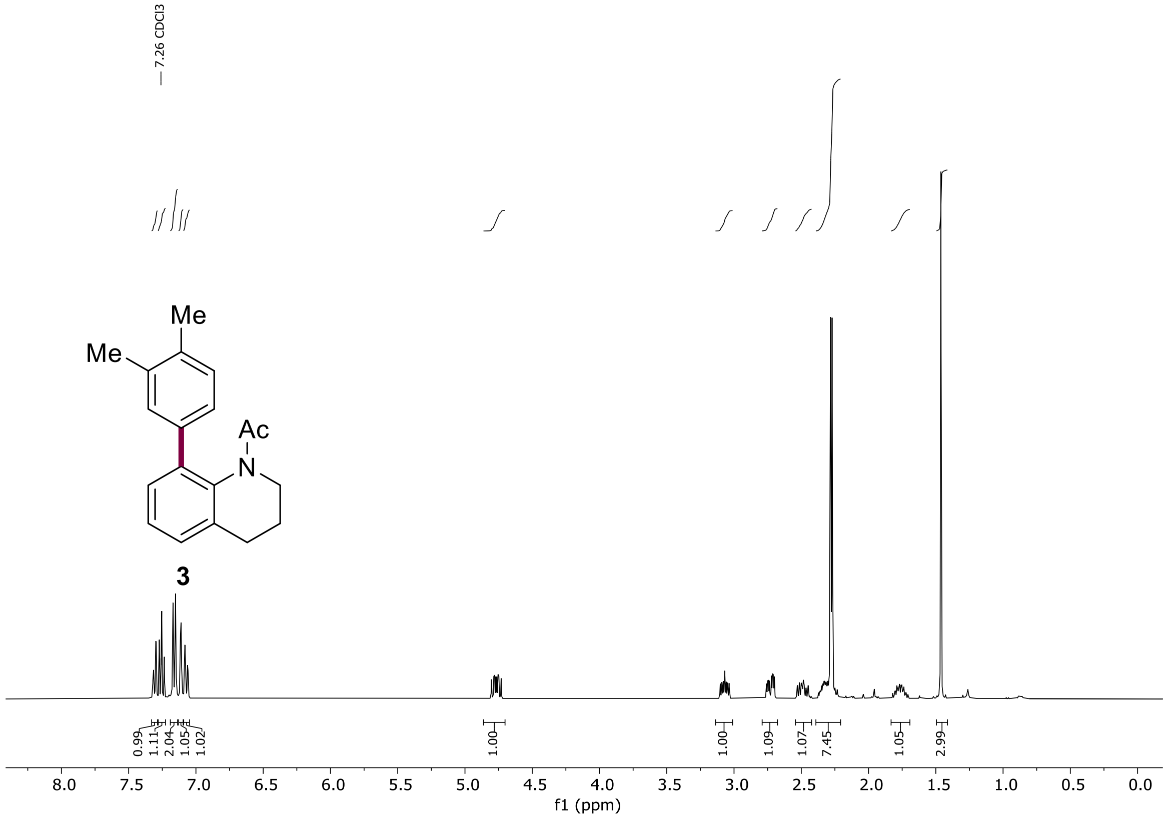

NMR Spectrum 1 ^{1}H **NMR for 3, 400 MHz,** $CDCl_3$**, room temperature.**

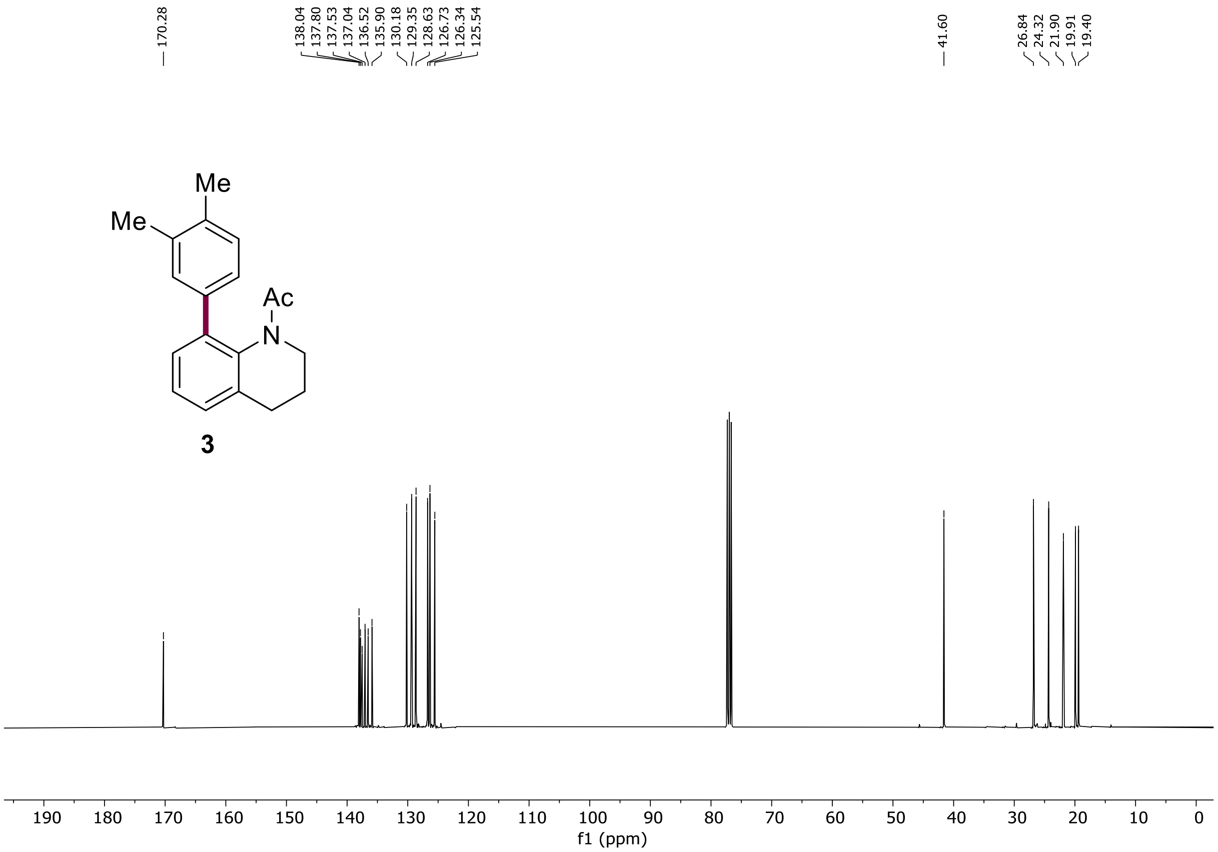

NMR Spectrum 2 ^{13}C **NMR for 3, 101 MHz,** $CDCl_3$**, room temperature.**

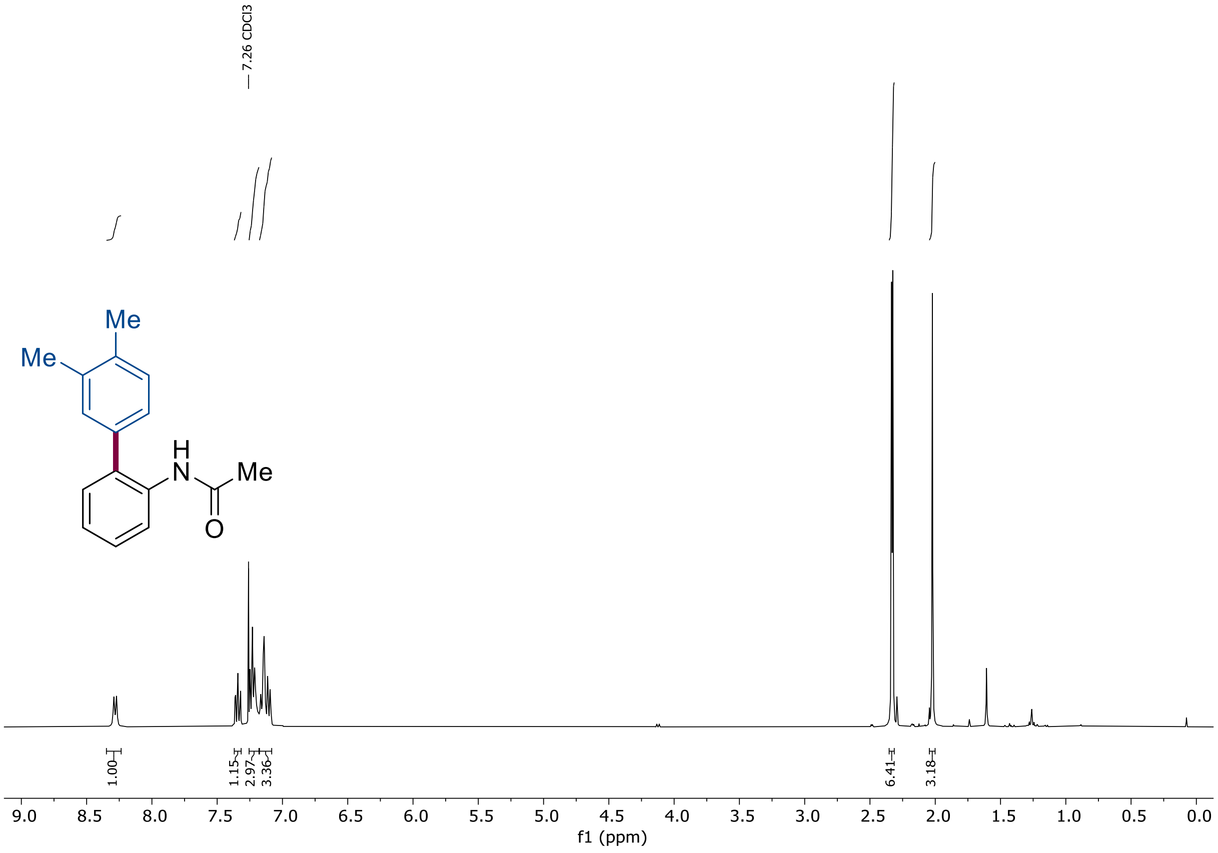

NMR Spectrum 3 ^{1}H NMR for 4, 400 MHz, $CDCl_3$, room temperature.

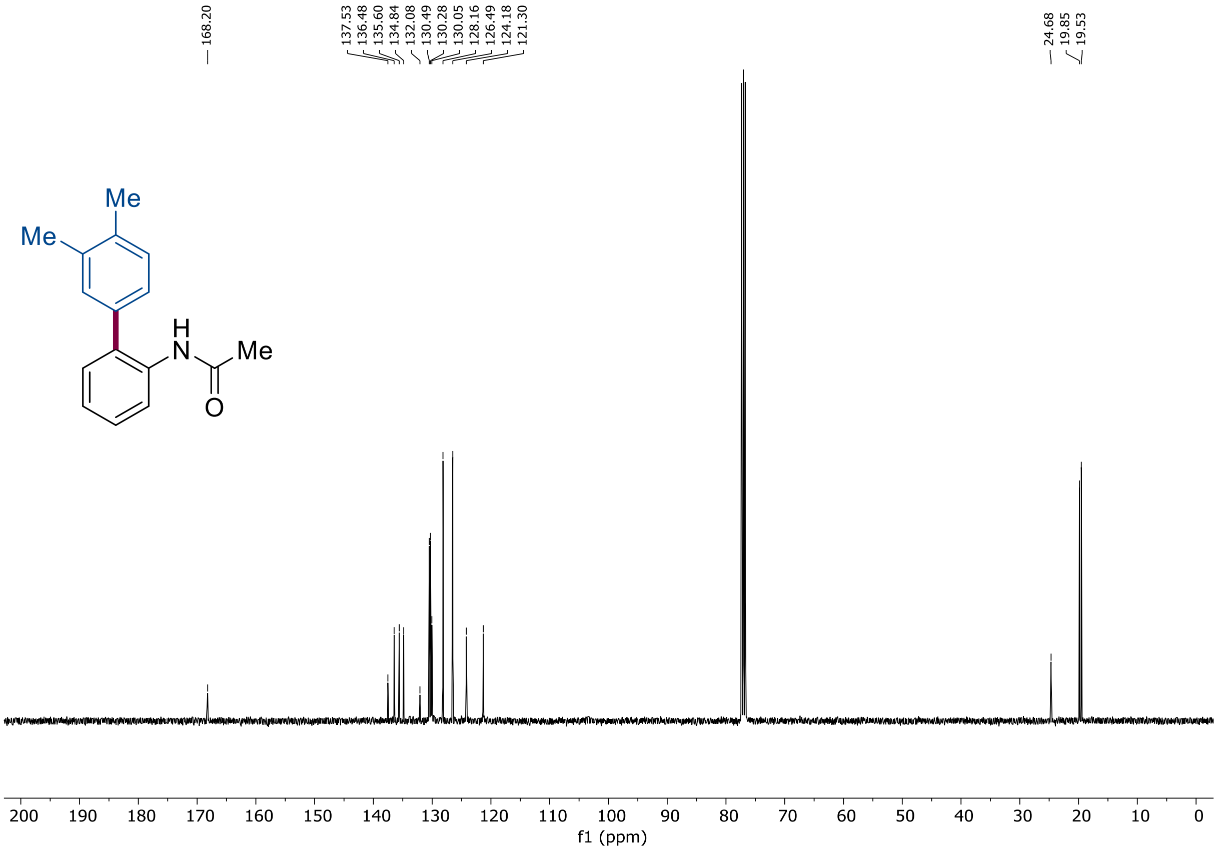

NMR Spectrum 4 ^{13}C NMR for 4, 101 MHz, $CDCl_3$, room temperature.

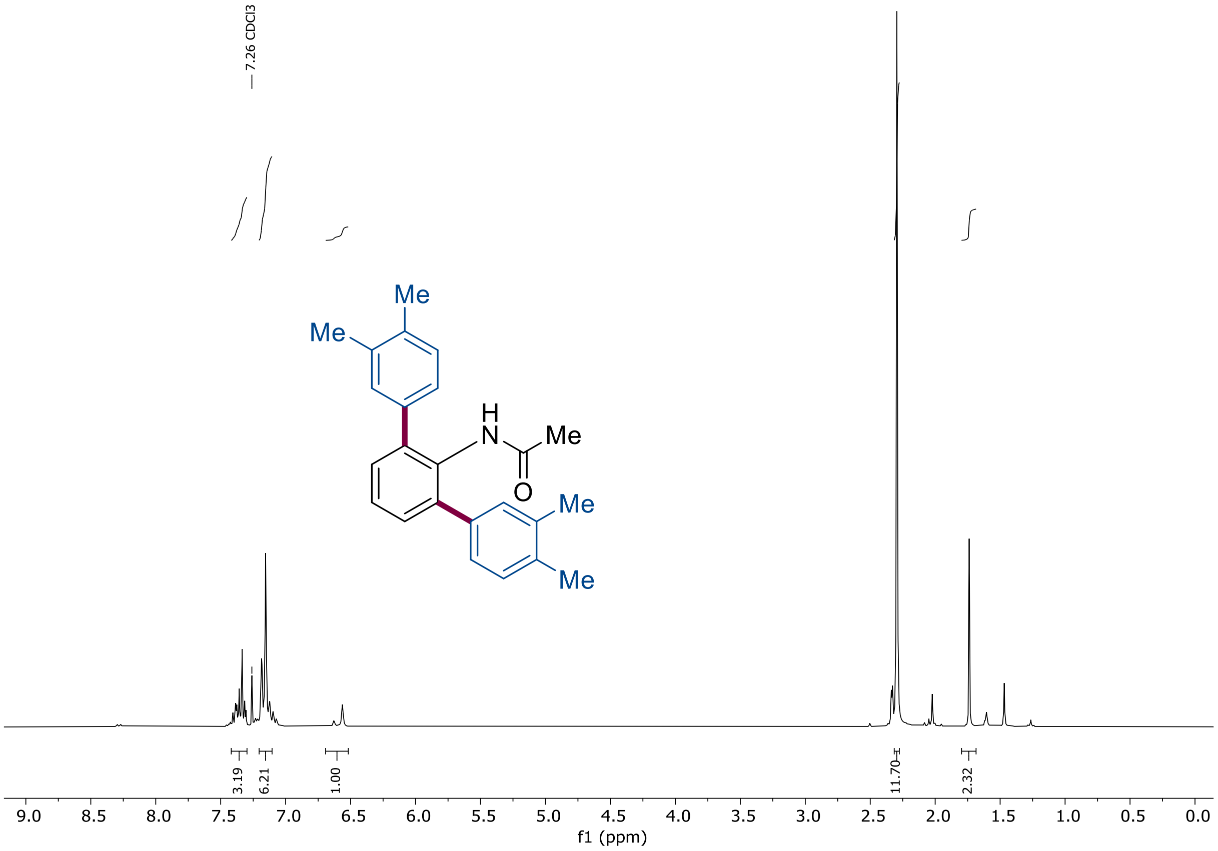

NMR Spectrum 5 ^{1}H NMR for di-4, 300 MHz, $CDCl_3$, room temperature.

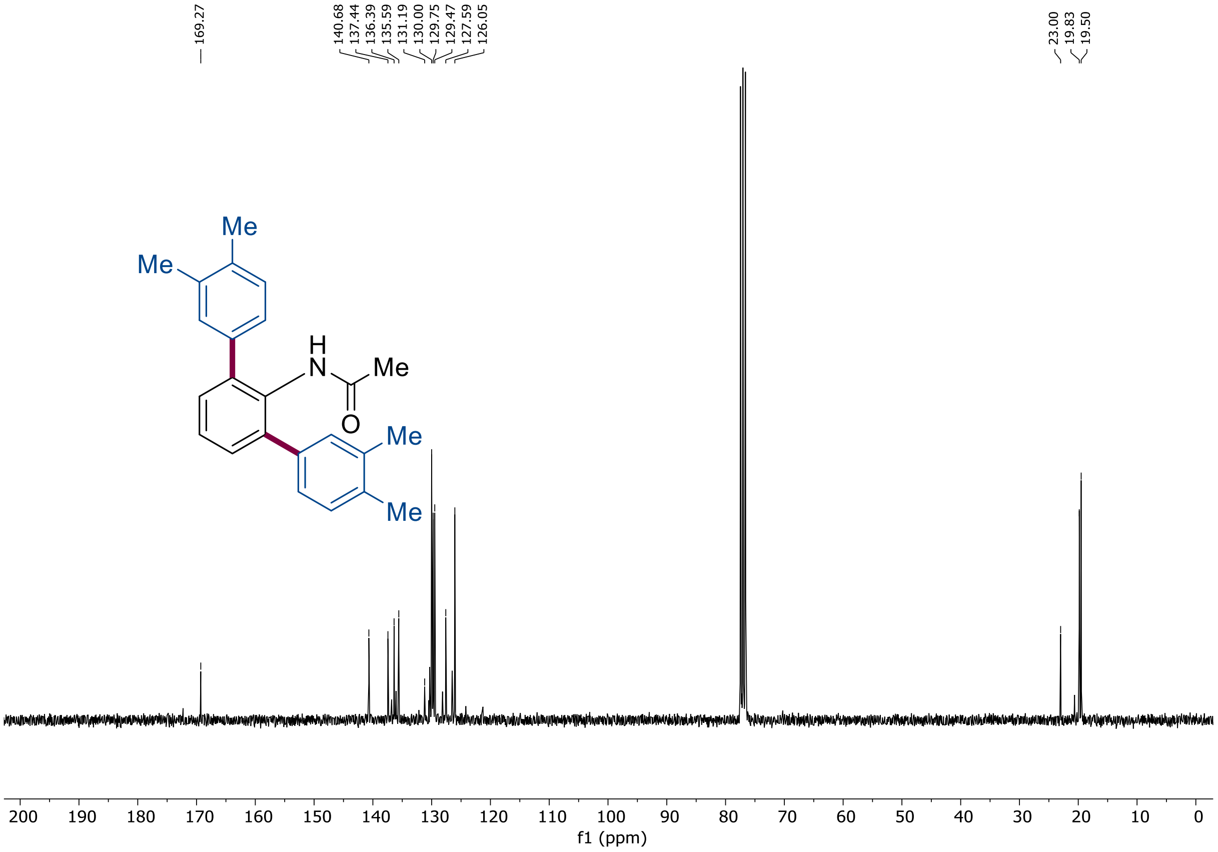

NMR Spectrum 6 ^{13}C NMR for di-4, 75 MHz, $CDCl_3$, room temperature.

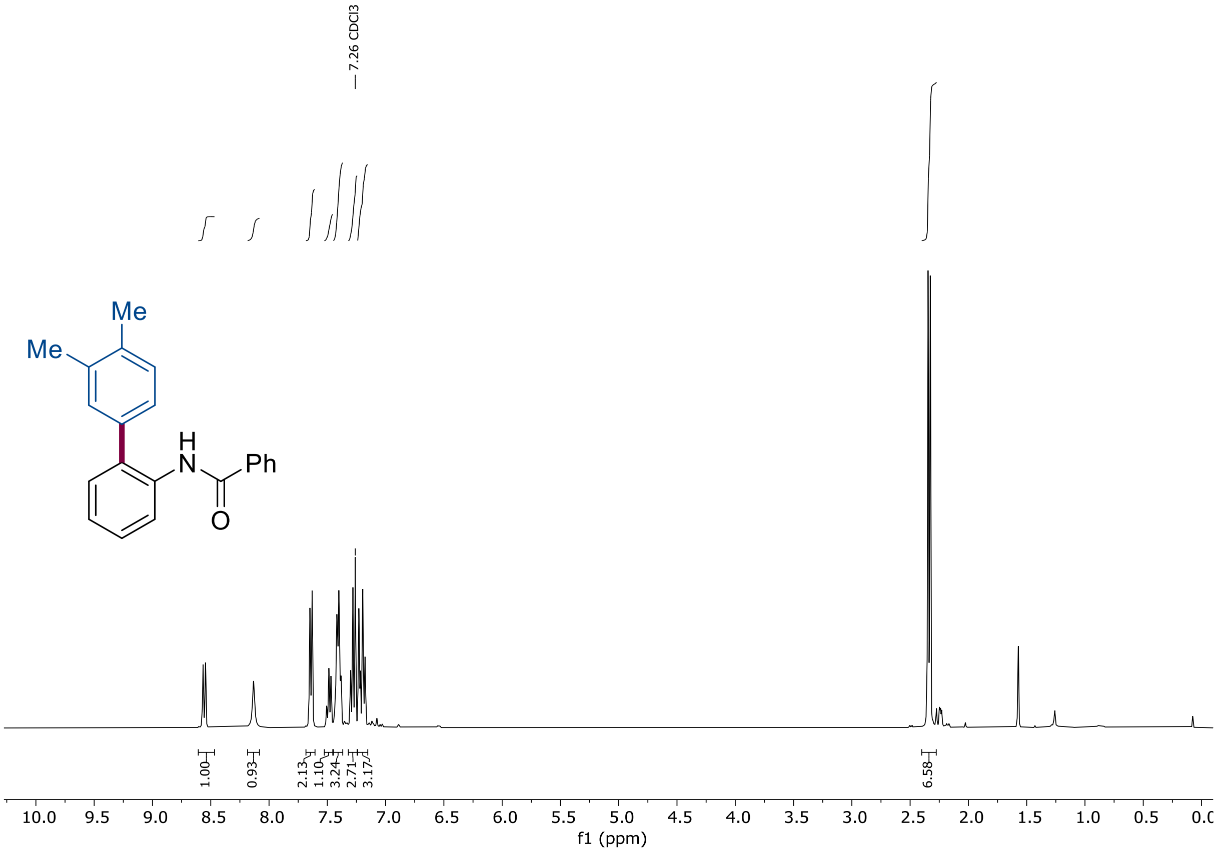

NMR Spectrum 7 ^{1}H NMR for 5, 400 MHz, $CDCl_3$, room temperature.

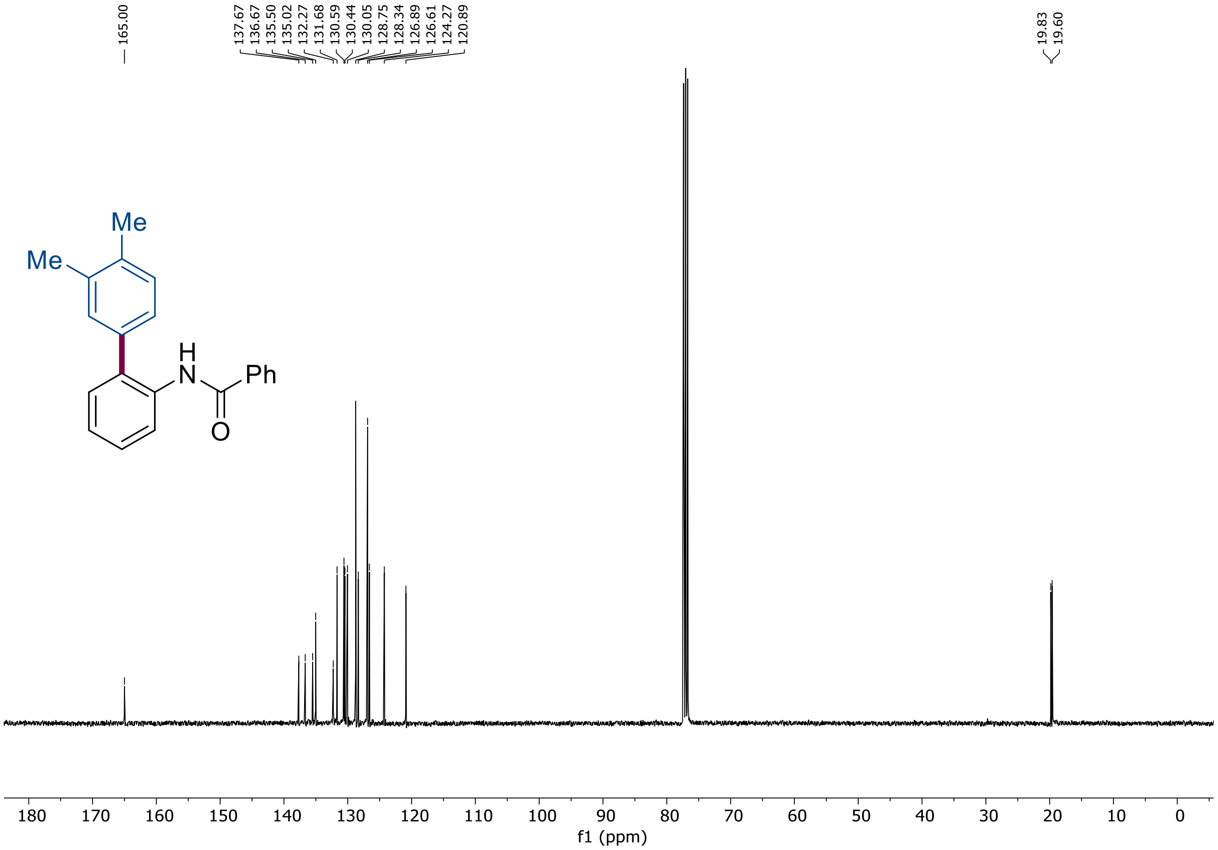

NMR Spectrum 8 ^{13}C NMR for 5, 101 MHz, $CDCl_3$, room temperature.

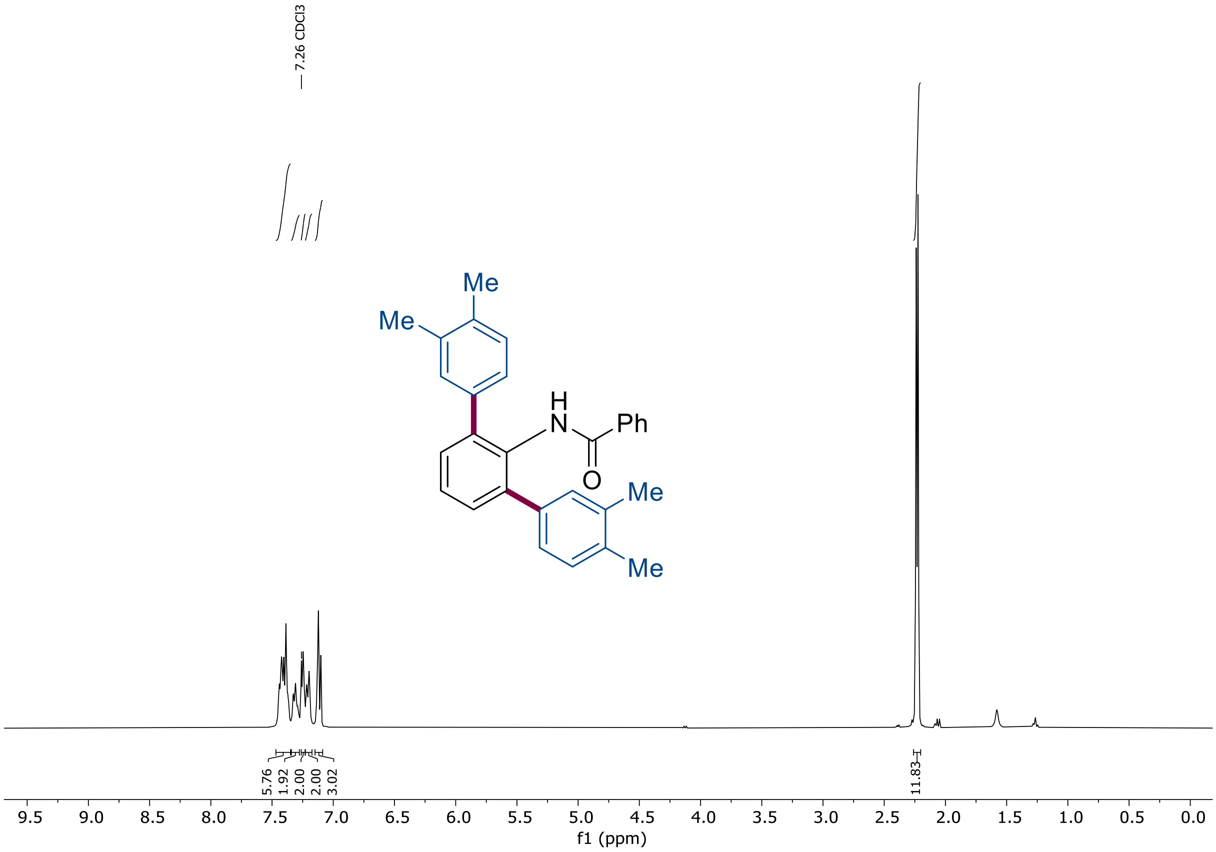

NMR Spectrum 9 ^{1}H NMR for di-5, 400 MHz, $CDCl_3$, room temperature.

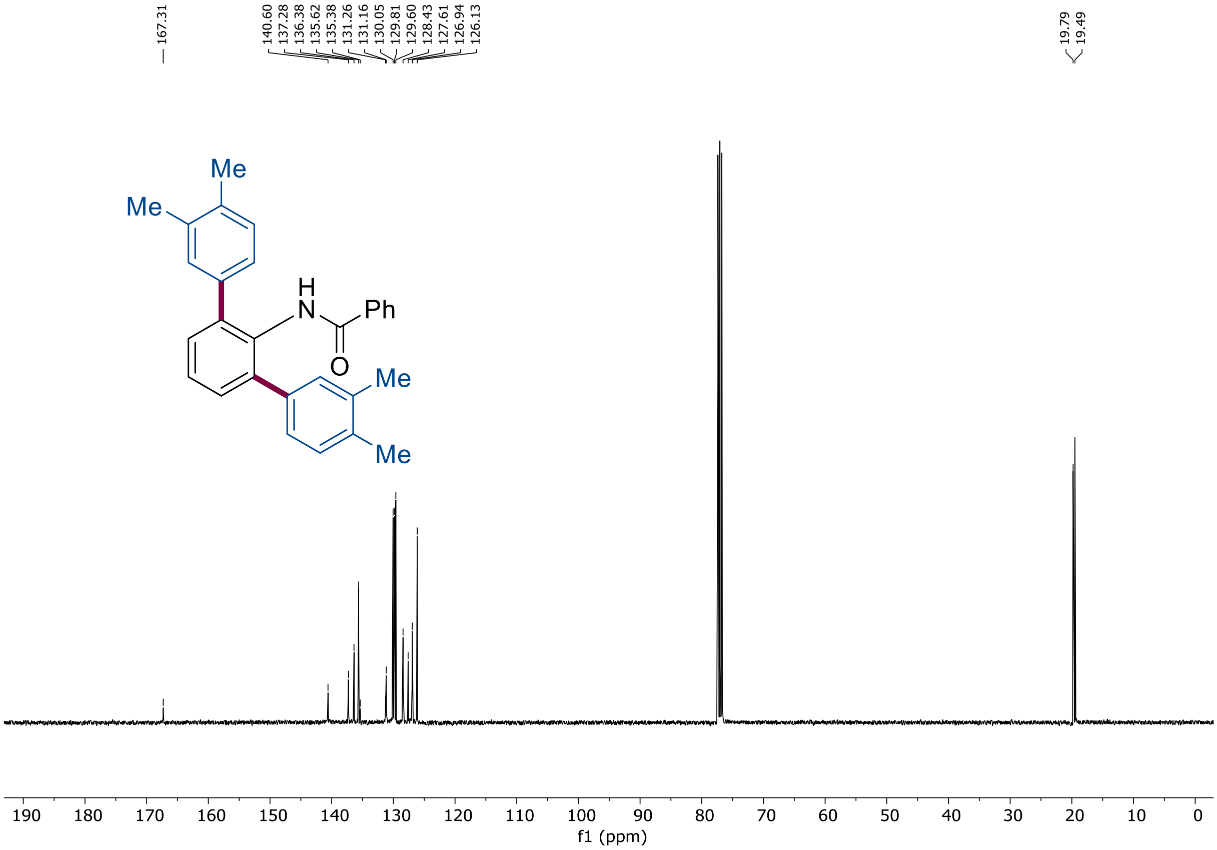

NMR Spectrum 10 ^{13}C NMR for di-5, 101 MHz, $CDCl_3$, room temperature.

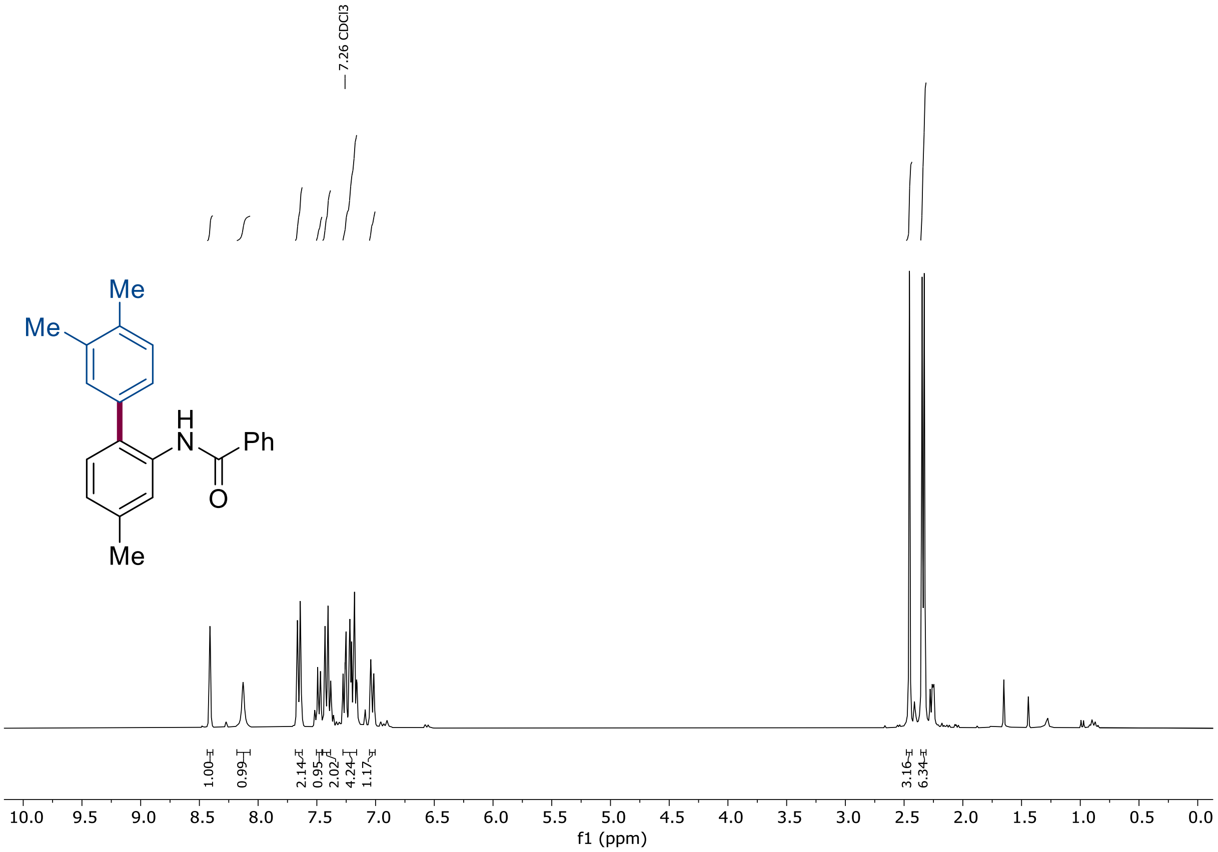

NMR Spectrum 11 ^{1}H **NMR for 6, 300 MHz,** $CDCl_3$**, room temperature.**

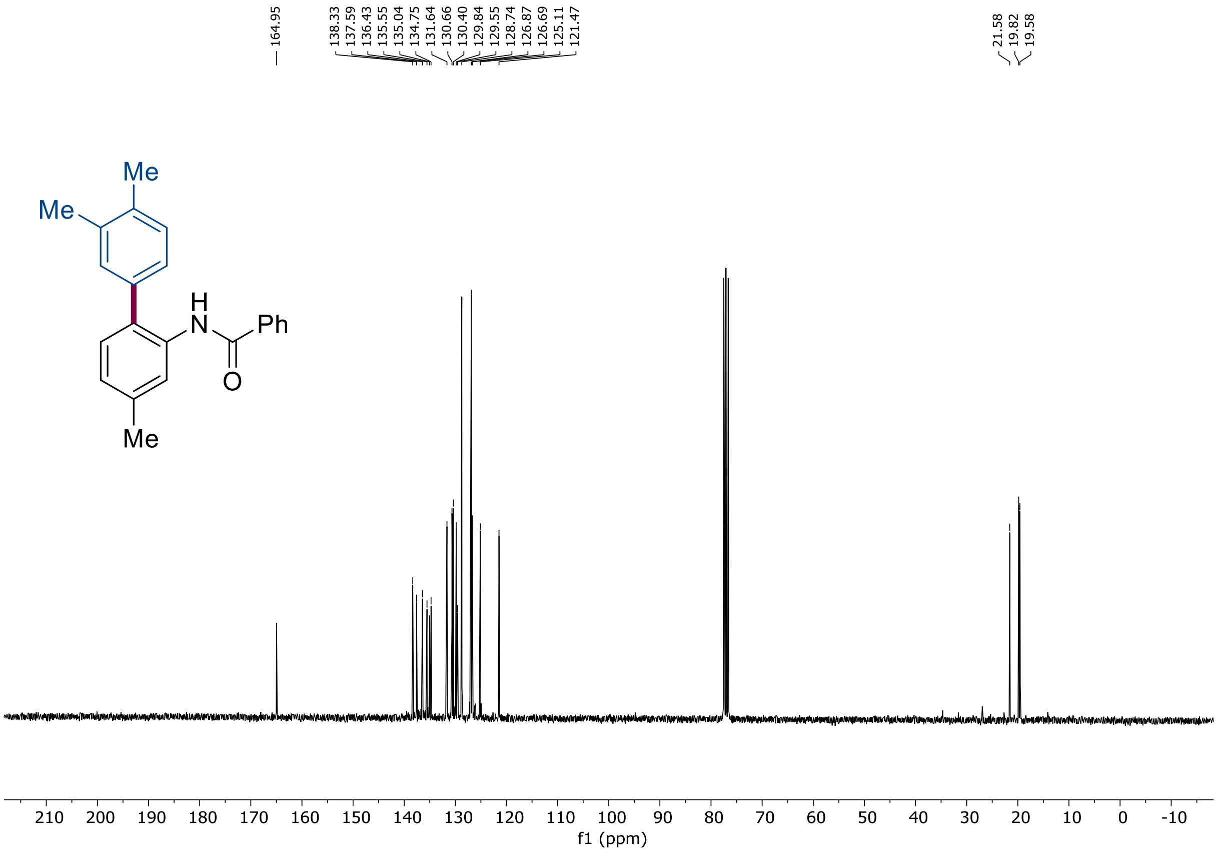

NMR Spectrum 12 ^{13}C **NMR for 6, 75 MHz,** $CDCl_3$**, room temperature.**

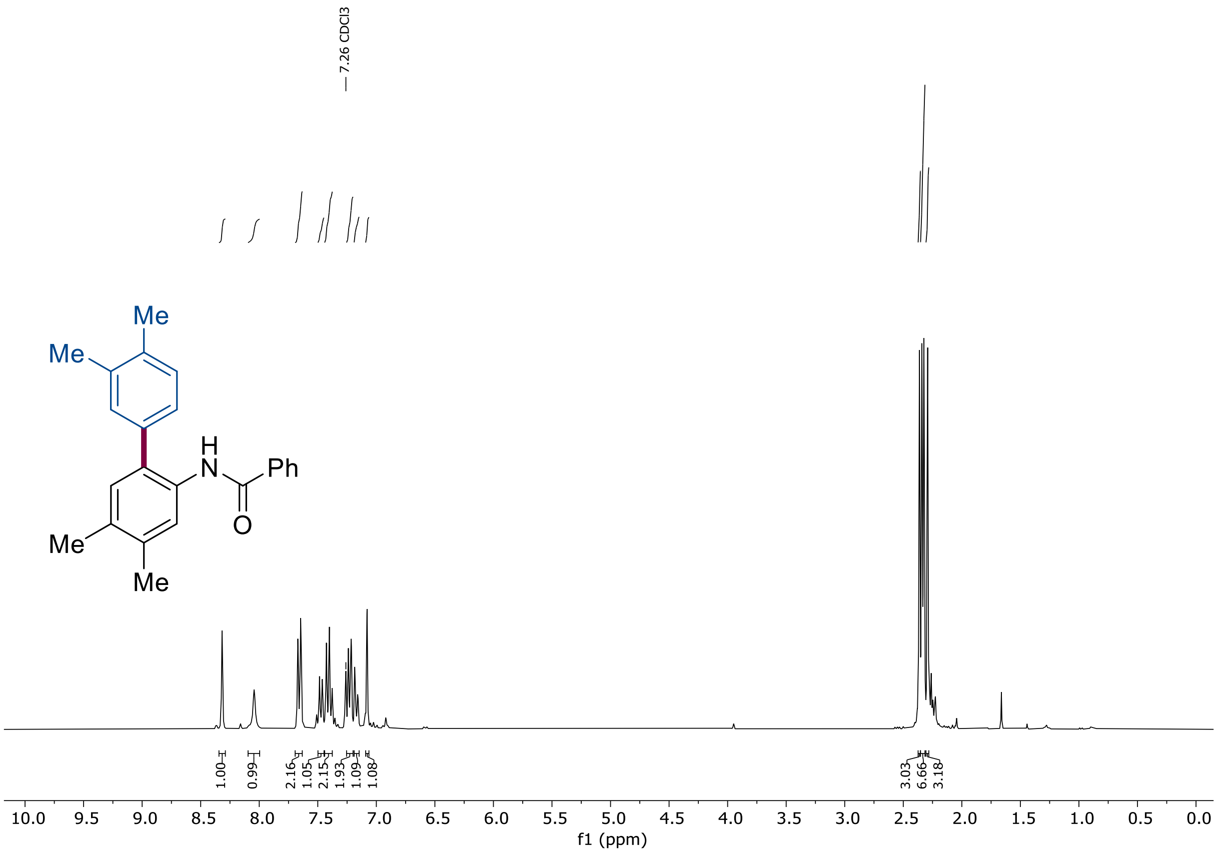

NMR Spectrum 13 ^{1}H NMR for 7, 300 MHz, CDCl$_3$, room temperature.

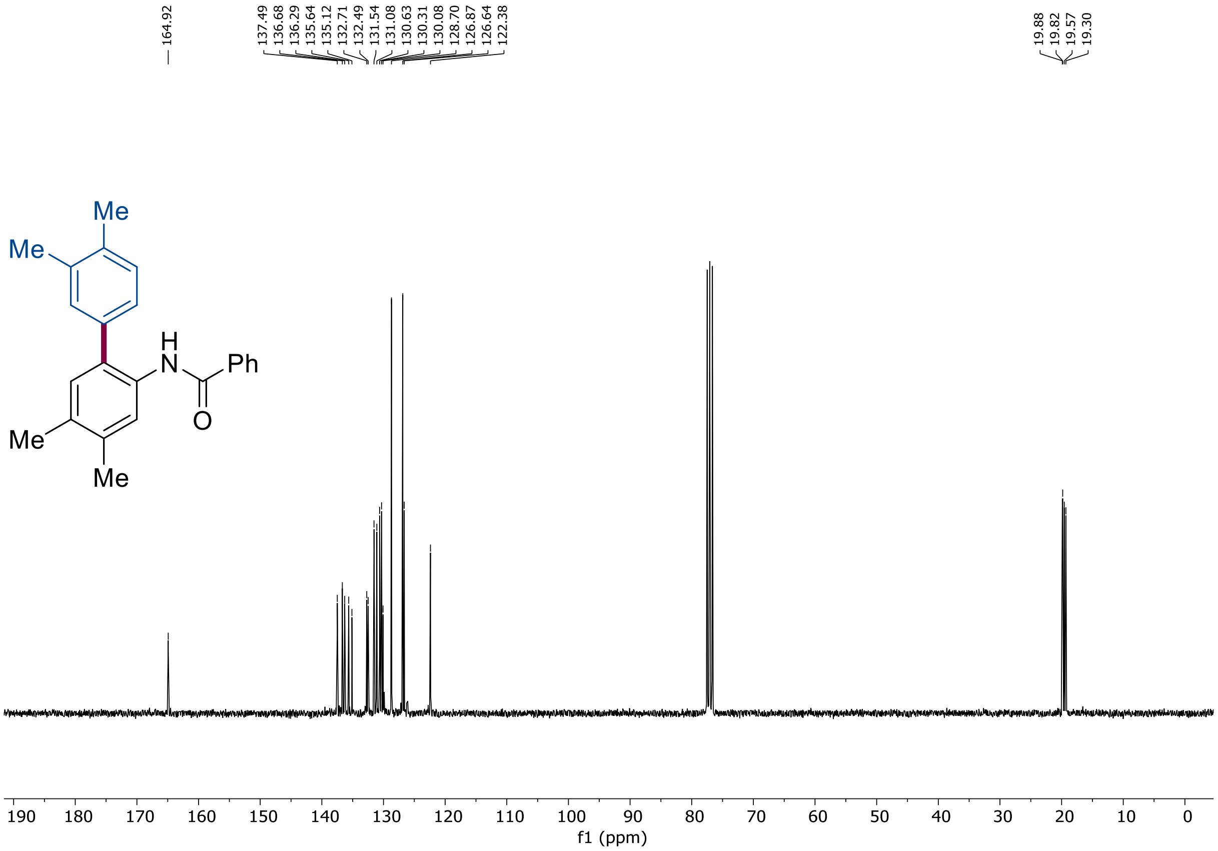

NMR Spectrum 14 ^{13}C NMR for 7, 75 MHz, CDCl$_3$, room temperature.

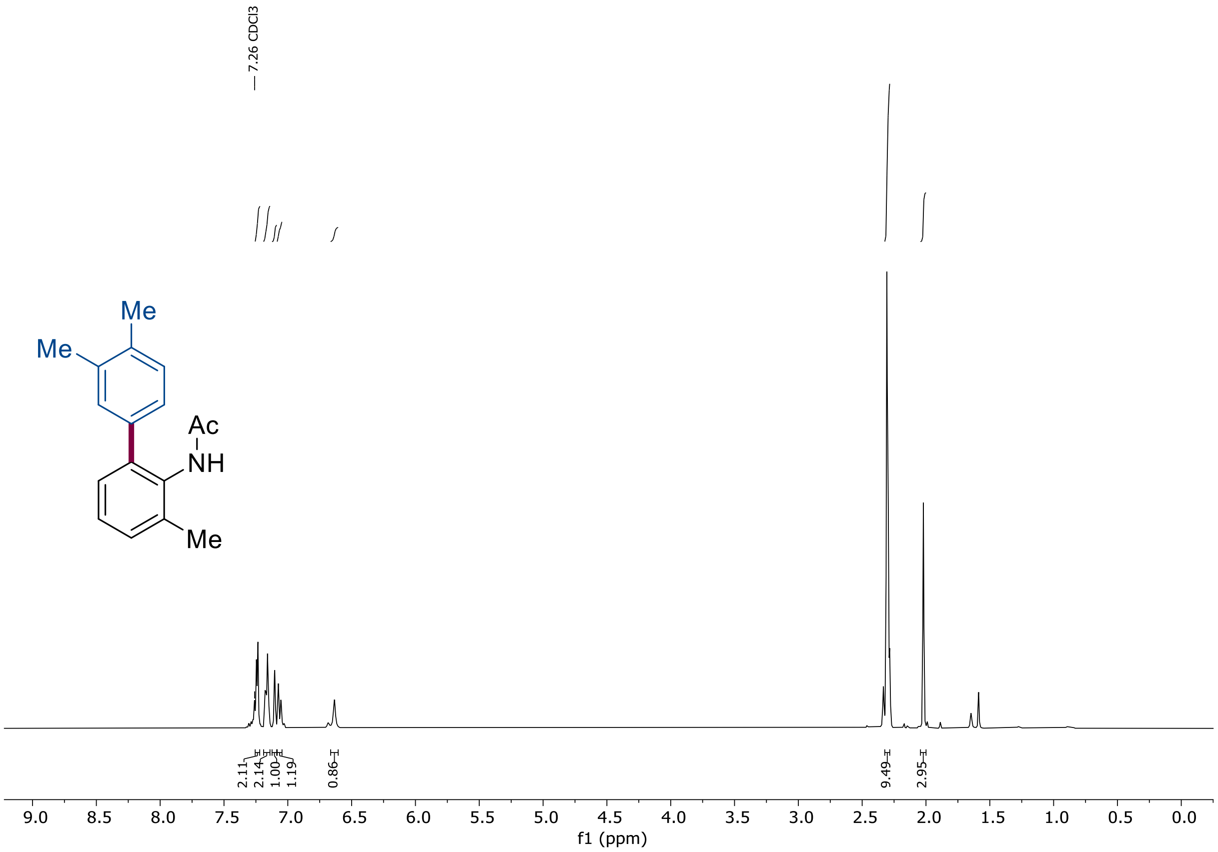

NMR Spectrum 15 ^{1}H NMR for 8, 400 MHz, $CDCl_3$, room temperature.

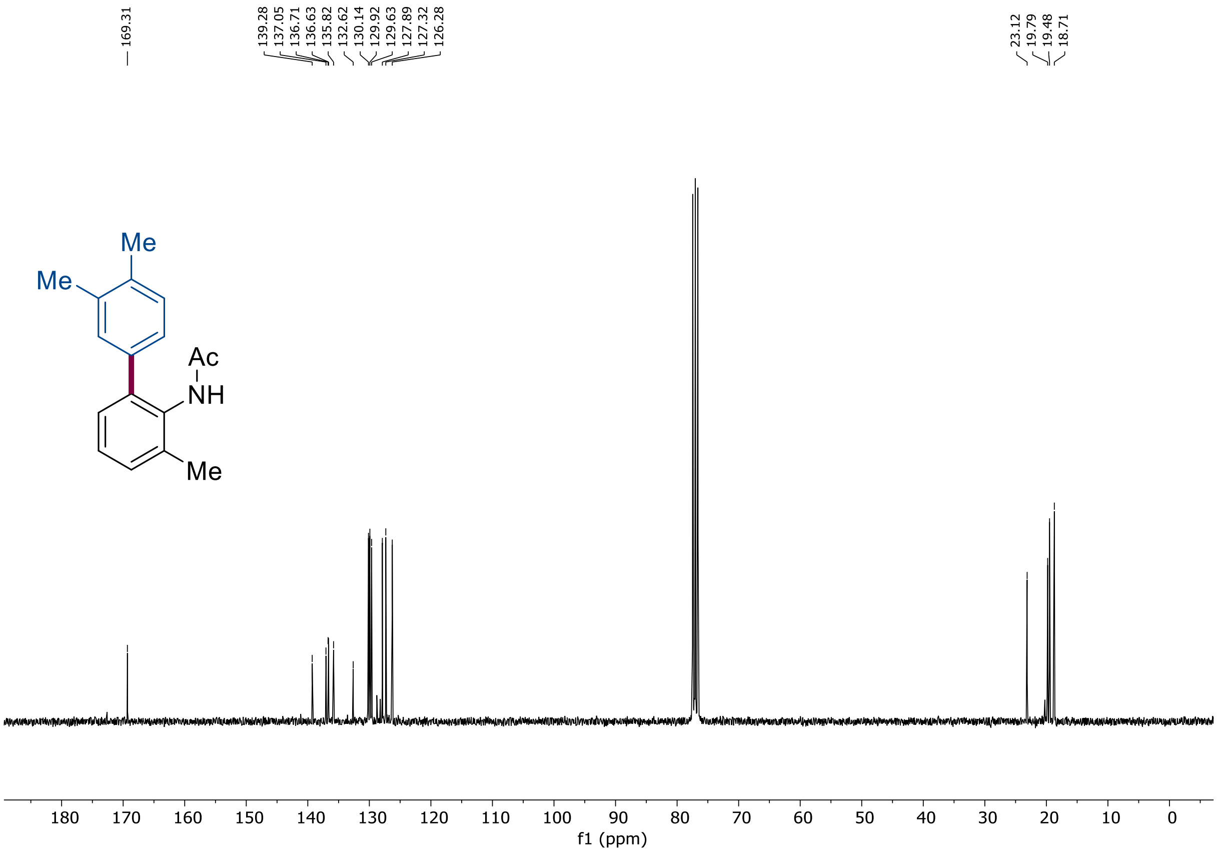

NMR Spectrum 16 ^{13}C NMR for 8, 75 MHz, $CDCl_3$, room temperature.

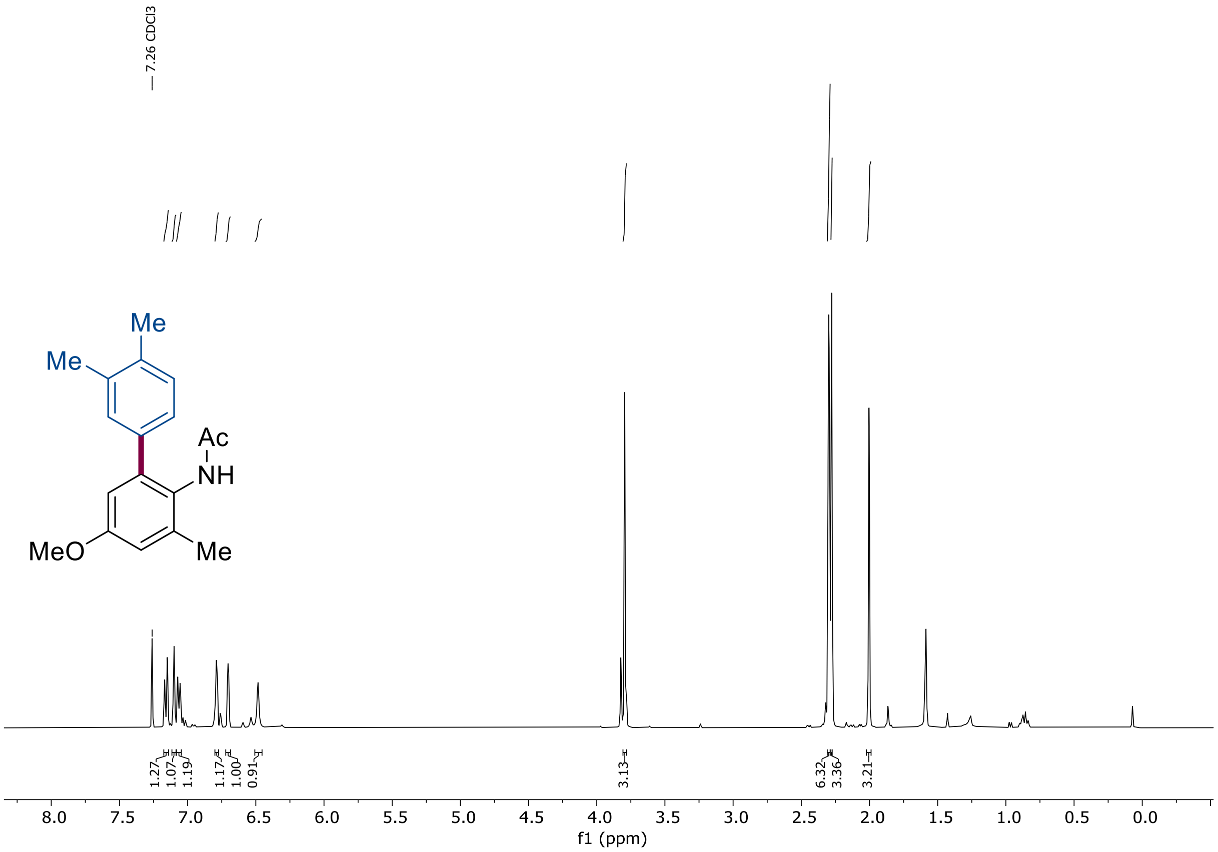

NMR Spectrum 17 ^{1}H NMR for 9, 400 MHz, $CDCl_3$, room temperature.

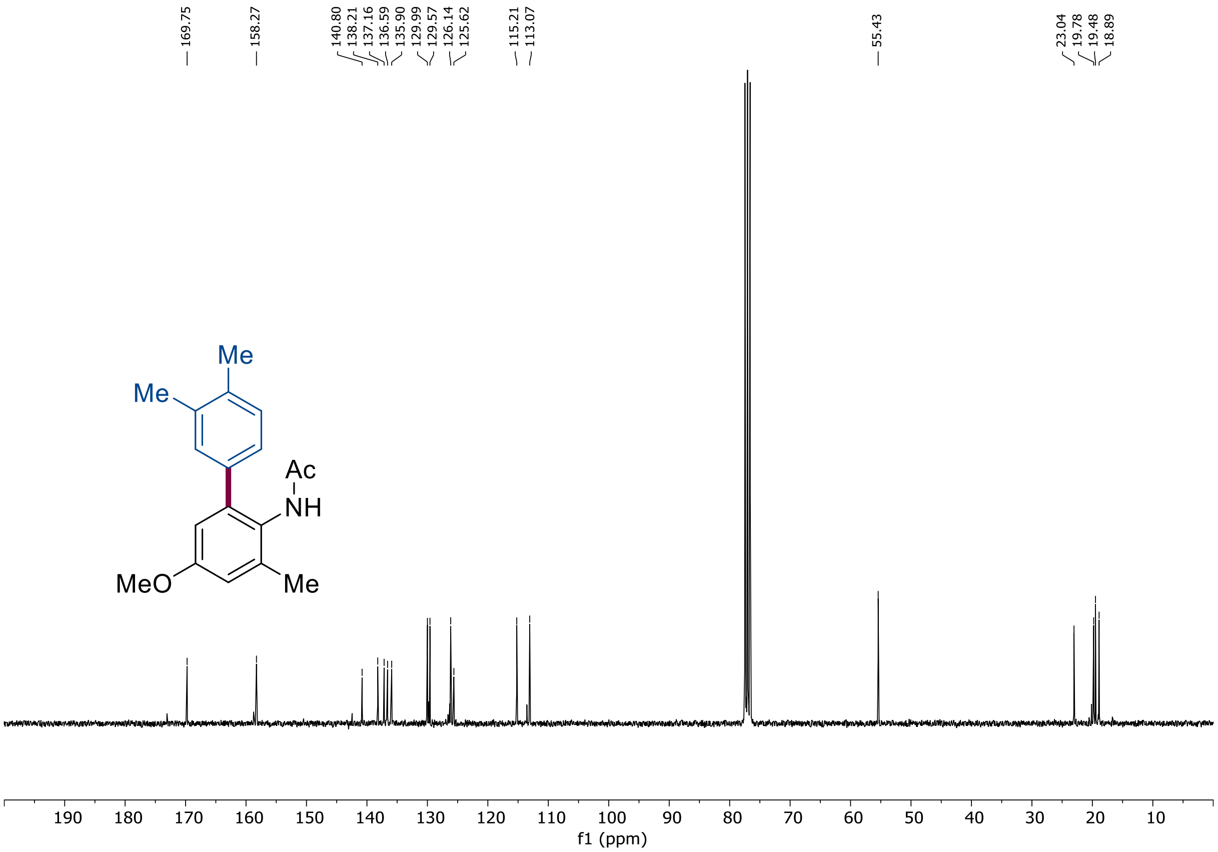

NMR Spectrum 18 ^{13}C NMR for 9, 75 MHz, $CDCl_3$, room temperature.

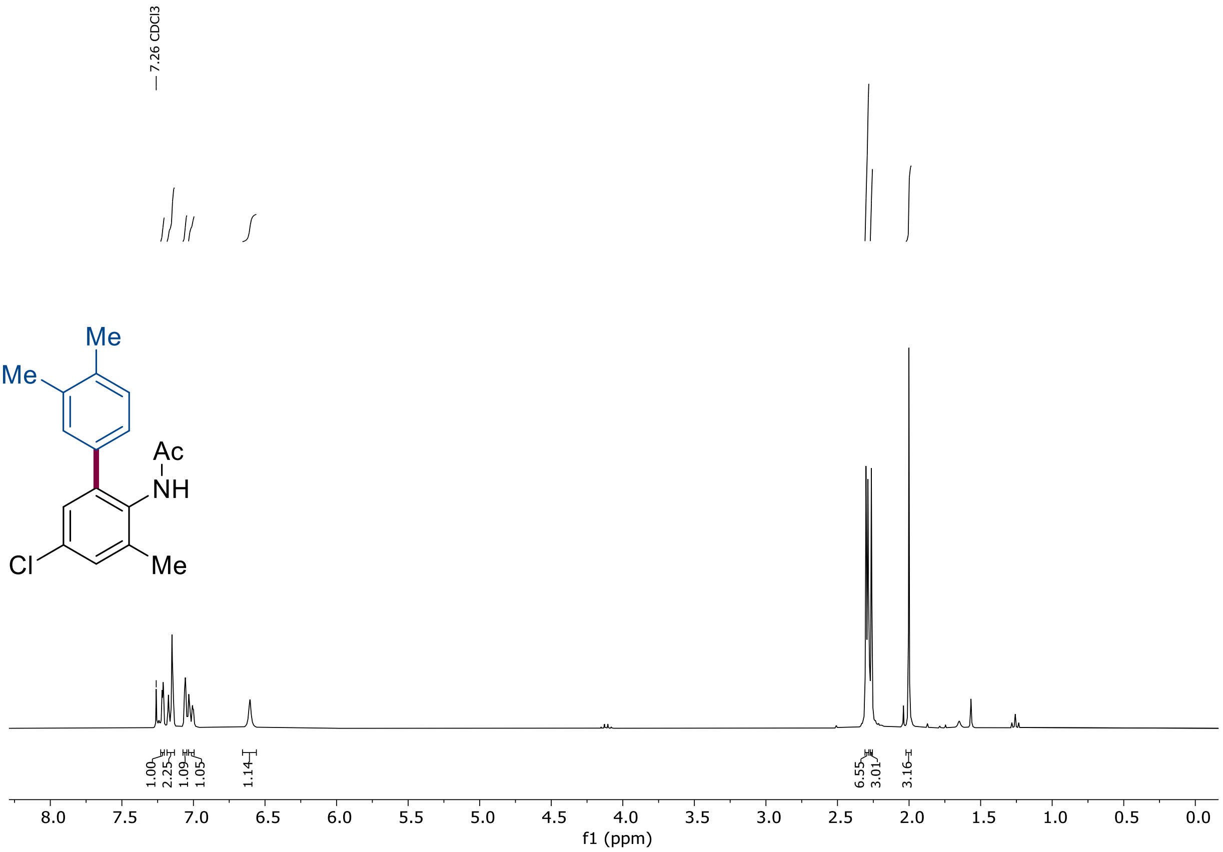

NMR Spectrum 19 ^{1}H NMR for 10, 300 MHz, CDCl$_3$, room temperature.

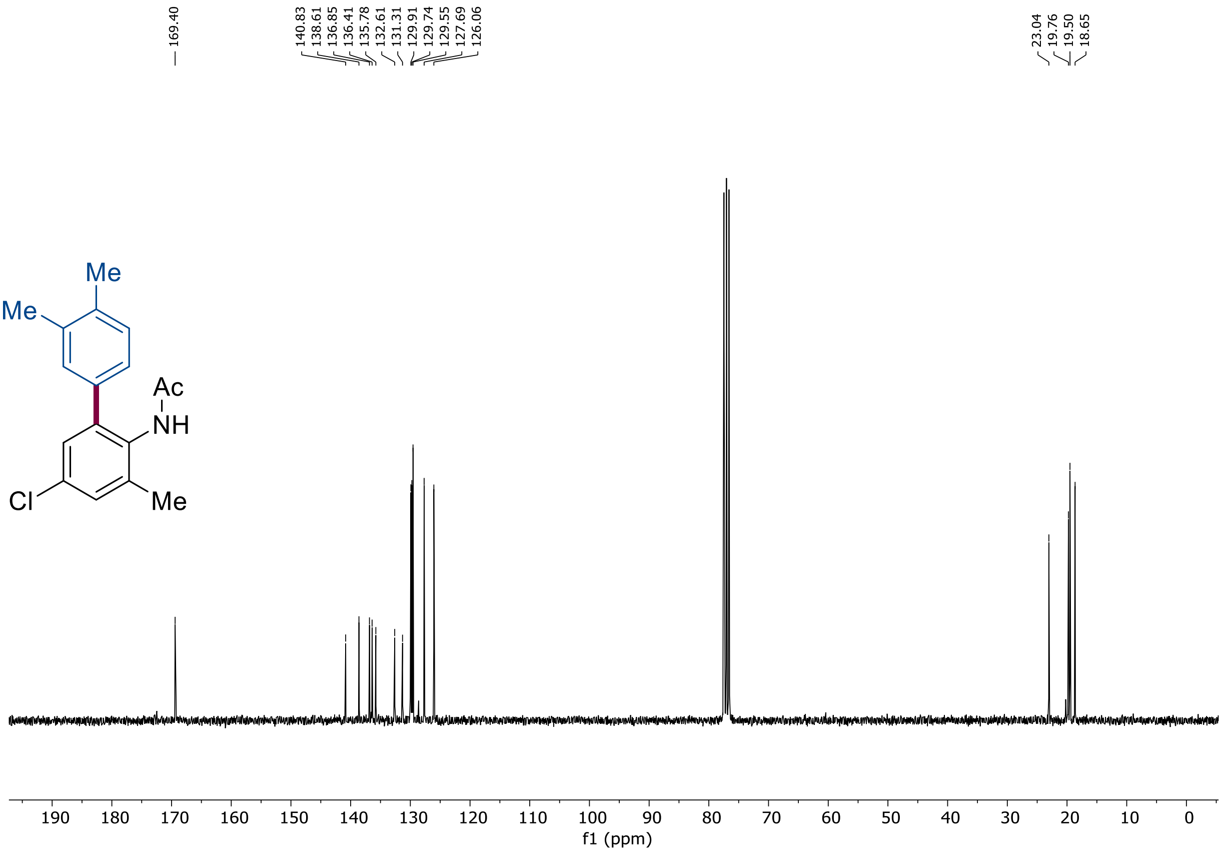

NMR Spectrum 20 ^{13}C NMR for 10, 75 MHz, CDCl$_3$, room temperature.

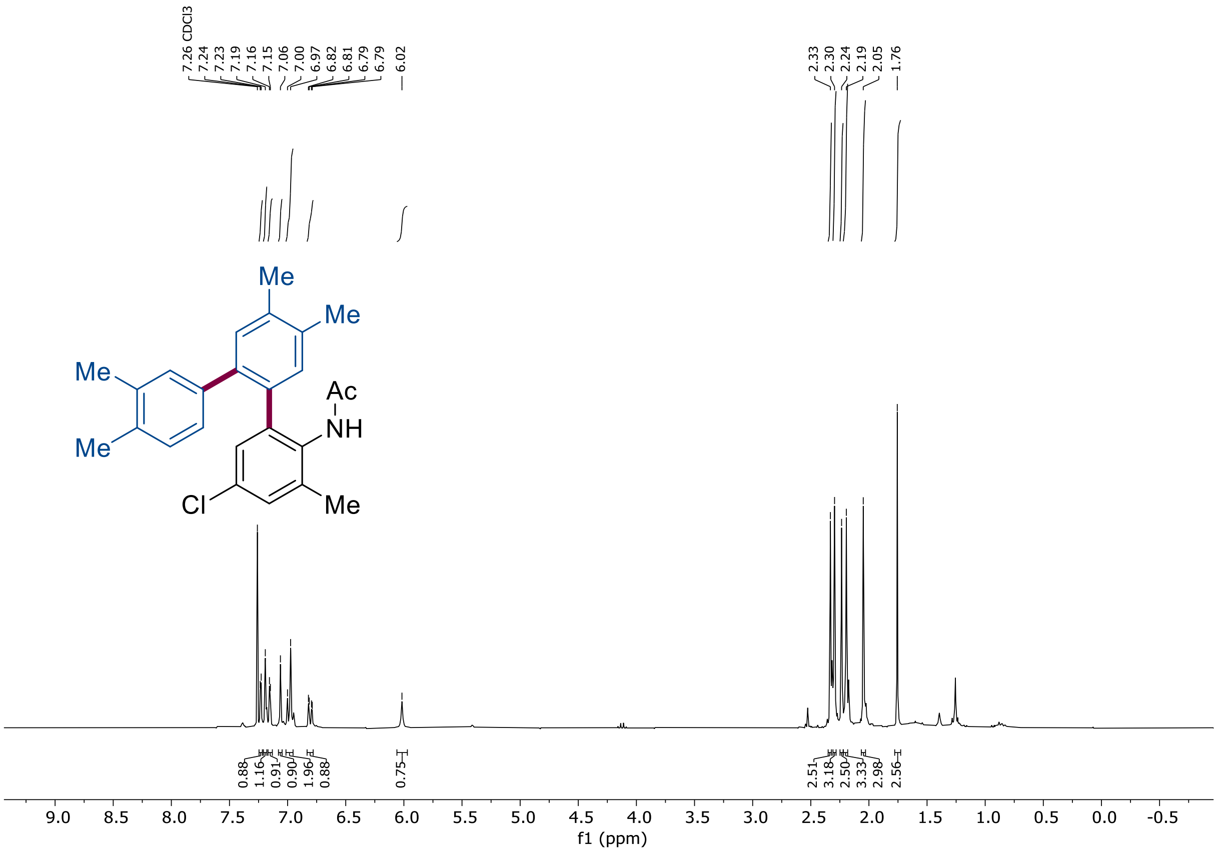

NMR Spectrum 21 ^{1}H **NMR for di-10, 300 MHz,** $CDCl_3$**, room temperature.**

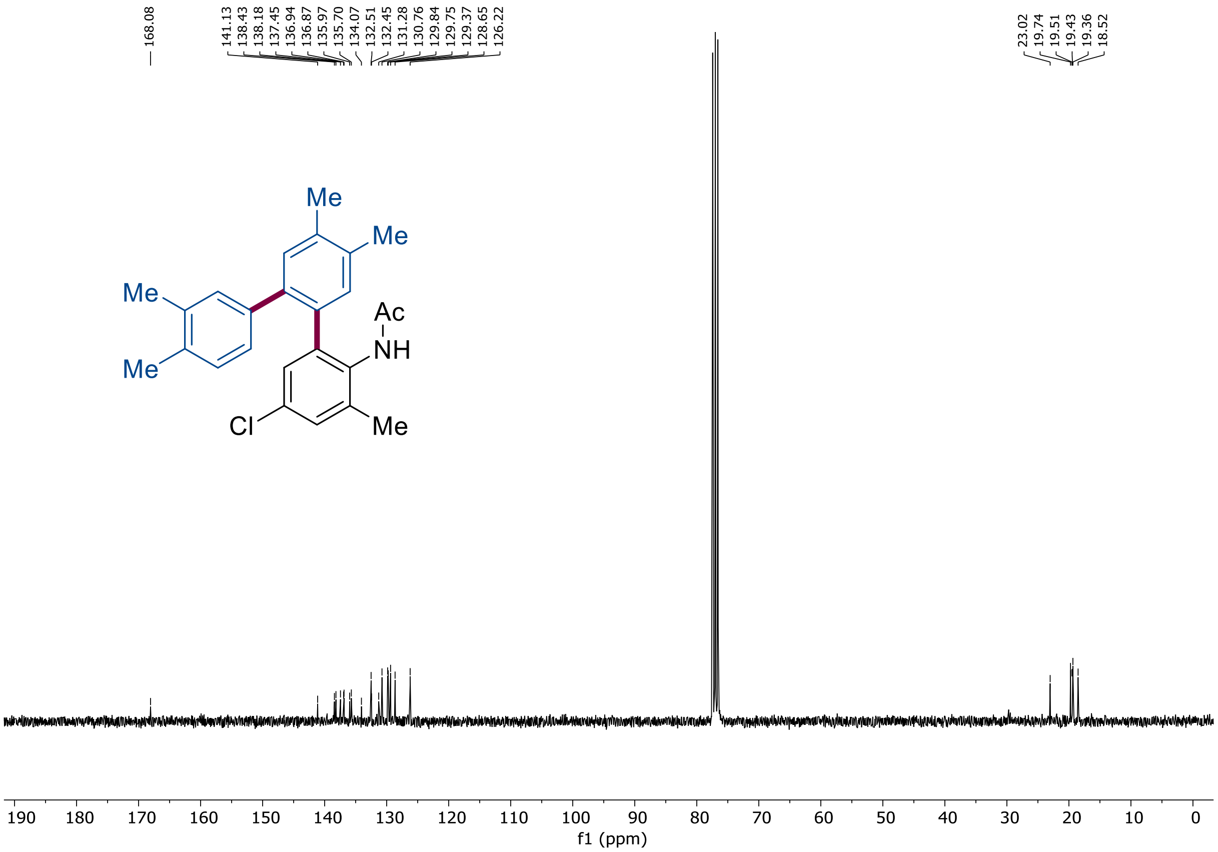

NMR Spectrum 22 ^{13}C **NMR for di-10, 75 MHz,** $CDCl_3$**, room temperature.**

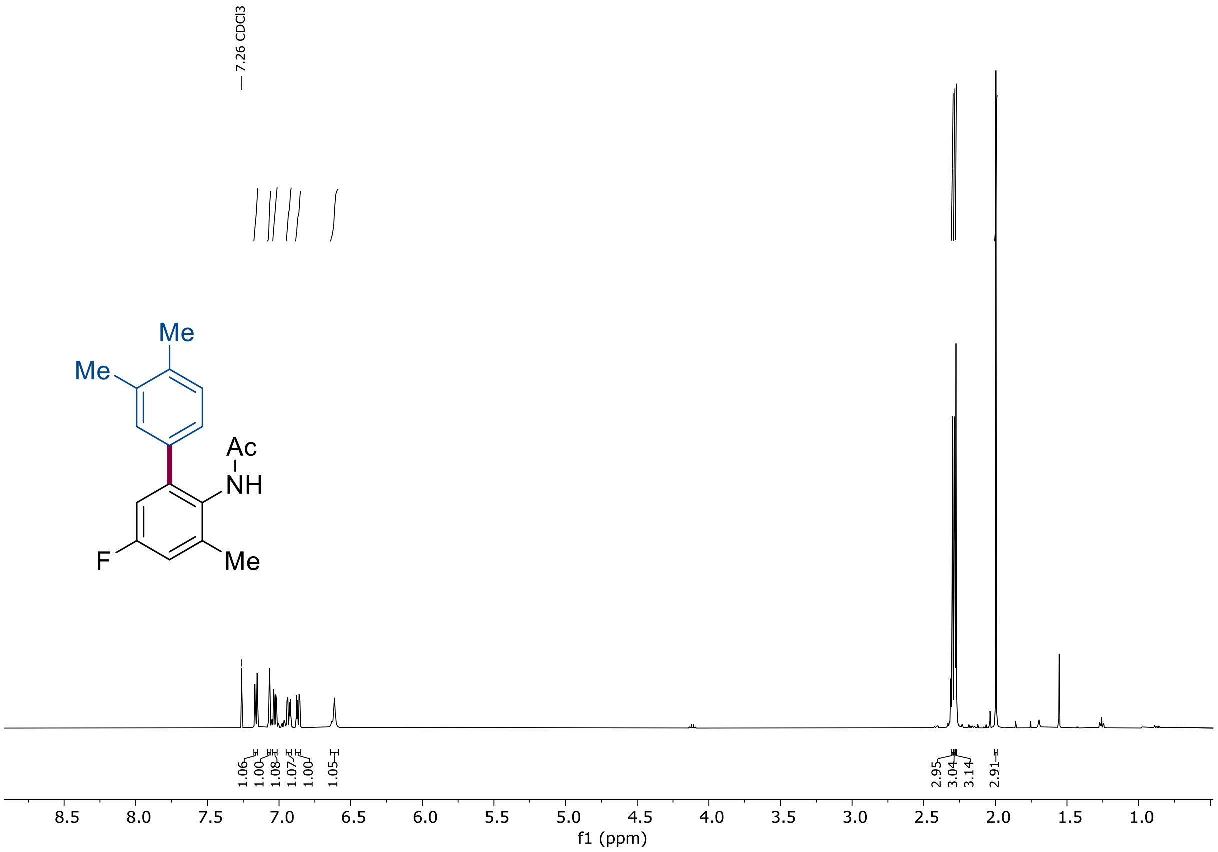

NMR Spectrum 23 ^{1}H NMR for 11, 500 MHz, $CDCl_3$, room temperature.

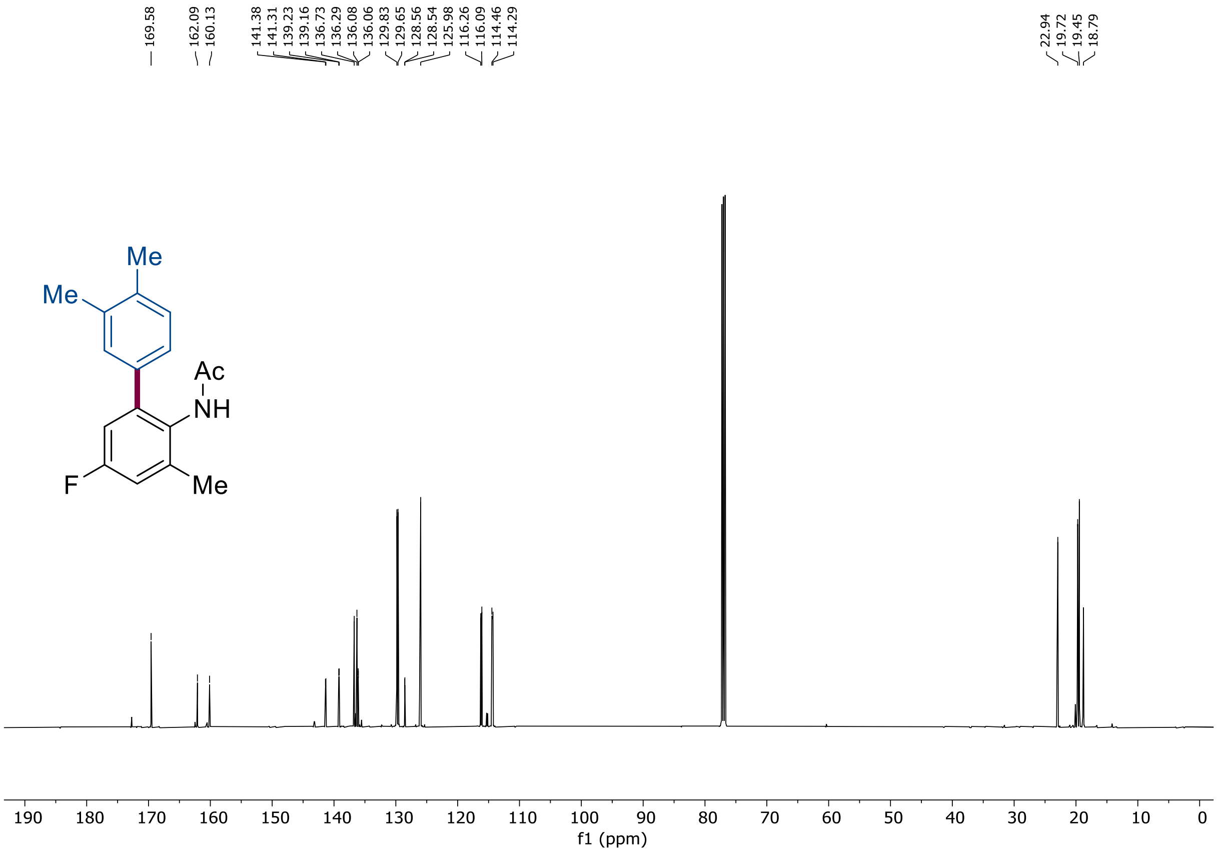

NMR Spectrum 24 ^{13}C NMR for 11, 101 MHz, $CDCl_3$, room temperature.

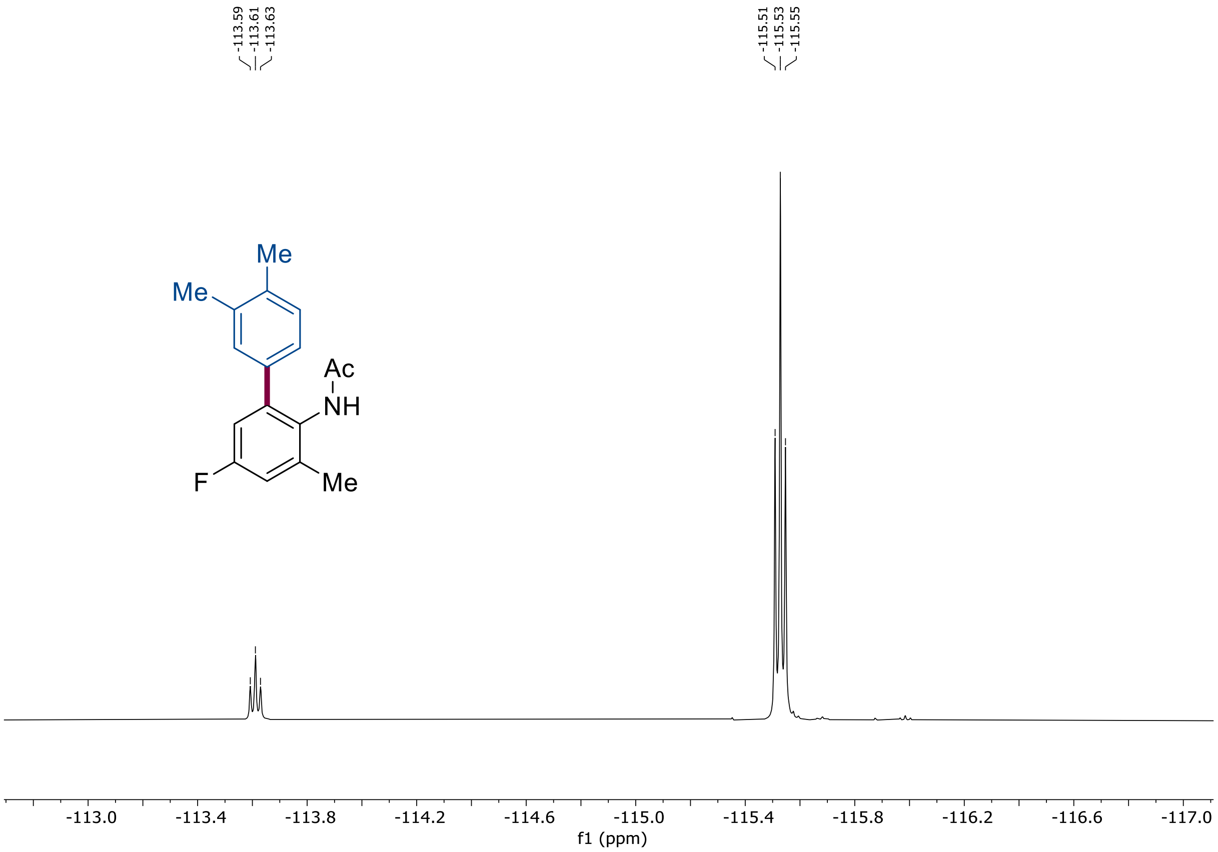

NMR Spectrum 25 ^{19}F NMR for 11, 471 MHz, CDCl$_3$, room temperature.

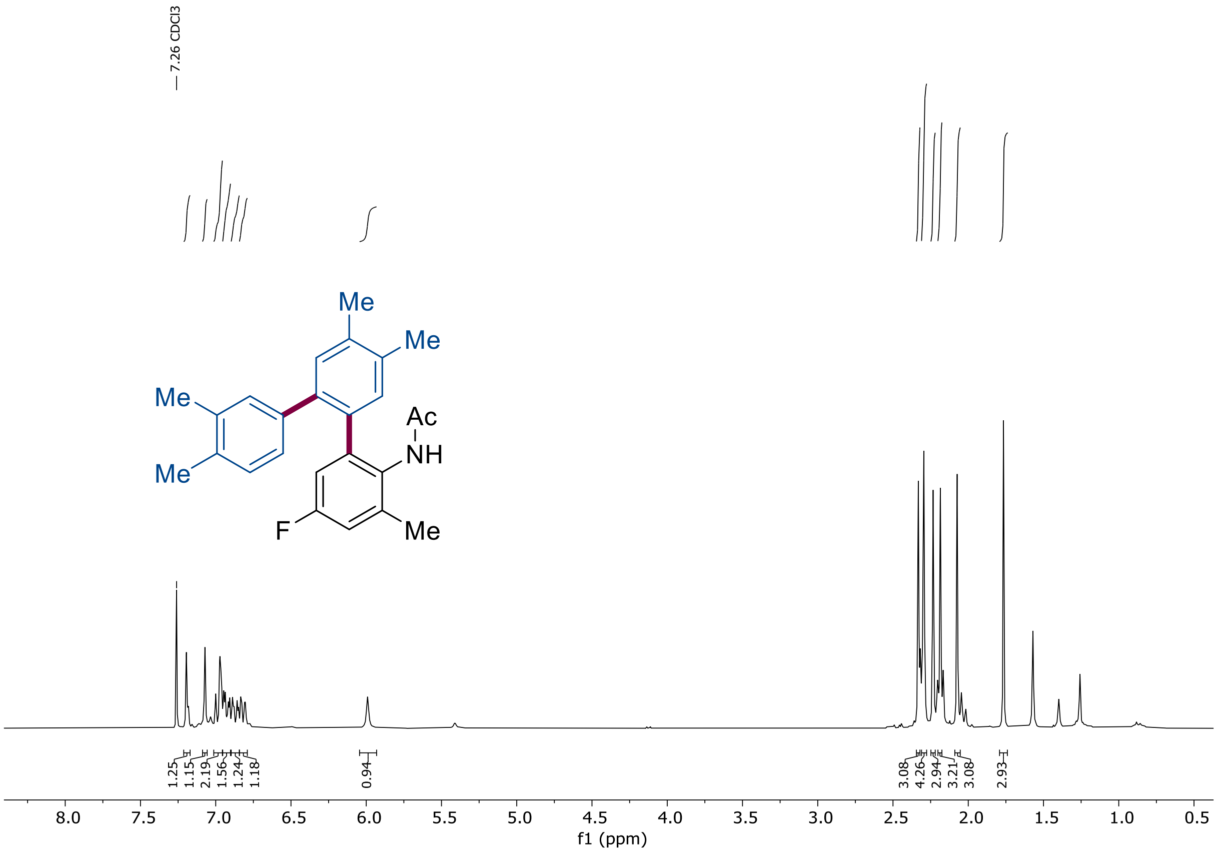

NMR Spectrum 26 ^{1}H NMR for di-11, 300 MHz, CDCl$_3$, room temperature.

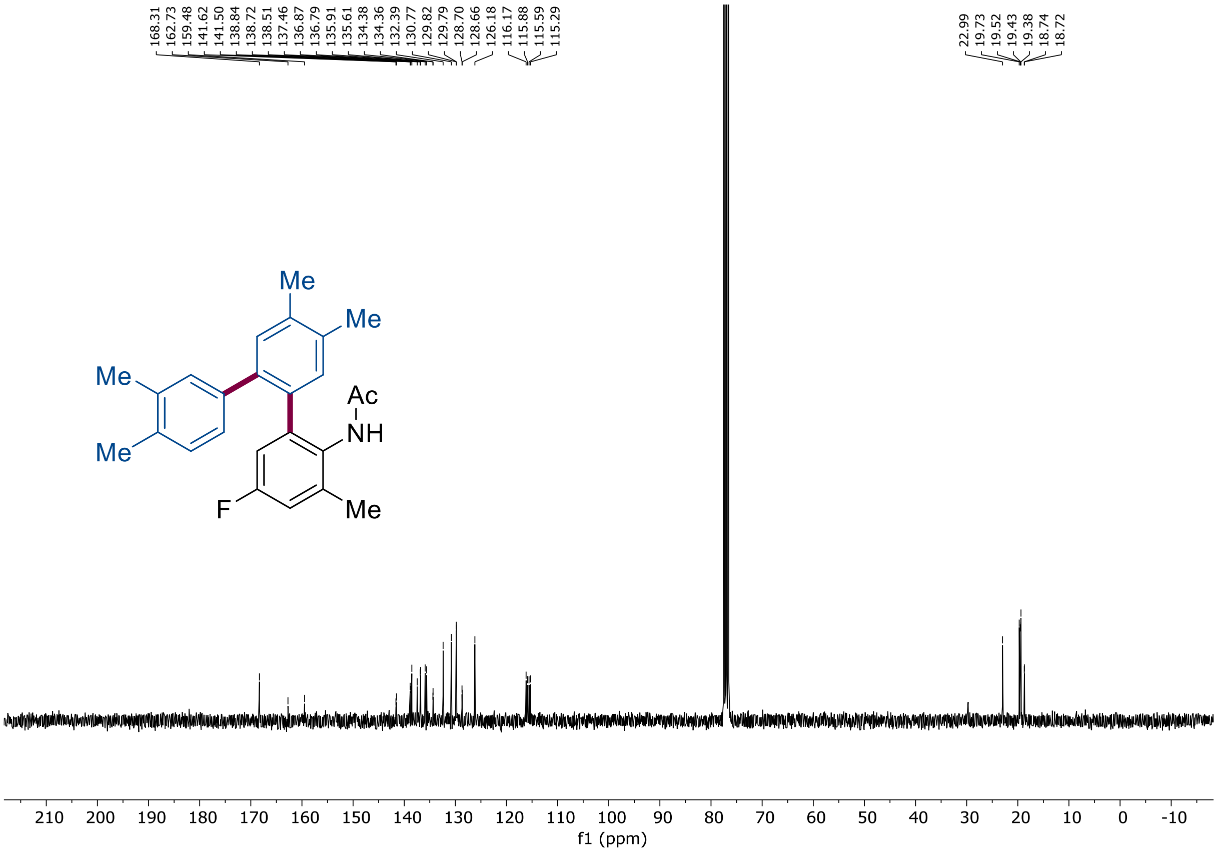

NMR Spectrum 27 ^{13}C **NMR for di-11, 75 MHz, $CDCl_3$, room temperature.**

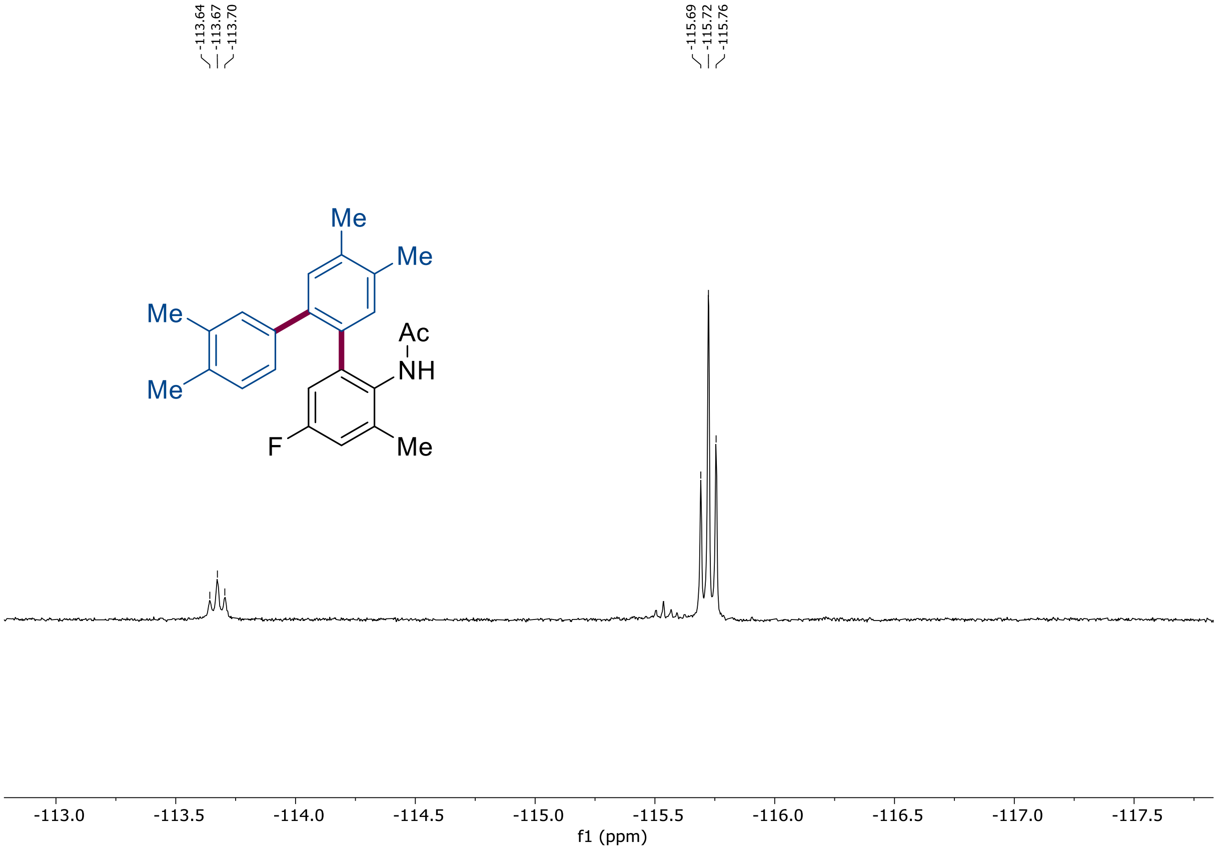

NMR Spectrum 28 ^{19}F **NMR for di-11, 282 MHz, $CDCl_3$, room temperature.**

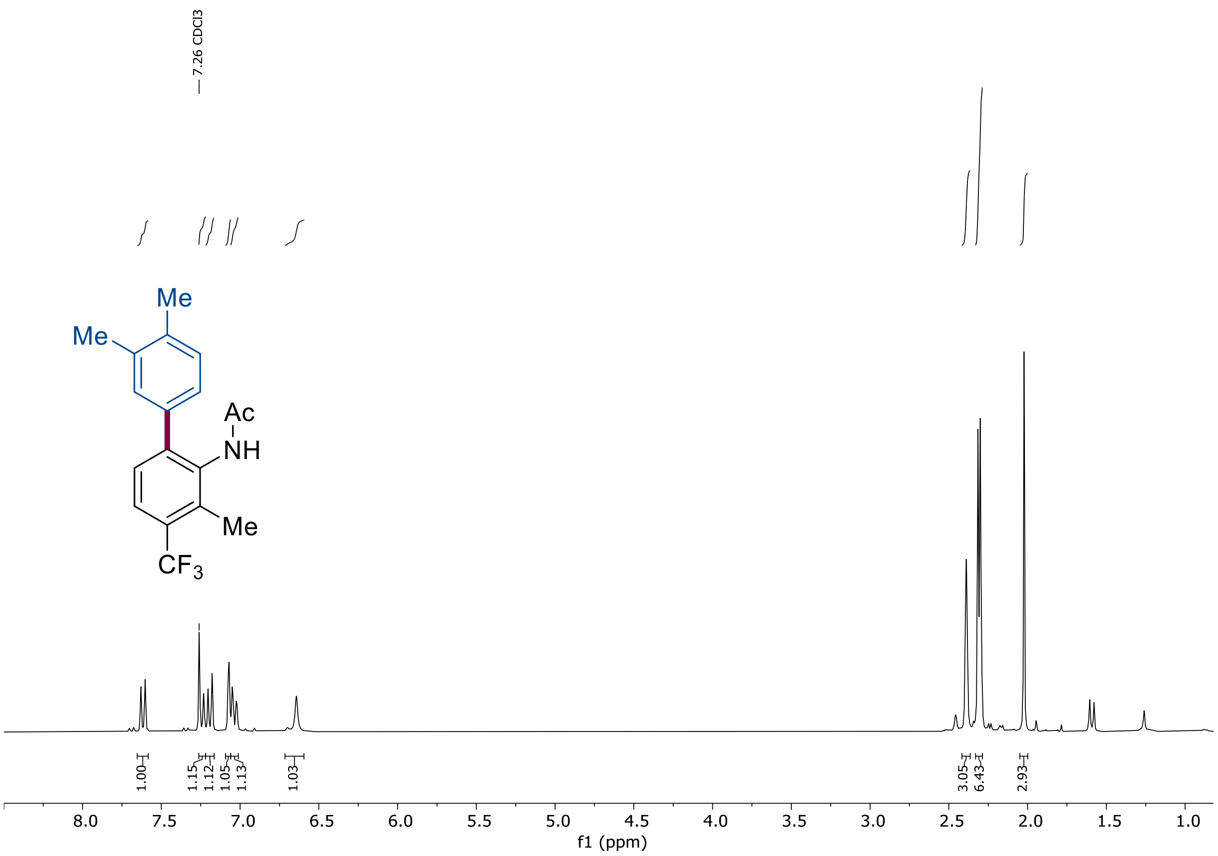

NMR Spectrum 29 ^{1}H NMR for 12, 300 MHz, CDCl3, room temperature.

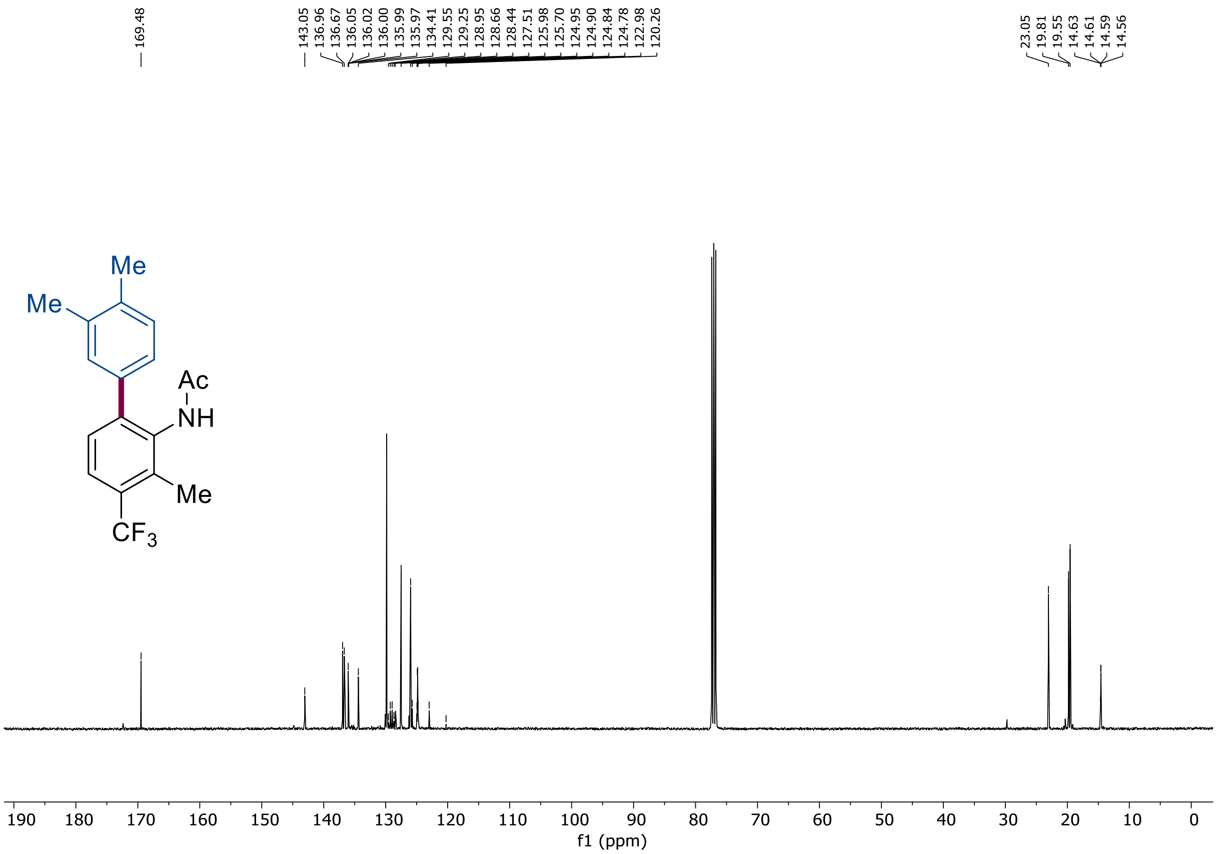

NMR Spectrum 30 ^{13}C NMR for 12, 101 MHz, CDCl3, room temperature.

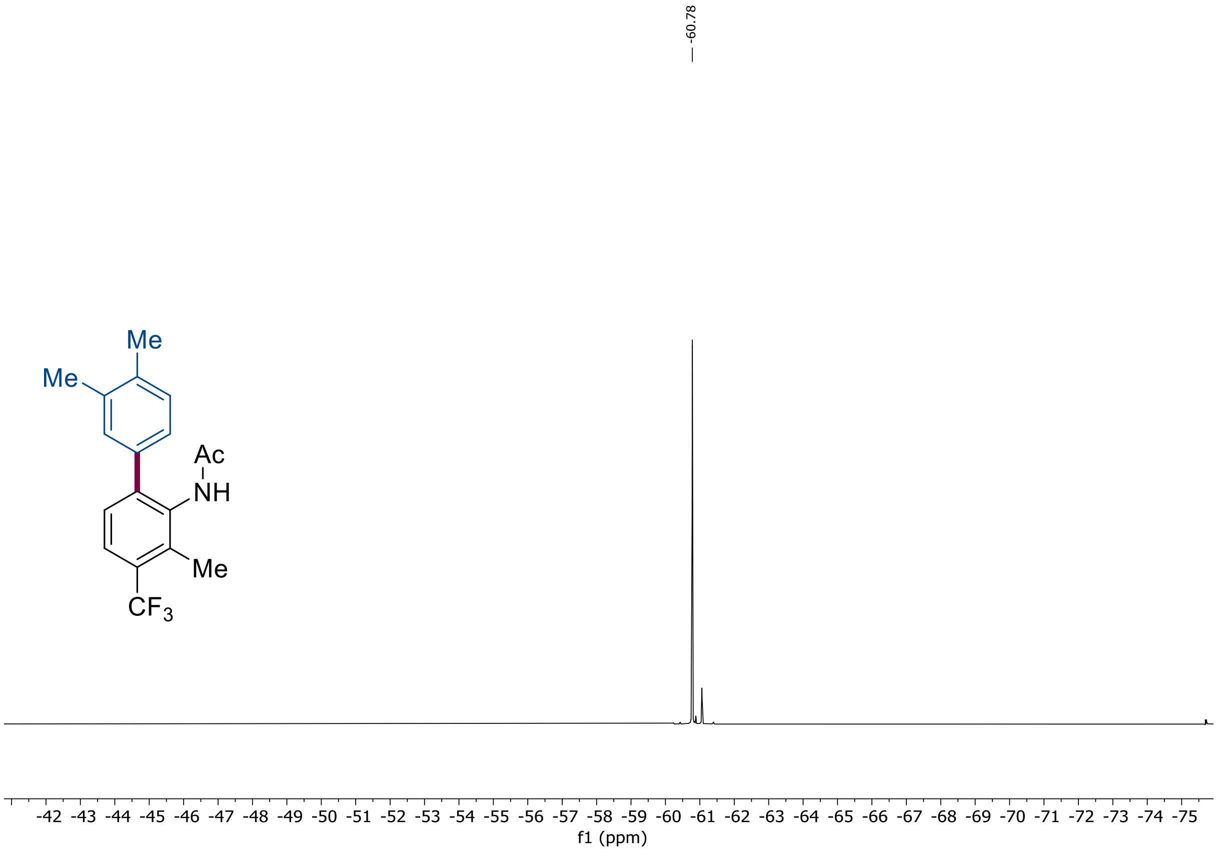

NMR Spectrum 31 ^{19}**F NMR for 12, 282 MHz, CDCl$_3$, room temperature.**

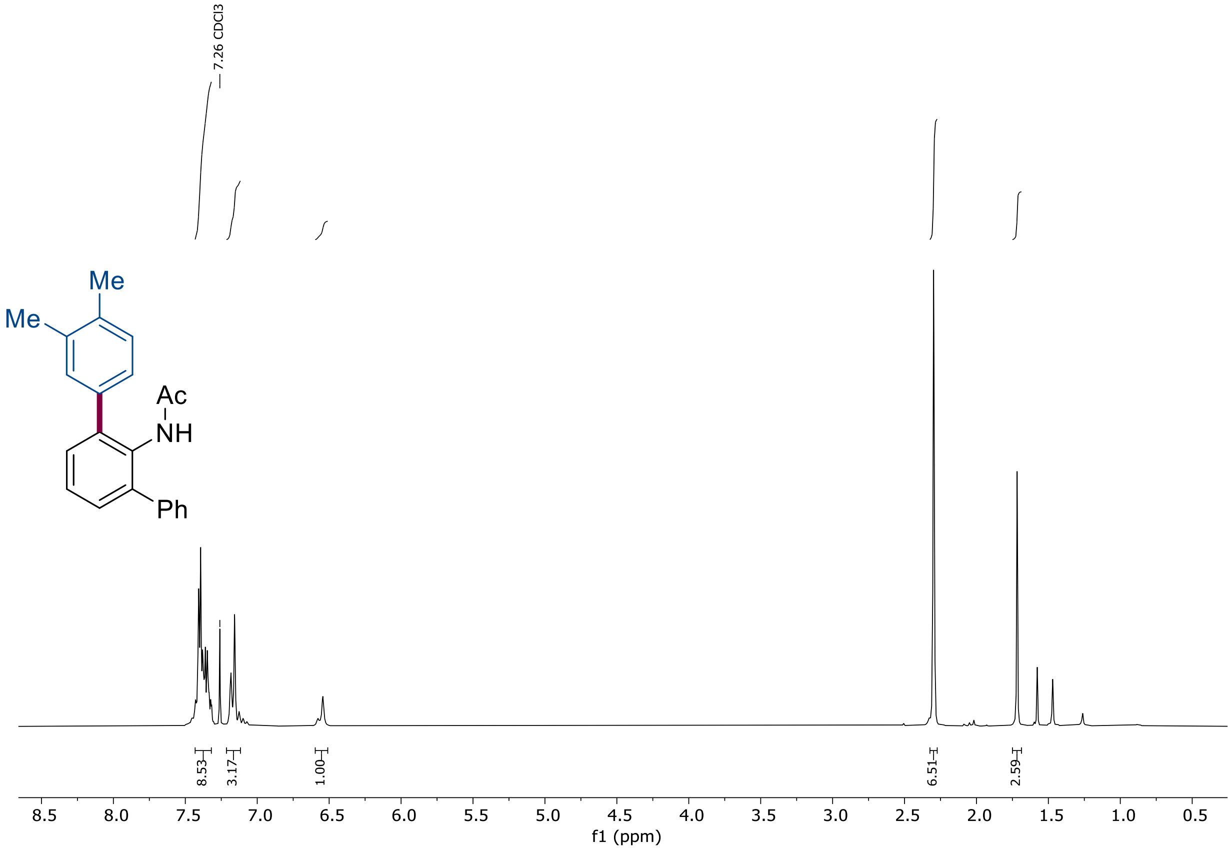

NMR Spectrum 32 ^{1}**H NMR for 13, 300 MHz, CDCl$_3$, room temperature.**

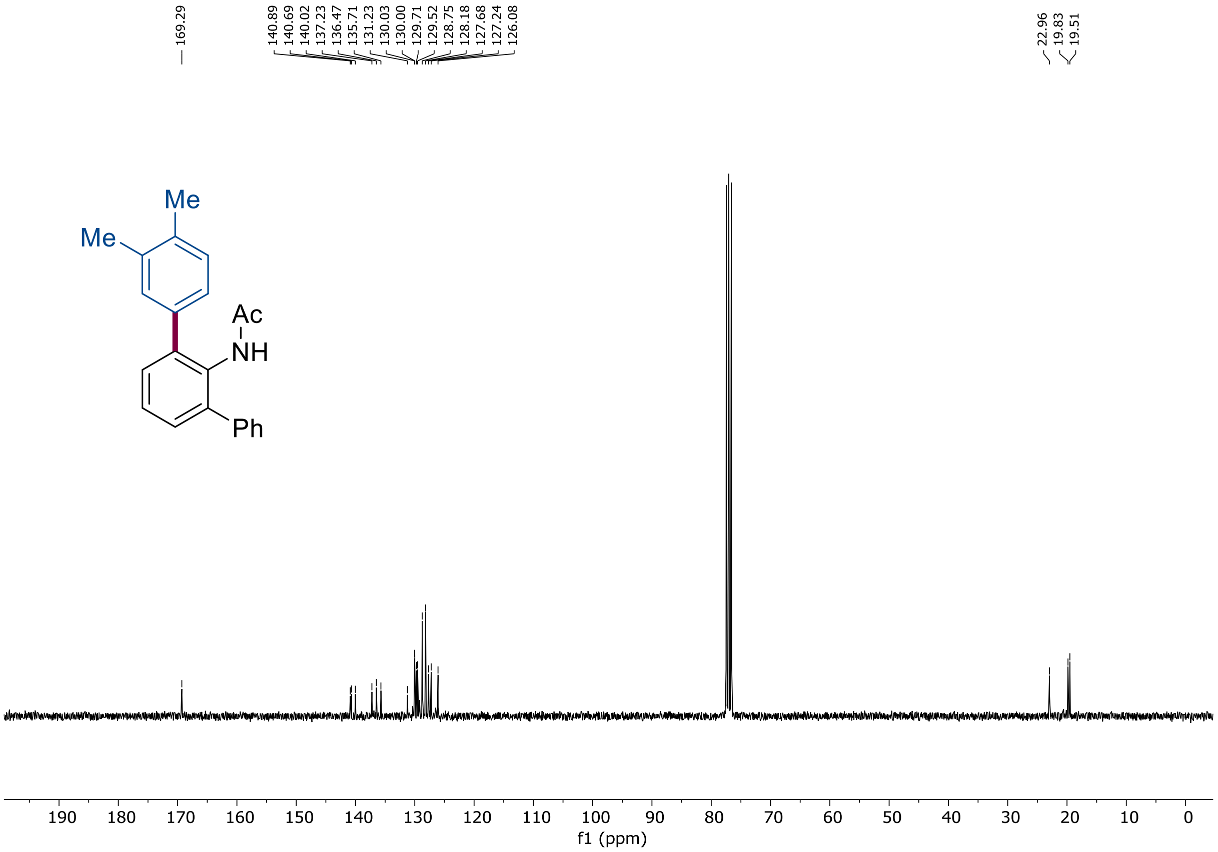

NMR Spectrum 33 ^{13}C NMR for 13, 75 MHz, $CDCl_3$, room temperature.

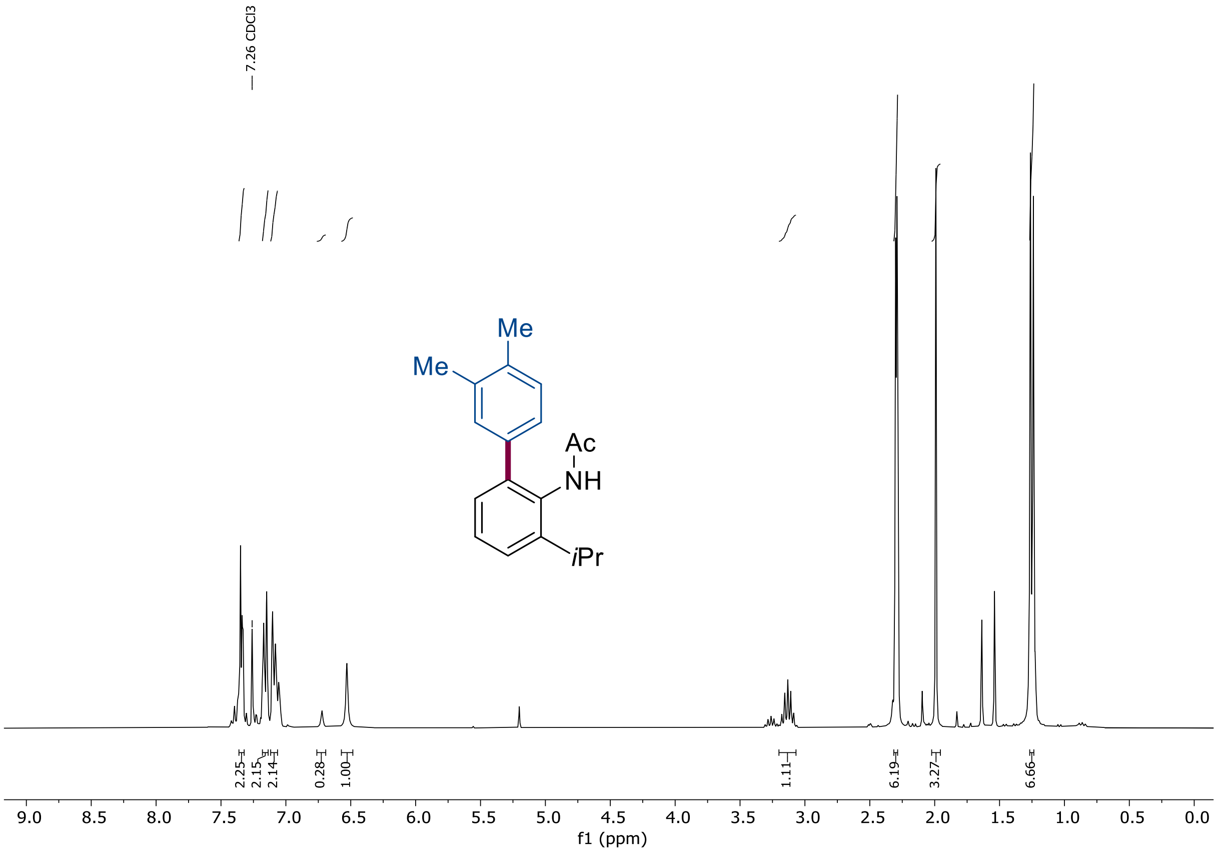

NMR Spectrum 34 ^{1}H NMR for 14, 300 MHz, $CDCl_3$, room temperature.

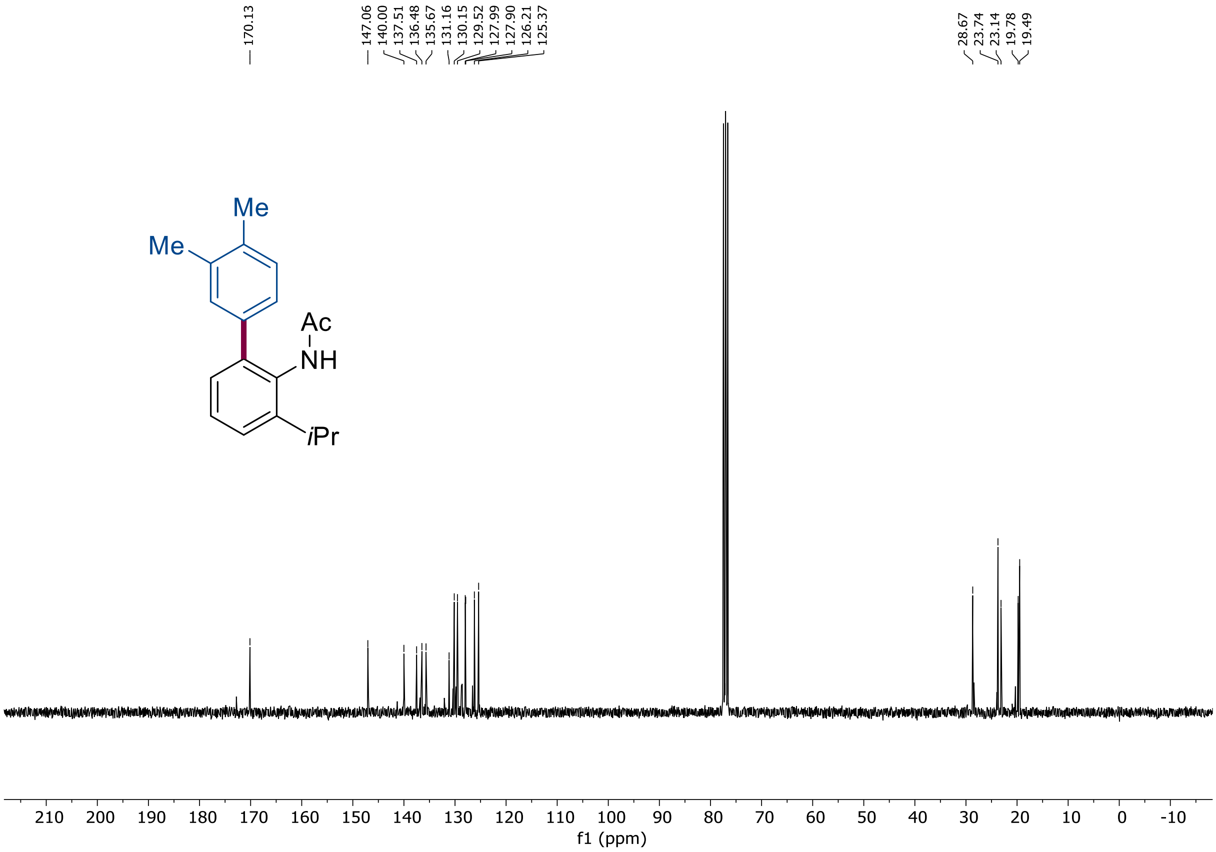

NMR Spectrum 35 ^{13}C NMR for 14, 75 MHz, CDCl$_3$, room temperature.

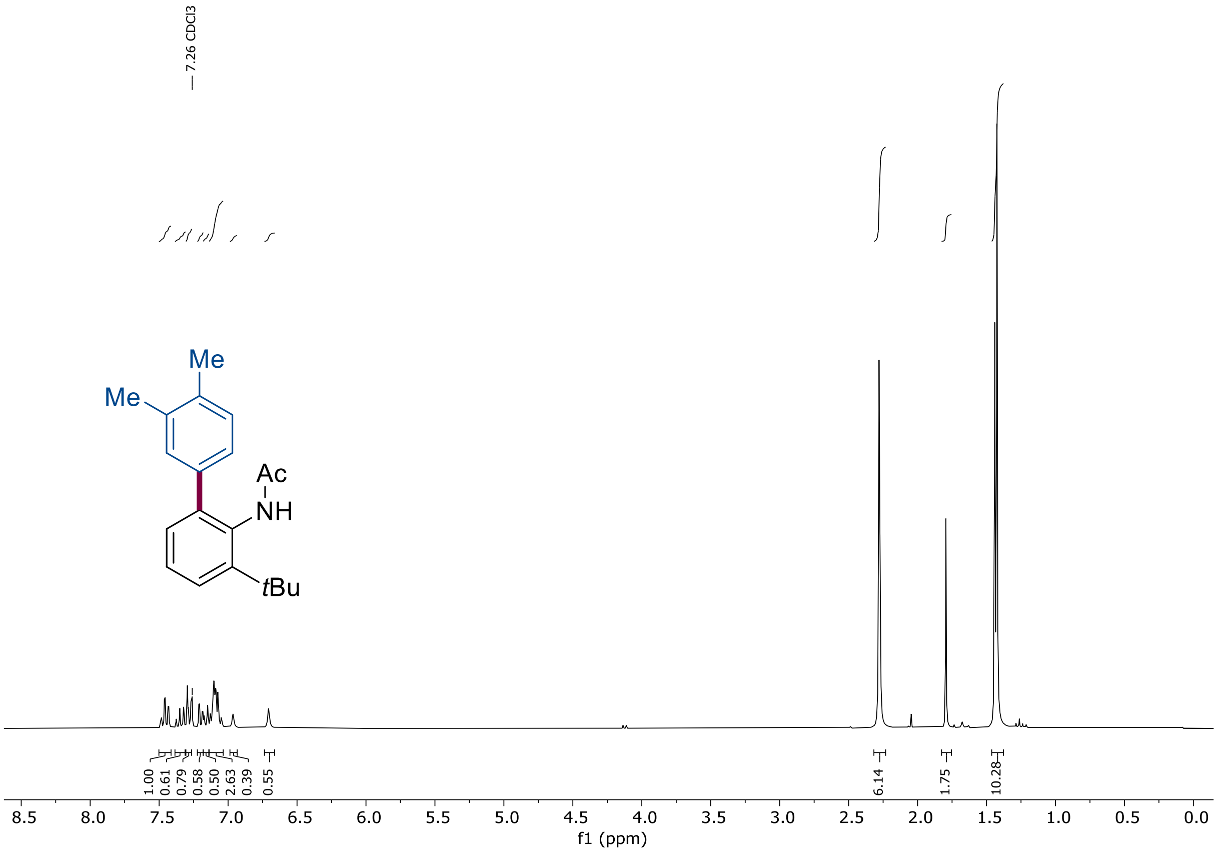

NMR Spectrum 36 ^{1}H NMR for 15, 300 MHz, CDCl$_3$, room temperature.

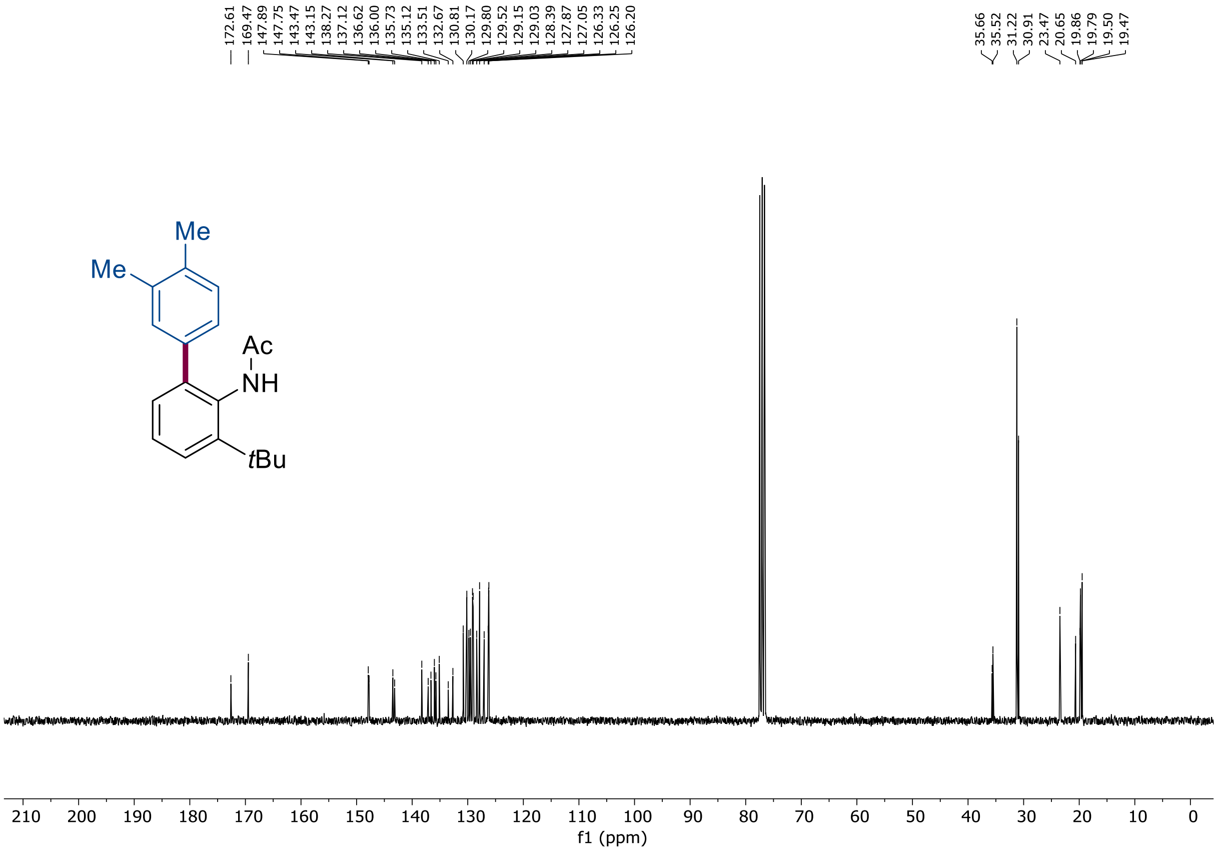

NMR Spectrum 37 ^{13}C NMR for 15, 75 MHz, $CDCl_3$, room temperature.

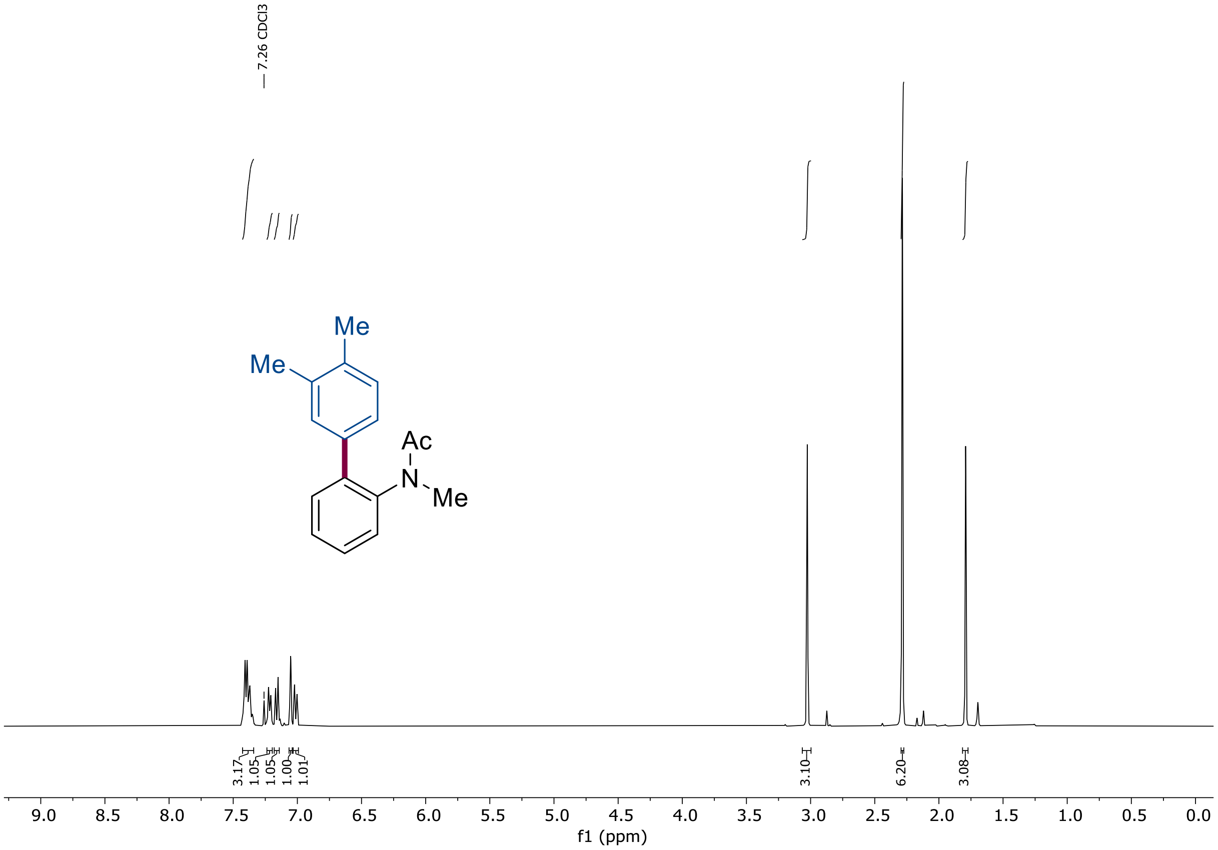

NMR Spectrum 38 ^{1}H NMR for 16, 400 MHz, $CDCl_3$, room temperature.

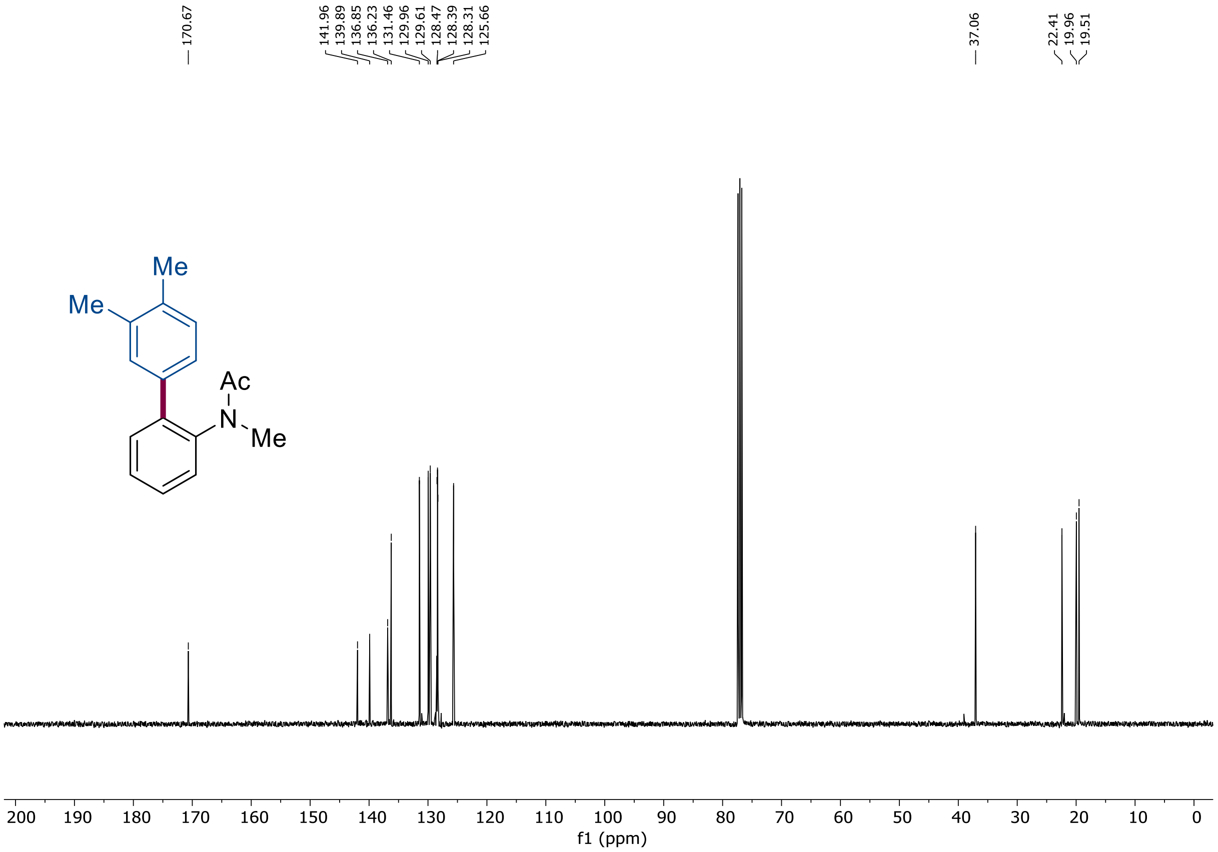

NMR Spectrum 39 ^{13}C NMR for 16, 101 MHz, CDCl3, room temperature.

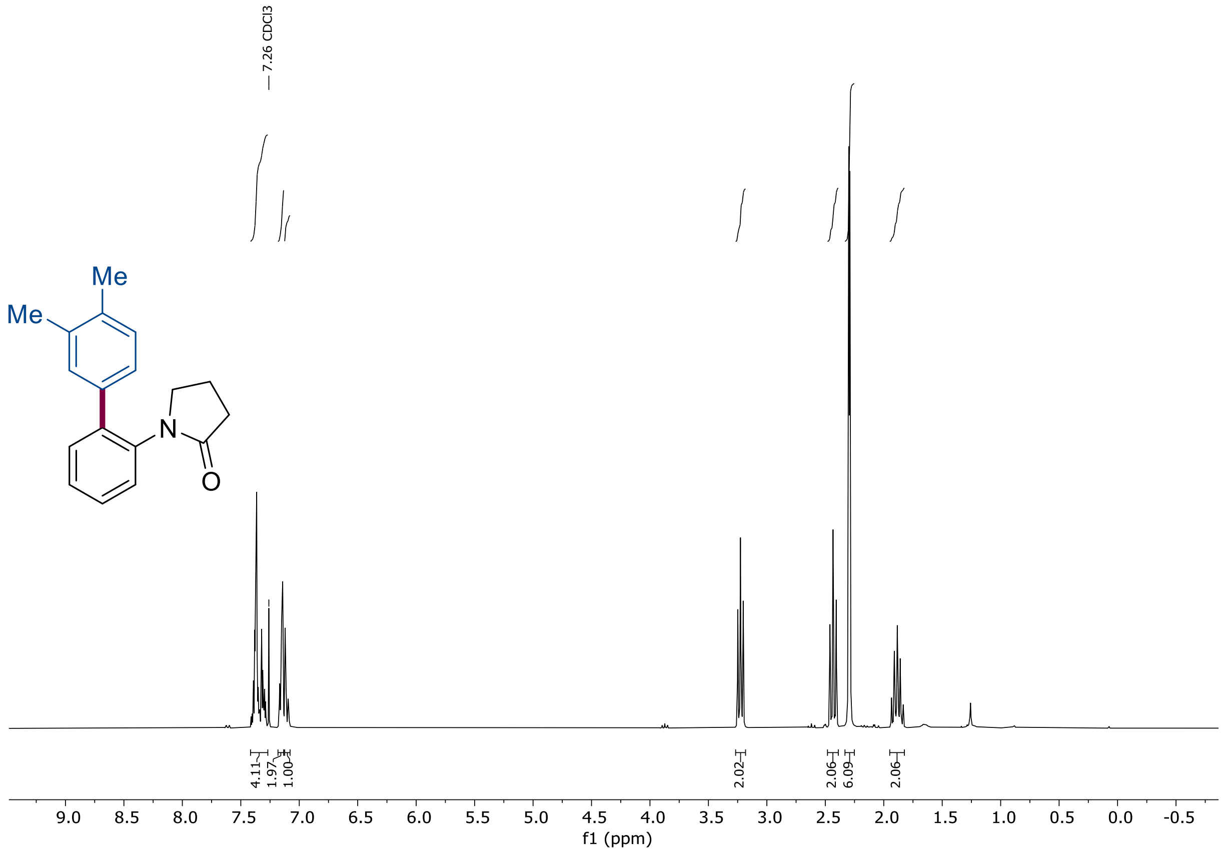

NMR Spectrum 40 ^{1}H NMR for 17, 300 MHz, CDCl3, room temperature.

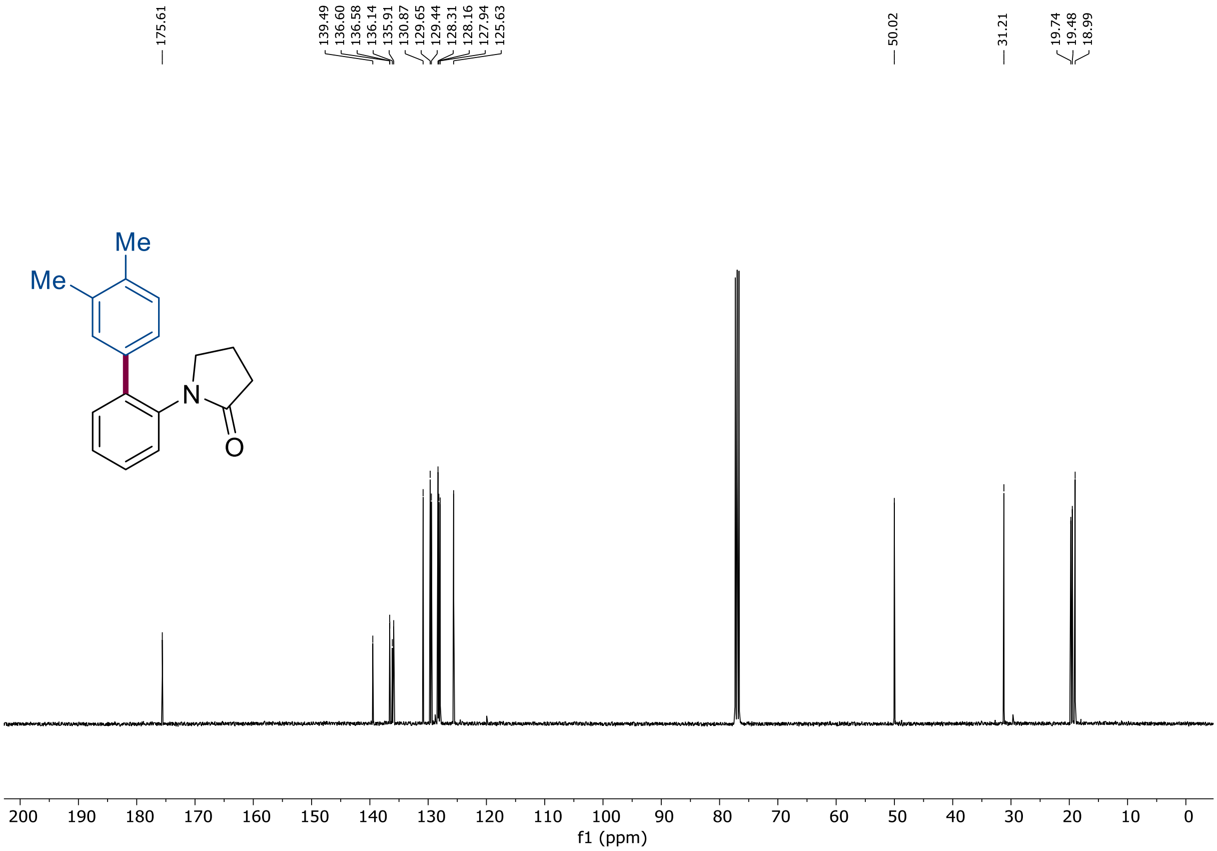

NMR Spectrum 41 ^{13}C **NMR for 17, 101 MHz, CDCl3, room temperature.**

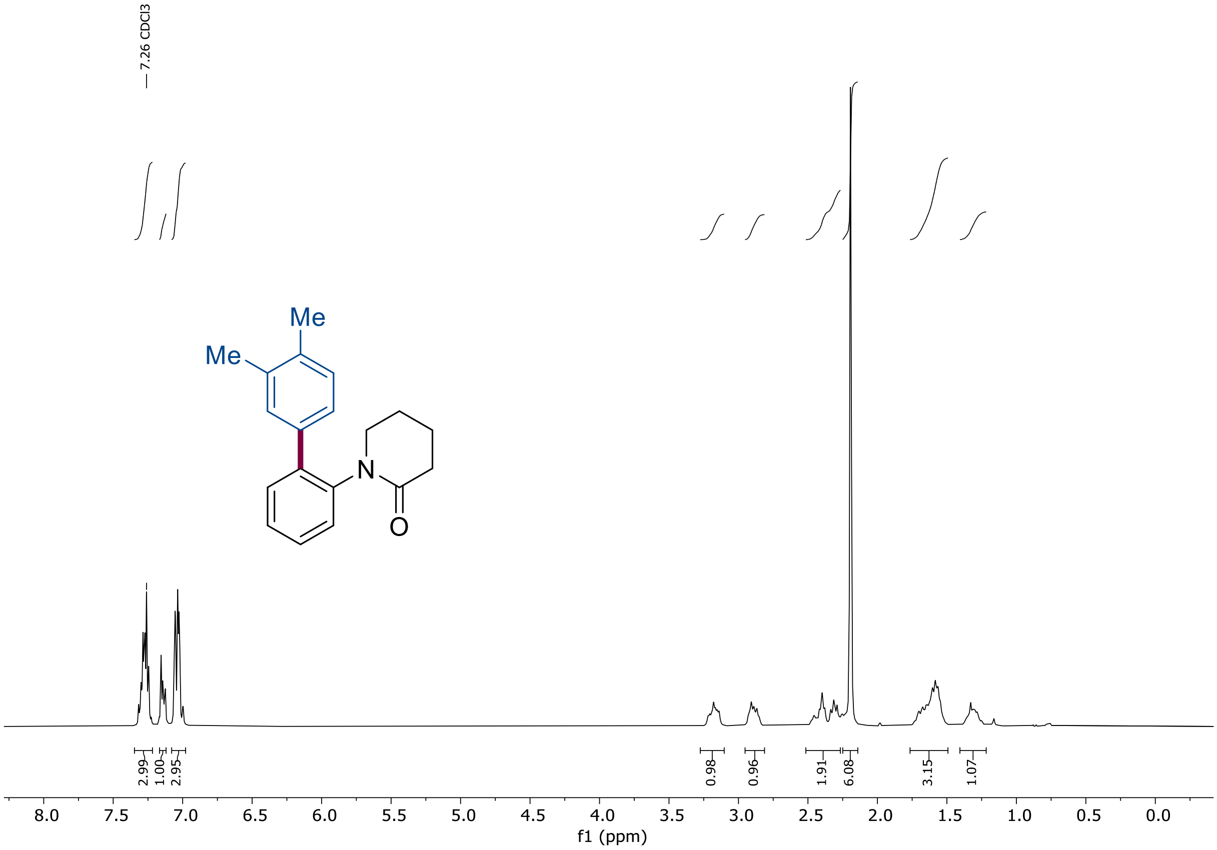

NMR Spectrum 42 ^{1}H **NMR for 18, 300 MHz, CDCl3, room temperature.**

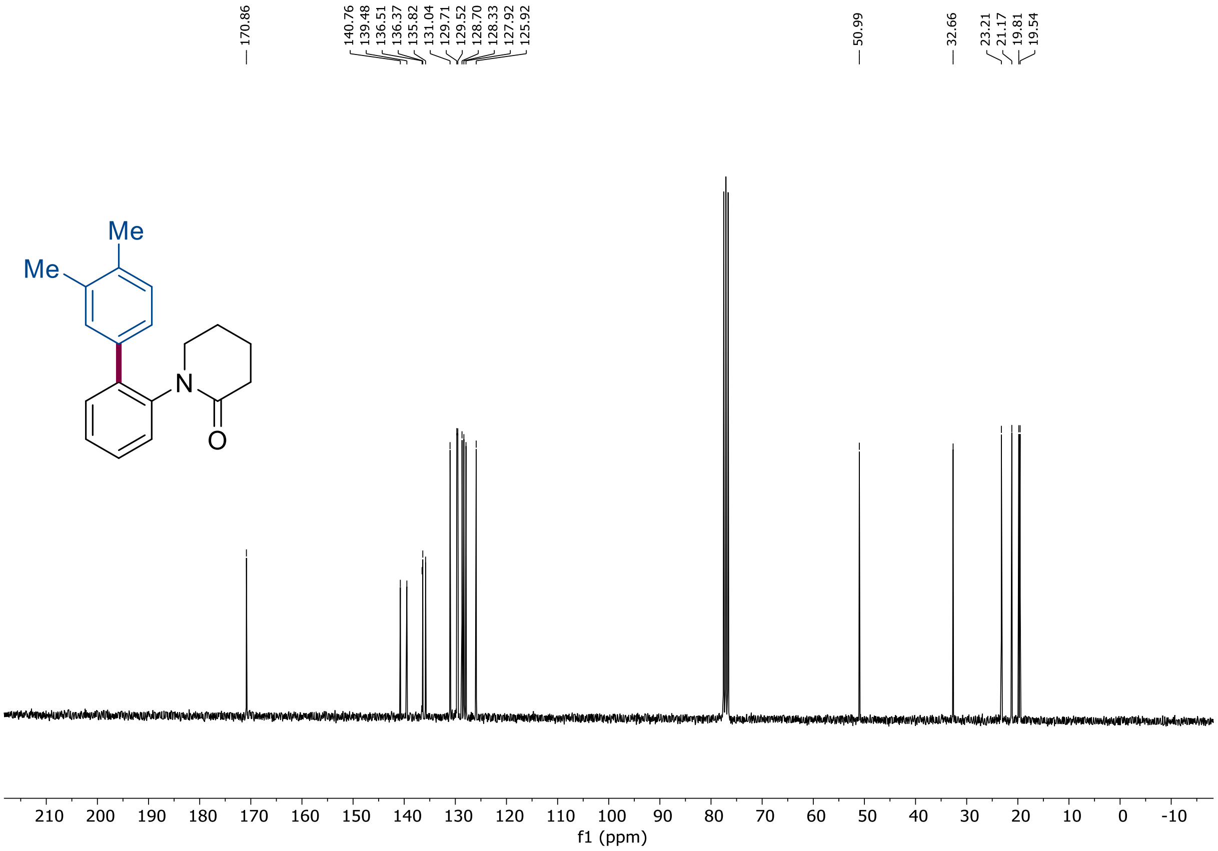

NMR Spectrum 43 ^{13}C NMR for 18, 75 MHz, $CDCl_3$, room temperature.

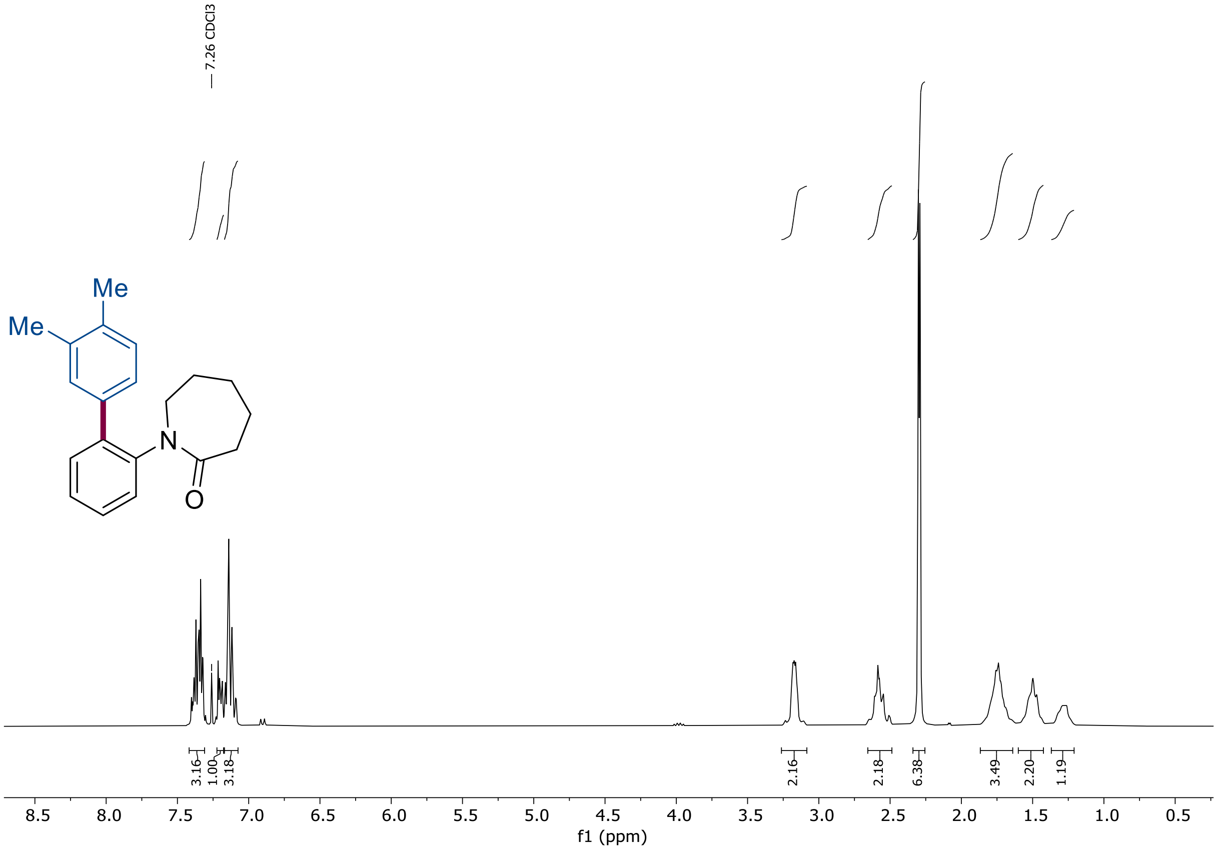

NMR Spectrum 44 ^{1}H NMR for 19, 300 MHz, $CDCl_3$, room temperature.

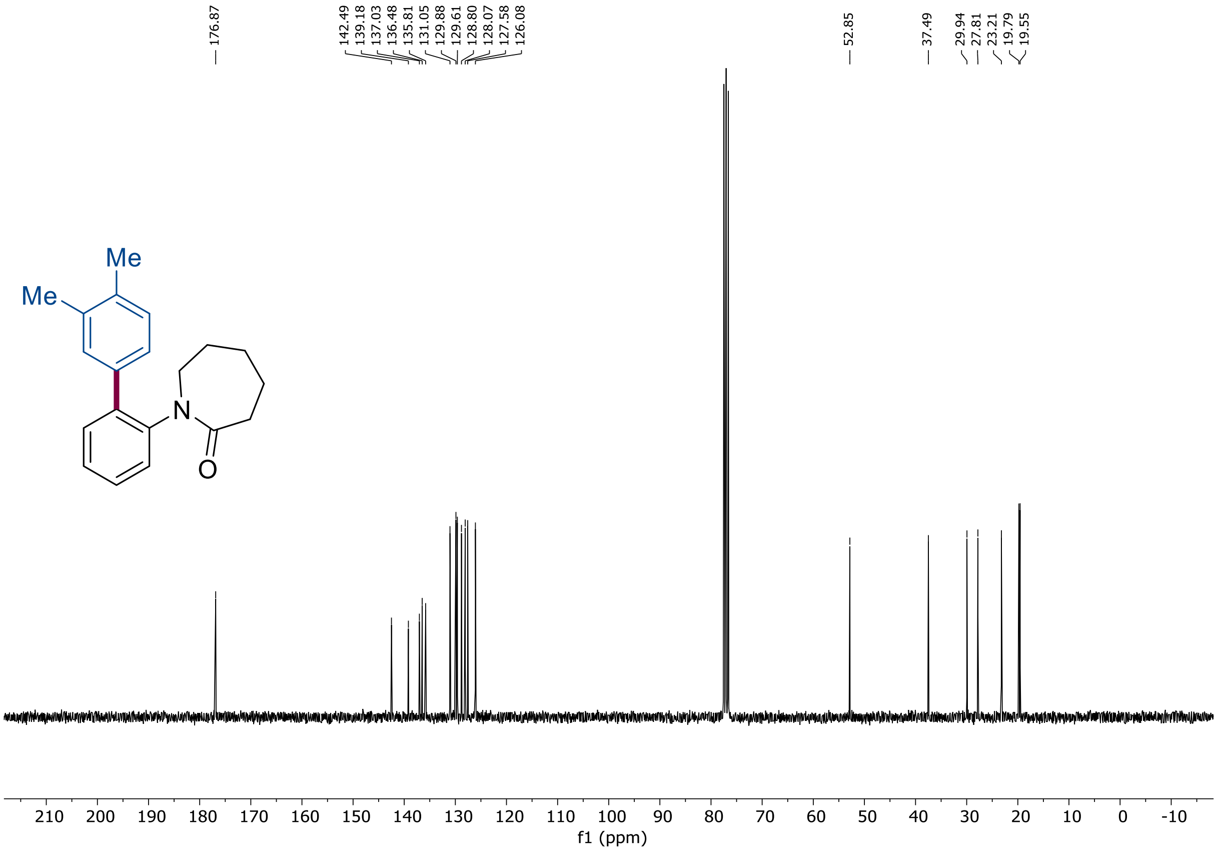

NMR Spectrum 45 ^{13}C NMR for 19, 75 MHz, $CDCl_3$, room temperature.

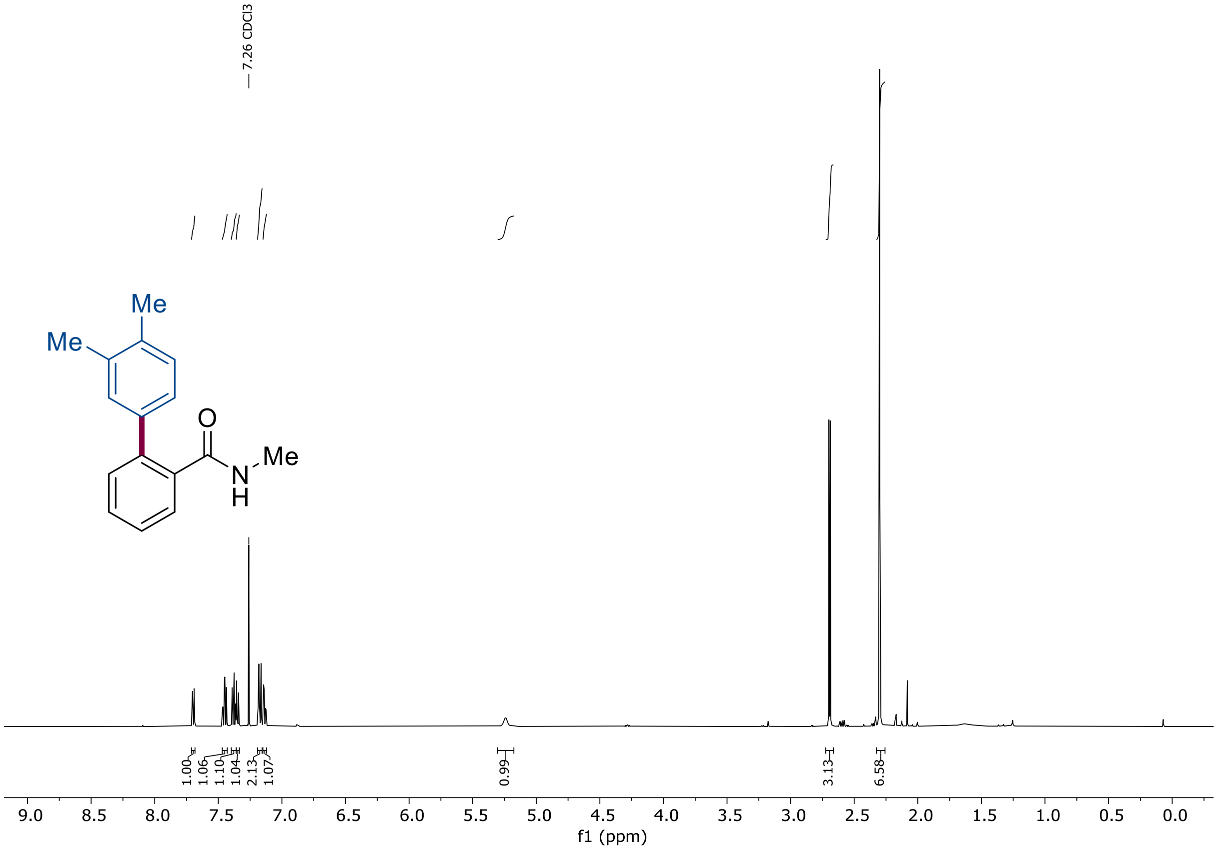

NMR Spectrum 46 ^{1}H NMR for 20, 500 MHz, $CDCl_3$, room temperature.

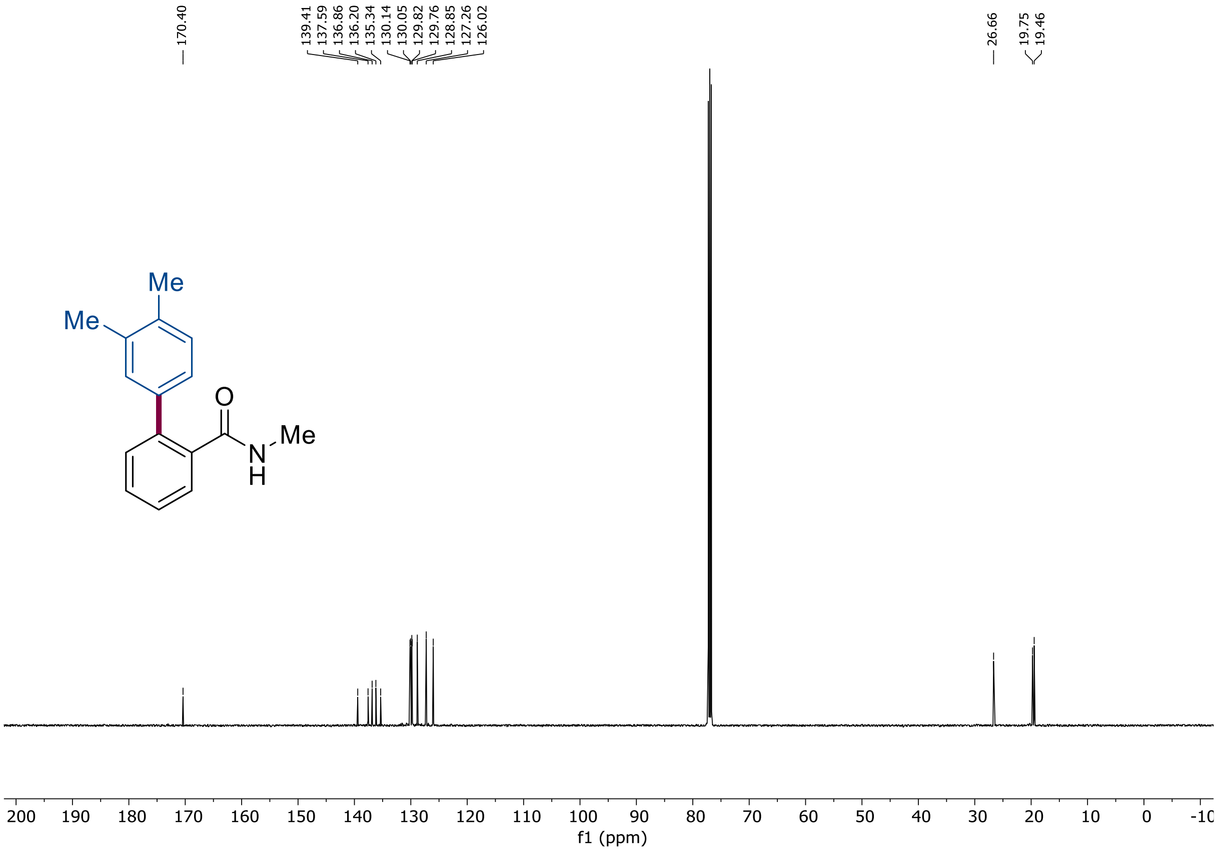

NMR Spectrum 47 ^{13}C NMR for 20, 126 MHz, $CDCl_3$, room temperature.

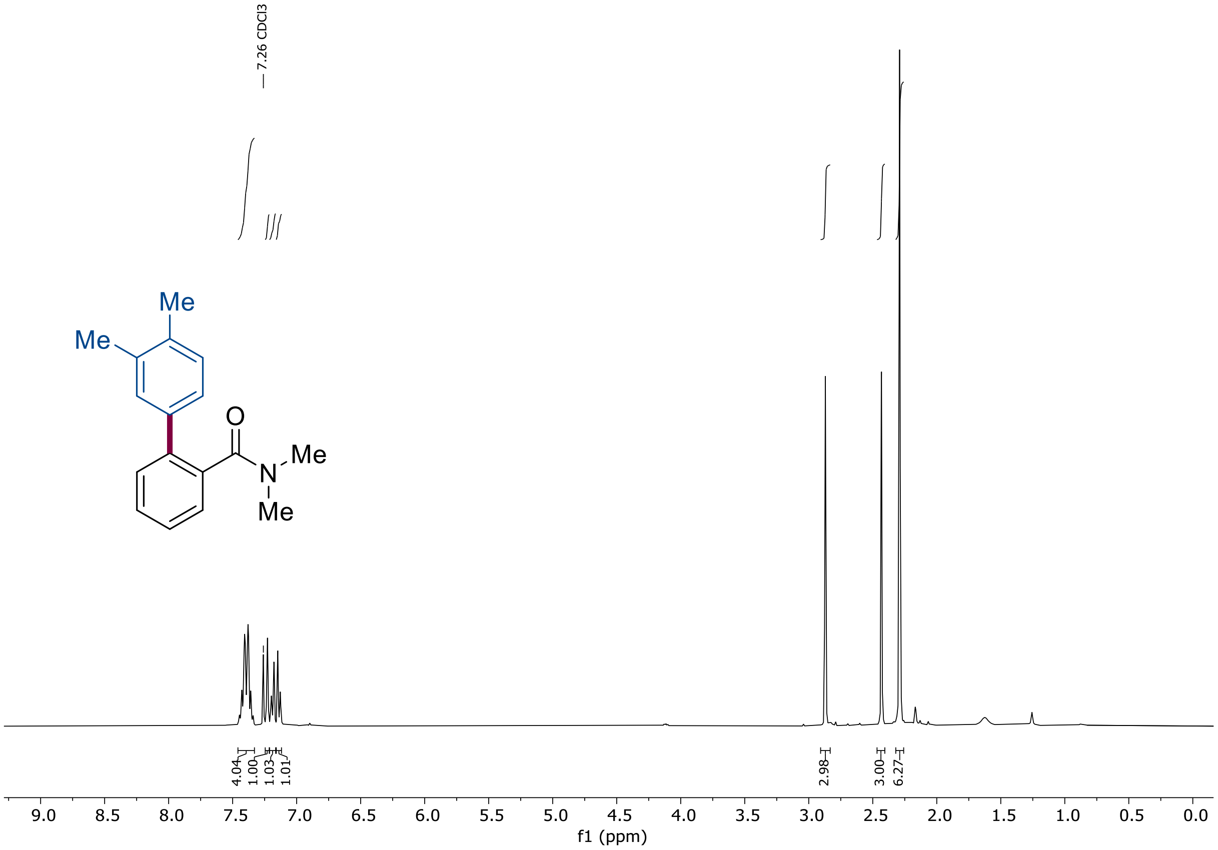

NMR Spectrum 48 ^{1}H NMR for 21, 400 MHz, $CDCl_3$, room temperature.

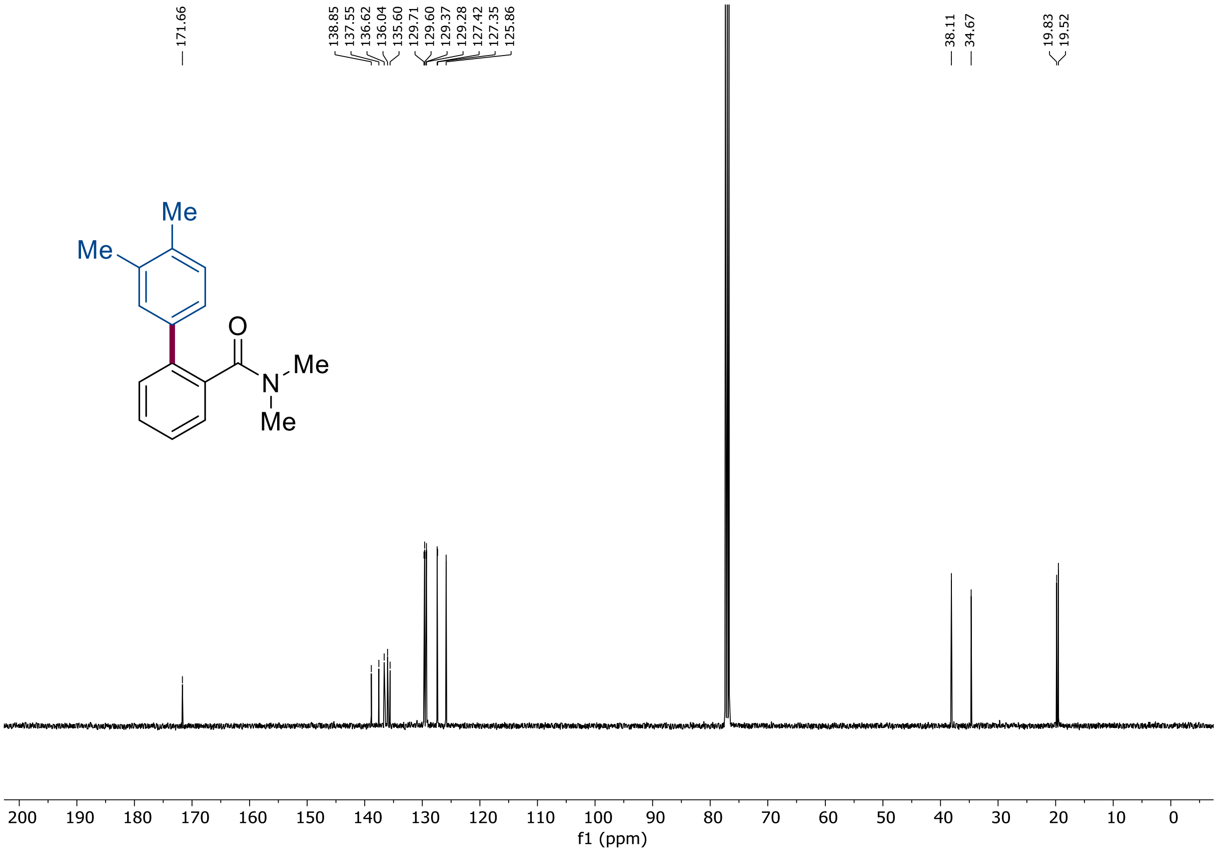

NMR Spectrum 49 ^{13}C NMR for 21, 101 MHz, $CDCl_3$, room temperature.

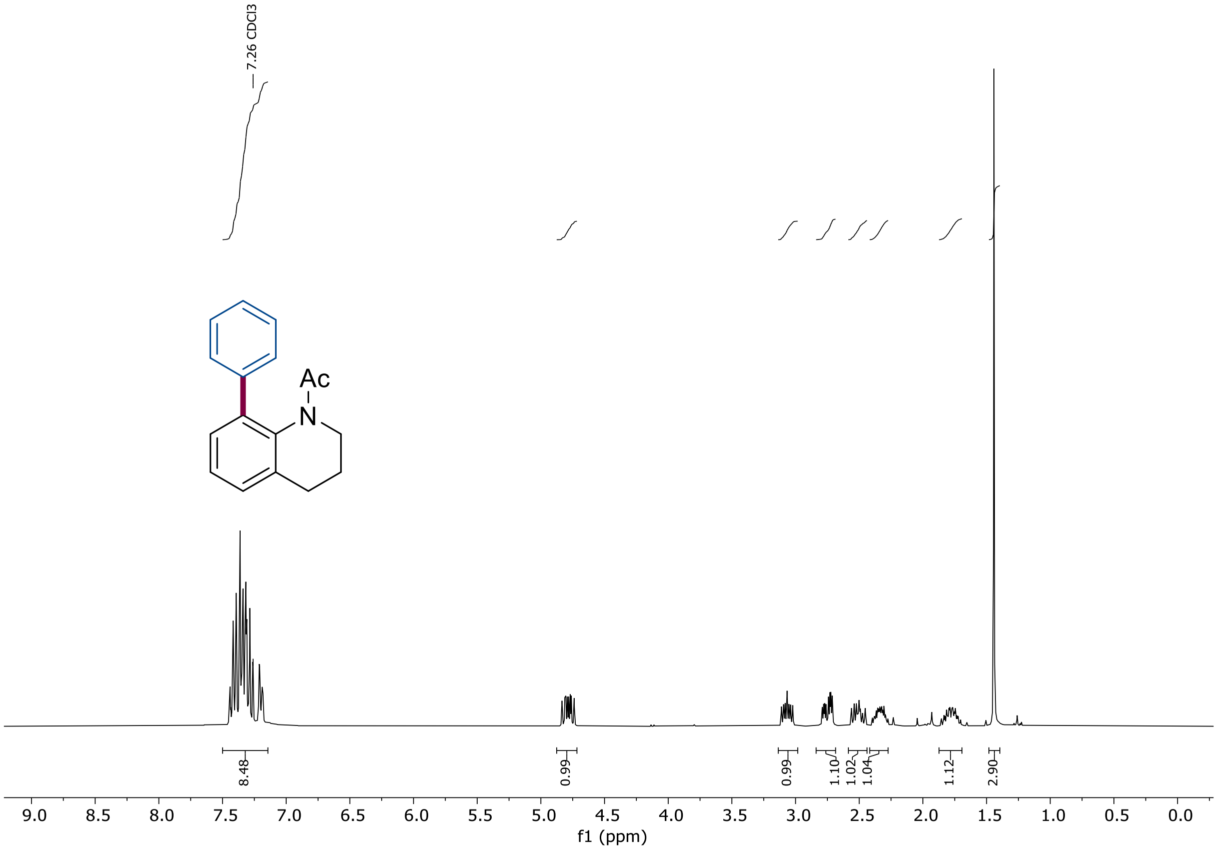

NMR Spectrum 50 ^{1}H NMR for 22, 300 MHz, $CDCl_3$, room temperature.

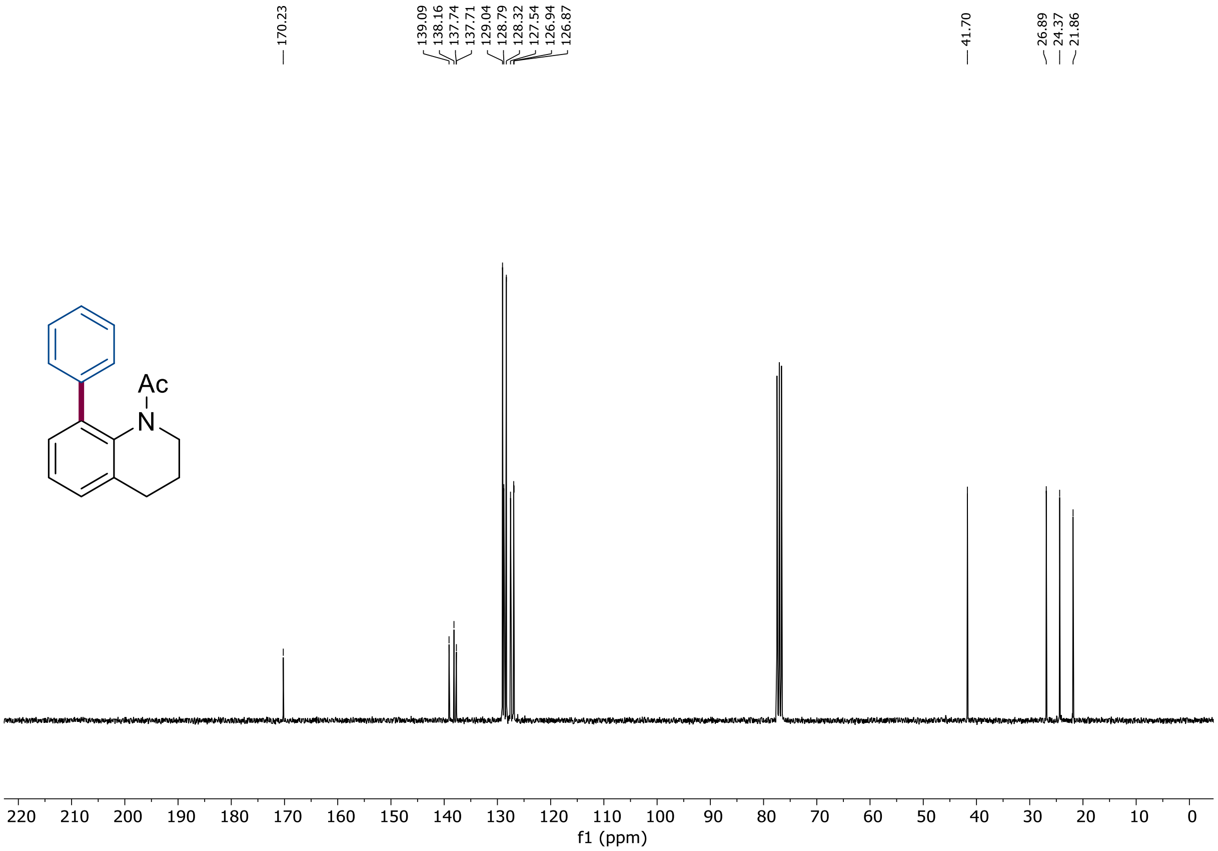

NMR Spectrum 51 ^{13}C NMR for 22, 75 MHz, $CDCl_3$, room temperature.

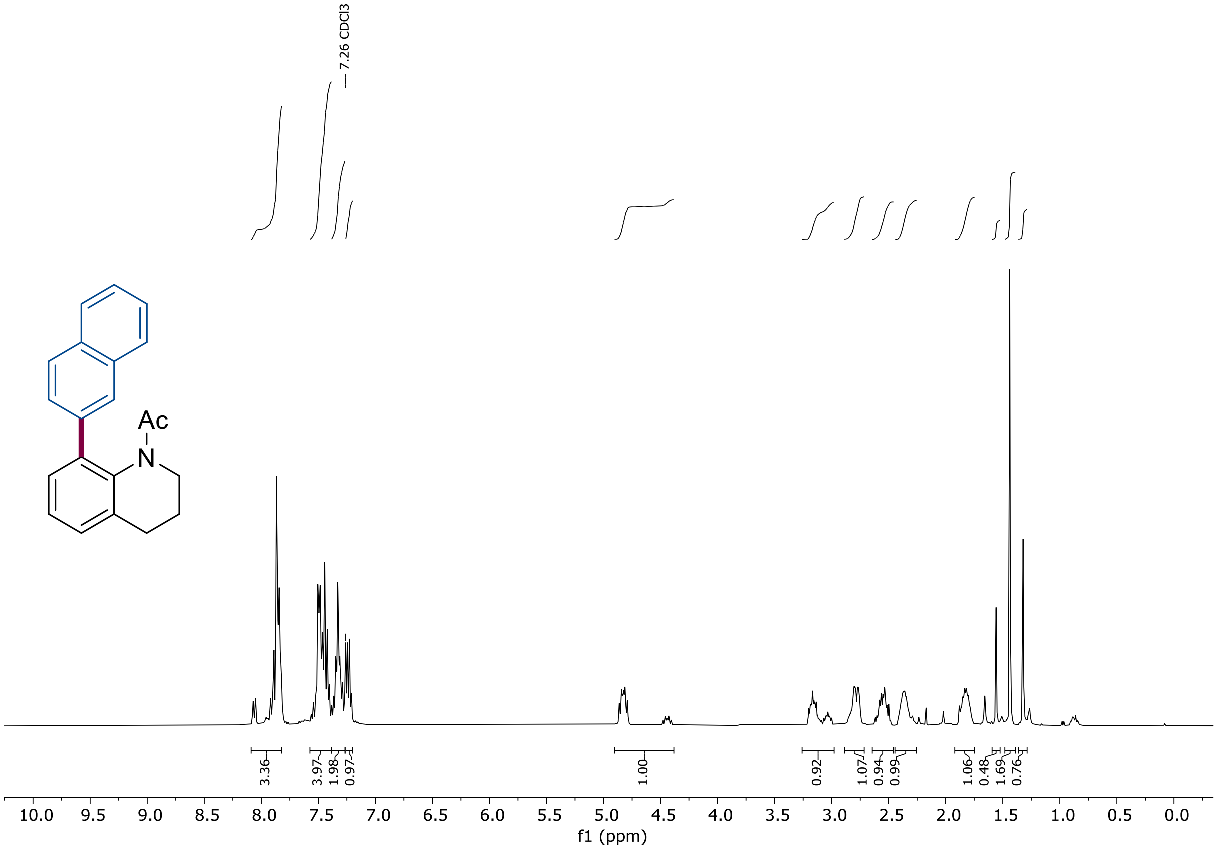

NMR Spectrum 52 ^{1}H NMR for 23, 400 MHz, $CDCl_3$, room temperature.

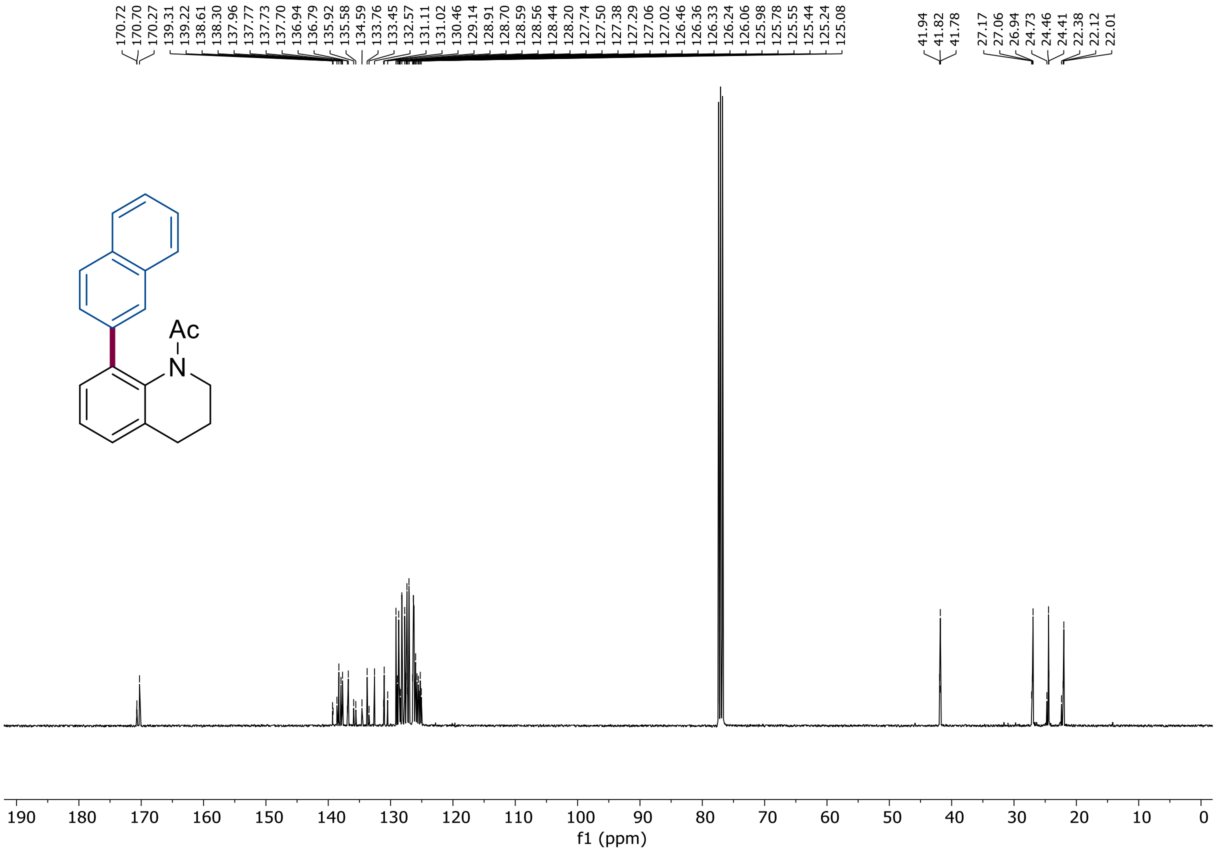

NMR Spectrum 53 ^{13}C NMR for 23, 101 MHz, $CDCl_3$, room temperature.

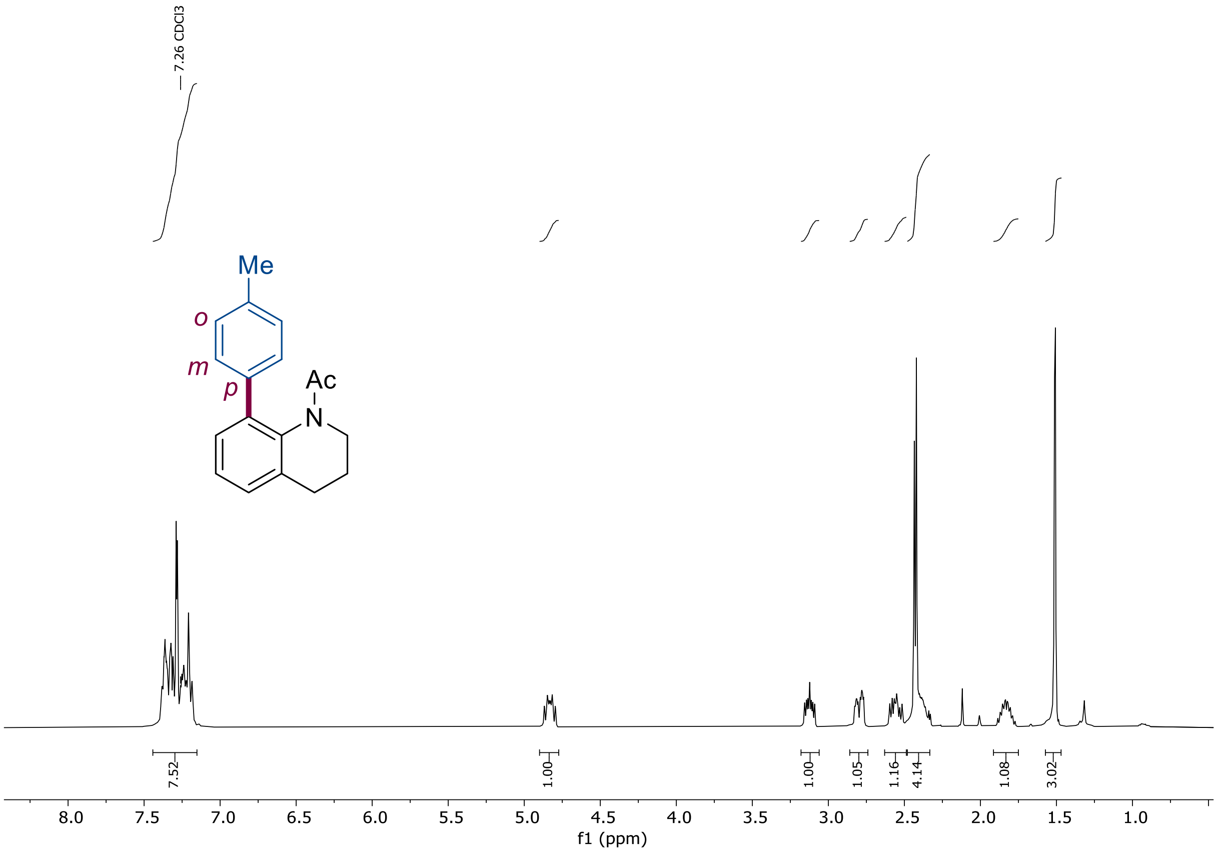

NMR Spectrum 54 ^{1}H NMR for 24, 400 MHz, $CDCl_3$, room temperature.

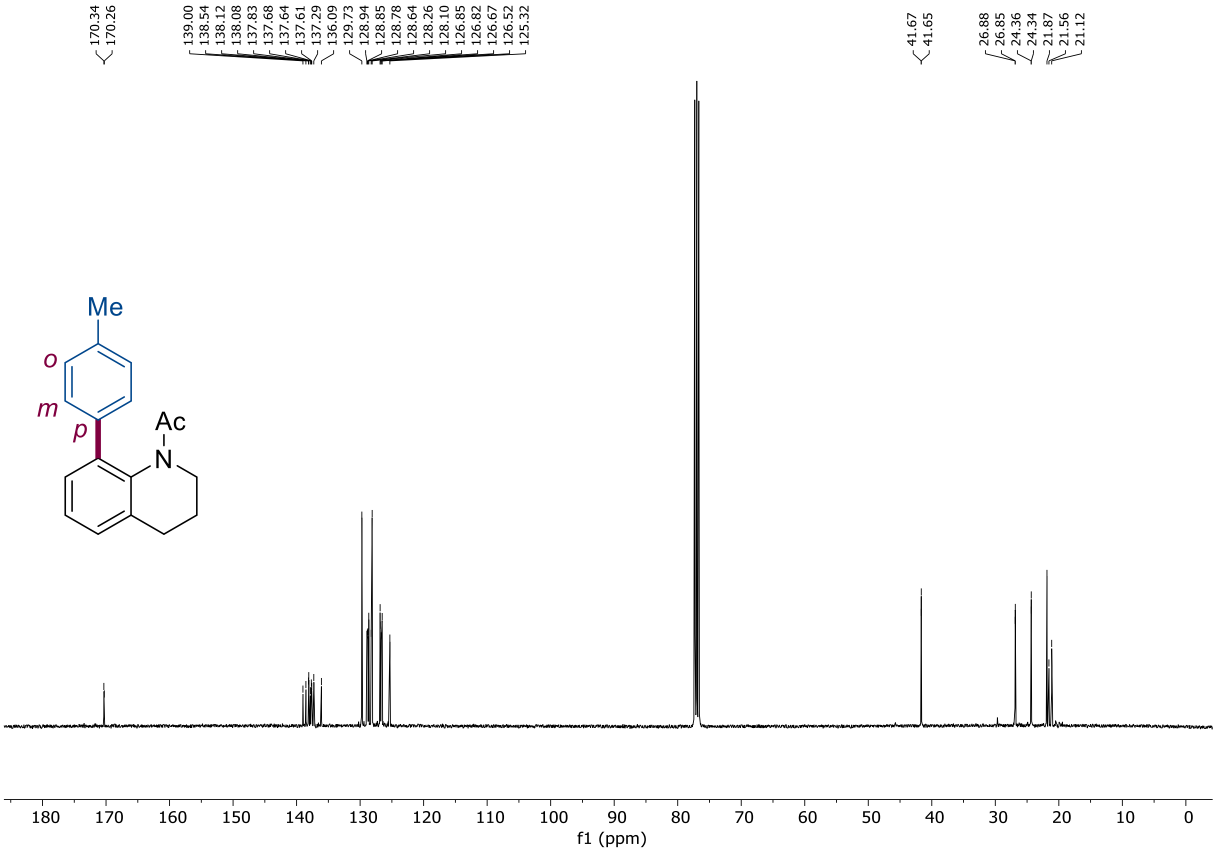

NMR Spectrum 55 **^{13}C NMR for 24, 101 MHz, CDCl$_3$, room temperature.**

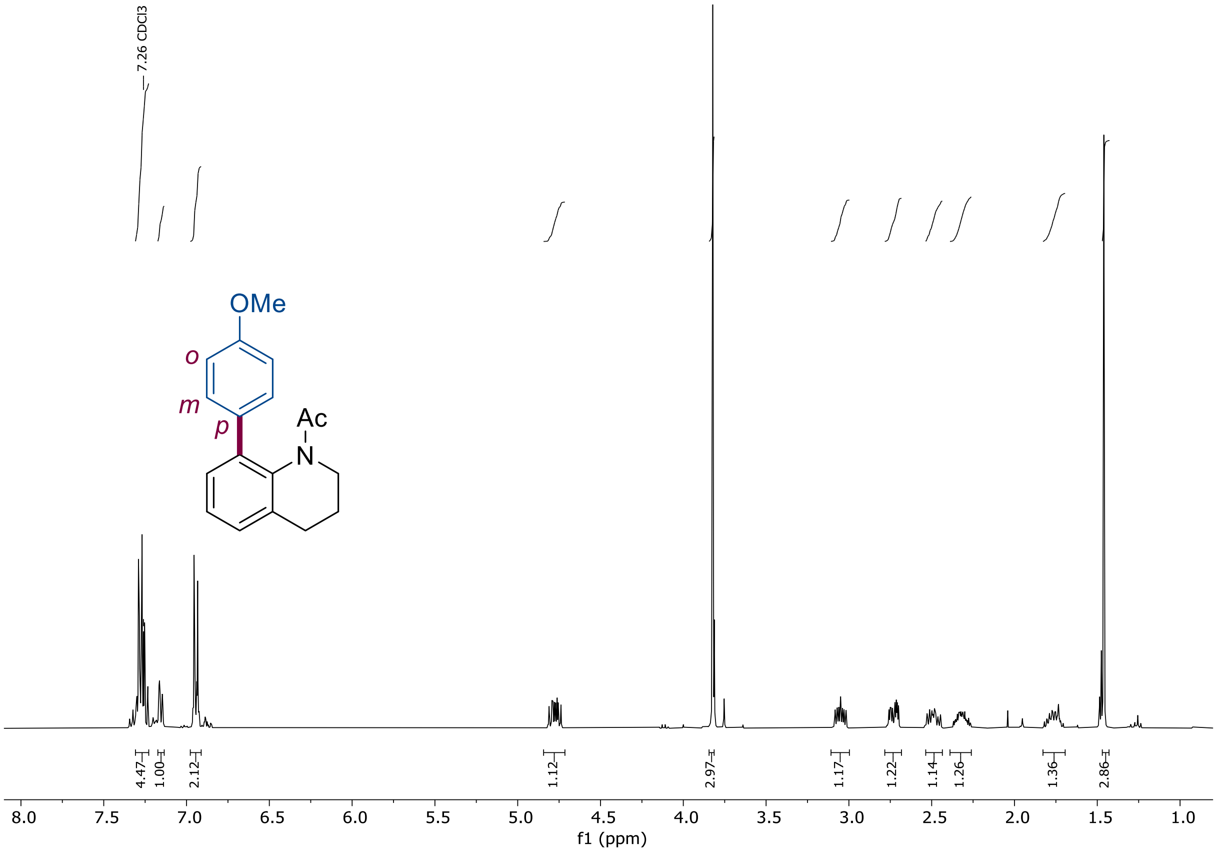

NMR Spectrum 56 **^{1}H NMR for 25, 400 MHz, CDCl$_3$, room temperature.**

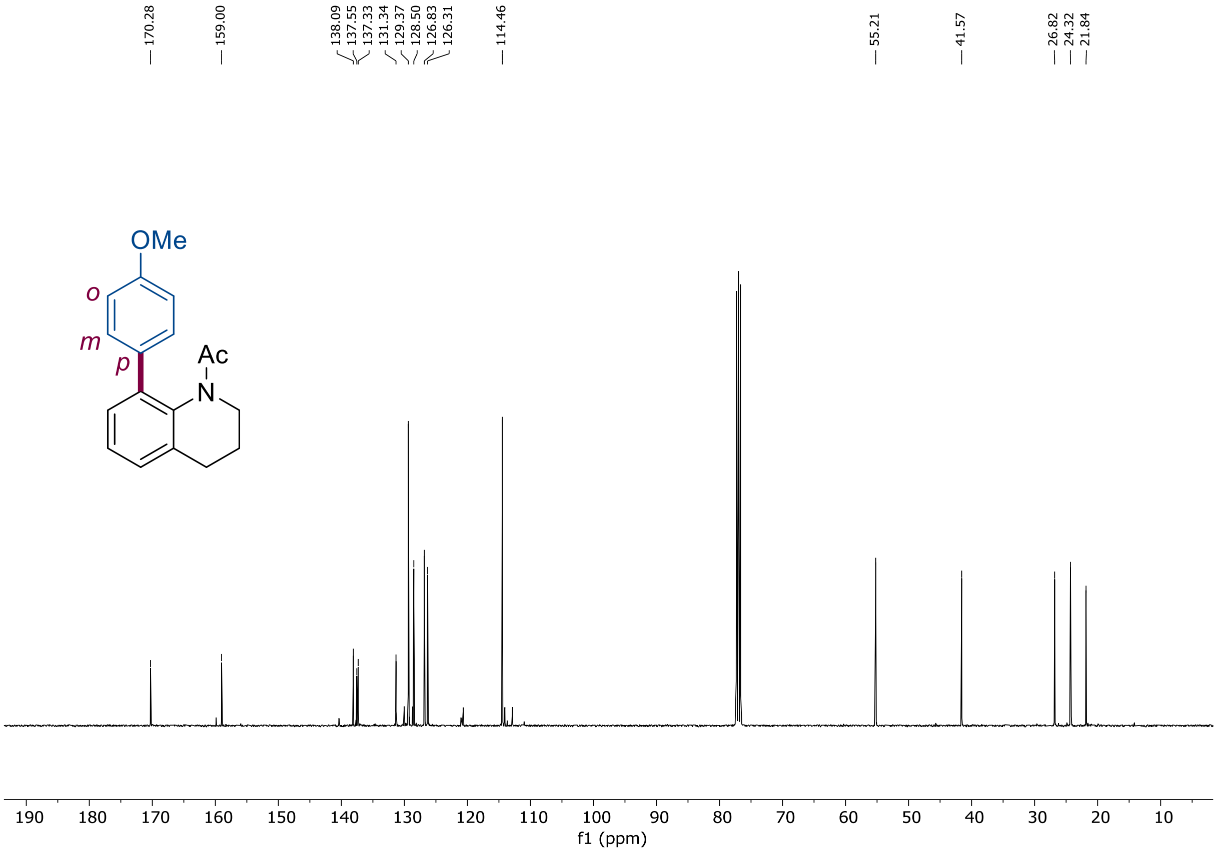

NMR Spectrum 57 ^{13}C NMR for 25, 101 MHz, $CDCl_3$, room temperature.

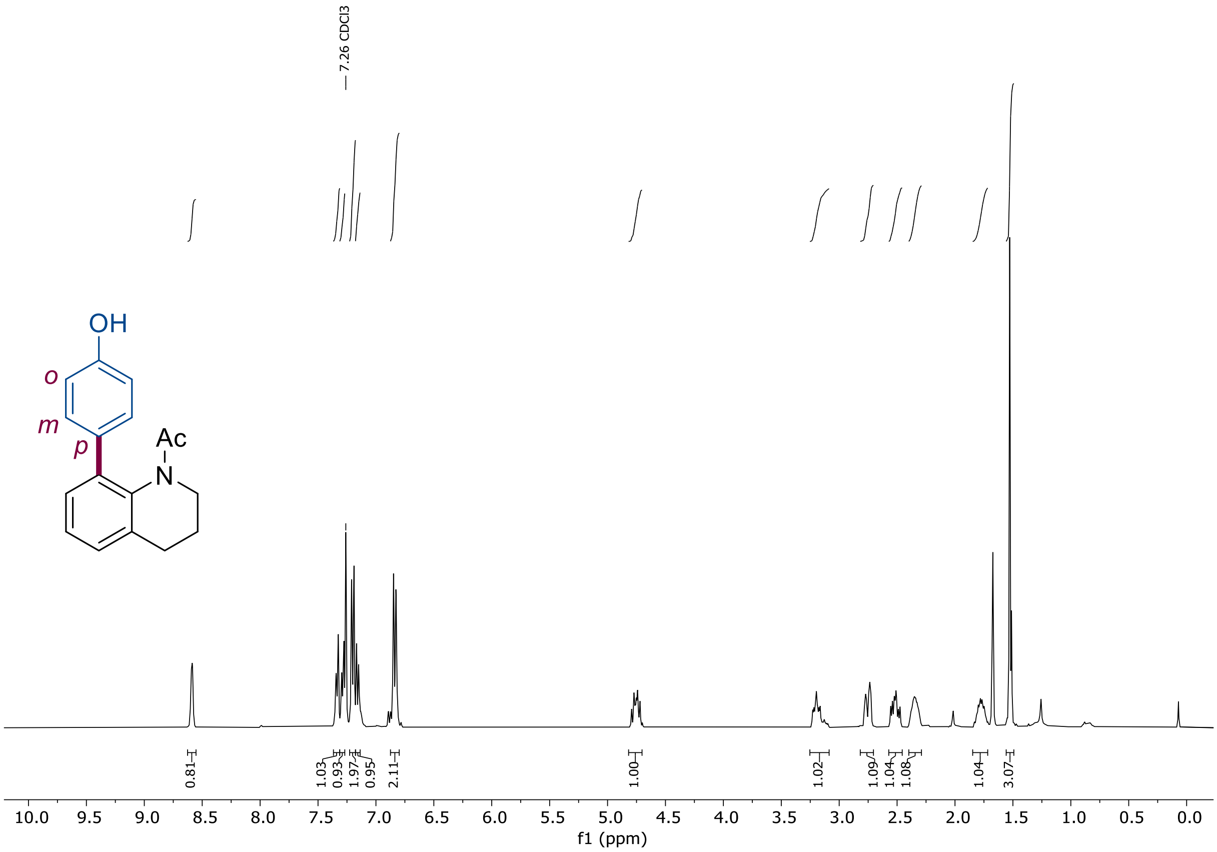

NMR Spectrum 58 ^{1}H NMR for 26, 400 MHz, $CDCl_3$, room temperature.

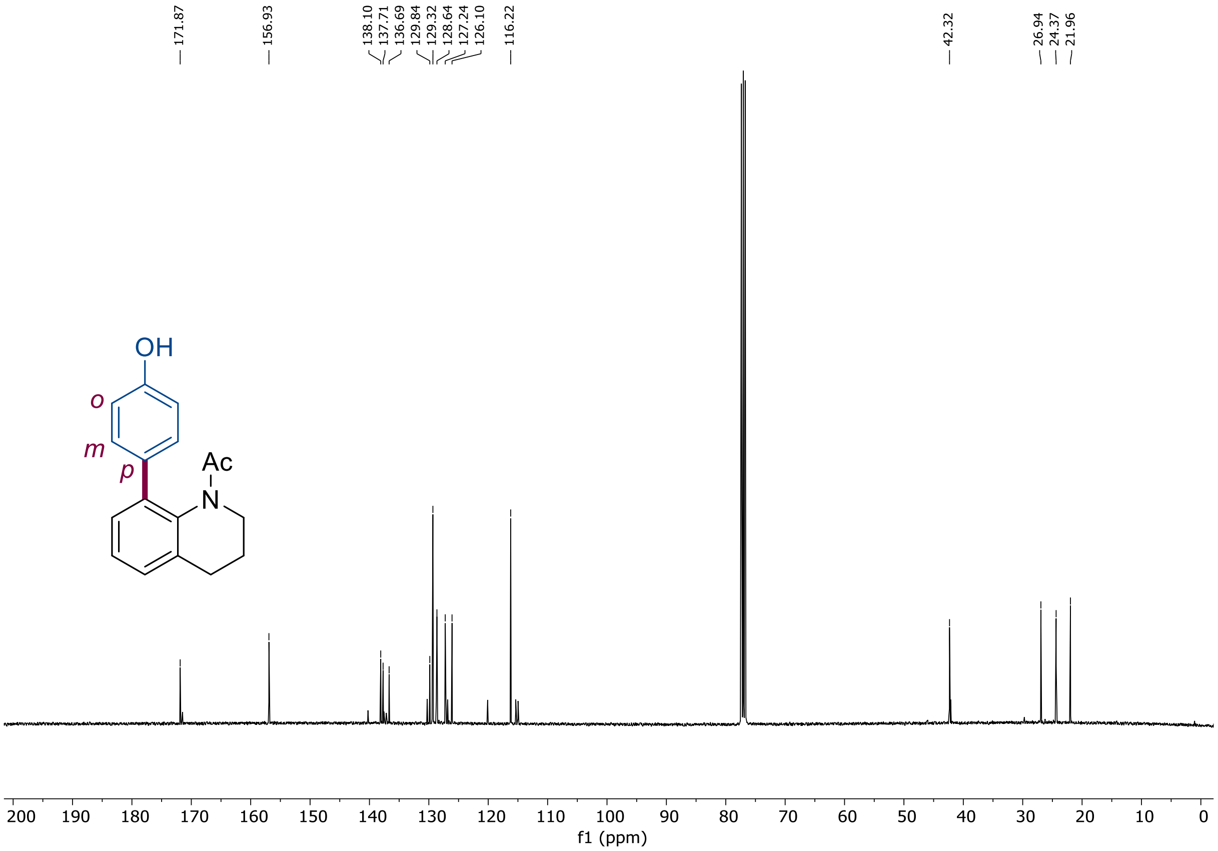

NMR Spectrum 59 ^{13}C NMR for 26, 101 MHz, $CDCl_3$, room temperature.

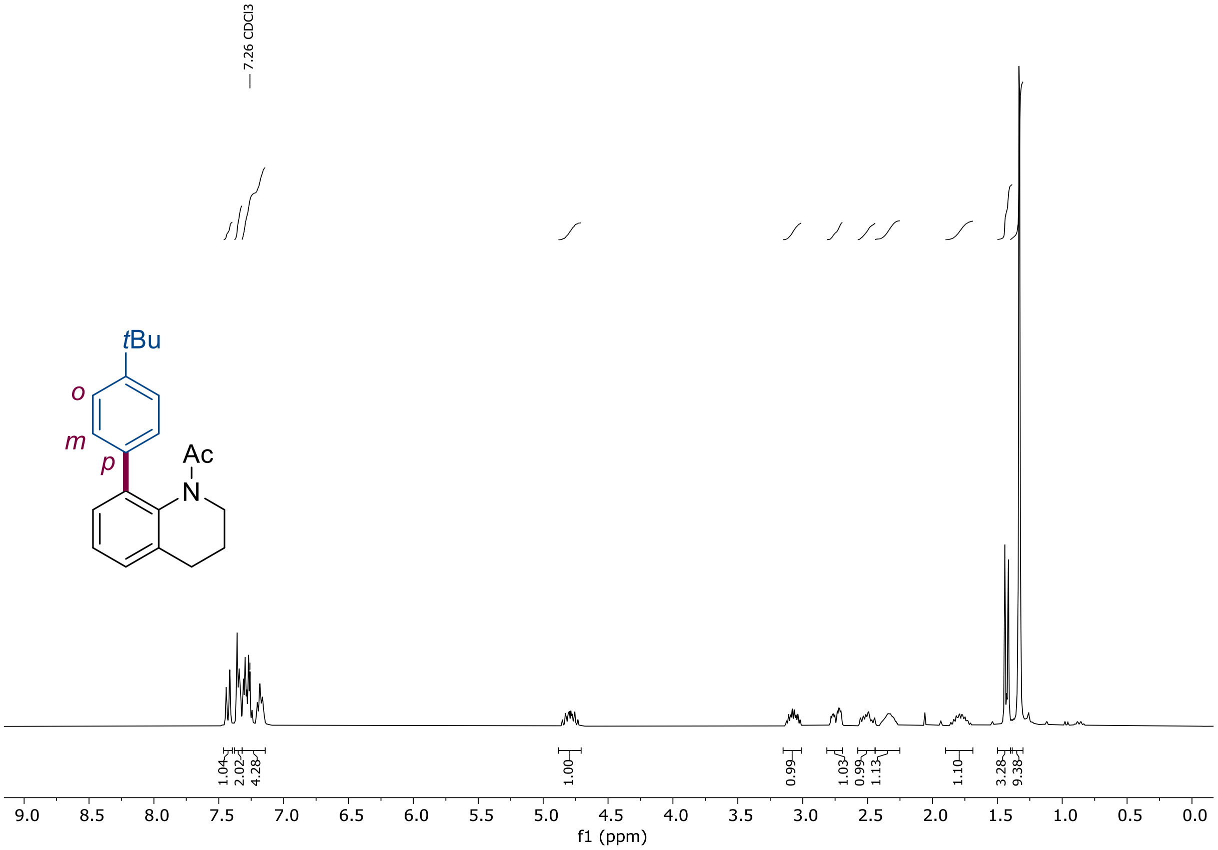

NMR Spectrum 60 ^{1}H NMR for 27, 300 MHz, $CDCl_3$, room temperature.

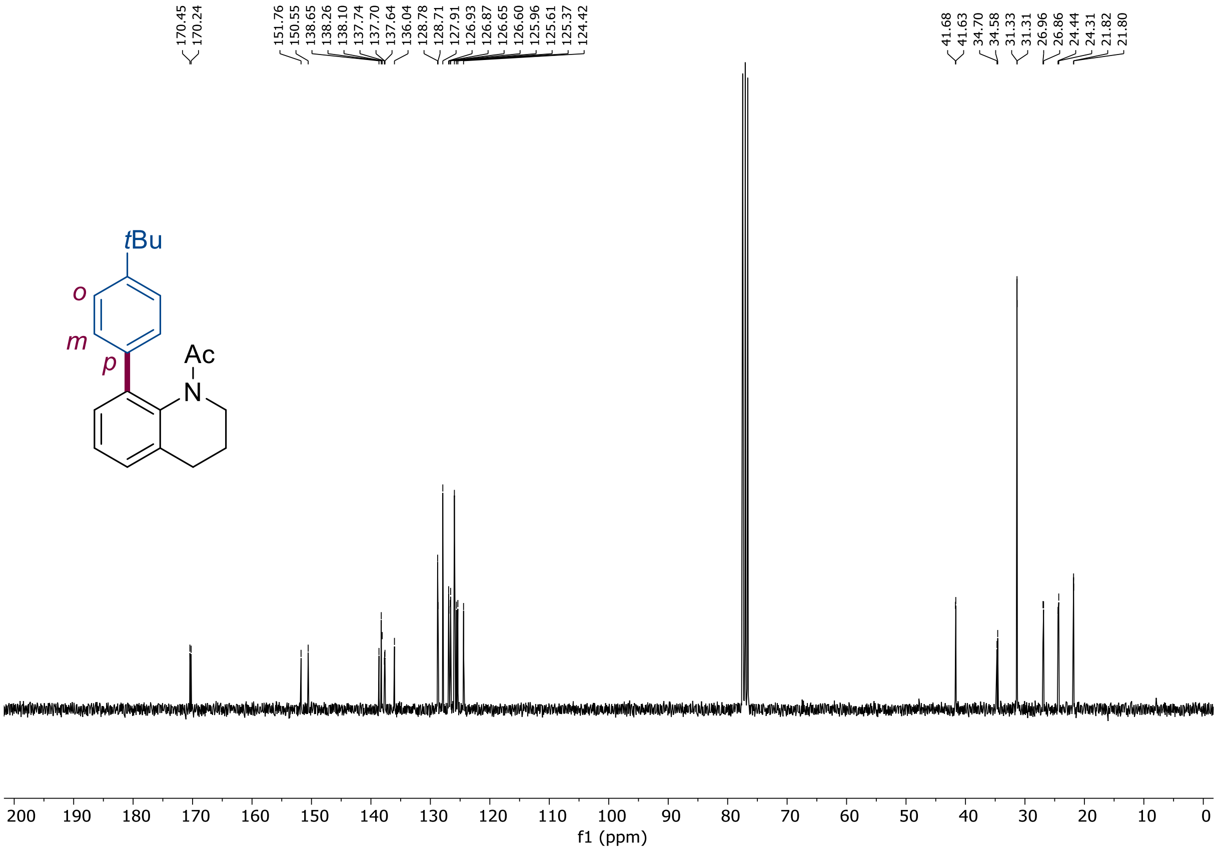

NMR Spectrum 61 ^{13}C **NMR for 27, 75 MHz, $CDCl_3$, room temperature.**

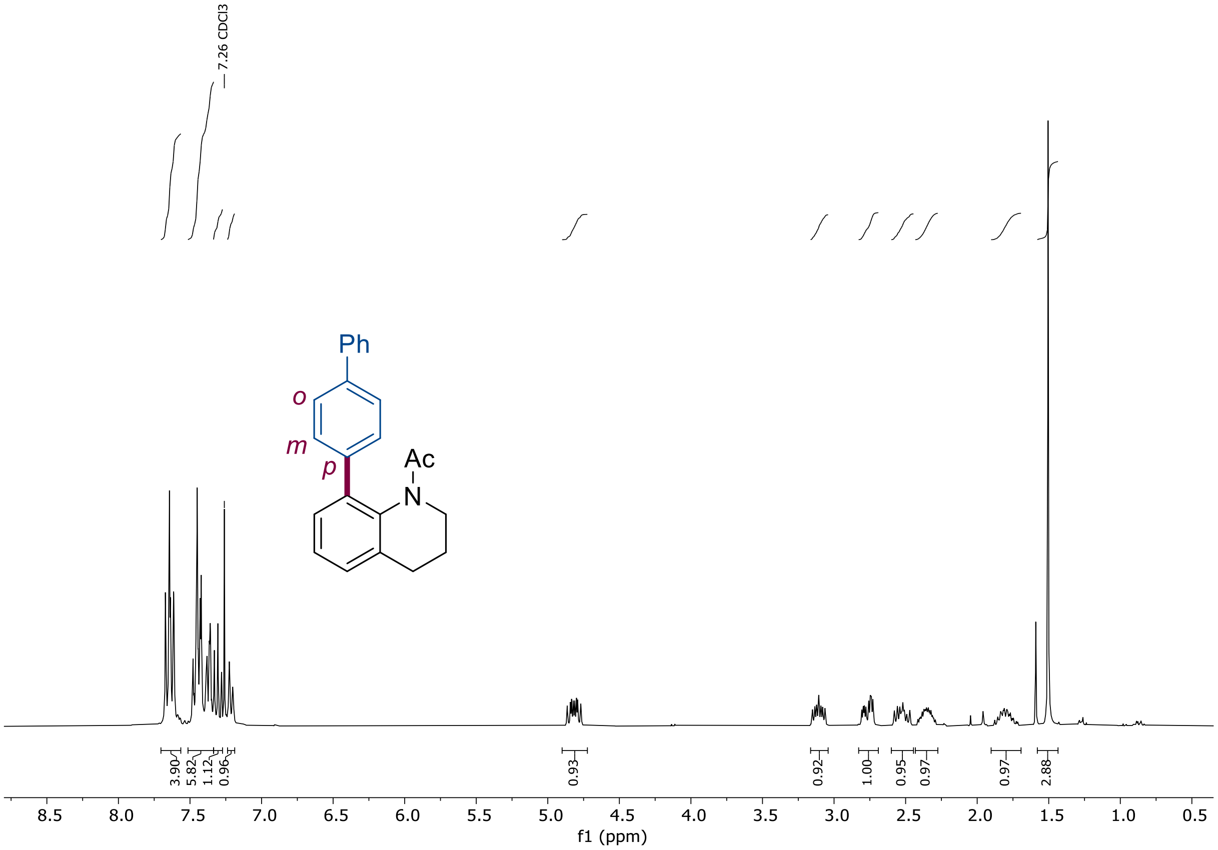

NMR Spectrum 62 ^{1}H **NMR for 28, 300 MHz, $CDCl_3$, room temperature.**

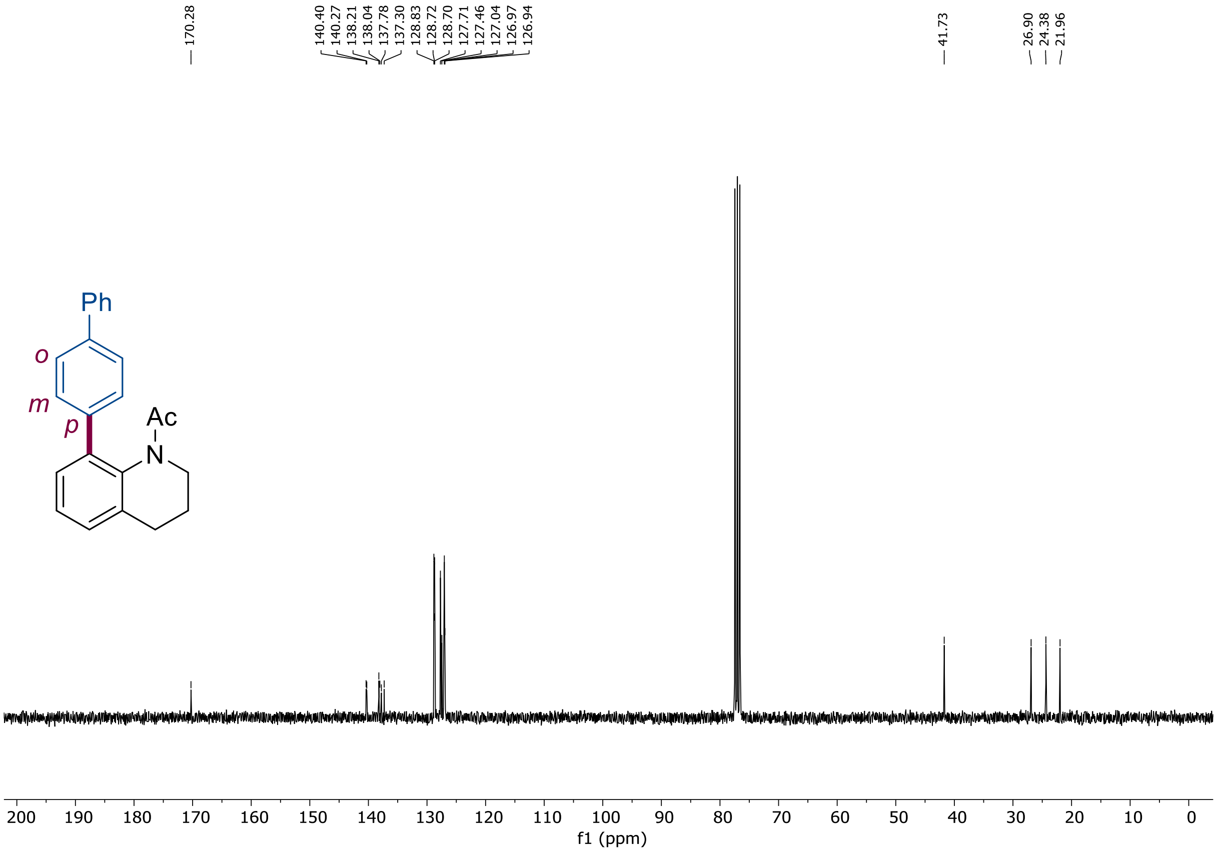

NMR Spectrum 63 ^{13}C NMR for 28, 75 MHz, CDCl₃, room temperature.

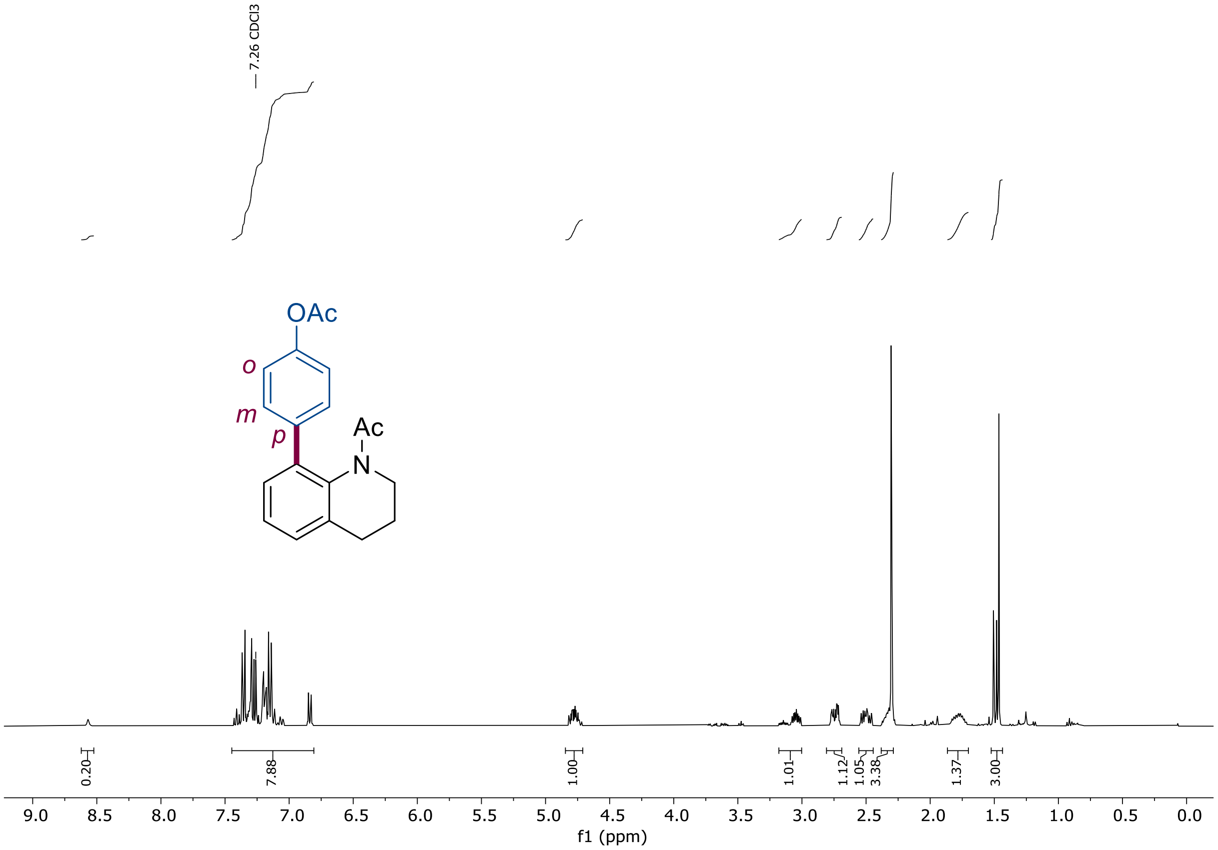

NMR Spectrum 64 ^{1}H NMR for 29, 400 MHz, CDCl₃, room temperature.

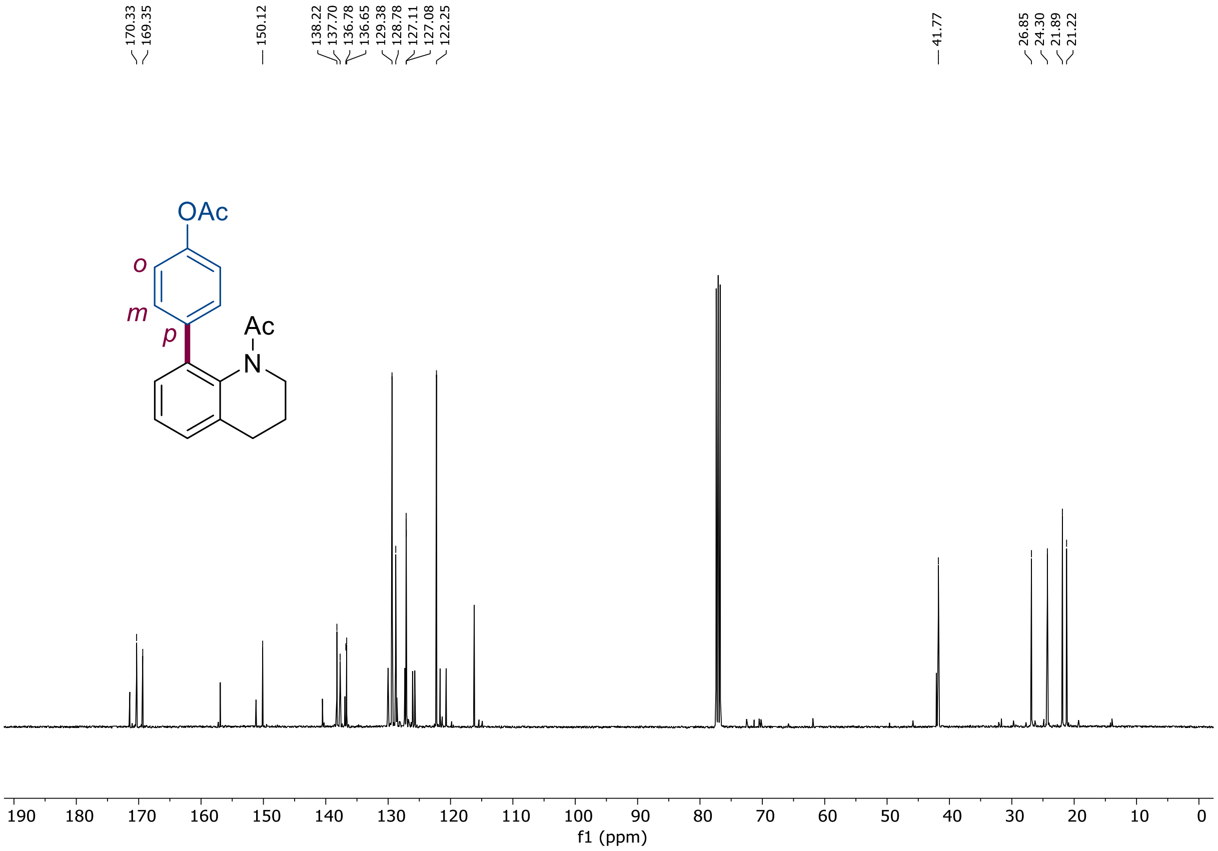

NMR Spectrum 65 ^{13}C NMR for 29, 101 MHz, $CDCl_3$, room temperature.

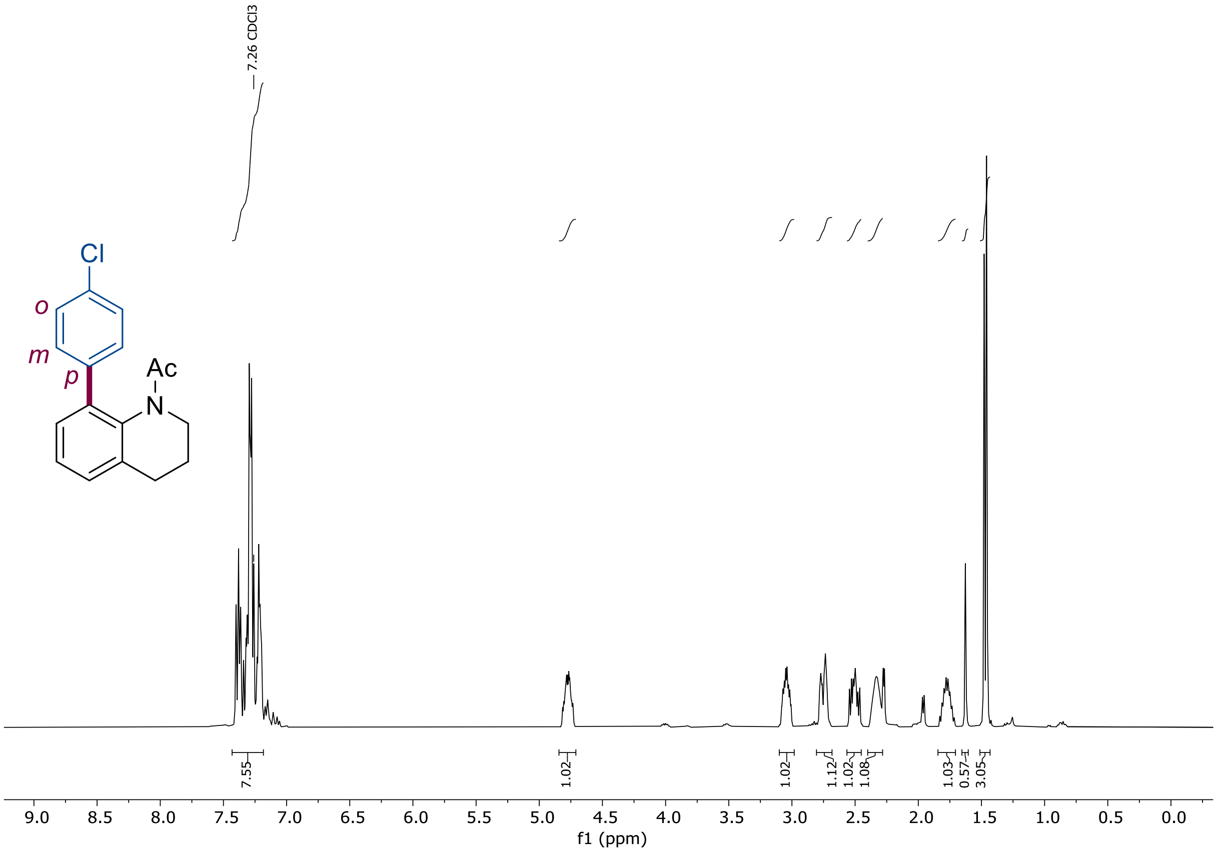

NMR Spectrum 66 ^{1}H NMR for 30, 400 MHz, $CDCl_3$, room temperature.

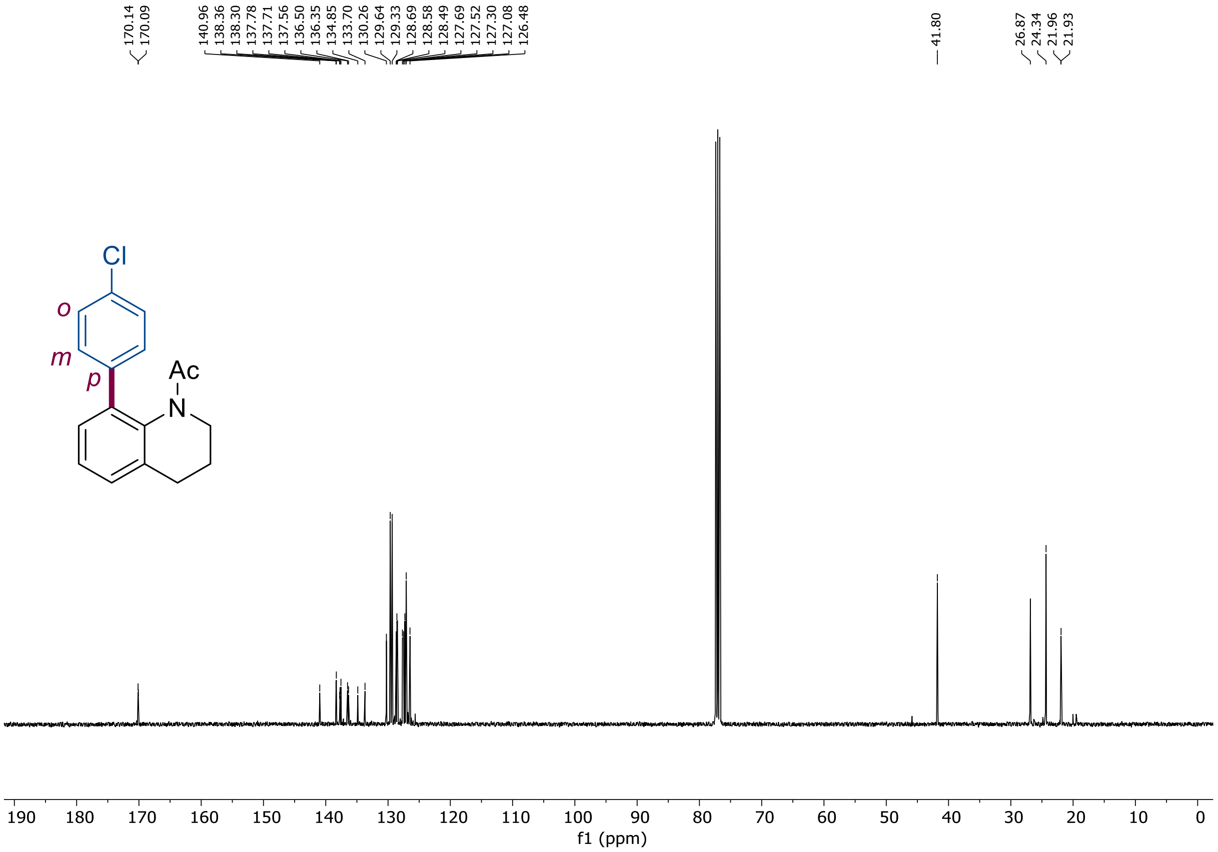

NMR Spectrum 67 ^{13}C NMR for 30, 101 MHz, CDCl$_3$, room temperature.

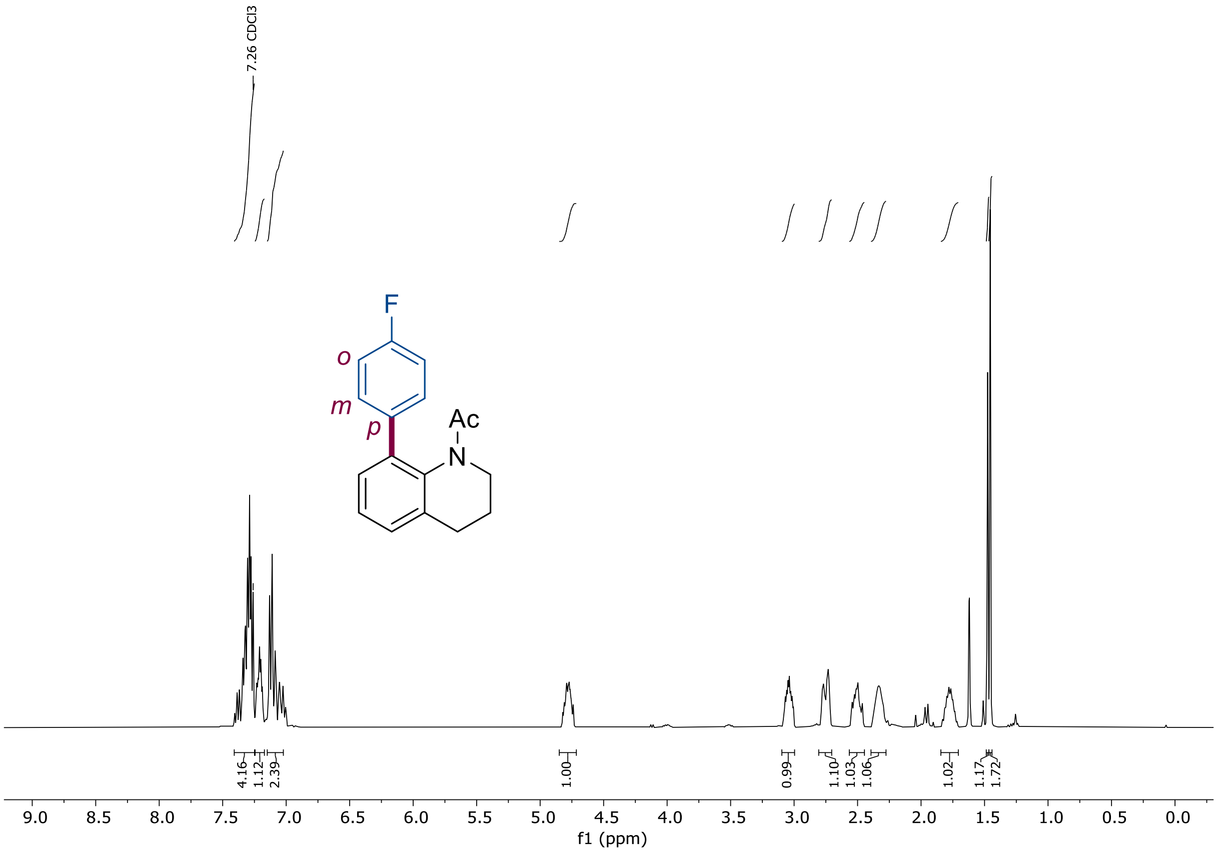

NMR Spectrum 68 ^{1}H NMR for 31, 400 MHz, CDCl$_3$, room temperature.

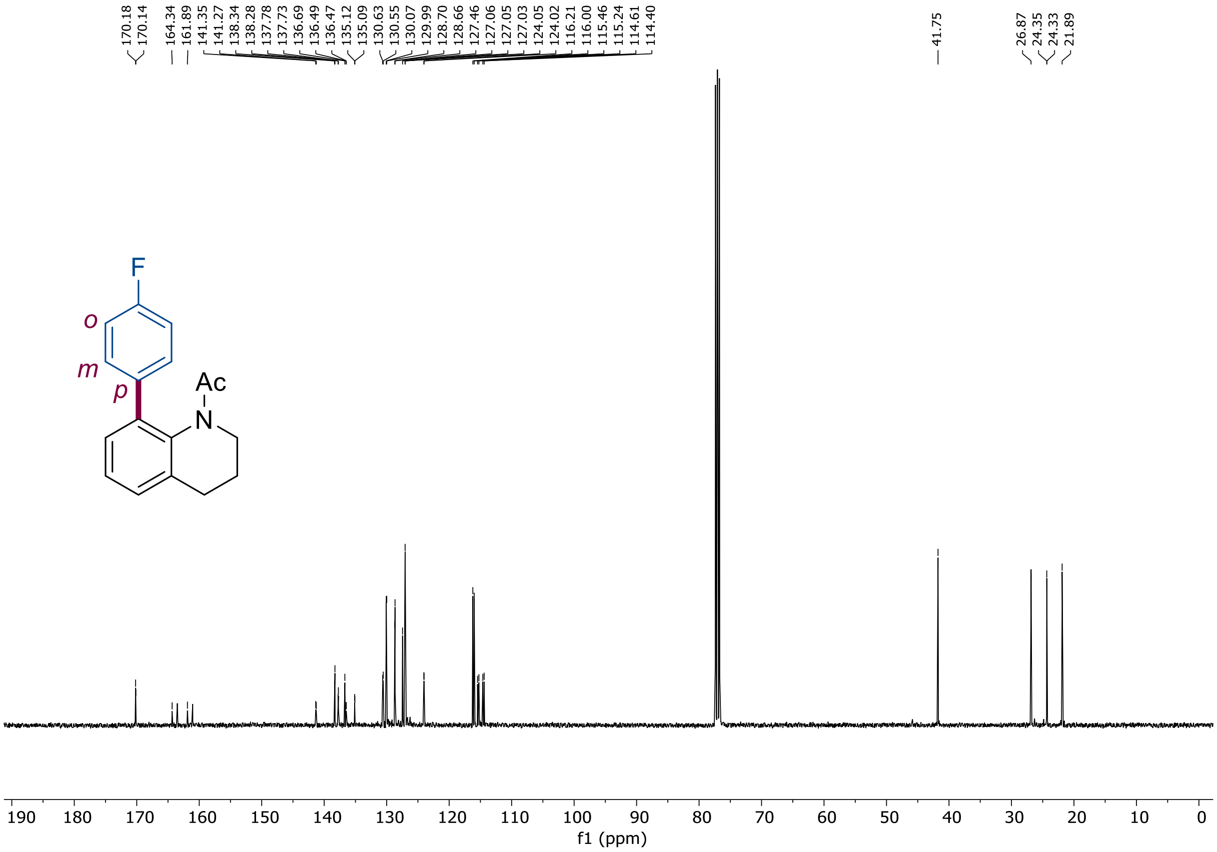

NMR Spectrum 69 ^{13}C NMR for 31, 101 MHz, $CDCl_3$, room temperature.

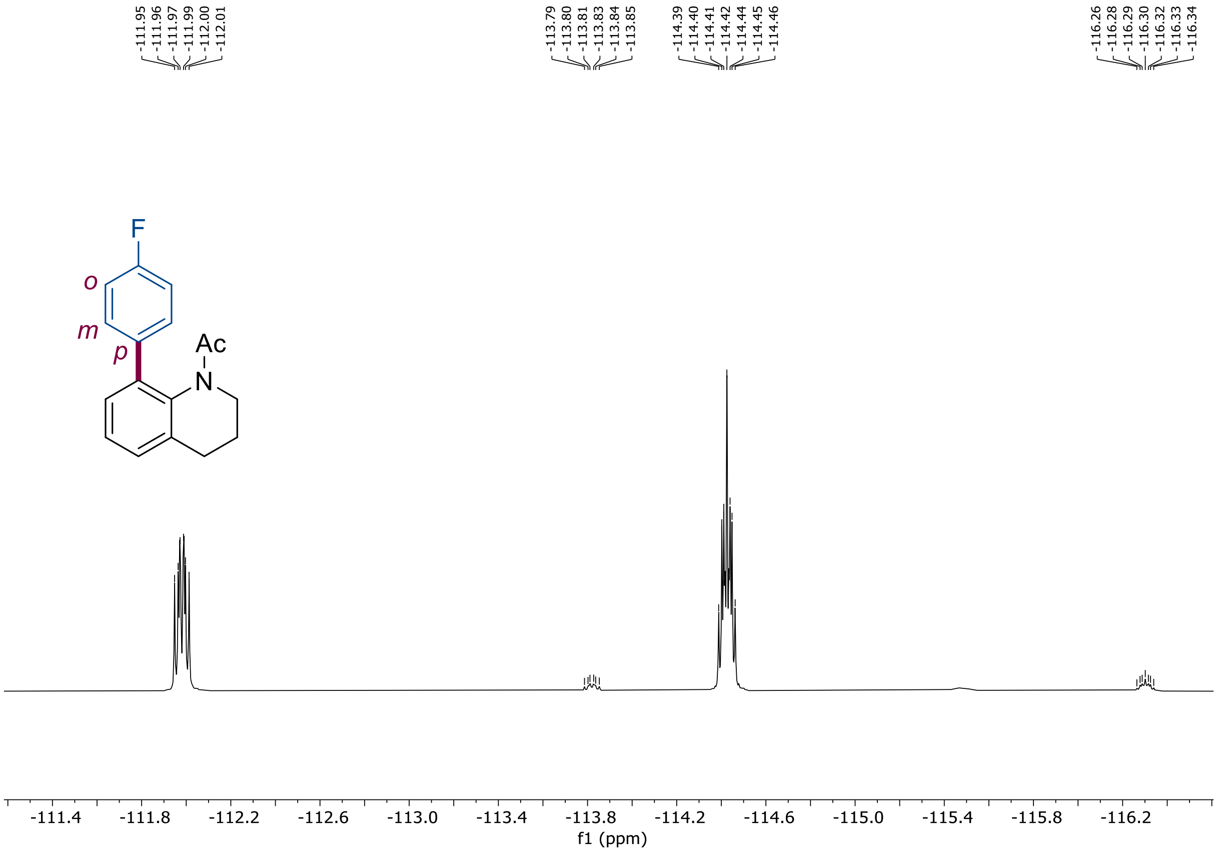

NMR Spectrum 70 ^{19}F NMR for 31, 377 MHz, $CDCl_3$, room temperature.

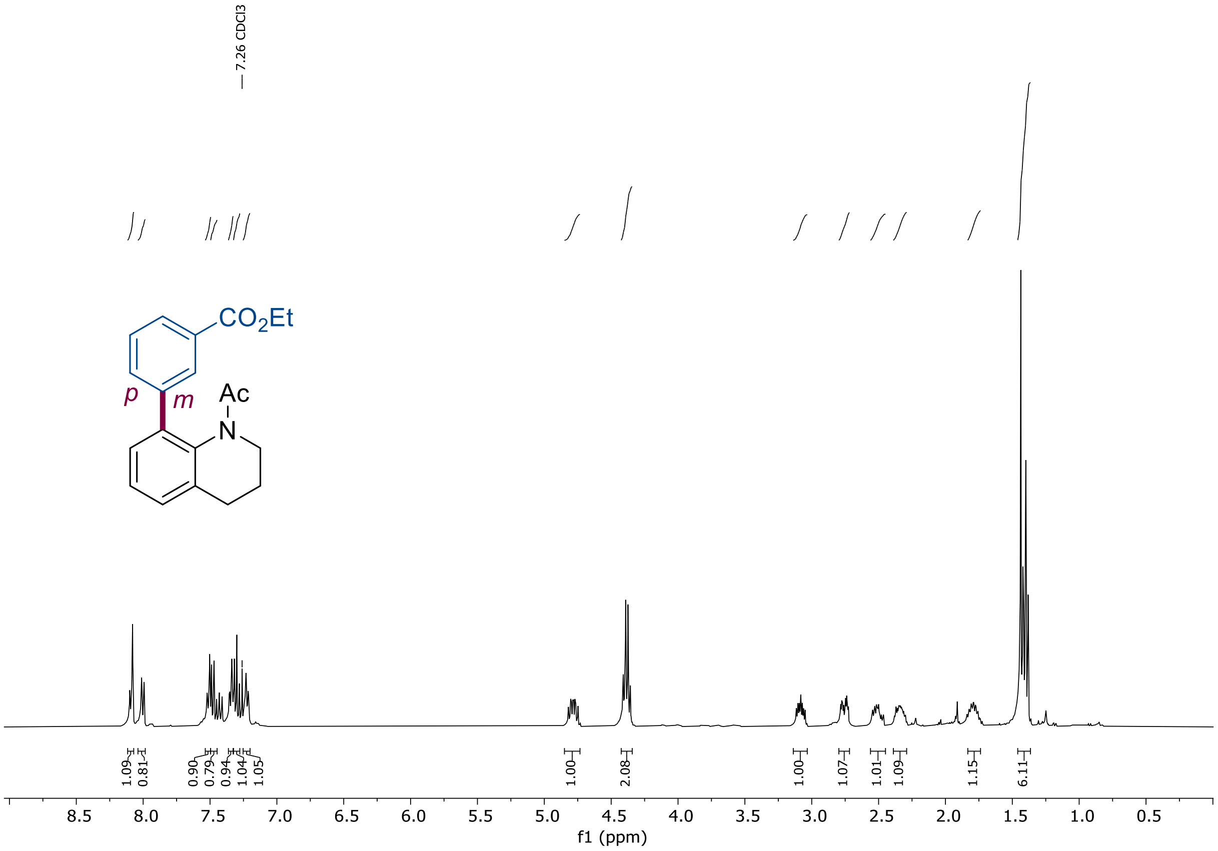

NMR Spectrum 71 ^{1}H NMR for 32, 400 MHz, CDCl$_3$, room temperature.

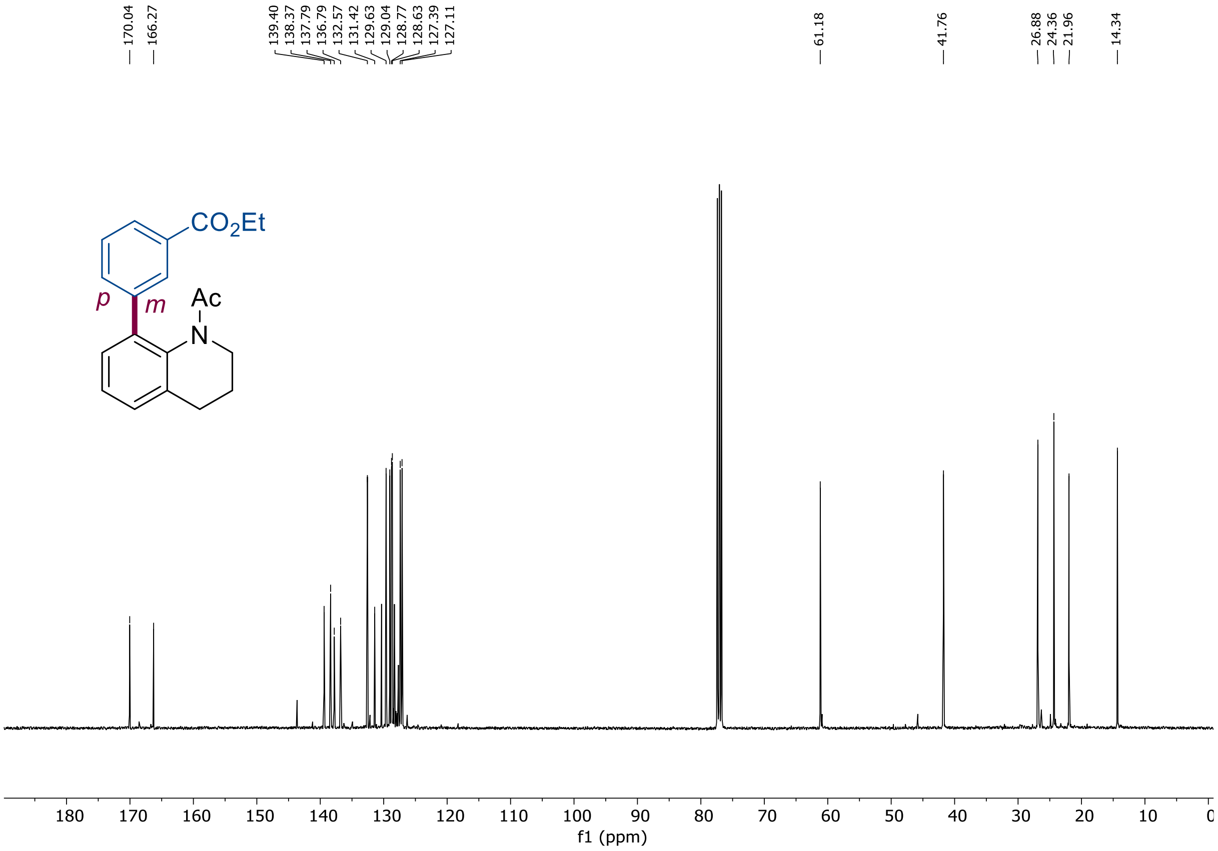

NMR Spectrum 72 ^{13}C NMR for 32, 101 MHz, CDCl$_3$, room temperature.

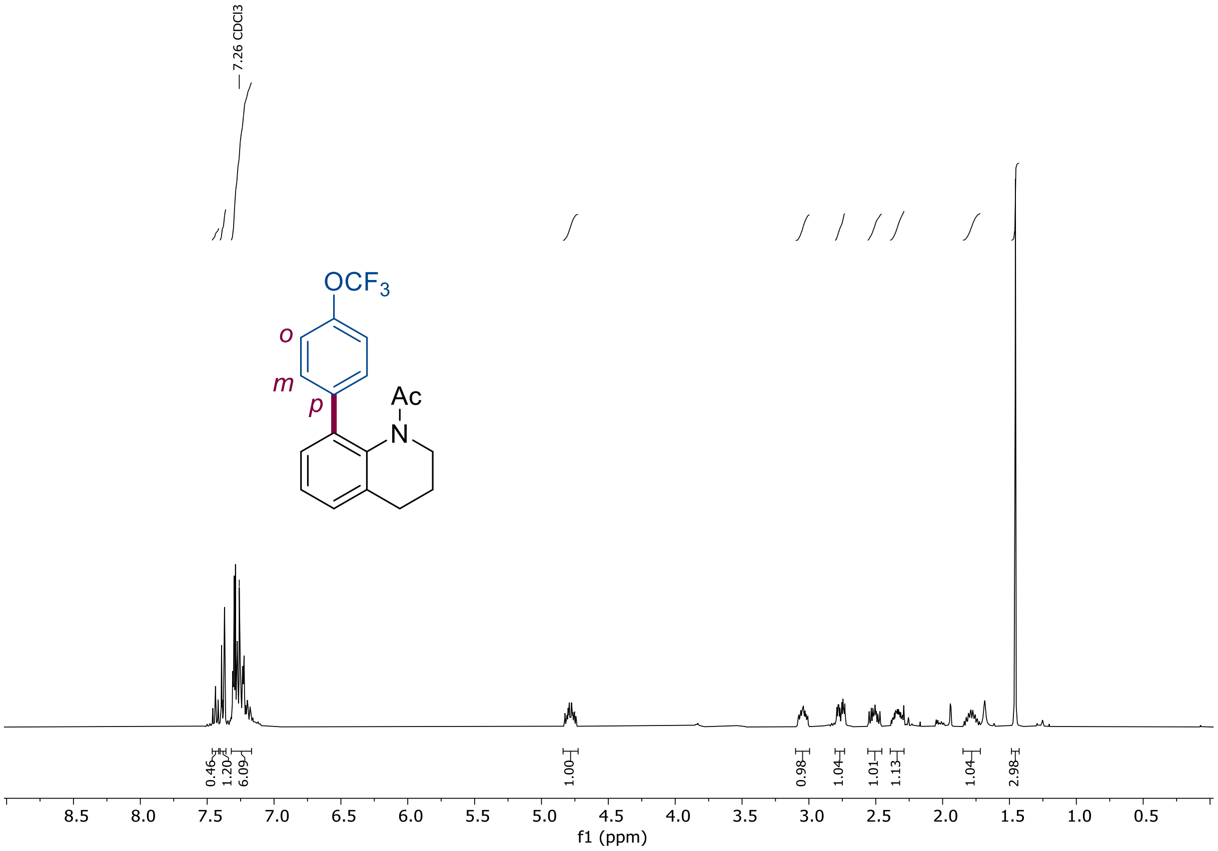

NMR Spectrum 73 ^{1}H NMR for 33, 400 MHz, $CDCl_3$, room temperature.

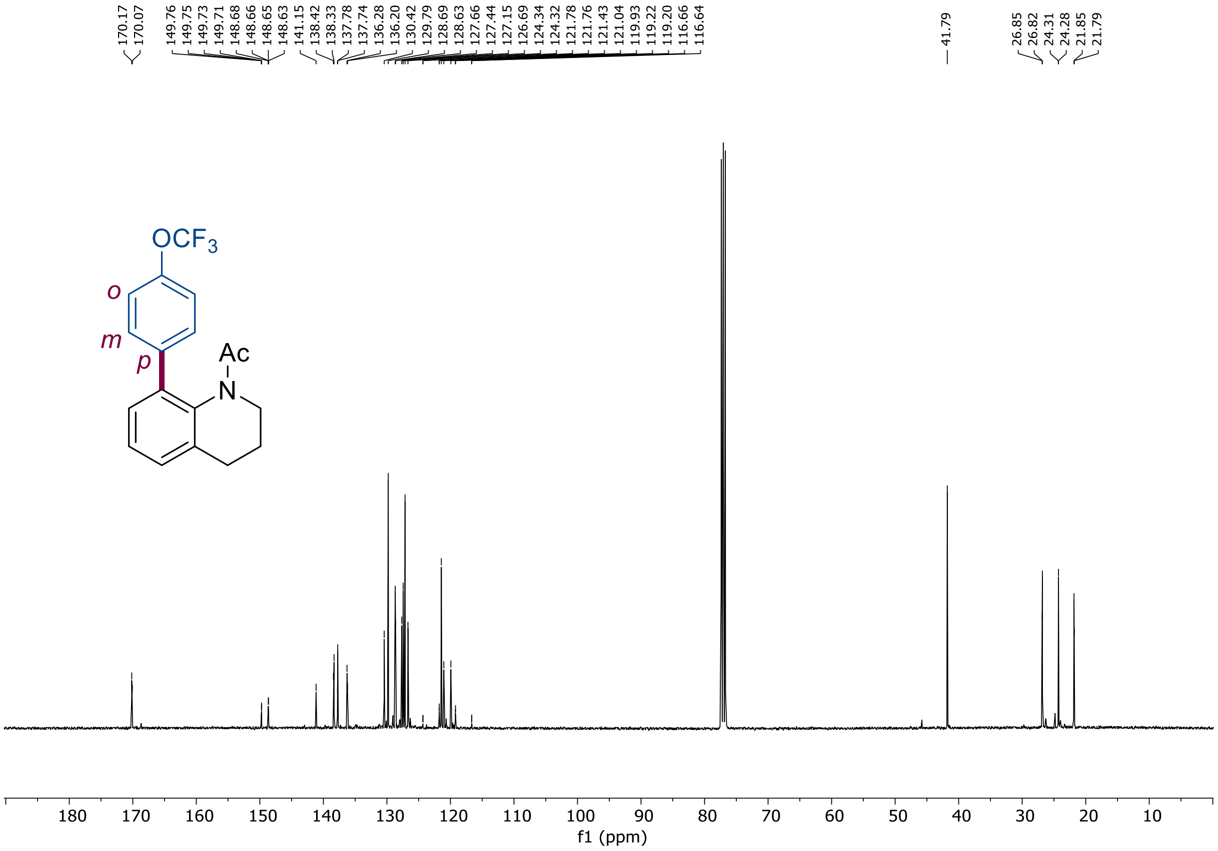

NMR Spectrum 74 ^{13}C NMR for 33, 101 MHz, $CDCl_3$, room temperature.

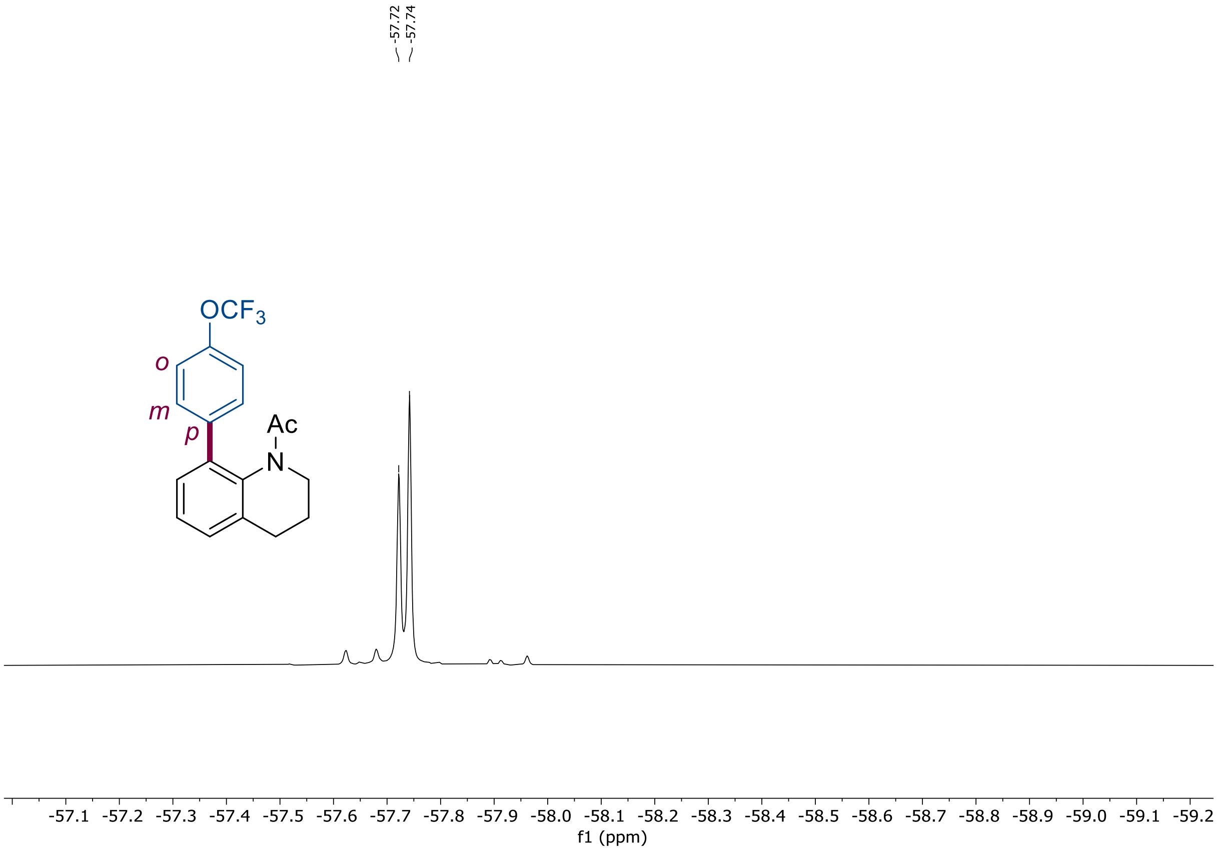

NMR Spectrum 75 ^{19}F NMR for 33, 377 MHz, CDCl3, room temperature.

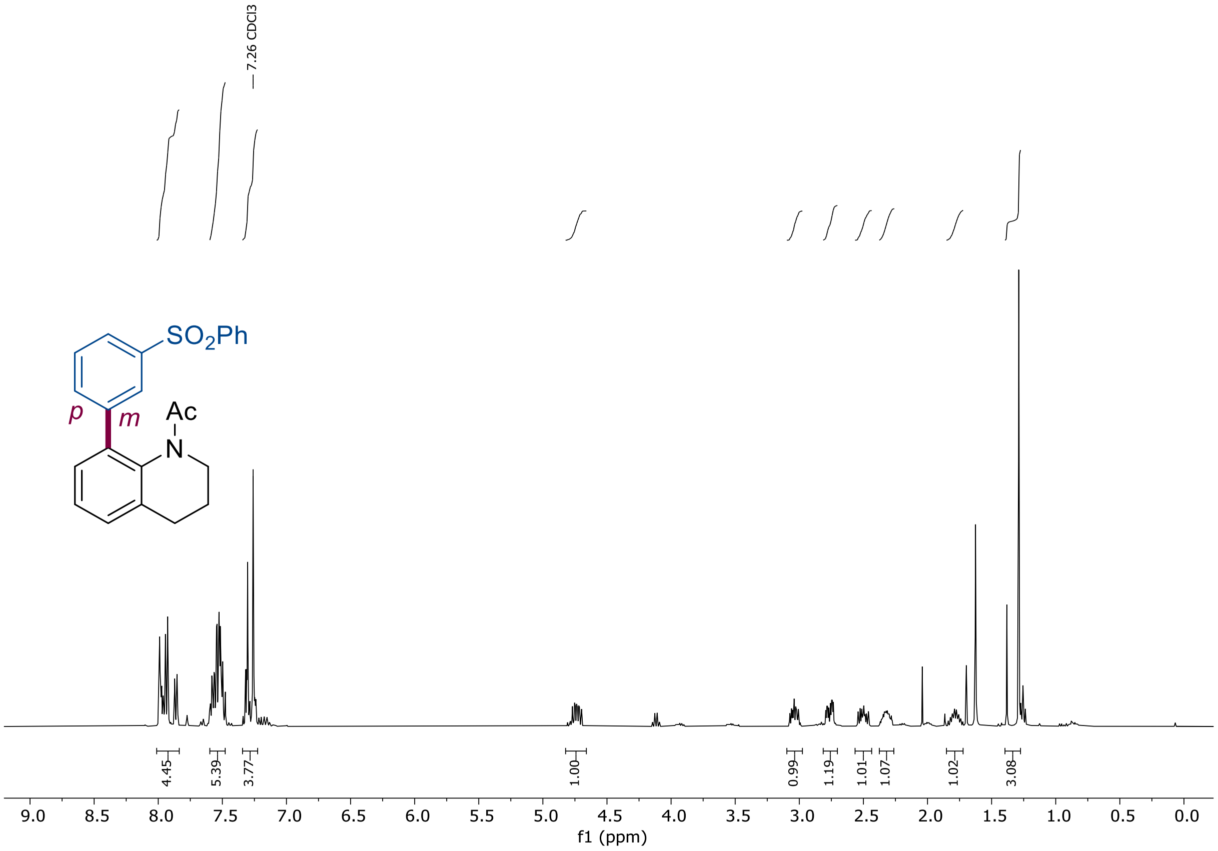

NMR Spectrum 76 ^{1}H NMR for 34, 400 MHz, CDCl3, room temperature.

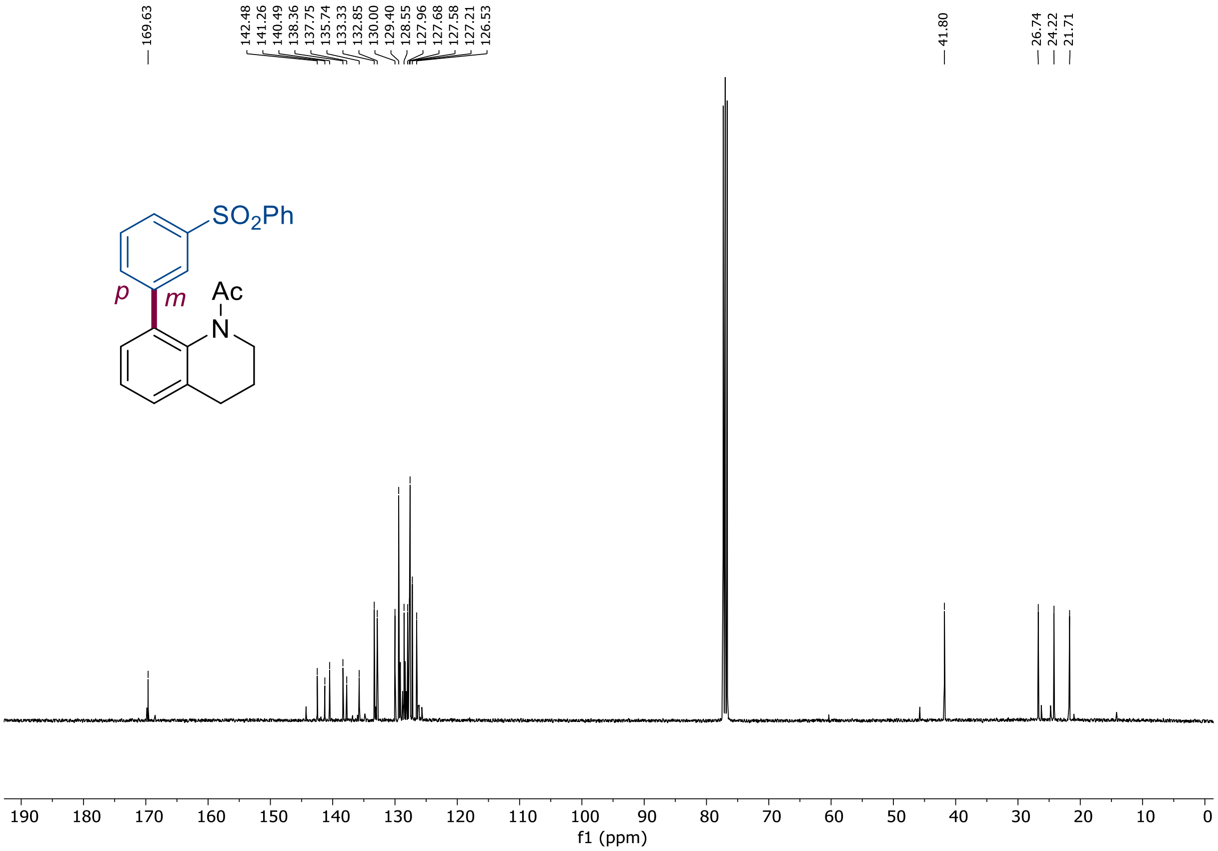

NMR Spectrum 77 ^{13}C NMR for 34, 101 MHz, $CDCl_3$, room temperature.

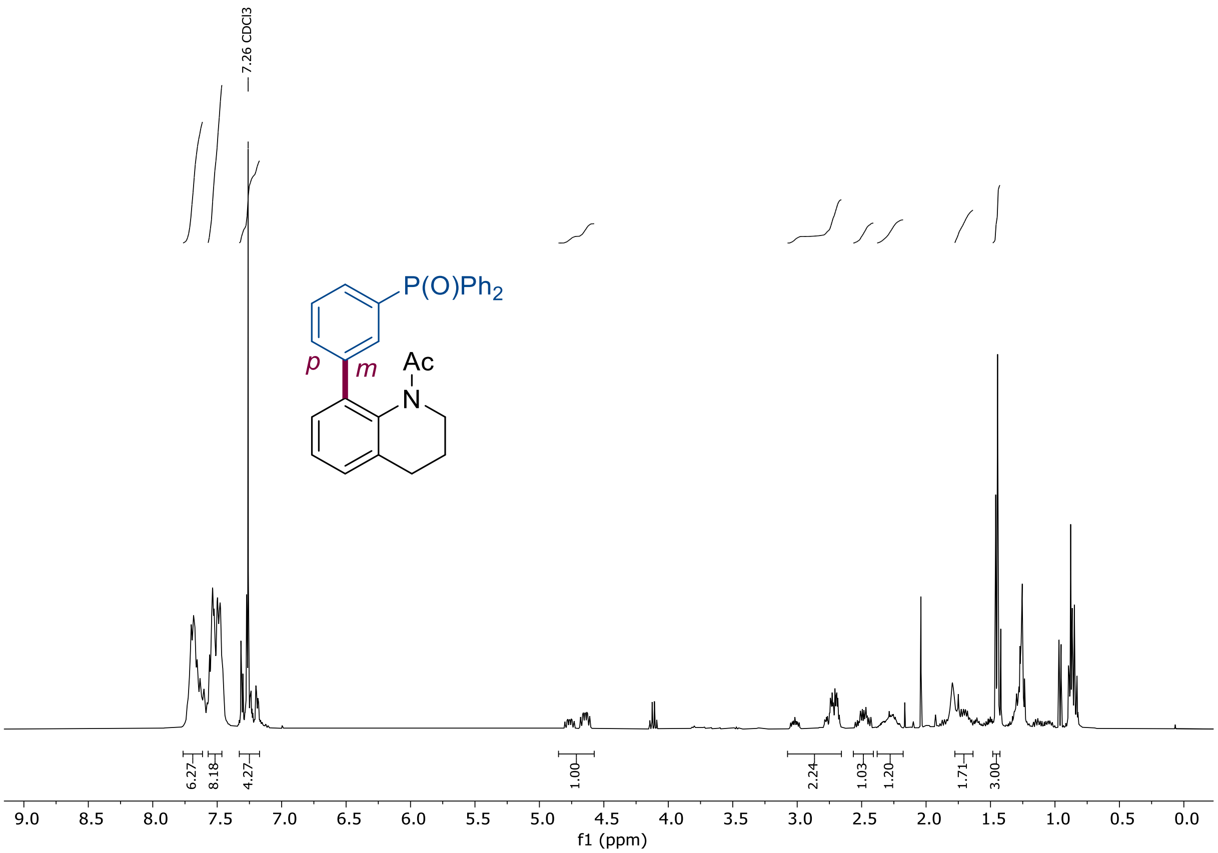

NMR Spectrum 78 ^{1}H NMR for 35, 400 MHz, $CDCl_3$, room temperature.

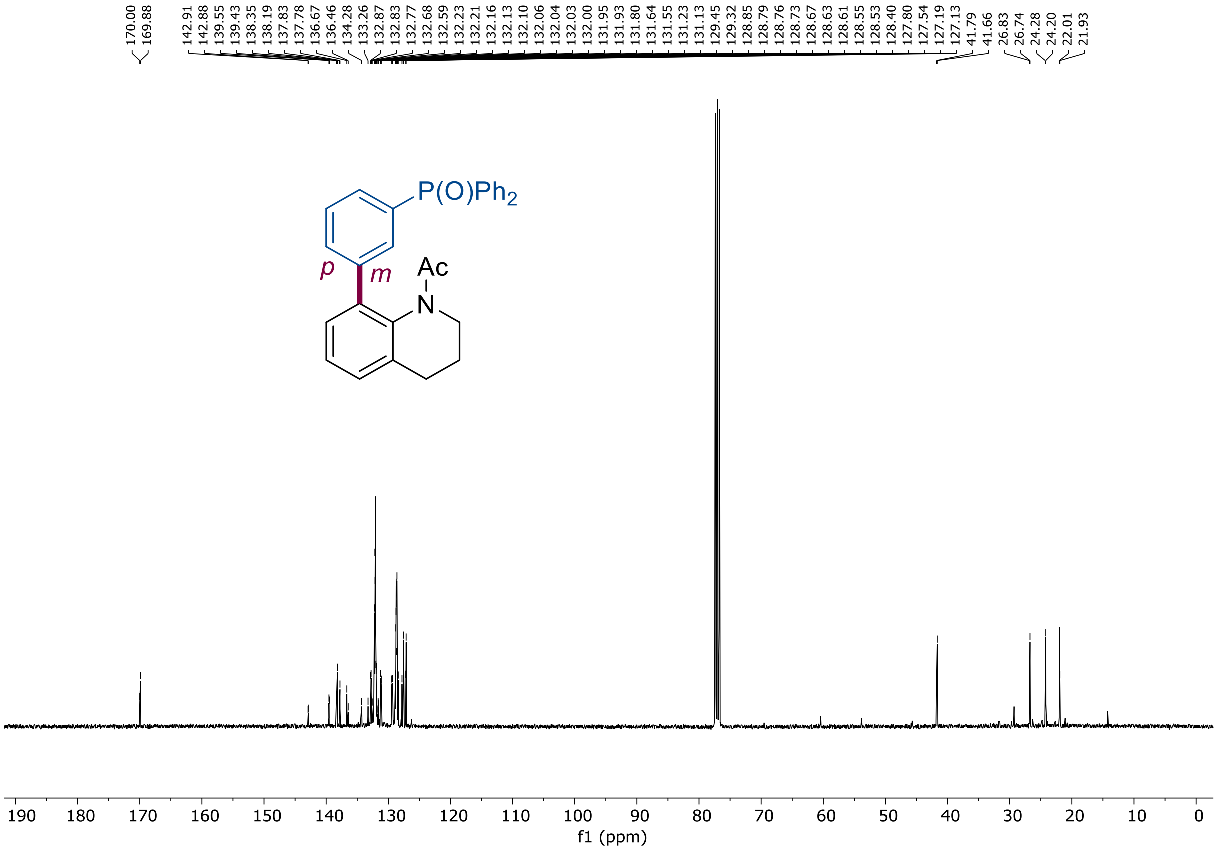

NMR Spectrum 79 ^{13}C NMR for 35, 101 MHz, $CDCl_3$, room temperature.

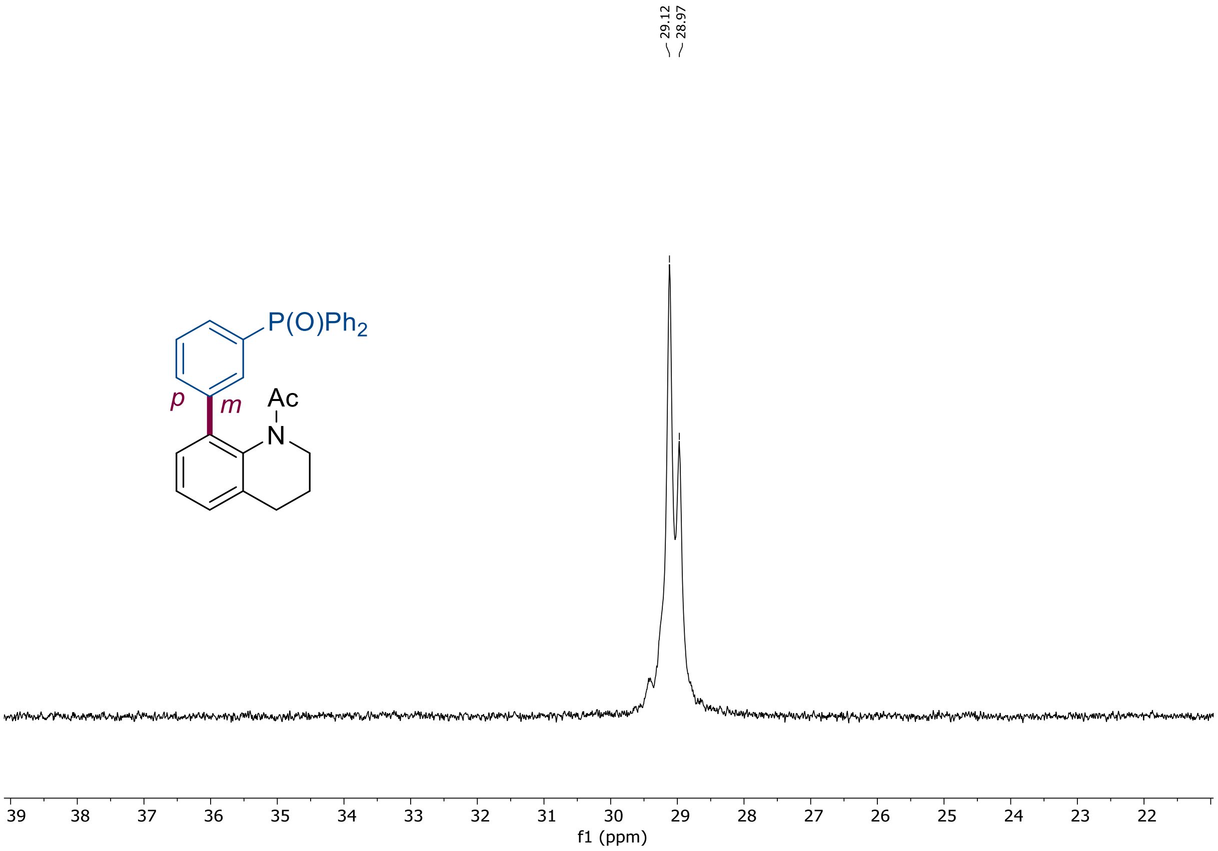

NMR Spectrum 80 ^{31}P NMR for 35, 162 MHz, $CDCl_3$, room temperature.

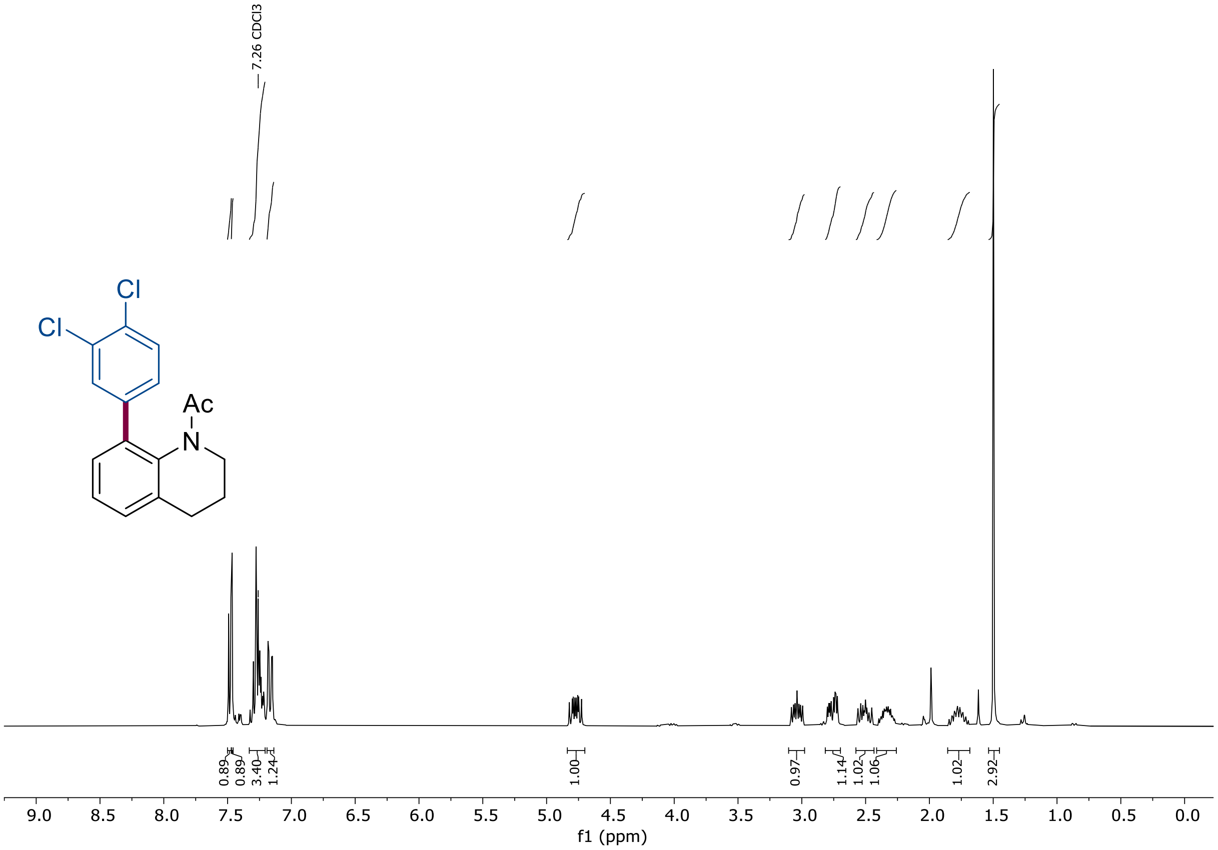

NMR Spectrum 81 ^{1}H NMR for 36, 300 MHz, $CDCl_3$, room temperature.

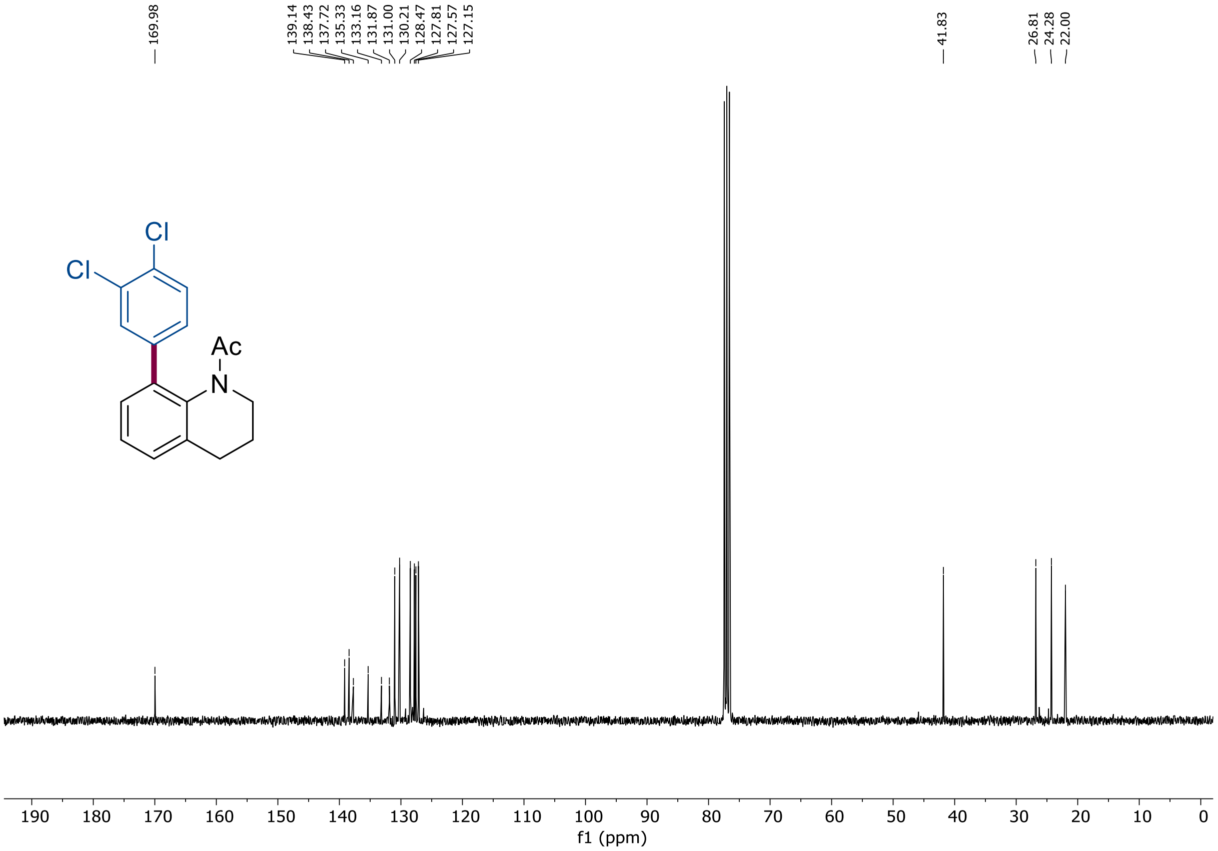

NMR Spectrum 82 ^{13}C NMR for 36, 75 MHz, $CDCl_3$, room temperature.

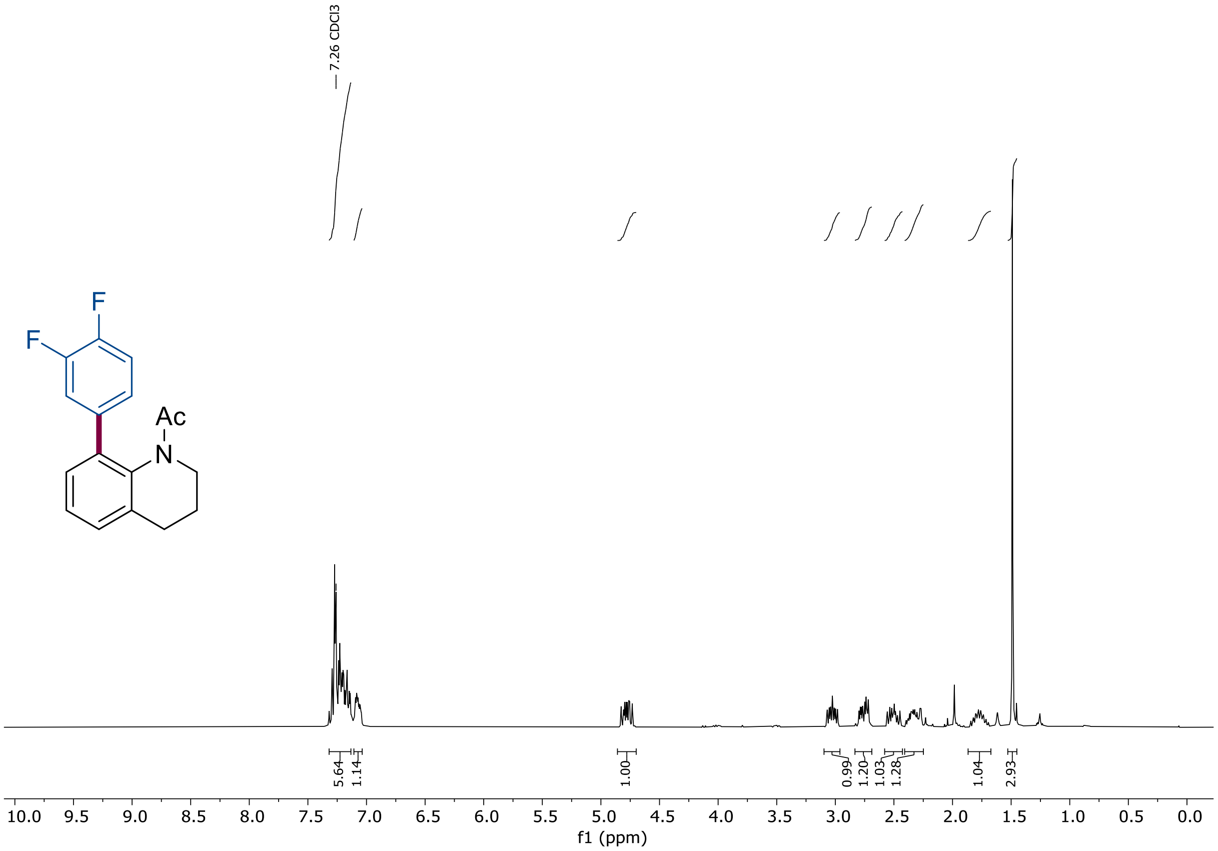

NMR Spectrum 83 ^{1}H NMR for 37, 300 MHz, $CDCl_3$, room temperature.

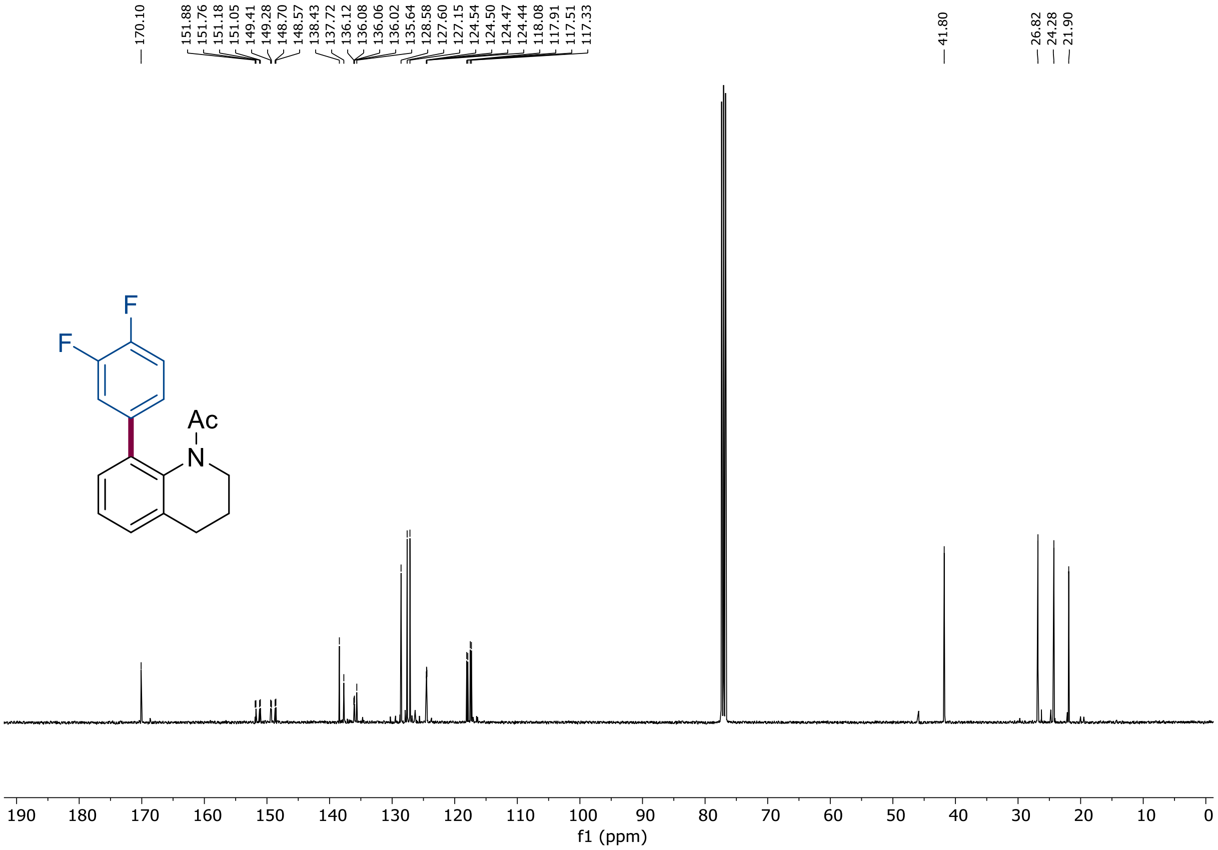

NMR Spectrum 84 ^{13}C NMR for 37, 101 MHz, $CDCl_3$, room temperature.

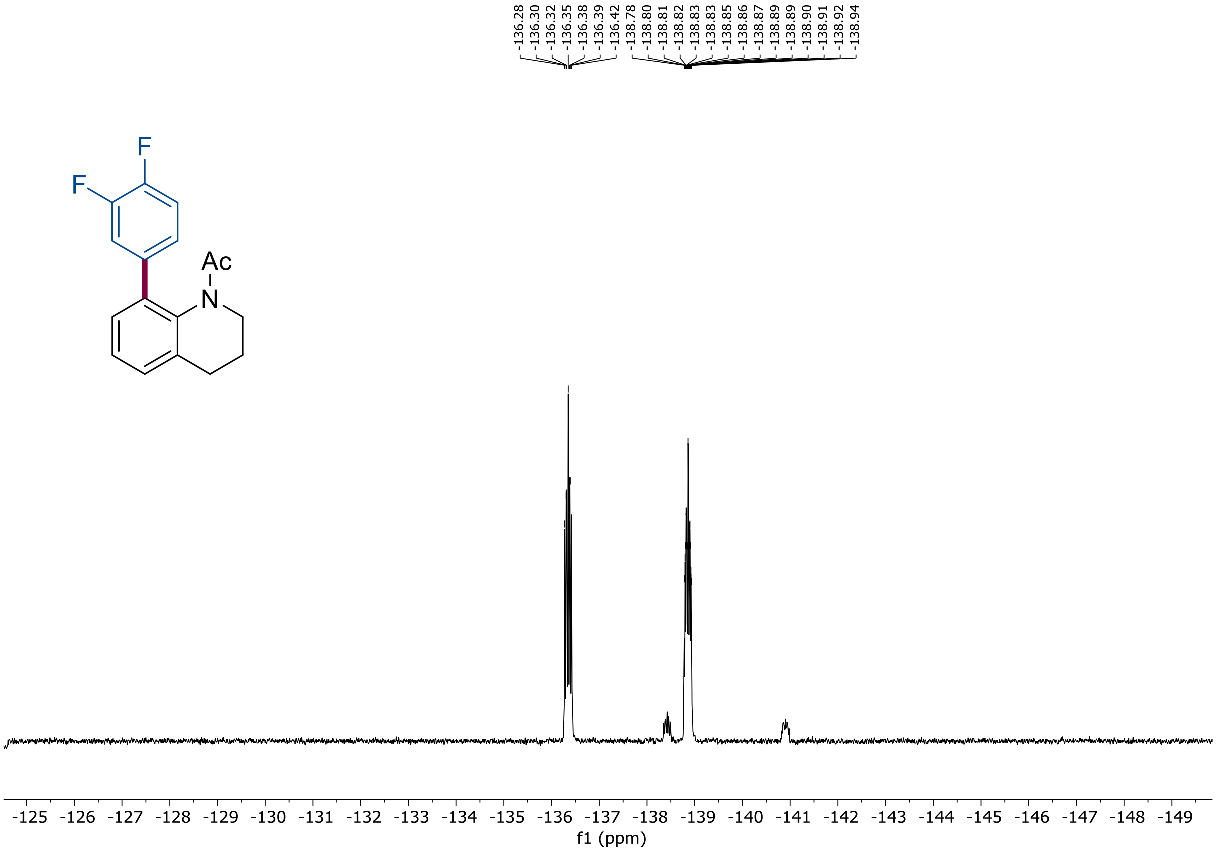

NMR Spectrum 85 ^{19}F NMR for 37, 282 MHz, $CDCl_3$, room temperature.

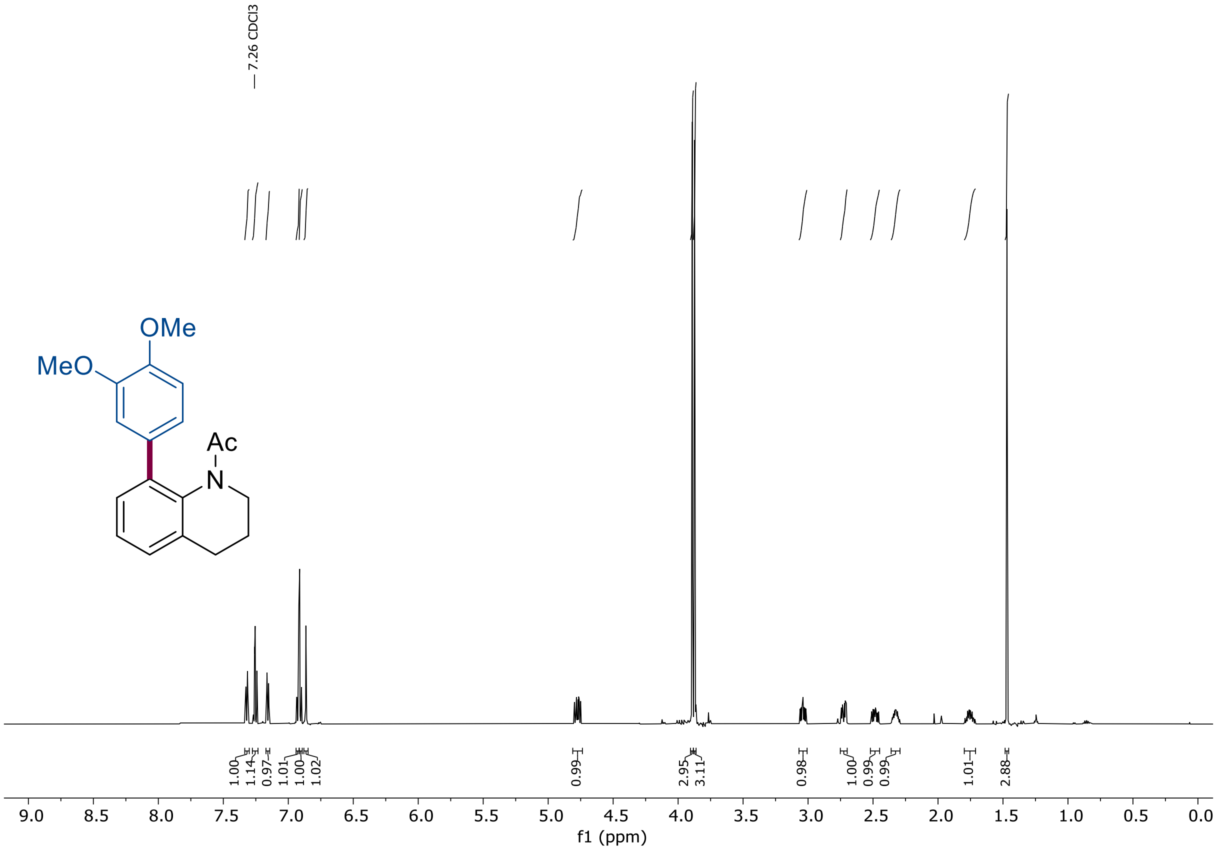

NMR Spectrum 86 ^{1}H NMR for 38, 600 MHz, $CDCl_3$, room temperature.

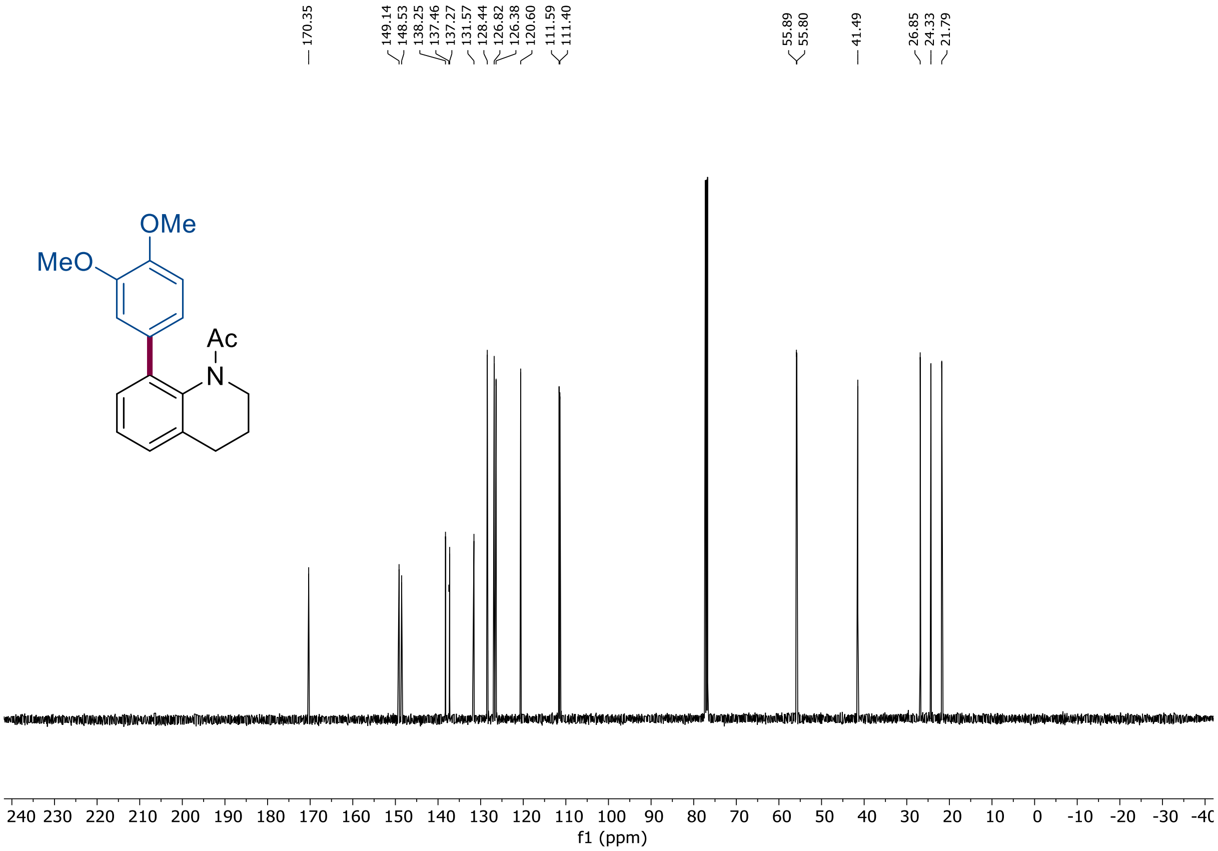

NMR Spectrum 87 ^{13}C NMR for 38, 126 MHz, $CDCl_3$, room temperature.

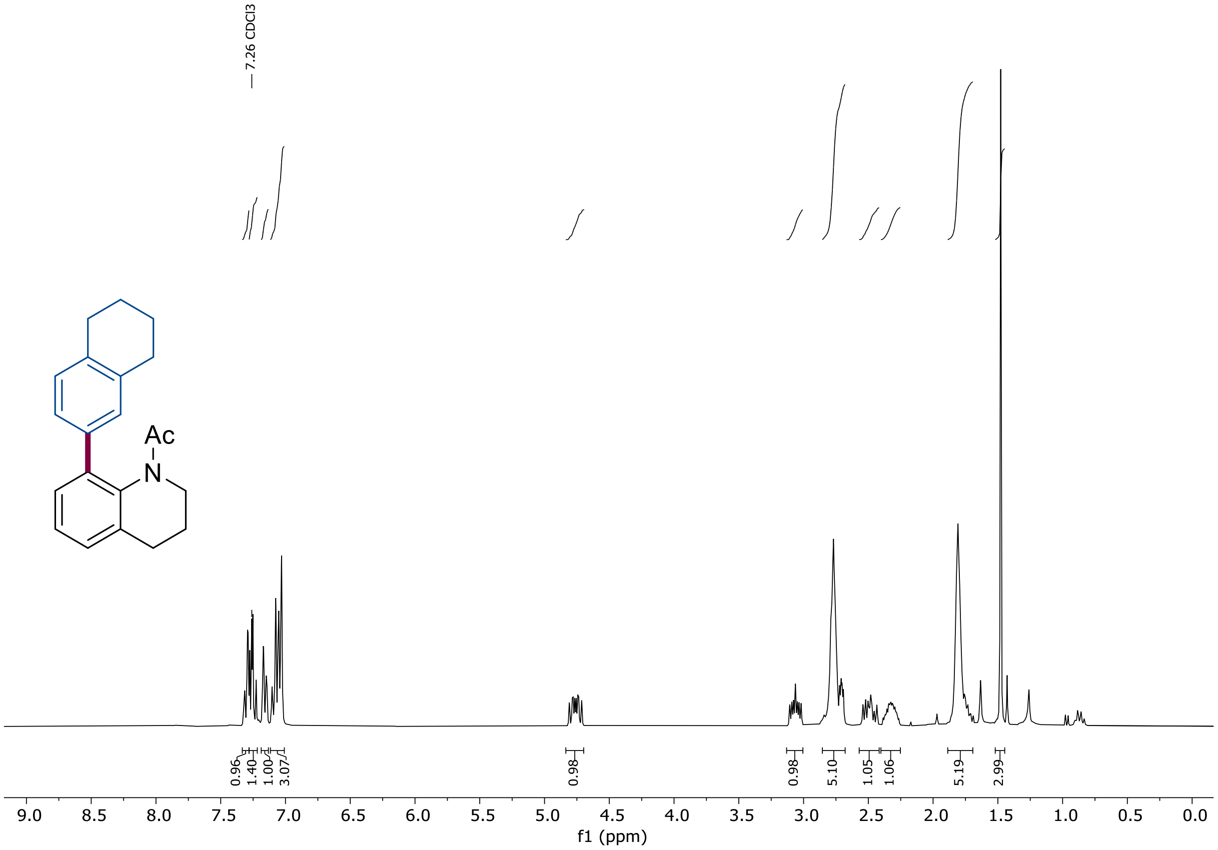

NMR Spectrum 88 ^{1}H NMR for 39, 300 MHz, $CDCl_3$, room temperature.

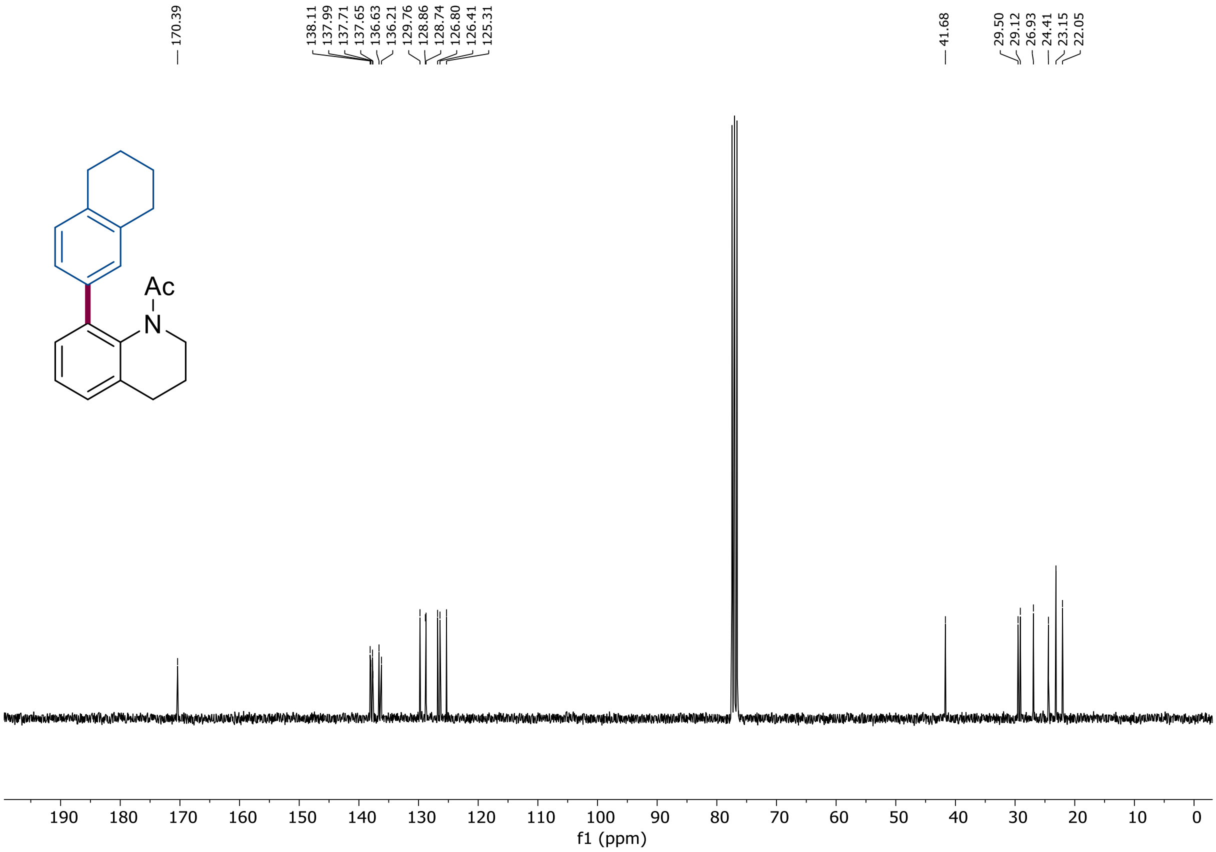

NMR Spectrum 89 ^{13}C NMR for 39, 75 MHz, $CDCl_3$, room temperature.

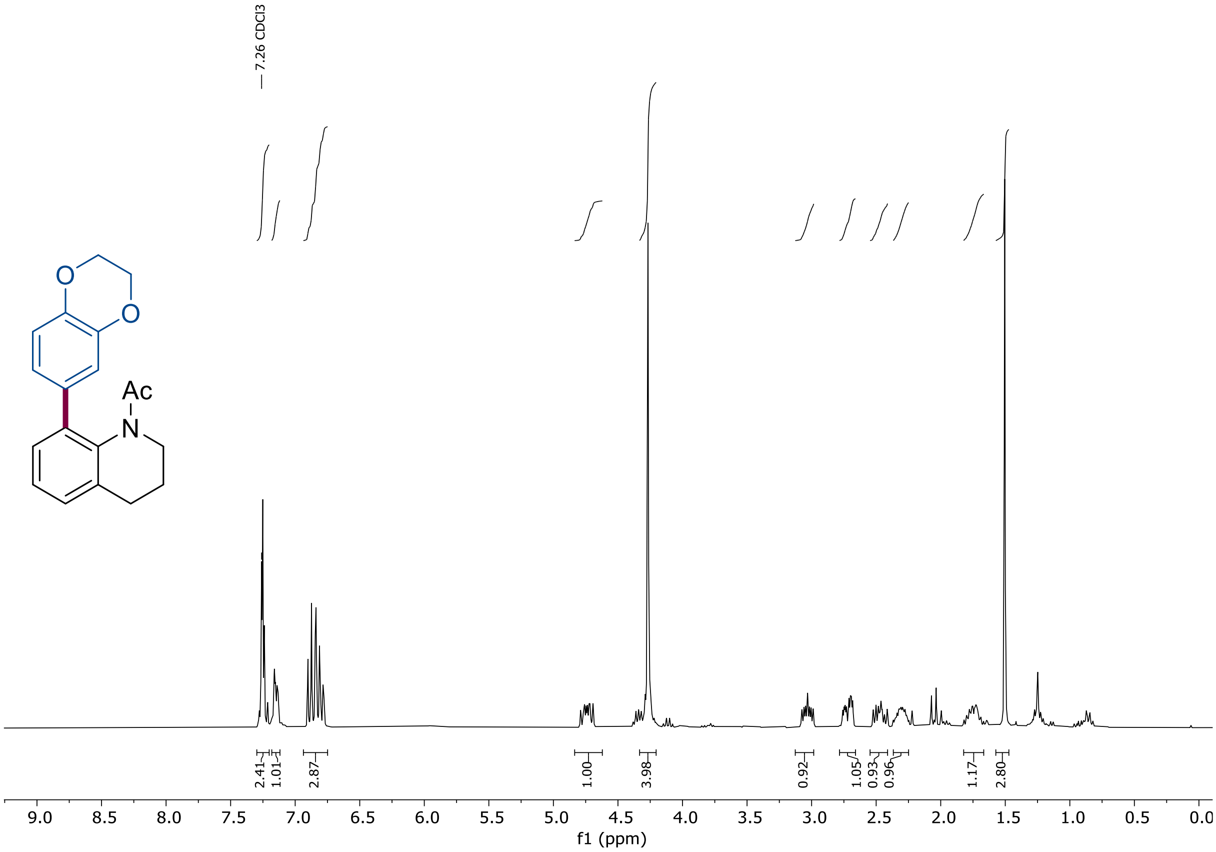

NMR Spectrum 90 ^{1}H NMR for 40, 300 MHz, $CDCl_3$, room temperature.

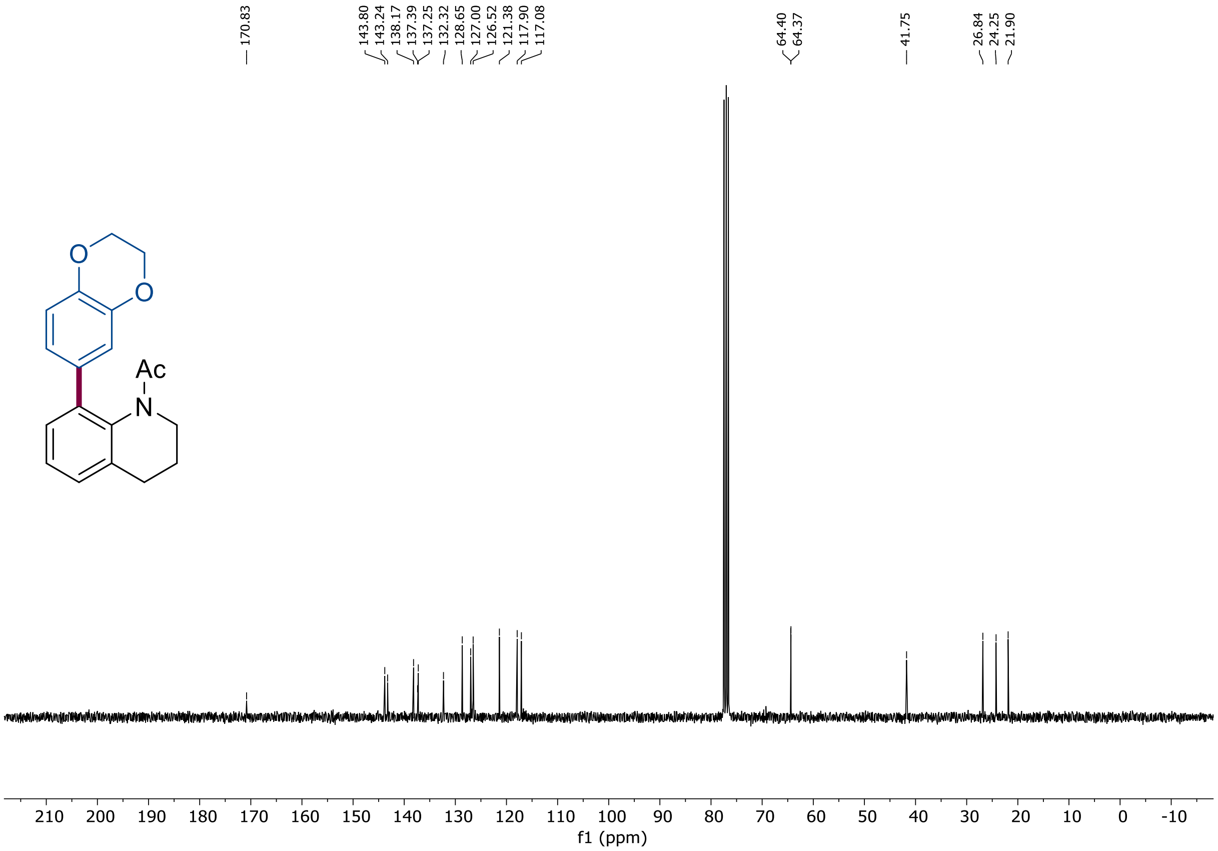

NMR Spectrum 91 ^{13}C NMR for 40, 75 MHz, $CDCl_3$, room temperature.

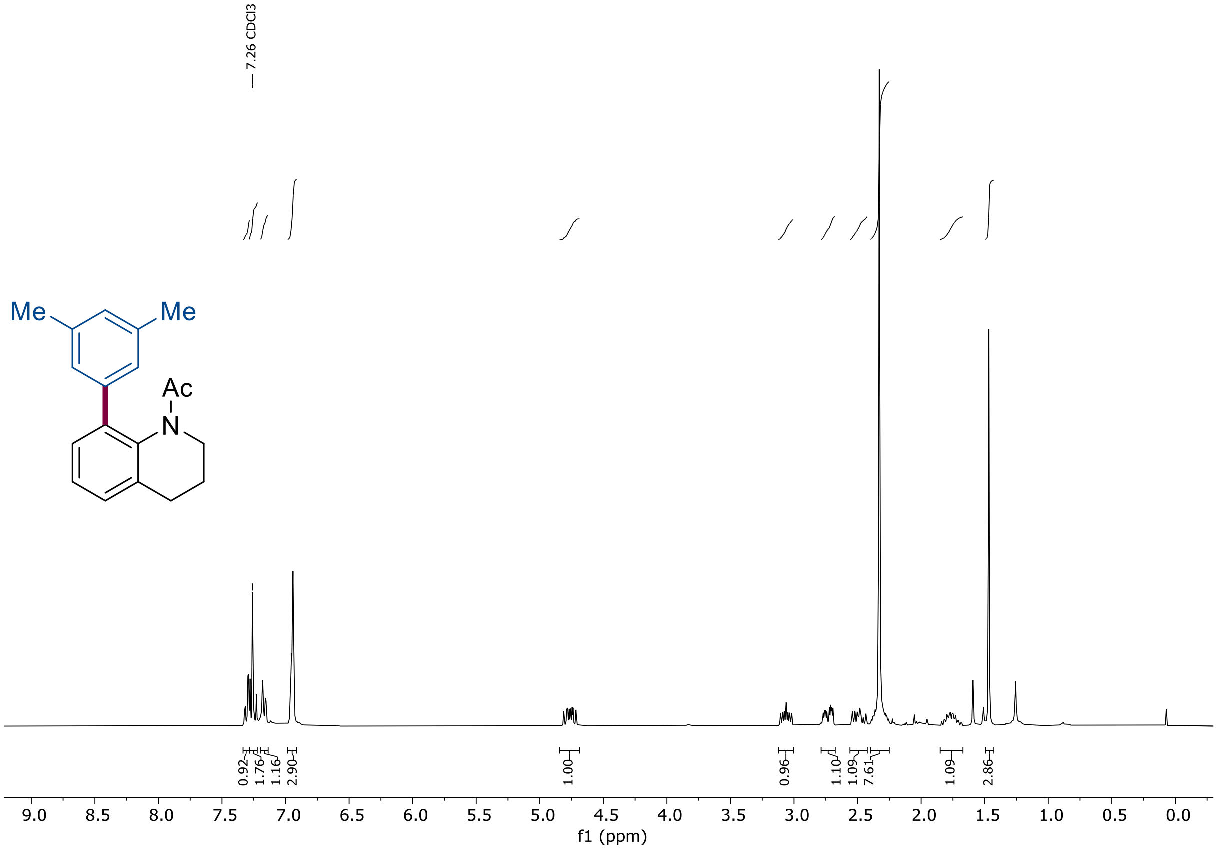

NMR Spectrum 92 ^{1}H NMR for 41, 300 MHz, $CDCl_3$, room temperature.

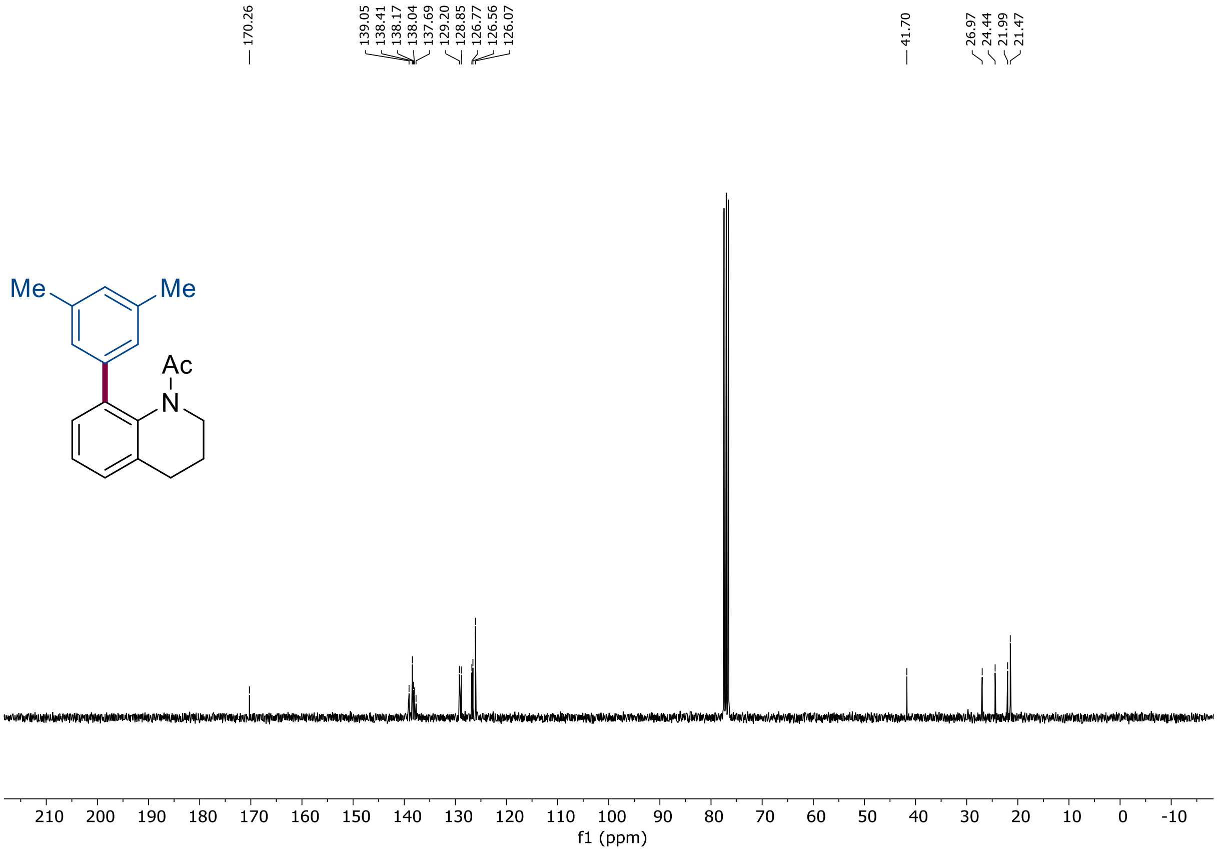

NMR Spectrum 93 ^{13}C NMR for 41, 75 MHz, $CDCl_3$, room temperature.

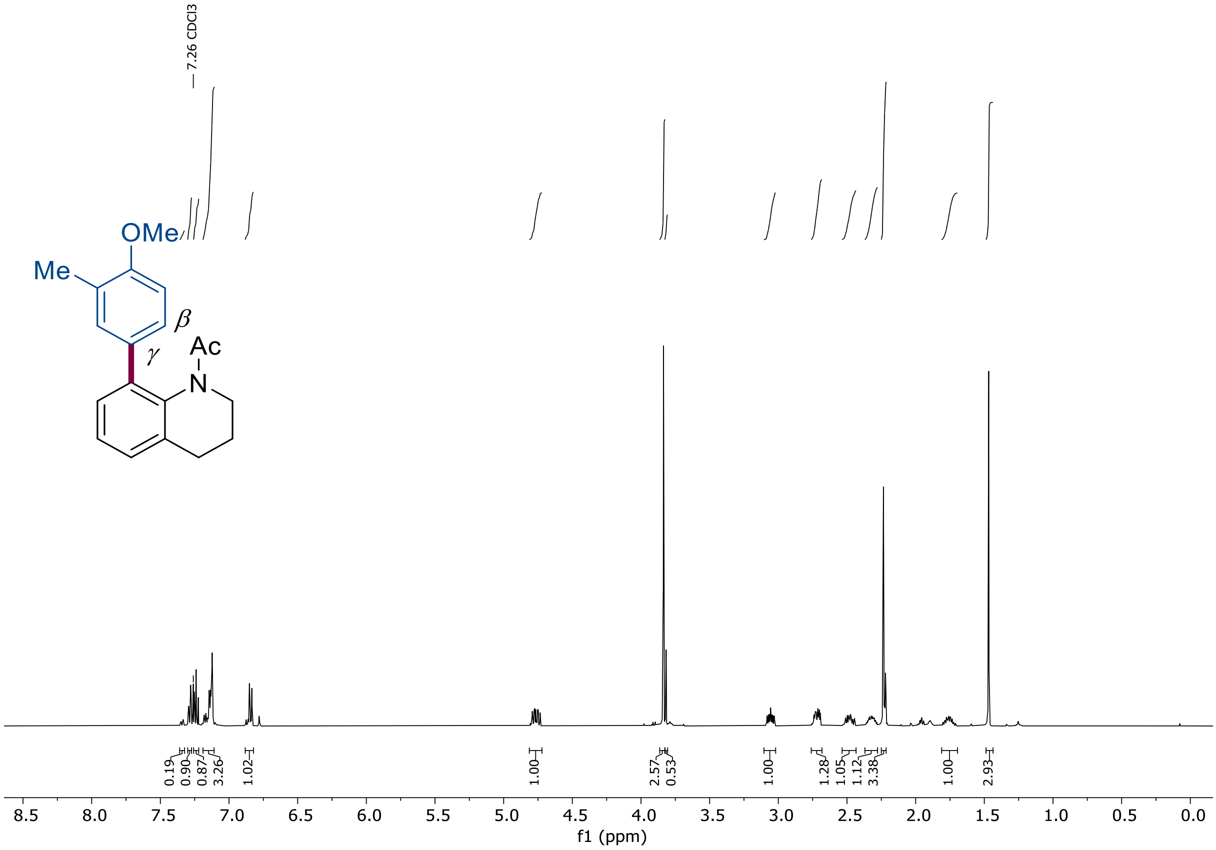

NMR Spectrum 94 ^{1}H NMR for 42, 500 MHz, $CDCl_3$, room temperature.

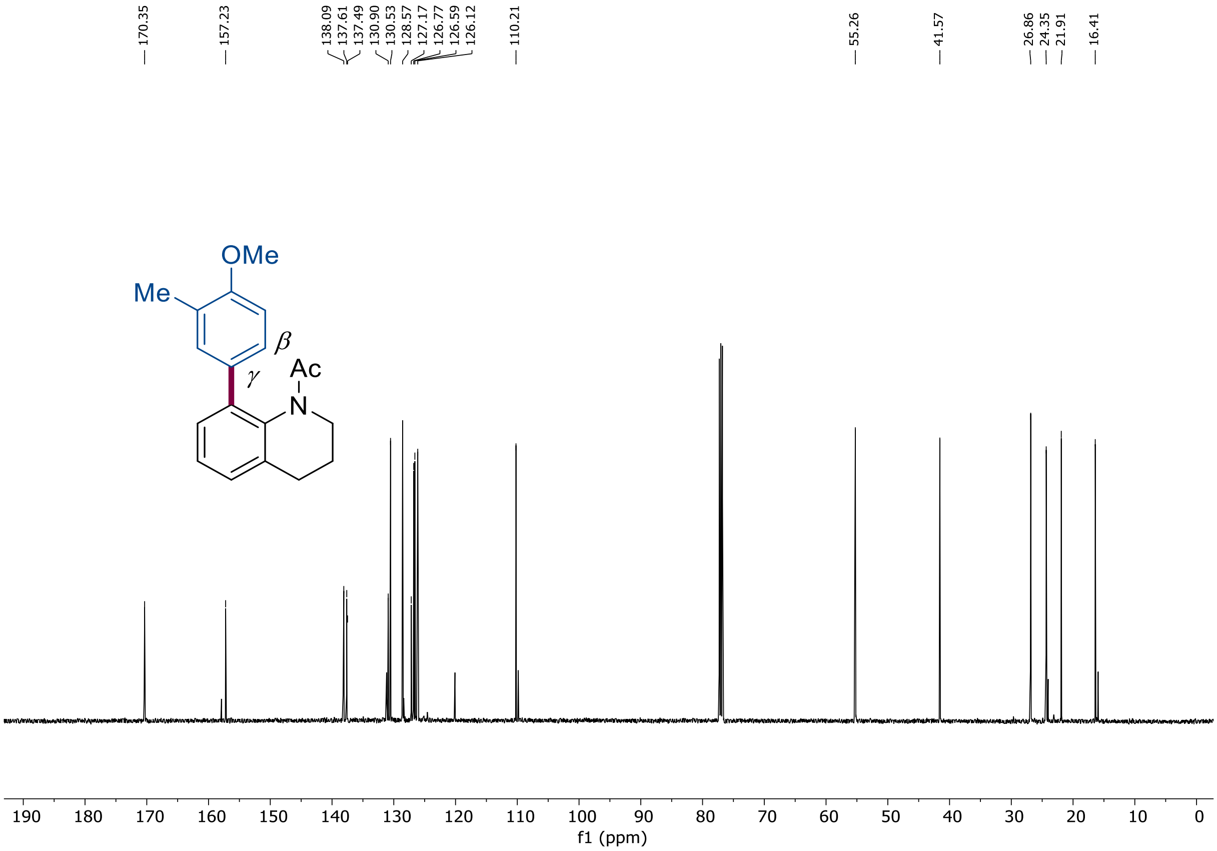

NMR Spectrum 95 ^{13}C NMR for 42, 126 MHz, $CDCl_3$, room temperature.

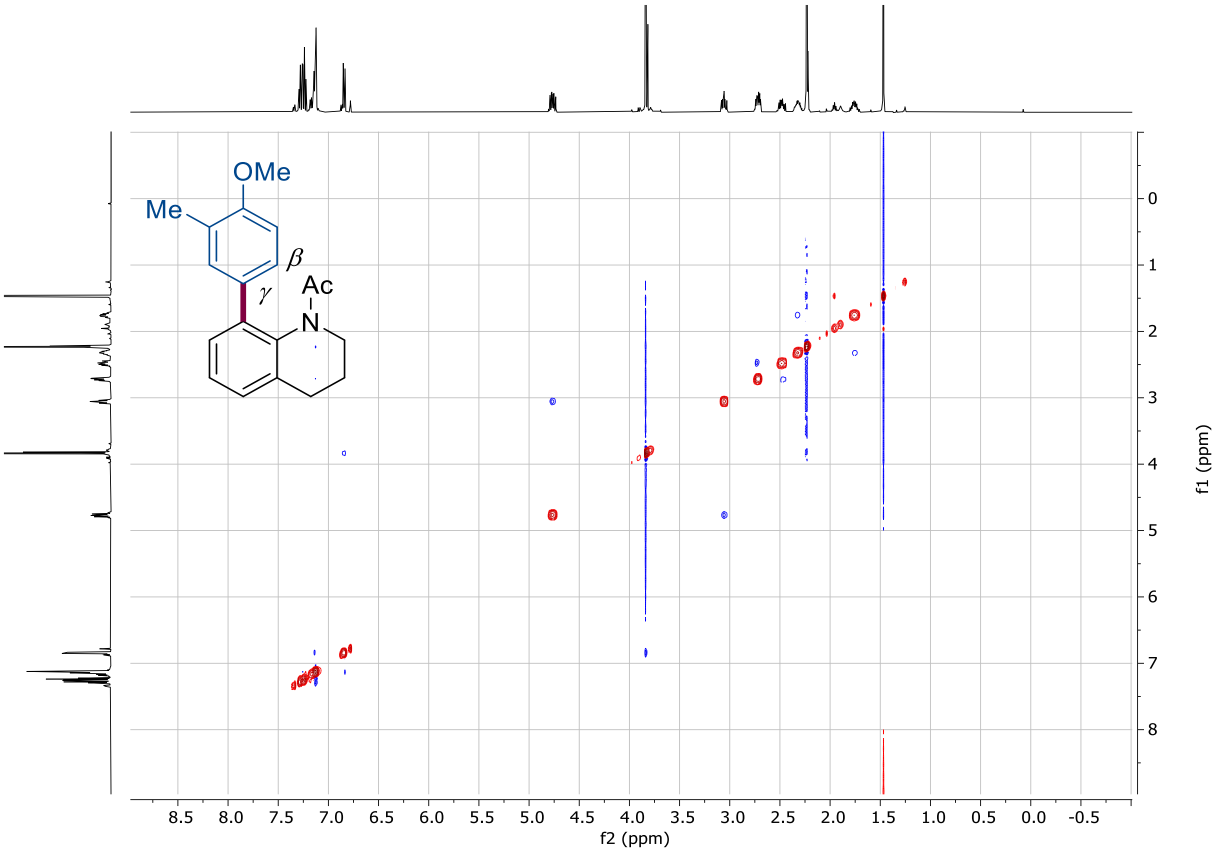

NMR Spectrum 96 NOESY NMR for 42, $CDCl_3$, room temperature.

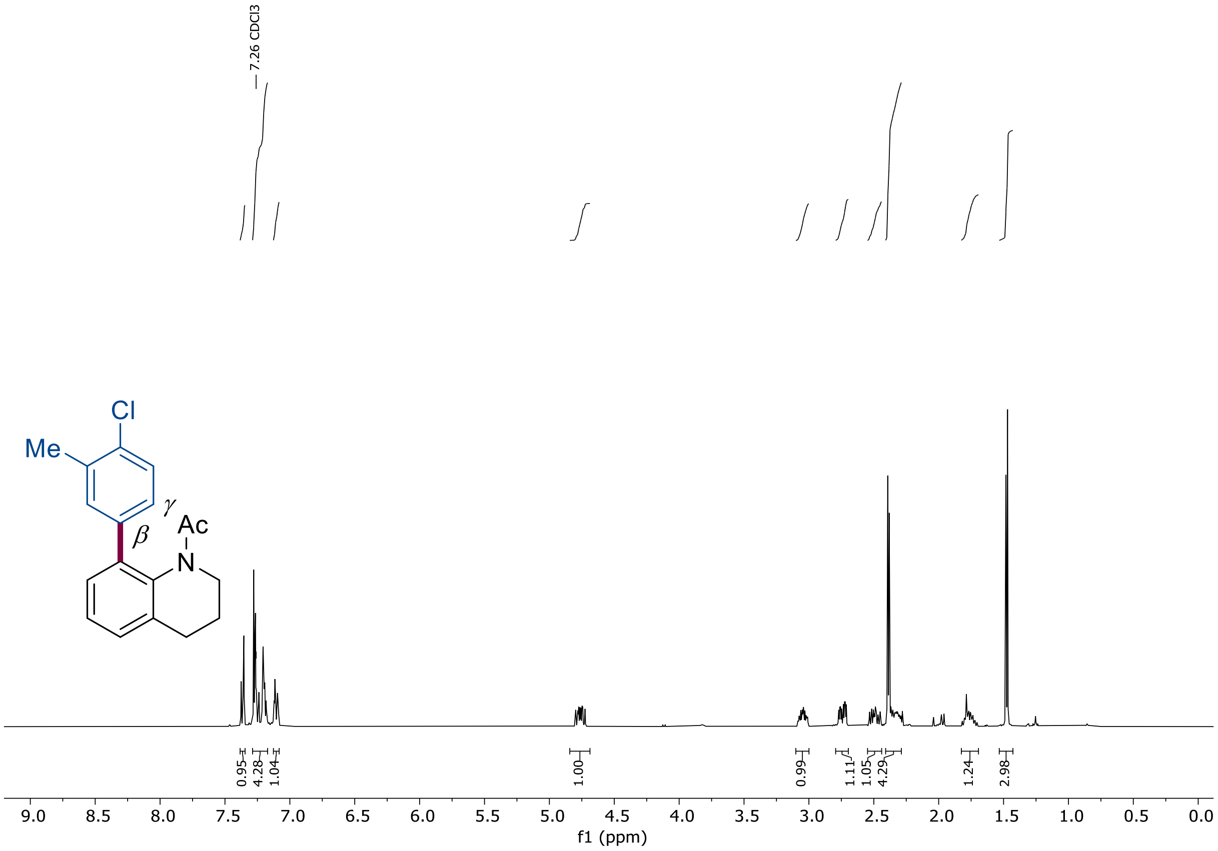

NMR Spectrum 97 ^{1}**H NMR for 43, 400 MHz, CDCl$_3$, room temperature.**

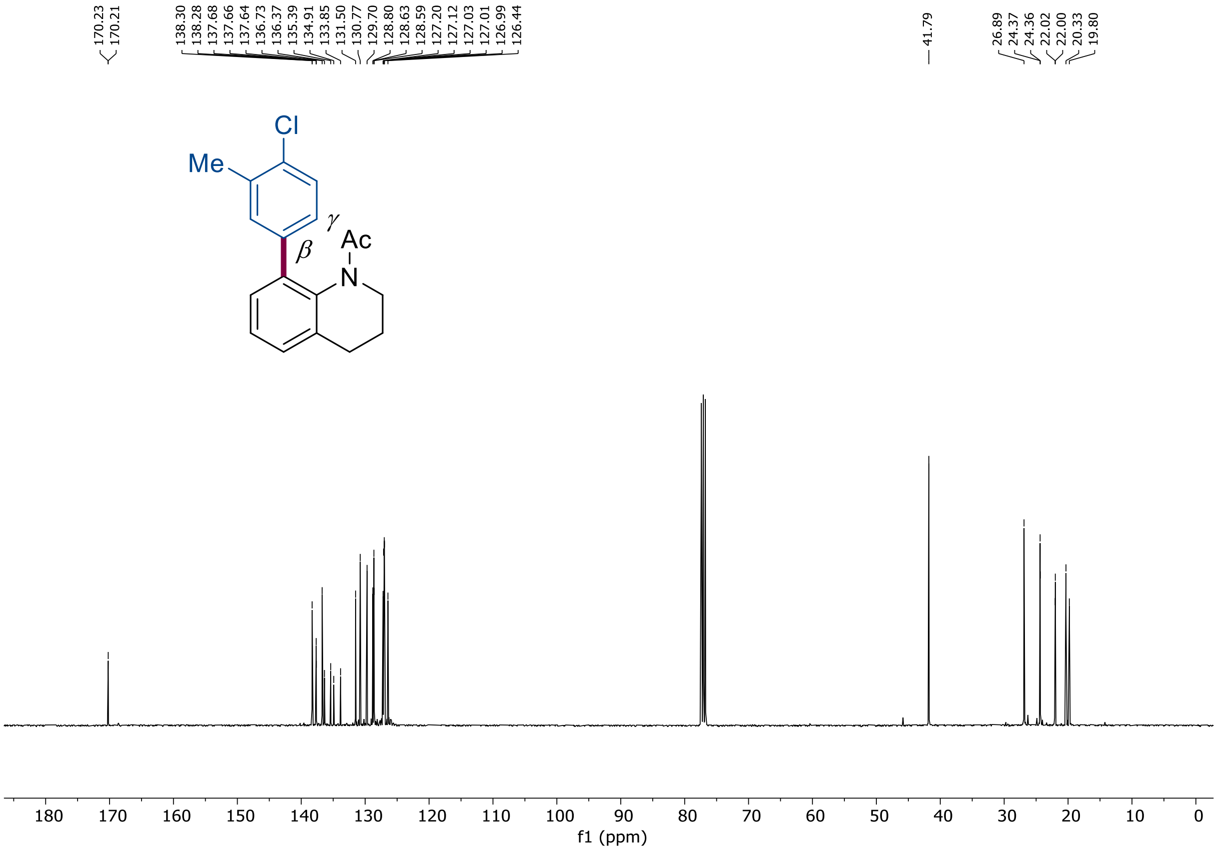

NMR Spectrum 98 ^{13}**C NMR for 43, 101 MHz, CDCl$_3$, room temperature.**

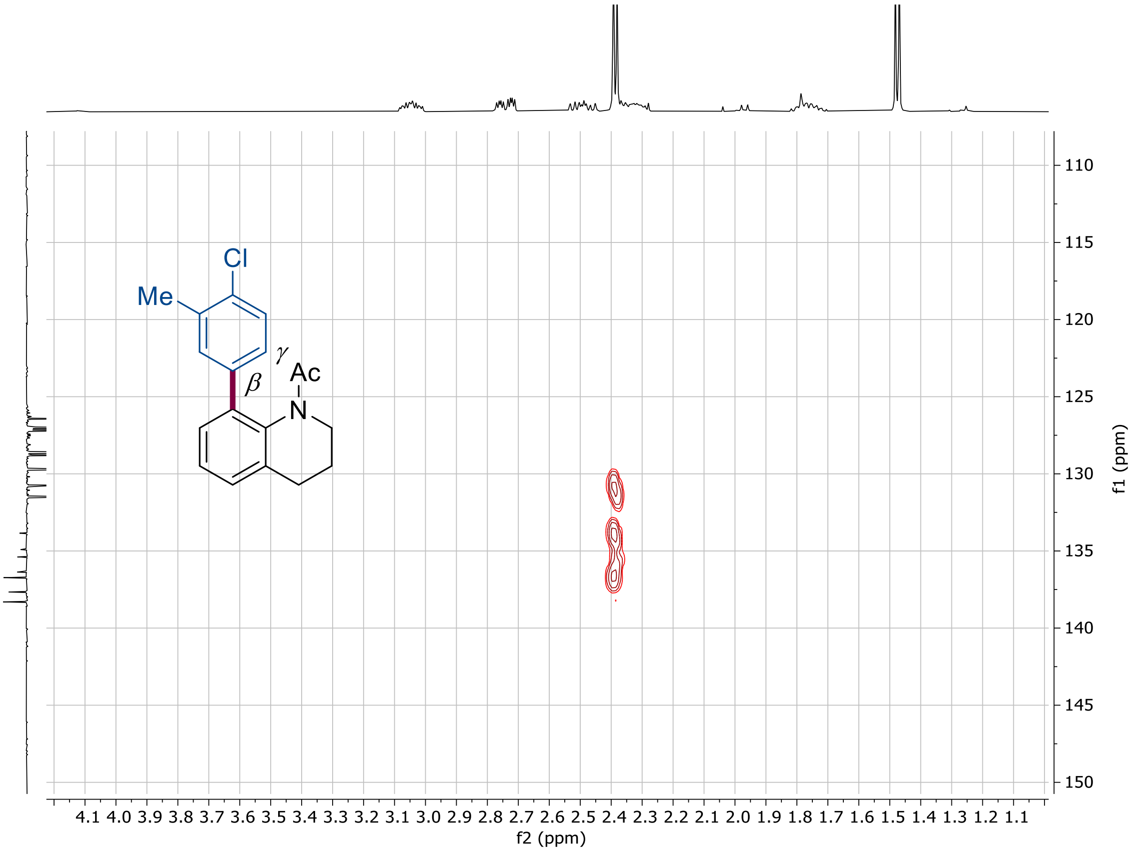

NMR Spectrum 99 HMBC NMR for 43, $CDCl_3$, room temperature.

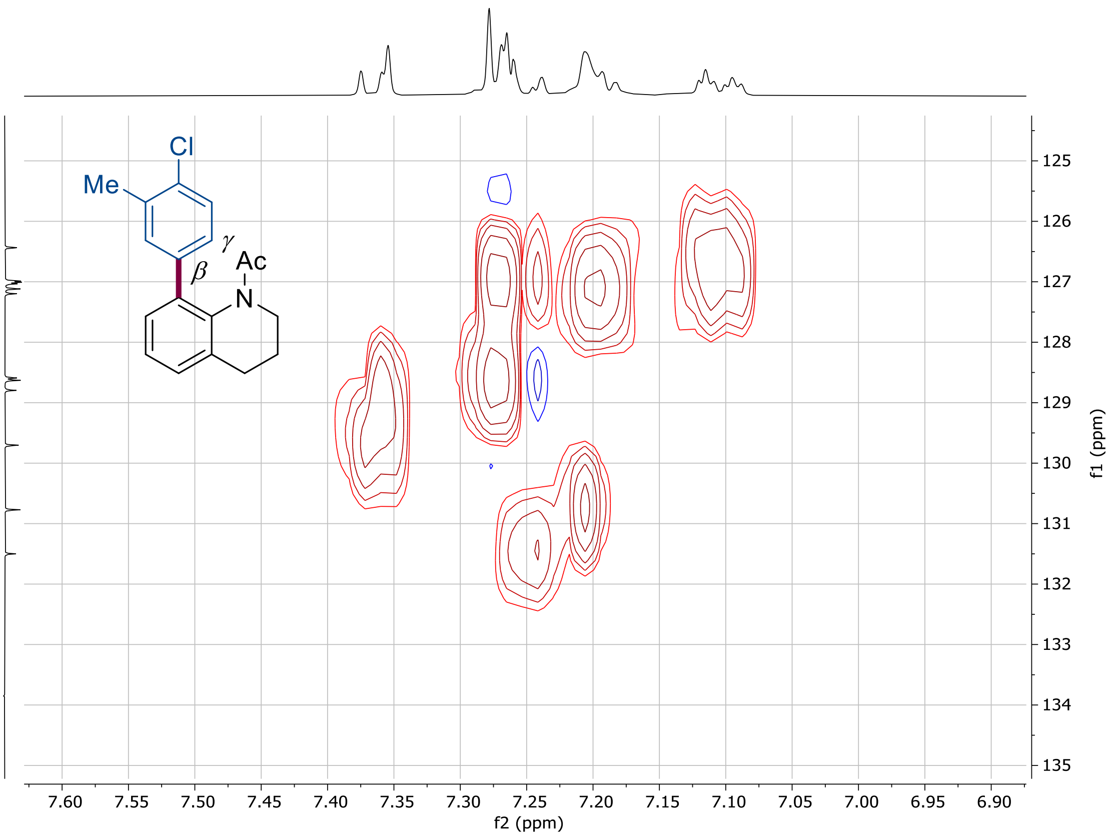

NMR Spectrum 100 HSQC NMR for 43, $CDCl_3$, room temperature.

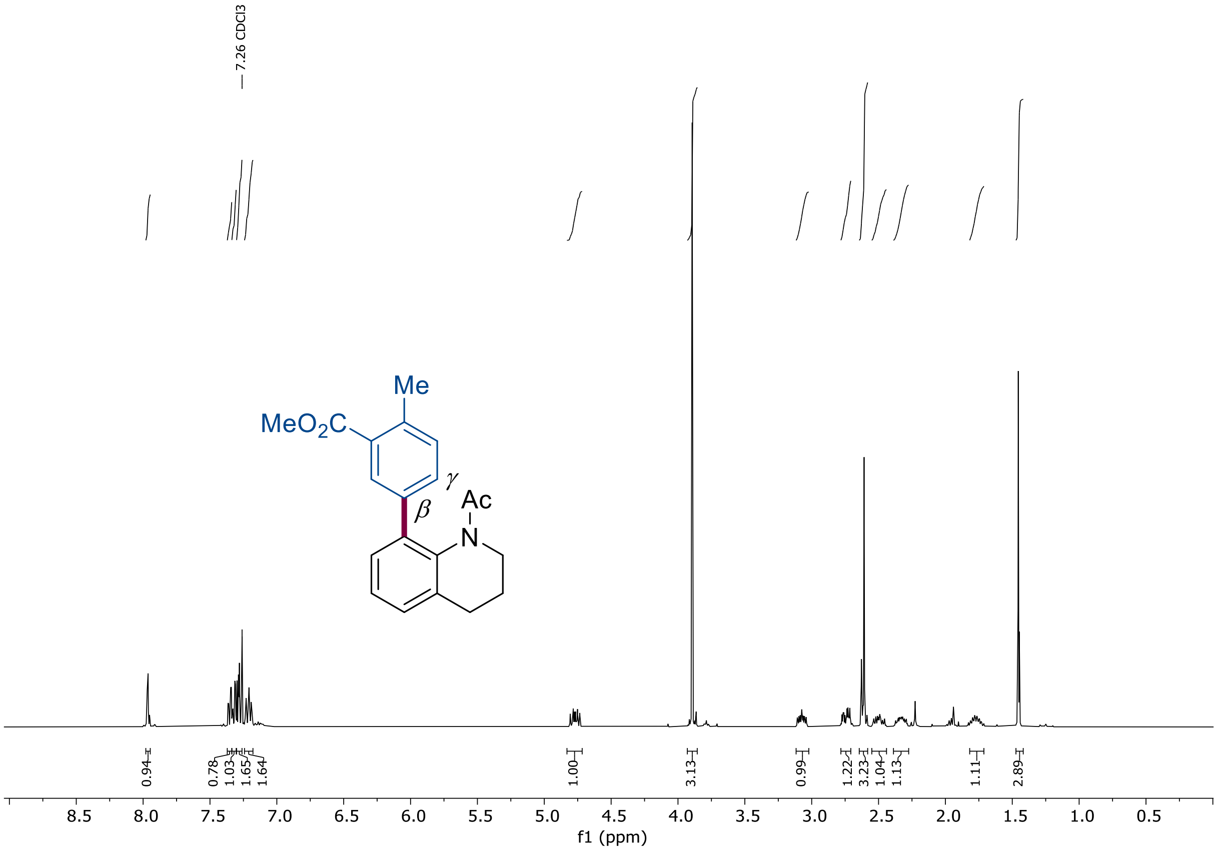

NMR Spectrum 101 ^{1}H NMR for 44, 400 MHz, $CDCl_3$, room temperature.

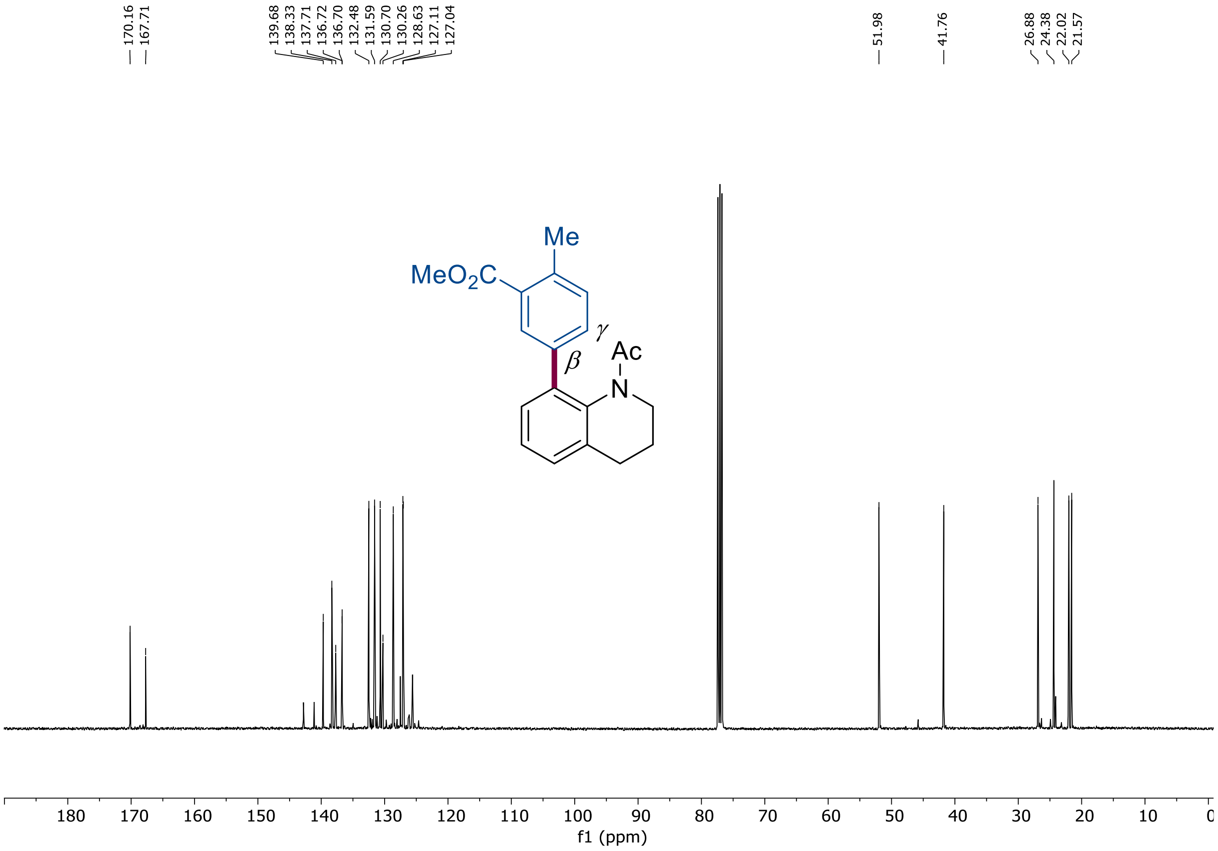

NMR Spectrum 102 ^{13}C NMR for 44, 101 MHz, $CDCl_3$, room temperature.

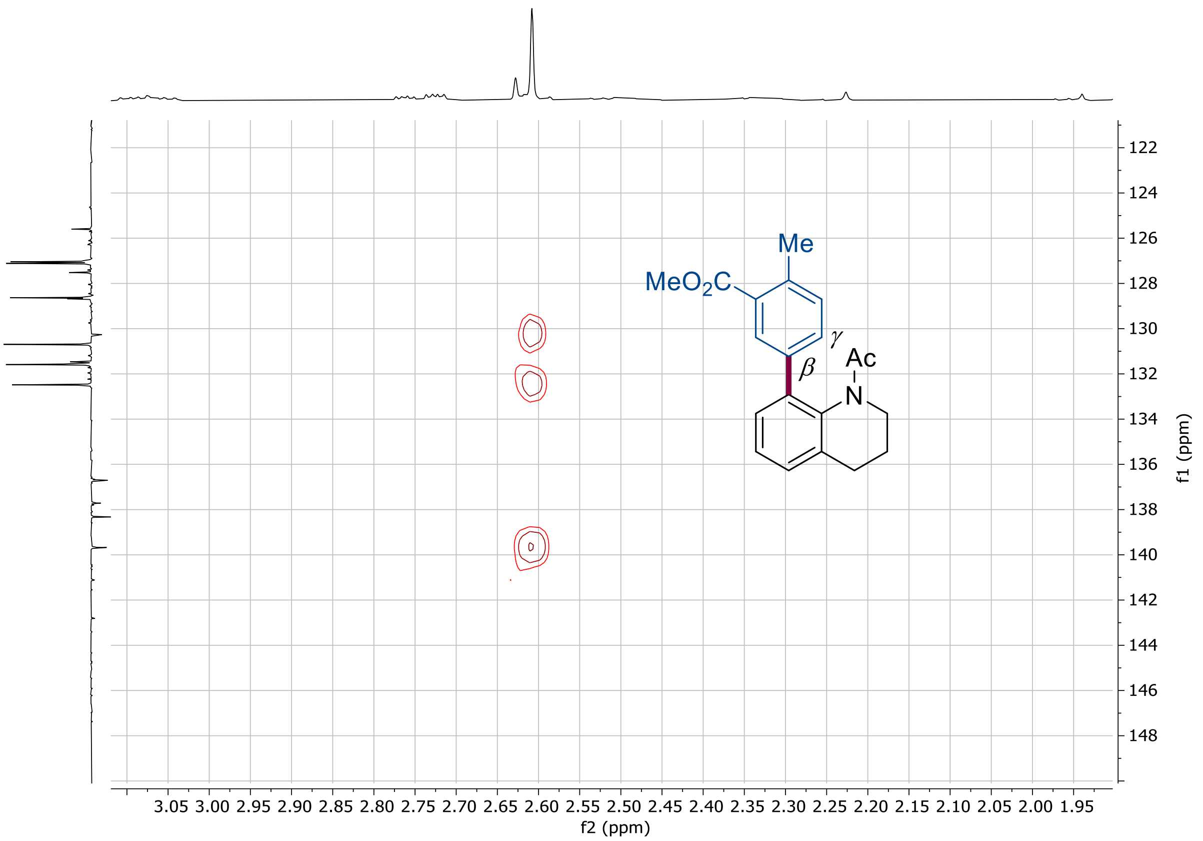

NMR Spectrum 103 HMBC NMR for 44, $CDCl_3$, room temperature.

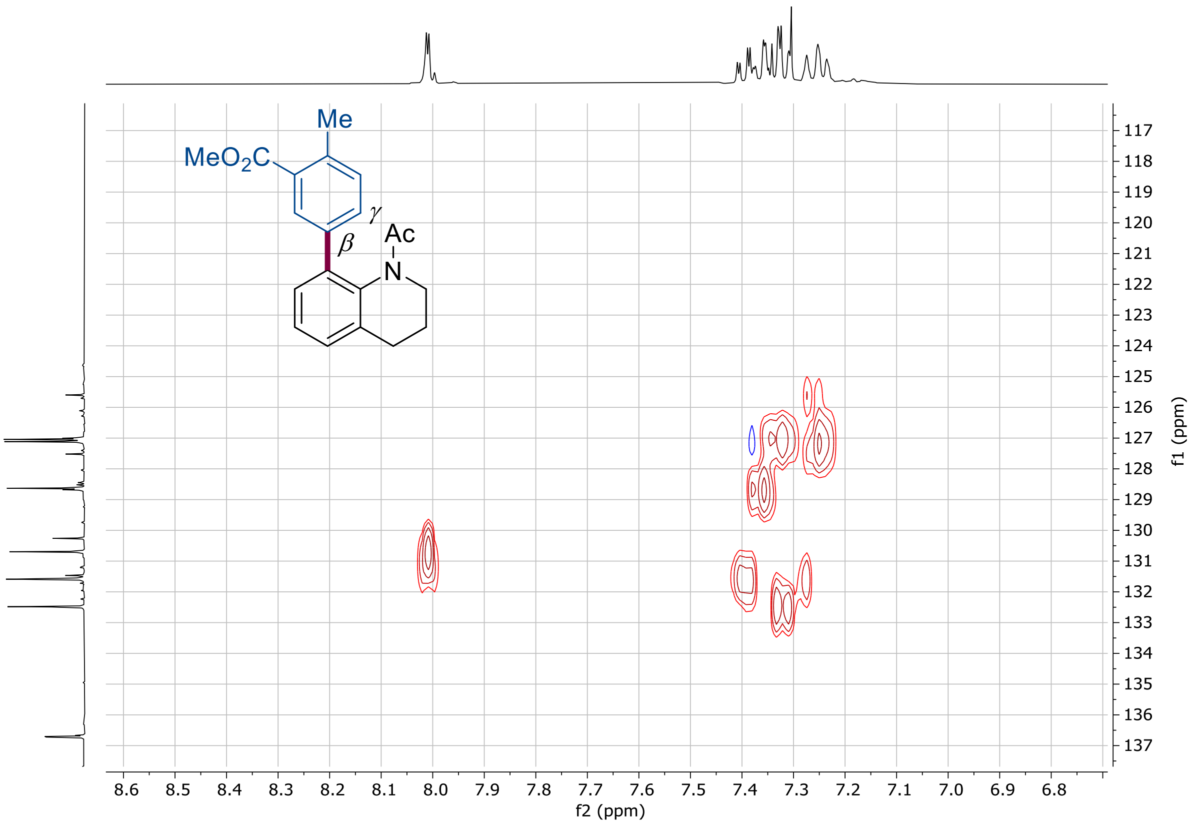

NMR Spectrum 104 HSQC NMR for 44, $CDCl_3$, room temperature.

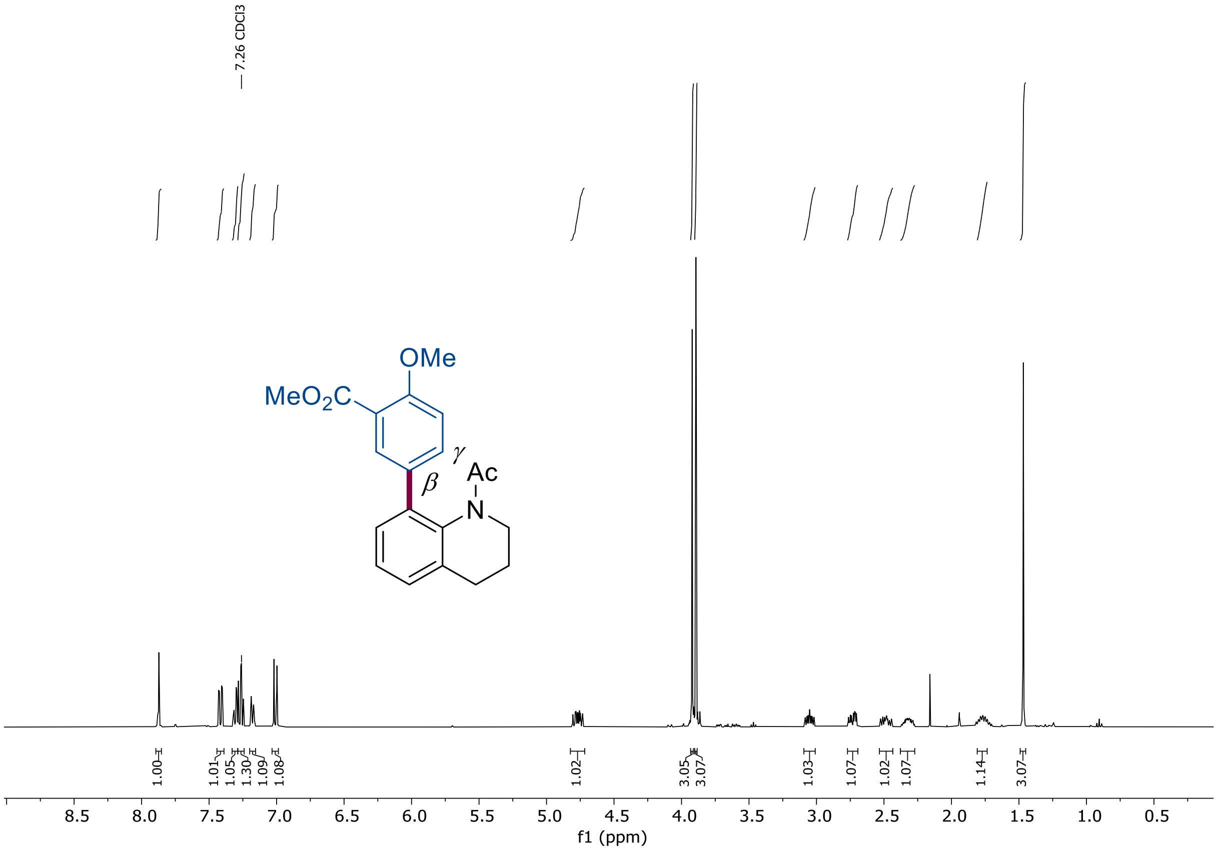

NMR Spectrum 105 ¹H NMR for 45, 400 MHz, CDCl₃, room temperature.

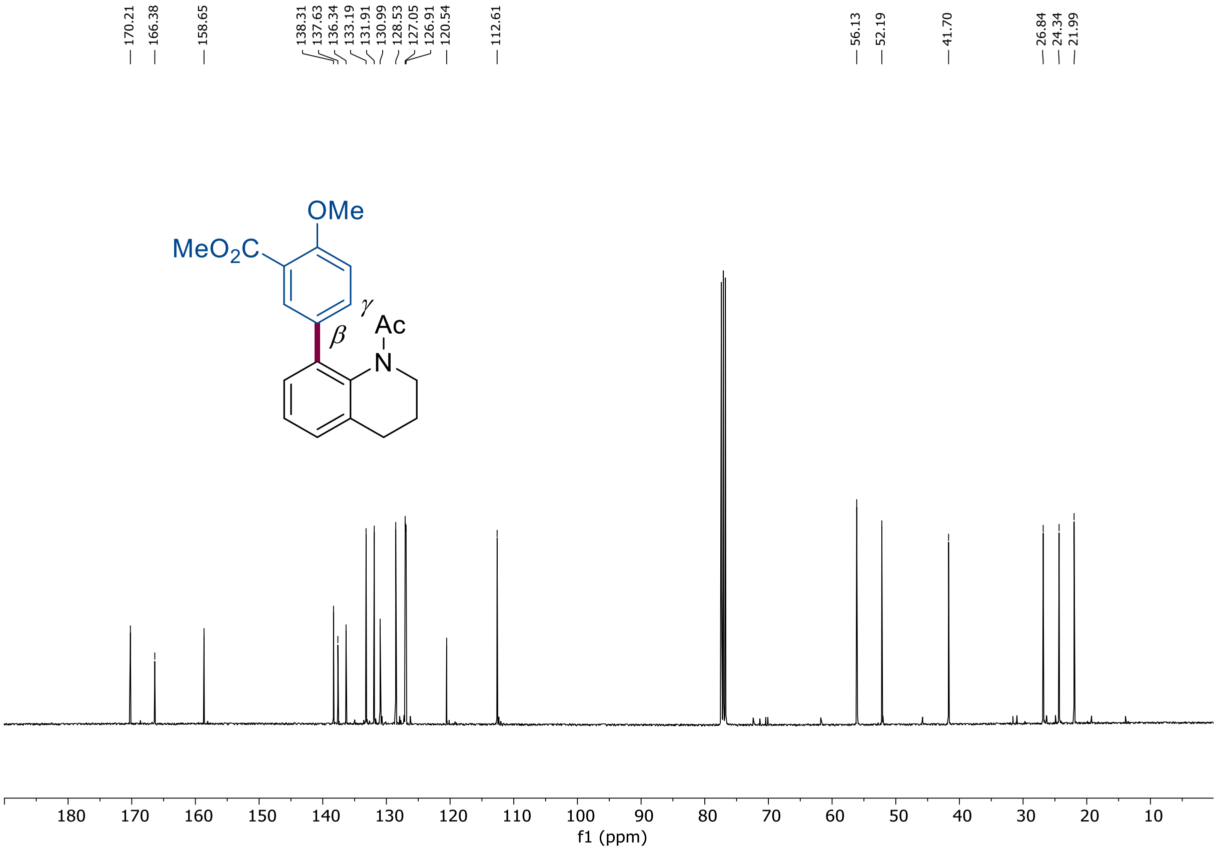

NMR Spectrum 106 ¹³C NMR for 45, 101 MHz, CDCl₃, room temperature.

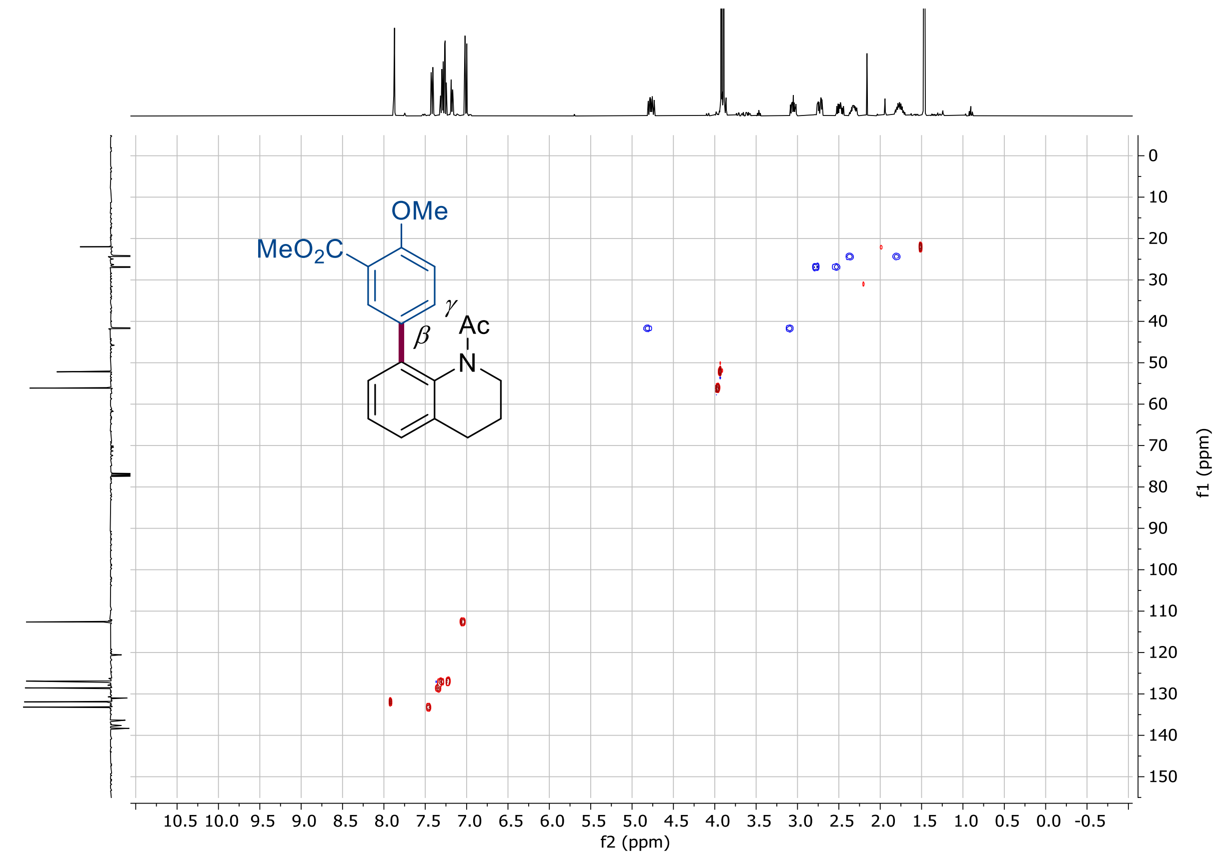

NMR Spectrum 107 HSQC NMR for 45, $CDCl_3$, room temperature.

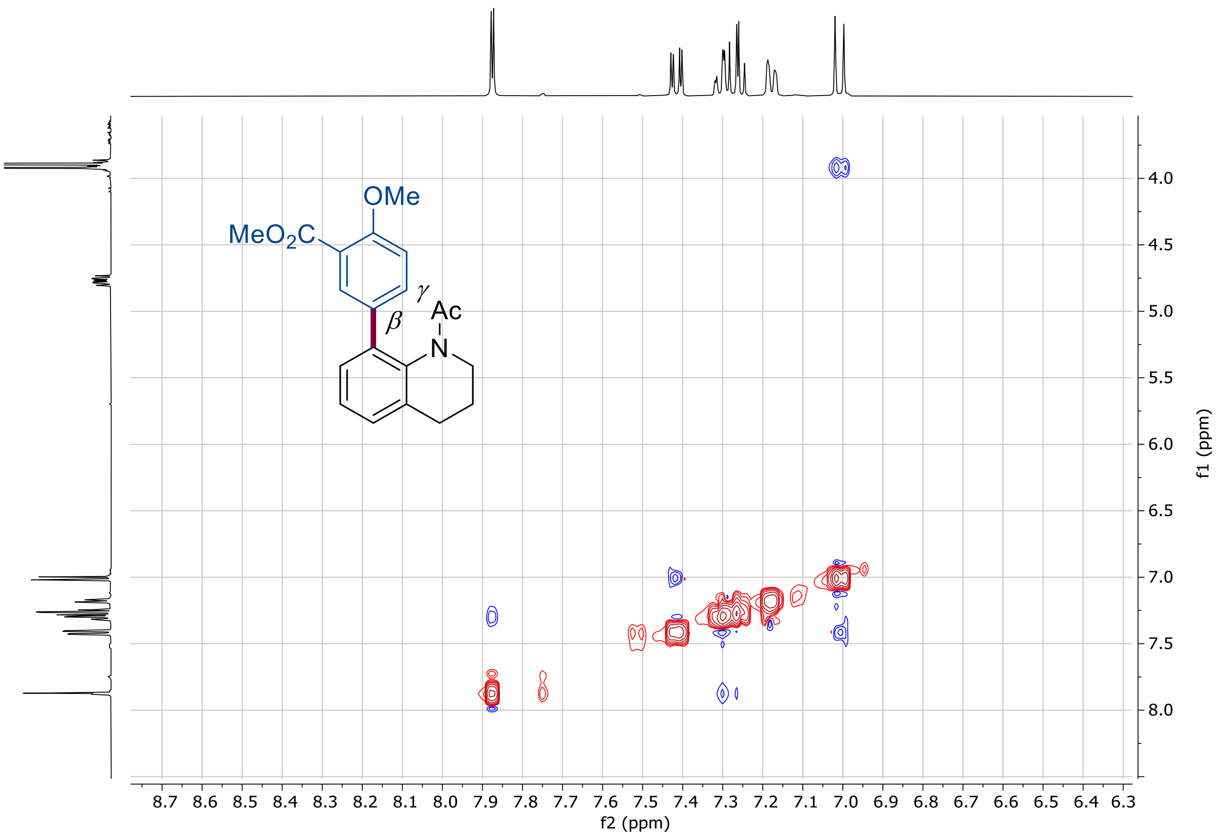

NMR Spectrum 108 NOESY NMR for 45, $CDCl_3$, room temperature.

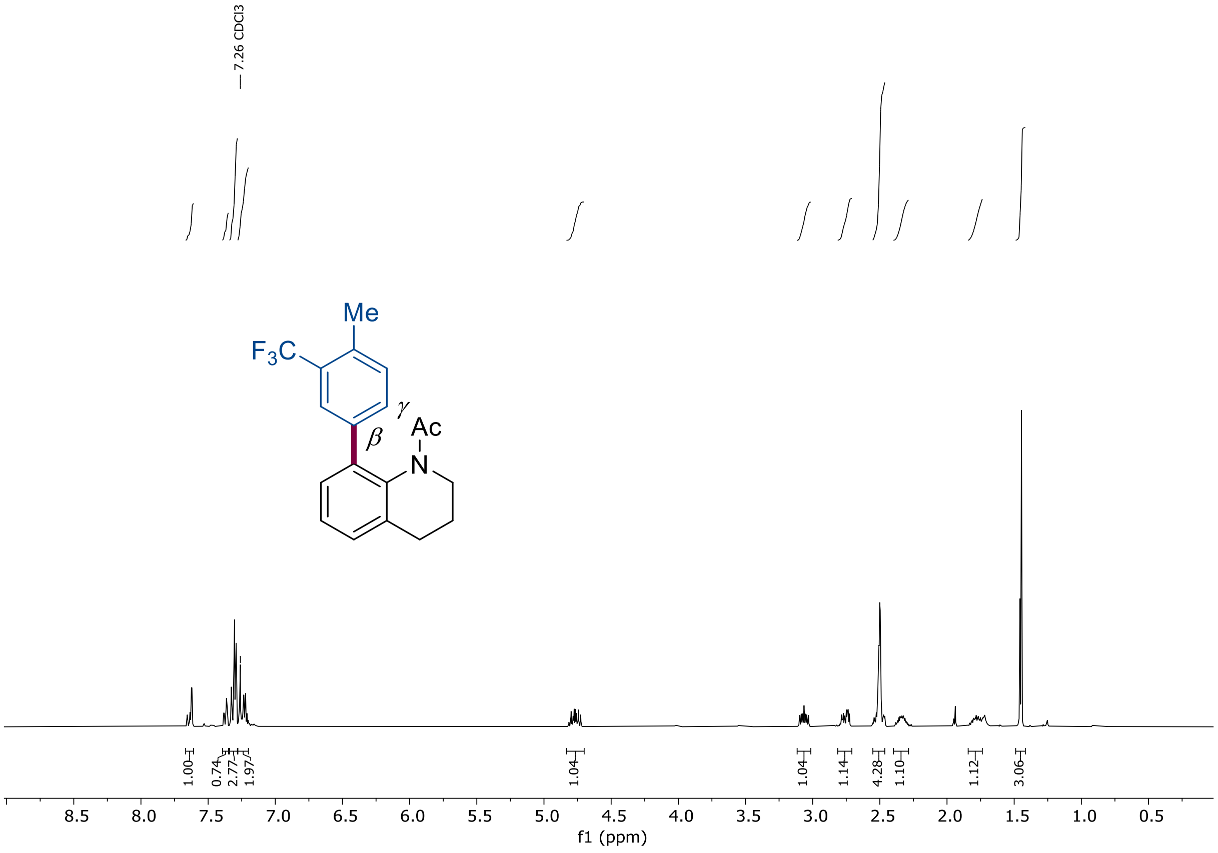

NMR Spectrum 109 ^{1}H NMR for 46, 400 MHz, CDCl$_3$, room temperature.

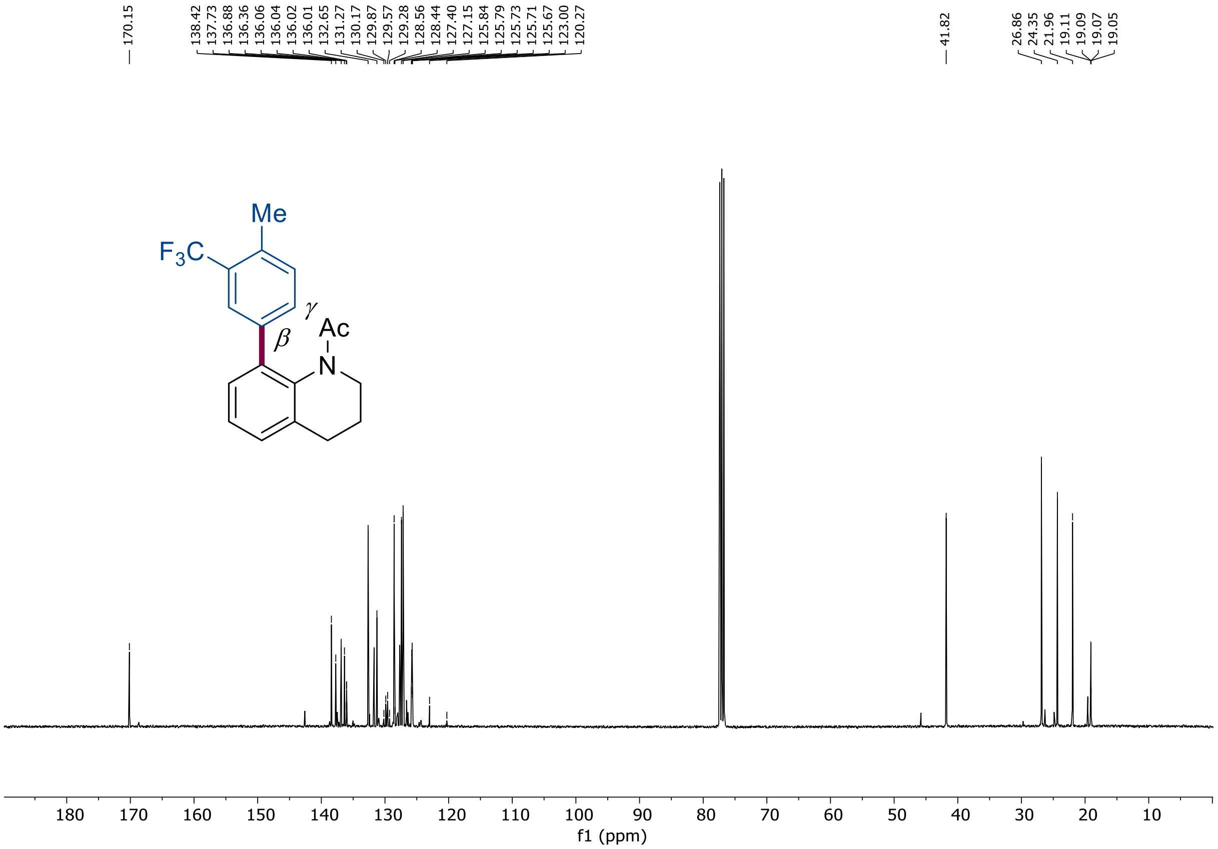

NMR Spectrum 110 ^{13}C NMR for 46, 101 MHz, CDCl$_3$, room temperature.

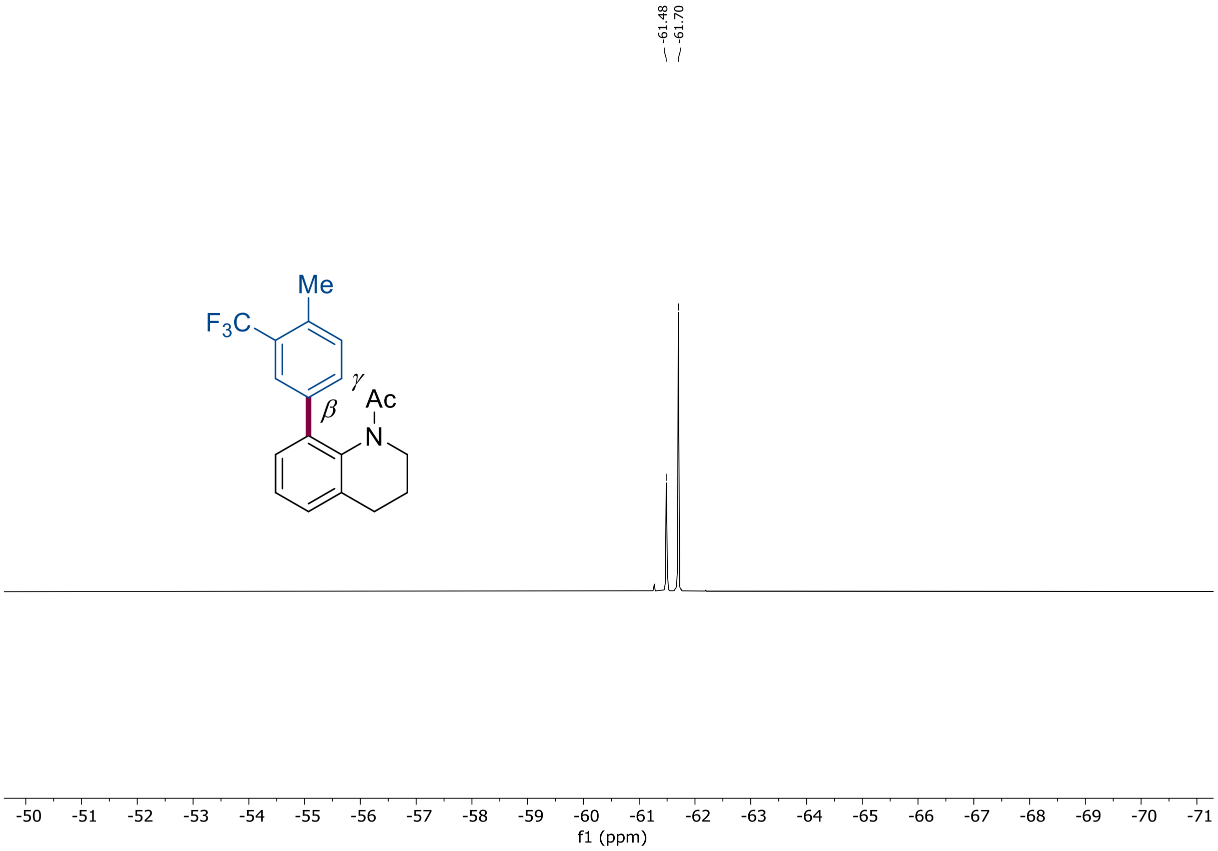

NMR Spectrum 111 ^{19}F NMR for 46, 377 MHz, $CDCl_3$, room temperature.

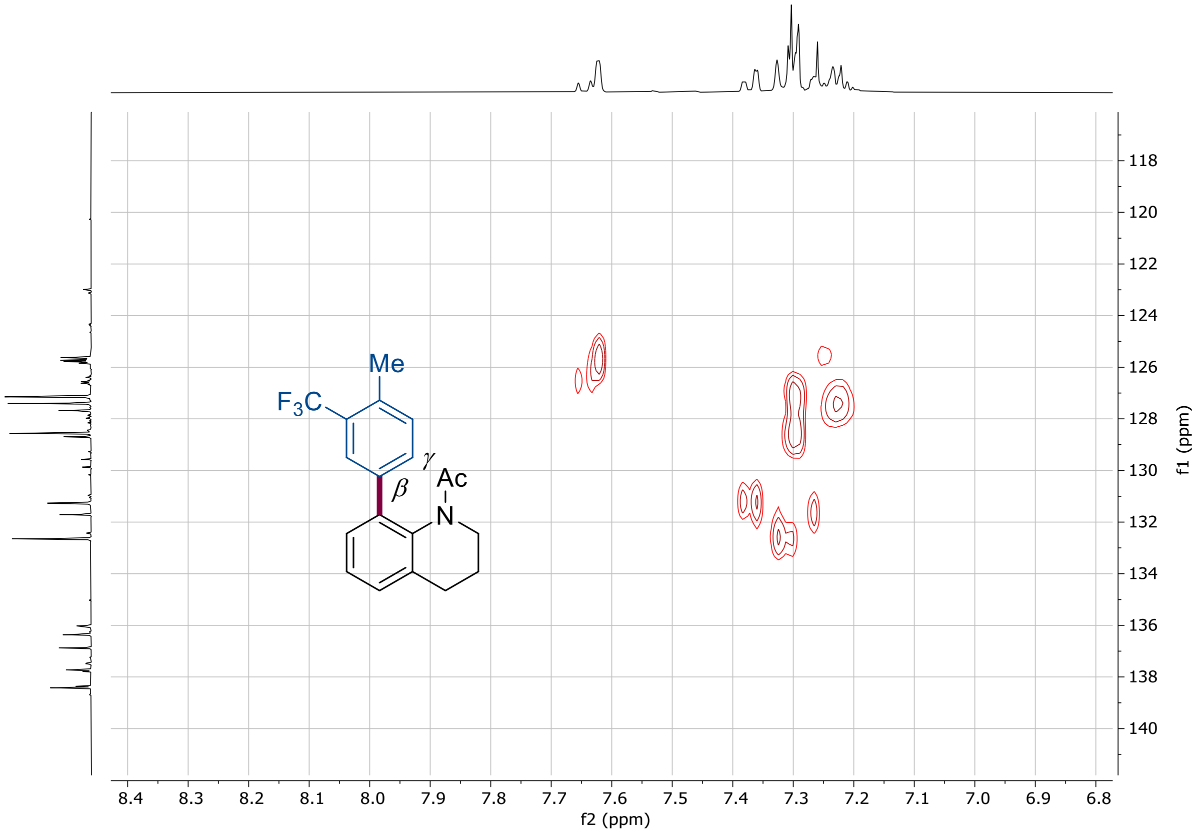

NMR Spectrum 112 HSQC NMR for 46, $CDCl_3$, room temperature.

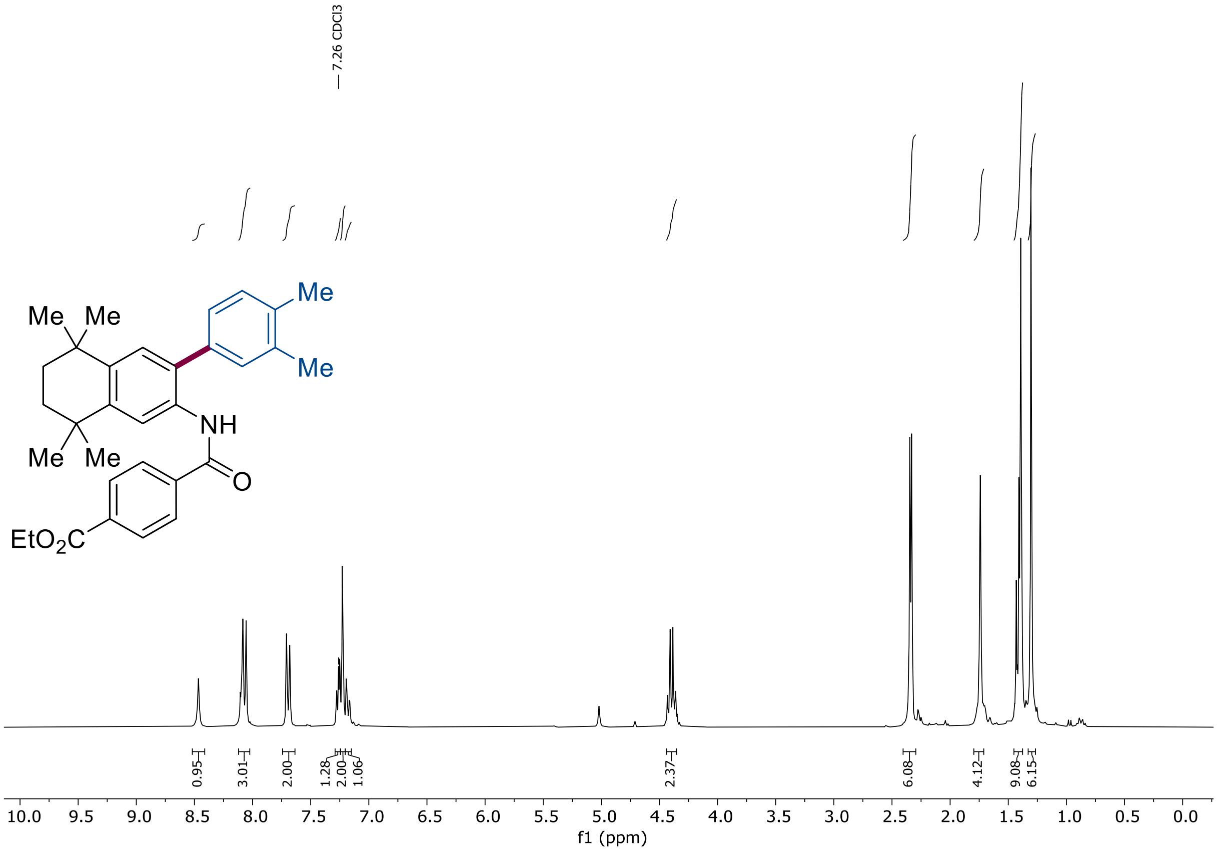

NMR Spectrum 113 ^{1}H NMR for 47, 300 MHz, $CDCl_3$, room temperature.

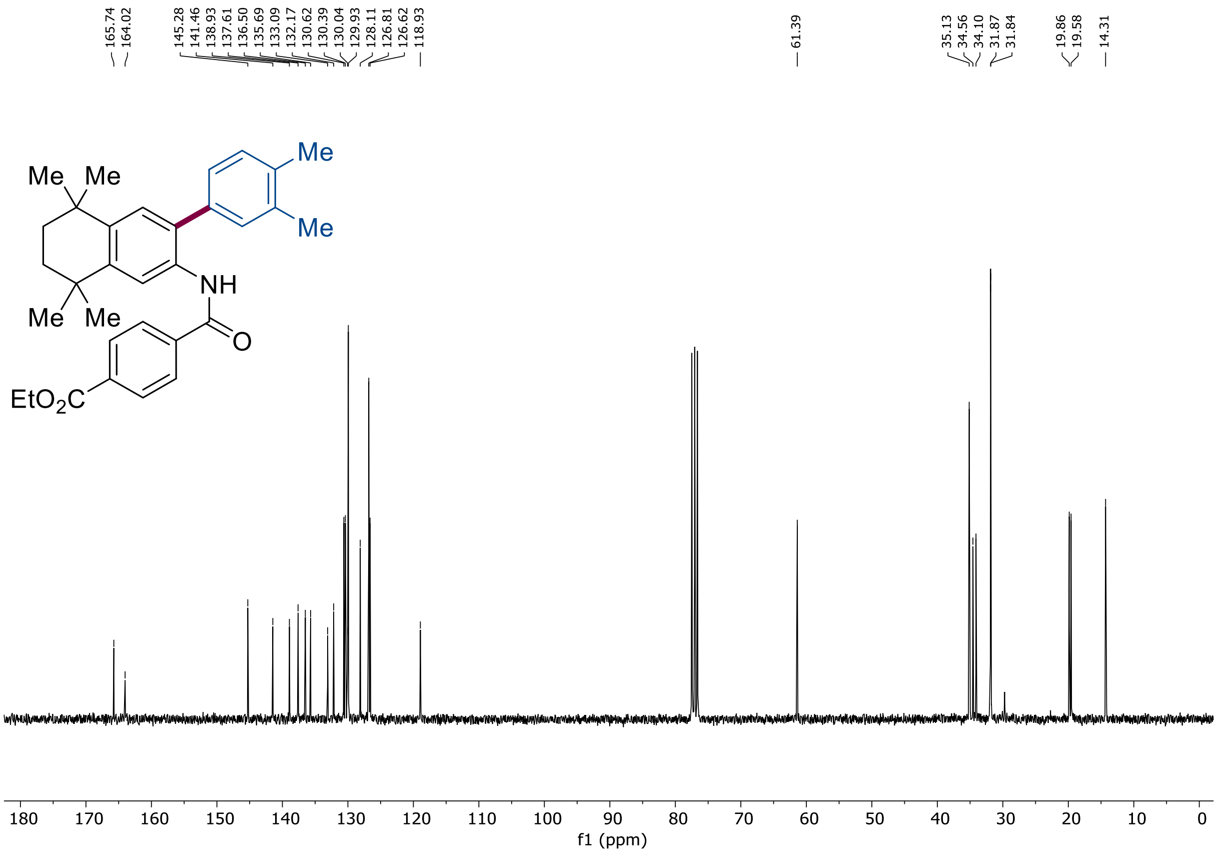

NMR Spectrum 114 ^{13}C NMR for 47, 75 MHz, $CDCl_3$, room temperature.

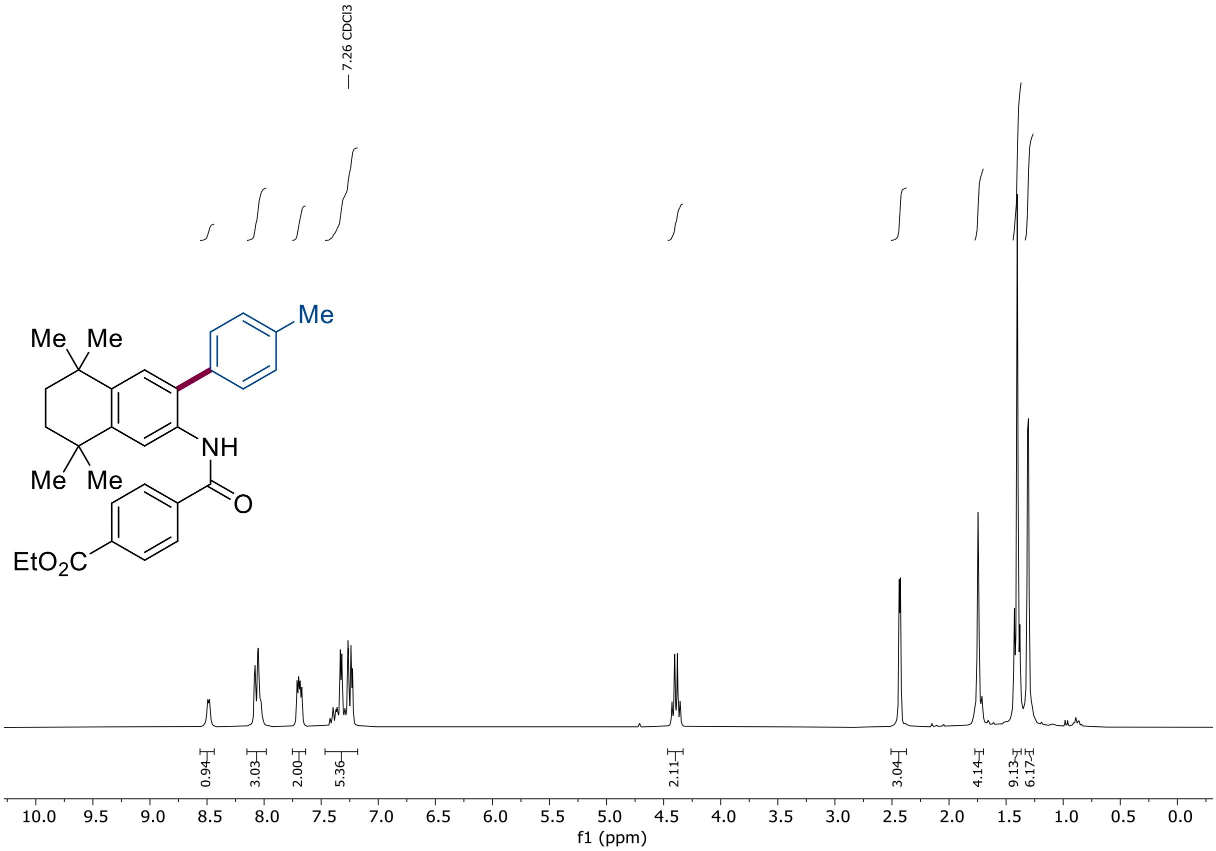

NMR Spectrum 115 ^{1}H NMR for 48, 300 MHz, $CDCl_3$, room temperature.

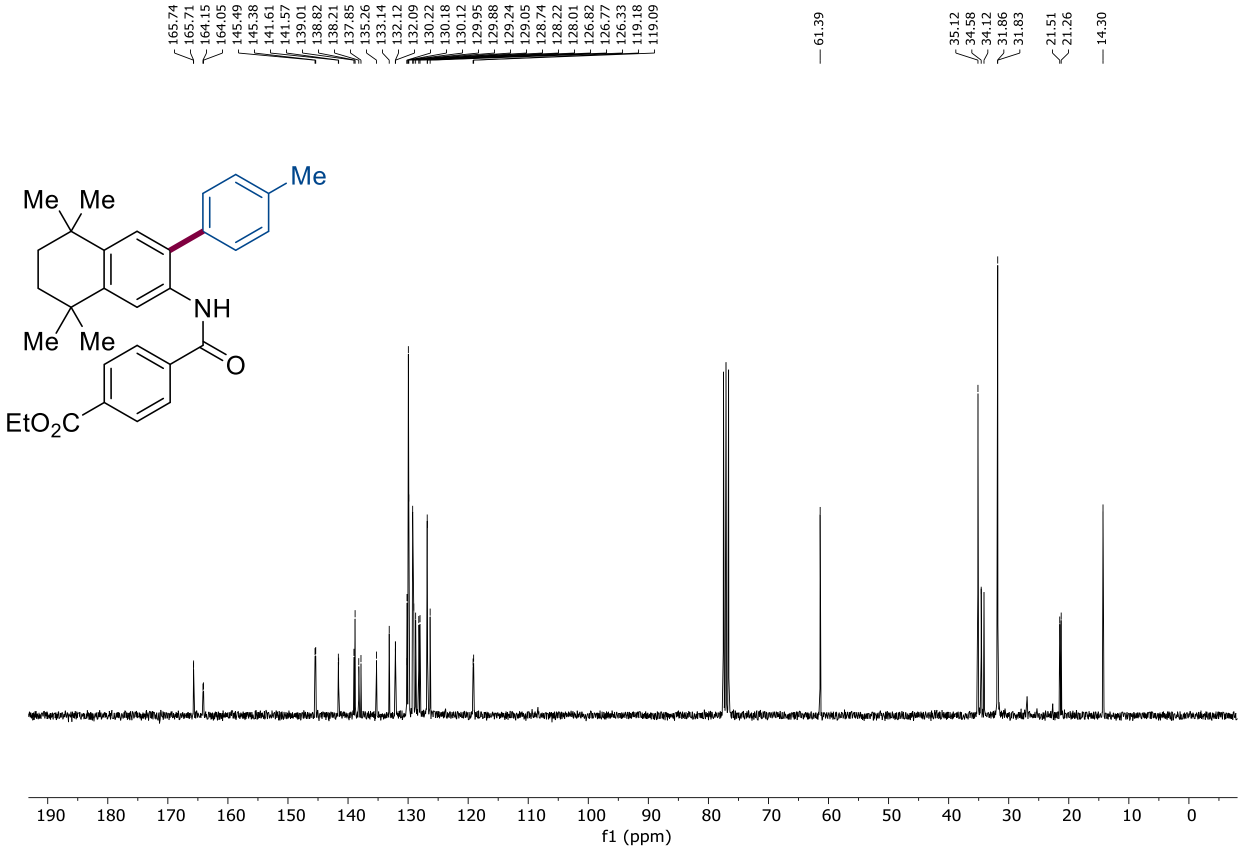

NMR Spectrum 116 ^{13}C NMR for 48, 75 MHz, $CDCl_3$, room temperature.

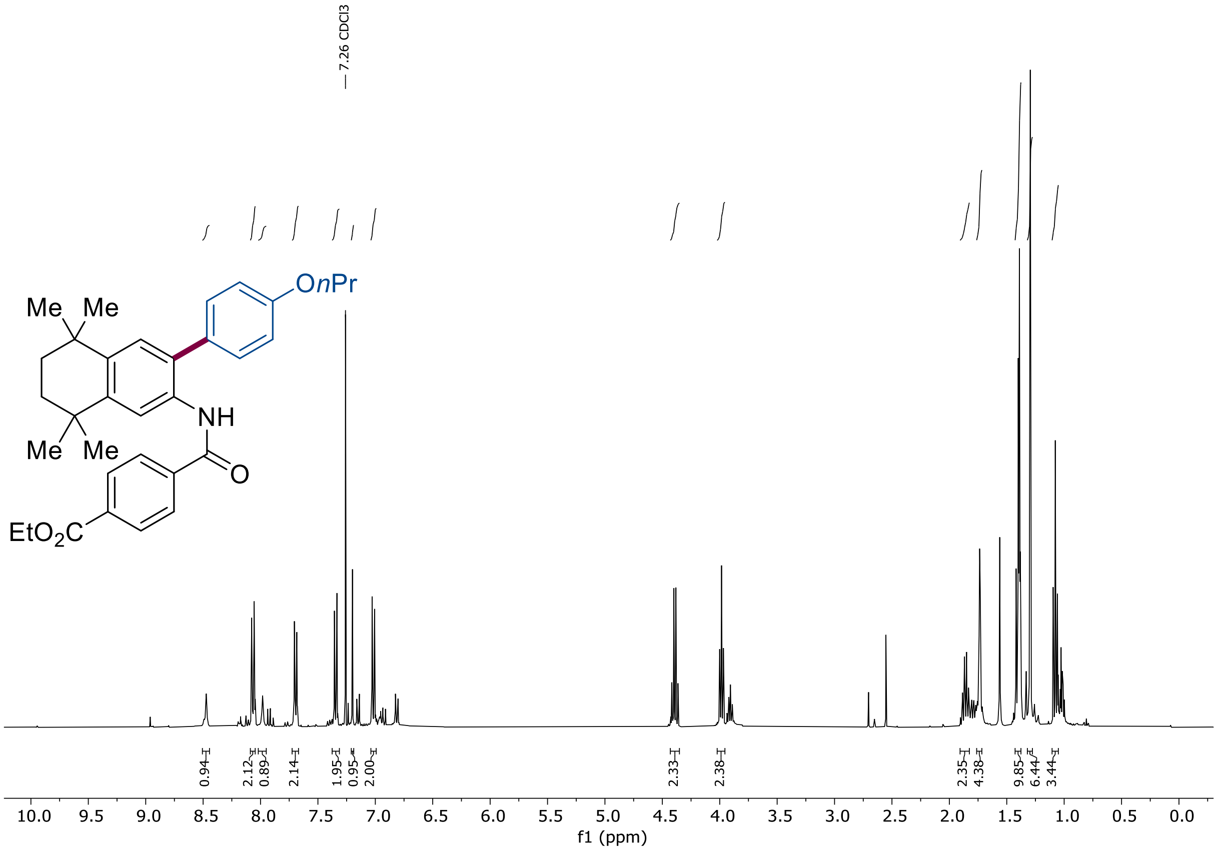

NMR Spectrum 117 ^{1}H NMR for 49, 400 MHz, CDCl$_3$, room temperature.

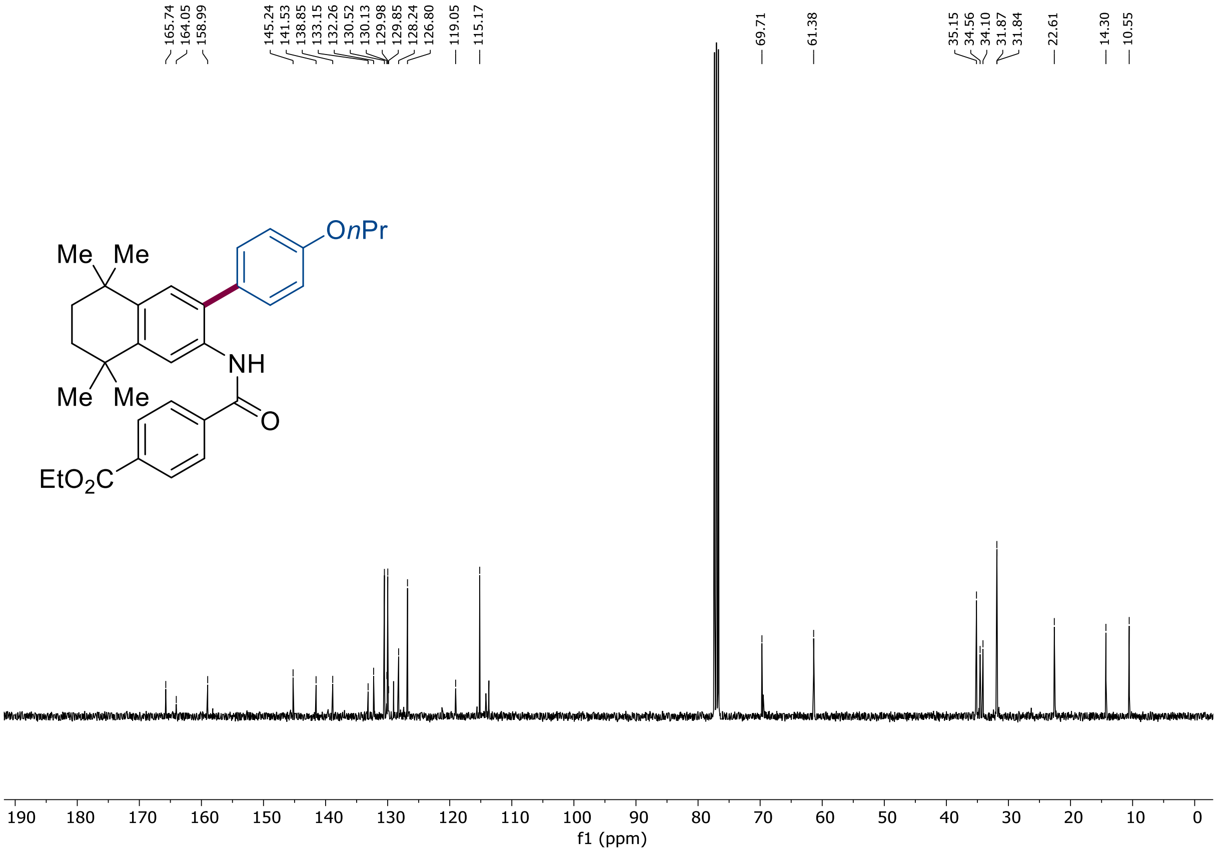

NMR Spectrum 118 ^{13}C NMR for 49, 101 MHz, CDCl$_3$, room temperature.

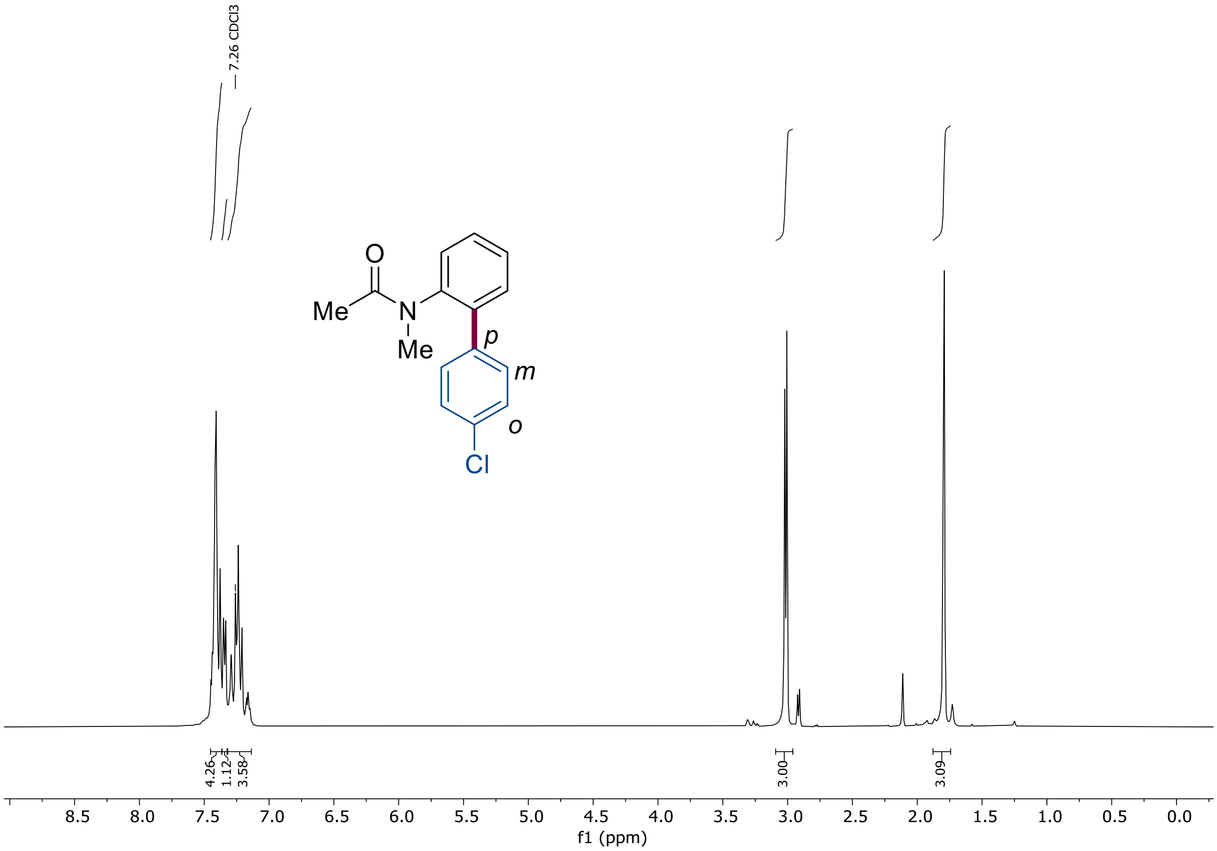

NMR Spectrum 119 ^{1}H NMR for 50, 300 MHz, $CDCl_3$, room temperature.

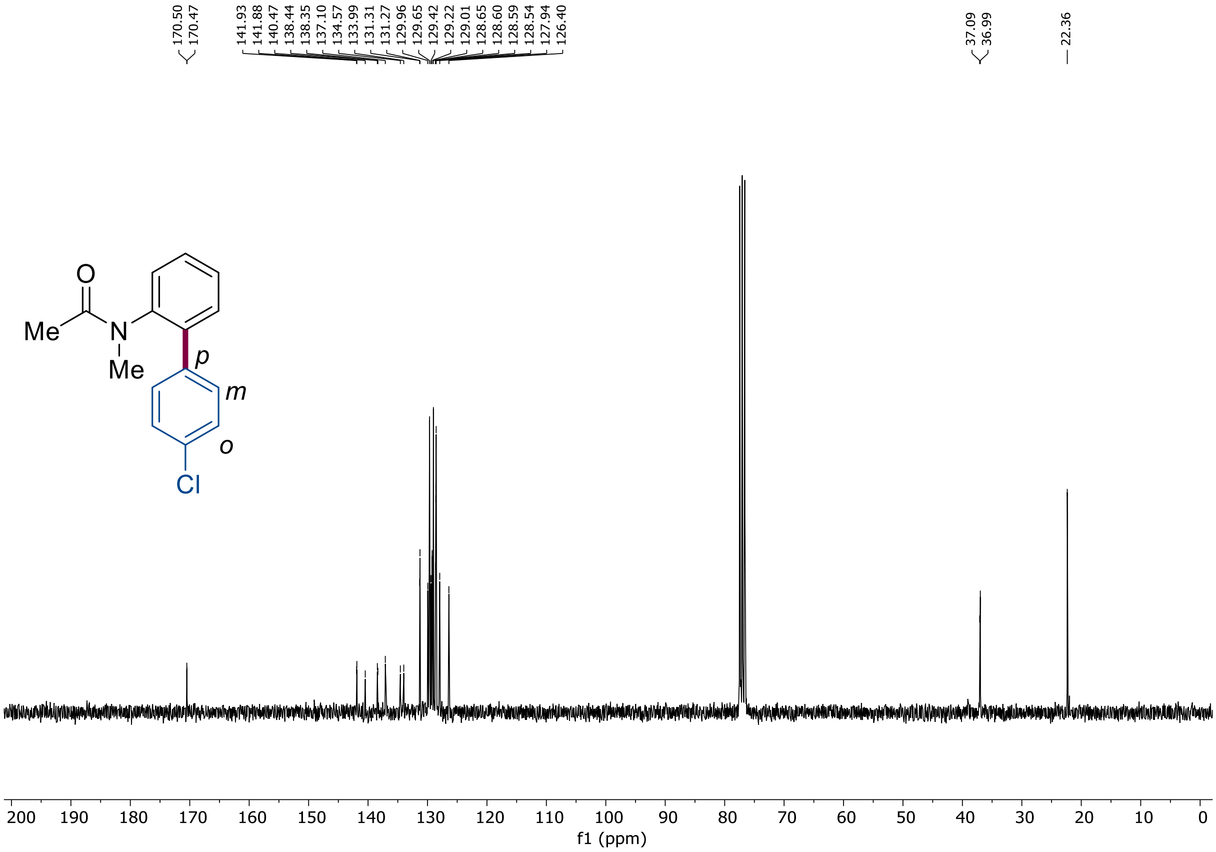

NMR Spectrum 120 ^{13}C NMR for 50, 75 MHz, $CDCl_3$, room temperature.

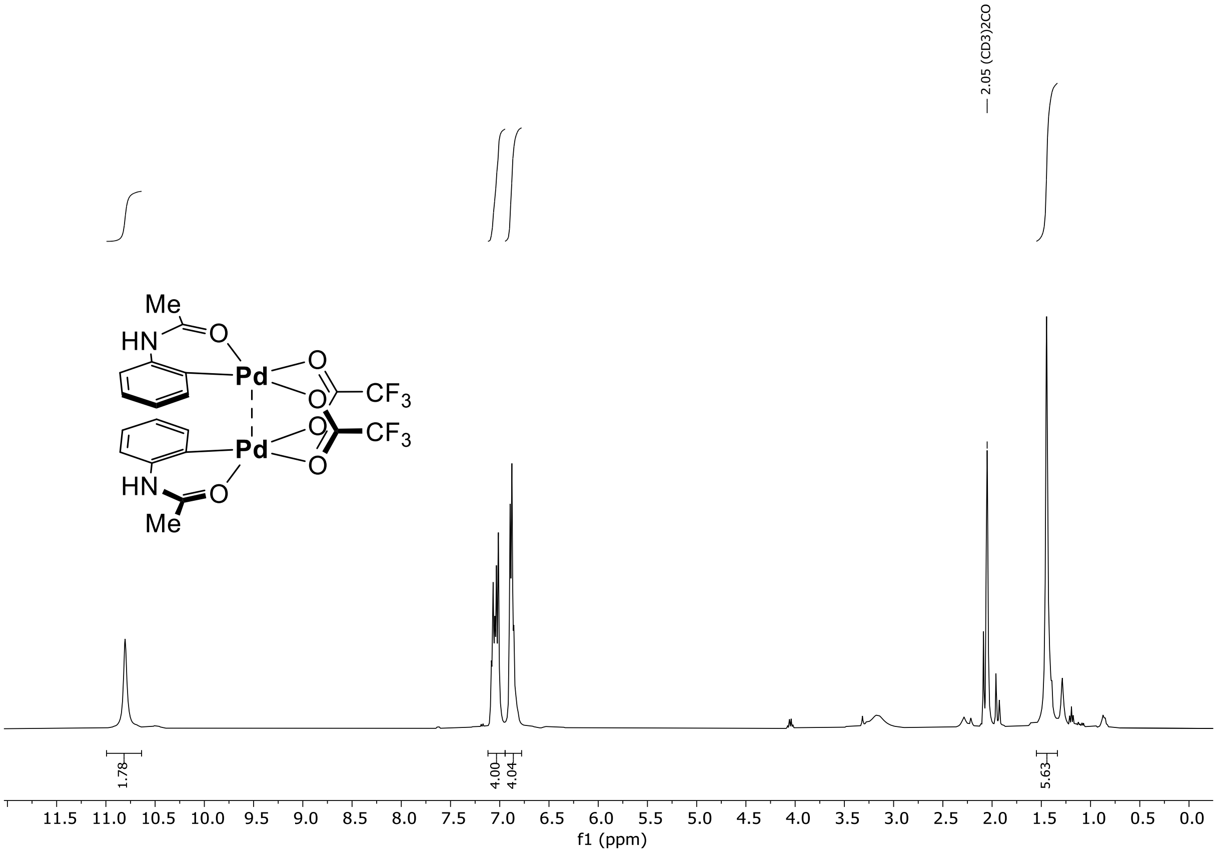

NMR Spectrum 121 ^{1}H NMR for 54, 400 MHz, acetone-d$_6$, room temperature.

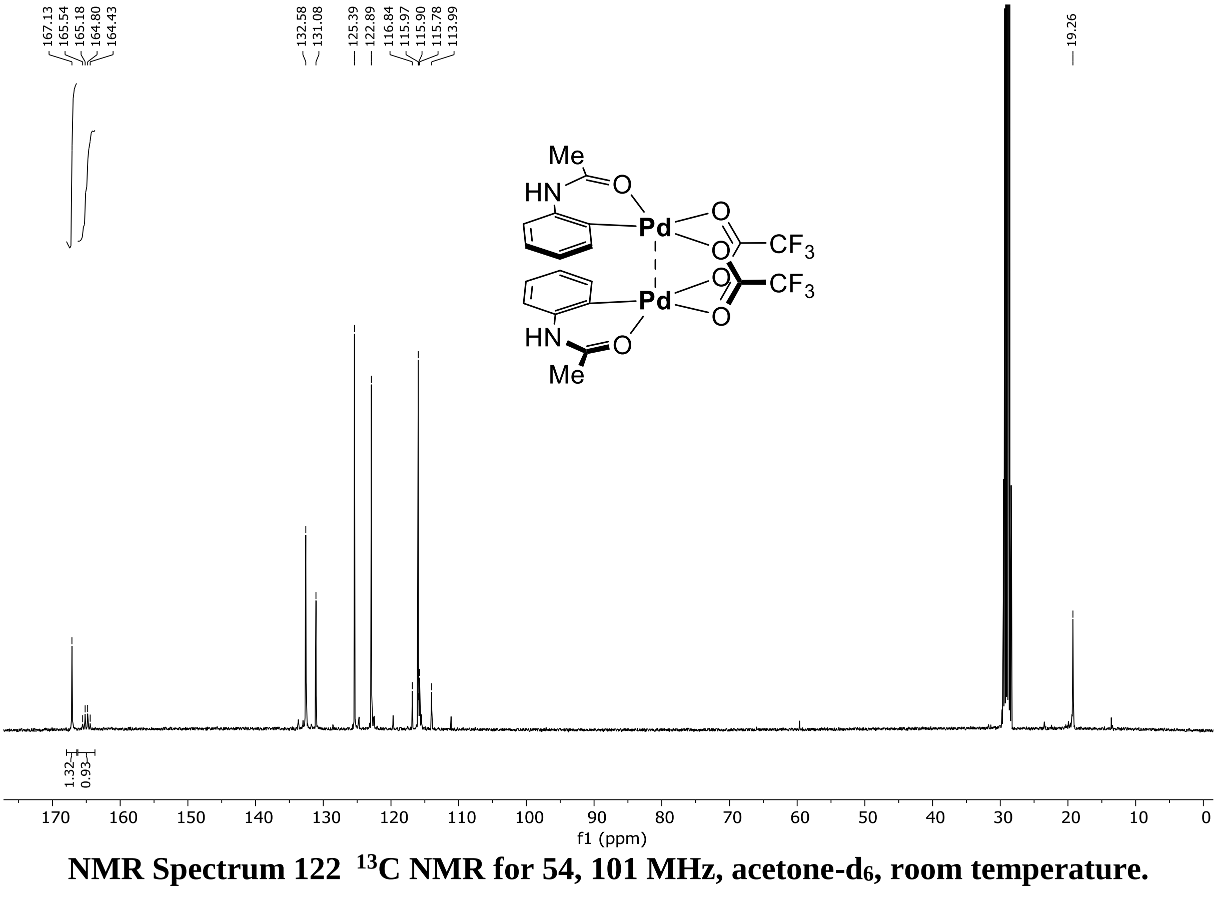

NMR Spectrum 122 ^{13}C NMR for 54, 101 MHz, acetone-d$_6$, room temperature.

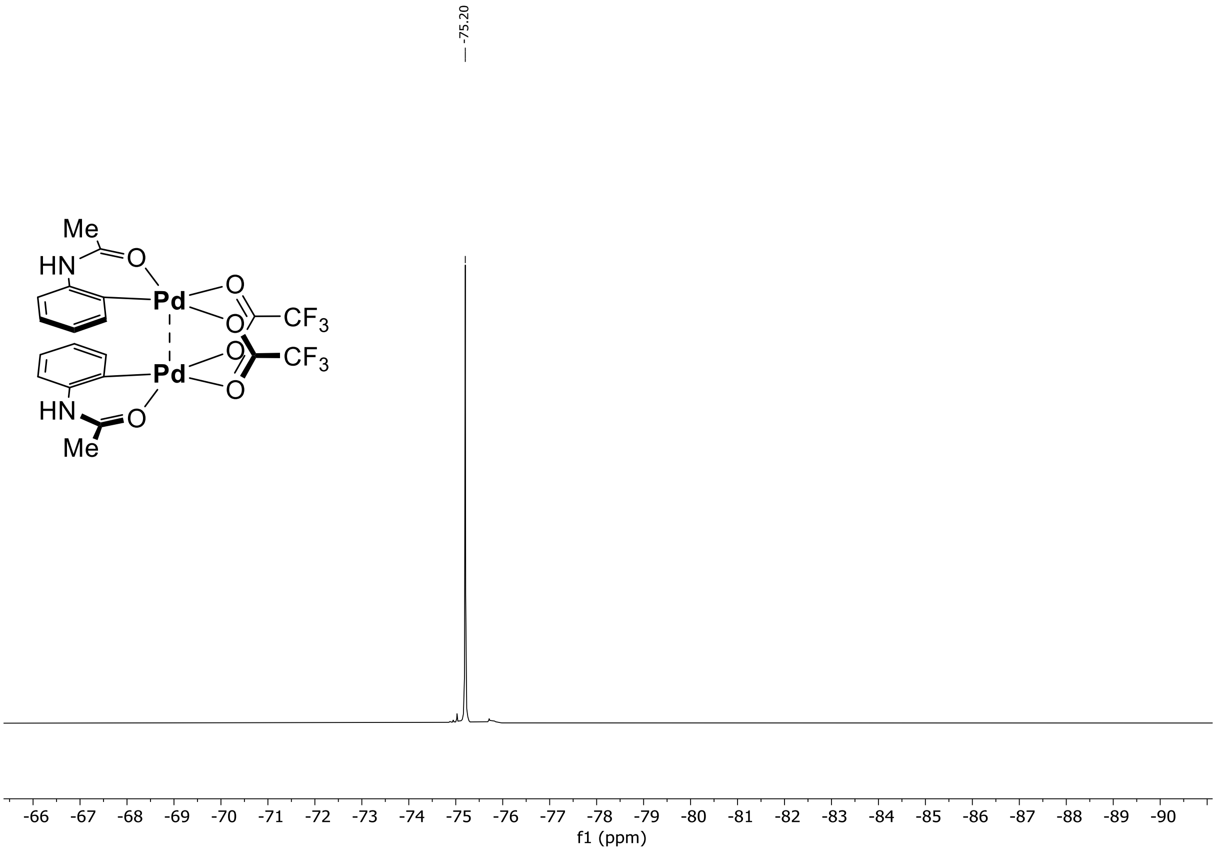

NMR Spectrum 123 ^{19}F NMR for 54, 377 MHz, acetone-d$_6$, room temperature.

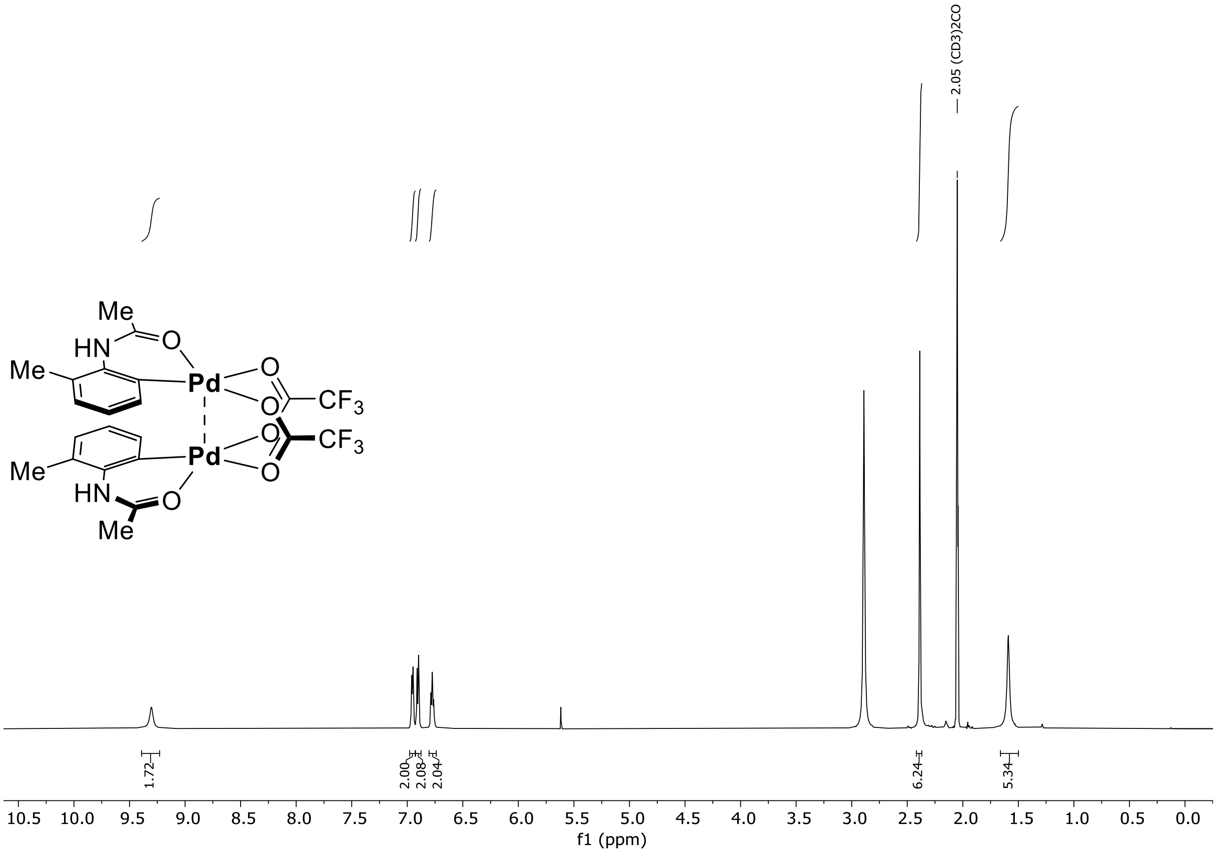

NMR Spectrum 124 ^{1}H NMR for 55, 600 MHz, acetone-d$_6$, room temperature.

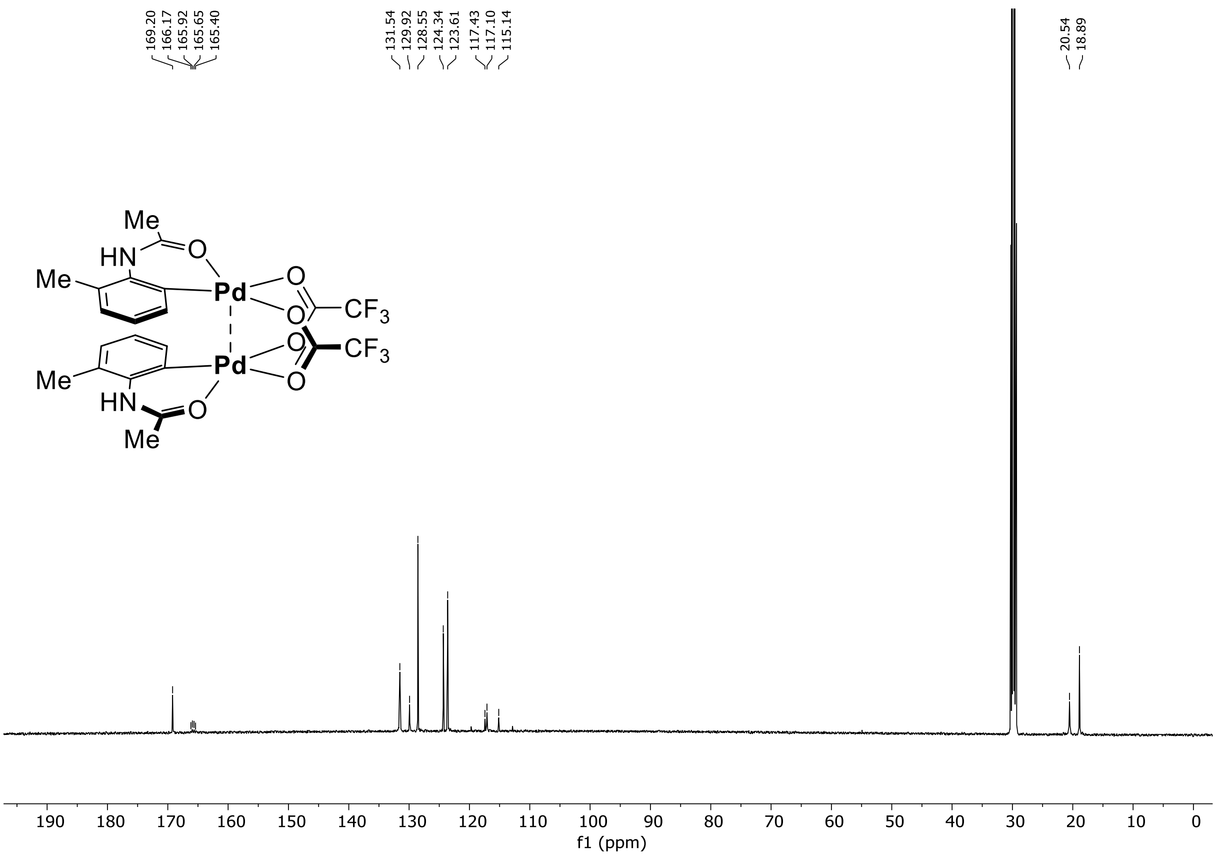

NMR Spectrum 125 ^{13}C NMR for 55, 126 MHz, acetone-d$_6$, room temperature.

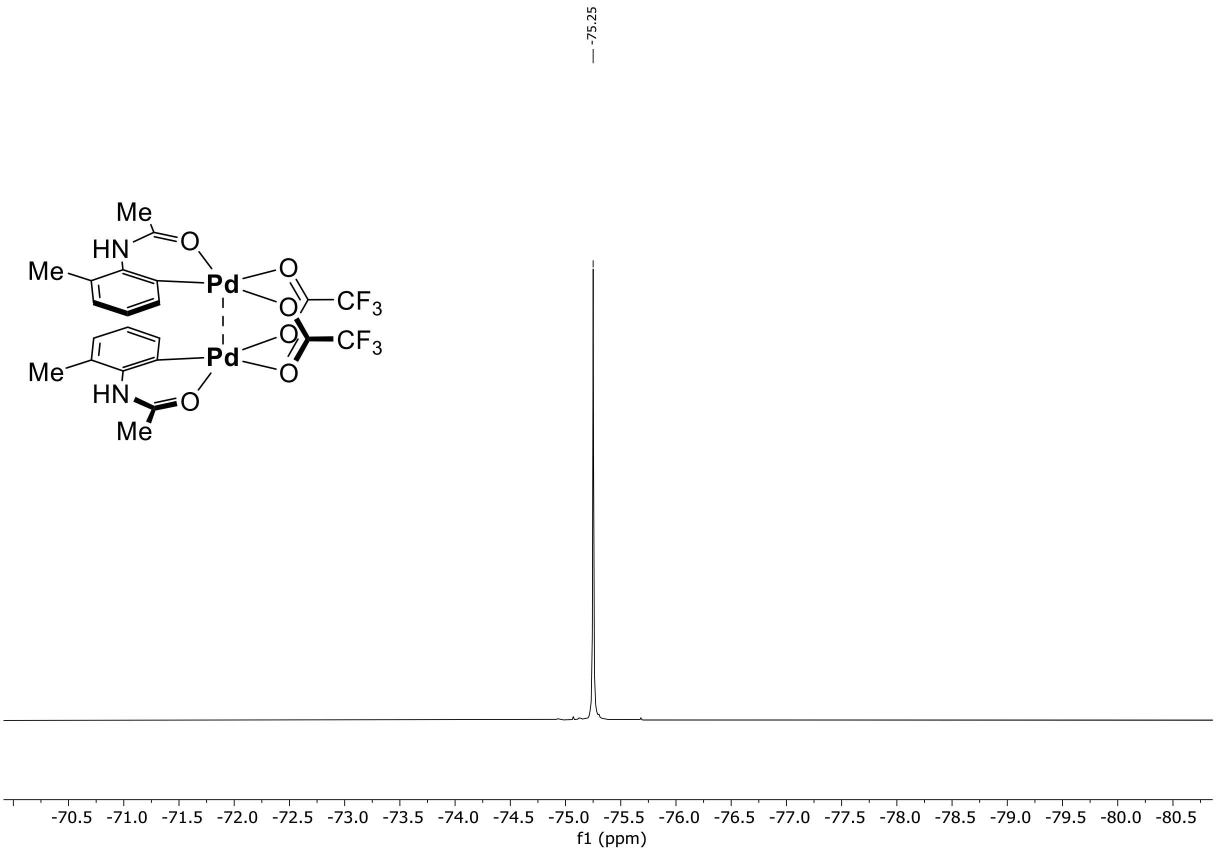

NMR Spectrum 126 ^{19}F NMR for 55, 471 MHz, acetone-d$_6$, room temperature.

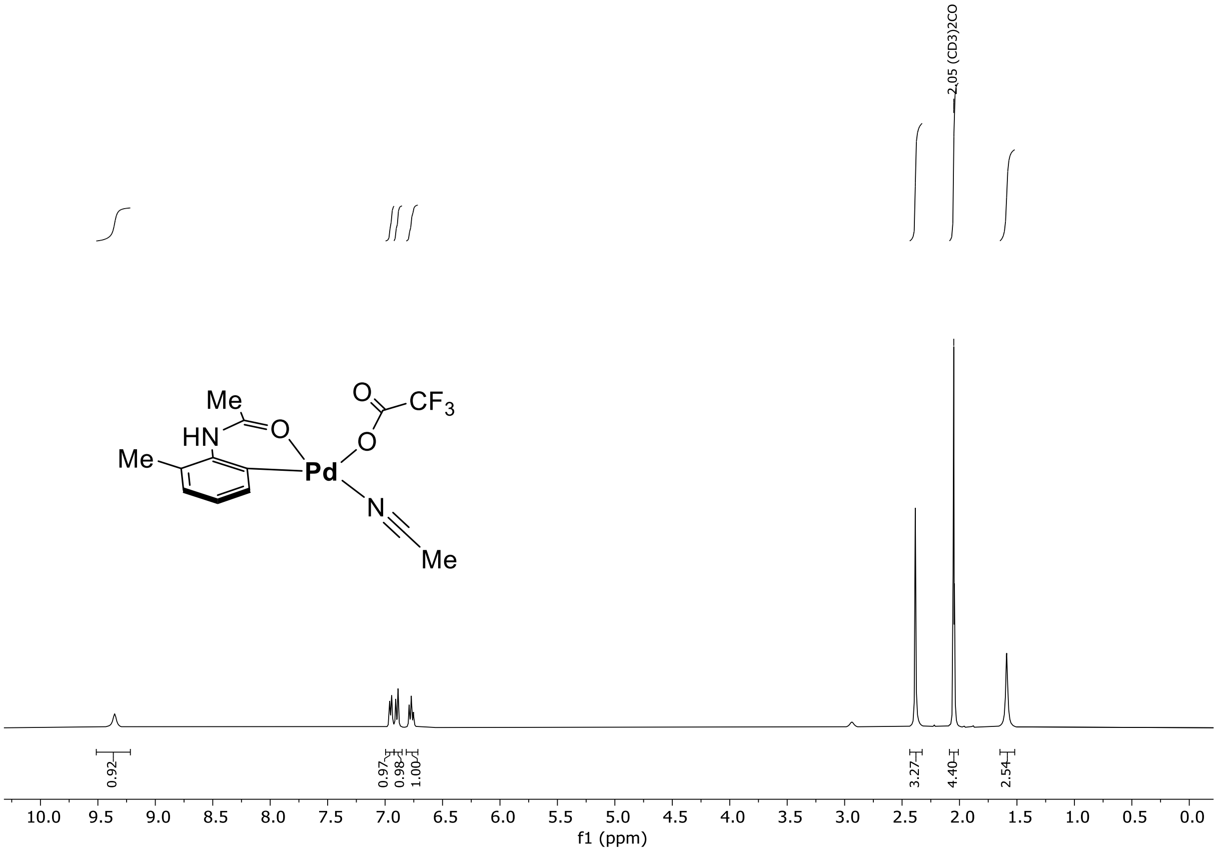

NMR Spectrum 127 ^{1}H NMR for 56, 400 MHz, acetone-d_6, room temperature.

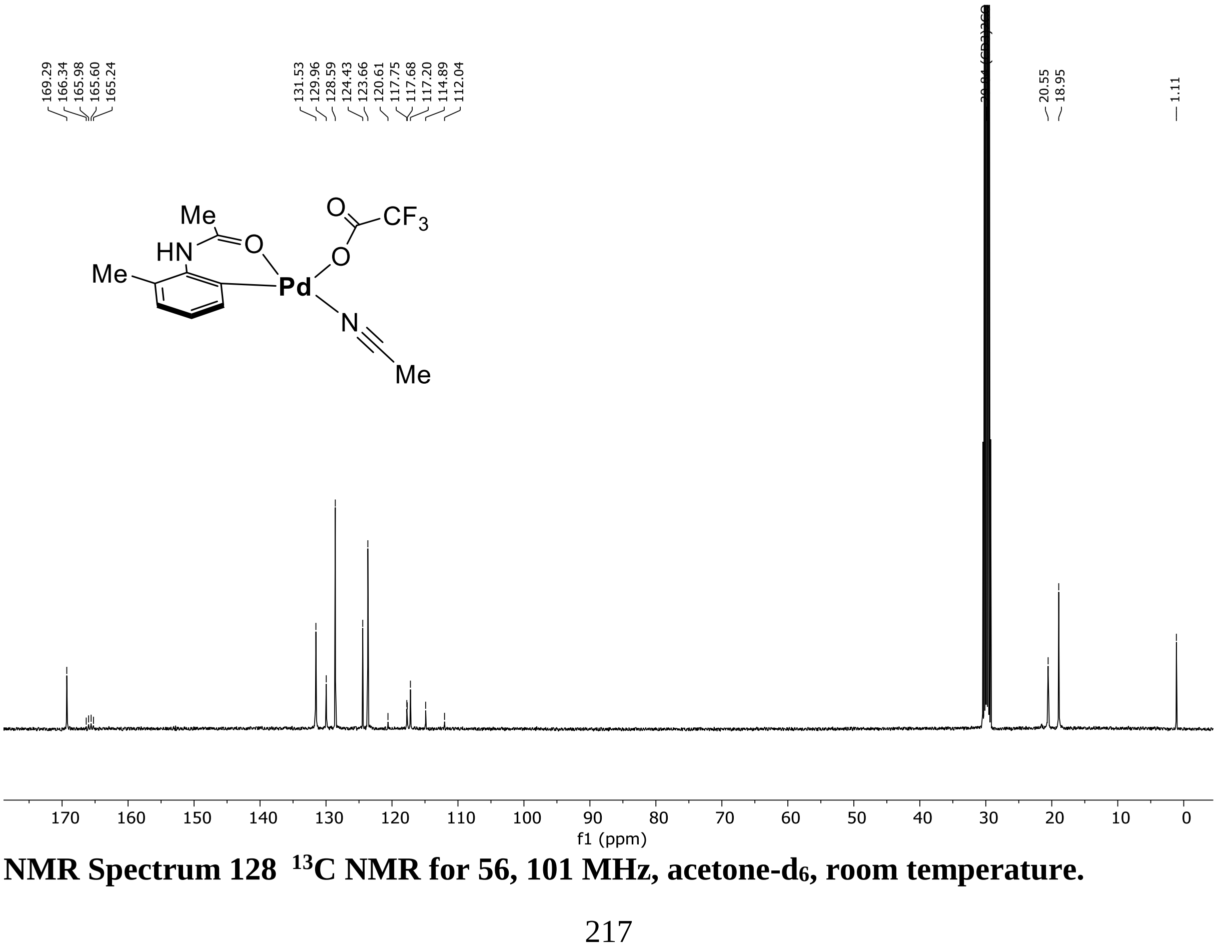

NMR Spectrum 128 ^{13}C NMR for 56, 101 MHz, acetone-d_6, room temperature.

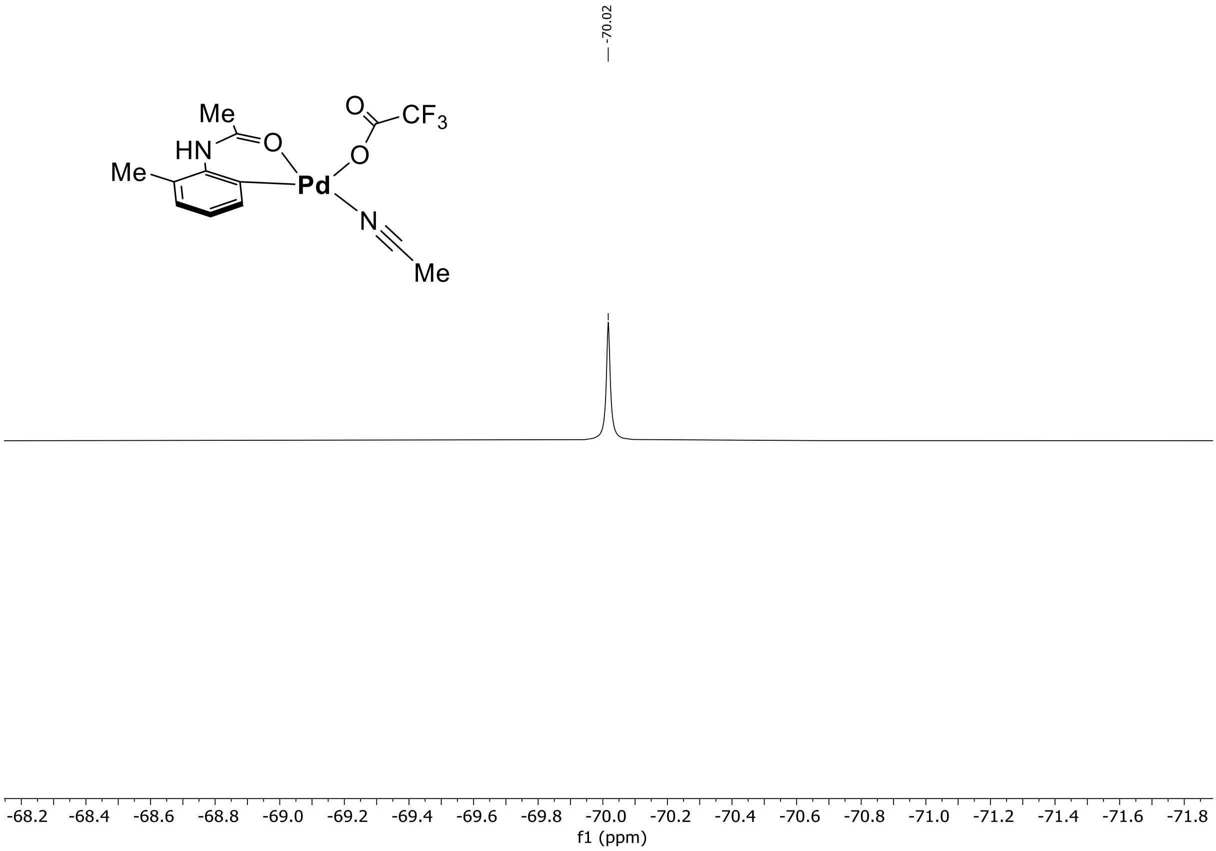

NMR Spectrum 129 ^{19}F NMR for 56, 377 MHz, acetone-d$_6$, room temperature.

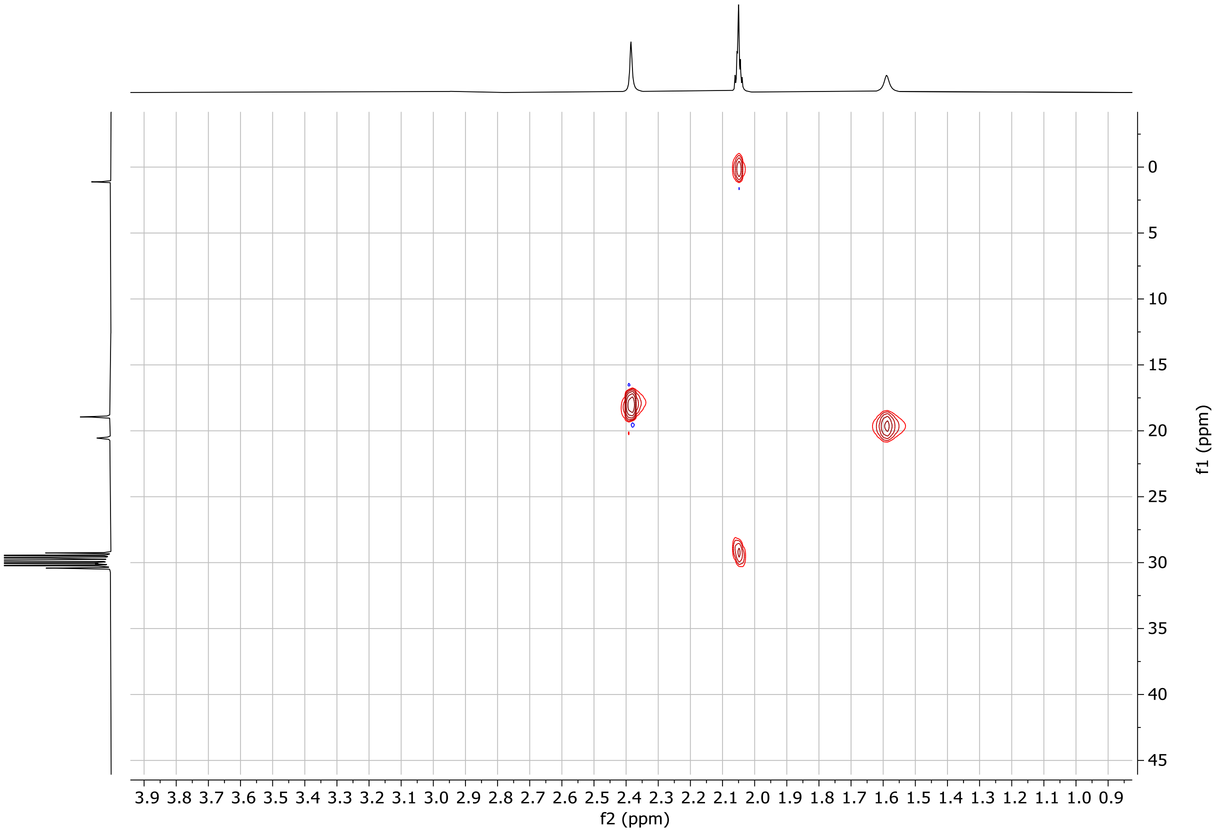

NMR Spectrum 130 HSQC NMR for 56, acetone-d$_6$, room temperature.

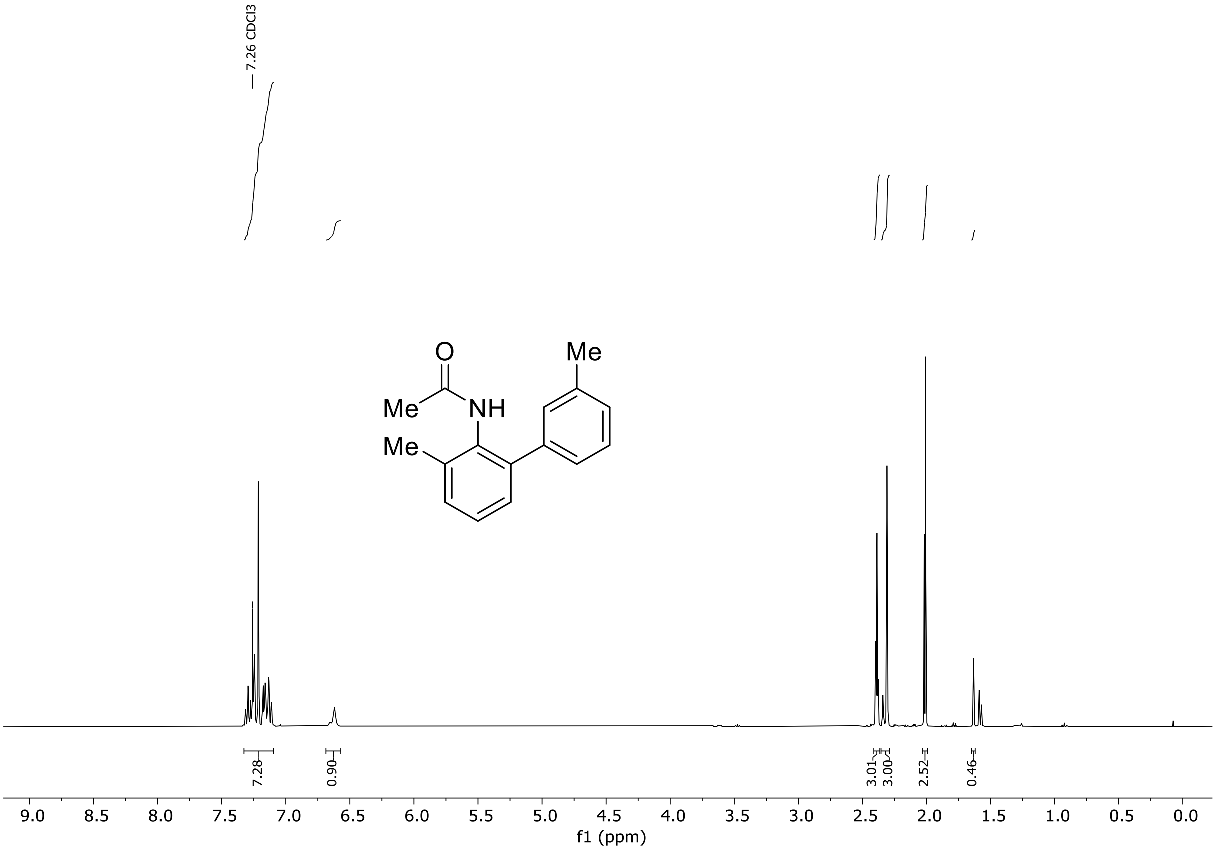

NMR Spectrum 131 ^{1}H NMR for 57, 400 MHz, $CDCl_3$, room temperature.

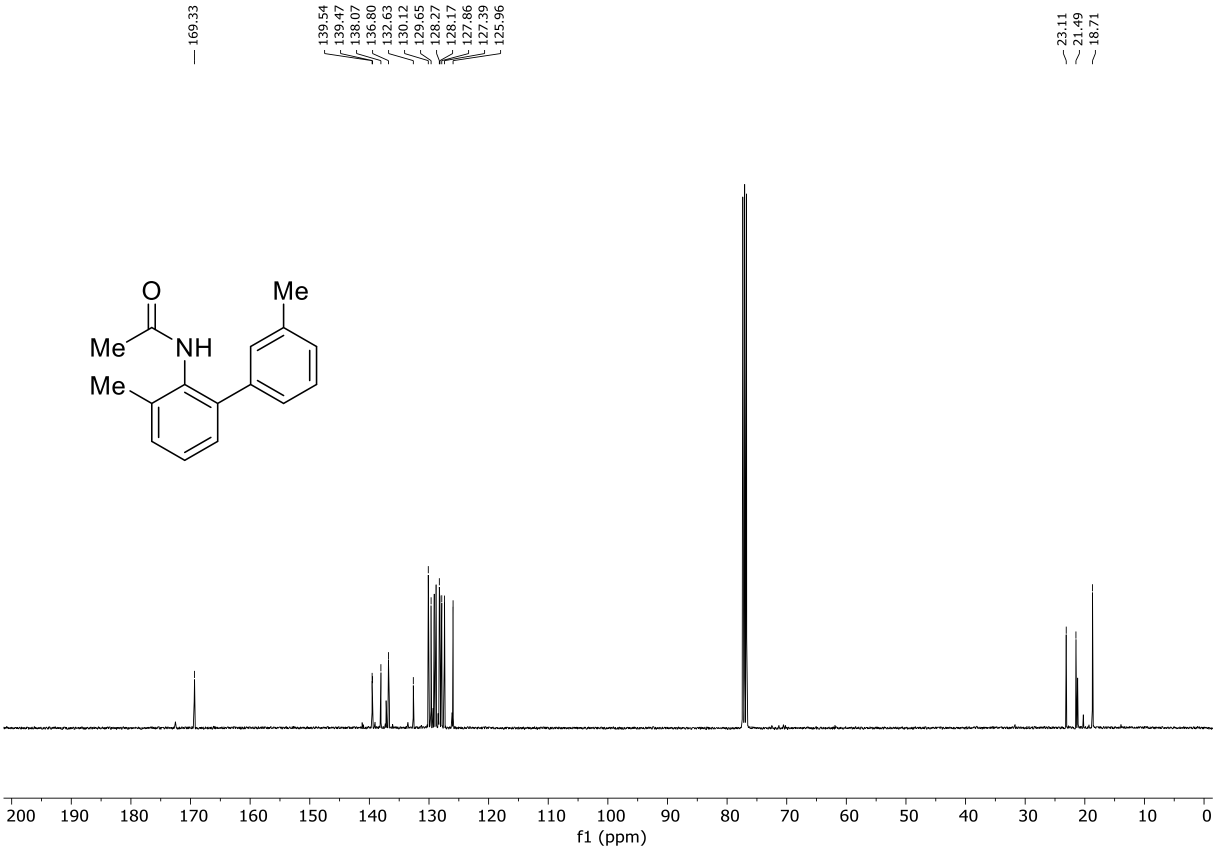

NMR Spectrum 132 ^{1}H NMR for 57, 400 MHz, $CDCl_3$, room temperature.

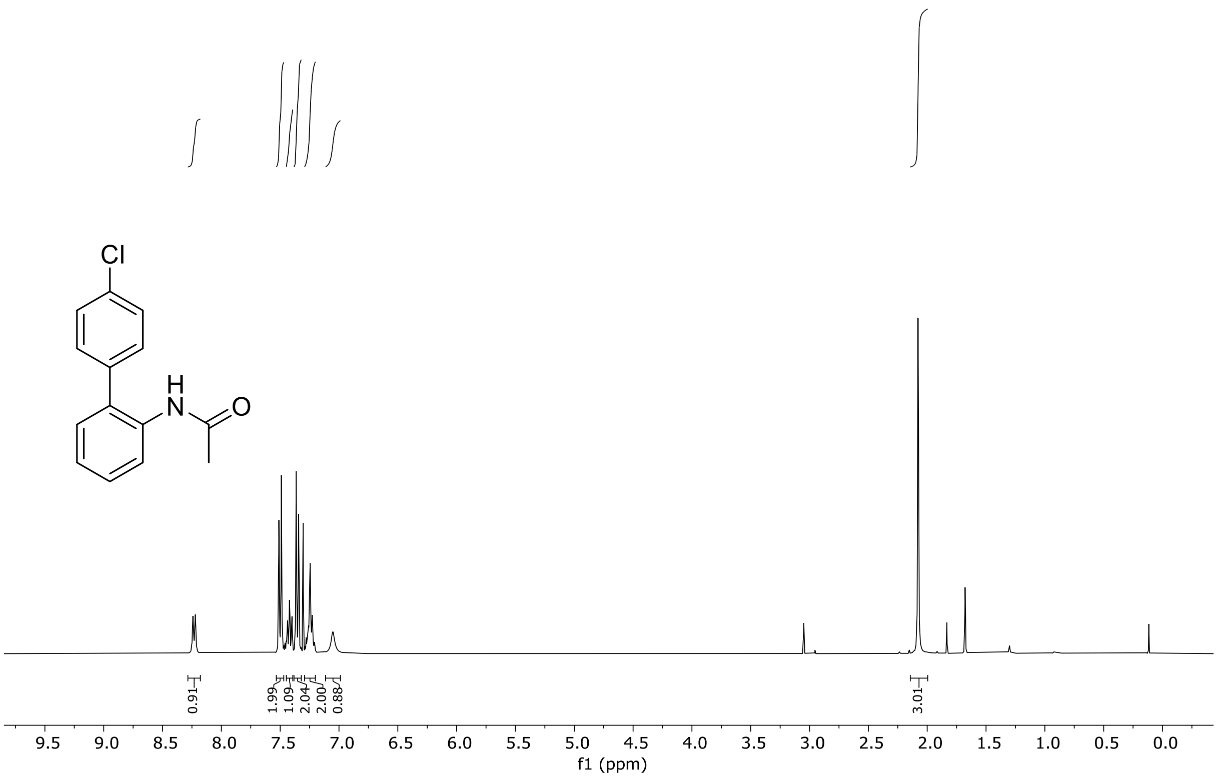

NMR Spectrum 133 ^{1}H NMR for s1, 400 MHz, $CDCl_3$, room temperature.

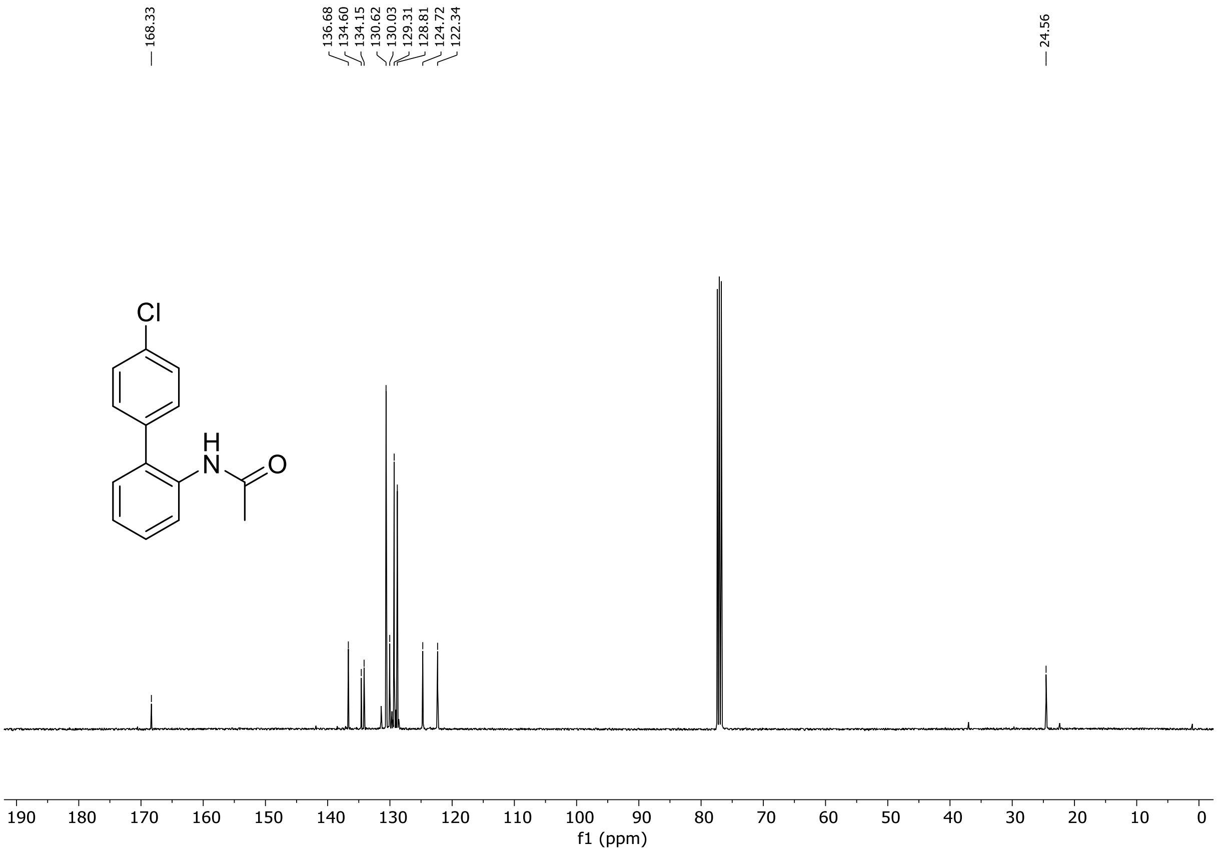

NMR Spectrum 134 ^{13}C NMR for s1, 101 MHz, $CDCl_3$, room temperature.

14 Reference

(1) Yin, J.; Buchwald, S. L. Pd-Catalyzed Intermolecular Amidation of Aryl Halides: The Discovery that Xantphos Can Be Trans-Chelating in a Palladium Complex. *J. Am. Chem. Soc.* **2002**, *124*, 6043-6048.

(2) Kim, B. S.; Jang, C.; Lee, D. J.; Youn, S. W. Highly Effective Pd-Catalyzed *ortho* Olefination of Acetanilides: Broad Substrate Scope and High Tolerability. *Chem Asian J.* **2010**, *5*, 2336-2340.

(3) van den Nieuwendijk, A. M. C. H.; Pietra, D.; Heitman, L.; Göblyös, A.; Ijzerman, A. P. Synthesis and Biological Evaluation of 2,3,5-Substituted [1,2,4]Thiadiazoles as Allosteric Modulators of Adenosine Receptors. *J. Med. Chem.* **2004**, *47*, 663-672.

(4) Yeung, C. S.; Zhao, X.; Borduas, N.; Dong, V. M. Pd-catalyzed *ortho*-arylation of phenylacetamides, benzamides, and anilides with simple arenes using sodium persulfate. *Chem. Sci.* **2010**, *1*, 331-336.

(5) Rauf, W.; Thompson, A. L.; Brown, J. M. Anilide activation of adjacent C–H bonds in the palladium-catalysed Fujiwara–Moritani reaction. *Dalton Trans.* **2010**, *39*, 10414-10421.

(6) *Gaussian 16 Rev. A.03*; Wallingford, CT, 2016.

(7) (a) Becke, A. D. Density‐functional thermochemistry. III. The role of exact exchange. *J. Chem. Phys.* **1993**, *98*, 5648-5652. (b) Lee, C.; Yang, W.; Parr, R. G. Development of the Colle-Salvetti correlation-energy formula into a functional of the electron density. *Phys. Rev. B* **1988**, *37*, 785-789.

(8) (a) Grimme, S.; Ehrlich, S.; Goerigk, L. Effect of the damping function in dispersion corrected density functional theory. *J. Comput. Chem.* **2011**, *32*, 1456-1465. (b) Grimme, S.; Antony, J.; Ehrlich, S.; Krieg, H. A consistent and accurate ab initio parametrization of density functional dispersion correction (DFT-D) for the 94 elements H-Pu. *J. Chem. Phys.* **2010**, *132*, 154104.

(9) (a) Hay, P. J.; Wadt, W. R. Ab initio effective core potentials for molecular calculations. Potentials for the transition metal atoms Sc to Hg. *J. Chem. Phys.* **1985**, *82*, 270-283. (b) Wadt, W. R.; Hay, P. J. Ab initio effective core potentials for molecular calculations. Potentials for main group elements Na to Bi. *J. Chem. Phys.* **1985**, *82*, 284-298. (c) Hay, P. J.; Wadt, W. R. Ab initio effective core potentials for molecular calculations. Potentials for K to Au including the outermost core orbitals. *J. Chem. Phys.* **1985**, *82*, 299-310.

(10) (a) Caldeweyher, E.; Ehlert, S.; Hansen, A.; Neugebauer, H.; Spicher, S.; Bannwarth, C.; Grimme, S. A generally applicable atomic-charge dependent London dispersion correction. *J. Chem. Phys.* **2019**, *150*, 154122. (b) Caldeweyher, E.; Bannwarth, C.; Grimme, S. Extension of the D3 dispersion coefficient model. *J. Chem. Phys.* **2017**, *147*, 034112.

(11) (a) Martin, J. M. L.; Sundermann, A. Correlation consistent valence basis sets for use with the Stuttgart–Dresden–Bonn relativistic effective core potentials: The atoms Ga–Kr and In–Xe. *J. Chem. Phys.* **2001**, *114*, 3408-3420. (b) Andrae, D.; Häußermann, U.; Dolg, M.; Stoll, H.; Preuß, H. Energy-adjustedab initio pseudopotentials for the second and third row transition elements. *Theor. Chim. Acta* **1990**, *77*, 123-141.

(12) Marenich, A. V.; Cramer, C. J.; Truhlar, D. G. Universal Solvation Model Based on Solute Electron Density and on a Continuum Model of the Solvent Defined by the Bulk Dielectric Constant and Atomic Surface Tensions. *J. Phys. Chem. B* **2009**, *113,* 6378-6396.

(13) Simlandy, A. K.; Rodphon, W.; Alturaifi, T. M.; Mai, B. K.; Ni, H.-Q.; Gurak, J. A., Jr.; Liu, P.; Engle, K. M. Catalytic Addition of Nitroalkanes to Unactivated Alkenes via Directed Carbopalladation. *ACS Catal.* **2022**, *12*, 13755-13762.

(14) Nishikata, T.; Abela, A. R.; Huang, S.; Lipshutz, B. H. Cationic Pd(II)-catalyzed C–H activation/cross-coupling reactions at room temperature: synthetic and mechanistic studies. *Beilstein J. Org. Chem.* **2016**, *12*, 1040-1064.

(15) Teng, Q.-H.; Sun, G.-X.; Huang, F.-P.; Wang, K.; Liang, F.-P. Visible-light Induced Cerium-catalyzed N-Demethylation of N-Methyl Amides under Air Conditions. *Adv. Synth. Catal.* **2023**, *365*, 307-311.

(16) Li, B. J.; Tian, S. L.; Fang, Z.; Shi, Z. J. Multiple C–H activations to construct biologically active molecules in a process completely free of organohalogen and organometallic components. *Angew. Chem. Int. Ed.* **2008**, *47*, 1115-1118.

(17) Zhu, R.; Lu, S.; Wang, Q.; Bai, J.; Wang, Y.; Yu, Q.; Huang, J. Selectfluor-mediated mono-C–H activation: The syntheses of mono-ortho-substituted anilides. *Tetrahedron* **2018**, *74*, 3879-3887.

(18) (a) Kitagawa, O.; Yoshikawa, M.; Tanabe, H.; Morita, T.; Takahashi, M.; Dobashi, Y.; Taguchi, T. Highly Enantioselective Synthesis of Atropisomeric Anilide Derivatives through Catalytic Asymmetric *N*-Arylation: Conformational Analysis and Application to Asymmetric Enolate Chemistry. *J. Am. Chem. Soc.* **2006**, *128*, 12923-12931. (b) Curran, D. P.; Hale, G. R.; Geib, S. J.; Balog, A.; Cass, Q. B.; Degani, A. L. G.; Hernandes, M. Z.; Freitas, L. C. G. Rotational features of carbon-nitrogen bonds in axially chiral *o-tert*-butyl anilides and related molecules. Potential substrates for the 'prochiral auxiliary' approach to asymmetric synthesis. *Tetrahedron: Asymmetry* **1997**, *8*, 3955-3975. (c) Masataka, M.; Kazuko, K.; Atsushi, K.; Yohei, H.; Makiko, S. Kinetic Studies of Fast Equilibrium by Means of High-performance Liquid Chromatography. X. Separation of Rotamers of Acetanilides. *Bull. Chem. Soc. Jpn.* **1985**, *58*, 2581-2585.

(19) Li, J.; Ackermann, L. Cobalt-Catalyzed C–H Arylations with Weakly-Coordinating Amides and Tetrazoles: Expedient Route to Angiotensin-II-Receptor Blockers. *Chem. Eur. J.* **2015**, *21*, 5718-5722.

(20) Shi, Z.; Li, B.; Wan, X.; Cheng, J.; Fang, Z.; Cao, B.; Qin, C.; Wang, Y. Suzuki-Miyaura Coupling Reaction by Pd^{II}-Catalyzed Aromatic C–H Bond Activation Directed by an *N*-Alkyl Acetamino Group. *Angew. Chem. Int. Ed.* **2007**, *46*, 5554-5558.

(21) Tischler, O.; Bokányi, Z.; Novák, Z. Activation of C–H Activation: The Beneficial Effect of Catalytic Amount of Triaryl Boranes on Palladium-Catalyzed C–H Activation. *Organometallics* **2016**, *35*, 741-746.

(22) Tóth, B. L.; Kovács, S.; Sályi, G.; Novák, Z. Mild and Efficient Palladium-Catalyzed Direct Trifluoroethylation of Aromatic Systems by C−H Activation. *Angew. Chem. Int. Ed.* **2016**, *55*, 1988-1992.
